# Comprehensive tumor molecular profile analysis in clinical practice

**DOI:** 10.1186/s12920-021-00952-9

**Published:** 2021-04-14

**Authors:** Mustafa Özdoğan, Eirini Papadopoulou, Nikolaos Tsoulos, Aikaterini Tsantikidi, Vasiliki-Metaxa Mariatou, Georgios Tsaousis, Evgenia Kapeni, Evgenia Bourkoula, Dimitrios Fotiou, Georgios Kapetsis, Ioannis Boukovinas, Nikolaos Touroutoglou, Athanasios Fassas, Achilleas Adamidis, Paraskevas Kosmidis, Dimitrios Trafalis, Eleni Galani, George Lypas, Bülent Orhan, Sualp Tansan, Tahsin Özatlı, Onder Kırca, Okan Çakır, George Nasioulas

**Affiliations:** 1 Division of Medical Oncology, Memorial Hospital, Antalya, Turkey; 2 Genekor Medical S.A, Athens, Greece; 3 Bioclinic Thessaloniki, Thessaloníki, Greece; 4 Department of Medical Oncology, Interbalkan Medical Center, Thessaloníki, Greece; 5 St. Luke’s Hospital, Thessaloníki, Greece; 6 Second Department of Medical Oncology, Hygeia Hospital, Athens, Greece; 7 Henry Dunant Hospital Center, Athina, Greece; 8 Second Department of Medical Oncology, “Metropolitan” Hospital, Piraeus, Greece; 9 Department of Genetic Oncology/Medical Oncology, Hygeia Hospital, Athens, Greece; 10 Department of Medical Oncology, Ceylan International Hospital, Bursa, Turkey; 11 Tansan Oncology, Istanbul, Turkey; 12 Istinye University Hospital, Istanbul, Turkey; 13 Applied Health Sciences, Edinburgh Napier University, Edinburgh, EH11 4BN Scotland, UK

**Keywords:** Molecular profile, Next Generation Sequencing, Targeted treatment, Immunotherapy, Tumor mutation burden, PD-L1, Microsatellite instability

## Abstract

**Background:**

Tumor molecular profile analysis by Next Generation Sequencing technology is currently widely applied in clinical practice and has enabled the detection of predictive biomarkers of response to targeted treatment. In parallel with targeted therapies, immunotherapies are also evolving, revolutionizing cancer therapy, with Programmed Death-ligand 1 (PD-L1), Microsatellite instability (MSI), and Tumor Mutational Burden (TMB) analysis being the biomarkers employed most commonly.

**Methods:**

In the present study, tumor molecular profile analysis was performed using a 161 gene NGS panel, containing the majority of clinically significant genes for cancer treatment selection. A variety of tumor types have been analyzed, including aggressive and hard to treat cancers such as pancreatic cancer. Besides, the clinical utility of immunotherapy biomarkers (TMB, MSI, PD-L1), was also studied.

**Results:**

Molecular profile analysis was conducted in 610 cancer patients, while in 393 of them a at least one biomarker for immunotherapy response was requested. An actionable alteration was detected in 77.87% of the patients. 54.75% of them received information related to on-label or off-label treatment (Tiers 1A.1, 1A.2, 2B, and 2C.1) and 21.31% received a variant that could be used for clinical trial inclusion. The addition to immunotherapy biomarker to targeted biomarkers’ analysis in 191 cases increased the number of patients with an on-label treatment recommendation by 22.92%, while an option for on-label or off-label treatment was provided in 71.35% of the cases.

**Conclusions:**

Tumor molecular profile analysis using NGS is a first-tier method for a variety of tumor types and provides important information for decision making in the treatment of cancer patients. Importantly, simultaneous analysis for targeted therapy and immunotherapy biomarkers could lead to better tumor characterization and offer actionable information in the majority of patients. Furthermore, our data suggest that one in two patients may be eligible for on-label ICI treatment based on biomarker analysis. However, appropriate interpretation of results from such analysis is essential for implementation in clinical practice and accurate refinement of treatment strategy.

**Supplementary Information:**

The online version contains supplementary material available at 10.1186/s12920-021-00952-9.

## Background

In recent years, technological advances and active research have permitted extensive tumor molecular characterization and have revealed a variety of tumorigenic pathways presenting tumor-specific alterations. These distinctive molecular characteristics of cancer cells can be targeted as they represent the malignant cell’s Achille’s heel, without affecting the healthy ones. To this regard, of great importance was the previous knowledge gained by large scale studies that used various, advanced technologies to obtain a comprehensive understanding of the tumor molecular profile [1].

Tumor molecular profile is nowadays becoming a reality mainly due to the increased availability, with concomitant reduction of cost of the Next Generation Sequencing technology (NGS) technology. The term personalized medicine in anticancer treatment has emerged, indicating the need to treat each patient based on his/her tumor’s specific characteristics [2]. The individualization of treatment strategy entails the use of biomarkers that are those quantifiable characteristics that can be related to cancer prognosis and prediction of treatment response [2–4].

Currently, NGS analysis of more than 35 genes is mandatory for approved targeted treatment selection in several neoplasias [4]. Furthermore, over 200 ongoing clinical trials are investigating the clinical utility of novel biomarkers, leading to additional biomarker approval each year (www.clinicaltrials.org). Various studies have also shown the clinical benefit obtained using gene-directed treatment in comparison to unselected treatment assignment for patients with metastatic tumors [5–7]. Thus, the abundance of treatments with approved gene targets available alongside the often low tissue availability, entails the simultaneous analysis of biomarkers using multigene panels for various tumor types such as lung cancer, colorectal, gastrointestinal, ovarian, breast prostate cancer and others [3, 4, 8]. Besides, tumor agnostic therapies approvals, with biomarkers associated independently form tumor type, have also boosted the number of genes that should be analyzed when targeted treatment is considered [9]. It is thus apparent that in the era of personalized treatment, single or few gene analysis is no longer recommended, leading to missing treatment options for cancer patients.

Moreover, even in the absence of biomarkers associated with on-label treatments, a broad molecular profile analysis could lead to the detection of an approved biomarker for a different tumor type, giving the option of off-label treatment selection or enrolment in an ongoing clinical trial. To this regard, the contribution of active research for the identification of actionable alterations is enormous and has led to the discovery of new agents targeting genes previously known for their important role in oncogenesis but without predictive utility. A major paradigm of such gene is KRAS that has been considered for decades not targetable, while recent studies have shown that certain frequently detected KRAS alterations, such as the G12C mutation, can be targeted efficiently leading to an eventual upcoming FDA approval of new investigational treatments showing efficiency at this regard [10–12].

The number of laboratories applying high throughput sequencing analysis is continuously increasing, in parallel with the increased request by the clinicians for such analysis. The frequently insufficient amount of good quality tissue specimen, coupled with the increasing number of approved targeted agents, make the simultaneous analysis of multiple biomarkers using multigene panels imperative. Thus, advanced technology solves one of the most significant limitations of tissue testing. It is nevertheless noteworthy that, the optimal paraffin embedding procedure remains crucial for obtaining accurate NGS results [13, 14].

Currently, in parallel with targeted therapies, an increasing armamentarium of immunotherapy agents is also emerging, revolutionizing cancer therapy. The high cost and toxicity that often accompanies immunotherapeutic agents mandate the use of appropriate biomarkers for selecting patients more likely to benefit from them.

The most widely used biomarker is currently PD-L1 expression, assessed by Immunohistochemistry (IHC) [15]. However, it is well known that this is not an ideal biomarker since it is not related to treatment response in many tumor types, while it is clearly not the sole predictor of response to check point inhibition. Moreover, even for those tumors with a proven utility for PDL-1 IHC testing, such as lung cancer, several questions regarding methodology and cut offs remain [15, 16].

Additionally, microsatellite instability (MSI) has also been associated with response to anti-PD-L1 treatment with pembrolizumab receiving approval for MSI-H tumors [17–19]. Of note, MSI was the first tumor agnostic biomarker that had ever shown efficiency regardless of tumor type. However, the presence of MSI varies among tumor types with the rate of MSI-H tumors ranging from 10 to 15% for colon cancer to 0% in others such as lung cancer [20]. Thus, still, the majority of responders will not be identified by it. Hence, the enrichment of biomarkers for the identification of patients eligible for immunotherapy administration is required.

Several additional biomarkers of immune response have been proposed and are currently under investigation while it seems that their combined use could increase the predictive value of the information obtained [21, 22]. Among the most studied ones is the analysis of Tumor Mutational Burden (TMB) that measures the number of somatic mutations present in a tumor sample. It has been shown in several studies and clinical trials that the greater the number of somatic alterations identified the greater the probability of response to immune treatment [23–25]. It has been reported that TMB cutoff values associated with improved survival from immunotherapy treatment vary significantly between cancer types [25]. Nevertheless, in the majority of studies and clinical trials, a cut off of 10 muts/MB is used [26–28]. Furthermore, the clinical utility of TMB as a predictive biomarker for anti-PD1 treatment administration has been shown in the KEYNOTE 158 study leading to the tumor agnostic approval by the USA FDA of pembrolizumab for metastatic untreatable solid tumors with tissue TMB value of ≥ 10 muts/MB [29].

The present study aimed to reveal the applicability and utility of tumor profile analysis in clinical practice, using a pan-cancer NGS panel for cancer treatment selection. The panel used in this study analyses 161 single genes using the Oncomine Technology (Thermo Fischer Scientific) and was selected based on the amount of actionable information contained, the robustness of the assay and its relatively low cost, which enables its use in clinical practice. A variety of tumor types have been analyzed, including aggressive and hard to treat cancers such as pancreatic cancer. Moreover, the clinical utility of immunotherapy biomarkers (TMB, MSI, PD-L1) was also explored.

## Methods

### Patients

In the present study, 629 cancer patients were referred by their treating oncologist for extensive molecular profile analysis from November 2017 to April 2020. All patients participating in the study provided written informed consent. Information concerning sex, age, and tumor histology was accessible, while the pathology report was available in all cases. In addition to molecular analysis for targeted treatment selection, analysis for at least one immunotherapy biomarker (PDL-1, MSI, TMB) was also requested in 395 patients. The analysis was performed using the most recent tissue specimen available.

### Tissue selection and nucleic acid isolation

Genomic DNA and RNA were isolated from formalin-fixed and paraffin-embedded (FFPE) tumor biopsies using the MagMAX™ Total Nucleic Acid Isolation Kit (Thermo Fischer Scientific) according to the Manufacturer’s instructions. The nucleic acid isolation was conducted in the areas of the FFPE block with the majority of tumor cell content (TCC), as indicated by experienced pathologists in Hematoxylin and eosin-stained sections. Minimum required TCC was over 20%, in a tumor area of > 4mm^2^.

### Next Generation Sequencing

Whenever tumor molecular profile analysis for targeted therapies was requested, the Oncomine Comprehensive Assay v3 (OCAv3) (Thermo Fischer Scientific) was performed, which is an amplicon based targeted NGS assay. This assay allows the identification of various mutation types such as Single nucleotide Variants (SNVs), insertion-deletions (indels), Copy Number Variations (CNVs), and gene fusions, from 161 unique genes. Run metrics were accessed in the Torrent Suite™ software, using the coverage analysis plugin v5.0.4.0. NGS data analysis was completed with the Ion Reporter™ 5.10.1.0 software (Thermo Fisher Scientific) using the manufacturer’s provided workflow (Oncomine Comprehensive v3-w3.2-DNA and Fusions-Single Sample). Furthermore, the analysis software Sequence Pilot (version 4.3.0, JSI medical systems, Ettenheim, Germany) was used for variant annotation. Sequencing data were aligned against the human reference assembly GRCh37/hg19.

Tumor Mutational Burden analysis was carried out using the Oncomine Tumor Mutation Load Assay (Thermo Fischer Scientific). This is a targeted NGS assay, with 1.65 MB of genomic coverage (1.2 MB exonic) that analyzes 409 genes to provide accurate quantitation of somatic mutations used for tumor mutation burden calculation, in FFPE tissues.

TMB was calculated using the Ion reporter pipeline that utilizes a custom variant calling and germline variant filtering to accurately calculate the number of exonic somatic mutations per MB (Oncomine Tumor Mutation Load-w2.0-DNA-Single Sample).

Microsatellite analysis was conducted using the Ion AmpliSeq™ Microsatellite Instability Panel (Thermo Fischer Scientific) which is an NGS based assay analyzing 76 markers to assess Microsatellite Instability (MSI) status in tumor-only and tumor-normal samples as indicated by the manufacturer. Analysis of the sequencing output from this panel was carried out using the “MSICall” plugin in the Torrent Suite.

### Variant classification

Variants were classified according to their predictive value using the four-tiered system jointly recommended by the Association for Molecular Pathology (AMP), the American College of Medical Genetics (ACMG), the American Society of Clinical Oncology (ASCO) and the College of American Pathologists (CAP) for the classification of somatic variants [30]. The Joint consensus recommendation system proposed by these major scientific institutions classifies the variants based on their clinical significance in 4 tiers 1–4. Tier 1 variants are of the most substantial clinical significance and are subdivided to those related to sensitivity or resistance to FDA approved treatments (Tier 1A.1), those proposed by professional guidelines to have predictive value (Tier 1A.2), and those with a strong consensus concerning their predictive significance (Tier 1B). The Tier 2 class involves biomarkers with potential clinical relevance. It can be subdivided in variants related to an approved treatment for a different tumor type (Tier 2C.1), variants related to investigational treatments that can be used as an inclusion criterion for patients’ enrollment in clinical trials (Tier 2C.1), and variants that have shown predictive value in preclinical studies (Tier 2D). Finally, the 3 and 4 Tiers, include biomarkers of unknown clinical significance and the benign or likely benign ones respectively [30, 31].

### Gene panel comparison

The clinical utility of the 161-gene panel used in this study was assessed by comparison with gene panels comprising a smaller number of genes. For this purpose, we selected two hotspot panels of 24 and 50 genes, respectively, that have been previously used both in our laboratory and in several publications for routine access to predictive biomarkers in various tumor types [32–36]. Thus, we compared the alterations that would have been detected in our cohort if the analysis was performed by these panels instead of the broader panel used. (Additional file 1: Table S1). Both smaller panels also included the analysis of 6 fusion driver genes (*ALK, ROS1, RET, NTRK1, NTRK2* and *NTRK3*) analyzed at the RNA level.

Furthermore, in order to investigate if the number of genes analyzed is adequate for implementation in clinical practice, or if by increasing the number of genes tested a more informative result could be retrieved, we compared the actionability of the results obtained from this panel to those obtained using a more comprehensive tumor panel that utilizes the same NGS technology. The panel implemented for this evaluation was the Oncomine Comprehensive plus assay (Thermo Fischer Scientific) that analyses the full coding sequence of 313 genes, hotspot analysis of 169 genes, CNV of 313 genes (most of them also analyzed for SNV and indels). Furthermore, it includes RNA analysis for 51 fusion driver genes (38 of them also analyzed at the DNA level), adding up to a total of 514 unique genes present in this panel. The intra-panel comparison was performed through a retrospective analysis of genomic data from The Pan-cancer Analysis of Whole Genomes (PCAWG) study [37].

The web-based Xena Browser was used for visualization and exploration of the data [38, 39]. More specifically available data sets from specimens with coding driver alterations information, including single nucleotide variations (SNVs) and small insertions-deletions (indel) and with consensus whole-genome copy number data as well as consensus fusion calls were downloaded and explored. The 990 specimens with information concerning all three types of alterations available were selected. Subsequently, we simulated the results that would have been obtained if this analysis had been performed using the gene sets included in the aforementioned panels and we explored the magnitude of the clinically actionable information obtained in each case. Variant classification and biomarker interpretation were performed as described above. For the copy number variation analysis, only the 43 genes of the Oncomine Comprehensive Panel v3 and the 333 of the Oncomine comprehensive plus panel with focal Copy Number Variations were included. In order to resemble the cutoff values used in everyday practice in our laboratory, a threshold of > 7 copies was used for considering a sample positive for copy number amplification and a threshold of < 1 copy for considering a gene loss [40].

### PD-L1 expression by immunohistochemistry

For the majority of tumors analyzed (such as lung, colorectal, pancreatic and ovarian cancer) as well as for tumors of unknown primary origin, the level of PD-L1 protein expression was defined as the percentage of viable tumor cells (TC) showing partial or complete membrane staining at any intensity. Furthermore, in some cases, the percentage of tumor Infiltrating Immune Cells (IC) showing staining at any intensity was also calculated [41–43]. In case of bladder, urothelial, and cervical carcinomas, PD-L1 was calculated through the Combined Positive Score (CPS) which is the percentage of positive cells (tumor, lymphocytes, and macrophages) showing partial or complete membrane staining at any intensity [44, 45]. In case of Head and Neck Squamous Cell Carcinoma, both CPS and TC values were calculated [46]. The analysis was conducted using the Immunohistochemistry (IHC) assay VENTANA PD-L1 (SP263) Assay (Roche Diagnostic) that utilizes the Monoclonal Mouse Anti-PD-L1, Clone SP263 accompanied by OptiView DAB IHC Detection Kit on a VENTANA BenchMark Series automated staining instrument.

For breast cancer patients, the VENTANA Monoclonal Mouse Anti-PD-L1, Clone SP142 antibody was used. The level of expression of the PD-L1 protein was defined as the percentage of tumor-infiltrating Immune Cells showing staining at any intensity [47].

### Physicians survey

In order to investigate the utility of a multi-biomarker analysis in clinical practice and if the results obtained from such approach have an impact in clinical decision making, a questionnaire was given to the referring oncologists, asking whether based on their experience, they consider such analysis useful for patients with the following tumor histological type:

Lung, Colorectal, Breast, Ovarian, Prostate, and rare or unknown origin tumors. It was a multichoice survey with the following options of response: (a) Useful, (b) in the metastatic setting only (c) not useful and (d) I do not know/not respond.

### Statistical analysis

Fisher’s Exact Test was used to compare the median TMB values and the percentages of TMB positivity of selected groups of patients (PD-L1 positive/negative, MSI-high/MSS) with SPSS (version 20. IBM SPSS STATISTICS). The p-values were based on Fisher’s Exact Test. A *p* value < 0.05 was considered to be statistically significant. Box plots were created using the Plotly.js charting library.

## Results

### Molecular analysis for targeted therapy

**Fig. 1:**
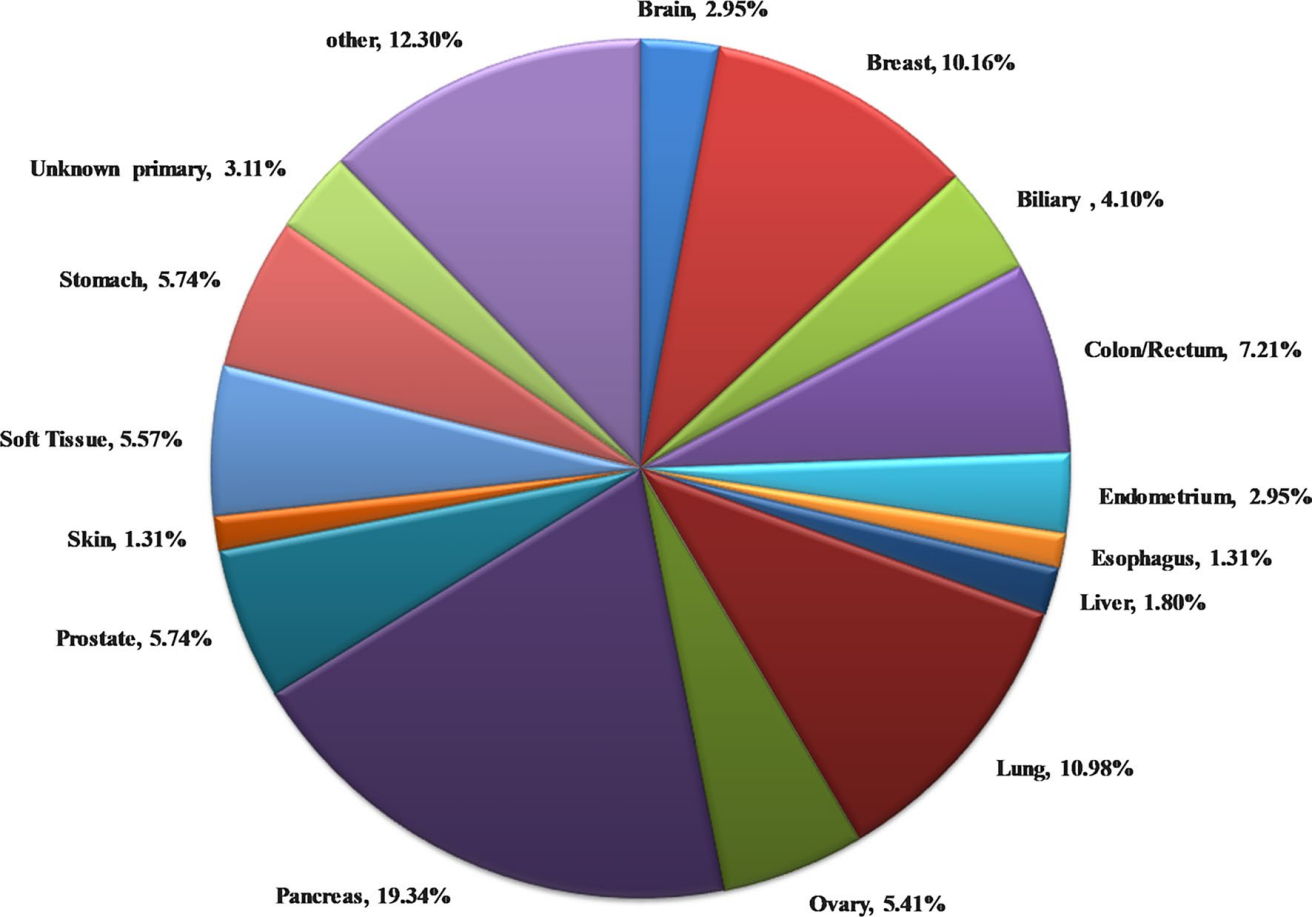
Tumor histological types analyzed by NGS

In the present study, 629 tumor tissues were subjected to targeted treatment biomarkers’ analysis, using a 161 gene NGS panel. Successful molecular analysis was achieved in 610 of the 629 patients analyzed, while in 19 (3.03%) cases, no results could be obtained due to low DNA/RNA quality or quantity. The tumor types analyzed included common tumor types with targeted treatment available, such as lung, breast and colorectal cancer, but also various hard to treat diseases such as pancreatic, ovarian, prostate, brain cancers, sarcomas, cholangiocarcinomas, and others (Fig. 1).

**Fig. 2:**
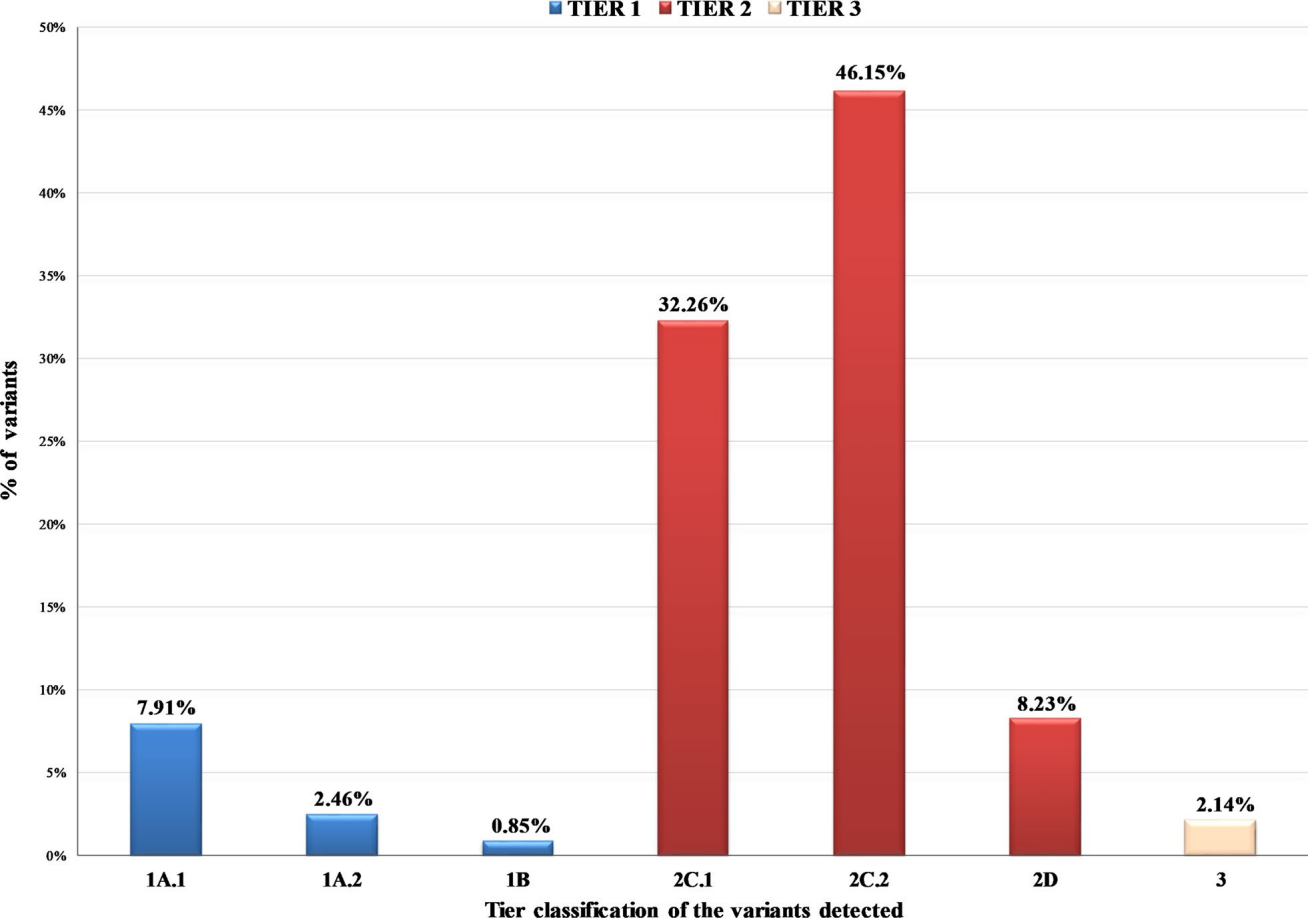
Tier Classification of the 936 pathogenic variants identified in the 610 tumors analyzed based on their predictive value. 1A.1: Biomarkers related to on-label treatment, 1A.2: biomarkers included in guidelines, 1B: biomarkers with strong evidence of correlation to treatment, 2C.1: biomarkers related to off-label treatment, 2C.1: biomarkers related to clinical trials, 2D: biomarkers with preclinical evidence of actionability, 3: biomarkers with unknown actionability

**Fig. 3:**
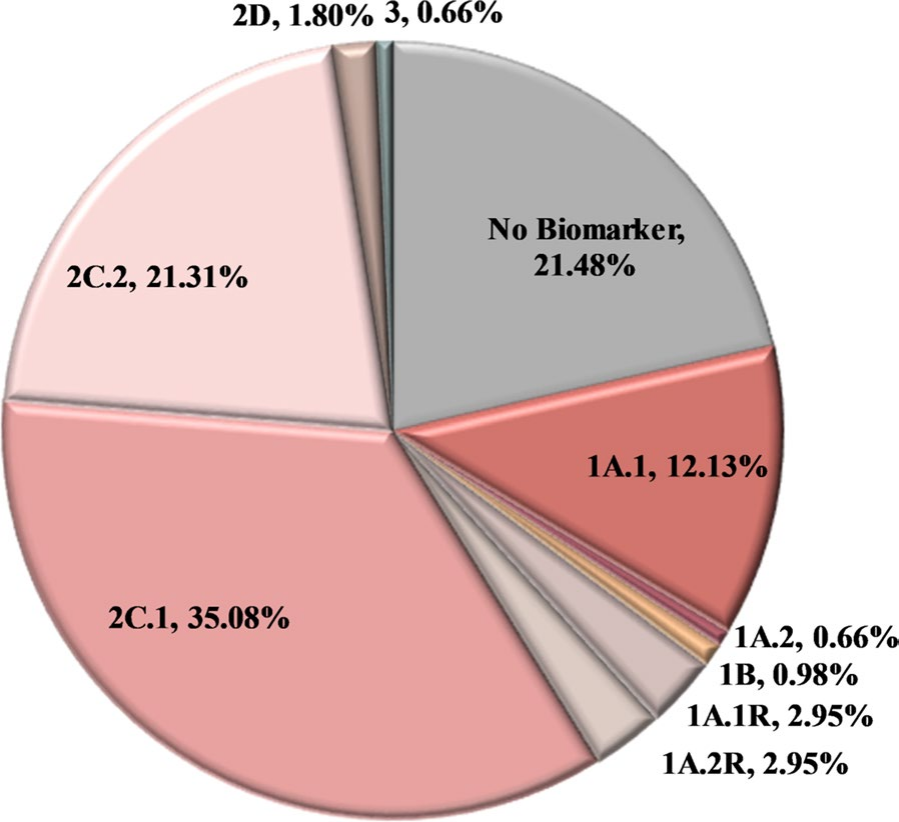
Patients categorization in the entire cohort based on the most clinically significant variant detected. In the case of multiple mutations present in the same patient, the variant with the higher level of evidence was used for establishing patient’s category. 1A.1: Biomarkers related to on-label treatment, 1A.1R: biomarkers related to resistance to an on-label treatment, 1A.2: biomarkers included in guidelines, 1A.2R: biomarkers related to resistance in a treatment included in guidelines, 1B: biomarkers with strong evidence of correlation to treatment, 2C.1: biomarkers related to off-label treatment, 2C.1: biomarkers related to clinical trials, 2D: biomarkers with preclinical evidence of actionability, 3: biomarkers with unknown actionability, no biomarker: patients with no biomarker available. In the case of colorectal cancer patients, the clinical significance of the RAS wild type phenotype was not considered in this figure

**Fig. 4:**
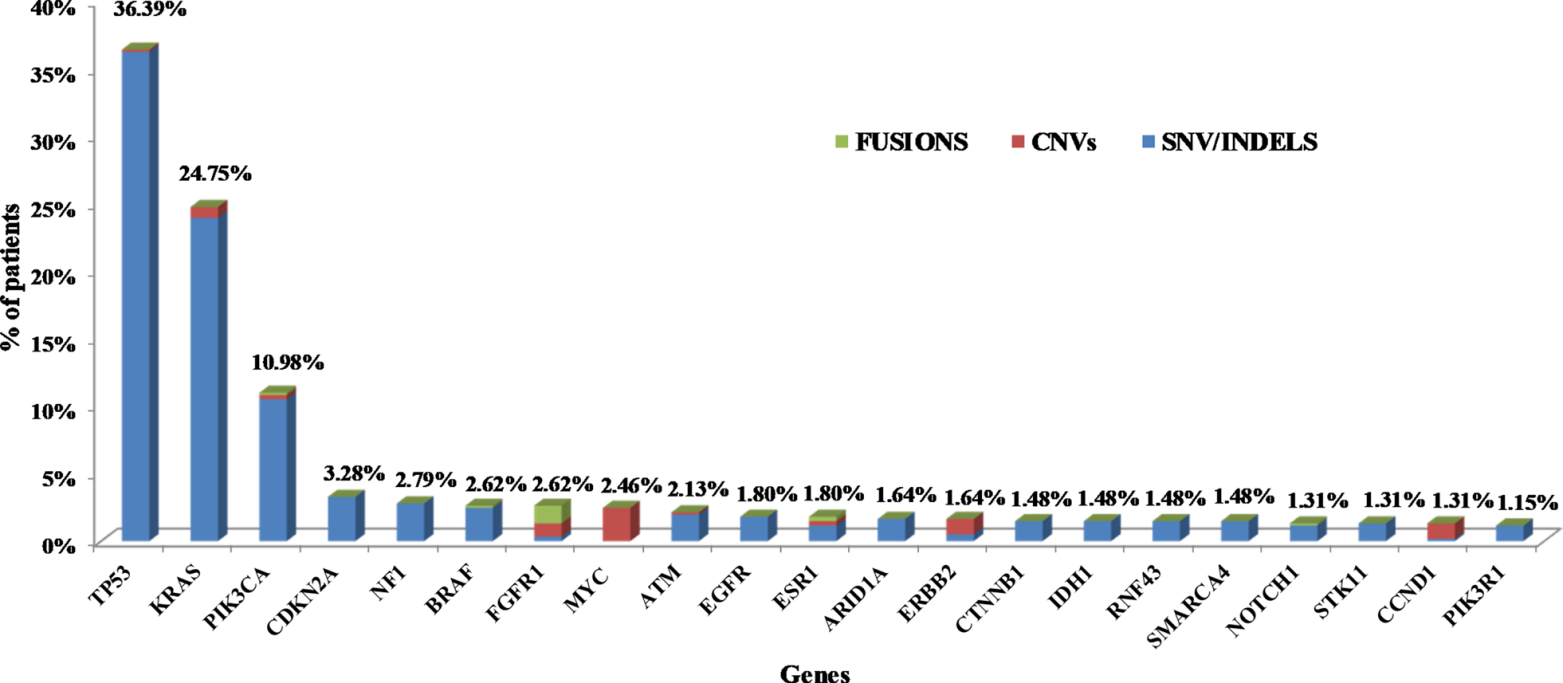
The Top 20 most frequently altered genes in the cohort analyzed

In total, 936 pathogenic variants in 112 genes were detected in 472 patients (Additional file 2: Table S2). Of those, 85.15% were single nucleotide Variants (SNVs) or a small insertions-deletions (indels) detected at the DNA level, while 3.31% of the variants concerned gene fusions and 11.54% Copy Number Variations (CNVs). 11.22% of the 936 variants identified were classified as Tier 1, 86.65% of them as Tier 2 and 2.14% as Tier 3 (Fig. 2, Additional file 3: Table S3). At least one variant was detected in 78.52% of the cases. 34.98% of the individuals analyzed carried one genomic alteration, while 23.81% and 19.87% carried two and three or more mutations respectively. The main reason for multigene test request was the assignment of the appropriate treatment based on patients’ molecular profile. Thus, patients were apportioned based on the clinical significance of the alterations detected. In the case of multiple mutations present in the same patient, the variant with the highest level of evidence (LoE) was used for establishing the patient’s category. Using this biomarker-defined categorization, 54.75% of the patients analyzed received information that is related to on-label or off-label treatment (Tiers 1A.1, 1A.2, 1B, and 2C.1). Additionally, the variant detected could be used as a criterion for inclusion in clinical trials (2C.2) or is under investigation in preclinical studies (2D) in 21.48% and 1.80% of the cases respectively. Furthermore, 5.90% of the patients harbored a variant associated with resistance to treatment (1A.1R, 1A.2R) (Fig. 3). As expected, the most frequently mutated gene in this cohort was the gatekeeper *TP53* gene, followed by the *KRAS* and *PIK3CA* genes. These genes were mutated in 36.39%, 24.75% and 10.98% of the patients, respectively (Fig. 4). Furthermore, 7.05% of the patients carried an alteration in a gene involved in the homologous recombination pathway. This type of alterations could be used as predictive biomarkers of response to PARP inhibitors (PARPi) treatment [48, 49].

### Tissue specific tumor molecular profile

In order to evaluate if molecular profile analysis is more useful in specific tumor types compared to others, the mutation frequency and clinical significance of the variants detected were calculated for the most common tumor types analyzed in our cohort (Additional file 4: Table S4).

#### Pancreatic cancer

**Fig. 5:**
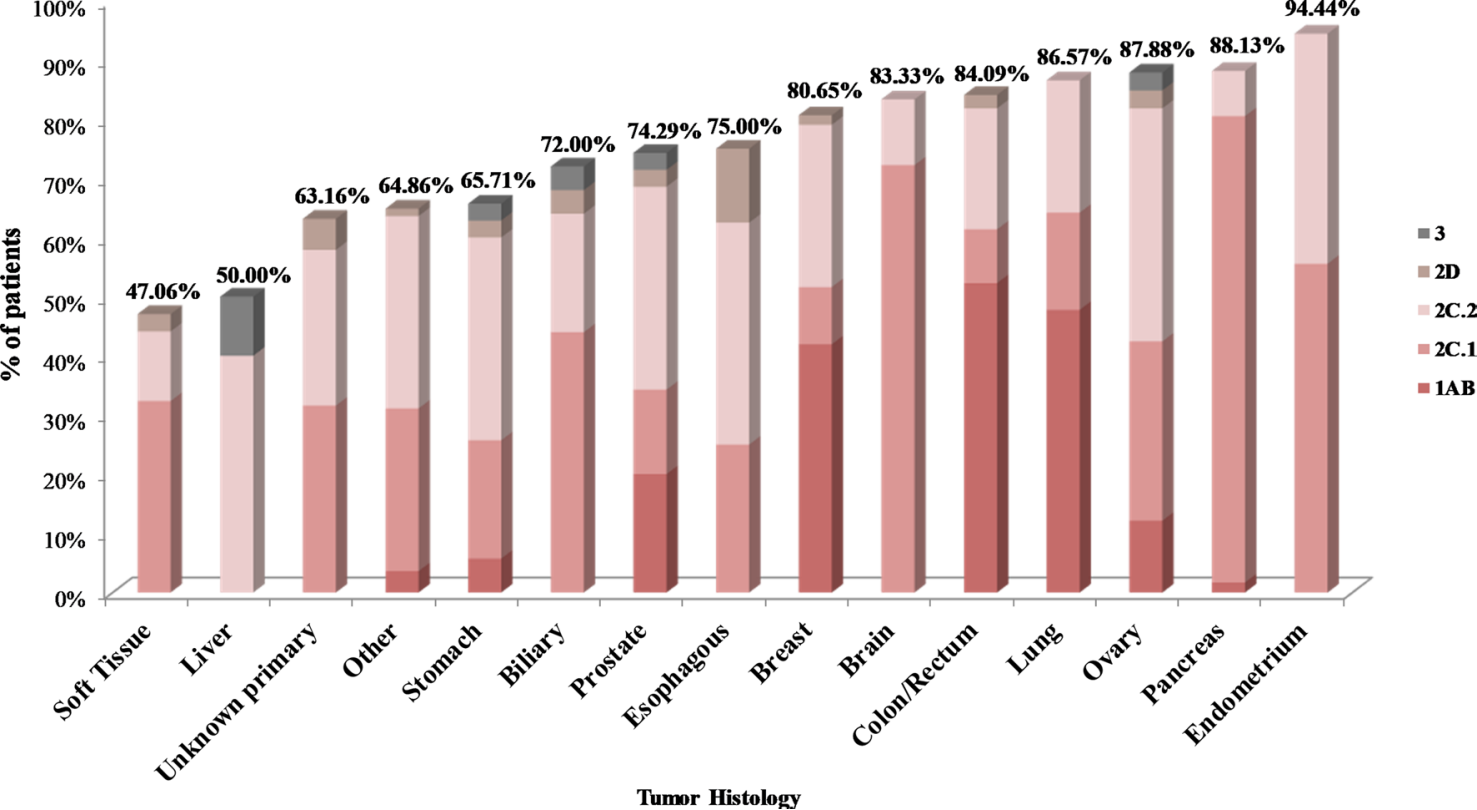
Mutation Rate and patients' categorization based on the Tier classification of the most clinically significant variant for each tumor histology. The following categories were used: 1A/1B: patients harboring alterations that are biomarkers for on-label treatments or with strong evidence of predictive value for an on-label treatment (Tier 1), 2C.1: patients with biomarkers related to off-label treatment, 2C.2: patients with biomarkers related to clinical trials, 2D: patients with biomarkers with preclinical evidence of predictive value, 3: patients harboring alterations with conflicting evidence of cancer association. B. Percentage of patients with On-label and off-label mutations identified and the type of alterations detected. Genes of the homologous recombination complex are labeled in blue.

In the present study, 118 patients undertaking tumor molecular analysis had a diagnosis of pancreatic cancer. *KRAS* mutation was the prevalent mutated gene in this tumor type, with a mutation frequency of 74.57%. In 64.41% of the patients, an alteration in this gene was the finding with the higher LoE. However, other gene alterations with predictive value (2C.1) coexisted in 10.16% of the *KRAS* mutant patients. Moreover, in 6 cases (5.08%), the mutation detected was in an HR gene (1 *ATM*, 2 *PALB2*, 1 *CDK12*, 1 *FANCA*, 1 *NBN*) with evidence of response to PARPi. Additional variants with associated off-label treatments were detected in *FGFR1* & 4, *HER2*, *MET*, *PIK3CA* and *POLE* genes (Fig. 5, Additional file 5: Figure S1).

Furthermore, 2 patients (1.69%) carried a somatic mutation related to an on-label drug or with strong evidence of actionability. These mutations were detected in genes of the mismatch repair complex (*MLH1* and *MSH2*) and were indicative of microsatellite instability and thus response to immunotherapy.

#### Lung cancer

In the 67 Lung cancer, patients tested an alteration was detected in 86.57% of the cases (Fig. 5). The variant identified was related to an FDA approved treatment in 20.89% of the patients. These variants concerned *EGFR*, *BRAF* (p.V600) and *HER2* mutations in percentages of 8.96%, 4.48% and 1.49%, respectively. Moreover, *ALK* and *RET* translocations were detected in 1.49% and 4.48% of the cases, respectively. EGFR TKI resistance conferring *KRAS* mutations (Tier 1A.2) were detected in 26.87% of the cases. Apart from these established biomarkers, the expanded gene panel analysis was able to detect additional mutations in multiple other genes with 2C.1 evidence of predictive value in 16.42% of the cases (Additional file 6: Figure S2). Unexpectedly, 6 of the patients (8.95%) carried a mutation in a gene related to PARP inhibitor therapy.

#### Breast cancer

In the 62 Breast Cancer Patients included in our cohort, a pathogenic variant was found in 80.65% of the cases. A Tier 1 variant was detected in 41.94% of the patients, while in 9.68% a Tier 2C.1 variant, related to off-label treatment, was identified. The most prevalent altered gene in these patients was the *PIK3CA* gene, with 33.87% mutation rate. Additionally, an HR gene alteration was present in 9.68% of the tumors analyzed (Additional file 7: Figure S3).

#### Other cancers

In the 44 patients with Colorectal cancer, the mutation rate was 84.09% (Fig. 5, Additional file 8: Figure S4). Eighteen patients (40.91%) carried a mutation in one of the *RAS* genes which are biomarkers of resistance to EGFR antibodies treatment [50, 51]. Additionally, three patients carried a targetable *BRAF* somatic mutation. One *PMS2* positive tumor mutation was proven to be of germline origin, and thus it was considered eligible for immunotherapy treatment. Tumor analysis is essential for patients with colorectal carcinoma, because it provides Tier 1 information on treatment strategy in all cases. Thus, treatment can be directed toward EGFR antibody therapy in the presence of a wild-type *KRAS/NRAS* gene finding or toward alternative treatment options if a mutation is detected.

Among the 35 patients with prostate cancer, at least one somatic alteration was identified in 74.29% of them (Fig. 5). In 6 cases, the mutation detected was in an HR gene (17.14%). Furthermore, 87.88% of the 33 patients with ovarian cancer, carried at least one somatic alteration. Four patients carried a mutation in *BRCA1*/2 genes, which are biomarkers of response to PARPi therapy, while in four patients, somatic mutations in off-label biomarkers were identified. Concerning brain tumors, the mutation rate was 83.33%. An alteration with associated potentially significant predictive biomarker was detected in 13 patients (72.22%) (Fig. 5). However, in this tumor histology, the multigene analysis seems to confer not only predictive but also prognostic/diagnostic information [52, 53]. Genes with diagnostic significance are used by the World Health Organization Classification of Tumors of the Central Nervous System. For example, *IDH1* and *IDH2* mutations are used for distinguishing primary from secondary gliomas, while the simultaneous presence of *IDH1/2* and *TP53* alterations are distinctive of the diffuse astrocytoma histology [52].

Concerning the other histological types, even if the number of patients tested is small, it seems that in tumors of the endometrium (18 cases), esophagus (8 cases) and cholangiocarcinoma (25 cases) the mutation rate is relatively high (94.44%, 75.00% and 72.00% respectively). On the contrary low mutation rates are observed in gastric tumors (35 cases), hepatocellular carcinomas (10 cases) as well as in the 34 sarcomas analyzed (65.71%, 50.00% and 47.06% respectively).

### Panel comparison

The genetic information obtained by the 161 gene panel used in this study compared to that obtained from panels containing fewer genes was evaluated. At this regard, we conducted a comparison of the alterations that would have been detected if two smaller hotspot panels, of 24 and 50 genes respectively, had been used in the 610 patients analyzed (Additional file 9: Table S5).

**Fig. 6:**
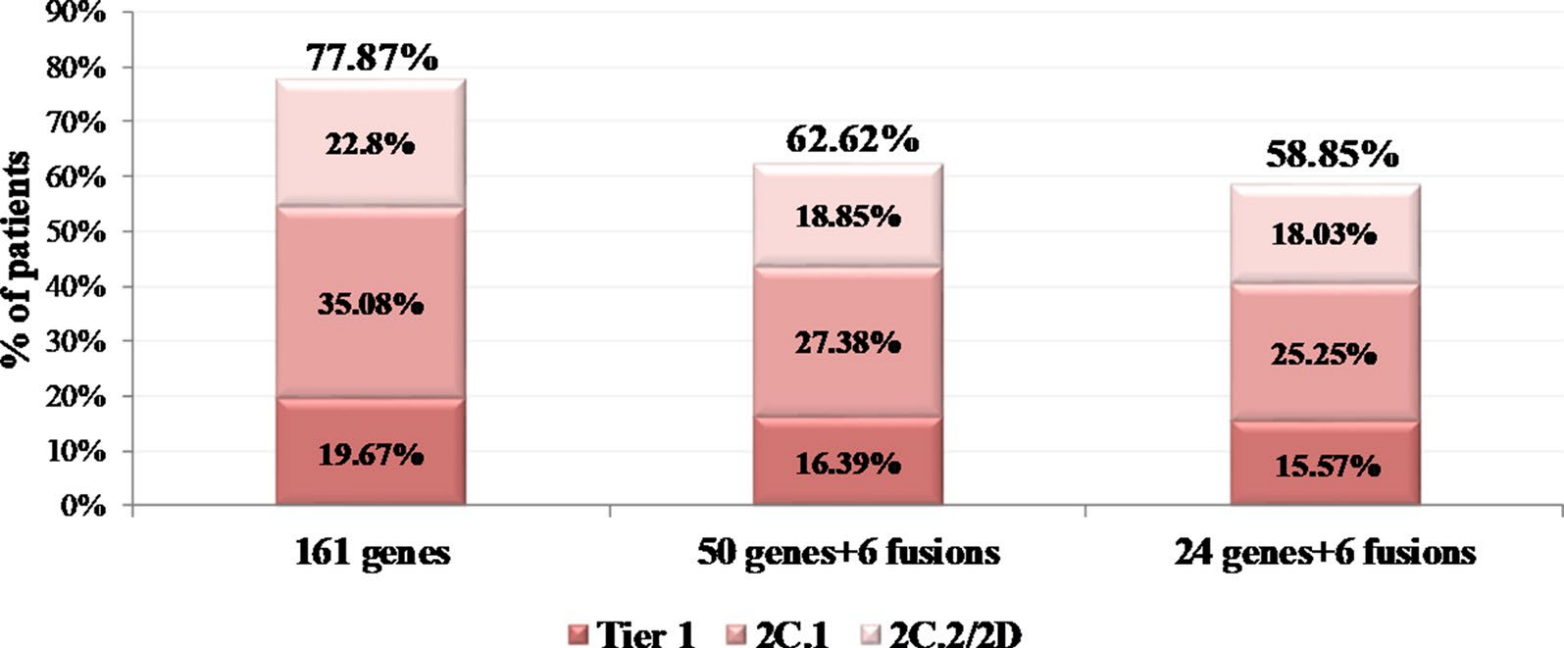
Simulation of patients' biomarker-defined categorization based on their most clinically significant variant when the analysis is performed using either the 161 or the 50 or the 24 gene panels. The following categories were used: 1A/1B: patients carrying Tier 1 alterations, 2C.1: patients with 2C.1 alterations, 2C.2/2D Patients with 2C.2 or 2D alterations

If the 24 gene panel had been used in our cohort, a clinically significant variant (Tier 1 and 2) would have been detected in 58.85% of the cases. In comparison, this percentage would have been 62.62% by using the 50 gene panel. However, these rates are much lower than the 77.87% obtained by the 161 gene panel. Furthermore, considering the on-label and off-label biomarkers, the larger panel managed to detect 13.94% and 10.98% more on/off-label treatment-related biomarkers compared to the 24 and 50 gene panel respectively (Fig. 6).

In order to evaluate if the number of genes analyzed is adequate for implementation in clinical practice, or if by increasing the number of genes tested a more informative result would have been obtained, we compared the actionability of our panel with a more comprehensive panel containing 501 DNA genes and 51 fusion drivers genes (38 of them also analyzed at the DNA level), for a total of 514 unique genes present in this panel (Additional file 10: Table S6, Additional file 11: Table S7).

Among the 990 patients with DNA sequencing results available, an SNV or indel alteration to a driver gene was obtained in 90.4% (895/990) of the cases using the whole genome sequencing approach of the study. In comparison the 161 gene panel would have detected such alterations in 72.12% of the patients and the larger panel 83.03%. At least one Copy Number Variations would have been detected in 29.09% and 47.37% of the cases by the smaller (161 genes) and bigger panel (500+ genes) respectively. Both panels would have detected a fusion driver gene in 7.68% of the cases.

**Fig. 7:**
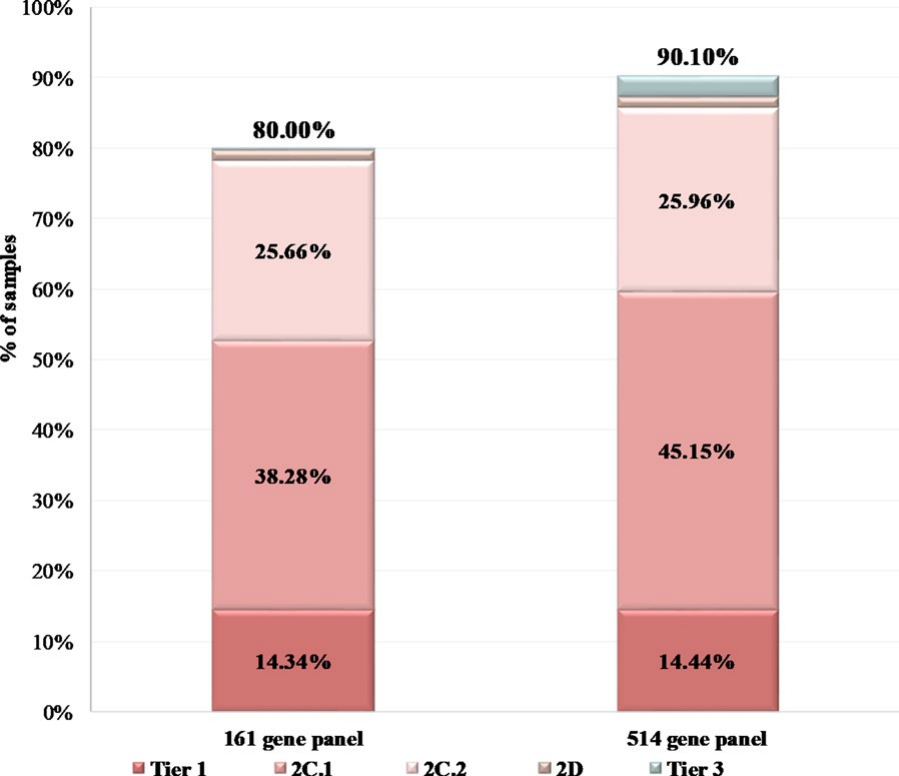
Simulation of the 990 PCAWG samples’ categorization based on their most clinically significant variant when the analysis is performed using either the 161 the 514 gene panels. The following categories were used: Tier 1 variants: patients carrying at least one Tier 1 alteration, 2C.1: patients whose most clinically significant variant is classified as 2C.1, 2C.2: patients with 2C.2 alterations, 2D: patients with 2D alterations, Tier 3: patients carrying Tier 3 alterations

Considering all type of alterations (SNV, indel, CNV, gene fusion), at least one actionable alteration would have been identified in 80.00% of the samples if the 161 gene panel was used and in 90.10% of them if the 514 gene panel was implemented for the analysis (Fig. 7). Furthermore, at least one clinically relevant biomarker, related to on/off-label treatment or to clinical trials would have been detected in 78.28% and 85.56% of the cases by the 161 and the 514 gene panels respectively.

Thus, the increase in the number of genes analyzed seems to increase the yield of patients who could benefit from targeted treatments.

### Physicians survey

Additionally, in order to investigate the implementation of tumor molecular profile analysis among physicians, a questionnaire was sent to referral oncologists asking whether they consider useful, such analysis for treatment decision making in various tumor types. 61 physicians responded to the survey. By far, the tumor type with the majority of positive responses was lung cancer, with 100% of the physicians responding that multigene panel should be performed for such tumor type (Table 1).

**Table 1:** Oncologists responses concerning the clinical utility of NGS multigene analysis in various tumor types

Oncologists responses	Tumor histology						
	Lung (%)	Colorectal (%)	Breast (%)	Ovarian (%)	Pancreas (%)	Prostate (%)	Rare/unknown (%)
Useful	60.66	65.57	45.90	65.57	50.82	50.82	85.25
Useful in the metastatic setting	39.34	29.51	34.43	14.75	44.26	39.34	14.75
Not useful	0.00	4.92	9.84	9.84	0.00	0.00	0.00
No opinion/response	0.00	0.00	9.84	9.84	4.92	9.84	0.00

For colorectal cancer patients, a multigene analysis was considered useful in the primary or metastatic setting by 95.08% of the participants. For breast, ovarian, prostate and pancreatic cancers, the NGS utility was recognized by 80.33%, 80.32%, 90.16% and 95.08% of the participants respectively.

### Immunotherapy biomarkers analysis

Tumor testing can give information for the selection of both appropriate targeted treatment and immunotherapy. The most known immunotherapy biomarkers are TMB, PD-L1 and MSI analysis. In the cohort of 610 patients with successful NGS testing for targeted therapy, 395 also requested TMB analysis. PD-L1 testing was performed in 198 cases, and MSI analysis in 206 patients. In 204 cases, all three immunologic biomarkers were analyzed (Additional file 12: Table S8) with successful analysis for all of them achieved in 191 cases.

#### Tumor Mutation Burden

Among the 395 patients with TMB analyzed, 14 cases (3.54%) could not receive a result due to the low quality of the genetic material analyzed. In these cases a high proportion (> 60) of variants consistent with de-amination artifacts was detected, and thus these sequencing result could not be evaluated for TMB analysis, as indicated by the manufacturer [54]. A successful TMB calculation was obtained for the remaining 381 patients.

**Fig. 8:**
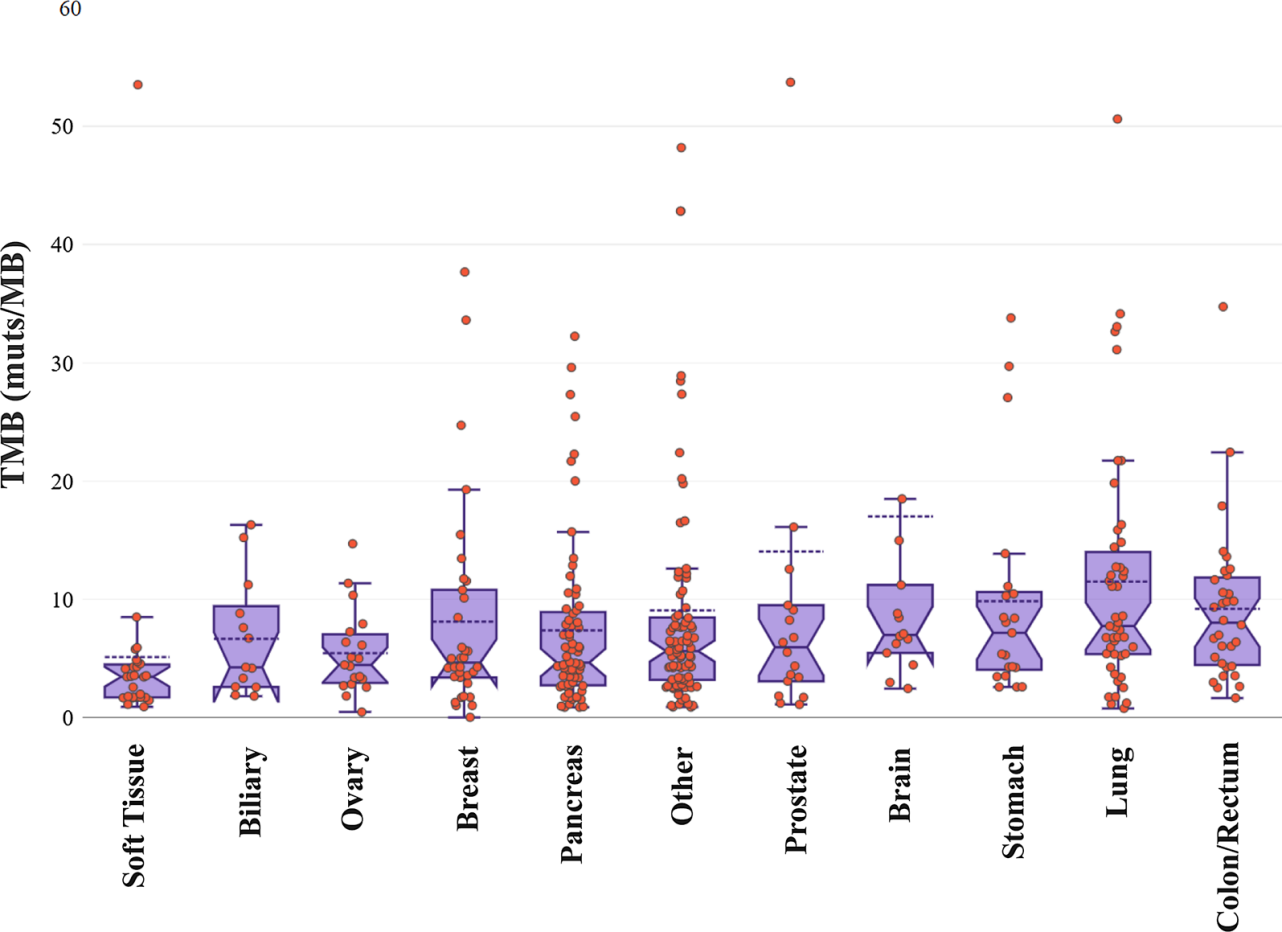
Box plots showing the median TMB values in various tumor types. Three samples with TMB values > 60 were omitted from the plot for visualization purposes

**Fig. 9:**
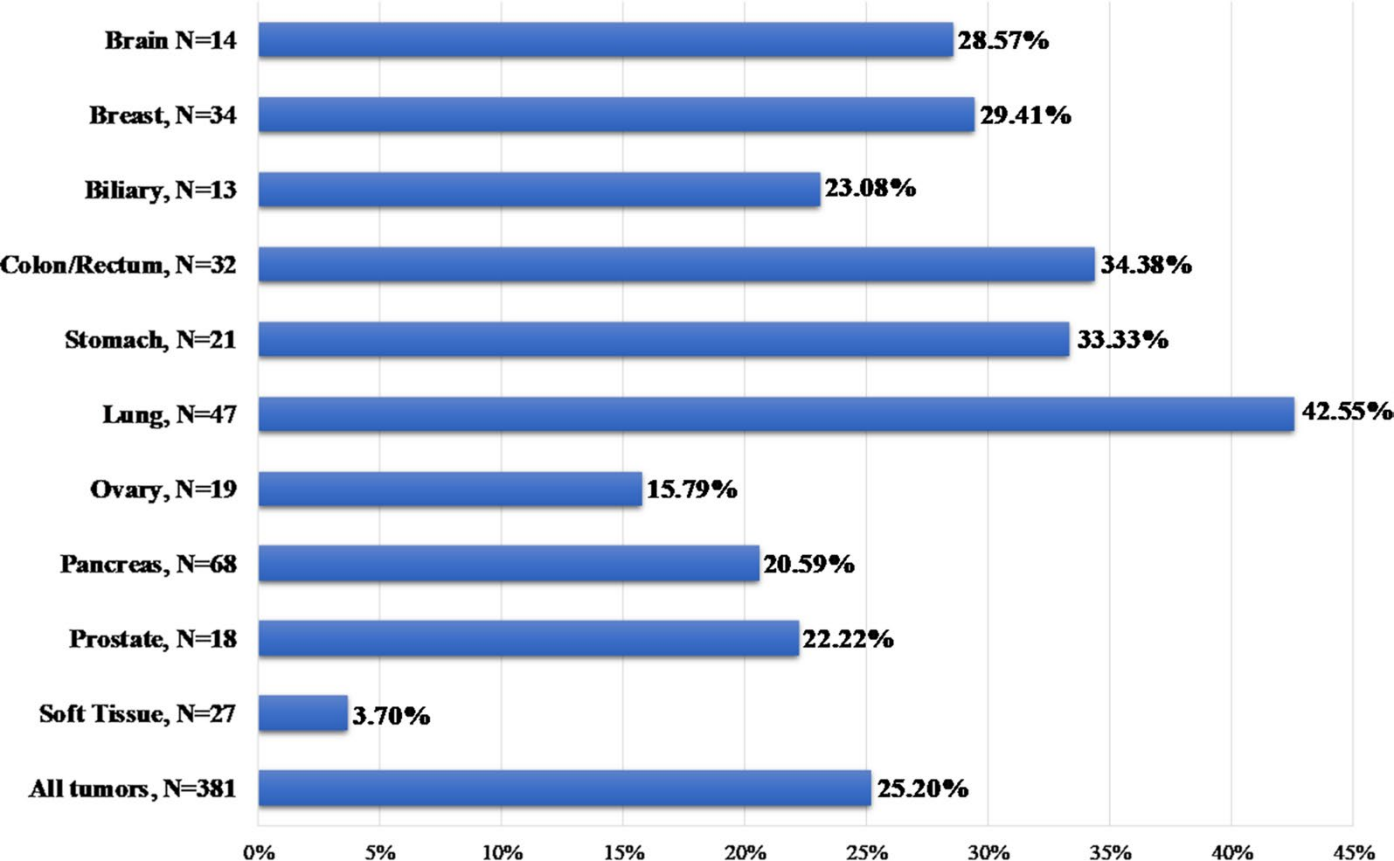
TMB positivity rate in various histological tumor types

The TMB value ≥ 10 muts/MB has been employed to separate high and low TMB values as indicated by the results of the open-label, phase 2 KEYNOTE-158 study that led to the recent FDA approval of Pembrolizumab for metastatic solid tumors [29]. The median TMB value obtained was 5.60 (min 0; max 134), with 96 samples showing a TMB value higher than 10 muts/Mb and 285 samples with a lower than 10 muts/Mb value. The tumor type with the highest TMB median value in our cohort was colorectal cancer (median TMB = 8.02), with 11 samples showing TMB > 10 and 21 samples TMB < 10 muts/Mb, followed by lung cancer (median TMB = 7.72, 25 samples with TMB > 10 and 22 with TMB < 10 muts/Mb (Fig. 8). The tumor types with the lowest TMB values were sarcomas, ovarian and pancreatic cancers (median TMB 3.43, 4.44 and 4.63 muts/Mb respectively). Accordingly, the positivity rates varied by tumor type with lung cancer showing the highest (42.55%) and soft tissue tumors displaying the lowest positivity rate (3.70%) (Fig. 9).

#### PD-L1 expression

Among the 206 patients referred for PD-L1 analysis by immunohistochemistry, a successful analysis was achieved in 198 cases. PD-L1 positivity (> 1%) was observed in 38.89% of them (77/988). Moreover, an intense PD-L1 expression was observed in 9.09% of the patients, exhibiting TPS values greater than 50% or CPS greater than 50.

**Fig. 10:**
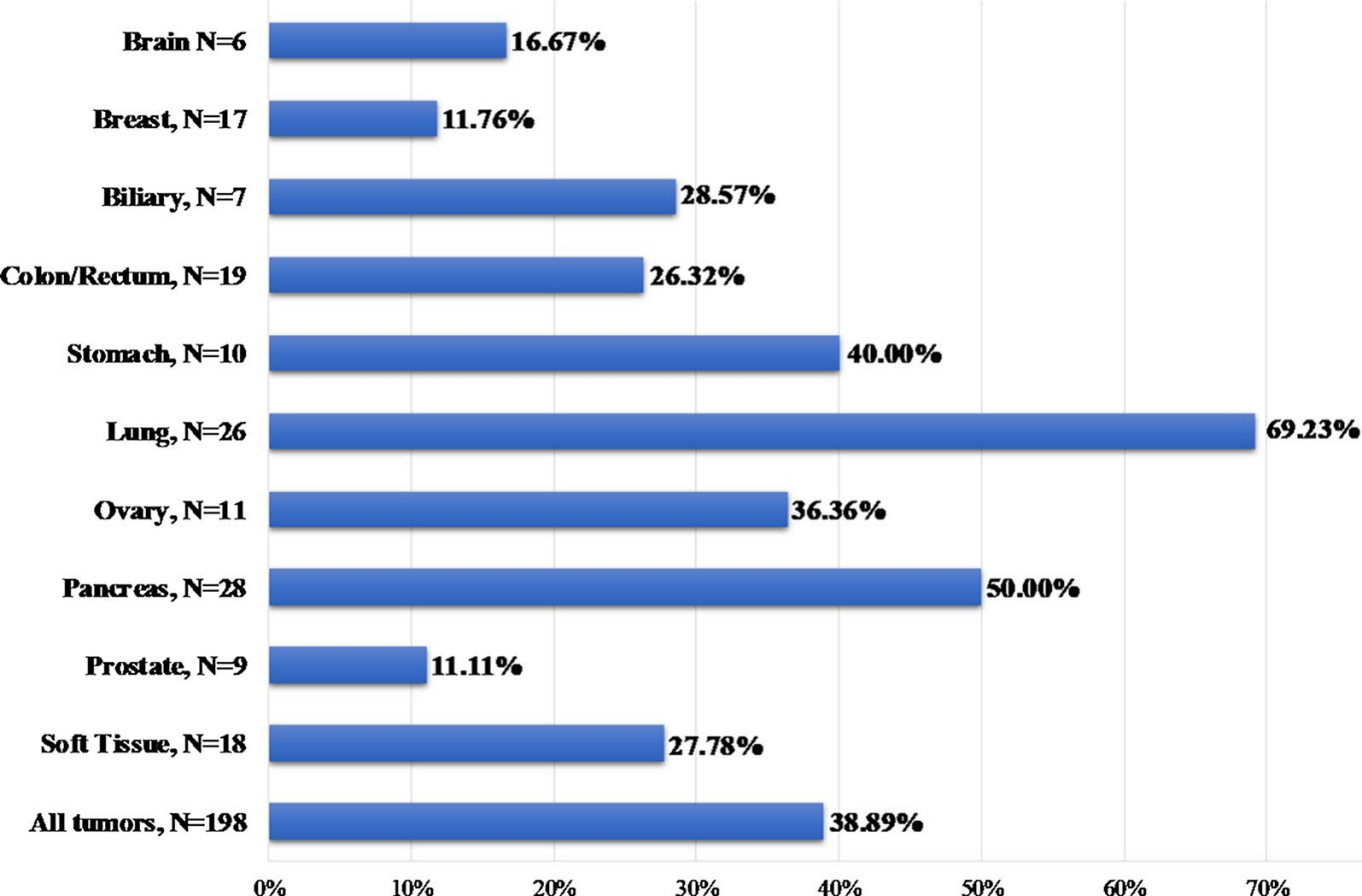
PD-L1 positivity rate in various tumor histological types

In the 26 lung cancer patients tested 69.23% had a TPS value > 1, with 19.23% showing an intense (> 50%) PD-L1 expression. The positivity rate in various tumor types is illustrated in Fig. 10. Among the 77 PD-L1 positive cases identified in our cohort 26 patients (33.77%) showed concomitant TMB positivity (> 10muts/MB).

**Fig. 11:**
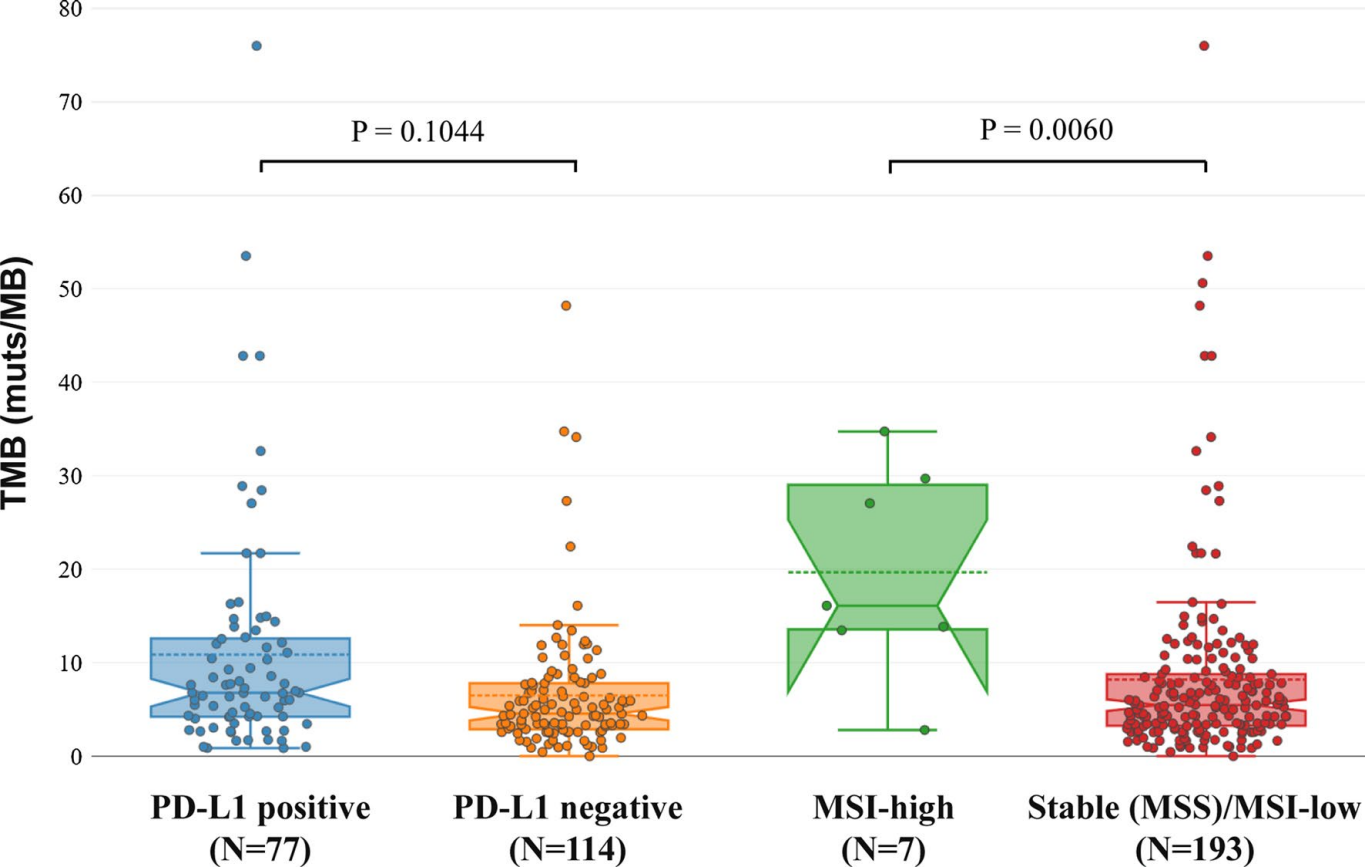
TMB-MSI and TMB-PD-L1 correlation

In accordance to previous studies, no association of TMB and PD-L1 values was observed (Fig. 11) [55, 56].

#### Microsatellite instability

Microsatellite instability was detected in 8 out of the 206 tumors tested (3.88%), while for one tumor the analysis failed due to the low quality of the genetic material obtained. Patients with tumors showing MSI high status had a diagnosis of Ovarian cancer, Pancreatic cancer, Colorectal cancer, Prostate cancer, Gastric cancer and Sarcoma. In 2 cases, the tumor instability was linked to hereditary mutations in MMR genes (*MSH2* and *PMS2*). TMB analysis data were also available in 7 of these patients with 6 of them showing high TMB value (> 13.46muts/MB). Thus a strong correlation between TMB and MSI was observed with MSI high tumors showing higher median TMB values, in accordance with previous studies [57, 58]. However, it should be noted that among the 193 MSI stable patients with TMB data available, high TMB values were also observed in 42 cases (Fig. 11).

MSI is known to be caused by impairment of the MMR gene system, leading to increased neo-antigen burden and thus elevated TMB. However, this represents only one of the oncogenic processes related to elevated TMB values. Several other mechanisms, such as environmental carcinogens and specific gene alterations are known to induce mutagenesis and thus TMB increment, resulting in higher positivity rates for this biomarker in several tumor types [59, 60]. Given its compelling evidence of predictive value; TMB is superior to MSI analysis, identifying more patients eligible for PD-1/PD-L1 blockade compared to MSI.

#### Immunotherapy biomarkers’ comparison

Among the 191 patients with all three immunotherapy biomarkers tested, ICIs option based on TMB result could be considered in 44 patients (23.04%), 26 of them with simultaneous PD-L1 positivity. Furthermore, 51 additional patients showed PD-L1 positivity and 1 MSI-high result. Collectively, positivity to one of these biomarkers and thus a possibility of benefit from ICIs treatment was observed in 50.26% (96/191) of these patients.

**Fig. 12:**
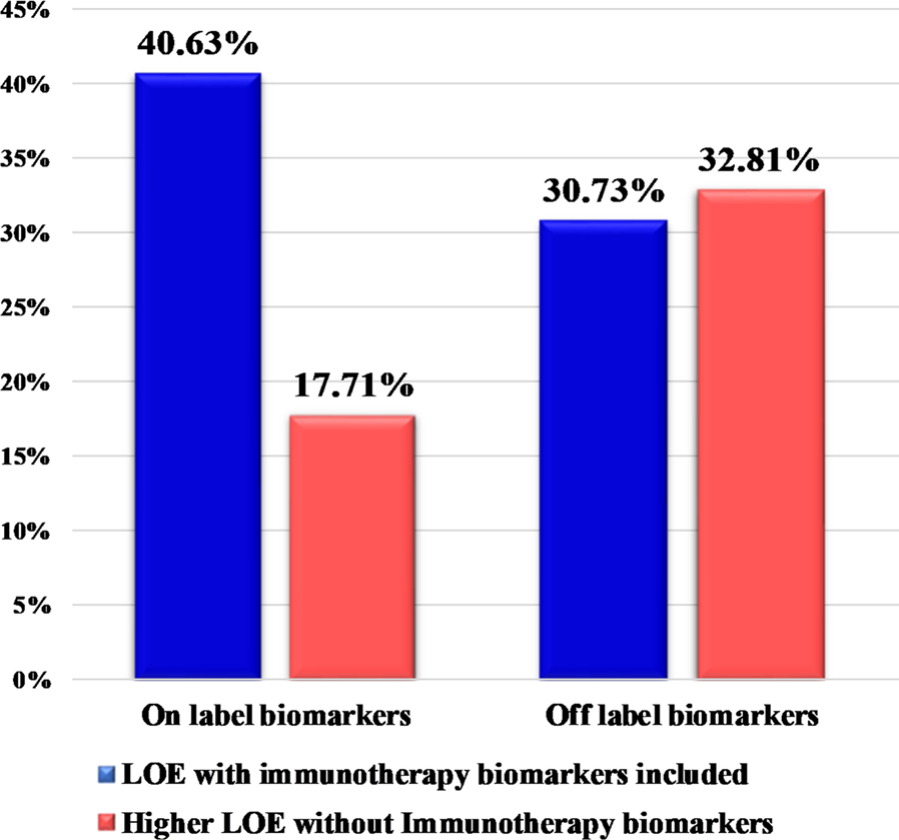
Patients' categorization based on the level of evidence of the most clinically significant variant associated with response, with and without the use of immunotherapy biomarkers. Biomarkers associated with resistance were excluded from this analysis

Furthermore, the analysis of both targeted treatment and immunotherapy biomarkers, revealed an actionable finding (Tier 1 or 2) in 83.25% of the cases. Moreover, the addition of the immunotherapy biomarkers to the molecular profile analysis increased the number of patients with an on-label treatment recommendation by 22.92% (Fig. 12). TMB analysis increased the LoE of treatment recommendations to 1A.1 in 36 cases, with 23 of them showing concomitant PD-L1 positivity.

The value of ICI biomarker analysis was also observed in tumor types that are highly represented in this cohort, such as lung and pancreatic cancer. In the 26 lung cancer patients analyzed for both types of biomarkers, an increase in on-label treatment recommendation of 61.54% was observed after targeted therapy biomarkers were supplemented with immunotherapy biomarkers, with 96.15% of these patients having an on-label or off-label biomarker detected. In addition, among the 29 pancreatic cancer patients with comprehensive tumor analysis of ICI and targeted therapy biomarkers, a biomarker associated with on-label treatment was detected in 10.34% of cases, whereas this percentage would be reduced to 3.45% if ICI biomarkers were excluded from the analysis.

## Discussion

### Molecular profile analysis

In the present study, 629 cancer patients have been referred by their treating physician for biomarkers’ analysis using a 161 gene NGS panel. In 610 of them, a successful tumor molecular profile was obtained with at least one actionable variant (Tier 1–2) being detected in 77.87% of the cases. All pathogenic variants were categorized based on their clinical significance, and only Tier 1 and 2 variants were reported since variants of unknown significance, and the benign/likely benign ones were considered confusing rather than useful for the treatment course information. In 54.75% of the patients, the information obtained could be used for on-label or off-label treatment reception (Tiers 1A.1, 1A.2, 1B, and 2C.1) while 21.31% of the cases received a variant that could be used for clinical trials inclusion.

Tests offering comprehensive tumor molecular profiling are currently being requested by a steadily increasing number of oncologists, especially for patients with limited treatment options available. A good implementation of tissue analysis in treatment decision making was observed in the survey conducted in this study among oncologists. More than 80% of the participating physicians consider clinically useful the tissue NGS analysis for a variety of common tumor types. This percentage was increased to 100% for tumors with many targeted treatment options available such as lung cancer and for tumors with few treatments available such as tumors of unknown origin or rare tumors.

Advances in sequencing technologies and NGS platforms throughput have permitted simultaneous analysis of multiple tumor biomarkers at an adequate time frame to be tailored fit in the design of the treatment plan and at an affordable cost for the patients. The information obtained can be used to address targeted treatment, immunotherapies or in case of negative results traditional treatment approaches. Various studies have shown the efficacy of gene-directed treatment compared to the unselected treatment assignment [5, 61, 62]. In the IMPACT (Initiative for Molecular Profiling and Advanced Cancer Therapy) study the overall response rate (ORR), and the time-to-treatment failure (TTF) were higher in patients with a molecular aberration that received a matched treatment compared to those who received unmatched treatment [6]. Similarly, in the IMPACT/COMPACT trial, the response rate of patients treated according to their genotype had an overall response rate superior compared to those treated on genotype unmatched clinical trials (19% VS 9% respectively) [7]. Accordingly, the National Comprehensive Cancer Network (NCCN) guidelines outline the contribution of broad molecular profile analysis in the improvement of patients' care in various tumor types. Likewise, the European Society for Medical Oncology (ESMO) recommends routine utilization of tumor NGS analysis for NSCLC, prostate cancer, ovarian cancer and cholangiocarcinoma [63].

Furthermore, more than 200 ongoing clinical trials are currently investigating the impact of molecular directed treatment and the eventual benefit of this approach in patients with several advanced solid tumors and hematological malignancies (www.clinicaltrials.org). Among these, several tumor-agnostic randomized (NCT02152254, NCT03084757) and non-randomized (NCT02465060, NCT03155620, NCT02693535, NCT02290522, NCT03297606, NCT02029001) trials are expected to provide evidence of the clinical benefit of such approach in multiple solid tumor types. Hence, several pharmaceutical companies are focusing on the development of treatments with pan-cancer efficacy [9]. The first tumor agnostic therapy with a biomarker included receiving FDA approval was the PD-1 inhibitor Pembrolizumab, which was approved for patients with MSI unstable tumors [9]. Subsequently, TRK inhibitor therapy gained approval in NTRK fusion-positive cancers independently from the tumor’s histology [64–66]. Even though the clinical value of these biomarkers cannot be disputed, the percentage of patients positive for these biomarkers is relatively small. For example, in our study, only 8 out of the 198 patients analyzed presented microsatellite instability. This biomarker seems to be more significant for colorectal cancer patients, where it is present in 10–15% of the cases, while it is of no use for other tumor types, where it is rarely detected [20]. Similarly, the frequency of *NTRK* fusions in solid tumors of adults is extremely rare in certain tumor types [64, 67]. Consequently, no positive *NTRK* tumor was detected in our cohort.

On the other hand, there are agents, with associated biomarkers, that have shown activity in a variety of tumor types. PARP inhibitors are a typical example of such agents having already received approval for Ovarian, Breast, Pancreatic and Prostate cancer patients harboring *BRCA1*/*2* mutations (https://www.fda.gov). Apart from *BRCA1*/*2* mutations, other genes involved in the same pathway of homologous recombination seem to be adequate biomarkers of response to such agents, with several clinical trials investigating the expansion of PARPi targeting biomarkers [48, 68–71] (www.clinicaltrials.org). These efforts led to the recent approval of the PARP inhibitor Olaparib for metastatic castrate-resistant prostate cancer patients with mutations in other HR genes besides *BRCA1*/*2*, increasing the percentage of patients with a potential predictive biomarker result who could benefit from that treatment [69, 72]. Thus, multigene analysis providing comprehensive information about the mutational status of HR genes should be used for better identification of responders to such therapy. In our cohort, 7.05% of the patients carried an alteration in an HR gene, with certain tumors showing increased levels of these alterations, such as breast cancer (9.68%), ovarian cancer (20%) and prostate cancer (17.14%). Moreover, the majority (74.42%) of the HR-positive patients, carried an HR gene mutation in a non *BRCA1/2* gene, indicating the necessity of gene panel analysis for the identification of patients eligible for PARPi treatment.

An important issue when a multigene analysis is requested is the number of genes that should be included in such analysis and whether analyzing so many genes is offering more solutions in the physicians' search for an appropriate targeted treatment option for their patients. Therefore, we compared our 161 gene panel with two smaller ones of 24 and 50 genes. The genes included in these panels have been widely used in our laboratory and others to identify clinically relevant mutations in various tumor types [32–36]. However, recent advances in the discovery of predictive biomarkers seem to be forcing the analysis of more genes that could provide more treatment options for these patients. Nevertheless, there is still skepticism among some oncologists about the clinical utility of broader gene analysis. However, our results showed that if the analysis had been performed with the 24 and 50 gene panels, the percentage of positive cases would have been reduced to 58.85% and 62.62%, respectively, compared to the 77.87% obtained with the 161-gene panel. On the contrary, for lung cancer patients, the use of 24 gene panel seems acceptable for analysis since it could identify all biomarkers related to on-label treatments' sensitivity or resistance (46.97%). Thus, our results indicate that the 24 and 50 gene panels are not adequate for pan-cancer analysis since drug approvals of the recent years recommend the analysis of more biomarkers, with the exception of lung cancer.

A panel analyzing 514 single genes has been recently implemented for tumor analysis in our laboratory. Since we have observed an increase in the rate of patients with a positive tumor finding of at least 10% in the first 50 samples analyzed, we decided to compare it with the panel used in this study in order to evaluate if it could increase the actionable information obtained by tumor testing analysis. Thus, 990 samples with known genetic profile from PCAWG database were used in order to simulate the percentage of tumor alterations that could be obtained using different size of cancer panels. Among these samples, if the 161 gene panel was used, an SNV or indel alteration in a driver gene would have been detected in about 72% of the cases. The utilization of a larger panel slightly increases the number of actionable alterations obtained to 83%.

A result related to on/off-label treatment or to a clinical trial would be obtained in 85.56% of the cases if the 514 gene panel was used compared to 78.28% obtained by the 161 gene panel. Thus, both panels seem to give comparable results in terms of the actionable information obtained with the 514 gene panel, including the most actionable biomarkers. The main limitation of this comparison is that the variant calling, and copy number methodologies vary between the targeted assays and the whole genome methodology used in the PCAWG project. Nevertheless, the increase in the number of clinically significant variants identified when a larger panel is used, reflects what is usually observed in clinical practice.

Despite all the advantages, there is much skepticism concerning the use of a personalized selection of appropriate treatment. A first difficulty in using broad tumor molecular profile analysis for treatment selection is the unavailability in some cases of appropriate tumor tissue to perform the analysis. This could be due to the low quantity/quality of the tissue available or to its inaccessibility in some inoperable tumor types [73, 74]. It has been shown that among patients enrolled in tumor-directed treatments, only 70–90% of them had adequate tissue quantity/quality to achieve a successful molecular profile [75]. The technology used in our study permits tumor molecular profile analysis from a limited quantity of genetic material. Hence, in our cohort more than 97% of the tumor samples were successfully analyzed.

Furthermore, such analysis can provide an immense quantity of genetic data that needs to be appropriately analyzed and interpreted. Thus, the role of bioinformatics analysis is becoming major to provide accurate molecular analysis results [76]. Moreover, standardization of variant annotation and reporting could facilitate the understanding of the results obtained and increase their reliability. In our experience, in the majority of cases with findings associated to off-label treatments recommendations, the long lasting procedures required for the off-label approval of the suggested treatment from the local National Drug Organization for Medicines (“EOF”), or for clinical trial enrollment, often challenged the utilization of the results, especially in cases with advanced disease, requiring immediate management.

While, it is standard practice to perform accurate pre- and post-test counseling prior to a genetic testing for hereditary cancer susceptibility, this is not the case for somatic mutation analysis [77]. However, it is critical that patients referred for genetic tumour analysis to be accurately informed of the need for and potential outcomes of such testing. In addition, patients should be informed of the possibility that a variant in a gene with known germline mutations may be identified and that variants detected in a high percentage (> 40%) are considered germline suspicious. Since this analysis cannot distinguish between germline and somatic variants, clarification of the origin of a variant requires analysis of the patient's healthy tissue, usually blood or saliva.

In our cohort, 17 patients with a family history of cancer, requested blood analysis for suspicious germline variant identified in tissue. In 14 of them (82.35%) the germline origin of the tissue alteration was confirmed (Additional file 13: Table S9).

#### Immunotherapy biomarkers

Analysis of the tumor’s molecular profile useful as it is, it seems to be just another piece of the puzzle, since comprehensive tumor profile should include both biomarkers to guide treatment decision making for both targeted therapy as well as for immunotherapy. Thus, the physician having more biomarkers in his disposition could better comprehend the tumor’s biology and decide whether targeted therapy or immunotherapy matches better in each case. In our cohort analysis of biomarkers for both immunotherapy and targeted therapy, was requested in 395 patients, with TMB being the most common immunotherapy biomarker requested. All three biomarkers’ analysis was successful in 191 cases.

25.20% of the 381 patients tested had a TMB value > 10muts/MB and thus were eligible for ICI treatment. The median TMB values observed in our population were slightly increased compared to those observed in previous studies [57, 78]. This could be attributed to methodological differences and to the fact that in the majority of cases the patients analyzed have received more than one treatment lines, commonly chemotherapy, which is known to increase tumor’s mutation load [79]. Similarly, to our study a TMB positivity rate of 21.1% was observed in a recent study analyzing immunotherapy biomarkers in 48.782 clinical samples [80]. TMB has emerged as a promising biomarker of response to such treatments, and several clinical trials have shown that both blood and tissue samples TMB can effectively be used [23, 25, 27, 81]. Moreover, the recent approval of anti-PD1 treatment Pembrolizumab for metastatic cancer patients harboring a TMB value > 10mut/ΜΒ renders the analysis of such biomarker indispensable for treatment selection strategy.

However, this biomarker has also limitations since TMB calculation methods can differ between different assays, while the gene content of the methodology used seems to affect the TMB values obtained [82–84]. Furthermore, the cut-off values for this marker are not yet fully established. All these issues are addressed from the International harmonization initiatives led by Friends of Cancer Research (FOCR) and the Qualitätssicherungs-Initiative Pathologie (QuIP) [82–84].

Concerning the other immunotherapy biomarkers, analyzed in this study (PD-L1 and MSI), they could assist in a more accurate patients’ selection for treatment with checkpoint inhibitors. PD-L1 expression, measured by immunohistochemistry methods is the most widely used biomarker and the first to be approved for treatment with checkpoint inhibitors [15]. Nevertheless, it is not applicable in many tumor types, and its sensitivity and specificity in identifying patients eligible for immunotherapy have also been questioned [15, 16, 27, 85–87]. Moreover, while MSI analysis seems to be an appropriate biomarker, its low incidence in the majority of tumor types limits its clinical utility in the majority of neoplasms. In our cohort microsatellite instability was observed in just 3.88% of the cases; thus, it cannot stand alone as an immunotherapy biomarker, rendering the addition of other biomarkers indispensable to increase the number of patients who could benefit from such treatments.

The incidence of TMB positivity is superior to that of MSI (25.20% compared to 3.88%). Furthermore, in 21.88% (42/193) of the MSI stable cases, a TMB value of > 10muts/MB was observed; thus, these patients could receive ICI based on the TMB result only. Moreover, no association between TMB and PD-L1 values was observed. This is in agreement with previous studies, indicating lack of association between median values of these biomarkers. However, in accordance to a recent study, a higher TMB positivity rate was observed in the PD-L1 positive group [80]. The TMB positivity rate among the PD-L1 positive patients was 33.77% (26/77) compared to 15.79% (18/114) in the PD-L1 negative group (p = 0.005). Importantly, it has been reported that patients with positive values for both TMB and PD-L1 could have greater benefit from such treatment compared to those showing positivity for only one of these biomarkers [55, 56]. Collectively, among the 191 patients with all three immunotherapy biomarkers tested, ICIs option based on TMB result could be considered in 44 patients (23.04%), 26 of them with simultaneous PD-L1 positivity.

**Fig. 13:**
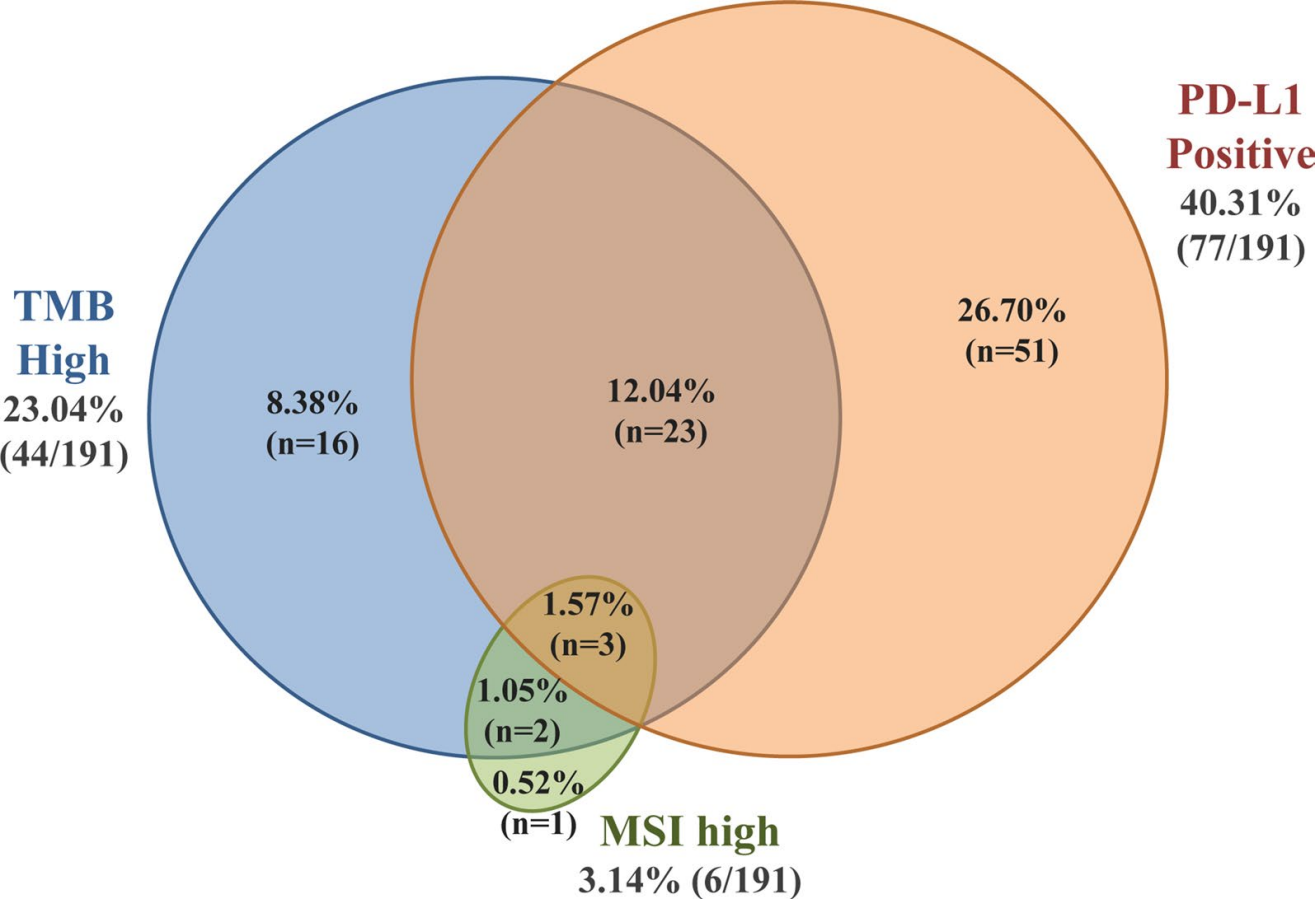
Venn Diagram showing the correlation among the three immunotherapy biomarkers tested (PD-L1, TMB, MSI)

As it can be seen in the Venn diagram (Fig. 13) showing the correlation among these biomarkers in 191 patients tested for all three biomarkers, 50.26% of the cases had at least one positive biomarker. A positive result for both PD-L1 and TMB was seen in 13.61% of the cases (with simultaneous MSI high result in 3 cases). In 2 patients concomitant TMB and MSI high values were observed (1.05%). An additional 35.60% % of the patients could receive immunotherapy-based one either TMB or PDL-1 or MSI positivity (8.38%, 26.70%, 0.52% respectively).

The analysis of immunotherapy biomarkers, though, does not seem to be the only determinant of response to ICI, since the tumor mutational status also seems to have a significant influence on the probability of response. For example, several studies have shown the reduced efficacy of ICIs in Non-Small Cell Lung cancer patients harboring *EGFR* mutations and *ALK* rearrangements [55, 88, 89]. The absence of such targetable alteration could direct the treatment strategy to immunotherapy in these malignancies. In addition, it has been shown that alterations in certain genes, such as *KRAS, TP53, MET, ARID1A* and others are enriched in immunotherapy responsive patients. Thus, their identification could lead to such treatment option [90–92].

Moreover, alterations in DNA repair genes such as the MMR genes, *POLE* and HR genes have been shown to have a positive predictive effect and are correlated to increased TMB values [93–96]. In contrast, other gene alterations such as *JAK1/2* and *STK11*/LKB1, *KEAP1* and *PTEN* mutations are related to resistance to PD-1 Blockade [90, 97–99]. Interestingly, in our study, 2 of the patients with TMB high values and one patient with PD-L1 positive result also harbored an *STK11* mutation. In none of these cases, immunotherapy response was achieved.

Thus, the addition of immunotherapy biomarkers to tumor molecular profiling seems to be a one-way road in order to achieve a comprehensive tumor characterization and provide the right treatment option for each patient. Moreover, the simultaneous analysis of such biomarkers, leads to the increase of patients with an on-label treatment recommendation by 22.92%. By combining immunotherapy and targeted therapy biomarkers, 71.35% of the patients analyzed received information related to on-label or off-label treatments. This is obviously improved compared to the 50.52% of on/off-label biomarkers achieved by analyzing only the molecular profile of the tumor in the same patient cohort.

Nowadays it seems that the tissue is not the issue anymore, since NGS technological advantages permit the simultaneous analysis of many targets from limited tissue material, achieving to analyze up to 97% of the tissue samples as in the present study. The challenge, though, when these analyses are performed is their implementation in clinical practice. Thus, the results obtained must be appropriately comprehended and adopted for the designation of the treatment selection strategy, which can be achieved through inter-discipline collaboration. To this regard, of great use would be the presence of a multidisciplinary Molecular Tumor Board that could assist in the accurate interpretation of the findings obtained from such complex NGS analysis and provide therapeutic recommendations based on all available clinical data for each individual patient [100–102].

## Conclusions

The NGS analysis conducted in this study offered actionable information (Tier1 and 2) in 77.87% of the 610 patients with tumor molecular profile analysis available. Moreover, simultaneous analysis for targeted therapy and immunotherapy biomarkers resulted in a better tumor characterization and provided actionable information in 83.25% of the 191 patients tested, with one to two patients being eligible for ICI treatment based on the biomarkers’ analysis. Thus, the comprehensive analysis of these biomarkers increased the number of patients with a treatment-related finding and contributed to a more individualized approach for cancer treatment. In conclusion, the present study has shown that the implementation of molecular profiling using appropriate pan-cancer panels in clinical practice is feasible. Of significance, is the appropriate comprehension of the molecular results obtained from such analysis and their proper utilization for designing the treatment selection strategy, which can be achieved through inter-discipline collaboration.

## Supplementary Information

**Additional file 1: Table S1**. Title: Genomic Regions and fusions analyzed with the 24 and 50 gene panels. **Additional file 2: Table S2**. Title: Gene alterations detected by the 161 gene panel. Description: The frequency of the different mutation types (SNVs, indels, CNV, fusions) for each gene is reported. **Additional file 3: Table S3**. Title: Alterations identified by the 161 gene panel in the 610 patients analyzed. Description: Genomic alterations and level of evidence of the variants detected in the 610 patients analyzed. The tumor type, age of diagnosis and gender are also reported. **Additional file 4: Table S4**. Title: Biomarker's summary in the 610 patients included in the study. Description: Patients' categorization based on TIER classification of their most clinically significant variant and immunotherapy biomarkers’ results are reported. **Additional file 5: Figure S1**. Title and description: Pancreatic cancer patients' categorization based on TIER classification of their most clinically significant variant. A. Pancreatic cancer patients' categorization based on TIER classification of their most clinically significant variant. Patients were categorized in the following categories: No Biomarker: Patients with no biomarker available, 1B: Patients harboring biomarkers with strong evidence of correlation to treatment, 2C.1 KRAS: Patients with a single finding in the KRAS gene, 2C.1: Patients with biomarkers related to off-label treatment. B. Percentage of patients with On-label and off-label mutations identified and the type of alterations detected. Genes of the homologous recombination complex are labeled in blue. **Additional file 6: Figure S2**. Title and description: Lung cancer patients' categorization based on TIER classification. A. Lung cancer patients' categorization based on TIER classification of their most clinically significant variant. The following categories were used:, 1A.1: Patients with biomarkers related to on-label treatment, 1B: Patients harboring biomarkers with strong evidence of correlation to treatment, 2C.1: Patients with biomarkers related to off-label treatment, 1A.2R; 2C.1: Patients harboring a KRAS mutation related to resistance to treatment plus an off-label, 1A.2R: Patients harboring a KRAS mutation related to EGFR TKIs resistance, 2.C.2: Patients B. % of patients with On-label and off-label mutations identified and the type of alterations detected. Genes of the homologous recombination complex are labeled in blue. **Additional file 7: Figure S3**. Title and description: Breast cancer patients' categorization based on the TIER classification. A. Patients' categorization based on TIER classification of their most clinically significant variant. The following categories were used: No Biomarker: Patients with no biomarker available, 1A.1: Patients with biomarkers related to on-label treatment, 2C.1: Patients with biomarkers related to off-label treatment, 2C.2: Patients with biomarkers related to clinical trials, 2D: Patients with biomarkers with preclinical evidence. B. Percentage of patients with On-label and off-label mutations identified and the type of alterations detected. Genes of the homologous recombination complex are labeled in blue. **Additional file 8: Figure S4**. Title and description: Colorectal cancer patients' categorization based on TIER classification. A. Patients' categorization based on TIER classification of their most clinically significant variant. Patients were categorized in the following categories: No Mutation, 1A.1: Patients with no mutation in KRAS/NRAS genes with or without other variations, 1A.2: Patients with biomarkers included in professional guidelines, 1A.1R: Patients harboring either KRAS or NRAS mutations related to resistance to treatment. B. % of patients with on-label and off-label mutations identified and the type of alterations detected. **Additional file 9: Table S5**. Title and description: Alterations that would have been detected if two hotspot panels of 24 and 50 genes respectively, had been used in the 610 patients analyzed. **Additional file 10: Table S6**. Title and description: Simulation results of the alterations that would have been identified if the gene set of the 161 gene NGS panel was used in the PCAWG samples. **Additional file 11: Table S7**. Title and description: Simulation results of the alterations that would have been identified if the gene set of the 514 gene NGS panel was used in the PCAWG samples. **Additional file 12: Table S8**. Title and Description: TMB, PD-L1 and MSI results in the 395 patients with at least one immunotherapy biomarker requested. **Additional file 13: Table S9**. Title and Description: Patients with suspicious hereditary pathogenic findings detected in tumor tissue.

## Data Availability

All data generated or analyzed during this study are included in this published article and its supplementary information files.

## Abbreviations

NGS: Next Generation Sequencing technology
IHC: Immunohistochemistry
MSI: Microsatellite instability
TMB: Tumor Mutational Burden
SNVs: Single nucleotide Variants
Indels: Insertion–deletions
CNVs: Copy Number Variations
AMP: Association for Molecular Pathology
ACMG: The American College of Medical Genetics
ASCO: The American Society of Clinical Oncology
CAP: The College of American Pathologists
TC: Tumor cells
IC: Immune cells
ICI: Immune checkpoint inhibitors
ESMO: European Society for Medical Oncology
NCCN: National Comprehensive Cancer Network
PARPi: PARP inhibitors

## References

[1] Nakagawa H, Wardell CP, Furuta M, Taniguchi H, Fujimoto A (2015). Cancer whole-genome sequencing: present and future. Oncogene.

[2] Jürgensmeier JM, Eder JP, Herbst RS (2014). New strategies in personalized medicine for solid tumors: molecular markers and clinical trial designs. Clin Cancer Res.

[3] Garinet S, Laurent-Puig P, Blons H, Oudart J-B (2018). Current and future molecular testing in NSCLC, what can we expect from new sequencing technologies?. J Clin Med.

[4] Malone ER, Oliva M, Sabatini PJB, Stockley TL, Siu LL (2020). Molecular profiling for precision cancer therapies. Genome Med.

[5] Zimmer K, Kocher F, Spizzo G, Salem M, Gastl G, Seeber A (2019). Treatment according to molecular profiling in relapsed/refractory cancer patients: a review focusing on latest profiling studies. Comput Struct Biotechnol J.

[6] Tsimberidou A-M, Iskander NG, Hong DS, Wheler JJ, Falchook GS, Fu S (2012). Personalized medicine in a phase I clinical trials program: the MD Anderson Cancer Center initiative. Clin Cancer Res.

[7] Stockley TL, Oza AM, Berman HK, Leighl NB, Knox JJ, Shepherd FA (2016). Molecular profiling of advanced solid tumors and patient outcomes with genotype-matched clinical trials: the Princess Margaret IMPACT/COMPACT trial. Genome Med.

[8] Coco S, Truini A, Vanni I, Dal Bello MG, Alama A, Rijavec E (2015). Next generation sequencing in non-small cell lung cancer: new avenues toward the personalized medicine. Curr Drug Targets.

[9] Pestana RC, Sen S, Hobbs BP, Hong DS (2020). Histology-agnostic drug development—considering issues beyond the tissue. Nat Rev Clin Oncol..

[10] Hong DS, Fakih MG, Strickler JH, Desai J, Durm GA, Shapiro GI (2020). KRASG12C inhibition with Sotorasib in advanced solid tumors. N Engl J Med.

[11] Nagasaka M, Li Y, Sukari A, Ou S-HI, Al-Hallak MN, Azmi AS (2020). KRAS G12C Game of Thrones, which direct KRAS inhibitor will claim the iron throne?. Cancer Treat Rev.

[12] Sotorasib Edges Closer to Approval. Cancer Discov. 2021. 10.1158/2159-8290.CD-NB2021-0309. 10.1158/2159-8290.CD-NB2021-0309 33547148

[13] Srinivasan M, Sedmak D, Jewell S (2002). Effect of fixatives and tissue processing on the content and integrity of nucleic acids. Am J Pathol.

[14] Ascierto PA, Bifulco C, Palmieri G, Peters S, Sidiropoulos N (2019). Preanalytic variables and tissue stewardship for reliable next-generation sequencing (NGS) clinical analysis. J Mol Diagn.

[15] Kim H, Chung J-H (2019). PD-L1 testing in non-small cell lung cancer: past, present, and future. J Pathol Transl Med.

[16] Shen X, Zhao B (2018). Efficacy of PD-1 or PD-L1 inhibitors and PD-L1 expression status in cancer: meta-analysis. BMJ.

[17] Le DT, Uram JN, Wang H, Bartlett BR, Kemberling H, Eyring AD (2015). PD-1 Blockade in tumors with mismatch-repair deficiency. N Engl J Med.

[18] Le DT, Durham JN, Smith KN, Wang H, Bartlett BR, Aulakh LK (2017). Mismatch repair deficiency predicts response of solid tumors to PD-1 blockade. Science.

[19] Zhao P, Li L, Jiang X, Li Q (2019). Mismatch repair deficiency/microsatellite instability-high as a predictor for anti-PD-1/PD-L1 immunotherapy efficacy. J Hematol Oncol.

[20] Bonneville R, Krook MA, Kautto EA, Miya J, Wing MR, Chen H-Z, et al. Landscape of microsatellite instability across 39 cancer types. JCO Precis Oncol. 2017;2017:PO.17.00073. 10.1200/PO.17.00073 PMC5972025 29850653

[21] Walk EE, Yohe SL, Beckman A, Schade A, Zutter MM, Pfeifer J (2020). The cancer immunotherapy biomarker testing landscape. Arch Pathol Lab Med.

[22] Signorelli D, Giannatempo P, Grazia G, Aiello MM, Bertolini F, Mirabile A (2019). Patients selection for immunotherapy in solid tumors: overcome the naïve vision of a single biomarker. Biomed Res Int.

[23] Yarchoan M, Hopkins A, Jaffee EM (2017). Tumor mutational burden and response rate to PD-1 inhibition. N Engl J Med.

[24] Campesato LF, Barroso-Sousa R, Jimenez L, Correa BR, Sabbaga J, Hoff PM (2015). Comprehensive cancer-gene panels can be used to estimate mutational load and predict clinical benefit to PD-1 blockade in clinical practice. Oncotarget.

[25] Samstein RM, Lee C-H, Shoushtari AN, Hellmann MD, Shen R, Janjigian YY (2019). Tumor mutational load predicts survival after immunotherapy across multiple cancer types. Nat Genet.

[26] Wu Y, Xu J, Du C, Wu Y, Xia D, Lv W (2019). The predictive value of tumor mutation burden on efficacy of immune checkpoint inhibitors in cancers: a systematic review and meta-analysis. Front Oncol.

[27] Sholl LM, Hirsch FR, Hwang D, Botling J, Lopez-Rios F, Bubendorf L, et al. The promises and challenges of tumor mutation burden as an immunotherapy biomarker: a perspective from the International Association for the Study of Lung Cancer Pathology Committee. J Thorac Oncol. 2020;15(9):1409–24. 10.1016/j.jtho.2020.05.019 PMC8363213 32522712

[28] Galuppini F, Dal Pozzo CA, Deckert J, Loupakis F, Fassan M, Baffa R (2019). Tumor mutation burden: from comprehensive mutational screening to the clinic. Cancer Cell Int.

[29] Marabelle A, Fakih M, Lopez J, Shah M, Shapira-Frommer R, Nakagawa K, et al. Association of tumour mutational burden with outcomes in patients with advanced solid tumours treated with pembrolizumab: prospective biomarker analysis of the multicohort, open-label, phase 2 KEYNOTE-158 study. Lancet Oncol. 2020;21(10):1353–65. 10.1016/S1470-2045(20)30445-9 32919526

[30] Li MM, Datto M, Duncavage EJ, Kulkarni S, Lindeman NI, Roy S (2017). Standards and guidelines for the interpretation and reporting of sequence variants in cancer: a joint consensus recommendation of the Association for Molecular Pathology, American Society of Clinical Oncology, and College of American Pathologists. J Mol Diagn.

[31] Leichsenring J, Horak P, Kreutzfeldt S, Heining C, Christopoulos P, Volckmar A-L (2019). Variant classification in precision oncology. Int J Cancer.

[32] D’Haene N, Le Mercier M, De Nève N, Blanchard O, Delaunoy M, El Housni H (2015). Clinical validation of targeted next generation sequencing for colon and lung cancers. PLoS ONE.

[33] D’Haene N, Fontanges Q, De Nève N, Blanchard O, Melendez B, Delos M (2018). Clinical application of targeted next-generation sequencing for colorectal cancer patients: a multicentric Belgian experience. Oncotarget.

[34] Nemtsova MV, Kalinkin AI, Kuznetsova EB, Bure IV, Alekseeva EA, Bykov II (2020). Clinical relevance of somatic mutations in main driver genes detected in gastric cancer patients by next-generation DNA sequencing. Sci Rep.

[35] de Leng WWJ, Gadellaa-van Hooijdonk CG, Barendregt-Smouter FAS, Koudijs MJ, Nijman I, Hinrichs JWJ (2016). Targeted Next Generation Sequencing as a reliable diagnostic assay for the detection of somatic mutations in tumours using minimal DNA amounts from formalin fixed paraffin embedded material. PLoS ONE.

[36] Tsoulos N, Papadopoulou E, Metaxa-Mariatou V, Tsaousis G, Efstathiadou C, Tounta G (2017). Tumor molecular profiling of NSCLC patients using next generation sequencing. Oncol Rep.

[37] Campbell PJ, Getz G, Korbel JO, Stuart JM, Jennings JL, Stein LD (2020). Pan-cancer analysis of whole genomes. Nature.

[38] Goldman MJ, Craft B, Hastie M, Repečka K, McDade F, Kamath A (2020). Visualizing and interpreting cancer genomics data via the Xena platform. Nat Biotechnol.

[39] Goldman MJ, Zhang J, Fonseca NA, Cortés-Ciriano I, Xiang Q, Craft B (2020). A user guide for the online exploration and visualization of PCAWG data. Nat Commun.

[40] Dehghani M, Rosenblatt KP, Li L, Rakhade M, Amato RJ (2019). Validation and clinical applications of a comprehensive Next Generation Sequencing system for molecular characterization of solid cancer tissues. Front Mol Biosci.

[41] Marchetti A, Barberis M, Franco R, De Luca G, Pace MV, Staibano S (2017). Multicenter comparison of 22C3 PharmDx (Agilent) and SP263 (Ventana) assays to test PD-L1 expression for NSCLC patients to be treated with immune checkpoint inhibitors. J Thorac Oncol.

[42] Villaruz LC, Ancevski Hunter K, Kurland BF, Abberbock S, Herbst C, Dacic S (2019). Comparison of PD-L1 immunohistochemistry assays and response to PD-1/L1 inhibitors in advanced non-small-cell lung cancer in clinical practice. Histopathology.

[43] From the American Association of Neurological Surgeons (AANS), American Society of Neuroradiology (ASNR), Cardiovascular and Interventional Radiology Society of Europe (CIRSE), Canadian Interventional Radiology Association (CIRA), Congress of Neurological and WSO (WSO), Sacks D, Baxter B, Campbell BC V, Carpenter JS, Cognard C, et al. Multisociety consensus quality improvement revised consensus statement for endovascular therapy of acute ischemic stroke. Int J Stroke. 2018;13(6):612–32. 10.1177/1747493018778713 29786478

[44] Tretiakova M, Fulton R, Kocherginsky M, Long T, Ussakli C, Antic T (2018). Concordance study of PD-L1 expression in primary and metastatic bladder carcinomas: comparison of four commonly used antibodies and RNA expression. Mod Pathol.

[45] Ning Y-M, Suzman D, Maher VE, Zhang L, Tang S, Ricks T (2017). FDA approval summary: atezolizumab for the treatment of patients with progressive advanced urothelial carcinoma after platinum-containing chemotherapy. Oncologist.

[46] Rasmussen JH, Lelkaitis G, Håkansson K, Vogelius IR, Johannesen HH, Fischer BM (2019). Intratumor heterogeneity of PD-L1 expression in head and neck squamous cell carcinoma. Br J Cancer.

[47] Schmid P, Adams S, Rugo HS, Schneeweiss A, Barrios CH, Iwata H (2018). Atezolizumab and nab-paclitaxel in advanced triple-negative breast cancer. N Engl J Med.

[48] Mateo J, Lord CJ, Serra V, Tutt A, Balmaña J, Castroviejo-Bermejo M (2019). A decade of clinical development of PARP inhibitors in perspective. Ann Oncol Off J Eur Soc Med Oncol.

[49] Faraoni I, Graziani G (2018). Role of BRCA mutations in cancer treatment with poly(ADP-ribose) polymerase (PARP) inhibitors. Cancers (Basel)..

[50] De Roock W, Claes B, Bernasconi D, De Schutter J, Biesmans B, Fountzilas G (2010). Effects of KRAS, BRAF, NRAS, and PIK3CA mutations on the efficacy of cetuximab plus chemotherapy in chemotherapy-refractory metastatic colorectal cancer: a retrospective consortium analysis. Lancet Oncol.

[51] Douillard J-Y, Oliner KS, Siena S, Tabernero J, Burkes R, Barugel M (2013). Panitumumab-FOLFOX4 treatment and RAS mutations in colorectal cancer. N Engl J Med.

[52] Louis DN, Perry A, Reifenberger G, von Deimling A, Figarella-Branger D, Cavenee WK (2016). The 2016 World Health Organization Classification of tumors of the central nervous system: a summary. Acta Neuropathol.

[53] Kristensen BW, Priesterbach-Ackley LP, Petersen JK, Wesseling P (2019). Molecular pathology of tumors of the central nervous system. Ann Oncol Off J Eur Soc Med Oncol.

[54] Chaudhary R, Quagliata L, Martin JP, Alborelli I, Cyanam D, Mittal V (2018). A scalable solution for tumor mutational burden from formalin-fixed, paraffin-embedded samples using the Oncomine Tumor Mutation Load Assay. Transl lung cancer Res.

[55] Rizvi H, Sanchez-Vega F, La K, Chatila W, Jonsson P, Halpenny D (2018). Molecular determinants of response to anti-programmed cell death (PD)-1 and anti-programmed death-ligand 1 (PD-L1) blockade in patients with non-small-cell lung cancer profiled with targeted Next-Generation Sequencing. J Clin Oncol.

[56] Yarchoan M, Albacker LA, Hopkins AC, Montesion M, Murugesan K, Vithayathil TT, et al. PD-L1 expression and tumor mutational burden are independent biomarkers in most cancers. JCI Insight. 2019;4(6):e126908. 10.1172/jci.insight.126908 PMC6482991 30895946

[57] Chalmers ZR, Connelly CF, Fabrizio D, Gay L, Ali SM, Ennis R (2017). Analysis of 100,000 human cancer genomes reveals the landscape of tumor mutational burden. Genome Med.

[58] Luchini C, Bibeau F, Ligtenberg MJL, Singh N, Nottegar A, Bosse T (2019). ESMO recommendations on microsatellite instability testing for immunotherapy in cancer, and its relationship with PD-1/PD-L1 expression and tumour mutational burden: a systematic review-based approach. Ann Oncol Off J Eur Soc Med Oncol.

[59] Goodman AM, Kato S, Bazhenova L, Patel SP, Frampton GM, Miller V (2017). Tumor mutational burden as an independent predictor of response to immunotherapy in diverse cancers. Mol Cancer Ther.

[60] Klempner SJ, Fabrizio D, Bane S, Reinhart M, Peoples T, Ali SM (2020). Tumor mutational burden as a predictive biomarker for response to immune checkpoint inhibitors: a review of current evidence. Oncologist.

[61] Wheler JJ, Janku F, Naing A, Li Y, Stephen B, Zinner R (2016). Cancer therapy directed by comprehensive genomic profiling: a single center study. Cancer Res.

[62] Remon J, Dienstmann R (2018). Precision oncology: separating the wheat from the chaff. ESMO Open.

[63] Mosele F, Remon J, Mateo J, Westphalen C, Barlesi F, Lolkema M, et al. Journal pre-proof recommendations for the use of next-generation sequencing (NGS) for patients with metastatic cancers: a report from the ESMO Precision Medicine Working Group Recommendations for the use of next-generation sequencing (NGS) for patients wi. Ann Oncol. 2020. 10.1016/j.annonc.2020.07.014. 10.1016/j.annonc.2020.07.014 32853681

[64] Cocco E, Scaltriti M, Drilon A (2018). NTRK fusion-positive cancers and TRK inhibitor therapy. Nat Rev Clin Oncol.

[65] Drilon A, Siena S, Ou S-HI, Patel M, Ahn MJ, Lee J (2017). Safety and antitumor activity of the multitargeted pan-TRK, ROS1, and ALK inhibitor entrectinib: combined results from two phase I trials (ALKA-372–001 and STARTRK-1). Cancer Discov.

[66] Drilon A, Laetsch TW, Kummar S, DuBois SG, Lassen UN, Demetri GD (2018). Efficacy of larotrectinib in TRK fusion-positive cancers in adults and children. N Engl J Med.

[67] Solomon JP, Benayed R, Hechtman JF, Ladanyi M (2019). Identifying patients with NTRK fusion cancer. Ann Oncol Off J Eur Soc Med Oncol..

[68] Yi T, Feng Y, Sundaram R, Tie Y, Zheng H, Qian Y (2019). Antitumor efficacy of PARP inhibitors in homologous recombination deficient carcinomas. Int J Cancer.

[69] Mateo J, Porta N, Bianchini D, McGovern U, Elliott T, Jones R (2020). Olaparib in patients with metastatic castration-resistant prostate cancer with DNA repair gene aberrations (TOPARP-B): a multicentre, open-label, randomised, phase 2 trial. Lancet Oncol.

[70] Yi M, Dong B, Qin S, Chu Q, Wu K, Luo S (2019). Advances and perspectives of PARP inhibitors. Exp Hematol Oncol.

[71] Pilié PG, Gay CM, Byers LA, O’Connor MJ, Yap TA (2019). PARP inhibitors: extending benefit beyond BRCA-mutant cancers. Clin Cancer Res.

[72] Thomas A, Murai J, Pommier Y (2018). The evolving landscape of predictive biomarkers of response to PARP inhibitors. J Clin Invest.

[73] Do H, Dobrovic A (2015). Sequence artifacts in DNA from formalin-fixed tissues: causes and strategies for minimization. Clin Chem.

[74] Zhang P, Lehmann BD, Shyr Y, Guo Y (2017). The utilization of formalin fixed-paraffin-embedded specimens in high throughput genomic studies. Int J Genomics.

[75] Nawrocki S (2018). Molecular profiling of tumours for precision oncology—high hopes versus reality. Contemp Oncol (Poznan, Poland).

[76] Singer J, Irmisch A, Ruscheweyh H-J, Singer F, Toussaint NC, Levesque MP (2019). Bioinformatics for precision oncology. Brief Bioinform.

[77] Gori S, Barberis M, Bella MA, Buttitta F, Capoluongo E, Carrera P (2019). Recommendations for the implementation of BRCA testing in ovarian cancer patients and their relatives. Crit Rev Oncol Hematol.

[78] Alexandrov LB, Nik-Zainal S, Wedge DC, Aparicio SAJR, Behjati S, Biankin AV (2013). Signatures of mutational processes in human cancer. Nature.

[79] McGranahan N, Furness AJS, Rosenthal R, Ramskov S, Lyngaa R, Saini SK (2016). Clonal neoantigens elicit T cell immunoreactivity and sensitivity to immune checkpoint blockade. Science.

[80] Huang RSP, Haberberger J, Severson E, Duncan DL, Hemmerich A, Edgerly C (2020). A pan-cancer analysis of PD-L1 immunohistochemistry and gene amplification, tumor mutation burden and microsatellite instability in 48,782 cases. Mod Pathol.

[81] Gandara DR, Paul SM, Kowanetz M, Schleifman E, Zou W, Li Y (2018). Blood-based tumor mutational burden as a predictor of clinical benefit in non-small-cell lung cancer patients treated with atezolizumab. Nat Med.

[82] Stenzinger A, Allen JD, Maas J, Stewart MD, Merino DM, Wempe MM (2019). Tumor mutational burden standardization initiatives: recommendations for consistent tumor mutational burden assessment in clinical samples to guide immunotherapy treatment decisions. Genes Chromosomes Cancer.

[83] Merino DM, McShane LM, Fabrizio D, Funari V, Chen S-J, White JR (2020). Establishing guidelines to harmonize tumor mutational burden (TMB): in silico assessment of variation in TMB quantification across diagnostic platforms: phase I of the Friends of Cancer Research TMB Harmonization Project. J Immunother Cancer..

[84] Stenzinger A, Endris V, Budczies J, Merkelbach-Bruse S, Kazdal D, Dietmaier W (2020). Harmonization and standardization of panel-based tumor mutational burden measurement: real-world results and recommendations of the quality in pathology study. J Thorac Oncol.

[85] Sunshine J, Taube JM (2015). PD-1/PD-L1 inhibitors. Curr Opin Pharmacol.

[86] Cottrell TR, Taube JM (2018). PD-L1 and emerging biomarkers in immune checkpoint blockade therapy. Cancer J.

[87] Apolo AB (2016). PDL1: The illusion of an ideal biomarker. Eur Urol Focus.

[88] Lee CK, Man J, Lord S, Cooper W, Links M, Gebski V (2018). Clinical and molecular characteristics associated with survival among patients treated with checkpoint inhibitors for advanced non-small cell lung carcinoma: a systematic review and meta-analysis. JAMA Oncol.

[89] Gainor JF, Shaw AT, Sequist LV, Fu X, Azzoli CG, Piotrowska Z (2016). EGFR mutations and ALK rearrangements are associated with low response rates to PD-1 pathway blockade in non-small cell lung cancer: a retrospective analysis. Clin Cancer Res.

[90] Skoulidis F, Goldberg ME, Greenawalt DM, Hellmann MD, Awad MM, Gainor JF (2018). STK11/LKB1 mutations and PD-1 inhibitor resistance in KRAS-mutant lung adenocarcinoma. Cancer Discov.

[91] Kauffmann-Guerrero D, Tufman A, Kahnert K, Bollmann BA, Reu S, Syunyaeva Z (2020). Response to checkpoint inhibition in non-small cell lung cancer with molecular driver alterations. Oncol Res Treat.

[92] Okamura R, Kato S, Lee S, Jimenez RE, Sicklick JK, Kurzrock R. ARID1A alterations function as a biomarker for longer progression-free survival after anti-PD-1/PD-L1 immunotherapy. J Immunother Cancer. 2020;8(1):e000438. 10.1136/jitc-2019-000438 PMC7057434 32111729

[93] Kim JH, Kim SY, Baek JY, Cha YJ, Ahn JB, Kim HS, et al. A Phase II study of avelumab monotherapy in patients with mismatch repair-deficient/microsatellite instability-high or POLE-mutated metastatic or unresectable colorectal cancer. Cancer Res Treat. 2020;52(4):1135–44. 10.4143/crt.2020.218 PMC7577804 32340084

[94] Picard E, Verschoor CP, Ma GW, Pawelec G (2020). Relationships between immune landscapes, genetic subtypes and responses to immunotherapy in colorectal cancer. Front Immunol.

[95] Peyraud F, Italiano A. Combined PARP inhibition and immune checkpoint therapy in solid tumors. Cancers (Basel). 2020;12(6):1502. 10.3390/cancers12061502 PMC7352466 32526888

[96] Pellegrino B, Musolino A, Llop-Guevara A, Serra V, De Silva P, Hlavata Z (2020). Homologous recombination repair deficiency and the immune response in breast cancer: a literature review. Transl Oncol.

[97] Shin DS, Zaretsky JM, Escuin-Ordinas H, Garcia-Diaz A, Hu-Lieskovan S, Kalbasi A (2017). Primary resistance to PD-1 blockade mediated by JAK1/2 mutations. Cancer Discov.

[98] Zaretsky JM, Garcia-Diaz A, Shin DS, Escuin-Ordinas H, Hugo W, Hu-Lieskovan S (2016). Mutations associated with acquired resistance to PD-1 blockade in melanoma. N Engl J Med.

[99] Piro G, Carbone C, Carbognin L, Pilotto S, Ciccarese C, Iacovelli R (2019). Revising PTEN in the era of immunotherapy: new perspectives for an old story. Cancers (Basel).

[100] Knepper TC, Bell GC, Hicks JK, Padron E, Teer JK, Vo TT (2017). Key lessons learned from Moffitt’s molecular tumor board: the Clinical Genomics Action Committee experience. Oncologist.

[101] van der Velden DL, van Herpen CML, van Laarhoven HWM, Smit EF, Groen HJM, Willems SM (2017). Molecular tumor boards: current practice and future needs. Ann Oncol Off J Eur Soc Med Oncol.

[102] Kato S, Kim KH, Lim HJ, Boichard A, Nikanjam M, Weihe E (2020). Real-world data from a molecular tumor board demonstrates improved outcomes with a precision N-of-One strategy. Nat Commun.

