# Supplementary material for: Comprehensive tumor molecular profile analysis in clinical practice
Source: BMC Med Genomics. 2021 Apr 14;14:105. doi: 10.1186/s12920-021-00952-9 (PMC8045191; doi:10.1186/s12920-021-00952-9)
Supplement: Supplementary file 1 — Additional file 1: Table S1. Title: Genomic Regions and fusions analyzed with the 24 and 50 gene panels. Additional file 2: Table S2. Title: Gene alterations detected by the 161 gene panel. Description: The frequency of the different mutation types (SNVs, indels, CNV, fusions) for each gene is reported. Additional file 3: Table S3. Title: Alterations identified by the 161 gene panel in the 610 patients analyzed. Description: Genomic alterations and level of evidence of the variants detected in the 610 patients analyzed. The tumor type, age of diagnosis and gender are also reported. Additional file 4: Table S4. Title: Biomarker's summary in the 610 patients included in the study. Description: Patients' categorization based on TIER classification of their most clinically significant variant and immunotherapy biomarkers’ results are reported. Additional file 5: Figure S1. Title and description: Pancreatic cancer patients' categorization based on TIER classification of their most clinically significant variant. A. Pancreatic cancer patients' categorization based on TIER classification of their most clinically significant variant. Patients were categorized in the following categories: No Biomarker: Patients with no biomarker available, 1B: Patients harboring biomarkers with strong evidence of correlation to treatment, 2C.1 KRAS: Patients with a single finding in the KRAS gene, 2C.1: Patients with biomarkers related to off-label treatment. B. Percentage of patients with On-label and off-label mutations identified and the type of alterations detected. Genes of the homologous recombination complex are labeled in blue. Additional file 6: Figure S2. Title and description: Lung cancer patients' categorization based on TIER classification. A. Lung cancer patients' categorization based on TIER classification of their most clinically significant variant. The following categories were used:, 1A.1: Patients with biomarkers related to on-label treatment, 1B: Patients harboring bioma [file 12920_2021_952_MOESM1_ESM.docx]

Comprehensive tumor molecular profile analysis in clinical practice

Mustafa Özdoğan^1^, Eirini Papadopoulou^2*^, Nikolaos Tsoulos^2^, Aikaterini Tsantikidi^2^, Vasiliki-Metaxa Mariatou^2^, Georgios Tsaousis^2^, Evgenia Kapeni^2^, Evgenia Bourkoula^2^, Dimitrios Fotiou^2^, Georgios Kapetsis^2^, Ioannis Boukovinas^3^, Nikolaos Touroutoglou^4^, Athanasios Fassas^5^, Achilleas Adamidis^5^, Paraskevas Kosmidis^6^, Dimitrios Trafalis^7^, Eleni Galani^8^, George Lypas ^9^, Bülent Orhan^10^, Sualp Tansan^11^, Tahsin Özatlı^12^, Onder Kırca^1^, Okan Çakır^13^, George Nasioulas^2^

1. Division of Medical Oncology, Memorial Hospital, Antalya, Turkey.
2. Genekor Medical S.A, Athens, Greece
3. Bioclinic Thessaloniki, Thessaloniki, GREECE
4. Department of Medical Oncology, Interbalkan Medical Center, Thessaloniki, Greece.
5. St. Luke’s Hospital, Thessaloniki, Greece
6. Second Department of Medical Oncology, Hygeia Hospital, Athens, Greece.
7. Henry Dunant Hospital Center
8. Second Department of Medical Oncology, "Metropolitan" Hospital, Piraeus, Greece.
9. Department of Genetic Oncology/Medical Oncology, Hygeia Hospital, Athens, Greece.
10. Department of Medical Oncology, Ceylan International Hospital, Bursa, Turkey.
11. Tansan Oncology, Istanbul, Turkey.
12. Istinye University Hospital, Istanbul, Turkey.
13. Applied Health Sciences, Edinburgh Napier University, EH11 4BN Scotland, United Kingdom

Additional file 1.: Table S1 Genomic Regions and fusions analyzed with the 24 and 50 gene panels

| **24 gene panel design** | | | | **50 gene panel** | | | | **Fusions analyzed** |
| --- | --- | --- | --- | --- | --- | --- | --- | --- |
| **Chromosome** | **Start** | **End** | **Gene** | **Chromosome** | **Start** | **End** | **Gene** |  |
| chr1 | 115252190 | 115252305 | NRAS | chr1 | 43814968 | 43815086 | MPL | EML4-ALK.E6A19.COSF1296.1 |
| chr1 | 115256504 | 115256584 | NRAS | chr1 | 115252185 | 115252269 | NRAS | EML4-ALK.E13A20.AB462411 |
| chr1 | 115258689 | 115258774 | NRAS | chr1 | 115256504 | 115256584 | NRAS | EML4-ALK.E18A20.COSF487.1 |
| chr1 | 162724289 | 162724421 | DDR2 | chr1 | 115258689 | 115258774 | NRAS | EML4-ALK.E2A20.COSF478.1 |
| chr1 | 162724505 | 162724631 | DDR2 | chr2 | 29432572 | 29432680 | ALK | EML4-ALK.E20A20.COSF409.1 |
| chr1 | 162729596 | 162729706 | DDR2 | chr2 | 29443607 | 29443729 | ALK | EML4-ALK.E6aA20.AB374361 |
| chr1 | 162740116 | 162740247 | DDR2 | chr2 | 209113103 | 209113206 | IDH1 | KIF5B-ALK.K15A20.COSF1381 |
| chr1 | 162741785 | 162741920 | DDR2 | chr2 | 212288904 | 212288990 | ERBB4 | KIF5B-ALK.K17A20.COSF1257 |
| chr1 | 162743218 | 162743347 | DDR2 | chr2 | 212530051 | 212530180 | ERBB4 | KIF5B-ALK.K24A20.COSF1058 |
| chr1 | 162745447 | 162745576 | DDR2 | chr2 | 212576799 | 212576910 | ERBB4 | KLC1-ALK.K9A20.COSF1276 |
| chr1 | 162748336 | 162748452 | DDR2 | chr2 | 212578288 | 212578415 | ERBB4 | EML4-ALK.E14A20.COSF1064 |
| chr2 | 29432654 | 29432735 | ALK | chr2 | 212587133 | 212587239 | ERBB4 | KIF5B-RET.K15R11.COSF1255 |
| chr2 | 29443572 | 29443702 | ALK | chr2 | 212589764 | 212589867 | ERBB4 | KIF5B-RET.K24R11.COSF1262 |
| chr2 | 29445208 | 29445320 | ALK | chr2 | 212652719 | 212652806 | ERBB4 | KIF5B-RET.K24R8.COSF1236 |
| chr2 | 212288904 | 212288990 | ERBB4 | chr2 | 212812075 | 212812169 | ERBB4 | CCDC6-RET.C1R12.COSF1271 |
| chr2 | 212530051 | 212530180 | ERBB4 | chr3 | 10183765 | 10183854 | VHL | KIF5B-RET.K15R12.COSF1232 |
| chr2 | 212576799 | 212576910 | ERBB4 | chr3 | 10188186 | 10188306 | VHL | KIF5B-RET.K16R12.COSF1230 |
| chr2 | 212578288 | 212578415 | ERBB4 | chr3 | 10191418 | 10191527 | VHL | KIF5B-RET.K22R12.COSF1253 |
| chr2 | 212587133 | 212587239 | ERBB4 | chr3 | 37067207 | 37067333 | MLH1 | KIF5B-RET.K23R12.COSF1234 |
| chr2 | 212589764 | 212589867 | ERBB4 | chr3 | 41266029 | 41266147 | CTNNB1 | CD74-ROS1.C6R32.COSF1202 |
| chr2 | 212652719 | 212652806 | ERBB4 | chr3 | 178916775 | 178916881 | PIK3CA | SDC4-ROS1.S2R32.COSF1265 |
| chr2 | 212812075 | 212812169 | ERBB4 | chr3 | 178916931 | 178917035 | PIK3CA | SDC4-ROS1.S4R32.COSF1278 |
| chr3 | 41266029 | 41266147 | CTNNB1 | chr3 | 178921464 | 178921570 | PIK3CA | SLC34A2-ROS1.S4R32.COSF1197 |
| chr3 | 178936023 | 178936105 | PIK3CA | chr3 | 178927405 | 178927525 | PIK3CA | SDC4-ROS1.S2R34 |
| chr3 | 178938787 | 178938918 | PIK3CA | chr3 | 178927901 | 178927986 | PIK3CA | SDC4-ROS1.S4R34.COSF1280 |
| chr3 | 178951996 | 178952097 | PIK3CA | chr3 | 178928069 | 178928160 | PIK3CA | CD74-ROS1.C6R34.COSF1200 |
| chr4 | 1803551 | 1803653 | FGFR3 | chr3 | 178936023 | 178936105 | PIK3CA | EZR-ROS1.E10R34.COSF1267 |
| chr4 | 1806081 | 1806187 | FGFR3 | chr3 | 178938787 | 178938918 | PIK3CA | SLC34A2-ROS1.S4R34.COSF1198 |
| chr4 | 1807833 | 1807930 | FGFR3 | chr3 | 178947818 | 178947896 | PIK3CA | LRIG3-ROS1.L16R35.COSF1269 |
| chr4 | 1808311 | 1808399 | FGFR3 | chr3 | 178951996 | 178952097 | PIK3CA | GOPC-ROS1.G8R35.COSF1139 |
| chr4 | 1808881 | 1809006 | FGFR3 | chr3 | 178952140 | 178952237 | PIK3CA | GOPC-ROS1.G4R36.COSF1188 |
| chr4 | 153245410 | 153245492 | FBXW7 | chr4 | 1803551 | 1803653 | FGFR3 | CEL-NTRK1.C7N7 |
| chr4 | 153247277 | 153247369 | FBXW7 | chr4 | 1806081 | 1806187 | FGFR3 | NFASC-NTRK1.N18N10 |
| chr4 | 153249355 | 153249477 | FBXW7 | chr4 | 1807833 | 1807930 | FGFR3 | IRF2BP2-NTRK1.I1N10 |
| chr4 | 153250852 | 153250926 | FBXW7 | chr4 | 1808311 | 1808399 | FGFR3 | TFG-NTRK1.T5N10 |
| chr4 | 153258901 | 153259023 | FBXW7 | chr4 | 1808881 | 1809006 | FGFR3 | SQSTM1-NTRK1.S5N10 |
| chr7 | 55227950 | 55228057 | EGFR | chr4 | 55140981 | 55141103 | PDGFRA | SSBP2-NTRK1.S12N12 |
| chr7 | 55241635 | 55241729 | EGFR | chr4 | 55144101 | 55144195 | PDGFRA | NTRK1-DYNC2H1.N17D86 |
| chr7 | 55242411 | 55242540 | EGFR | chr4 | 55144538 | 55144653 | PDGFRA | CD74-NTRK1.C3N13 |
| chr7 | 55248974 | 55249100 | EGFR | chr4 | 55152024 | 55152154 | PDGFRA | MPRIP-NTRK1.M14N13 |
| chr7 | 55259507 | 55259628 | EGFR | chr4 | 55561654 | 55561784 | KIT | MPRIP-NTRK1.M18N13 |
| chr7 | 116339615 | 116339701 | MET | chr4 | 55592157 | 55592246 | KIT | MPRIP-NTRK1.M21N13 |
| chr7 | 116340155 | 116340270 | MET | chr4 | 55593417 | 55593513 | KIT | CUX1-RET.C10R12 |
| chr7 | 116403131 | 116403251 | MET | chr4 | 55593575 | 55593695 | KIT | HIP1-ALK.H28A20 |
| chr7 | 116411878 | 116412061 | MET | chr4 | 55594170 | 55594279 | KIT | HIP1-ALK.H21A20 |
| chr7 | 116417427 | 116417542 | MET | chr4 | 55595496 | 55595562 | KIT | TPR-ALK.T15A20 |
| chr7 | 116423407 | 116423492 | MET | chr4 | 55597436 | 55597524 | KIT | HMBS.ENCTRL.E8E9 |
| chr7 | 140453102 | 140453221 | BRAF | chr4 | 55599280 | 55599358 | KIT | TBP.ENCTRL.E3E4 |
| chr7 | 140481391 | 140481515 | BRAF | chr4 | 55602673 | 55602751 | KIT | MYC.ENCTRL.E2E3 |
| chr8 | 38282140 | 38282254 | FGFR1 | chr4 | 55946088 | 55946208 | KDR | LMNA.ENCTRL.E3E4 |
| chr8 | 38285851 | 38285975 | FGFR1 | chr4 | 55946250 | 55946371 | KDR | ALK.5p_NM_004304.4.e5e6 |
| chr9 | 139397762 | 139397879 | NOTCH1 | chr4 | 55953775 | 55953860 | KDR | ALK.3p_NM_004304.4.e23e24 |
| chr9 | 139399337 | 139399447 | NOTCH1 | chr4 | 55955078 | 55955168 | KDR | RET.5p_NM_020975.4.e6e7 |
| chr10 | 89624207 | 89624300 | PTEN | chr4 | 55960976 | 55961059 | KDR | RET.3p_NM_020975.4.e18e19 |
| chr10 | 89685258 | 89685374 | PTEN | chr4 | 55962444 | 55962548 | KDR | ROS1.5p_NM_002944.2.e11e12 |
| chr10 | 89711804 | 89711932 | PTEN | chr4 | 55972952 | 55973071 | KDR | ROS1.3p_NM_002944.2.e38e39 |
| chr10 | 89717503 | 89717620 | PTEN | chr4 | 55979574 | 55979655 | KDR | NTRK1.5p.eNST00000392302.e2e3 |
| chr10 | 89717666 | 89717780 | PTEN | chr4 | 55980238 | 55980359 | KDR | NTRK1.3p.eNST00000392302.e17e18 |
| chr10 | 89720695 | 89720747 | PTEN | chr4 | 153245410 | 153245492 | FBXW7 | EML4-ALK.E6A19.COSF1296 |
| chr10 | 123257952 | 123258045 | FGFR2 | chr4 | 153247277 | 153247369 | FBXW7 | EML4-ALK.E2A20.COSF479 |
| chr10 | 123274721 | 123274835 | FGFR2 | chr4 | 153249355 | 153249477 | FBXW7 | EML4-ALK.E2A20.COSF479.1 |
| chr10 | 123279417 | 123279544 | FGFR2 | chr4 | 153250852 | 153250926 | FBXW7 | EML4-ALK.E15A20.COSF413 |
| chr10 | 123279607 | 123279713 | FGFR2 | chr4 | 153258901 | 153259023 | FBXW7 | EML4-ALK.E17A20.COSF1367 |
| chr11 | 533812 | 533930 | HRAS | chr5 | 112173871 | 112173962 | APC | EML4-ALK.E17A20.COSF1367.1 |
| chr11 | 534220 | 534308 | HRAS | chr5 | 112174557 | 112174666 | APC | EML4-ALK.E17A20.COSF1366 |
| chr12 | 25378549 | 25378658 | KRAS | chr5 | 112175143 | 112175268 | APC | EML4-ALK.E17A20.COSF1366.1 |
| chr12 | 25380260 | 25380364 | KRAS | chr5 | 112175315 | 112175443 | APC | EML4-ALK.E14A20.COSF477 |
| chr12 | 25398186 | 25398304 | KRAS | chr5 | 112175567 | 112175703 | APC | EML4-ALK.E14A20.COSF477.1 |
| chr12 | 133249741 | 133249878 | POLE | chr5 | 112175740 | 112175862 | APC | EML4-ALK.E13A20.COSF1062 |
| chr12 | 133250082 | 133250218 | POLE | chr5 | 112175920 | 112176035 | APC | EML4-ALK.E13A20.COSF1062.1 |
| chr12 | 133250249 | 133250385 | POLE | chr5 | 149433593 | 149433692 | CSF1R | EML4-ALK.E13A20.COSF408 |
| chr12 | 133251918 | 133252056 | POLE | chr5 | 149452991 | 149453073 | CSF1R | EML4-ALK.E18A20.COSF487 |
| chr12 | 133252082 | 133252221 | POLE | chr5 | 170837501 | 170837616 | NPM1 | EML4-ALK.E2A20.COSF478 |
| chr12 | 133252311 | 133252436 | POLE | chr7 | 55211044 | 55211126 | EGFR | EML4-ALK.E20A20.COSF409 |
| chr12 | 133252667 | 133252801 | POLE | chr7 | 55221792 | 55221919 | EGFR | EML4-ALK.E20A20.COSF730 |
| chr12 | 133253117 | 133253254 | POLE | chr7 | 55232962 | 55233053 | EGFR | EML4-ALK.E20A20.COSF730.1 |
| chr14 | 105246445 | 105246583 | AKT1 | chr7 | 55241635 | 55241729 | EGFR | EML4-ALK.E6aA20.COSF411 |
| chr15 | 66727413 | 66727529 | MAP2K1 | chr7 | 55242411 | 55242540 | EGFR | EML4-ALK.E6bA20.COSF412 |
| chr17 | 7573923 | 7574035 | TP53 | chr7 | 55248965 | 55249090 | EGFR | EML4-ALK.E6bA20.COSF412.1 |
| chr17 | 7577015 | 7577151 | TP53 | chr7 | 55249122 | 55249245 | EGFR | EML4-ALK.E6bA20.AB374362 |
| chr17 | 7577508 | 7577612 | TP53 | chr7 | 55259507 | 55259628 | EGFR | KIF5B-ALK.K15A20.COSF1060 |
| chr17 | 7578180 | 7578298 | TP53 | chr7 | 116339615 | 116339701 | MET | SLC34A2-ROS1.S13R32.COSF1259 |
| chr17 | 7578352 | 7578483 | TP53 | chr7 | 116340155 | 116340270 | MET | SLC34A2-ROS1.S13R34.COSF1261 |
| chr17 | 7578516 | 7578601 | TP53 | chr7 | 116403131 | 116403251 | MET | TPM3-ROS1.T7R35.COSF1273 |
| chr17 | 7579350 | 7579485 | TP53 | chr7 | 116411829 | 116411942 | MET | ITGB7.ENCTRL.E14E15 |
| chr17 | 7579853 | 7579960 | TP53 | chr7 | 116411983 | 116412097 | MET | ETV6-NTRK3.E4N15.COSF823.1 |
| chr17 | 37880212 | 37880340 | ERBB2 | chr7 | 116417427 | 116417542 | MET | ETV6-NTRK3.E5N15.COSF571.1 |
| chr17 | 37880953 | 37881061 | ERBB2 | chr7 | 116423407 | 116423492 | MET | AFAP1-NTRK2.A14N12 |
| chr17 | 37881324 | 37881453 | ERBB2 | chr7 | 128845063 | 128845188 | SMO | AGBL4-NTRK2.A6N16 |
| chr18 | 48575099 | 48575213 | SMAD4 | chr7 | 128845957 | 128846063 | SMO | BTBD1-NTRK3.B4N14 |
| chr18 | 48581190 | 48581302 | SMAD4 | chr7 | 128846337 | 128846419 | SMO | COX5A-NTRK3.C1N15 |
| chr18 | 48584551 | 48584678 | SMAD4 | chr7 | 128850269 | 128850363 | SMO | ETV6-NTRK3.E4N14 |
| chr18 | 48586251 | 48586361 | SMAD4 | chr7 | 128851499 | 128851612 | SMO | ETV6-NTRK3.E5N14 |
| chr18 | 48591814 | 48591931 | SMAD4 | chr7 | 140453102 | 140453221 | BRAF | NACC2-NTRK2.N4N13 |
| chr18 | 48593399 | 48593519 | SMAD4 | chr7 | 140481391 | 140481515 | BRAF | QKI-NTRK2.Q6N16 |
| chr18 | 48603028 | 48603119 | SMAD4 | chr7 | 148508706 | 148508791 | EZH2 | SQSTM1-NTRK2.S5N17 |
| chr18 | 48604658 | 48604774 | SMAD4 | chr8 | 38282140 | 38282254 | FGFR1 | TRIM24-NTRK2.T12N15 |
| chr19 | 1206977 | 1207104 | STK11 | chr8 | 38285851 | 38285975 | FGFR1 | VCL-NTRK2.V16N12 |
| chr19 | 1220480 | 1220603 | STK11 | chr9 | 5073729 | 5073857 | JAK2 |  |
| chr19 | 1221236 | 1221332 | STK11 | chr9 | 21970940 | 21971066 | CDKN2A |  |
| chr19 | 1223014 | 1223144 | STK11 | chr9 | 21971090 | 21971219 | CDKN2A |  |
|  |  |  |  | chr9 | 80409375 | 80409498 | GNAQ |  |
|  |  |  |  | chr9 | 133738294 | 133738378 | ABL1 |  |
|  |  |  |  | chr9 | 133747442 | 133747530 | ABL1 |  |
|  |  |  |  | chr9 | 133748279 | 133748417 | ABL1 |  |
|  |  |  |  | chr9 | 133750307 | 133750405 | ABL1 |  |
|  |  |  |  | chr9 | 139390764 | 139390885 | NOTCH1 |  |
|  |  |  |  | chr9 | 139397762 | 139397879 | NOTCH1 |  |
|  |  |  |  | chr9 | 139399337 | 139399447 | NOTCH1 |  |
|  |  |  |  | chr10 | 43609066 | 43609182 | RET |  |
|  |  |  |  | chr10 | 43609874 | 43610010 | RET |  |
|  |  |  |  | chr10 | 43613794 | 43613893 | RET |  |
|  |  |  |  | chr10 | 43615546 | 43615687 | RET |  |
|  |  |  |  | chr10 | 43617314 | 43617433 | RET |  |
|  |  |  |  | chr10 | 89624207 | 89624300 | PTEN |  |
|  |  |  |  | chr10 | 89685258 | 89685374 | PTEN |  |
|  |  |  |  | chr10 | 89692813 | 89692920 | PTEN |  |
|  |  |  |  | chr10 | 89711804 | 89711932 | PTEN |  |
|  |  |  |  | chr10 | 89717503 | 89717620 | PTEN |  |
|  |  |  |  | chr10 | 89717666 | 89717780 | PTEN |  |
|  |  |  |  | chr10 | 89720695 | 89720747 | PTEN |  |
|  |  |  |  | chr10 | 89720784 | 89720900 | PTEN |  |
|  |  |  |  | chr10 | 123257952 | 123258045 | FGFR2 |  |
|  |  |  |  | chr10 | 123274721 | 123274835 | FGFR2 |  |
|  |  |  |  | chr10 | 123279417 | 123279544 | FGFR2 |  |
|  |  |  |  | chr10 | 123279607 | 123279713 | FGFR2 |  |
|  |  |  |  | chr11 | 533812 | 533930 | HRAS |  |
|  |  |  |  | chr11 | 534220 | 534308 | HRAS |  |
|  |  |  |  | chr11 | 108117765 | 108117865 | ATM |  |
|  |  |  |  | chr11 | 108119815 | 108119891 | ATM |  |
|  |  |  |  | chr11 | 108123515 | 108123618 | ATM |  |
|  |  |  |  | chr11 | 108137931 | 108138025 | ATM |  |
|  |  |  |  | chr11 | 108155083 | 108155180 | ATM |  |
|  |  |  |  | chr11 | 108170456 | 108170556 | ATM |  |
|  |  |  |  | chr11 | 108172362 | 108172467 | ATM |  |
|  |  |  |  | chr11 | 108173630 | 108173703 | ATM |  |
|  |  |  |  | chr11 | 108180902 | 108180960 | ATM |  |
|  |  |  |  | chr11 | 108200915 | 108200993 | ATM |  |
|  |  |  |  | chr11 | 108204634 | 108204684 | ATM |  |
|  |  |  |  | chr11 | 108205731 | 108205816 | ATM |  |
|  |  |  |  | chr11 | 108206523 | 108206628 | ATM |  |
|  |  |  |  | chr11 | 108218015 | 108218144 | ATM |  |
|  |  |  |  | chr11 | 108225549 | 108225632 | ATM |  |
|  |  |  |  | chr11 | 108236042 | 108236140 | ATM |  |
|  |  |  |  | chr11 | 108236186 | 108236285 | ATM |  |
|  |  |  |  | chr12 | 25378549 | 25378658 | KRAS |  |
|  |  |  |  | chr12 | 25380260 | 25380364 | KRAS |  |
|  |  |  |  | chr12 | 25398186 | 25398304 | KRAS |  |
|  |  |  |  | chr12 | 112888118 | 112888228 | PTPN11 |  |
|  |  |  |  | chr12 | 112926835 | 112926961 | PTPN11 |  |
|  |  |  |  | chr12 | 121431371 | 121431459 | HNF1A |  |
|  |  |  |  | chr12 | 121432010 | 121432099 | HNF1A |  |
|  |  |  |  | chr13 | 28592579 | 28592663 | FLT3 |  |
|  |  |  |  | chr13 | 28602275 | 28602379 | FLT3 |  |
|  |  |  |  | chr13 | 28608227 | 28608348 | FLT3 |  |
|  |  |  |  | chr13 | 28610093 | 28610184 | FLT3 |  |
|  |  |  |  | chr13 | 48919223 | 48919312 | RB1 |  |
|  |  |  |  | chr13 | 48923139 | 48923255 | RB1 |  |
|  |  |  |  | chr13 | 48941601 | 48941724 | RB1 |  |
|  |  |  |  | chr13 | 48942597 | 48942711 | RB1 |  |
|  |  |  |  | chr13 | 48953753 | 48953874 | RB1 |  |
|  |  |  |  | chr13 | 48955525 | 48955605 | RB1 |  |
|  |  |  |  | chr13 | 49027105 | 49027178 | RB1 |  |
|  |  |  |  | chr13 | 49033827 | 49033934 | RB1 |  |
|  |  |  |  | chr13 | 49037846 | 49037932 | RB1 |  |
|  |  |  |  | chr13 | 49039149 | 49039232 | RB1 |  |
|  |  |  |  | chr14 | 105241433 | 105241519 | AKT1 |  |
|  |  |  |  | chr14 | 105246445 | 105246583 | AKT1 |  |
|  |  |  |  | chr15 | 90631824 | 90631954 | IDH2 |  |
|  |  |  |  | chr16 | 68835602 | 68835697 | CDH1 |  |
|  |  |  |  | chr16 | 68846024 | 68846151 | CDH1 |  |
|  |  |  |  | chr16 | 68847199 | 68847302 | CDH1 |  |
|  |  |  |  | chr17 | 7573923 | 7574035 | TP53 |  |
|  |  |  |  | chr17 | 7577015 | 7577151 | TP53 |  |
|  |  |  |  | chr17 | 7577508 | 7577612 | TP53 |  |
|  |  |  |  | chr17 | 7578180 | 7578298 | TP53 |  |
|  |  |  |  | chr17 | 7578352 | 7578483 | TP53 |  |
|  |  |  |  | chr17 | 7578516 | 7578601 | TP53 |  |
|  |  |  |  | chr17 | 7579350 | 7579485 | TP53 |  |
|  |  |  |  | chr17 | 7579853 | 7579960 | TP53 |  |
|  |  |  |  | chr17 | 37880212 | 37880340 | ERBB2 |  |
|  |  |  |  | chr17 | 37880953 | 37881061 | ERBB2 |  |
|  |  |  |  | chr17 | 37881324 | 37881453 | ERBB2 |  |
|  |  |  |  | chr18 | 48575099 | 48575213 | SMAD4 |  |
|  |  |  |  | chr18 | 48575556 | 48575677 | SMAD4 |  |
|  |  |  |  | chr18 | 48581190 | 48581302 | SMAD4 |  |
|  |  |  |  | chr18 | 48584551 | 48584678 | SMAD4 |  |
|  |  |  |  | chr18 | 48586251 | 48586361 | SMAD4 |  |
|  |  |  |  | chr18 | 48591814 | 48591931 | SMAD4 |  |
|  |  |  |  | chr18 | 48593399 | 48593519 | SMAD4 |  |
|  |  |  |  | chr18 | 48603028 | 48603119 | SMAD4 |  |
|  |  |  |  | chr18 | 48604658 | 48604774 | SMAD4 |  |
|  |  |  |  | chr19 | 1206977 | 1207104 | STK11 |  |
|  |  |  |  | chr19 | 1220310 | 1220450 | STK11 |  |
|  |  |  |  | chr19 | 1220480 | 1220603 | STK11 |  |
|  |  |  |  | chr19 | 1221236 | 1221332 | STK11 |  |
|  |  |  |  | chr19 | 1223014 | 1223144 | STK11 |  |
|  |  |  |  | chr19 | 3118881 | 3118973 | GNA11 |  |
|  |  |  |  | chr19 | 17945616 | 17945734 | JAK3 |  |
|  |  |  |  | chr19 | 17947986 | 17948074 | JAK3 |  |
|  |  |  |  | chr19 | 17954134 | 17954225 | JAK3 |  |
|  |  |  |  | chr20 | 36031666 | 36031769 | SRC |  |
|  |  |  |  | chr20 | 57484396 | 57484504 | GNAS |  |
|  |  |  |  | chr20 | 57484562 | 57484672 | GNAS |  |
|  |  |  |  | chr22 | 24133953 | 24134064 | SMARCB1 |  |
|  |  |  |  | chr22 | 24143200 | 24143311 | SMARCB1 |  |
|  |  |  |  | chr22 | 24145477 | 24145598 | SMARCB1 |  |
|  |  |  |  | chr22 | 24176259 | 24176391 | SMARCB1 |  |

Additional file 2: Table S2 Gene alterations detected by the 161 gene panel.

| **GENE** | **SNVs/indels** | **Copy Number Variations (CNVs)** | **Gene Fusions** | **Total** | **% mutations** |
| --- | --- | --- | --- | --- | --- |
| TP53 | 229 | 1 |  | 230 | 24.57% |
| KRAS | 147 | 5 |  | 152 | 16.24% |
| PIK3CA | 69 | 2 | 1 | 72 | 7.69% |
| CDKN2A | 21 |  |  | 21 | 2.24% |
| NF1 | 19 |  |  | 19 | 2.03% |
| BRAF | 15 |  | 1 | 16 | 1.71% |
| FGFR1 | 3 | 5 | 8 | 16 | 1.71% |
| MYC |  | 15 |  | 15 | 1.60% |
| ATM | 13 | 1 |  | 14 | 1.50% |
| EGFR | 13 |  |  | 13 | 1.39% |
| ESR1 | 7 | 2 | 2 | 11 | 1.18% |
| ARID1A | 10 |  |  | 10 | 1.07% |
| ERBB2 | 3 | 7 |  | 10 | 1.07% |
| CTNNB1 | 9 |  |  | 9 | 0.96% |
| IDH1 | 9 |  |  | 9 | 0.96% |
| NOTCH1 | 8 |  | 1 | 9 | 0.96% |
| RNF43 | 9 |  |  | 9 | 0.96% |
| SMARCA4 | 9 |  |  | 9 | 0.96% |
| STK11 | 9 |  |  | 9 | 0.96% |
| CCND1 | 1 | 7 |  | 8 | 0.85% |
| PIK3R1 | 8 |  |  | 8 | 0.85% |
| PTEN | 8 |  |  | 8 | 0.85% |
| SETD2 | 8 |  |  | 8 | 0.85% |
| AKT1 | 6 | 1 |  | 7 | 0.75% |
| FBXW7 | 7 |  |  | 7 | 0.75% |
| NRAS | 7 |  |  | 7 | 0.75% |
| POLE | 7 |  |  | 7 | 0.75% |
| RET | 4 |  | 3 | 7 | 0.75% |
| BRCA1 | 6 |  |  | 6 | 0.64% |
| BRCA2 | 6 |  |  | 6 | 0.64% |
| CDK4 | 1 | 5 |  | 6 | 0.64% |
| CREBBP | 6 |  |  | 6 | 0.64% |
| PTCH1 | 6 |  |  | 6 | 0.64% |
| RB1 | 5 | 1 |  | 6 | 0.64% |
| TERT | 6 |  |  | 6 | 0.64% |
| AR | 2 | 2 | 1 | 5 | 0.53% |
| ATRX | 5 |  |  | 5 | 0.53% |
| CDK12 | 5 |  |  | 5 | 0.53% |
| ERG |  |  | 5 | 5 | 0.53% |
| PALB2 | 5 |  |  | 5 | 0.53% |
| CCND3 |  | 4 |  | 4 | 0.43% |
| CCNE1 |  | 4 |  | 4 | 0.43% |
| FGF19 |  | 4 |  | 4 | 0.43% |
| FGFR2 | 1 | 3 |  | 4 | 0.43% |
| JAK3 | 4 |  |  | 4 | 0.43% |
| MDM2 |  | 4 |  | 4 | 0.43% |
| MET | 1 | 2 | 1 | 4 | 0.43% |
| NF2 | 4 |  |  | 4 | 0.43% |
| PDGFRA | 1 | 3 |  | 4 | 0.43% |
| RSPO2 |  |  | 4 | 4 | 0.43% |
| APC | 3 |  |  | 3 | 0.32% |
| BAP1 | 3 |  |  | 3 | 0.32% |
| CHEK2 | 3 |  |  | 3 | 0.32% |
| FGF3 |  | 3 |  | 3 | 0.32% |
| GNAS | 3 |  |  | 3 | 0.32% |
| KIT | 1 | 2 |  | 3 | 0.32% |
| MSH2 | 2 | 1 |  | 3 | 0.32% |
| NTRK2 |  | 3 |  | 3 | 0.32% |
| PDGFRA |  | 3 |  | 3 | 0.32% |
| PPP2R1A | 3 |  |  | 3 | 0.32% |
| SF3B1 | 3 |  |  | 3 | 0.32% |
| SMAD4 | 3 |  |  | 3 | 0.32% |
| TSC1 | 3 |  |  | 3 | 0.32% |
| TSC2 | 3 |  |  | 3 | 0.32% |
| AKT3 | 1 | 1 |  | 2 | 0.21% |
| ALK |  | 1 | 1 | 2 | 0.21% |
| ATR | 2 |  |  | 2 | 0.21% |
| AXL |  | 2 |  | 2 | 0.21% |
| CCND2 | 1 | 1 |  | 2 | 0.21% |
| CDK2 |  | 2 |  | 2 | 0.21% |
| FANCA | 2 |  |  | 2 | 0.21% |
| FANCD2 | 2 |  |  | 2 | 0.21% |
| FGFR3 | 2 |  |  | 2 | 0.21% |
| GNA11 | 2 |  |  | 2 | 0.21% |
| MSH6 | 2 |  |  | 2 | 0.21% |
| MYCL |  | 2 |  | 2 | 0.21% |
| NBN | 2 |  |  | 2 | 0.21% |
| NFE2L2 | 2 |  |  | 2 | 0.21% |
| NOTCH3 | 2 |  |  | 2 | 0.21% |
| PMS2 | 2 |  |  | 2 | 0.21% |
| PTPN11 | 2 |  |  | 2 | 0.21% |
| RAD50 | 2 |  |  | 2 | 0.21% |
| RICTOR |  | 2 |  | 2 | 0.21% |
| SMARCB1 | 1 | 1 |  | 2 | 0.21% |
| ABL1 | 1 |  |  | 1 | 0.11% |
| AKT2 |  | 1 |  | 1 | 0.11% |
| CDK6 |  | 1 |  | 1 | 0.11% |
| CDKN2B | 1 |  |  | 1 | 0.11% |
| ERBB3 | 1 |  |  | 1 | 0.11% |
| FBXO11 | 1 |  |  | 1 | 0.11% |
| FGFR4 |  | 1 |  | 1 | 0.11% |
| FOXL2 | 1 |  |  | 1 | 0.11% |
| GATA3 | 1 |  |  | 1 | 0.11% |
| GNAQ | 1 |  |  | 1 | 0.11% |
| HNF1A | 1 |  |  | 1 | 0.11% |
| HRAS | 1 |  |  | 1 | 0.11% |
| IGFR1 |  | 1 |  | 1 | 0.11% |
| JAK2 | 1 |  |  | 1 | 0.11% |
| KNSTRN | 1 |  |  | 1 | 0.11% |
| MDM4 |  | 1 |  | 1 | 0.11% |
| MLH1 | 1 |  |  | 1 | 0.11% |
| MRE11A | 1 |  |  | 1 | 0.11% |
| MTOR | 1 |  |  | 1 | 0.11% |
| MYCN |  | 1 |  | 1 | 0.11% |
| NOTCH2 | 1 |  |  | 1 | 0.11% |
| PPARG |  |  | 1 | 1 | 0.11% |
| ROS1 |  |  | 1 | 1 | 0.11% |
| RSPO3 |  |  | 1 | 1 | 0.11% |
| SLX4 | 1 |  |  | 1 | 0.11% |
| SPOP | 1 |  |  | 1 | 0.11% |
| VHL | 1 |  |  | 1 | 0.11% |

Additional file 3: Table S3 Alterations identified by the 161 gene panel in the 610 patients analyzed.

| **Sample** | **Tumor Type** | **Gender** | **Age of diagnosis** | **Genomic Alterations (GAs)** | **Biomarkers with on label and off label treatments associated (LoE 1A-2C.1)** | **Biomarkers with Lower LoE  (2C.2-3)** | **Patients categorization based on the biomarker with the Highest LoE** |
| --- | --- | --- | --- | --- | --- | --- | --- |
| 1 | Prostate cancer | male | 56 | NOTCH1 c.1710C>A(p.C570*), NOTCH1 c.7027C>T(p.Q2343*), TP53 c.644G>A(p.S215N) |  | NOTCH1;2C.2;NOTCH1;2C.2;TP53;2C.2; | 2C.2 |
| 2 | Endometrial cancer | male | 68 | TP53 c.706T>C(p.Y236H), FBXW7 c.1394G>A(p.R465H ) |  | TP53;2C.2;FBXW7;2C.2; | 2C.2 |
| 3 | Breast cancer | female | 45 | ATM c.8404C>T(p.Q2802*), BRCA1 c.213-1G>A , NF2 c.439C>T(p.Q147*), TSC2 c.1432C>T(p.Q478*), | ATM;2C.1;BRCA1;1A.1;TSC2;2C.1; | NF2;3; | 1A.1 |
| 4 | Breast cancer | female | 78 | TP53 c.266_267delCCinsTG(p.P89L) |  | TP53;2C.2; | 2C.2 |
| 5 | Pancreatic cancer | female | 82 | KRAS c.35G>A(p.G12D) | KRAS;2C.1; |  | 2C.1 |
| 6 | Endometrial cancer | female | 69 | MET amplification, TP53 c.557A>G(p.D186G), BRCA2 c.7831G>A(p.D2611N) | BRCA2;2C.1;MET;2C.1; | TP53;2C.2; | 2C.1 |
| 7 | Lung cancer | male | 73 | KRAS c.35G>A(p.G12D), TP53 c.637C>T(p.R213*) | KRAS;1A.2R | TP53;2C.2; | 1A.2R |
| 8 | Lung cancer | female | 67 | NRAS c.182A>G(p.Q61R), TSC1 c.956T>A(p.L319*), TP53 c.524G>A(p.R175H) | TSC1;2C.1; | TP53;2C.2;NRAS;2C.2; | 2C.1 |
| 9 | Uterine ancer | female | 82 | TP53 c.314G>T(p.G105V) |  | TP53;2C.2; | 2C.2 |
| 10 | Ovarian cancer | female | 65 | nl |  |  | normal |
| 11 | Lung cancer | male | 45 | EGFR c.2235_2252delGGAATTA(p.E746_T751delinsFPI), CTNNB1 c.134C>T(p.S45F) | EGFR;1A.1; | CTNNB1;2C.2; | 1A.1 |
| 12 | kidney cancer | male | 32 | TP53 c.430C>T(p.Q144*) |  | TP53;2C.2; | 2C.2 |
| 13 | Colorectal cancer | male | 69 | TP53 c.799C>T ( p.R267W), TP53 c.916C>T ( p.R306*), TP53 c.691A>G(p.T231A) | KRAS/NRAS;1A.1; | TP53;2C.2;TP53;2C.2;TP53;2C.2; | 1A.1 |
| 14 | Colorectal cancer | male | 21 | nl | KRAS/NRAS;1A.1; |  | 1A.1 |
| 15 | Prostate cancer | male | 45 | nl |  |  | normal |
| 16 | Pancreatic cancer | female | 65 | nl |  |  | normal |
| 17 | Breast cancer | female | 74 | JAK2 c.1711G>A(p.G571S), TP53 c.524G>A(p.R175H) |  | TP53;2C.2; JAK2;2D | 2C.2 |
| 18 | Pancreatic cancer | female | 32 | KRAS c.35G>A(p.G12D), ERBB2 c.1998_1999delCT(p.L667fs*36), CDKN2A c.83_100delTGCGGGCGCTGCTGGAGG(p.V28_E33del ), TP53 c.818G>A(p.R273H) | KRAS;2C.1;ERBB2;2C.1; | CDKN2A;2D;TP53;2C.2; | 2C.1 |
| 19 | Colorectal cancer | female | 69 | nl | KRAS/NRAS;1A.1; |  | 1A.1 |
| 20 | Colorectal cancer | female | 87 | TP53 c.841_842dupGA(p.D281fs*65), APC c.3956delC(p.P1319fs*2) | KRAS/NRAS;1A.1; | TP53;2C.2;APC;2C.2 | 1A.1 |
| 21 | Pancreatic cancer | male | 32 | TP53 c.451C>T (p.Pro151Ser) |  | TP53;2C.2; | 2C.2 |
| 22 | Pancreatic cancer | female | 36 | nl |  |  | normal |
| 23 | Lung cancer | male | 48 | EGFR exon 19 c.2239_2251delTTAAGAGAAGCAAinsC(p.L747_T751delinsP), EGFR c.2369C>T(p.T790M), EGFR c.2390G>C(p.C797S), MET c.3335A>G(p.H1112R), PIK3CA c.1630A>G(p.T544A), APC c.7141C>T(p.Q2381*) , ATM c.3382C>T(p.Q1128), STK11 c.924G>A(p.W308*), STK11 c.3G>A(p.M1I), TP53 c.112C>T(p.Q38*) | EGFR;1A.1;EGFR;1A.1;EGFR;1A.2;MET;2C.1;PIK3CA;2C.1;ATM;2C.1;STK11;2C.1;STK11;2C.1; | APC;2C.2;TP53;2C.2; | 1A.1 |
| 24 | Cholangiocarcinoma | male | 59 | IDH1 c.394C>T(p.R132C) | IDH1;2C.1; |  | 2C.1 |
| 25 | Colorectal cancer | female | 56 | nl | KRAS/NRAS;1A.1; |  | 1A.1 |
| 26 | Unknown primary | female | 45 | TP53 c.1024C>T ( p.R342*) |  | TP53;2C.2; | 2C.2 |
| 27 | Lung cancer | female | 46 | VHL c.203C>T(p.S68L) |  | VHL;2C.2; | 2C.2 |
| 28 | Breast cancer | female | 43 | GATA3 c.1223_1224insA(p.P409fs) |  | GATA3;2D; | 2D |
| 29 | Ovarian cancer | female | 42 | KRAS c.35G>A (p.Gly12Asp), TP53 c.856G>A (p.Glu286Lys), JAK3 c.2164G>A(p.V722I) | KRAS;2C.1; | TP53;2C.2;JAK3;2D; | 2C.1 |
| 30 | Pancreatic cancer | female | 68 | nl |  |  | normal |
| 31 | Uknown | female | 76 | ESR1 c.1607T>C(p.L536P) |  | ESR1;2C.2; | 2C.2 |
| 32 | Uknown | female | 84 | JAK3 c.2164G>A(p.V722I) |  | JAK3;2D; | 2D |
| 33 | Pancreatic cancer | male | 59 | KRAS c.34G>C p.Gly12Arg, TP53 c.332T>A (p.Leu111Gln), CCNE1 amplification | KRAS;2C.1; | TP53;2C.2;CCNE1;2C.2; | 2C.1 |
| 34 | Endometrial cancer | male | 63 | CTNNB1 c.133T>C(p.S45P), PIK3CA c.3140A>G(p.H1047R) ,ESR1 c.1607T>C(p.L536P), PTEN c.281_290delACCCACCACA(p.N94fs*) | PIK3CA;2C.1;PTEN;2C.1; | CTNNB1;2C.1;ESR1;2C.2; | 2C.1 |
| 35 | Lung cancer | female | 71 | KRAS c.34G>T (p.Gly12Cys), TP53 c.524G>A (p.Arg175His), JAK3 c.2164G>A(p.V722I | KRAS;1A.2R | TP53;2C.2;JAK3;2D; | 1A.2R |
| 36 | Lung cancer | female | 86 | nl |  |  | normal |
| 37 | Ovarian cancer | female | 47 | TP53 c.796G>A(p.Gly266Glu) |  | TP53;2C.2; | 2C.2 |
| 38 | Breast cancer | female | 52 | FGFR2 amplification, TP53 c.329G>C ( p.R110P), TP53 loss, RB1 loss | FGFR2;2C.1; | TP53;2C.2;TP53;2C.2;RB1;2C.2; | 2C.1 |
| 39 | Ovarian cancer | female | 73 | TP53 c.1006G>T(p.E336*),TP53 c.772G>A(p.E258K) |  | TP53;2C.2;TP53;2C.2;TP53;2C.2; | 2C.2 |
| 40 | Breast cancer | female | 36 | BRAF c.1406G>C(p.G469A), PIK3CA c.3140A>G (H1047R), CCND1 amplification, TP53 c.839G>C(p.R280T), SF3B1 c.2098A>G(p.K700E) | PIK3CA;1A.1; | BRAF;2C.2;TP53;2C.2;SF3B1;3:CCND1;2C.2; | 1A.1 |
| 41 | Breast cancer | female | 81 | PIK3CA c.1633G>A(p.Ε545Κ) | PIK3CA;1A.1; |  | 1A.1 |
| 42 | Breast cancer | female | 72 | CCND1 amplification, PIK3CA amplification, TP53 c.637C>T(p.Arg213Ter) |  | TP53;2C.2;CCND1;2C.2;PIK3CA;2C.2; | 2C.2 |
| 43 | Breast cancer | female | 45 | PIK3CA c.3140A>G(p.His1047Arg), TP53 c.818G>A(p.Arg273His) , ERBB2 (HER2) amplification | PIK3CA;1A.1;ERBB2;1A.1; | TP53;2C.2; | 1A.1 |
| 44 | Lung cancer | male | 58 | EGFR c.2303G>T(p.S768I), ABL1 c.943A>G(p.T315A), SMARCB1 Loss, SMARCB1 c.1154G>A(p.W385*), SMARCB1 c.2164G>A, TP53 c.764_765delTCinsAT(p.I255N) | EGFR;2C.1;ABL1;2C.1; | SMARCB1;2D;SMARCB1;2D;TP53;2C.2; | 2C.1 |
| 45 | Cervical cancer | female | 63 | KRAS c.34 G>T(p.G12C) | KRAS;2C.1; |  | 2C.1 |
| 46 | Gastric cancer | male | 69 | nl |  |  | normal |
| 47 | Ovarian cancer | female | 54 | KRAS c.38 G>A(p.G13D), PIK3CA c.2702G>T(p.C901F), PIK3CA c .311C>G(p.P104R), JAK3 c.2164G>A(p.V722I) | KRAS;2C.1;PIK3CA;2C.1;PIK3CA;2C.1; | JAK3;2D; | 2C.1 |
| 48 | Breast cancer | female | 53 | TP53 c.818G>A ( p.Arg273His) |  | TP53;2C.2; | 2C.2 |
| 49 | Hepatocellular carcinoma | male | 52 | CTNNB1 c.101G>T p.G34V |  | CTNNB1;2C.2; | 2C.2 |
| 50 | Lung cancer | male | 56 | ret rearrangement | RET fusion;1A.1 |  | 1A.1 |
| 51 | Ovarian cancer | female | 72 | TP53 c.958A>T(p.Lys320Ter) |  | TP53;2C.2; | 2C.2 |
| 52 | Unknown primary | female | 45 | nl |  |  | normal |
| 53 | esophageal cancer | male | 82 | TP53 c.743G>A (p.Arg248Gln) |  | TP53;2C.2; | 2C.2 |
| 54 | Lung cancer | female | 82 | EGFR c.2237_2255del19insT(p.Glu746_Ser752delinsVal), TP53 c.853G>A(p.Glu285Lys), APC c.3920T>A(p.Ile1307Lys) | EGFR;1A.1; | TP53;2C.2;APC; 2C.2 | 1A.1 |
| 55 | Pancreatic cancer | male | 73 | FGFR4 amplification | FGFR4;2C.1; |  | 2C.1 |
| 56 | Breast cancer | female | 73 | PIK3CA c.1633G>A(p.Glu545Lys), TP53 c.817C>T(p.Arg273Cys), RB1 c.1510C>T(p.Gln504Ter) | PIK3CA;1A.1; | TP53;2C.2;RB1;2C.2; | 1A.1 |
| 57 | Sarcoma | male | 71 | nl |  |  | normal |
| 58 | Pancreatic cancer | male | 82 | KRAS c.34G>C(p.Gly12Arg), TP53 c.817C>T(p.Arg273Cys) | KRAS;2C.1; | TP53;2C.2; | 2C.1 |
| 59 | Pancreatic cancer | male | 69 | TP53 c.607G>A(p.Val203Met) |  | TP53;2C.2; | 2C.2 |
| 60 | Vaginal Cancer | male | 45 | nl |  |  | normal |
| 61 | Gastric cancer | male | 36 | TP53 c.844C>T(p.Arg282Trp) |  | TP53;2C.2; | 2C.2 |
| 62 | Cholangiocarcinoma | male | 69 | nl |  |  | normal |
| 63 | Gastric cancer | female | 25 | nl |  |  | normal |
| 64 | Lung cancer | male | 45 | KIF5B(15)-RET(12) | RET fusion;1A.1; |  | 1A.1 |
| 65 | cervical | female | 69 | BAP1 c.598G>T(p.Glu200Ter) | BAP1;2C.1; |  | 2C.1 |
| 66 | Breast cancer | female | 74 | TP53 c.701A>G p.Y234C, CCND1 amplification, FGFR1 amplification, ATM Loss | ATM;2C.1; FGFR1;2C.1 | TP53;2C.2;CCND1;2C.2; | 2C.1 |
| 67 | esophageal cancer | female | 36 | CDKN2A c.71G>C(p.R24P), NFE2L2 c.80A>G(p.D27G) |  | CDKN2A;2D;NFE2L2;2D | 2D |
| 68 | Gastric cancer | female | 69 | KRAS c.35G>A(p.G12D) | KRAS;2C.1; |  | 2C.1 |
| 69 | Ovarian cancer | female | 91 | BRCA2 c.9371A>T p.(Asn3124Ile), BRCA2 LOSS, TP53 c.707A>G(p.Y236C) | BRCA2;1A.1;BRCA2;1A.1; | TP53;2C.2; | 1A.1 |
| 70 | Pancreatic cancer | female | 32 | TP53 c.557A>G(p.D186G) |  | TP53;2C.2; | 2C.2 |
| 71 | sarcoma | female | 40 | nl |  |  | normal |
| 72 | Pancreatic cancer | female | 48 | KRAS c.35G>A(p.Gly12Asp), TP53 c.215_216insG(p.Val73fs) | KRAS;2C.1; | TP53;2C.2; | 2C.1 |
| 73 | Unknown primary | female | 63 | nl |  |  | normal |
| 74 | Breast cancer | female | 56 | TP53 c.256_257delGC(p.Ala86fs) |  | TP53;2C.2; | 2C.2 |
| 75 | Hepatocellular carcinoma CARCINOMA | female | 49 | TP53 c.313G>T(p.Gly105Cys) |  | TP53;2C.2; | 2C.2 |
| 76 | Breast cancer | female | 46 | PALB2 c.2748+1G>C, PALB2 c.2587-1G>C | PALB2;2C.1;PALB2;2C.1; |  | 2C.1 |
| 77 | Lung cancer | male | 47 | KRAS c.34G>T(p.Gly12Cys), TP53 c.637C>T(p.Arg213Ter) | KRAS;1A.2R | TP53;2C.2; | 1A.2R |
| 78 | Unknown primary | male | 42 | AKT1 c.49G>A(p.Glu17Lys) |  | AKT1;2C.2; | 2C.2 |
| 79 | Colorectal cancer | male | 72 | KRAS c.183A>C(p.Gln61His), PIK3 c.1625A>T(p.Glu542Val) & c.3145G>C(p.Gly1049Arg), GNAS c.2531G>A(p.Arg844His) | KRAS;1A.1R;PIK3CA;2C.1;PIK3CA;2C.1; | GNAS;2C.2; | 1A.1R |
| 80 | Pancreatic cancer | male | 76 | KRAS c.35G>A(p.Gly12Asp), TP53 c.430C>T(p.Gln144Ter) | KRAS;2C.1; | TP53;2C.2; | 2C.1 |
| 81 | Colorectal cancer | male | 88 | KRAS c.38G>A(p.G13D), TP53 c.844C>T(p.R282W) | KRAS;1A.1R; | TP53;2C.2; | 1A.1R |
| 82 | Parotid gland tumor | male | 59 | FGFR1(1)-PLAG1(3) | FGFR1 fusion;2C.1; |  | 2C.1 |
| 83 | Ovarian cancer | female | 67 | TP53 c.228_229insG(p.Pro77fs) |  | TP53;2C.2; | 2C.2 |
| 84 | Hepatocellular carcinoma | male | 71 | KNSTRN c.33_34delAG ( p.Arg11fs) |  | KNSTRN;3; | 3 |
| 85 | Pancreatic cancer | female | 90 | KRAS c.35G>A(p.Gly12Asp) | KRAS;2C.1; |  | 2C.1 |
| 86 | Hepatocellular carcinoma CARCINOMA and CHOLANGIO | male | 47 | BAP1 c.639dupT(p.Ile214Tyrfs*29) | BAP1;2C.1; |  | 2C.1 |
| 87 | Malignant Peripheral Nerve Sheath Tumors (MPNST) | female | 56 | nl |  |  | normal |
| 88 | Prostate cancer | male | 73 | PIK3R1 c.1985+3AT>A, PIK3R1 c.1106C>T(p.Thr369Ile) |  | PIK3R1;2C.2;PIK3R1;2C.2; | 2C.2 |
| 89 | Colorectal cancer | female | 40 | ATM c.6040G>T(p.E2014*), PIK3R1 c.1372_1377delGAAAAA(p.E458_K459del), PTPN11 c.205G>A(p.E69K), TP53 c.844C>T(p.R282W) | ATM;2C.1;KRAS/NRAS;1A.1; | PIK3R1;2C.2;PTPN11;2C.2;TP53;2C.2; | 1A.1 |
| 90 | Hepatocellular carcinoma | female | 81 | ARID1A c.3999_4001delGCA(p.Q1334del), TP53 c.527G>A(p.C176Y) |  | ARID1A;2C.2;TP53;2C.2; | 2C.2 |
| 91 | Lung cancer | male | 76 | PIK3CA c.1666C>T(p.His556Tyr), TP53c.493C>T(p.Gln165Ter | PIK3CA;2C.1; |  | 2C.1 |
| 92 | Colorectal cancer | male | 45 | BRAF c.1781A>G(p.D594G), ATM c.5933_5934insCCAC(p.E1978fs*12), ATM c.8584+2T>C, CTNNB1 c.14C>T(p.A5V), CCND2 c.570delC(p.Asp191fs), CDK4 c.541C>T(p.Arg181Ter) | ATM;2C.1;ATM;2C.1;KRAS/NRAS;1A.1; | BRAF;2C.2;CTNNB1;2C.2;CCND2;2C.2;CDK4;2C.2; | 1A.1 |
| 93 | Lung cancer | male | 62 | KRAS c.35G>A(p.Gly12Asp) | KRAS;1A.2R |  | 1A.2R |
| 94 | Pancreatic cancer | female | 63 | KRAS c.35G>T(p.Gly12Val), TP53 c.532C>T(p.His178Tyr), ATM c.9022C>T(p.Arg3008Cys), TSC1 c.433C>T(p.Gln145Ter), CREBBP c.6019C>T(p.Gln2007Ter) | KRAS;2C.1;ATM;2C.1;TSC1;2C.1; | TP53;2C.2;CREBBP;2D; | 2C.1 |
| 95 | Lung cancer | female | 73 | KRAS c.35G>A(p.Gly12Asp), TP53 c.818G>T(p.Arg273Leu) | KRAS;1A.2R | TP53;2C.2; | 1A.2R |
| 96 | Sarcoma | female | 54 | nl |  |  | normal |
| 97 | Cholangiocarcinoma | male | 57 | nl |  |  | normal |
| 98 | Lung cancer | female | 52 | KRAS c.35G>A(p.Gly12Asp) | KRAS;1A.2R |  | 1A.2R |
| 99 | Pancreatic cancer | female | 28 | KRAS c.35G>T(p.Gly12Val) | KRAS;2C.1; |  | 2C.1 |
| 100 | Breast cancer | female | 56 | nl |  |  | normal |
| 101 | Prostate cancer | male | 84 | CTNNB1 c.121A>G (p.Thr41Ala) |  | CTNNB1;2C.2; | 2C.2 |
| 102 | Gastric cancer | female | 66 | RICTOR AMPLIFICATION, TP53 c.919+1G>T |  | TP53;2C.2;RICTOR; 2C.2 | 2C.2 |
| 103 | Pancreatic cancer | male | 36 | KRAS c.35G>T(p.Gly12Val) | KRAS;2C.1; |  | 2C.1 |
| 104 | Mouth Cancer | female | 82 | TP53 c.524G>A(p.R175H), TP53 c.742C>T(p.R248W), SETD2 c.4375C>T(p.R1459*), SETD2 c.1321C>T(p.R441*) , NOTCH3 c.3385C>T(p.Q1129*), CREBBP c.4507T>G(p.Y1503D) |  | TP53;2C.2;TP53;2C.2;SETD2;2D;SETD2;2D;NOTCH3;2D;CREBBP;2D; | 2C.2 |
| 105 | Pancreatic cancer | female | 46 | TSC2 c.2380C>T(p.Q794*), STK11 c.465-1G>A | TSC2;2C.1;STK11;2C.1;STK11;2C.1; |  | 2C.1 |
| 106 | Breast cancer | female | 71 | PIK3CA c.1357G>A(p.E453K), PTEN c.733C>T(p.Q245*), TP53 c.991C>T(p.Q331*) | PIK3CA;1A.1;PTEN;2C.1; | TP53;2C.2; | 1A.1 |
| 107 | Sarcoma | female | 66 | FGFR1 AMPLIFICATION, TP53 c.524G>A(p.R175H), SETD2 c.3048_3066delTTATGCATTAAAGTGTGAC(p.Y1017fs) |  | TP53;2C.2;SETD2;2D;FGFR1; | 2C.1 |
| 108 | Breast cancer | female | 52 | nl |  |  | normal |
| 109 | Ovarian cancer | female | 45 | TP53 c.586C>T(p.Arg196Ter), CCND3 amplification, PIK3CA amplification, KRAS amplification |  | TP53;2C.2;CCND3;2C.2;PIK3CA;2C.2;KRAS;2C.2; | 2C.2 |
| 110 | Sarcoma | female | 49 | nl |  |  | normal |
| 111 | Sarcoma | male | 32 | nl |  |  | normal |
| 112 | Colorectal cancer | male | 48 | PIK3CA c.1634A>C(p.Glu545Ala), PIK3CA c.3129G>T(p.Met1043Ile) | PIK3CA;2C.1;PIK3CA;2C.1;KRAS/NRAS;1A.1; | TP53;2C.2; | 1A.1 |
| 113 | Sarcoma | male | 59 | POLE c.6649C>T(p.Gln2217Ter), POLE c.6622C>T(p.Gln2208Ter), POLE c.3275+3G>A | POLE;2C.1;POLE;2C.1;POLE;2C.1; |  | 2C.1 |
| 114 | Ureteral cancer | female | 33 | nl |  |  | normal |
| 115 | Ovarian cancer | female | 66 | NF1 c.2060_2061insT(p.Glu688fs), TP53 c.375+1G>T | NF1;2C.1; | TP53;2C.2; | 2C.1 |
| 116 | Lung cancer | male | 49 | PIK3CA c.1624G>A(p.Glu542Lys), TP53 c.469G>T(p.Val157Phe), NFE2L2 c.85G>C(p.Asp29His) | PIK3CA;2C.1; | TP53;2C.2;NFE2L2;2D; | 2C.1 |
| 117 | Gastric cancer | male | 56 | TP53 c.733G>A(p.Gly245Ser) |  | TP53;2C.2; | 2C.2 |
| 118 | Unknown primary | female | 59 | PDGFRA c.2525A>T(p.D842V) | PDGFRA;2C.1 |  | 2C.1 |
| 119 | Breast cancer | female | 76 | nl |  |  | normal |
| 120 | Adenocortical | male | 50 | nl |  |  | normal |
| 121 | Prostate cancer | male | 41 | nl |  |  | normal |
| 122 | Lung cancer | male | 26 | KRAS c.34G>T(p.G12C) | KRAS;1A.2; |  | 1A.2R |
| 123 | Gastric cancer | male | 56 | CDKN2A c.131_132insA(p.Tyr44Ter) |  | CDKN2A;2D; | 2D |
| 124 | Esophageal cancer | male | 66 | KRAS c.35G>C(p.Gly12Ala), TP53 c.527G>A(p.Cys176Tyr), ARID1Α c.3999_4001delGCA(p.Gln1327del) | KRAS;2C.1;PD-L1 expression;1;PD-L1 expression;2C.1; | TP53;2C.2;ARID1A;2C.2; | 2C.1 |
| 125 | Ovarian cancer | female | 74 | nl |  |  | normal |
| 126 | Lung cancer | female | 32 | KRAS c.183A>T(p.Gln61His) | KRAS;1A.2;PD-L1 expression;1; |  | 1A.2R |
| 127 | Breast cancer | female | 79 | TP53 c.524G>A(p.Arg175His) |  | TP53;2C.2; | 2C.2 |
| 128 | Ovarian cancer | female | 57 | KRAS c.35G>A(p.Gly12Asp), PIK3CA c.1634A>C(p.Glu545Ala), ARID1Α c.1790_1791insT(p.Pro599fs) | KRAS;2C.1;PIK3CA;2C.1; | ARID1A;2C.2; | 2C.1 |
| 129 | Lung cancer | female | 39 | TP53 c.731delG (p.Gly244fs) |  | TP53;2C.2; | 2C.2 |
| 130 | Cholangiocarcinoma | female | 48 | KRAS c.35G>A(p.Gly12Asp), TP53 c.1024C>T(p.Arg342Ter) | KRAS;2C.1; | TP53;2C.2; | 2C.1 |
| 131 | Brain tumor | male | 61 | EGFR c.866C>T(p.Ala289Val) | EGFR;2C.1; |  | 2C.1 |
| 132 | Hepatocellular carcinoma | male | 65 | nl |  |  | normal |
| 133 | Lung cancer | female | 42 | MRE11A c.341delG(p.G114fs), SETD2 c.6799G>T(p.G2267*), SMARCA4 c.3474_3475insT( p.G1159fs) | MRE11A;2C.1;PD-L1 expression;1; | SETD2;2D;SMARCA4; | 2C.1 |
| 134 | Colorectal cancer | male | 78 | KRAS c.35G>A(p.Gly12Asp), PIK3CA c.3140A>G(p.H1047R), TP53 c.584T>C(p.I195T), RNF43 c.2057_2058insG(p.S687fs) | KRAS;2C.1;KRAS;1A.1;PIK3CA;2C.2;PIK3CA;2C.1; | TP53;2C.2;RNF43;2C.2; | 1A.1R |
| 135 | Ovarian cancer | female | 69 | FOXL2 c.402C>G(p.Cys134Trp) |  | FOXL2;3; | 3 |
| 136 | Penile | female | 42 | CDKN2A c.238C>T(p.R80*), ATR c.5156_5157insCCAC(p.A1720fs), TP53 c.733G>A(p.G245S) | PD-L1 expression;2C.1; | CDKN2A;2D;ATR;2C.2;TP53;2C.2; | 2C.2 |
| 137 | Brain tumor | female | 53 | TP53 c.524G>A(p.Arg175His) |  | TP53;2C.2; | 2C.2 |
| 138 | Ovarian cancer | female | 64 | BRCA2 c.6466_6469delTCTC(Ser2156Asnfs * 11), TP53 c.584T>C(p.Ile195Thr) | BRCA2;1A.1; | TP53;2C.2; | 1A.1 |
| 139 | Pancreatic cancer | male | 79 | KRAS c.35G>A(p.Gly12Asp), RNF43 c.2057_2058insG(p.Ser687fs) | KRAS;2C.1; | RNF43;2C.2; | 2C.1 |
| 140 | Pancreatic cancer | female | 45 | KRAS c.35G>T(p.Gly12Val) | KRAS;2C.1; |  | 2C.1 |
| 141 | Cholangiocarcinoma | female | 61 | KRAS c.181_182insTTCTCGACACAGCAGGTC(p.Gly60_Gln61insLeuLeuAspThrAlaGly),ARID1A c.3223_3242delGAACTTGCAACCAACCTCAA(p.Glu1075Cysfs*23) | KRAS;2C.1;PD-L1 expression;2C.1; | ARID1A;2C.2; | 2C.1 |
| 142 | Pancreatic cancer | female | 53 | KRAS c.35G>A(p.Gly12Asp) | KRAS;2C.1; |  | 2C.1 |
| 143 | Pancreatic cancer | male | 66 | KRAS c.35G>A(p.Gly12Asp), TP53 c.706T>A(p.Tyr236Asn) | KRAS;2C.1; | TP53;2C.2; | 2C.1 |
| 144 | Pancreatic cancer | male | 45 | KRAS c.35G>T(p.Gly12Val) | KRAS;2C.1; |  | 2C.1 |
| 145 | Brain tumor | male | 81 | POLE c.6649C>T(p.Gln2217Ter) | POLE;2C.1; |  | 2C.1 |
| 146 | Serous | male | 62 | TP53 c.747_753delGCCCATC(p.Arg249fs) |  | TP53;2C.2; | 2C.2 |
| 147 | Lacrimal gland cancer | male | 69 | TP53 c.451C>T(p.Pro151Ser) |  | TP53;2C.2; | 2C.2 |
| 148 | Vulvar cancer | female | 72 | CDKN2A c.358G>T(p.Glu120Ter), c.172C>T(p.Arg58Ter), TP53 c.568_569insT(p.Pro190fs) |  | CDKN2A;2D;CDKN2A;2D;TP53;2C.2; | 2C.2 |
| 149 | Gastric cancer | female | 53 | TP53 c.807_837delCTTTGAGGTGCGTGTTTGTGCCTGTCCTGGG(p.Ser269fs) |  | TP53;2C.2; | 2C.2 |
| 150 | Pancreatic cancer | male | 77 | TP53 c.743G>A(p.Arg248Gln) |  | TP53;2C.2; | 2C.2 |
| 151 | Pancreatic cancer | female | 46 | KRAS c.35G>A(p.Gly12Asp) | KRAS;2C.1; |  | 2C.1 |
| 152 | Colorectal cancer | female | 52 | nl |  |  | 1A.1 |
| 153 | Lung cancer | female | 64 | nl |  |  | normal |
| 154 | Pancreatic cancer | male | 35 | KRAS c.35G>A(p.Gly12Asp), TP53 c.823delT(p.Cys275fs) | KRAS;2C.1; | TP53;2C.2; | 2C.1 |
| 155 | Sarcoma | female | 45 | ATM c.2289delT(p.Phe763fs), TP53 c.841G>A(p.Asp281Asn) | ATM;2C.1; | TP53;2C.2; | 2C.1 |
| 156 | Gastric cancer | female | 72 | KRAS Amplification, BRAF c.1781A>C(p.Asp594Ala), MYC Amplification, ARID1A c.6553delA(p.Ile2185fs), TP53 c.524G>A(p.Arg175His) |  | BRAF;2C.2;ARID1A;2C.2;TP53;2C.2;KRAS;MYC; | 2C.2 |
| 157 | Colorectal cancer | female | 56 | KRAS c.38G>A(p.Gly13Asp), PIK3CA c.353G>A(p.Gly118Asp) | KRAS;2C.1;KRAS;1A.1;PIK3CA;2C.2;PIK3CA;2C.1; |  | 1A.1R |
| 158 | Thyroid | male | 34 | TP53 c.364delG(p.Val122Te) |  | TP53;2C.2; | 2C.2 |
| 159 | Sarcoma | female | 56 | nl |  |  | normal |
| 160 | Pancreatic cancer | male | 21 | KRAS c.34_35delGGinsCT(p.G12L), CDK12 c.3283delG(p.E1095fs) | KRAS;2C.1;CDK12;2C.1; |  | 2C.1 |
| 161 | Prostate cancer | male | 66 | TP53 c.818G>A(p.Arg273His) |  | TP53;2C.2; | 2C.2 |
| 162 | Unknown primary | female | 45 | nl |  |  | normal |
| 163 | Pancreatic cancer | male | 55 | TP53 c.517G>A(p.Val173Met) |  | TP53;2C.2; | 2C.2 |
| 164 | Salivary gland | male | 64 | TP53 c.847C>T(p.Arg283Cys) |  | TP53;2C.2; | 2C.2 |
| 165 | Maxillary | male | 68 | WHSC1L1(1)-FGFR1(2) | FGFR1 fusion;2C.1; |  | 2C.1 |
| 166 | Uterine | male | 65 | nl |  |  | normal |
| 167 | Lepidic | male | 32 | KRAS c.35G>C(p.Gly12Ala), ATM c.7976T>G(p.Leu2659Ter) | KRAS;2C.1;ATM;2C.1; |  | 2C.1 |
| 168 | Squamous Cell Carcinoma | female | 45 | nl |  |  | normal |
| 169 | Prostate cancer | male | 68 | TP53 c.1039delG(p.Ala347fs) |  | TP53;2C.2; | 2C.2 |
| 170 | Lung cancer | female | 75 | NF1 c.3493delA(p.I1165*), TP53 c.659A>G(p.Y220C) | NF1;2C.1; | TP53;2C.2; | 2C.1 |
| 171 | Ampullary **cancer** | male | 42 | nl | PD-L1 expression;2C.1; |  | normal |
| 172 | Lung cancer | male | 65 | NOTCH3 c.5502delG(p.Thr1835fs) | PD-L1 expression;1; | NOTCH3;2C.2; | 2C.2 |
| 173 | Lung cancer | female | 47 | TP53 c.398T>C(p.Met133Thr) |  | TP53;2C.2; | 2C.2 |
| 174 | Pancreatic cancer | male | 56 | KRAS c.37G>T(p.Gly13Cys) | KRAS;2C.1; |  | 2C.1 |
| 175 | Lung cancer | male | 64 | KRAS c.35G>Α(p.Gly12Asp), TP53 c.701A>G(p.Tyr234Cys) | KRAS;1A.2; | TP53;2C.2; | 1A.2R |
| 176 | Breast cancer | female | 74 | NOTCH1 c.7398delG p.Ser2467fs), CREBBP c.3118G>T(p.Glu1040Ter), TP53 c.365_366delTG(p.Val122fs) |  | NOTCH1;2C.2;CREBBP;2D;TP53;2C.2; | 2C.2 |
| 177 | Pancreatic cancer | female | 46 | KRAS c.183A>C(p.Gln61His), TP53 c.646G>A(p.Val216Met) | KRAS;2C.1; | TP53;2C.2; | 2C.1 |
| 178 | Prostate cancer | male | 54 | SPOP c.397T>G(p.Phe133Val) |  | SPOP;3; | 3 |
| 179 | sarcoma | male | 85 | nl |  |  | normal |
| 180 | Endometrial cancer | male | 38 | PIK3CA c.3140A>G(p.His1047Arg) | PIK3CA;2C.1; |  | 2C.1 |
| 181 | Brain tumor | male | 61 | IDH1 c.395G>A(p.R132H), NF1 c.412G>T(p.Glu138Ter), ATRX c.3620delA(p.Asp1207fs) | IDH1;2C.1;IDH1;2C.1;NF1;2C.1; | ATRX;2C.2; | 2C.1 |
| 182 | Pancreatic cancer | female | 69 | KRAS c.35G>T(p.Gly12Val), SMARCA4 c.2935C>T(p.Arg979Ter) | KRAS;2C.1; | SMARCA4;2C.2; | 2C.1 |
| 183 | Unknown primary | female | 55 | nl |  |  | normal |
| 184 | Pancreatic cancer | male | 66 | KRAS c.35G>A(p.Gly12Asp), PIK3R1 c.211G>T(p.Gly71Ter) | KRAS;2C.1; | PIK3R1;2C.2; | 2C.1 |
| 185 | Squamous epithelial carcinoma | female | 45 | nl |  |  | normal |
| 186 | Thyroid cancer | male | 42 | nl |  |  | normal |
| 187 | Gastric cancer | female | 79 | nl |  |  | normal |
| 188 | Pancreatic cancer | female | 68 | nl |  |  | normal |
| 189 | Lung cancer | male | 55 | nl |  |  | normal |
| 190 | Lung cancer | male | 56 | KRAS c.35G>T(p.Gly12Val), PIK3CA c.1633G>A(p.Glu545Lys), CTNNB1 c.110C>G(p.Ser37Cys) | KRAS;1A.2;;PIK3CA;2C.1;PD-L1 expression;1; | CTNNB1;2C.2; | 1A.2R |
| 191 | Pancreatic cancer | female | 52 | TP53 c.532C>T(p.His178Tyr) |  | TP53;2C.2; | 2C.2 |
| 192 | Pancreatic cancer | female | 59 | KRAS c.35G>A(p.Gly12Asp), CDKN2A c.221delA(p.Leu74fs), TP53 c.659A>G(p.Tyr220Cys) | KRAS;2C.1;PD-L1 expression;2C.1; | CDKN2A;2D;TP53;2C.2; | 2C.1 |
| 193 | Pancreatic cancer | male | 64 | KRAS c.35G>T(p.Gly12Val), CDKN2A c.233_234delTC(p.Leu78fs), RNF43 c.2057_2058insG(p.Ser687fs) | KRAS;2C.1; | CDKN2A;2D;RNF43;2C.2; | 2C.1 |
| 194 | Cholangiocarcinoma | male | 72 | ERBB3 c.889G> (p.Asp297Tyr) |  | ERBB3;2C.2; | 2C.2 |
| 195 | Cholangiocarcinoma | female | 39 | IDH1 c.394C>T(p.Arg132Cys) | IDH1;2C.1;IDH1;2C.1; |  | 2C.1 |
| 196 | Lung cancer | male | 57 | IDH1 c.394C>T(p.Arg132Cys), PIK3CA c.1624G>A(p.Glu542Lys) | IDH1;2C.1;PIK3CA;2C.1;PD-L1 expression;1; | SETD2;2D; | 2C.1 |
| 197 | Cholangiocarcinoma | male | 69 | nl |  |  | normal |
| 198 | Pancreatic cancer | female | 55 | KRAS c.34G>C(p.Gly12Arg), TP53 c.332T>A (p.Leu111Gln) | KRAS;2C.1; | TP53;2C.2; | 2C.1 |
| 199 | Lung cancer | male | 82 | KRAS c.34G>C(p.Gly12Arg), STK11 c.680delG(p.Gly227fs) | KRAS;1A.2;;STK11;2C.1;STK11;2C.1; |  | 1A.2R |
| 200 | Gastric cancer | female | 67 | PIK3CA c.1634A>G(p.Glu545Gly) | PIK3CA;2C.1;Microsatellite Instability (MSI);1A.1;Microsatellite Instability (MSI);2C.1;PD-L1 expression;1;PD-L1 expression;2C.1; |  | 2C.1 |
| 201 | Cholangiocarcinoma | female | 46 | NRAS c.182A>G(p.Gln61Arg) |  | NRAS;2D; | 2D |
| 202 | Breast cancer | female | 53 | PIK3CA c.3140A>G(p.His1047Arg) | PIK3CA;1A.1;PIK3CA;2C.1; |  | 1A.1 |
| 203 | Lung cancer | male | 69 | EGFR exon 19:c.2236_2250delGAATTAAGAGAAGCA(p.Glu746_Ala750del), EGFR exon 20: c.2369C>T(p.T790M) | EGFR;1A.1; |  | 1A.1 |
| 204 | Pancreatic cancer | male | 81 | KRAS c.35G>T(p.Gly12Val), POLE c.6649C>T(p.Gln2217Ter) | KRAS;2C.1;POLE;2C.1; |  | 2C.1 |
| 205 | Adrenal gland | male | 52 | nl |  |  | normal |
| 206 | Lung cancer | male | 58 | nl | PD-L1 expression;1; |  | normal |
| 207 | GIST | female | 64 | KIT exon 11 c.1669_1674delTGGAAG(p.Trp557_Lys558del) | KIT;1A.1; |  | 1A.1 |
| 208 | Cholangiocarcinoma | male | 58 | WHSC1L1(1)-FGFR1(2) | FGFR1 fusion;2C.1; |  | 2C.1 |
| 209 | Ovarian cancer | female | 66 | TP53 c.997delC(p.Arg333fs), EIF3E(1)-RSPO2(2) | Microsatellite Instability (MSI);1A.1;Microsatellite Instability (MSI);2C.1; | TP53;2C.2;RSPO2 fusion;PD-L1 expression; | 2C.2 |
| 210 | kidney | male | 48 | MLH1 c.1609C>T(p.Gln537Ter) | PD-L1 expression;2C.1; | TP53;2C.2; | 2C.2 |
| 211 | Lung cancer | female | 44 | PIK3CA c.1624G>A(p.Glu542Lys), TP53 c.817C>T(p.R273C) | PIK3CA;2C.1;PD-L1 expression;1; | TP53;2C.2;SETD2;2D; | 2C.1 |
| 212 | Pancreatic cancer | female | 55 | KRAS c.35G>A(p.Gly12Asp), TP53 c.646G>A(p.Val216Met) | KRAS;2C.1; | TP53;2C.2; | 2C.1 |
| 213 | Pancreatic cancer | male | 65 | PIK3CA c.3140A>G(p.H1047R), MSH2 deletion of exons 9-10 | PIK3CA;2C.1;MSH2;1B;Microsatellite Instability (MSI);1A.1;Microsatellite Instability (MSI);2C.1;PD-L1 expression;2C.1; |  | 1B |
| 214 | Melanoma | male | 52 | CDKN2A c.88delG (p.Ala30fs), NOTCH1 c.1705G>T(p.Glu569Ter) |  | CDKN2A;2D;NOTCH1;2C.2; | 2C.2 |
| 215 | Breast cancer | female | 79 | nl |  |  | normal |
| 216 | Pancreatic cancer | male | 82 | TP53 c.211_220delCCCCCCGTGG(p.Pro72fs) |  | TP53;2C.2; | 2C.2 |
| 217 | Gastric cancer | male | 63 | nl |  |  | normal |
| 218 | Breast cancer | female | 59 | PIK3CA c.3140A>G(p.His1047Arg), POLE c.6649C>T(p.Gln2217Ter),TP53 c.1363C>T(p.Cys238Phe), RB1 c.1363C>T( p.Arg455Ter) | PIK3CA;1A.1;PIK3CA;2C.1;POLE;2C.1; | TP53;2C.2;RB1;2C.2; | 1A.1 |
| 219 | Pancreatic cancer | female | 66 | nl |  |  | normal |
| 220 | Melanoma | female | 56 | nl |  |  | normal |
| 221 | Lung cancer | male | 62 | KRAS c.34G>T(p.Gly12Cys), ATM c.3832delG(p.Asp1278fs) | KRAS;1A.2;;ATM;2C.1;PD-L1 expression;1; |  | 1A.2R |
| 222 | Prostate cancer | male | 83 | nl |  |  | normal |
| 223 | Appendix cancer | female | 55 | KRAS c.38G>A(p.Gly13Asp), TP53 c.1024C>T(p.Arg342Ter) | KRAS;2C.1; | TP53;2C.2; | 2C.1 |
| 224 | Pancreatic cancer | male | 65 | KRAS c.35G>T(p.Gly12Val) | KRAS;2C.1; |  | 2C.1 |
| 225 | Pancreatic cancer | male | 71 | PALB2 c.1037_1041delAAGAA(p.Lys346Thrfs) | PALB2;2C.1; |  | 2C.1 |
| 226 | Lung cancer | male | 64 | BRAF c.1799T>A(p.Val600Glu), TP53 c.991C>T(p.Gln331Ter), SETD2 c.1665_1672delTAAATCTA(p.Lys556fs) | PD-L1 expression;1; | BRAF;1A.1;TP53;2C.2;SETD2;2D; | 1A.1 |
| 227 | Desmoplastic small-round-cell **tumor** | male | 55 | nl |  |  | normal |
| 228 | Pancreatic cancer | male | 56 | KRAS c.35G>A(p.Gly12Asp) | KRAS;2C.1;PD-L1 expression;2C.1; |  | 2C.1 |
| 229 | Colorectal cancer | male | 58 | TP53 c.641A>G(p.His214Arg) |  | TP53;2C.2; | 1A.1 |
| 230 | Medullary Thyriod | female | 44 | RET c.2753T>C(p.Met918Thr), RET c.2410G>A(p.Val804Met) | RET;1A.1;RET;1A.1; |  | 1A.1 |
| 231 | Sarcoma | male | 56 | nl |  |  | normal |
| 232 | Colorectal cancer | male | 67 | TP53 c.586C>T(p.Arg196Ter) | KRAS/NRAS;1A.1;PD-L1 expression;2C.1; | TP53;2C.2; | 1A.1 |
| 233 | Prostate cancer | male | 46 | nl |  |  | normal |
| 234 | Breast cancer | female | 55 | PIK3CA c.1633G>A(p.Glu545Lys) | PIK3CA;1A.1;PIK3CA;2C.1; |  | 1A.1 |
| 235 | Cholangiocarcinoma | male | 46 | nl |  |  | normal |
| 236 | Lung cancer | male | 44 | EML4(13)-ALK(20) | ALK fusion;1.A.1;PD-L1 expression;1; |  | 1A.1 |
| 237 | Pancreatic cancer | female | 52 | KRAS c.35G>A(p.Gly12Asp), TP53 c.818G>A(p.Arg273His) | KRAS;2C.1; | TP53;2C.2; | 2C.1 |
| 238 | Colorectal cancer | male | 69 | nl | KRAS/NRAS;1A.1; |  | 1A.1 |
| 239 | Sarcoma | male | 55 | NTRK2 Amplification |  | NTRK2;3; | 2C.2 |
| 240 | Colorectal cancer | male | 45 | KRAS c.38G>A(p.Gly13Asp), TP53 c.469G>T(p.Val157Phe) | KRAS;2C.1;KRAS;1A.1; | TP53;2C.2; | 1A.1R |
| 241 | Pancreatic cancer | female | 81 | KRAS c.35G>A(p.Gly12Asp) | KRAS;2C.1; |  | 2C.1 |
| 242 | Adenocarcinoma | male | 56 | TP53 c.842G>T (p.Cys275Phe) |  | TP53;2C.2; | 2C.2 |
| 243 | Brain tumor | male | 55 | nl |  |  | normal |
| 244 | Pancreatic cancer | male | 69 | nl |  |  | normal |
| 245 | Melanoma | male | 58 | GNA11 c.626A>T(p.Gln209Leu), BAP1 c.587G>A(p.Trp196Ter) | BAP1;2C.1; | GNA11;2C.2; | 2C.1 |
| 246 | Prostate cancer | male | 54 | nl |  |  | normal |
| 247 | Gastric cancer | female | 66 | RET c.2410G>A(p.Val804Met), TP53 c.733G>A(p.Gly245Ser) | RET;2C.1;PD-L1 expression;1;PD-L1 expression;2C.1; | TP53;2C.2; | 2C.1 |
| 248 | Breast cancer | female | 43 | PIK3CA c.1035T>A(p.Asn345Lys) | PIK3CA;1A.1;PIK3CA;2C.1; |  | 1A.1 |
| 249 | Adrenocortical Carcinoma | male | 76 | NF1 c.3810_3820delCATGCAGACTC(p.Met1271fs) | NF1;2C.1; |  | 2C.1 |
| 250 | Ovarian cancer | female | 80 | NRAS c.182A>G(p.Gln61Arg) |  | NRAS;2D; | 2D |
| 251 | kidney cancer | male | 67 | MTOR c.4379T>C(p.Leu1460Pro) | MTOR;2C.1; |  | 2C.1 |
| 252 | Endometrial cancer | male | 71 | TP53 c.712_725delTGTAACAGTTCCTG(p.Cys238fs) |  | TP53;2C.2; | 2C.2 |
| 253 | Cholangiocarcinoma | female | 43 | CTNNB1 c.121A>G(p.Thr41Ala), TP53 c.796G>A(p.Gly266Arg) |  | CTNNB1;2C.2;TP53;2C.2; | 2C.2 |
| 254 | Lung cancer | male | 44 | TP53 c.405_405delCinsTGTTTTGA(p.Gln136fs), TSC1 c.2644_2645insA(p.Ala882fs) | TSC1;2C.1; | TP53;2C.2; | 2C.1 |
| 255 | Lung cancer | male | 41 | BRAF c.1405_1406delGGinsTC(p.Gly469Ser), TP53 c.743G>A(p.Arg248Gln) |  | BRAF;2C.2;TP53;2C.2; | 2C.2 |
| 256 | Brain tumor | male | 48 | EGFR c.866C>T(p.Ala289Val) | EGFR;2C.1;PD-L1 expression;2C.1; |  | 2C.1 |
| 257 | Lung cancer | female | 74 | nl |  |  | normal |
| 258 | Lung cancer | male | 82 | nl | PD-L1 expression;1; |  | normal |
| 259 | Lung cancer | female | 84 | TP53 c.660T>G(p.Tyr220Ter), RB1 c.2484delA(p.Arg828fs) |  | TP53;2C.2;RB1;2C.2; | 2C.2 |
| 260 | Sarcoma | male | 63 | nl |  |  | normal |
| 261 | Ovarian cancer | female | 73 | nl |  |  | normal |
| 262 | melanoma | male | 70 | BRAF c.1397G>A(p.Gly466Glu) |  | BRAF;2C.2; | 2C.2 |
| 263 | Colorectal cancer | female | 56 | KRAS c.34G>T(p.Gly12Cys) | KRAS;2C.1;KRAS;1A.1R; |  | 1A.1R |
| 264 | Breast cancer | female | 72 | nl |  |  | normal |
| 265 | Gastric cancer | female | 61 | PIK3CA c.1633G>A(p.Glu545Lys) | PIK3CA;2C.1; |  | 2C.1 |
| 266 | Breast cancer | female | 54 | PIK3CA c.3140A>G(p.His1047Arg), TP53 c.949C>T(p.Gln317Ter) | PIK3CA;1A.1;PIK3CA;2C.1; | TP53;2C.2; | 1A.1 |
| 267 | Cholangiocarcinoma | female | 43 | nl |  |  | normal |
| 268 | Endometrial cancer | male | 69 | KRAS c.35G>C(p.Gly12Ala), KRAS c.182A>T(p.Gln61Leu) | KRAS;2C.1;KRAS;2C.1; |  | 2C.1 |
| 269 | Colorectal cancer | female | 41 | PMS2 c.151delG(p.Ala51Profs), PIK3CA c.3062A>G(p.Tyr1021Cys) | PMS2;1B;PIK3CA;2C.2;PIK3CA;2C.1;KRAS/NRAS;1A.1 |  | 1A.1 |
| 270 | Breast cancer | female | 46 | nl |  |  | normal |
| 271 | Cholangiocarcinoma | female | 59 | IDH1 c.394C>T(p.R132C) | IDH1;2C.1; |  | 2C.1 |
| 272 | Breast cancer | female | 62 | nl |  |  | normal |
| 273 | sarcoma | female | 68 | nl |  |  | normal |
| 274 | Brain tumor | male | 53 | CDKN2A c.382_383insC(p.Arg128fs), NF1 c.1336delA(p.Thr446fs), TP53 c.647_648delTG(p.Val216fs) | NF1;2C.1; | CDKN2A;2D;TP53;2C.2; | 2C.1 |
| 275 | Schwanomma | female | 51 | nl |  |  | normal |
| 276 | Pancreatic cancer | male | 50 | KRAS c.34G>C(p.Gly12Arg) | KRAS;2C.1; |  | 2C.1 |
| 277 | Pancreatic cancer | male | 54 | KRAS c.35G>T(p.Gly12Val) | KRAS;2C.1; |  | 2C.1 |
| 278 | Head and Neck | female | 70 | PIK3CA c.3140A>G(p.His1047Arg), TP53 c.309C>A( p.Tyr103Ter) | PIK3CA;2C.1; | TP53;2C.2; | 2C.1 |
| 279 | Colorectal cancer | male | 43 | TP53 c.841G>A(p.Asp281Asn), TP53 c.855G>C(p.Glu285Asp) | KRAS/NRAS;1A.1; | TP53;2C.2;TP53;2C.2; | 1A.1 |
| 280 | Hepatocellular carcinoma | female | 82 | GNAQ c.626A>C(p.Gln209Pro), SF3B1 c.1873C>T(p.Arg625Cys) |  | GNAQ;2C.2;SF3B1;3; | 2C.2 |
| 281 | Colorectal cancer | male | 77 | nl | KRAS/NRAS;1A.1; |  | 1A.1 |
| 282 | Pancreatic cancer | male | 69 | KRAS c.34G>C(p.Gly12Arg), TP53 c.916C>T(p.Arg306Ter) | KRAS;2C.1; | TP53;2C.2; | 2C.1 |
| 283 | Endometrial cancer | male | 73 | PIK3CA c.3140A>G(p.His1047Arg), CHEK2 c.1100delC(p.Thr367fs), NF2 c.540_542delTCCinsCT(p.Pro181fs), KRAS c.38G>A(p.Gly13Asp) | PIK3CA;2C.1;CHEK2;2C.1;KRAS;2C.1; | NF2;3; | 2C.1 |
| 284 | Urothelial | male | 71 | FGFR Amplification | FGFR;2C.1; |  | 2C.1 |
| 285 | Breast cancer | female | 82 | ERBB2 (HER2) Amplification | ERBB2;1A.1; |  | 1A.1 |
| 286 | Breast cancer | female | 68 | PTCH1 c.3944_3945insT(p.Tyr1316fs) | PTCH1;2C.1; |  | 2C.1 |
| 287 | esophageal cancer | male | 44 | RNF43 c.2057_2058insG (p.S687fs), TP53 c.586C>T(p.Arg196Ter), MYC amplification |  | RNF43;2C.2;TP53;2C.2;MYC;2C.2; | 2C.2 |
| 288 | Pancreatic cancer | female | 35 | KRAS c.34G>C(p.Gly12Arg) | KRAS;2C.1; |  | 2C.1 |
| 289 | Ovarian cancer | female | 68 | KRAS c.35G>A (p.Gly12Asp) | KRAS;2C.1; |  | 2C.1 |
| 290 | Pancreatic cancer | male | 24 | KRAS c.38G>A(p.Gly13Asp), TP53 c.524G>A(p.Arg175His) | KRAS;2C.1; | TP53;2C.2; | 2C.1 |
| 291 | Ovarian cancer | female | 44 | MYC Amplification |  | MYC;2C.2; | 2C.2 |
| 292 | Pancreatic cancer | male | 68 | KRAS c.34G>C(p.Gly12Arg) | KRAS;2C.1; |  | 2C.1 |
| 293 | Lung cancer | female | 73 | KRAS c.35G>T(p.Gly12Val), TP53 c.524G>A(p.Arg175His) | KRAS;1A.2; | TP53;2C.2; | 1A.2R |
| 294 | Pancreatic cancer | female | 35 | WHSC1L1(1)-FGFR1(2) | FGFR1 fusion;2C.1; |  | 2C.1 |
| 295 | Gastric cancer | female | 68 | nl |  |  | normal |
| 296 | Prostate cancer | male | 88 | TMPRSS2-ERG |  | ERG fusion; | 2C.2 |
| 297 | Gastric cancer | male | 29 | TP53 c.518T>C(p.Val173Ala) |  | TP53;2C.2; | 2C.2 |
| 298 | sarcoma | male | 37 | nl |  |  | normal |
| 299 | Prostate cancer | male | 45 | RAD50 c.7_8insC(p.Arg3fs), TP53 c.395A>T(p.Lys132Met) | RAD50;2A.1; | TP53;2C.2; | 1A.1 |
| 300 | Breast cancer | female | 60 | PIK3CA c.1035T>A(p.Asn345Lys), MIR143HG(1)-NOTCH1(27) | PIK3CA;1A.1;PIK3CA;2C.1;Microsatellite Instability (MSI);1A.1;Microsatellite Instability (MSI);2C.1;PD-L1 expression;1;PD-L1 expression;2C.1; | NOTCH1 fusion;2C.2; | 1A.1 |
| 301 | Breast cancer | female | 53 | ESR1 c.1613A>G(p.Asp538Gly), CHEK2 c.1100delC(p.Thr367fs), TP53 c.291_292insTTCTGTC(p.Pro98fs) | CHEK2;2C.1;Microsatellite Instability (MSI);1A.1;Microsatellite Instability (MSI);2C.1; | ESR1;2C.2;TP53;2C.2;PD-L1 expression; | 2C.1 |
| 302 | Melanoma | male | 46 | BRAF c.1801A>G(p.Lys601Glu) |  | BRAF;2C.2; | 2C.2 |
| 303 | Pancreatic cancer | female | 43 | KRAS c.35G>T(p.Gly12Val), SLX4 c.212C>G(p.Ser71Ter), TP53 c.743G>A(p.Arg248Gln),TSEN2(5)-PPARG(4) | KRAS;2C.1; | SLX4;TP53;2C.2;PPARG fusion; | 2C.1 |
| 304 | Breast cancer | female | 44 | PIK3CA c.1624G>A(p.Glu542Lys), PIK3CA c.2176G>A(p.Glu726Lys) | PIK3CA;1A.1;PIK3CA;2C.1;PIK3CA;1A.1;PIK3CA;2C.1; |  | 1A.1 |
| 305 | Pancreatic cancer | male | 39 | KRAS c.35G>A(p.Gly12Asp) | KRAS;2C.1; |  | 2C.1 |
| 306 | Colorectal cancer | female | 69 | TP53 c.833C>T(p.Pro278Leu) | KRAS/NRAS;1A.1; | TP53;2C.2; | 1A.1 |
| 307 | Pancreatic cancer | male | 73 | KRAS c.35G>A(p.Gly12Asp), TP53 c.725G>T(p.Cys242Phe), EIF3E(1)-RSPO2(2) | KRAS;2C.1;PD-L1 expression;2C.1; | TP53;2C.2;RSPO2 fusion; | 2C.1 |
| 308 | Gastric cancer | male | 85 | nl |  |  | normal |
| 309 | Ampulla of Vater | male | 56 | KRAS c.35G>A(p.Gly12Asp), RNF43 c.1177_1178insG(p.Ala393fs), TP53 c.536A>G(p.His179Arg) | KRAS;2C.1; | RNF43;2C.2;TP53;2C.2; | 2C.1 |
| 310 | Ovarian cancer | female | 64 | TP53 c.659A>G(p.Tyr220Cys) |  | TP53;2C.2; | 2C.2 |
| 311 | Endometrial cancer | female | 68 | TP53 c.843C>G(p.Asp281Glu) |  | TP53;2C.2; | 2C.2 |
| 312 | Pancreatic cancer | female | 87 | KRAS c.35G>A(p.Gly12Asp), CDKN2A c.172C>T(p.Arg58Ter), TP53 c.818G>A(p.Arg273His) | KRAS;2C.1; | CDKN2A;2D;TP53;2C.2; | 2C.1 |
| 313 | Sarcoma | male | 44 | PIK3CA c.3140A>G(p.His1047Arg), TP53 c.151G>T(p.Glu51Ter) | PIK3CA;2C.1; | TP53;2C.2; | 2C.1 |
| 314 | Brain tumor | male | 53 | EGFR vIII (deletion of exons 2–7) | EGFR;2C.1; |  | 2C.1 |
| 315 | Lung cancer | male | 70 | BRAF c.1799T>A(p.V600E) | PD-L1 expression;1; | BRAF;1A.1; | 1A.1 |
| 316 | Breast cancer | female | 37 | BRCA2 c.1389delA(p.Val464Trpfs*3) | BRCA2;1A.1; |  | 1A.1 |
| 317 | Brain tumor | male | 78 | nl |  |  | normal |
| 318 | Bladder cancer | male | 73 | TERT c.-124C>T(C228T), TP53 c.839G>C(p.Arg280Thr) |  | TERT;2D;TP53;2C.2; | 2C.2 |
| 319 | Pancreatic cancer | male | 42 | FANCA c.3094C>T(p.Gln1032Ter), KRAS c.35G>A(p.Gly12Asp), STK11 c.923G>A(p.Trp308Ter), TP53 c.310C>T(p.Gln104Ter) | FANCA;2C.1;KRAS;2C.1;STK11;2C.1;STK11;2C.1; | TP53;2C.2; | 2C.1 |
| 320 | Sarcoma | female | 59 | NF1 c.1246C>T(p.Arg416Ter) | NF1;2C.1;Microsatellite Instability (MSI);1A.1;Microsatellite Instability (MSI);2C.1; | PD-L1 expression; | 2C.1 |
| 321 | Lung cancer | male | 60 | nl |  |  | normal |
| 322 | kidney cancer | female | 70 | PIK3CA c.1633G>A(p.Glu545Lys), PIK3CA c.2176G>A(p.Glu726Lys) | PIK3CA;2C.1;PIK3CA;2C.1;PD-L1 expression;2C.1; |  | 2C.1 |
| 323 | Ovarian cancer | female | 51 | BRCA1 c.3628G>T(p.Glu1210Ter), TP53 c.578A>G(p.His193Arg) | BRCA1;1A.1; | TP53;2C.2; | 1A.1 |
| 324 | Gastric cancer | male | 54 | nl |  |  | normal |
| 325 | Endometrial cancer | male | 49 | nl |  |  | normal |
| 326 | Endometrial cancer | male | 43 | KRAS c.34G>A(p.Gly12Ser), FBXW7 c.1513C>T(p.Arg505Cys), TP53c.743G>A(p.Arg248Gln) | KRAS;2C.1; | FBXW7;2C.2;FBXW7;2C.2; | 2C.1 |
| 327 | Breast cancer | female | 53 | ΑΚΤ1 c.49G>A(p.Glu17Lys), TP53 c.715A>G(p.Asn239Asp) |  | AKT1;2C.2;TP53;2C.2; | 2C.2 |
| 328 | Ovarian cancer | female | 81 | NF1 c.2087G>A(p.Trp696Ter) | NF1;2C.1;PD-L1 expression;2C.1; |  | 2C.1 |
| 329 | kidney | male | 63 | TP53 c.747G>T(p.Arg249Ser) |  | TP53;2C.2; | 2C.2 |
| 330 | Melanoma | female | 42 | NTRK2 Amplification, MYC Amplification, CCND3 Amplification |  | NTRK2;3;MYC;2C.2;CCND3; | 2C.2 |
| 331 | Pancreatic cancer | female | 79 | KRAS c.35G>T(p.Gly12Val), PIK3CA c.3139C>T(p.His1047Tyr) | KRAS;2C.1;PIK3CA;2C.1; |  | 2C.1 |
| 332 | Colorectal cancer | female | 43 | KRAS c.35G>C(p.Gly12Ala), TP53 c.844C>T(p.Arg282Trp) | KRAS;2C.1;KRAS;1A.1; | TP53;2C.2; | 1A.1R |
| 333 | Urothelial carcinoma | male | 68 | PIK3CA c.1030G>A(p.Val344Met) | PIK3CA;2C.1;PD-L1 expression;1;PD-L1 expression;2C.1;PD-L1 expression;1;PD-L1 expression;2C.1; |  | 2C.1 |
| 334 | Breast cancer | female | 63 | BRCA1 c.5266dupC(p.Gln1756Profs*74) | BRCA1;1A.1; |  | 1A.1 |
| 335 | Prostate cancer | male | 49 | TMPRSS2-ERG | Microsatellite Instability (MSI);1A.1;Microsatellite Instability (MSI);2C.1; | ERG fusion;2C.2;PD-L1 expression; | 2C.2 |
| 336 | Endometrial cancer | male | 81 | TP53 c.843C>G(p.Asp281Glu) |  | TP53;2C.2; | 2C.2 |
| 337 | Breast cancer | female | 56 | PIK3R1 c.1692C>G(p.Asn564Lys), TP53 c.586C>T(p.Arg196Ter) |  | PIK3R1;2C.2;TP53;2C.2; | 2C.2 |
| 338 | Pancreatic cancer | male | 60 | PALB2 c.1140_1143delTCTT(p.Ser380fs) | PALB2;2C.1; |  | 2C.1 |
| 339 | Colorectal cancer | female | 68 | TP53 c.743G>A(p.Arg248Gln) | KRAS/NRAS;1A.1; | TP53;2C.2; | 1A.1 |
| 340 | Prostate cancer | male | 83 | nl |  |  | normal |
| 341 | Adenoid cystic carcinoma | male | 44 | NOTCH1 c.7470C>A(p.Tyr2490Ter) |  | NOTCH1;2C.2; | 2C.2 |
| 342 | Lung cancer | male | 49 | SMARCA4 c.4402G>T(p.Glu1468Ter), TP53 c.713G>A(p.Cys238Tyr) |  | SMARCA4;2C.2;TP53;2C.2; | 2C.2 |
| 343 | Breast cancer | female | 70 | nl |  |  | normal |
| 344 | Unknown primary | male | 39 | TP53 c.592G>T(p.Glu198Ter), ARID1A c.1185T>G(p.Tyr395Ter) |  | TP53;2C.2;ARID1A;2C.2; | 2C.2 |
| 345 | Breast cancer | female | 78 | nl |  |  | normal |
| 346 | Prostate cancer | male | 69 | PIK3CA c.1633G>A(p.Glu545Lys), RB1 c.1654C>T(p.Arg552Ter) | PIK3CA;2C.1; | RB1;2C.2; | 2C.1 |
| 347 | Prostate cancer | male | 52 | nl |  |  | normal |
| 348 | Colorectal cancer | female | 51 | BRAF: c.1799T>A p.V600E) | BRAF;1A.2;KRAS/NRAS;1A.1; |  | 1A.2 |
| 349 | Prostate cancer | male | 59 | nl |  |  | normal |
| 350 | Pancreatic cancer | male | 61 | TP53 c.916C>T(p.Arg306Ter) |  | TP53;2C.2; | 2C.2 |
| 351 | Pancreatic cancer | male | 75 | KRAS c.35G>T(p.Gly12Val) | KRAS;2C.1; |  | 2C.1 |
| 352 | Ovarian cancer | female | 72 | BRCA1 c.3785C>A(p.Ser1262Ter) | BRCA1;1A.1;PD-L1 expression;2C.1; |  | 1A.1 |
| 353 | Pancreatic cancer | male | 48 | KRAS c.35G>A(p.Gly12Asp), CDKN2A c.130_131insC(p.Tyr44fs) | KRAS;2C.1; | CDKN2A;2D; | 2C.1 |
| 354 | Gastric cancer | male | 52 | ATR c.823del(p.Glu275fs) |  | ATR;2C.2; | 2C.2 |
| 355 | Malignant Mesenchymal tumor | female | 66 | nl | PD-L1 expression;2C.1; |  | normal |
| 356 | esophageal cancer | female | 64 | nl | PD-L1 expression;1;PD-L1 expression;2C.1; |  | normal |
| 357 | sarcoma | female | 69 | nl | PD-L1 expression;2C.1; |  | normal |
| 358 | Pancreatic cancer | female | 52 | KRAS c.35G>A(p.Gly12Asp), TP53 c.731G>T(p.Gly244Val) | KRAS;2C.1;PD-L1 expression;2C.1; | TP53;2C.2; | 2C.1 |
| 359 | kidney cancer | female | 48 | MYC Amplification | PD-L1 expression;2C.1; | MYC;2C.2; | 2C.2 |
| 360 | Brain tumor | male | 44 | IDH1 c.394C>G(p.Arg132Gly), NF1 c.5503C>T(p.Gln1835Ter),BRAF c.1391G>A(p.Gly464Glu), TP53 c.745A>G(p.Arg249Gly), TP53 c.817C>T(p.Arg273Cys), ATRX1 c.2669delC(p.Ser890Ter), PIK3CAc.328_330delGAA(p.Glu109del) | IDH1;2C.1;NF1;2C.1; | BRAF;2C.2;TP53;2C.2;TP53;2C.2;ATRX1;ATRX1; | 2C.1 |
| 361 | Lung cancer | male | 66 | FGFR1 c.2059A>G(p.Lys687Glu), NOTCH1 c.4129_4130insT(p.Pro1377fs) | FGFR1;2C.1; | NOTCH1;2C.2; | 2C.1 |
| 362 | Breast cancer | female | 64 | ESR1 c.1613A>G(p.Asp538Gly), AKT1 c.49G>A(p.Glu17Lys) |  | ESR1;2C.2;AKT1;2C.2; | 2C.2 |
| 363 | Head and Neck cancer | female | 53 | TP53 c.743G>A(p.Arg248Gln) |  | TP53;2C.2; | 2C.2 |
| 364 | Pancreatic cancer | male | 71 | KRAS c.35G>T(p.Gly12Val), CDKN2A c.7G>T(p.Glu27Ter), TP53 c.646G(p.Val216Leu) | KRAS;2C.1; | CDKN2A;2D;TP53;2C.2; | 2C.1 |
| 365 | Colorectal cancer | male | 78 | KRAS c.35G>A(p.Gly12Asp), PIK3CA c.3140A>G(p.His1047Arg), PTPRK(1)-RSPO3(2) | KRAS;2C.1;KRAS;1A.1;PIK3CA;2C.2;PIK3CA;2C.1; | RSPO3 fusion;2C.2; | 1A.1R |
| 366 | sarcoma | female | 55 | nl |  |  | normal |
| 367 | Lymph Nodes | female | 46 | TP53 c.706T>C(p.Tyr236His) |  | TP53;2C.2; | 2C.2 |
| 368 | Pancreatic cancer | female | 55 | nl |  |  | normal |
| 369 | sarcoma | male | 21 | nl |  |  | normal |
| 370 | Pancreatic cancer | male | 58 | KRAS c.35G>A(p.Gly12Asp), SMAD4 c.1576G>T(p.Glu526Ter), TP53 c.733G>A(p.Gly245Ser) | KRAS;2C.1; | SMAD4;2C.2;TP53;2C.2; | 2C.1 |
| 371 | Cholangiocarcinoma | male | 41 | KRAS c.35G>A(p.Gly12Asp) | KRAS;2C.1; |  | 2C.1 |
| 372 | Lung cancer | female | 69 | BRAF c.1799T>A(p.Val600Glu), STK11 c.109C>T(p.Gln37Ter) | STK11;2C.1;STK11;2C.1; | BRAF;1A.1; | 1A.1 |
| 373 | Brain tumor | female | 68 | FGFR1 Amplification, AXL Amplification, CCNE1 Amplification | FGFR12C.1; | AXL;2C.2;CCNE1;2C.2; | 2C.1 |
| 374 | Pancreatic cancer | female | 59 | KRAS c.35G>A(p.Gly12Asp), TP53 c.524G>A(p.Arg175His) | KRAS;2C.1;PD-L1 expression;2C.1; | TP53;2C.2; | 2C.1 |
| 375 | Endometrial cancer | male | 68 | PIK3CA c.3129G<T(p.Met1043Ile), TP53 c.818G>A(p.Arg273His) | PIK3CA;2C.1; | TP53;2C.2; | 2C.1 |
| 376 | Gastric cancer | male | 52 | TP53 c.524G>A(p.Arg175His) |  | TP53;2C.2; | 2C.2 |
| 377 | Pancreatic cancer | male | 44 | MLH1 c.1292_1293insC(p.Met431fs), FBXW7 c.1629_1630delAG(p.Arg543fs), FBXW7 c.1099C>T(p.Arg367Ter), CDKN2B c.244C>T(p.Arg82Ter) | MLH1;1B; | FBXW7;2C.2;FBXW7;2C.2;CDKN2B;2D; | 1B |
| 378 | Lung cancer | male | 83 | KRAS c.35G>C(p.Gly12Ala) | KRAS;1A.2; |  | 1A.2R |
| 379 | Lung cancer | male | 81 | NF1 c.3049C>T(p.Gln1017Ter | NF1;2C.1; |  | 2C.1 |
| 380 | Head and Neck | male | 55 | nl | PD-L1 expression;1;PD-L1 expression;2C.1; |  | normal |
| 381 | Prostate cancer | male | 59 | PIK3R1 c.1042C>T(p.Arg348Ter), FBXO11 c.3850_3851insATTA(p.Thr1284fs)" |  | PIK3R1;2C.2;FBXO11; | 2C.2 |
| 382 | Breast cancer | female | 64 | PIK3CA c.3140A>G(p.His1047Arg), PTEN c.367C>T(p.His123Tyr), PTEN c.511C>T(p.Gln171Ter), SMARCA4 c.4990G>T(p.Glu1664Ter) | PIK3CA;1A.1;;PTEN;2C.1; | PTEN;2C.2;;PTEN;2C.2;;SMARCA4;2C.2; | 1A.1 |
| 383 | Lung cancer | female | 59 | KRAS c.35G>T(p.Gly12Val), TP53 c.613T>G(p.Tyr205Asp), RNF43 c.2057_2058insG(p.Ser687fs) | KRAS;1A.2; | TP53;2C.2;RNF43;2C.2; | 1A.2R |
| 384 | Pancreatic cancer | female | 55 | KRAS c.34G>C(p.Gly12Arg) | KRAS;2C.1; |  | 2C.1 |
| 385 | Pancreatic cancer | male | 46 | KRAS c.38G>A(p.Gly13Asp), TP53 c.742C>T(p.Arg248Trp) | KRAS;2C.1; | TP53;2C.2; | 2C.1 |
| 386 | sarcoma | male | 71 | PIK3CA c.3140A>G (p.His1047Arg), SETD2 c.4792C>T(p.Arg1598Ter) | PIK3CA;2C.1;; | SETD2;2D; | 2C.1 |
| 387 | Lung cancer | male | 31 | KRAS Amplification, KRAS c.34G>A(p.Gly12Ser), RNF43 c.2057_2058insG(p.Ser687fs), ATRX c.7156C>T(p.Arg2386Ter) | KRAS;1A.2; | RNF43;2C.2;ATRX;2C.2;KRAS; | 1A.2R |
| 388 | Hepatocellular carcinoma carcinoma | male | 2 | nl |  |  | normal |
| 389 | Gastric cancer | female | 64 | nl |  |  | normal |
| 390 | Gastric cancer | female | 52 | nl |  |  | normal |
| 391 | Lung cancer | male | 59 | SMARCA4 c.4276G>T(p.Glu1426Ter), TP53 c.614_621delATTTGGAT(p.Tyr205Ter) |  | SMAR4;TP53;2C.2; | 2C.2 |
| 392 | Breast cancer | female | 65 | PIK3CA c.1633G>A(p.Glu545Lys) | PIK3CA;1A.1; |  | 1A.1 |
| 393 | Hepatocellular carcinoma | female | 49 | nl |  |  | normal |
| 394 | Lung cancer | male | 53 | ERBB2 (HER2) c.2326_2327insTGT(p.Gly776delinsValCys) | ERBB2;1B; |  | 1B |
| 395 | kidney kidney | male | 52 | nl |  |  | normal |
| 396 | Gastric cancer | male | 57 | TP53 c.743G>A(p.Arg248Gln) |  | TP53;2C.2; | 2C.2 |
| 397 | Brain tumor | female | 55 | nl |  |  | normal |
| 398 | Ovarian cancer | female | 45 | TP53 c.617delT(p.Leu206fs) |  | TP53;2C.2; | 2C.2 |
| 399 | Lung cancer | male | 52 | BRCA1 c.1693G>T(p.Glu565Ter), TP53 c.724T>C(p.Cys242Arg), SMARCA4 c.2716delC(p.Arg906fs) |  | BRCA1;2C.1,TP53;2C.2;SMARCA4;2C.2 | 2C.1 |
| 400 | Colorectal cancer | male | 56 | BRAF c.1799T>A(p.Val600Glu) | BRAF;1A.2;KRAS/NRAS;1A.1; |  | 1A.2 |
| 401 | Colorectal cancer | female | 64 | TP53 c.844C>T(p.Arg282Trp), PPP2R1A c.547C>T(p.Arg183Trp) | KRAS/NRAS;1A.1; | TP53;2C.2;PPP2R1A; | 1A.1 |
| 402 | Prostate cancer | male | 86 | FGFR3 c.742C>T(p.Arg248Cys), CDKN2A c.79G>T(p.Glu27Ter) | FGFR3;2C.1; | CDKN2A;2D; | 2C.1 |
| 403 | Pancreatic cancer | male | 72 | KRAS c.35G>A(p.Gly12Asp) | KRAS;2C.1; |  | 2C.1 |
| 404 | Lymphoma Lymphoma | female | 36 | CDK12 c.1769C>G(p.Ser590Ter) | CDK12;2C.1;PD-L1 expression;2C.1; |  | 2C.1 |
| 405 | Pancreatic cancer | male | 72 | KRAS c.34G>C(p.Gly12Arg) | KRAS;2C.1; |  | 2C.1 |
| 406 | Colorectal cancer | male | 56 | KRAS c.38G>A(p.Gly13Asp), AKT1 c.155T>G(p.Leu52Arg) | KRAS;2C.1;KRAS;1A.1; | AKT1;2C.2; | 1A.1R |
| 407 | Pancreatic cancer | female | 64 | KRAS c.35G>A(p.Gly12Asp) | KRAS;2C.1; |  | 2C.1 |
| 408 | Esophageal cancer | male | 52 | TP53 c.742C>T(p.Arg248Trp) |  | TP53;2C.2; | 2C.2 |
| 409 | Prostate cancer | male | 68 | TP53 c.662_672delAGCCGCCTGAG(p.Glu221fs), c.215_216insG(p.Val73fs) |  | TP53;2C.2;TP53;2C.2; | 2C.2 |
| 410 | Pancreatic cancer | female | 71 | KRAS c.183A>C(p.Gln61His) | KRAS;2C.1; |  | 2C.1 |
| 411 | Colorectal cancer | male | 72 | KRAS c.180_181delTCinsAA(p.Gln61Lys) | KRAS;2C.1;KRAS;1A.1; |  | 1A.1R |
| 412 | Bladder ancer | male | 56 | nl | PD-L1 expression;1; |  | normal |
| 413 | Pancreatic cancer | female | 68 | KRAS c.35G>T(p.Gly12Val), TP53 c.577C>T(p.His193Tyr) | KRAS;2C.1; | TP53;2C.2; | 2C.1 |
| 414 | Prostate cancer | male | 65 | CDKN2A c.79G>T(p.Glu27Ter) |  | CDKN2A;2D; | 2D |
| 415 | Unknown primary | male | 72 | nl |  |  | normal |
| 416 | Colorectal cancer | male | 77 | NRAS c.182A>G(p.Gln61Arg), PIK3 c.1634A>C(p.Glu545Ala) | NRAS;1A.1;PIK3CA;2C.2;PIK3CA;2C.1; |  | 1A.1R |
| 417 | Prostate cancer | male | 73 | CDK12 c.858_859insCCTACG(p.Ser287fs) | CDK12;1A.1; |  | 1A.1 |
| 418 | Lung cancer | female | 49 | TP53 c.451C>T(p.Pro151Ser) |  | TP53;2C.2; | 2C.2 |
| 419 | Pancreatic cancer | male | 66 | KRAS c.35G>T(p.Gly12Val) | KRAS;2C.1; |  | 2C.1 |
| 420 | Prostate cancer | male | 72 | PTEN c. 371G>A(p.Cys124Tyr), FANCA c.2426_2427insA(p.Ala810fs) | KRAS;2C.1;FANCA;1A.1 |  | 1A.1 |
| 421 | Pancreatic cancer | female | 58 | NF2 c.1331C>A(p.Ser444Ter), NF2 c.1294G>T(p.Glu432Ter), TP53 c.817C>T(p.Arg273Cys), MYC amplifition |  | NF2;3;NF2;3;TP53;2C.2;MYC;2C.2; | 2C.2 |
| 422 | Ovarian cancer | female | 64 | TP53 c.711G>A(p.Met237Ile) |  | TP53;2C.2; | 2C.2 |
| 423 | Hepatocellular carcinoma | male | 53 | nl |  |  | normal |
| 424 | Pancreatic cancer | male | 55 | KRAS c.35G>A(p.Gly12Asp), TP53 c.480_496delGGCTCTAAGGT(p.Met160fs) | KRAS;2C.1; | TP53;2C.2; | 2C.1 |
| 425 | Brain tumor | female | 49 | NF1 c.3958G>T(p.Glu1320Ter), PTPN c.227A>G(p.Glu76Gly) | NF1;2C.1; | PTPN11;2C.2; | 2C.1 |
| 426 | Pancreatic cancer | female | 59 | KRAS c.35G>A(p.Gly12Asp), TP53 c.839G>C(p.Arg280Thr) | KRAS;2C.1; | TP53;2C.2; | 2C.1 |
| 427 | Unknown primary | male | 40 | nl |  | PD-L1 expression; | normal |
| 428 | Lung cancer | male | 52 | EGFR exon 19 c.2235_2249delGGAATTAAGAGAAGC(p.Glu746_Ala750del) | EGFR;1A.1; |  | 1A.1 |
| 429 | Lung cancer | male | 79 | KRAS c.35G>C(p.Gly12Ala), TP53 c.469G>T(p.Val157Phe) | KRAS;1A.2; | TP53;2C.2; | 1A.2R |
| 430 | Sarcoma | female | 55 | IDH1 c.394C>T(p.Arg132Cys) | IDH1;2C.1; |  | 2C.1 |
| 431 | Cholangiocarcinoma | male | 63 | nl |  |  | normal |
| 432 | Lung cancer | male | 38 | PIK3 c.1633G>A(p.Glu545Lys),TP53 c.949C>T(p.Gln317Ter) | PIK3CA;2C.1; | TP53;2C.2; | 2C.1 |
| 433 | Breast cancer | female | 49 | TP53c.743G>A(p.Arg248Gln) |  | TP53;2C.2; | 2C.2 |
| 434 | Pancreatic cancer | male | 56 | nl |  |  | normal |
| 435 | Cervical cancer | female | 53 | ΑΚΤ3 c.49G>A(p.Glu17Lys) |  | AKT3;2C.2;;AKT1;2C.2; | 2C.2 |
| 436 | Colorectal cancer | male | 48 | nl | KRAS/NRAS;1A.1; |  | 1A.1 |
| 437 | Thymic carcinoma | male | 52 | nl |  |  | normal |
| 438 | Small intestine Small intestine | male | 67 | KRAS c.182_183delAAinsTT(p.Gln61Leu), SMAD4 c.1081C>T(p.Arg361Cys), TP53 c.733G>A(p.Gly245Ser) | KRAS;2C.1; | SMAD4;2C.2;TP53;2C.2; | 2C.1 |
| 439 | Breast cancer | female | 72 | TP53 c.742C>T(p.Arg248Trp) |  | TP53;2C.2; | 2C.2 |
| 440 | Brain tumor | female | 69 | NF1 c.5861C>G(p.Ser1954Ter), RB1 c.2245delT(p.Tyr749fs | NF1;2C.1; | RB1;2C.2; | 2C.1 |
| 441 | Ovarian cancer | female | 68 | TP53 c.659A>G(p.Tyr220Cys) | PD-L1 expression;2C.1; | TP53;2C.2; | 2C.2 |
| 442 | Gastric cancer | female | 72 | SF3B1 c.2098A>G(p.Lys700Glu) |  | SF3B1;3; | 3 |
| 443 | Melanoma | male | 71 | NRAS c.182A>G(p.Gln61Arg), FGFR3 c.1921G>A(p.Asp641Asn), TERT c.-146C>T | NRAS;2C.1;FGFR3;2C.1; | TERT;2D; | 2C.1 |
| 444 | Pancreatic cancer | male | 45 | nl |  |  | normal |
| 445 | Unknown primary | male | 55 | PIK3 c.1633G>A(p.Glu545Lys) | PIK3CA;2C.1; |  | 2C.1 |
| 446 | Breast cancer | female | 64 | PIK3 c.3127A>G(p.Met1043Val), KIT Amplifition, PDGFRA Amplifition, FGFR1 Amplifition, MYC Amplifition, MDM2 Amplifition, EIF3E(1)-RSPO2(2) | PIK3CA;1A.1; | RSPO2 fusion;KIT;PDGFRA;2C.1FGFR1;MYC;2C.2;MDM2; | 1A.1 |
| 447 | Ovarian cancer | female | 61 | TP53 c.379T>A(p.Ser127Thr), TBL1XR1(1)-PIK3(2) |  | TP53;2C.2;PIK3CA fusion; | 2C.2 |
| 448 | Pancreatic cancer | male | 68 | KRAS c.34G>T(p.Gly12Cys), TP53 c.524G>A(p.Arg175His) | KRAS;2C.1; | TP53;2C.2; | 2C.1 |
| 449 | Colorectal cancer | male | 70 | NRAS c.34G>A(p.Gly12Ser) | NRAS;1A.1; |  | 1A.1R |
| 450 | Pancreatic cancer | male | 55 | KRAS c.35G>A(p.Gly12Asp), PIK3 c.1633G>A(p.Glu545Lys), TP53 c.524G>A(p.Arg175His) | KRAS;2C.1;PIK3CA;2C.1; | TP53;2C.2; | 2C.1 |
| 451 | Sarcoma | male | 29 | WHSC1L1(1) - FGFR1(2) | FGFR1 fusion;2C.1;PD-L1 expression;2C.1; |  | 2C.1 |
| 452 | Prostate cancer | male | 77 | ALK Amplifition, PDGFRA Amplifition, KIT Amplifition, RICTOR Amplifition, ESR1 Amplifition, MYC Amplifition, NTRK2 Amplifition, CDK2 Amplifition, AR Amplifition | AR;2C.1; | ALK;PDGFRA;2C.1KIT;RICTOR;ESR1;2C.2;MYC;2C.2;NTRK2;3;CDK2; | 2C.1 |
| 453 | Pancreatic cancer | female | 75 | KRAS c.35G>A(p.Gly12Asp), MET(13)-MET(15) | KRAS;2C.1;MET fusion;2C.1; |  | 2C.1 |
| 454 | Lung cancer | male | 36 | RAD50 c.2983_2986delGAAA(p.Glu995fs), KIF5B(15)-RET(11) | RAD50;2C.1;RET fusion;1A.1; |  | 1A.1 |
| 455 | Lung cancer | female | 48 | EGFR c.2235_2249delGGAATTAAGAGAAGC (p.Glu746_Ala750del), TP53 c.730G>A (p.Gly244Ser) | EGFR;1A.1; | TP53;2C.2; | 1A.1 |
| 456 | Pancreatic cancer | male | 55 | KRAS c.35G>A(p.Gly12Asp), CDKN2A c.47_50delTGGC(p.Leu16fs) | KRAS;2C.1; |  | 2C.1 |
| 457 | Unknown primary | male | 64 | SMARCA4 c.4081G>T(p.Glu1361Ter), TP53 c.535C>T(p.His179Tyr) | PD-L1 expression;2C.1; | SMAR4;TP53;2C.2; | 2C.2 |
| 458 | Ovarian cancer | female | 53 | MYCL Amplifition, MYC Amplifition, ERBB2 Amplifition, CCNE1 Amplifition | ERBB2;2C.1; | MYCL;MYC;2C.2;CCNE1;2C.2; | 2C.1 |
| 459 | Pancreatic cancer | male | 52 | KRAS c.35G>A(p.Gly12Asp) | KRAS;2C.1; |  | 2C.1 |
| 460 | Head and Neck Head and Neck | male | 55 | SMARCA4 c.1345G>T(p.Glu449Ter), TP53 c.1027G>T(p.Glu343Ter) |  | SMARCA4;2C.2;TP53;2C.2; | 2C.2 |
| 461 | Colorectal cancer | male | 56 | FBXW7 c.1513C>T(p.Arg505Cys), NOTCH2 c.7198C>T(p.Arg2400Ter),TP53 c.844C>T(p.Arg282Trp) | KRAS/NRAS;1A.1; | FBXW7;2C.2;NOTCH2;TP53;2C.2; | 1A.1 |
| 462 | Pancreatic cancer | male | 67 | KRAS c.35G>A(p.Gly12Asp) | KRAS;2C.1; |  | 2C.1 |
| 463 | Tongue Tongue | male | 72 | nl |  |  | normal |
| 464 | Pancreatic cancer | male | 71 | KRAS c.35G>T(p.Gly12Val), TP53 c.566_567insCCCT(p.Gln192fs) | KRAS;2C.1; | TP53;2C.2; | 2C.1 |
| 465 | Lung cancer | male | 77 | BRAF c.1397G>T(p.Gly466Val), KRAS c.436G>C(p.Ala146Pro), STK11 c.182delG(p.Gly61fs), MYC Amplifition | KRAS;1A.2;STK11;2C.1;STK11;2C.1; | BRAF;2C.2;MYC;2C.2; | 1A.2R |
| 466 | Metastatic clear cell rcinoma Metastatic clear cell rcinoma | male | 68 | nl |  |  | normal |
| 467 | Pancreatic cancer | male | 64 | KRAS c.35G>A(p.Gly12Asp), PIK3 c.331A>G(p.Lys111Glu), CDKN2A c.172C>T(p.Arg58Ter), TP53 c.472C>G(p.Arg158Gly) | KRAS;2C.1;PIK3CA;2C.1; | CDKN2A;2D;TP53;2C.2; | 2C.1 |
| 468 | Breast cancer | female | 59 | NF1 c.4812_4813insT(p.Ile1605fs), TP53 c.742C>T(p.Arg248Trp) | NF1;2C.1; | TP53;2C.2; | 2C.1 |
| 469 | Vaginal | female | 65 | nl |  |  | normal |
| 470 | Colorectal cancer | male | 55 | KRAS c.35G>T(p.Gly12Val) | KRAS;1A.1R; |  | 1A.1R |
| 471 | Pancreatic cancer | male | 56 | KRAS c.34G>C(p.Gly12Arg), CDKN2A c.47_50delTGGC(p.Leu16fs), TP53 c.743G>A(p.Arg248Gln) | KRAS;2C.1; | CDKN2A;2D;TP53;2C.2; | 2C.1 |
| 472 | Lung cancer | male | 69 | NF1 c.3616G>T(p.Glu1206Ter), NF1 c.8047G>T(p.Gly2683Ter) | NF1;2C.1;NF1;2C.1; |  | 2C.1 |
| 473 | Melanoma | male | 66 | AR c.2623C>T(p.His875Tyr), TSC2 c.3179G>A(p.Trp1060Ter) | TSC2;2C.1; | AR;2C.2; | 2C.1 |
| 474 | Pancreatic cancer | male | 78 | KRAS c.35G>T(p.Gly12Val) | KRAS;2C.1; |  | 2C.1 |
| 475 | Breast cancer | female | 73 | CCND1 Amplifition, FGF19 Amplifition, FGF3 Amplifition, CCND2 Amplifition |  | CCND1;2C.2;FGF19;FGF3;CCND2;2C.2; | 2C.2 |
| 476 | Gastric cancer | male | 71 | PTCH1 c.3944_3945insT(p.Tyr1316fs) | PTCH1;2C.1; |  | 2C.1 |
| 477 | Colorectal cancer | male | 69 | KRAS c.35G>C(p.Gly12Ala), TP53 c.836_837delGG(p.Gly279fs) | ;KRAS;1A.1R; | TP53;2C.2; | 1A.1R |
| 478 | Lung cancer | female | 41 | CHEK2 c.1169A>C(p.Tyr390Ser) | CHEK2;2C.1; |  | 2C.1 |
| 479 | Cholangiocarcinoma | male | 59 | TP53 c.452C>G(p.Pro151Arg) |  | TP53;2C.2; | 2C.2 |
| 480 | esophageal cancer | male | 67 | UNKNOWN |  |  | normal |
| 481 | Endometrial cancer | female | 53 | FGFR2 c.755C>G(p.Ser252Trp) | FGFR2;2C.1; |  | 2C.1 |
| 482 | Ovarian cancer | female | 52 | TP53 c.452C>G(p.Pro151Arg) |  | TP53;2C.2; | 2C.2 |
| 483 | Breast cancer | female | 67 | ERBB2 Amplifition, CDK2 Amplifition, MYCL Amplifition, MDM2 Amplifition, CDK4 Amplifition | ERBB2;1A.1; | CDK2;2C.2;MYCL;2D;MDM2;**2C.2;**CDK4;2C.2; | 1A.1 |
| 484 | Teratoma | male | 51 | nl |  |  | normal |
| 485 | Lung cancer | female | 71 | SMARCA4 c.698_711delGCCCTGGCCCTGGC(p.Gly233fs), TP53 c.801delG(p.Asn268fs) |  | SMAR4;TP53;2C.2; | 2C.2 |
| 486 | Breast cancer | female | 72 | TP53 c.818G>A(p.Arg273His) |  | TP53;2C.2; | 2C.2 |
| 487 | Pancreatic cancer | male | 77 | KRAS c.35G>A(p.Gly12Asp), TP53 c.578A>G (p.His193Arg) | KRAS;2C.1; | TP53;2C.2; | 2C.1 |
| 488 | Endometrial cancer | female | 75 | PIK3 c.1624G>A(p.Glu542Lys), AKT1 c.49G>A(p.Glu17Lys), ARID1A c.4344delA(p.Gly1449fs), CTNNB1 c.98C>G(p.Ser33Cys) | PIK3CA;2C.1; | AKT1;2C.2;ARID1A;2C.2;CTNNB1;2C.2; | 2C.1 |
| 489 | Hepatocellular carcinoma | male | 81 | nl |  |  | normal |
| 490 | Gasrtric | male | 59 | NOTCH1 c.3225G>A(p.Trp1075Ter), TP53 c.734G>A(p.Gly245Asp) |  | NOTCH1;2C.2;TP53;2C.2; | 2C.2 |
| 491 | Pancreatic cancer | male | 63 | KRAS c.35G>A(p.Gly12Asp) | KRAS;2C.1; |  | 2C.1 |
| 492 | Colorectal cancer | male | 71 | KRAS c.34G>T(p.Gly12Cys), ATRX c.5561T>A(p.Leu1854Ter), TP53 c.281C>A(p.Ser94Ter) | KRAS;1A.1R; | ATRX;2C.2;TP53;2C.2; | 1A.1R |
| 493 | Ovarian cancer | female | 52 | PIK3CA c.3140A>G(p.His1047Arg), TP53 c.1014_1018delCGAGA(p.Glu339fs), ESR1(5)-AKAP12(4) | PIK3CA;2C.1; | TP53;2C.2;ESR1 fusion;2C.2 | 2C.1 |
| 494 | Colorectal cancer | female | 46 | KRAS Amplification, ESR1 Amplification, MYC Amplification, FGFR2 Amplification, CCND1 Amplification, FGF19 Amplification, FGF3 Amplification TP53 c.487_488insCT(p.Tyr163fs), SEC16A(1) - FGFR2(7) | KRAS/NRAS;1A.1;FGFR2;2C.1; | TP53;2C.2;KRAS;ESR1;2C.2;MYC;2C.2;CCND1;2C.2;FGF19;FGF3; | 1A.1 |
| 495 | Sarcoma | male | 55 | CCND3 Amplification, ATRX c.972T>A(p.Cys324Ter), WHSC1L1(1)-FGFR1(2) | FGFR1 fusion;2C.1; | ATRX;2C.2;CCND3;2C.2; | 2C.1 |
| 496 | Prostate cancer | male | 82 | PTCH1 c.3944_3945insT(p.Tyr1316fs), CDK12 c.1069delA(p.Ser357fs) | PTCH1;2C.1;CDK12;1A.1; |  | 1A.1 |
| 497 | Endometrial cancer | female | 49 | TERT c.-124C>T(C228T) |  | TERT;2D; | 2D |
| 498 | Brain tumor | male | 65 | MYCN Amplification, PDGFRA Amplification, CDK6 Amplification, MET Amplification, TP53 c.731G>A(p.Gly244Asp) | MET;2C.1; | TP53;2C.2;MYCN;2D;PDGFRA;2C.2;CDK6;2C.2 | 2C.1 |
| 499 | Pancreatic cancer | male | 69 | nl |  |  | normal |
| 500 | Pancreatic cancer | male | 73 | KRAS c.35G>T(p.Gly12Val) | KRAS;2C.1; |  | 2C.1 |
| 501 | Pancreatic cancer | male | 41 | ERBB2 Amplification, KRAS c.183A>C(p.Gln61His), TP53 c.524G>A(p.Arg175His) | KRAS;2C.1;ERBB2;2C.1; | TP53;2C.2; | 2C.1 |
| 502 | Angioblastoma | female | 52 | nl |  |  | normal |
| 503 | Gasrtric cancer | male | 89 | nl |  |  | normal |
| 504 | Lung cancer | male | 64 | nl |  |  | normal |
| 505 | Pancreatic cancer | male | 75 | nl |  |  | normal |
| 506 | Pancreatic cancer | female | 52 | KRAS c.35G>T(p.Gly12Val), CDKN2A c.135_138delTCGG(p.Arg46fs) | KRAS;2C.1; | CDKN2A;2D; | 2C.1 |
| 507 | Breast cancer | female | 50 | PIK3CA c.1633G>A(p.Glu545Lys) | PIK3CA;1A.1; |  | 1A.1 |
| 508 | Prostate cancer | male | 89 | nl |  |  | normal |
| 509 | Gasrtric cancer | female | 73 | nl |  |  | normal |
| 510 | Colorectal cancer | male | 71 | KRAS c.35G>A(p.Gly12Asp) | KRAS;2C.1;KRAS;1A.1; |  | 1A.1R |
| 511 | Colorectal cancer | female | 69 | BRAF c.1799T>A(p.Val600Glu), TP53 c.394A>C (p.Lys132Gln) | BRAF;1A.2;KRAS/NRAS;1A.1; | TP53;2C.2; | 1A.2 |
| 512 | Colorectal cancer | male | 58 | KRAS c.35G>A(p.Gly12Asp), PIK3CA c.3140A>G(p.His1047Arg), TP53 c.586C>T(p.Arg196Ter) | KRAS;1A.1R;PIK3CA;2C.2; | TP53;2C.2; | 1A.1R |
| 513 | Pancreatic cancer | male | 66 | KRAS c.34G>C(p.Gly12Arg) | KRAS;2C.1; |  | 2C.1 |
| 514 | Prostate cancer | male | 76 | AGAP3(9) - BRAF(9) fusion |  | BRAF fusion;2C.2; | 2C.2 |
| 515 | Gastric cancer | male | 60 | nl |  |  | normal |
| 516 | Head and Neck cancer | female | 53 | HRAS c.182A>G(p.Gln61Arg), PIK3CA c.3140A>G(p.His1047Arg) | PIK3CA;2C.1; | HRAS;2C.2; | 2C.1 |
| 517 | Ovarian cancer | female | 66 | MYC Amplification, FGFR2 Amplification, CCNE1 Amplification, AKT2 Amplification, AXL Amplification | FGFR2;2C.1; | MYC;2C.2;CCNE1;2C.2;AKT2;2C.2;AXL;2D; | 2C.1 |
| 518 | Prostate cancer | male | 67 | TMPRSS2- ERG Fusion, FGFR1 Amplification, PMS2 c.874delT(p.Leu625Ter), TP53 c.403T>C (p.Cys135Arg) | PMS2;1B;FGFR1;2C.1 | TP53;2C.2;ERG fusion;2C.2; | 1B |
| 519 | Colorectal cancer | male | 69 | EIF3E(1) - RSPO2(2) | KRAS/NRAS;1A.1; | RSPO2 fusion;3; | 1A.1 |
| 520 | Cholangiocarcinoma | male | 43 | IDH1 c.394C>T(p.Arg132Cys), ARID1A c.598C>T(p.Gln200Ter) | IDH1;2C.1; | ARID1A;2C.2; | 2C.1 |
| 521 | Pancreatic cancer | male | 64 | KRAS c.35G>A(p.Gly12Asp), ARID1A c.6466delA(p.Ser2156fs), TP53 c.742C>T(p.Arg248Trp) | KRAS;2C.1; | ARID1A;2C.2;TP53;2C.2; | 2C.1 |
| 522 | Breast cancer | female | 73 | MDM4 Amplification, AKT3 Amplification, PIK3CA c.1624G>A (p.Glu542Lys), ERBB2 c.929C>T(p.Ser310Phe) | PIK3CA;1A.1;PIK3CA;2C.1;ERBB2;2C.1; | MDM4;2C.2;AKT3;2C.2; | 1A.1 |
| 523 | Head and Neck cancer | female | 81 | PIK3CA c.1357G>A(p.Glu453Lys) | PIK3CA;2C.1; |  | 2C.1 |
| 524 | Pancreatic cancer | male | 76 | KRAS c.35G>T(p.Gly12Val), TP53 c.526T>A( p.Cys176Ser) | KRAS;2C.1; | TP53;2C.2; | 2C.1 |
| 525 | Colorectal cancer | male | 53 | ERBB2 ampl., RNF43 c.130C>T(p.Gln44Ter), TP53 c.993G>T(p.Gln331His) | KRAS/NRAS wildtype + ERRB2 amplification;1A.2; | RNF43;2C.2;TP53;2C.2; | 1A.2 |
| 526 | Pancreatic cancer | male | 65 | KRAS c.35G>A(p.Gly12Asp), CDKN2A c.247C>T(p.His83Tyr), TP53 c.226_227insG(p.Ala76fs) | KRAS;2C.1; | CDKN2A;2D;TP53;2C.2; | 2C.1 |
| 527 | Breast cancer | female | 63 | PIK3CA c.1357G>A(p.Glu453Lys), PTCH1 c.3944_3945insT(p.Tyr1316fs), RNF43 c.2057_2058insG(p.Ser687fs), MDM2 Amplification, CCND1 Amplification, FGF19 Amplification, IGFR1 Amplification Αναδιάταξη ESR1(2) - CCDC170(8) | PIK3CA;1A.1;PTCH1;2C.1; | RNF43;2C.2;ESR1 fusion;2C.2;MDM2;2C.2;CCND1;2C.2;FGF19;2C.2;IGFR1;3; | 1A.1 |
| 528 | Lung cancer | male | 56 | SMAD4 c.1051G>C(p.Asp351His) |  | SMAD4;2C.2; | 2C.2 |
| 529 | Brain tumor | male | 52 | CDK4 amplification, NF1 c.1381C>T(.Arg461Ter), NF1 c.1484_1487delCCAT (p.Ser495fs) | NF1;2C.1;NF1;2C.1; | CDK4;2C.2; | 2C.1 |
| 530 | Colorectal cancer | male | 64 | KRAS c.38G>A (p.Gly13Asp), TP53 c.844C>T (p.Arg282Trp) | KRAS;2C.1;KRAS;1A.1; | TP53;2C.2; | 1A.1R |
| 531 | Unknown primary | male | 69 | KRAS c.35G>A(p.Gly12Asp) | KRAS;2C.1; |  | 2C.1 |
| 532 | Gastric cancer | male | 46 | MSH2 c.1056_1057delTA(p.Asp352fs), MSH2 c.1874delT(p.Leu625fs), FBXW7 c.1393C>T(p.Arg465Cys), PTCH1 c.2701delC(p.Gln901fs), CCND1 c.860C>T(p.Pro287Leu), NF1 c.146_147insA(p.Tyr49Ter), NF1c.6852_6855delTTAC(p.Tyr2285fs), GNA11 c.548G>A(p.Arg183His), GNAS c.602G>A(p.Arg201His) | MSH2;1B;MSH2;1B;PTCH1;2C.1;NF1;2C.1;NF1;2C.1; | FBXW7;2C.2;CCND1;2C.2;GNA11;GNAS;2C.2; | 1B |
| 533 | Cholangiocarcinoma | male | 42 | KRAS c.35G>T(p.Gly12Val), CDKN2A c.131_132insA(p.Tyr44Ter), TP53 c.742C>T(p.Arg248Trp) | KRAS;2C.1; | CDKN2A;2D;TP53;2C.2; | 2C.1 |
| 534 | Sarcoma | female | 56 | CDK4 amplification, MDM2 amplification |  | CDK4;2C.2;MDM2; | 2C.2 |
| 535 | Cholangiocarcinoma | female | 55 | nl |  |  | normal |
| 536 | Unknown primary | female | 49 | CD74(6) - ROS1(34) | ROS1 fusion;2C.1; |  | 2C.1 |
| 537 | Pancreatic cancer | male | 64 |  |  |  | normal |
| 538 | Unknown primary | male | 58 | TP53 c.349_370delGGGACAGCCAAGTCTGTGACTT (p.Gly117fs), FGFR1 fusion\| | FGFR1 fusion;2C.1; | TP53;2C.2; | 2C.1 |
| 539 |  | male | 56 |  |  |  | normal |
| 540 | Cholangiocarcinoma | female | 46 | PALB2 c.2257C>T (p.Arg753Ter) | PALB2;2C.1; |  | 2C.1 |
| 541 | Prostate cancer | male | 78 | TP53 c.524G>T (p.Arg175Leu) |  | TP53;2C.2; | 2C.2 |
| 542 | Cholangiocarcinoma | male | 55 | MYC amplification |  | MYC;2C.2; | 2C.2 |
| 543 | Renal cancer | male | 71 |  |  |  | normal |
| 544 | Pancreatic cancer | female | 48 | NBN c.657_661delACAAA (p.Lys219Asnfs*16) | NBN;2C.1; |  | 2C.1 |
| 545 | Breast cancer | female | 59 | PIK3CA c.3140A>G (p.His1047Arg) | PIK3CA;1A.1; |  | 1A.1 |
| 546 | kidney cancer | male | 56 |  |  |  | normal |
| 547 | Gastric cancer | male | 64 | KRAS c.35G>A (p.Gly12Asp), PIK3CA c.3140A>G (p.His1047Arg) | KRAS;2C.1;PIK3CA;2C.1; |  | 2C.1 |
| 548 | Gastric cancer | male | 66 | TP53 c.966delA (p.Leu323TrpfsTer22), ERBB2 amplification | ERBB2;1A.1; | TP53;2C.2; | 1A.1 |
| 549 | Endometrial cancer | male | 69 | PIK3CA c.3140A>G (p.His1047Arg), PIK3R1c.1251delT (p.Lys419AsnfsTer4), CTNNB1 c.98C>T (p.Ser33Phe), ARID1A c.4670_4673delCCTC (p.Pro1557LeufsTer7) | PIK3CA;2C.1; | PIK3R1;2C.2;CTNNB1;2C.2;ARID1A;2C.2; | 2C.1 |
| 550 | Pancreatic cancer | male | 68 | PIK3CA c.3140A>G (p.His1047Arg), PIK3R1c.1251delT (p.Lys419AsnfsTer4), CTNNB1 c.98C>T (p.Ser33Phe), ARID1A c.4670_4673delCCTC (p.Pro1557LeufsTer7) | KRAS;2C.1;STK11;2C.1; |  | 2C.1 |
| 551 | Breast cancer | female | 64 | PIK3CA c.3140A>G (p.His1047Arg), ESR1 c.1610A>C (p.Tyr537Ser) | PIK3CA;1A.1;PIK3CA;2C.1; | ESR1;2C.2; | 1A.1 |
| 552 | ADRENAL CANCER | female | 66 | CREBBP\| |  | CREBBP;2D; | 2D |
| 553 | Ovarian cancer | male | 72 |  | PD-L1 expression;2C.1; |  | normal |
| 554 | Brain tumor | female | 78 | PTEN c.277C>T (p.His93Tyr), TP53 c.642_643delTA (p.His214fs) |  | PTEN;2C.2;;TP53;2C.2; | 2C.2 |
| 555 | Breast cancer | female | 68 |  |  |  | normal |
| 556 | Prostate cancer | male | 74 | PTEN c.716_719delTGTA (p.Met239fs), TP53 c.1031T>C (p.Leu344Pro), ERG FUSION | PTEN;2C.1; | PTEN;2C.2;;TP53;2C.2;ERG fusion;2C.2; | 2C.1 |
| 557 | Brain tumor | male | 77 | PTEN c.203A>G (p.Tyr68Cys), EGFR c.323G>A (p.Arg108Lys) CDK4 AMPLIFICATION | PTEN;2C.1; | EGFR;CDK4;2C.2; | 2C.1 |
| 558 | Breast cancer | female | 72 | ESR1 c.1609T>G (p.Tyr537Asp) |  | ESR1;2C.2; | 2C.2 |
| 559 | kidney cancer | female | 63 | TP53 c.642_643delTA (p.His214fs) |  | TP53;2C.2;TP53;2C.2; | 2C.2 |
| 560 | Cholangiocarcinoma | male | 67 | IDH1 c.394C>T (p.Arg132Cys) | IDH1;2C.1;I |  | 2C.1 |
| 561 | sarcoma | female | 58 | CREBBP c.5154G>A (p.Trp1718Ter)" |  | CREBBP;2D; | 2D |
| 562 | Pancreatic cancer | male | 77 | KRAS\|STK11\| | KRAS;2C.1;STK11;2C.1; |  | 2C.1 |
| 563 | Lung cancer | male | 46 |  |  |  | normal |
| 564 | Pancreatic cancer | female | 66 | KRAS c.35G>A (p.Gly12Asp, TP53 c.586C>T (p.Arg196Ter) | KRAS;2C.1; | TP53;2C.2;TP53;2C.2; | 2C.1 |
| 565 | Breast cancer | female | 68 | ESR1\|AKT1\|AR fusion\|CCND1\|FGF3\|FGF19\| |  | ESR1;2C.2;AKT1;2C.2;AR fusion;CCND1;2C.2;FGF3;FGF19; | 2C.2 |
| 566 | VULVAR CANCER | female | 57 | TP53 c.949C>T (p.Gln317Ter) |  | TP53;2C.2; | 2C.2 |
| 567 | Pancreatic cancer | male | 71 | KRAS c.35G>T (p.Gly12Val), TP53 c.577C>T (p.His193Tyr) | KRAS;2C.1; | TP53;2C.2; | 2C.1 |
| 568 | Gastric cancer | male | 76 | TP53 c.745A>T (p.Arg249Trp) |  | TP53;2C.2; | 2C.2 |
| 569 | Pancreatic cancer | male | 72 | KRAS c.35G>T (p.Gly12Val), TP53 c.832C>T (p.Pro278Ser) | KRAS;2C.1; | TP53;2C.2; | 2C.1 |
| 570 | Sarcoma | female | 38 | FGFR1 fusion\| | FGFR1 fusion;2C.1; |  | 2C.1 |
| 571 | Pancreatic cancer | female | 61 | KRASExon 2 c.34G>C (p.Gly12Arg) \|MYC amplification, CCND3 amplification | KRAS;2C.1; | KRAS;2C.2;MYC;2C.2;CCND3;2C.2 | 2C.1 |
| 572 | Cholangiocarcinoma | male | 490 | PPP2R1A c.547C>T (p.Arg183Trp) |  | PPP2R1A;2C.2; | 2C.2 |
| 573 | Ampullary cancer | female | 56 | KRAS Exon 3 c.183A>T (p.Gln61His) | KRAS;2C.1; |  | 2C.1 |
| 574 | Breast cancer | female | 64 |  |  |  | normal |
| 575 | Unknown primary | female | 56 | RET c.2410G>A (p.Val804Met), NRAS Exon 3 c.181C>A (p.Gln61Lys), TERT  c.-146C>T | RET;2C.1; | NRAS;2D;TERT;2D; | 2C.1 |
| 576 | SALIVARY GLAND CARCINOMA | female | 72 |  |  |  | normal |
| 577 | Endometrial cancer | female | 52 | TP53 c.584T>C (p.Ile195Thr), PPP2R1A c.536C>G (p.Pro179Arg) |  | TP53;2C.2;PPP2R1A;2C.2 | 2C.2 |
| 578 | Gastric cancer | male | 56 | TP53 c.743G>A (p.Arg248Gln) |  | TP53;2C.2; | 2C.2 |
| 579 | Unknown primary | female | 72 |  |  |  | normal |
| 580 | sarcoma | male | 41 | FANCD2\|AR\| | FANCD2;2C.1; | AR;2C.2; | 2C.1 |
| 581 | Breast cancer | female | 59 | NOTCH1 c.1324C>T (p.Gln442Ter) |  | NOTCH1;2C.2; | 2C.2 |
| 582 | Pancreatic cancer | female | 55 | KRAS, c.35G>A (p.Gly12Asp),TP53 c.833C>T (p.Pro278Leu) | KRAS;2C.1; | TP53;2C.2; | 2C.1 |
| 583 | Desmoplastic small round cell tumor | male | 40 |  |  |  | normal |
| 584 | Sarcoma | male | 42 | TP53 c.499C>T (p.Gln167Ter) |  | TP53;2C.2; | 2C.2 |
| 585 | Breast cancer | female | 66 | PIK3CA c.1035T>A (p.Asn345Lys) | PIK3CA;1A.1; |  | 1A.1 |
| 586 | Pancreatic cancer | female | 78 | KRAS, c.35G>A (p.Gly12Asp), GNAS c.602G>A (p.Arg201His) | KRAS;2C.1; | GNAS;2C.2; | 2C.1 |
| 587 | Penile cancer | male | 36 | ΑΤΜ c.1215delT (p.Asn405Lysfs*15), CREBBP c.1618C>T (p.Gln540Ter) | ATM;2C.1; | CREBBP;2D; | 2C.1 |
| 588 | Prostate cancer | male | 71 | KRAS c.35G>A, (p.Gly12Asp), PIK3CA c.263G>A (p.Arg88Gln), FGFR1 c.1729A>G (p.Asn577Asp), CDK12 c.597_598delAA (p.His201SerfsTer28), MSH6 c.3312delT (p.Phe1104LeufsTer11), NF1 c.68delT (p.Ile23LysfsTer21), NBN c.2051delA (p.Asn684IlefsTer25), TP53 c.799C>T (p.Arg267Trp) | KRAS;2C.1;PIK3CA;2C.1;FGFR1;2C.1;CDK12;1A.1;MSH6;1B;NF1;2C.1;NBN;1A.1; | TP53;2C.2; | 1A.1 |
| 589 | Signet ring cell carcinoma | male | 51 | MSH6 c.3559G>T (p.Glu1187Ter, BRAF c.1397G>A (p.Gly466Glu), BRCA1 c.607G>T (p.Glu203Ter), ATM c.8592C>A (p.Tyr2864Ter), FANCD2 c.4029delG (p.His1344IlefsTer24), PTCH1 c.3535G>T (p.Gly1179Ter) | MSH6;1B;ATM;2C.1;FANCD2;2C.1;PTCH1;2C.1; | BRAF;2C.2;BRCA1; | 1B |
| 590 | Cholangiocarcinoma | male | 43 | CDKN2A c.341C>A (p.Pro114His) |  | CDKN2A;2D; | 2D |
| 591 | Pancreatic cancer | female | 66 |  |  |  | normal |
| 592 | Parotid gland CANCER | female | 67 | TERT c.-146C>T, TP53 c.818G>A (p.Arg273His) | PD-L1 expression;1;PD-L1 expression;2C.1; | TERT;2D;TP53;2C.2; | 2C.2 |
| 593 | Prostate cancer | male | 62 | Myc amplification |  | MYC;2C.2; | 2C.2 |
| 594 | Sarcoma | male | 40 |  |  |  | normal |
| 595 | Ovarian cancer | female | 68 | PIK3CA c.1635G>T (p.Glu545Asp) | PIK3CA;2C.1; |  | 2C.1 |
| 596 | Breast cancer | female | 68 |  |  |  | normal |
| 597 | Prostate cancer | male | 76 | AR c.2632A>G (p.Thr878Ala), POLE c.5729delG (p.Cys1910SerfsTer89), ERG TMPRSS2(2) - ERG(4) | AR;2C.1;POLE;2C.1; | ERG fusion;2C.2; | 2C.1 |
| 598 | Endometrial cancer | female | 72 | PTEN c.389G>A (p.Arg130Gln, TP53 c.702C>A (p.Tyr234Ter |  | PTEN;2C.2;TP53;2C.2; | 2C.2 |
| 599 | Pancreatic cancer | male | 71 | KRAS c.35G>T(p.Gly12Val) | KRAS;2C.1; |  | 2C.1 |
| 600 | Pancreatic cancer | male | 76 | KRAS c.35G>T(p.Gly12Val), TP53 c.524G>A (p.Arg175His) | KRAS;2C.1; | TP53;2C.2; | 2C.1 |
| 601 | Esophageal Cancer | female | 50 | ATM c.6247G>T (p.Gly2083Ter), CDK4 AMPLIFICATION | ATM;2C.1 | PIK3R1;2D;TP53;2C.2; | 2C.1 |
| 602 | GASTRIC CANCER | male | 52 | BRCA2 c.5681_5682insA (p.Tyr1894Ter), KRAS c.35G>A (p.Gly12Asp), PIK3R1 c.1701delA (p.Lys567AsnfsTer7), TP53 c.436_437insT (p.Trp146LeufsTer3) | BRCA2;2C.1;KRAS;2C.1; | HNF1A;3 | 3 |
| 603 | SOFT TISSUE SARCOMA | male | 68 | HNF1A c.864delG (p.Pro291GlnfsTer51) |  |  | normal |
| 604 | LEIOMYOSARCOMA | male | 46 |  |  |  | normal |
| 605 | THYMUS CELL CARCINOMA | male | 59 |  |  |  | normal |
| 606 | HEAD AND NECK CANCER | male | 57 | TP53 c.844C>T (p.Arg282Trp) |  | TP53;2C.2 | 2C.2 |
| 607 | SARCOMA | female | 55 |  |  | NL | normal |
| 608 | PAROTID GLAND CANCER | female | 59 | TERT c.-146C>T, TP53 C. .537T>A (p.His179Gln) |  | TERT;2D;TP53;2C.2 | 2C.2 |
| 609 | ANGIOSARCOMA | male | 52 | PIK3CA c.1624G>A p.Glu542Lys, TP53 c.1024C>T p.Arg342Ter | PIK3CA;2C.1 | TP53;2C.2 | 2C.1 |
| 610 | PROSTATE CANCER | male | 67 | ATM c.1402_1403delAA (p.Lys468GlufsTer18) | ATM;1A.1 |  | 1A.1 |

Additional file 4: Figure S1. Pancreatic cancer patients' categorization based on TIER classification of their most clinically significant variant.

A. Pancreatic cancer patients' categorization based on TIER classification of their most clinically significant variant. Patients were categorized in the following categories: No Biomarker: Patients with no biomarker available, 1B: Patients harboring biomarkers with strong evidence of correlation to treatment, 2C.1 *KRAS*: Patients with a single finding in the *KRAS* gene, 2C.1: Patients with biomarkers related to off-label treatment.

B. Percentage of patients with On-label and off-label mutations identified and the type of alterations detected. Genes of the homologous recombination complex are labeled in blue.


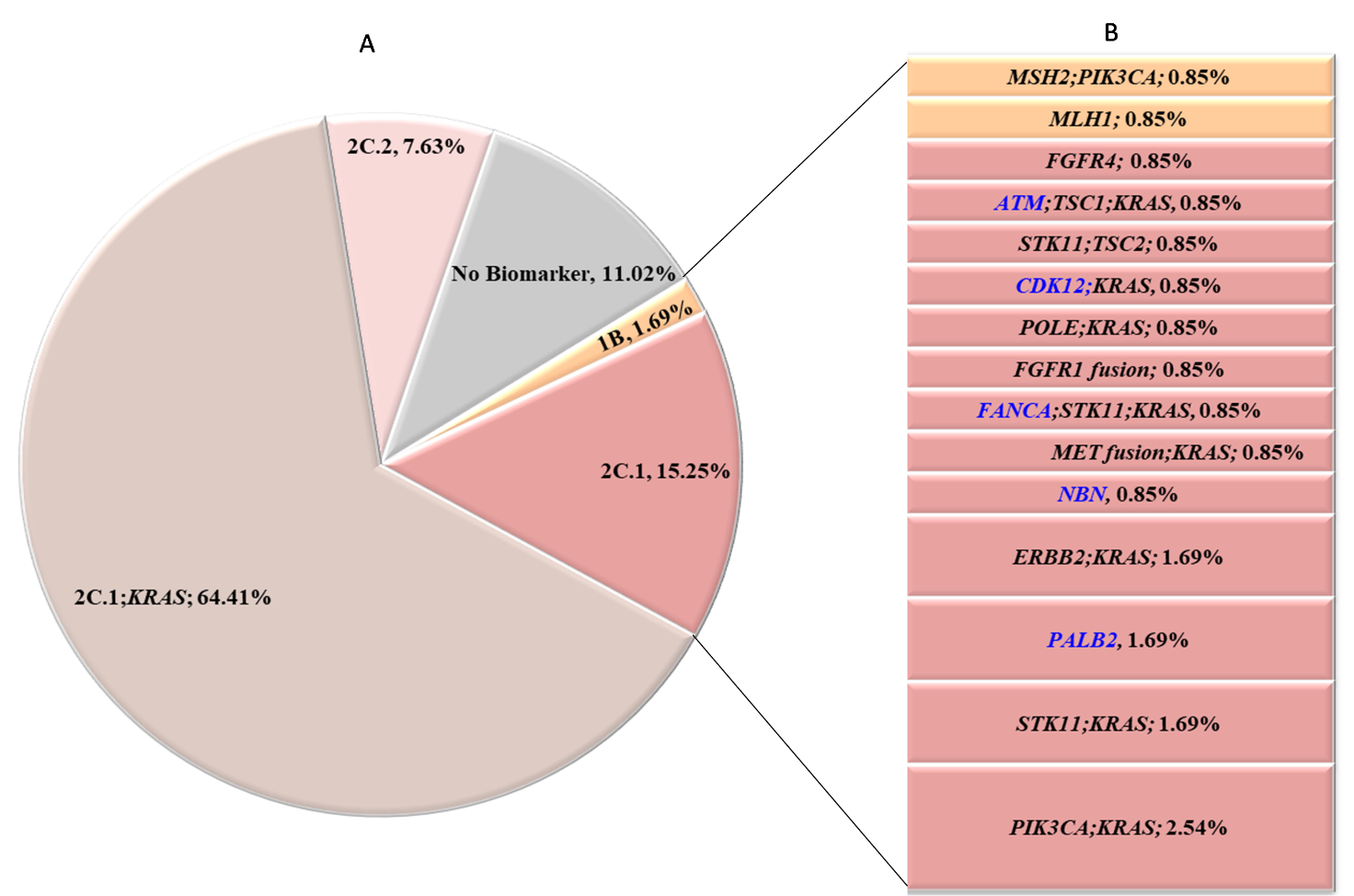


Additional file 5: Figure S2. Lung cancer patients' categorization based on TIER classification.

A. Lung cancer patients' categorization based on TIER classification of their most clinically significant variant. The following categories were used:, 1A.1: Patients with biomarkers related to on-label treatment, 1B: Patients harboring biomarkers with strong evidence of correlation to treatment, 2C.1: Patients with biomarkers related to off-label treatment, 1A.2R; 2C.1: Patients harboring a *KRAS* mutation related to resistance to treatment plus an off-label, 1A.2R: Patients harboring a *KRAS* mutation related to EGFR TKIs resistance, 2.C.2: Patients B. % of patients with On-label and off-label mutations identified and the type of alterations detected. Genes of the homologous recombination complex are labeled in blue.


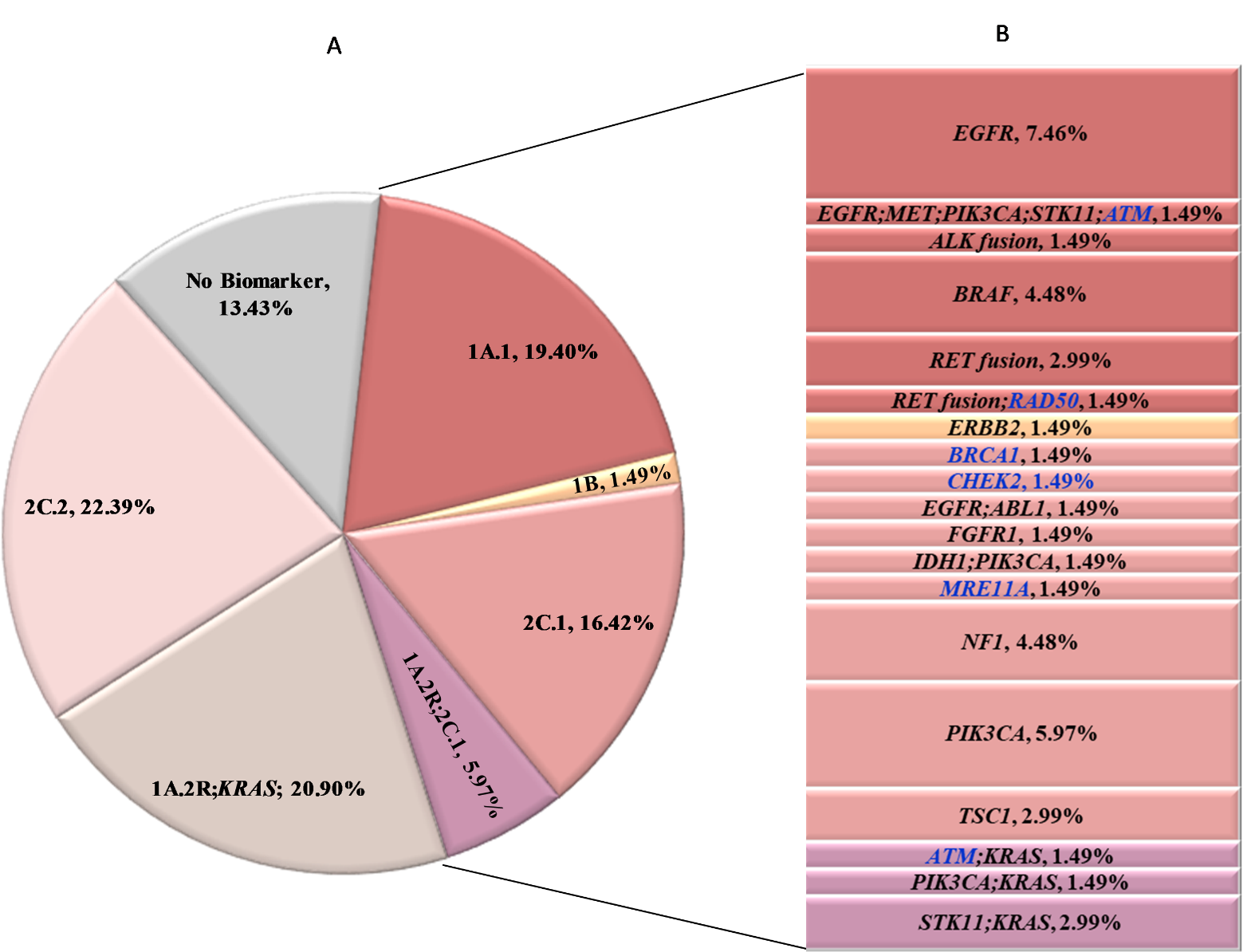


Additional file 6: Figure S3. Breast cancer patients' categorization based on the TIER classification.

A. Patients' categorization based on TIER classification of their most clinically significant variant. The following categories were used: No Biomarker: Patients with no biomarker available, 1A.1: Patients with biomarkers related to on-label treatment, 2C.1: Patients with biomarkers related to off-label treatment, 2C.2: Patients with biomarkers related to clinical trials, 2D: Patients with biomarkers with preclinical evidence.

B. Percentage of patients with On-label and off-label mutations identified and the type of alterations detected. Genes of the homologous recombination complex are labeled in blue.


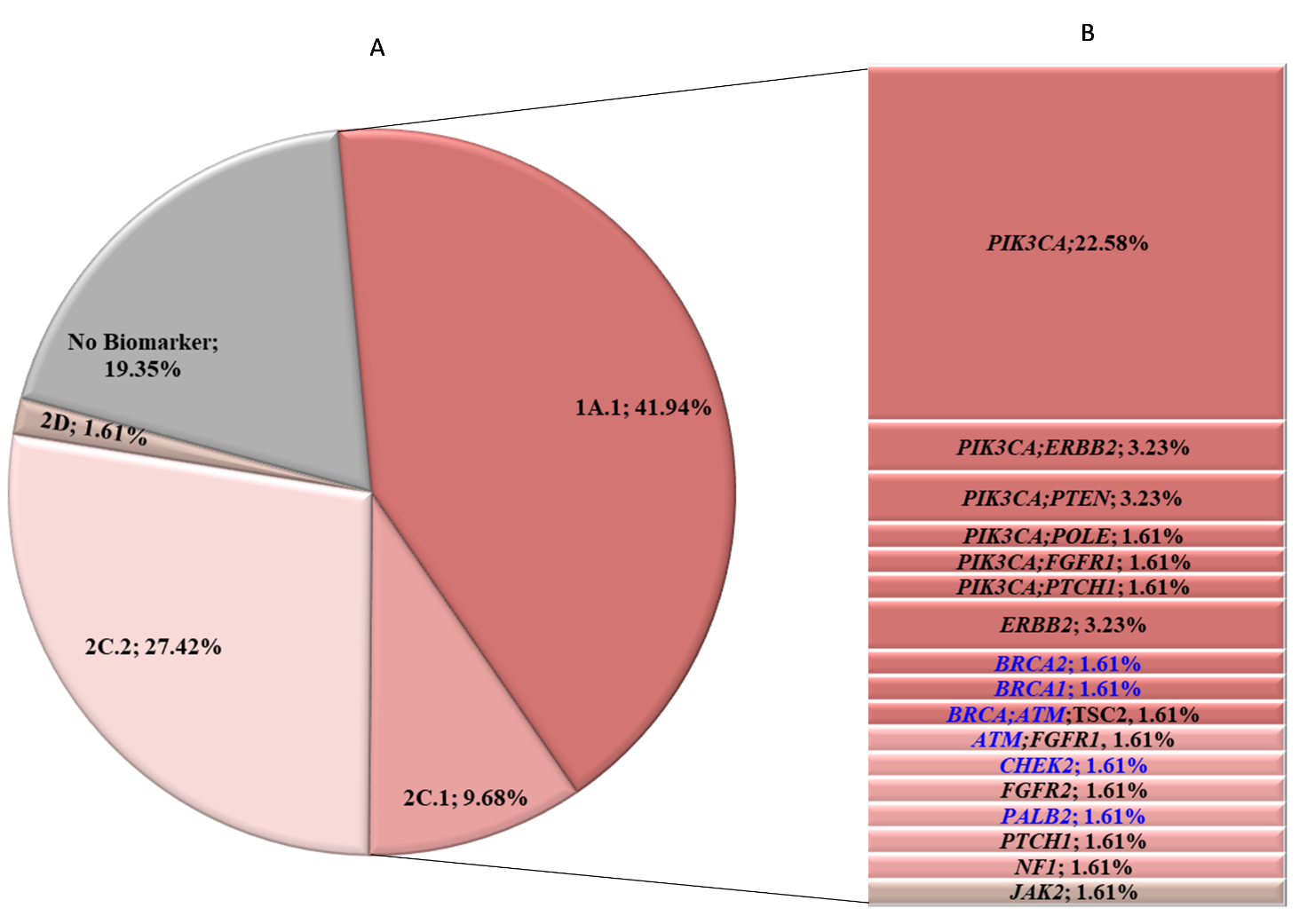


Additional file 7: Figure S4. Colorectal cancer patients' categorization based on TIER classification.

A. Patients' categorization based on TIER classification of their most clinically significant variant. Patients were categorized in the following categories: No Mutation,1A.1: Patients with no mutation in *KRAS*/NRAS genes with or without other variations, 1A.2: Patients with biomarkers included in professional guidelines, 1A.1R: Patients harboring either *KRAS* or NRAS mutations related to resistance to treatment

B. % of patients with on-label and off-label mutations identified and the type of alterations detected.


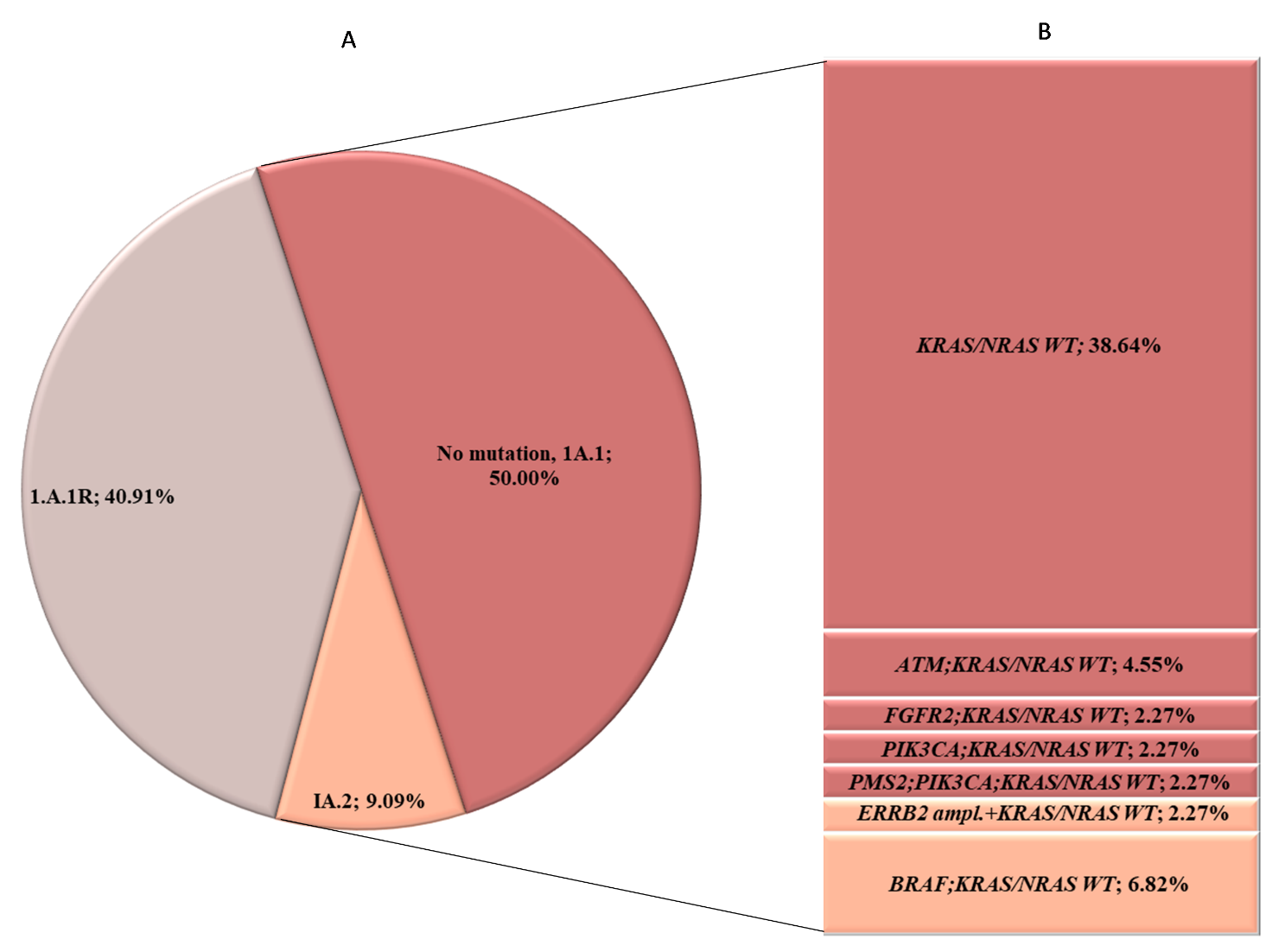


Additional file 4: Table S4. Biomarker's summary in the 610 patients included in the study

| **Tumor Type** | **Percentage of patients with Tier1 variants** | **Percentage of patients with 2C.1 variants** | **Percentage of patients with 2C.2/2D variants** | **Percentage of patients with Tier 3 variants** | **Percentage of patients with TMB>10muts/MB** | **MEDIAN TMB** | **Percentage of PD-L1 positive patients** | **Percentage MSI-High patients** |
| --- | --- | --- | --- | --- | --- | --- | --- | --- |
| Biliary | 0% (0/25) | 44.00% (11/25) | 28.00% (7/25) | 0% (0/25) | 23.08% (3/13) | 4.24 | 28.57% (2/7) | 0% (0/7) |
| Brain | 0% (0/18) | 72.22% (13/18) | 11.11% (2/18) | 0% (0/18) | 28.57% (4/14) | 6.97 | 16.67% (1/6) | 0% (0/6) |
| Breast Cancer | 41.94% (26/62) | 9.68% (6/62) | 29.03% (18/62) | 0% (0/62) | 29.41% (10/34) | 4.62 | 11.76% (2/17) | 0% (0/17) |
| Colon/Rectum | 52.27% (23/44) | 9.09% (4/44) | 20.45% (9/44) | 2.27% (1/44) | 34.37% (11/32) | 8.02 | 26.32% (5/19) | 5.26% (1/19) |
| Endometrium | 0% (0/18) | 55.56% (10/18) | 38.89% (7/18) | 0% (0/18) | 10.00% (1/10) | 3.83 | 0% (0/5) | 0% (0/2) |
| Esophagous | 0% (0/8) | 25.00% (2/8 ) | 50.00% (4/8) | 0% (0/8) | 20% (1/5) | 5.10 | Not Done | 0% (0/3) |
| Gastric | 5.71% (2/35) | 17.14% (6/35) | 37.14% (13/35) | 2/35 5.71% | 33.33% (7/21) | 7.99 | 40.00% (4/10) | 30% (3/10) |
| Hepatocellular | 0% (0/10) |  | 10.00% (4/40) | 1/10 10.00% | 40.00% (2/5) | 7.58 | Not Done | Not Done |
| Lung Cancer | 47.76% (32/67) | 22.39% (15/67) | 16.42% (11/67) | 0% (0/67) | 42.55% (20/47) | 7.72 | 69.23% (18/26) | 0% (0/27) |
| Ovarian Cancer | 12.12% (4/33) | 30.30% (10/33) | 42.42% (14/33) | 3.03% (1/33) | 15.79% (3/19) | 4.44 | 4/11 36.36% | 9.09% (1/11) |
| Pancreatic Cancer | 1.69% (2/118) | 79.66% (94/118) | 7.63% (9/118) | 0% (0/118) | 19.40% (13/67) | 4.63 | 50.00% (14/28 ) | 1/29(3.45%) |
| Prostate Cancer | 20% (7/35) | 14.29% (5/35) | 37.14% (13/35) | 2.86% (1/35) | 22.22% (4/18) | 5.92 | 11.11% (1/9) | 12.50% (1/8) |
| Soft Tissue | 0% (0/34) | 35.29% (12/34) | 11.76% (4/35) | 0% (0/34) | 3.70% (1/27) | 3.43 | 27.89% (5/18) | 5.56% (1/19) |
| Unknown Primary | 0% (0/19) | 31.58% (6/19) | 31.58% (6/19) | 0% (0/19) | 50% (3/6) | 11.46 | 66.67% (2/3) | Not Done |
| All tumors | 19.67% (120/610) | 35.08% (214/610) | 23.11% (141/610) | 0.66% (4/610) | 25.09% (96/381) | 5.60 | 38.89% (77/198) | 3.88% (8/206) |

Additional file 9: Table S5 Alterations that would have been detected if two hotspot panels of 24 and 50 genes respectively, had been used in the 610 patients analyzed.

| **Patient** | **Tumor Type** | **24 gene panel** | **CNAS-24** | **Fusions-24** | **50 gene panel** | **CNAS-50** | **Fusions-50** | **Higher Tier Classification using the 24 gene panel** | **Higher Tier Classification of the variants detected using the 50 gene panel** |
| --- | --- | --- | --- | --- | --- | --- | --- | --- | --- |
| 1 | prostate | TP53 c.644G>A (p.S215N) | - | - | TP53 c.644G>A (p.S215N) |  | - | 2C.2 | 2C.2 |
| 2 | endometrium | TP53 c.706T>C (p.Y236H), FBXW7 c.1394G>A (p.R465H ) | - | - | TP53 c.706T>C (p.Y236H), FBXW7 c.1394G>A (p.R465H ) |  | - | 2C.2 | 2C.2 |
| 3 | breast |  | - | - |  |  | - | normal | normal |
| 4 | breast | TP53 c.266_267delCCinsTG (p.P89L) | - | - | TP53 c.266_267delCCinsTG (p.P89L) |  | - | 2C.2 | 2C.2 |
| 5 | Pancreatic cancer | KRAS c.35G>A (p.G12D) | - | - | KRAS c.35G>A (p.G12D) |  | - | 2C.1 | 2C.1 |
| 6 | Endometrium | TP53 c.557A>G (p.D186G) | MET Amplification | - | TP53 c.557A>G (p.D186G) | MET Amplification | - | 2C.1 | 2C.1 |
| 7 | Lung | KRAS c.35G>A (p.G12D), TP53 c.637C>T (p.R213*) | - | - | KRAS c.35G>A (p.G12D), TP53 c.637C>T (p.R213*) |  | - | 1A.2R | 1A.2R |
| 8 | Lung | NRAS c.182A>G (p.Q61R), TP53 c.524G>A (p.R175H) | - | - | NRAS c.182A>G (p.Q61R), TP53 c.524G>A (p.R175H) |  | - | 2C.1 | 2C.1 |
| 9 | uterus | TP53 c.314G>T (p.G105V) | - | - | TP53 c.314G>T (p.G105V) |  | - | 2C.2 | 2C.2 |
| 10 | ovarian |  | - | - |  |  | - | normal | normal |
| 11 | Lung | EGFR c.2235_2252delGGAATTA (p.E746_T751delinsFPI), CTNNB1 c.134C>T (p.S45F) | - | - | EGFR c.2235_2252delGGAATTA (p.E746_T751delinsFPI), CTNNB1 c.134C>T (p.S45F) |  | - | 1A.1 | 1A.1 |
| 12 | kidney cancer |  | - | - |  |  | - | normal | normal |
| 13 | Colon cancer | TP53 c.799C>T (p.R267W), TP53 c.916C>T (p.R306*), TP53 c.691A>G (p.T231A) | - | - | TP53 c.799C>T (p.R267W), TP53 c.691A>G (p.T231A) |  | - | 1A.1 | 1A.1 |
| 14 | Colon cancer |  | - | - |  |  | - | 1A.1 | 1A.1 |
| 15 | Prostate cancer |  | - | - |  |  | - | normal | normal |
| 16 | Pancreatic cancer cancer |  | - | - |  |  | - | normal | normal |
| 17 | invasive breast cancer | TP53 c.524G>A (p.R175H) | - | - | TP53 c.524G>A (p.R175H) |  | - | 2C.2 | 2C.2 |
| 18 | Pancreatic cancer | KRAS c.35G>A (p.G12D), TP53 c.818G>A (p.R273H) | - | - | KRAS c.35G>A (p.G12D), TP53 c.818G>A (p.R273H) |  | - | 2C.1 | 2C.1 |
| 19 | colon |  | - | - |  |  | - | 1A.1 | 1A.1 |
| 20 | colon | TP53 c.841_842dupGA (p.D281fs*65) | - | - | TP53 c.841_842dupGA (p.D281fs*65) |  | - | 1A.1 | 1A.1 |
| 21 | Pancreatic cancer |  | - | - |  |  | - | 2C.2 | 2C.2 |
| 22 | Pancreatic |  | - | - |  |  | - | normal | normal |
| 23 | Lung cancer | EGFR exon 19 c.2239_2251delTTAAGAGAAGCAAinsC (p.L747_T751delinsP), EGFR c.2369C>T (p.T790M), EGFR c.2390G>C (p.C797S), MET c.3335A>G (p.H1112R), PIK3CA c.1630A>G (p.T544A) | - | - |  |  | - | 1A.1 | 1A.1 |
| 24 | Liver Cancer (Cholangiocarcinoma) |  | - | - | IDH1 c.394C>T (p.R132C) |  | - | normal | 2C.1 |
| 25 | colon |  | - | - |  |  | - | 1A.1 | 1A.1 |
| 26 | unknown | TP53 c.1024C>T (p.R342*) | - | - | TP53 c.1024C>T (p.R342*) |  | - | 2C.2 | 2C.2 |
| 27 | lung |  | - | - |  |  | - | normal | normal |
| 28 | Breast |  | - | - |  |  | - | normal | normal |
| 29 | ovarian | KRAS c.34G>T (p.G12C), TP53 c.747G>T (p.R249S) | - | - | KRAS c.34G>T (p.G12C), TP53 c.747G>T (p.R249S) |  | - | 2C.1 | 2C.1 |
| 30 | Pancreatic cancer |  | - | - |  |  | - | normal | normal |
| 31 | Uknown |  | - | - |  |  | - | normal | normal |
| 32 | Uknown |  | - | - |  |  | - | normal | normal |
| 33 | Pancreatic cancer | KRAS c.35G>A (p.G12D), TP53 c.796G>A (p.G266R) |  | - | KRAS c.35G>A (p.G12D), TP53 c.796G>A (p.G266R) |  | - | 2C.1 | 2C.1 |
| 34 | Endometrium |  | - | - |  |  | - | normal | normal |
| 35 | lung | KRAS c.34G>T (p.G12C), TP53 c.747G>T (p.R249S) | - | - | KRAS c.34G>T (p.G12C), TP53 c.747G>T (p.R249S) |  | - | 1A.2R | 1A.2R |
| 36 | lung |  | - | - |  |  | - | normal | normal |
| 37 | ovary | TP53 c.796G>A (p.Gly266Glu) | - | - | TP53 c.796G>A (p.Gly266Glu) |  | - | 2C.2 | 2C.2 |
| 38 | breast | TP53 c.329G>C (p.R110P) | FGFR2 Amplification | - | TP53 c.329G>C (p.R110P) | FGFR2 Amplification | - | 2C.1 | 2C.1 |
| 39 | ovary | TP53 c.1006G>T (p.E336*),TP53 c.772G>A (p.E258K) |  | - | TP53 c.1006G>T (p.E336*),TP53 c.772G>A (p.E258K) |  | - | 2C.2 | 2C.2 |
| 40 | breast | BRAF c.1406G>C (p.G469A), PIK3CA c.3140A>G (p.H1047R), TP53 c.839G>C (p.R280T) |  | - | BRAF c.1406G>C (p.G469A), PIK3CA c.3140A>G (p.H1047R), TP53 c.839G>C (p.R280T) |  | - | 1A.1 | 1A.1 |
| 41 | breast | PIK3CA c.1633G>A (p.Ε545Κ) |  | - | PIK3CA c.1633G>A (p.Ε545Κ) | - | - | 1A.1 | 1A.1 |
| 42 | breast | TP53 c.637C>T (p.Arg213Ter) | PIK3CA Amplification | - | TP53 c.637C>T (p.Arg213Ter) | PIK3CA Amplification | - | 2C.2 | 2C.2 |
| 43 | breast | PIK3CA c.3140A>G (p.His1047Arg), TP53 c.818G>A (p.Arg273His) | ERBB2 Amplification | - | PIK3CA c.3140A>G (p.His1047Arg), TP53 c.818G>A (p.Arg273His) | ERBB2 Amplification | - | 1A.1 | 1A.1 |
| 44 | lung | EGFR c.2303G>T (p.S768I), TP53 c.764_765delTCinsAT (p.I255N) |  | - | EGFR c.2303G>T (p.S768I), TP53 c.764_765delTCinsAT (p.I255N) | - | - | 2C.1 | 2C.1 |
| 45 | cervical | KRAS c.34 G>T (p.G12C) | - | - | KRAS c.34 G>T (p.G12C) | - | - | 2C.1 | 2C.1 |
| 46 | gastric |  | - | - |  | - | - | normal | normal |
| 47 | serous carcinoma | KRAS c.38 G>A (p.G13D) | - | - | KRAS c.38 G>A (p.G13D), PIK3CA c.2702G>T (p.C901F) | - | - | 2C.1 | 2C.1 |
| 48 | breast | TP53 c.818G>A (p.Arg273His) | - | - |  | - | - | 2C.2 | 2C.2 |
| 49 | liver cancer | CTNNB1 c.101G>T p.G34V | - | - |  | - | - | 2C.2 | 2C.2 |
| 50 | lung |  | - | RET, KIF5B(15)-RET(12) |  | - | RET, KIF5B(15)-RET(12) | 1A.1 | 1A.1 |
| 51 | ovarian |  | - | - |  | - | - | normal | normal |
| 52 | Unknown |  | - | - |  | - | - | normal | normal |
| 53 | oesophagus | TP53 c.743G>A (p.Arg248Gln) | - | - | TP53 c.743G>A (p.Arg248Gln) | - | - | 2C.2 | 2C.2 |
| 54 | lung | EGFR c.2237_2255del19insT (p.Glu746_Ser752delinsVal), TP53 c.853G>A (p.Glu285Lys) | - | - | EGFR c.2237_2255del19insT (p.Glu746_Ser752delinsVal), TP53 c.853G>A (p.Glu285Lys), APC c.3920T>A (p.Ile1307Lys) | - | - | 1A.1 | 1A.1 |
| 55 | Pancreatic cancer |  |  | - |  | - | - | normal | normal |
| 56 | breast | PIK3CA c.1633G>A (p.Glu545Lys), TP53 c.817C>T (p.Arg273Cys) | - | - | PIK3CA c.1633G>A (p.Glu545Lys), TP53 c.817C>T (p.Arg273Cys) | - | - | 1A.1 | 1A.1 |
| 57 | sarcoma? |  | - | - |  | - | - | normal | normal |
| 58 | Pancreatic cancer | KRAS c.34G>C (p.Gly12Arg), TP53 c.817C>T (p.Arg273Cys) | - | - | KRAS c.34G>C (p.Gly12Arg), TP53 c.817C>T (p.Arg273Cys) | - | - | 2C.1 | 2C.1 |
| 59 | Pancreatic cancer | TP53 c.817C>T (p.Arg273Cys) | - | - | TP53 c.817C>T (p.Arg273Cys) | - | - | 2C.2 | 2C.2 |
| 60 | Vaginal Cancer |  | - | - |  | - | - | normal | normal |
| 61 | gastric | TP53 c.817C>T (p.Arg273Cys) | - | - | TP53 c.817C>T (p.Arg273Cys) | - | - | 2C.2 | 2C.2 |
| 62 | Cholangiocarcinoma |  | - | - |  | - | - | normal | normal |
| 63 | gastric |  | - | - |  | - | - | normal | normal |
| 64 | lung |  | - | RET, KIF5B(15)-RET(12) |  | - | RET, KIF5B(15)-RET(12) | 1A.1 | 1A.1 |
| 65 | cervical |  | - | - |  | - | - | normal | normal |
| 66 | breast | TP53 c.701A>G p.Y234C | FGFR1 Amplification | - | TP53 c.701A>G p.Y234C | FGFR1 Amplification | - | 2C.1 | 2C.1 |
| 67 | oesophagus |  | - | - |  | - | - | normal | normal |
| 68 | gastric | KRAS c.35G>A (p.G12D) | - | - |  | - | - | 2C.1 | 2C.1 |
| 69 | OVARIAN NEOPLASM, LYMPHNODES AND INTESTINAL METASTASIS | TP53 c.707A>G (p.Y236C) | - | - | TP53 c.707A>G (p.Y236C) | - | - | 2C.2 | 2C.2 |
| 70 | Pancreatic cancer | TP53 c.557A>G (p.D186G) | - | - | TP53 c.557A>G (p.D186G) | - | - | 2C.2 | 2C.2 |
| 71 | Leiomyosarcoma |  | - | - |  | - | - | normal | normal |
| 72 | Pancreatic cancer | KRAS c.35G>A (p.Gly12Asp), TP53 c.215_216insG (p.Val73fs) | - | - | KRAS c.35G>A (p.Gly12Asp), TP53 c.215_216insG (p.Val73fs) | - | - | 2C.1 | 2C.1 |
| 73 | Unknown |  | - | - |  | - | - | normal | normal |
| 74 | Breast | TP53 c.256_257delGC (p.Ala86fs) | - | - | TP53 c.256_257delGC (p.Ala86fs) | - | - | 2C.2 | 2C.2 |
| 75 | HEPATOCELLULAR CARCINOMA METASTASIS | TP53 c.313G>T (p.Gly105Cys) | - | - | TP53 c.313G>T (p.Gly105Cys) | - | - | 2C.2 | 2C.2 |
| 76 | Breast |  | - | - |  | - | - | normal | normal |
| 77 | Lung | KRAS c.34G>T (p.Gly12Cys), TP53 c.637C>T (p.Arg213Ter) | - | - | KRAS c.34G>T (p.Gly12Cys), TP53 c.637C>T (p.Arg213Ter) | - | - | 1A.2R | 1A.2R |
| 78 | Unknown Primary | AKT1 c.49G>A (p.Glu17Lys) | - | - | AKT1 c.49G>A (p.Glu17Lys) | - | - | 2C.2 | 2C.2 |
| 79 | colon LIVER METASTASIS | KRAS c.183A>C (p.Gln61His), PIK3CA c.1625A>T (p.Glu542Val), PIK3CA c.3145G>C (p.Gly1049Arg), GNAS c.2531G>A (p.Arg844His) | - | - | KRAS c.183A>C (p.Gln61His), PIK3CA c.1625A>T (p.Glu542Val), PIK3CA c.3145G>C (p.Gly1049Arg), GNAS c.2531G>A (p.Arg844His) | - | - | 1A.1R | 1A.1R |
| 80 | Pancreatic cancer | KRAS c.35G>A (p.Gly12Asp), TP53 c.430C>T (p.Gln144Ter) | - | - | KRAS c.35G>A (p.Gly12Asp), TP53 c.430C>T (p.Gln144Ter) | - | - | 2C.1 | 2C.1 |
| 81 | colon | KRAS c.38G>A (p.G13D), TP53 c.844C>T (p.R282W) | - | - | KRAS c.38G>A (p.G13D), TP53 c.844C>T (p.R282W) | - | - | 1A.1R | 1A.1R |
| 82 | parotid gland |  | - |  |  | - |  | normal | normal |
| 83 | ovarian cancer | TP53 c.228_229insG (p.Pro77fs) | - | - | TP53 c.228_229insG (p.Pro77fs) | - | - | 2C.2 | 2C.2 |
| 84 | HEPATOCELLULAR |  | - | - |  | - | - | normal | normal |
| 85 | Pancreatic cancer | KRAS c.35G>A (p.Gly12Asp) | - | - | KRAS c.35G>A (p.Gly12Asp) | - | - | 2C.1 | 2C.1 |
| 86 | HEPATOCELLULAR CARCINOMA and CHOLANGIO |  | - | - |  | - | - | normal | normal |
| 87 | Malignant Peripheral Nerve Sheath Tumors (MPNST) |  | - | - |  | - | - | normal | normal |
| 88 | prostate |  | - | - |  | - | - | normal | normal |
| 89 | colon | TP53 c.844C>T (p.R282W) | - | - | PTPN11 c.205G>A (p.E69K), TP53 c.844C>T (p.R282W) | - | - | 1A.1 | 1A.1 |
| 90 | hepatocellular | TP53 c.527G>A (p.C176Y) | - | - | TP53 c.527G>A (p.C176Y) | - | - | 2C.2 | 2C.2 |
| 91 | lung | PIK3CA c.1666C>T (p.His556Tyr), TP53c.493C>T (p.Gln165Ter) | - | - | PIK3CA c.1666C>T (p.His556Tyr), TP53c.493C>T (p.Gln165Ter) | - | - | 2C.1 | 2C.1 |
| 92 | colon | BRAF c.1781A>G (p.D594G) | - | - | BRAF c.1781A>G (p.D594G) | - | - | 1A.1 | 1A.1 |
| 93 | lung | KRAS c.35G>A (p.Gly12Asp) | - | - | KRAS c.35G>A (p.Gly12Asp) | - | - | 1A.2R | 1A.2R |
| 94 | pancreatic cancer | KRAS c.35G>T (p.Gly12Val), TP53 c.532C>T (p.His178Tyr) | - | - | KRAS c.35G>T (p.Gly12Val), TP53 c.532C>T (p.His178Tyr), ATM c.9022C>T (p.Arg3008Cys) | - | - | 2C.1 | 2C.1 |
| 95 | lung | KRAS c.35G>A (p.Gly12Asp), TP53 c.818G>T (p.Arg273Leu) | - | - | KRAS c.35G>A (p.Gly12Asp), TP53 c.818G>T (p.Arg273Leu) | - | - | 1A.2R | 1A.2R |
| 96 | Sarcoma |  | - | - |  | - | - | normal | normal |
| 97 | Cholangiocarcinoma |  | - | - |  | - | - | normal | normal |
| 98 | lung | KRAS c.35G>A (p.Gly12Asp) | - | - | KRAS c.35G>A (p.Gly12Asp) | - | - | 1A.2R | 1A.2R |
| 99 | pancreatic cancer | KRAS c.35G>T (p.Gly12Val) | - | - | KRAS c.35G>T (p.Gly12Val) | - | - | 2C.1 | 2C.1 |
| 100 | Breast | - | - | - | - | - | - | normal | normal |
| 101 | Prostate | CTNNB1 c.134C>T (p.Ser45Phe) | - | - | CTNNB1 c.134C>T (p.Ser45Phe) | - | - | 2C.2 | 2C.2 |
| 102 | Gastric | TP53 c.919+1G>T |  | - | TP53 c.919+1G>T | - | - | 2C.2 | 2C.2 |
| 103 | Pancreatic cancer | KRAS c.35G>T (p.Gly12Val) | - | - | KRAS c.35G>T (p.Gly12Val) | - | - | 2C.1 | 2C.1 |
| 104 | Mouth Cancer | TP53 c.524G>A (p.R175H), TP53 c.742C>T (p.R248W) | - | - | TP53 c.524G>A (p.R175H), TP53 c.742C>T (p.R248W) | - | - | 2C.2 | 2C.2 |
| 105 | Pancreatic cancer |  | - | - |  | - | - | normal | normal |
| 106 | breast | PIK3CA c.1357G>A (p.E453K), PTEN c.733C>T (p.Q245*) | - | - |  | - | - | 1A.1 | 1A.1 |
| 107 | Sarcoma | TP53 c.524G>A (p.R175H) | FGFR1 Amplification | - | TP53 c.524G>A (p.R175H) | FGFR1 Amplification | - | 2C.1 | 2C.1 |
| 108 | Breast |  | - | - |  |  | - | normal | normal |
| 109 |  | TP53 c.586C>T (p.Arg196Ter) | PIK3CA Amplification, KRAS Amplification | - | TP53 c.586C>T (p.Arg196Ter) | PIK3CA Amplification, KRAS Amplification | - | 2C.2 | 2C.2 |
| 110 | Sarcoma |  | - | - |  | - | - | normal | normal |
| 111 | Sarcoma |  | - | - |  | - | - | normal | normal |
| 112 | colon | PIK3CA c.1634A>C (p.Glu545Ala), PIK3CA c.3129G>T (p.Met1043Ile) | - | - | PIK3CA c.1634A>C (p.Glu545Ala), PIK3CA c.3129G>T (p.Met1043Ile) | - | - | 1A.1 | 1A.1 |
| 113 | Sarcoma |  | - | - |  | - | - | normal | normal |
| 114 |  |  | - | - |  | - | - | normal | normal |
| 115 | ovarian |  | - | - |  | - | - | normal | normal |
| 116 | Lung | PIK3CA c.1624G>A (p.Glu542Lys), TP53 c.469G>T (p.Val157Phe) | - | - | PIK3CA c.1624G>A (p.Glu542Lys), TP53 c.469G>T (p.Val157Phe) | - | - | 2C.1 | 2C.1 |
| 117 | Gastric | TP53 c.733G>A (p.Gly245Ser) | - | - | TP53 c.733G>A (p.Gly245Ser) | - | - | 2C.2 | 2C.2 |
| 118 | Malignant neoplasm |  | - | - | PDGFRA c.2525A>T (p.D842V) | - | - | normal | 2C.1 |
| 119 | Breast |  | - | - |  | - | - | normal | normal |
| 120 | Adenocortical |  | - | - |  | - | - | normal | normal |
| 121 | Prostate |  | - | - |  | - | - | normal | normal |
| 122 | Lung | KRAS c.34G>T (p.G12C) | - | - | KRAS c.34G>T (p.G12C) | - | - | 1A.2R | 1A.2R |
| 123 | Gastric |  | - | - |  | - | - | normal | normal |
| 124 | Oesophageal | KRAS c.35G>C (p.Gly12Ala), TP53 c.527G>A (p.Cys176Tyr) | - | - | KRAS c.35G>C (p.Gly12Ala), TP53 c.527G>A (p.Cys176Tyr) | - | - | 2C.1 | 2C.1 |
| 125 | Ovarian |  | - | - |  | - | - | normal | normal |
| 126 | Lung | KRAS c.183A>T (p.Gln61His) | - | - | KRAS c.183A>T (p.Gln61His) | - | - | 1A.2R | 1A.2R |
| 127 | Breast | TP53 c.524G>A (p.Arg175His) | - | - | TP53 c.524G>A (p.Arg175His) | - | - | 2C.2 | 2C.2 |
| 128 | Ovarian | KRAS c.35G>A (p.Gly12Asp), PIK3CA c.1634A>C (p.Glu545Ala) | - | - | KRAS c.35G>A (p.Gly12Asp), PIK3CA c.1634A>C (p.Glu545Ala) | - | - | 2C.1 | 2C.1 |
| 129 | Lung | TP53 c.731delG (p.Gly244fs) | - | - | TP53 c.731delG (p.Gly244fs) | - | - | 2C.2 | 2C.2 |
| 130 | Cholangiocarcinoma | KRAS c.35G>A (p.Gly12Asp), TP53 c.1024C>T (p.Arg342Ter) | - | - | KRAS c.35G>A (p.Gly12Asp), TP53 c.1024C>T (p.Arg342Ter) | - | - | 2C.1 | 2C.1 |
| 131 | Brain |  | - | - | EGFR c.866C>T (p.Ala289Val) | - | - | normal | 2C.1 |
| 132 | Liver |  | - | - |  | - | - | normal | normal |
| 133 | Lung |  | - | - |  | - | - | 2C.1 | 2C.1 |
| 134 | Colon | KRAS c.35G>A (p.Gly12Asp), PIK3CA c.3140A>G (p.H1047R), TP53 c.584T>C (p.I195T) | - | - | KRAS c.35G>A (p.Gly12Asp), PIK3CA c.3140A>G (p.H1047R), TP53 c.584T>C (p.I195T) | - | - | 1A.1R | 1A.1R |
| 135 | Ovarian |  | - | - |  | - | - | normal | normal |
| 136 | Penile | TP53 c.733G>A (p.G245S) | - | - | CDKN2A c.238C>T (p.R80*), TP53 c.733G>A (p.G245S) | - | - | 2C.2 | 2C.2 |
| 137 | Brain | TP53 c.524G>A (p.Arg175His) | - | - | TP53 c.524G>A (p.Arg175His) | - | - | 2C.2 | 2C.2 |
| 138 | Ovarian | TP53 c.584T>C (p.Ile195Thr) | - | - | TP53 c.584T>C (p.Ile195Thr) | - | - | 2C.2 | 2C.2 |
| 139 | Pancreatic cancer | KRAS c.35G>A (p.Gly12Asp), | - | - | KRAS c.35G>A (p.Gly12Asp), | - | - | 2C.1 | 2C.1 |
| 140 | Pancreatic cancer | KRAS c.35G>T (p.Gly12Val) | - | - | KRAS c.35G>T (p.Gly12Val) | - | - | 2C.1 | 2C.1 |
| 141 | Liver | KRAS c.181_182insTTCTCGACACAGCAGGTC | - | - | KRAS c.181_182insTTCTCGACACAGCAGGTC | - | - | 2C.1 | 2C.1 |
| 142 | Pancreatic cancer | KRAS c.35G>A (p.Gly12Asp) | - | - | KRAS c.35G>A (p.Gly12Asp) | - | - | 2C.1 | 2C.1 |
| 143 | Pancreatic cancer | KRAS c.35G>A (p.Gly12Asp), TP53 c.706T>A (p.Tyr236Asn) | - | - | KRAS c.35G>A (p.Gly12Asp), TP53 c.706T>A (p.Tyr236Asn) | - | - | 2C.1 | 2C.1 |
| 144 | Pancreatic cancer | KRAS c.35G>T (p.Gly12Val) | - | - | KRAS c.35G>T (p.Gly12Val) | - | - | 2C.1 | 2C.1 |
| 145 | Extra adrenal paraganglioma |  | - | - |  | - | - | 2C.1 | 2C.1 |
| 146 | Serous | TP53 c.747_753delGCCCATC (p.Arg249fs) | - | - | TP53 c.747_753delGCCCATC (p.Arg249fs) | - | - | normal | normal |
| 147 | Lacrimal gland | TP53 c.451C>T (p.Pro151Ser) | - | - | TP53 c.451C>T (p.Pro151Ser) | - | - | 2C.2 | 2C.2 |
| 148 | Vulvar | TP53 c.568_569insT (p.Pro190fs) | - | - | CDKN2A c.358G>T (p.Glu120Ter), c.172C>T (p.Arg58Ter), TP53 c.568_569insT (p.Pro190fs) | - | - | 2C.2 | 2C.2 |
| 149 | Gastric | TP53 c.807_837delCTTTGAGGTGCGTGTTTGTGCCTGTCCTGGG (p.Ser269fs) | - | - | TP53 c.807_837delCTTTGAGGTGCGTGTTTGTGCCTGTCCTGGG (p.Ser269fs) | - | - | 2C.2 | 2C.2 |
| 150 | Pancreatic cancer | TP53 c.743G>A (p.Arg248Gln) | - | - | TP53 c.743G>A (p.Arg248Gln) | - | - | 2C.2 | 2C.2 |
| 151 | Pancreatic cancer | KRAS c.35G>A (p.Gly12Asp) | - | - | KRAS c.35G>A (p.Gly12Asp) | - | - | 2C.1 | 2C.1 |
| 152 | Rectal |  | - | - |  | - | - | normal | normal |
| 153 | Lung |  | - | - |  | - | - | normal | normal |
| 154 | Pancreatic cancer | KRAS c.35G>A (p.Gly12Asp), TP53 c.823delT (p.Cys275fs) | - | - | KRAS c.35G>A (p.Gly12Asp), TP53 c.823delT (p.Cys275fs) | - | - | 2C.1 | 2C.1 |
| 155 | Alveolar Soft tissue Sarcoma | TP53 c.841G>A (p.Asp281Asn) | - | - | TP53 c.841G>A (p.Asp281Asn) | - | - | 2C.1 | 2C.1 |
| 156 | Gastr╬┐esophageal | BRAF c.1781A>C (p.Asp594Ala),TP53 c.524G>A (p.Arg175His) | KRAS Amplification | - | BRAF c.1781A>C (p.Asp594Ala),TP53 c.524G>A (p.Arg175His) | KRAS Amplification | - | 2C.2 | 2C.2 |
| 157 | Colon | KRAS c.38G>A (p.Gly13Asp) | - | - | KRAS c.38G>A (p.Gly13Asp) | - | - | 1A.1R | 1A.1R |
| 158 | Thyroid |  | - | - |  | - | - | normal | normal |
| 159 | Sarcoma |  | - | - |  | - | - | normal | normal |
| 160 | Pancreatic cancer | KRAS c.34_35delGGinsCT (p.G12L) | - | - | KRAS c.34_35delGGinsCT (p.G12L) | - | - | 2C.1 | 2C.1 |
| 161 | Prostate | TP53 c.818G>A (p.Arg273His) | - | - | TP53 c.818G>A (p.Arg273His) | - | - | 2C.2 | 2C.2 |
| 162 | Unknown |  | - | - |  | - | - | normal | normal |
| 163 | Pancreatic cancer | TP53 c.517G>A (p.Val173Met) | - | - |  | - | - | 2C.2 | 2C.2 |
| 164 | Salivary gland | TP53 c.847C>T (p.Arg283Cys) | - | - |  | - | - | 2C.2 | 2C.2 |
| 165 | Maxillary |  | - |  |  | - |  | normal | normal |
| 166 | Uterine |  | - | - |  | - | - | normal | normal |
| 167 | Lepidic | KRAS c.35G>C (p.Gly12Ala) | - | - | KRAS c.35G>C (p.Gly12Ala), ATM c.7976T>G (p.Leu2659Ter) | - | - | 2C.1 | 2C.1 |
| 168 | Squamous Cell Carcinoma |  | - | - |  | - | - | normal | normal |
| 169 | Prostate | TP53 c.1039delG (p.Ala347fs) | - | - | TP53 c.1039delG (p.Ala347fs) | - | - | 2C.2 | 2C.2 |
| 170 | Lung | TP53 c.659A>G (p.Y220C) | - | - | TP53 c.659A>G (p.Y220C) | - | - | 2C.2 | 2C.2 |
| 171 | Ampulla of Vater |  | - | - |  | - | - | normal | normal |
| 172 | Lung |  | - | - |  | - | - | normal | normal |
| 173 | Lung | TP53 c.398T>C (p.Met133Thr) | - | - | TP53 c.398T>C (p.Met133Thr) | - | - | 2C.2 | 2C.2 |
| 174 | Pancreatic cancer | KRAS c.37G>T (p.Gly13Cys) | - | - | KRAS c.37G>T (p.Gly13Cys) | - | - | 2C.1 | 2C.1 |
| 175 | Lung | KRAS c.35G>Α (p.Gly12Asp), TP53 c.701A>G (p.Tyr234Cys) | - | - | KRAS c.35G>Α (p.Gly12Asp), TP53 c.701A>G (p.Tyr234Cys) | - | - | 1A.2R | 1A.2R |
| 176 | Breast |  | - | - | NOTCH1 c.7398delG p.Ser2467fs) | - | - | normal | 2C.2 |
| 177 | Pancreatic cancer | KRAS c.183A>C (p.Gln61His), TP53 c.646G>A (p.Val216Met) | - | - | KRAS c.183A>C (p.Gln61His), TP53 c.646G>A (p.Val216Met) | - | - | 2C.1 | 2C.1 |
| 178 | Prostate |  | - | - |  | - | - | normal | normal |
| 179 | Osteosarcoma |  | - | - |  | - | - | normal | normal |
| 180 | Endometrial | PIK3CA c.3140A>G (p.His1047Arg) | - | - |  | - | - | 2C.1 | 2C.1 |
| 181 | Brain | IDH1 c.395G>A (p.R132H | - | - |  | - | - | normal | 2C.1 |
| 182 | Pancreatic cancer | KRAS c.35G>T (p.Gly12Val) | - | - | KRAS c.35G>T (p.Gly12Val) | - | - | 2C.1 | 2C.1 |
| 183 | Unknown |  | - | - |  | - | - | normal | normal |
| 184 | Pancreatic cancer | KRAS c.35G>A (p.Gly12Asp) | - | - | KRAS c.35G>A (p.Gly12Asp) | - | - | 2C.1 | 2C.1 |
| 185 | Squamous epithelial |  | - | - |  | - | - | normal | normal |
| 186 | Thyroid |  | - | - |  | - | - | normal | normal |
| 187 | Gastric |  | - | - |  | - | - | normal | normal |
| 188 | Pancreatic cancer |  | - | - |  | - | - | normal | normal |
| 189 | Lung |  | - | - |  | - | - | normal | normal |
| 190 | Lung | KRAS c.35G>T (p.Gly12Val), PIK3CA c.1633G>A (p.Glu545Lys), CTNNB1 c.110C>G (p.Ser37Cys) | - | - | KRAS c.35G>T (p.Gly12Val), PIK3CA c.1633G>A (p.Glu545Lys), CTNNB1 c.110C>G (p.Ser37Cys) | - | - | 1A.2R | 1A.2R |
| 191 | Pancreatic cancer | TP53 c.532C>T (p.His178Tyr) | - | - | TP53 c.532C>T (p.His178Tyr) | - | - | 2C.2 | 2C.2 |
| 192 | Pancreatic cancer | KRAS c.35G>A (p.Gly12Asp), TP53 c.659A>G (p.Tyr220Cys) | - | - | KRAS c.35G>A (p.Gly12Asp), CDKN2A c.221delA (p.Leu74fs), TP53 c.659A>G (p.Tyr220Cys) | - | - | 2C.1 | 2C.1 |
| 193 | Pancreatic cancer | KRAS c.35G>T (p.Gly12Val), CDKN2A c.233_234delTC (p.Leu78fs) | - | - | KRAS c.35G>T (p.Gly12Val), CDKN2A c.233_234delTC (p.Leu78fs) | - | - | 2C.1 | 2C.1 |
| 194 | Billiary tract |  | - | - |  | - | - | normal | normal |
| 195 | Cholangiocarcinoma |  | - | - | IDH1 c.394C>T (p.Arg132Cys) | - | - | normal | 2C.1 |
| 196 | Lung | PIK3CA c.1624G>A(p.Glu542Lys) | - | - | IDH1 c.394C>T(p.Arg132Cys), PIK3CA c.1624G>A(p.Glu542Lys) | - | - | 2C.1 | 2C.1 |
| 197 | Cholangiocarcinoma |  | - | - |  | - | - | normal | normal |
| 198 | Pancreatic cancer | KRAS c.34G>C (p.Gly12Arg), TP53 c.527G>T (p.Cys176Phe) | - | - | KRAS c.34G>C (p.Gly12Arg), TP53 c.527G>T (p.Cys176Phe) | - | - | 2C.1 | 2C.1 |
| 199 | Lung | KRAS c.34G>C (p.Gly12Arg), | - | - | KRAS c.34G>C (p.Gly12Arg), | - | - | 1A.2R | 1A.2R |
| 200 | Gastric | PIK3CA c.1634A>G (p.Glu545Gly) | - | - | PIK3CA c.1634A>G (p.Glu545Gly) | - | - | 2C.1 | 2C.1 |
| 201 | Liver | NRAS c.182A>G (p.Gln61Arg) | - | - | NRAS c.182A>G (p.Gln61Arg) | - | - | 2D | 2D |
| 202 | Breast | PIK3CA c.3140A>G (p.His1047Arg) | - | - | PIK3CA c.3140A>G (p.His1047Arg) | - | - | 1A.1 | 1A.1 |
| 203 | Lung | EGFR exon 19:c.2236_2250delGAATTAAGAGAAGCA (p.Glu746_Ala750del), EGFR exon 20: c.2369C>T (p.T790M) | - | - | EGFR exon 19:c.2236_2250delGAATTAAGAGAAGCA (p.Glu746_Ala750del), EGFR exon 20: c.2369C>T (p.T790M) | - | - | 1A.1 | 1A.1 |
| 204 | Pancreatic cancer | KRAS c.35G>T (p.Gly12Val) | - | - | KRAS c.35G>T (p.Gly12Val) | - | - | 2C.1 | 2C.1 |
| 205 | Adrenal gland |  | - | - |  | - | - | normal | normal |
| 206 | Lung |  | - | - |  | - | - | normal | normal |
| 207 | GIST |  | - | - | KIT exon 11 c.1669_1674delTGGAAG (p.Trp557_Lys558del) | - | - | normal | 1A.1 |
| 208 | Cholangiocarcinoma |  | - |  |  | - |  | normal | normal |
| 209 | Ovarian | TP53 c.997delC (p.Arg333fs) | - |  | TP53 c.997delC (p.Arg333fs) | - |  | 2C.2 | 2C.2 |
| 210 | kidney |  | - | - |  | - | - | normal | normal |
| 211 | Lung | PIK3CA c.1624G>A(p.Glu542Lys), TP53 c.817C>T(p.R273C) | - | - | PIK3CA c.1624G>A(p.Glu542Lys), TP53 c.817C>T(p.R273C) | - | - | 2C.1 | 2C.1 |
| 212 | Pancreatic cancer | KRAS c.35G>A (p.Gly12Asp), TP53 c.646G>A (p.Val216Met) | - | - | KRAS c.35G>A (p.Gly12Asp), TP53 c.646G>A (p.Val216Met) | - | - | 2C.1 | 2C.1 |
| 213 | Pancreatic cancer | PIK3CA c.3140A>G (p.H1047R) |  | - | PIK3CA c.3140A>G (p.H1047R) | - | - | 2C.1 | 2C.1 |
| 214 | Skin |  | - | - |  | - | - | normal | normal |
| 215 | Breast |  | - | - |  | - | - | normal | normal |
| 216 | Pancreatic cancer | TP53 c.211_220delCCCCCCGTGG (p.Pro72fs) | - | - | TP53 c.211_220delCCCCCCGTGG (p.Pro72fs) | - | - | 2C.2 | 2C.2 |
| 217 | Gastric |  | - | - |  | - | - | normal | normal |
| 218 | Breast | PIK3CA c.3140A>G (p.His1047Arg) | - | - | PIK3CA c.3140A>G (p.His1047Arg) | - | - | 1A.1 | 1A.1 |
| 219 | Pancreatic cancer |  | - | - |  | - | - | normal | normal |
| 220 | Melanoma |  | - | - |  | - | - | normal | normal |
| 221 | Lung | KRAS c.34G>T (p.Gly12Cys) | - | - | KRAS c.34G>T (p.Gly12Cys) | - | - | 1A.2R | 1A.2R |
| 222 | Prostate |  | - | - |  | - | - | normal | normal |
| 223 | Appendix | KRAS c.38G>A (p.Gly13Asp), TP53 c.1024C>T (p.Arg342Ter) | - | - | KRAS c.38G>A (p.Gly13Asp), TP53 c.1024C>T (p.Arg342Ter) | - | - | 2C.1 | 2C.1 |
| 224 | Pancreatic cancer | KRAS c.35G>T (p.Gly12Val) | - | - | KRAS c.35G>T (p.Gly12Val) | - | - | 2C.1 | 2C.1 |
| 225 | Pancreatic cancer |  | - | - |  | - | - | normal | normal |
| 226 | Lung | BRAF c.1799T>A (p.Val600Glu), TP53 c.991C>T (p.Gln331Ter) | - | - | BRAF c.1799T>A (p.Val600Glu), TP53 c.991C>T (p.Gln331Ter) | - | - | 1A.1 | 1A.1 |
| 227 | Desmoplastic |  | - | - |  | - | - | normal | normal |
| 228 | Pancreatic cancer | KRAS c.35G>A (p.Gly12Asp) | - | - |  | - | - | 2C.1 | 2C.1 |
| 229 | Rectal | TP53 c.641A>G (p.His214Arg) | - | - |  | - | - | 1A.1 | 1A.1 |
| 230 | Medullary Thyriod |  | - | - | RET c.2753T>C (p.Met918Thr) | - | - | normal | 1A.1 |
| 231 | Sarcoma |  | - | - |  | - | - | normal | normal |
| 232 | Colon | TP53 c.586C>T (p.Arg196Ter) | - | - | TP53 c.586C>T (p.Arg196Ter) | - | - | 1A.1 | 1A.1 |
| 233 | Prostate | - | - | - | - | - | - | normal | normal |
| 234 | Breast | PIK3CA c.1633G>A (p.Glu545Lys) | - | - | PIK3CA c.1633G>A (p.Glu545Lys) | - | - | 1A.1 | 1A.1 |
| 235 | Cholangiocarcinoma | - | - | - | - | - | - | normal | normal |
| 236 | Lung | - | - | ALK, EML4(13)-ALK(20) | - | - | ALK, EML4(13)-ALK(20) | 1A.1 | 1A.1 |
| 237 | Pancreatic cancer | KRAS c.35G>A (p.Gly12Asp), TP53 c.818G>A (p.Arg273His) | - | - | KRAS c.35G>A (p.Gly12Asp), TP53 c.818G>A (p.Arg273His) | - | - | 2C.1 | 2C.1 |
| 238 | Colon |  | - | - |  | - | - | 1A.1 | 1A.1 |
| 239 | Sarcoma |  |  | - |  | - | - | normal | normal |
| 240 | Colon | KRAS c.38G>A (p.Gly13Asp), TP53 c.469G>T (p.Val157Phe) | - | - | KRAS c.38G>A (p.Gly13Asp), TP53 c.469G>T (p.Val157Phe) | - | - | 1A.1R | 1A.1R |
| 241 | Pancreatic cancer | KRAS c.35G>A (p.Gly12Asp) | - | - | KRAS c.35G>A (p.Gly12Asp) | - | - | 2C.1 | 2C.1 |
| 242 | Adenocarcinoma | TP53 c.842G>T (p.Cys275Phe) | - | - | TP53 c.842G>T (p.Cys275Phe) | - | - | normal | normal |
| 243 | Meningioma |  | - | - |  | - | - | normal | normal |
| 244 | Pancreatic cancer |  | - | - |  | - | - | normal | normal |
| 245 | Melanoma |  | - | - | GNA11 c.626A>T (p.Gln209Leu) | - | - | normal | 2C.2 |
| 246 | Prostate |  | - | - |  | - | - | normal | normal |
| 247 | Gastric | TP53 c.733G>A (p.Gly245Ser) | - | - | TP53 c.733G>A (p.Gly245Ser) | - | - | 2C.2 | 2C.2 |
| 248 | Breast |  | - | - | PIK3CA c.1035T>A (p.Asn345Lys) | - | - | normal | 1A.1 |
| 249 | Adrenocortical |  | - | - |  | - | - | normal | normal |
| 250 | Ovarian | NRAS c.182A>G (p.Gln61Arg) | - | - | NRAS c.182A>G (p.Gln61Arg) | - | - | 2D | 2D |
| 251 | kidney |  | - | - |  | - | - | 2C.1 | 2C.1 |
| 252 | Endometrial | TP53 c.712_725delTGTAACAGTTCCTG (p.Cys238fs) | - | - | TP53 c.712_725delTGTAACAGTTCCTG (p.Cys238fs) | - | - | 2C.2 | 2C.2 |
| 253 | Cholangiocarcinoma | CTNNB1 c.121A>G (p.Thr41Ala), TP53 c.796G>A (p.Gly266Arg) | - | - | CTNNB1 c.121A>G (p.Thr41Ala), TP53 c.796G>A (p.Gly266Arg) | - | - | 2C.2 | 2C.2 |
| 254 | Lung | TP53 c.405_405delCinsTGTTTTGA (p.Gln136fs) | - | - | TP53 c.405_405delCinsTGTTTTGA (p.Gln136fs) | - | - | 2C.2 | 2C.2 |
| 255 | Lung |  | - | - |  | - | - | normal | normal |
| 256 | Brain |  | - | - | EGFR c.866C>T (p.Ala289Val) | - | - | normal | 2C.1 |
| 257 | Lung |  | - | - |  | - | - | normal | normal |
| 258 | Lung |  | - | - |  | - | - | normal | normal |
| 259 | Lung | TP53 c.660T>G (p.Tyr220Ter) | - | - | TP53 c.660T>G (p.Tyr220Ter) | - | - | 2C.2 | 2C.2 |
| 260 | Sarcoma |  | - | - |  | - | - | normal | normal |
| 261 | Ovarian |  | - | - |  | - | - | normal | normal |
| 262 | Anal melanoma | BRAF c.1397G>A (p.Gly466Glu) | - | - | BRAF c.1397G>A (p.Gly466Glu) | - | - | 2C.2 | 2C.2 |
| 263 | Colon | KRAS c.34G>T (p.Gly12Cys) | - | - | KRAS c.34G>T (p.Gly12Cys) | - | - | 1A.1R | 1A.1R |
| 264 | Breast | - | - | - | - | - | - | normal | normal |
| 265 | Gastric | PIK3CA c.1633G>A (p.Glu545Lys) | - | - | PIK3CA c.1633G>A (p.Glu545Lys) | - | - | 2C.1 | 2C.1 |
| 266 | Breast |  | - | - |  | - | - | 1A.1 | 1A.1 |
| 267 | Cholangiocarcinoma |  | - | - |  | - | - | normal | normal |
| 268 | Endometrial | KRAS c.35G>C (p.Gly12Ala), KRAS c.182A>T (p.Gln61Leu) | - | - | KRAS c.35G>C (p.Gly12Ala), KRAS c.182A>T (p.Gln61Leu) | - | - | 2C.1 | 2C.1 |
| 269 | Colon | PIK3CA c.3062A>G (p.Tyr1021Cys) | - | - | PIK3CA c.3062A>G (p.Tyr1021Cys) | - | - | 1A.1 | 1A.1 |
| 270 | Breast |  | - | - |  | - | - | normal | normal |
| 271 | Liver |  | - | - | IDH1 c.394C>T (p.R132C) | - | - | normal | 2C.1 |
| 272 | Breast |  | - | - |  | - | - | normal | normal |
| 273 | Blue round desmoplastic sarcoma |  | - | - |  | - | - | normal | normal |
| 274 | Brain |  | - | - | CDKN2A c.382_383insC (p.Arg128fs),TP53 c.647_648delTG (p.Val216fs) | - | - | normal | 2C.2 |
| 275 | Schwanomma |  | - | - |  | - | - | normal | normal |
| 276 | Pancreatic cancer | KRAS c.34G>C (p.Gly12Arg) | - | - | KRAS c.34G>C (p.Gly12Arg) | - | - | 2C.1 | 2C.1 |
| 277 | Pancreatic cancer | KRAS c.35G>T (p.Gly12Val) | - | - | KRAS c.35G>T (p.Gly12Val) | - | - | 2C.1 | 2C.1 |
| 278 | Head and Neck | PIK3CA c.3140A>G (p.His1047Arg), TP53 c.309C>A ( p.Tyr103Ter) | - | - | PIK3CA c.3140A>G (p.His1047Arg), TP53 c.309C>A ( p.Tyr103Ter) | - | - | 2C.1 | 2C.1 |
| 279 | Colon | TP53 c.841G>A (p.Asp281Asn), TP53 c.855G>C (p.Glu285Asp) | - | - | TP53 c.841G>A (p.Asp281Asn), TP53 c.855G>C (p.Glu285Asp) | - | - | 1A.1 | 1A.1 |
| 280 | Liver |  | - | - | GNAQ c.626A>C (p.Gln209Pro), | - | - | normal | 2C.2 |
| 281 | Colon |  | - | - |  | - | - | 1A.1 | 1A.1 |
| 282 | Pancreatic cancer | KRAS c.34G>C (p.Gly12Arg), TP53 c.916C>T (p.Arg306Ter) | - | - | KRAS c.34G>C (p.Gly12Arg), TP53 c.916C>T (p.Arg306Ter) | - | - | 2C.1 | 2C.1 |
| 283 | Endometrial | PIK3CA c.3140A>G (p.His1047Arg) | - | - | PIK3CA c.3140A>G (p.His1047Arg) | - | - | 2C.1 | 2C.1 |
| 284 | Urothelial |  | FGFR1 Amplification | - |  | FGFR1 Amplification | - | 2C.1 | 2C.1 |
| 285 | breast |  | ERBB2 Amplification | - |  | ERBB2 Amplification | - | 1A.1 | 1A.1 |
| 286 | breast |  | - | - |  | - | - | normal | normal |
| 287 | Oesophagus | TP53 c.586C>T (p.Arg196Ter) | MYC Amplification | - | TP53 c.586C>T (p.Arg196Ter) | - | - | 2C.2 | 2C.2 |
| 288 | Pancreatic cancer | KRAS c.34G>C (p.Gly12Arg) | - | - | KRAS c.34G>C (p.Gly12Arg) | - | - | 2C.1 | 2C.1 |
| 289 | Ovarian | KRAS c.35G>A (p.Gly12Asp) | - | - | KRAS c.35G>A (p.Gly12Asp) | - | - | 2C.1 | 2C.1 |
| 290 | Pancreatic cancer | KRAS c.38G>A (p.Gly13Asp), TP53 c.524G>A (p.Arg175His) | - | - | KRAS c.38G>A (p.Gly13Asp), TP53 c.524G>A (p.Arg175His) | - | - | 2C.1 | 2C.1 |
| 291 | Ovarian | - | MYC Amplification | - | - | - | - | normal | normal |
| 292 | Pancreatic cancer | KRAS c.34G>C (p.Gly12Arg) | - | - | KRAS c.34G>C (p.Gly12Arg) | - | - | 2C.1 | 2C.1 |
| 293 | Lung | KRAS c.35G>T (p.Gly12Val), TP53 c.524G>A (p.Arg175His) | - | - | KRAS c.35G>T (p.Gly12Val), TP53 c.524G>A (p.Arg175His) | - | - | 1A.2R | 1A.2R |
| 294 | Pancreatic cancer |  | - |  |  | - |  | normal | normal |
| 295 | Gastric |  | - |  |  | - |  | normal | normal |
| 296 | Prostate |  | - |  |  | - |  | normal | normal |
| 297 | Gastric | TP53 c.518T>C (p.Val173Ala) | - |  | TP53 c.518T>C (p.Val173Ala) | - |  | 2C.2 | 2C.2 |
| 298 | Pulmonary angiosarcoma |  | - |  |  | - |  | normal | normal |
| 299 | Prostate cancer | TP53 c.395A>T (p.Lys132Met) | - |  | TP53 c.395A>T (p.Lys132Met) | - |  | 2C.2 | 2C.2 |
| 300 | Breast cancer |  | - |  | PIK3CA c.1035T>A (p.Asn345Lys) | - |  | normal | 2C.1 |
| 301 | Breast cancer | TP53 c.291_292insTTCTGTC (p.Pro98fs) | - |  | TP53 c.291_292insTTCTGTC (p.Pro98fs) | - |  | 2C.2 | 2C.2 |
| 302 | Melanoma | BRAF c.1801A>G (p.Lys601Glu) | - |  | BRAF c.1801A>G (p.Lys601Glu) | - |  | 2C.2 | 2C.2 |
| 303 | Pancreatic cancer | KRAS c.35G>T (p.Gly12Val), SLX4 c.212C>G (p.Ser71Ter), TP53 c.743G>A (p.Arg248Gln) | - |  | KRAS c.35G>T (p.Gly12Val), SLX4 c.212C>G (p.Ser71Ter), TP53 c.743G>A (p.Arg248Gln) | - |  | 2C.1 | 2C.1 |
| 304 | Breast cancer | PIK3CA c.1624G>A (p.Glu542Lys) | - | - | PIK3CA c.1624G>A (p.Glu542Lys), PIK3CA c.2176G>A (p.Glu726Lys) | - | - | 1A.1 | 1A.1 |
| 305 | Pancreatic cancer | KRAS c.35G>A (p.Gly12Asp) | - | - | KRAS c.35G>A (p.Gly12Asp) | - | - | 2C.1 | 2C.1 |
| 306 | Colon | TP53 c.833C>T (p.Pro278Leu) | - | - | TP53 c.833C>T (p.Pro278Leu) | - | - | 1A.1 | 1A.1 |
| 307 | Pancreatic cancer | KRAS c.35G>A (p.Gly12Asp), TP53 c.725G>T (p.Cys242Phe) | - |  | KRAS c.35G>A (p.Gly12Asp), TP53 c.725G>T (p.Cys242Phe) | - |  | 2C.1 | 2C.1 |
| 308 | Gastric cancer |  | - | - |  | - | - | normal | normal |
| 309 | Ampulla of Vater | KRAS c.35G>A (p.Gly12Asp), TP53 c.536A>G (p.His179Arg) | - | - | KRAS c.35G>A (p.Gly12Asp), RNF43 c.1177_1178insG (p.Ala393fs), TP53 c.536A>G (p.His179Arg) | - | - | 2C.1 | 2C.1 |
| 310 | Ovarian cancer | TP53 c.659A>G (p.Tyr220Cys) | - | - | TP53 c.659A>G (p.Tyr220Cys) | - | - | 2C.2 | 2C.2 |
| 311 | Endometrial cancer | TP53 c.843C>G (p.Asp281Glu) | - | - | TP53 c.843C>G (p.Asp281Glu) | - | - | 2C.2 | 2C.2 |
| 312 | Pancreatic cancer | KRAS c.35G>A (p.Gly12Asp), CDKN2A c.172C>T (p.Arg58Ter), TP53 c.818G>A (p.Arg273His) | - | - | KRAS c.35G>A (p.Gly12Asp), CDKN2A c.172C>T (p.Arg58Ter), TP53 c.818G>A (p.Arg273His) | - | - | 2C.1 | 2C.1 |
| 313 | Sarcoma | PIK3CA c.3140A>G (p.His1047Arg), TP53 c.151G>T (p.Glu51Ter) | - | - | PIK3CA c.3140A>G (p.His1047Arg), TP53 c.151G>T (p.Glu51Ter) | - | - | 2C.1 | 2C.1 |
| 314 | Brain tumor |  | - | - |  | - | - | normal | normal |
| 315 | Lung cancer | BRAF c.1799T>A (p.V600E) | - | - | BRAF c.1799T>A (p.V600E) | - | - | 1A.1 | 1A.1 |
| 316 | Breast cancer |  | - | - |  | - | - | normal | normal |
| 317 | Brain tumor |  | - | - |  | - | - | normal | normal |
| 318 | Bladder cancer | TP53 c.839G>C (p.Arg280Thr) | - | - | TP53 c.839G>C (p.Arg280Thr) | - | - | 2C.2 | 2C.2 |
| 319 | Pancreatic cancer | KRAS c.35G>A (p.Gly12Asp), TP53 c.310C>T (p.Gln104Ter) | - | - | KRAS c.35G>A (p.Gly12Asp), TP53 c.310C>T (p.Gln104Ter) | - | - | 2C.1 | 2C.1 |
| 320 | Sarcoma |  | - | - |  | - | - | 2C.1 | 2C.1 |
| 321 | Lung cancer |  | - | - |  | - | - | normal | normal |
| 322 | kidney cancer | PIK3CA c.1633G>A (p.Glu545Lys) | - | - | PIK3CA c.1633G>A (p.Glu545Lys), PIK3CA c.2176G>A (p.Glu726Lys) | - | - | 2C.1 | 2C.1 |
| 323 | Ovarian cancer | TP53 c.578A>G (p.His193Arg) | - | - | TP53 c.578A>G (p.His193Arg) | - | - | 1A.1 | 1A.1 |
| 324 | Gastric cancer |  | - | - |  | - | - | normal | normal |
| 325 | Endometrial cancer |  | - | - |  | - | - | normal | normal |
| 326 | Endometrial cancer | KRAS c.34G>A (p.Gly12Ser), FBXW7 c.1513C>T (p.Arg505Cys), TP53c.743G>A (p.Arg248Gln) | - | - | KRAS c.34G>A (p.Gly12Ser), FBXW7 c.1513C>T (p.Arg505Cys), TP53c.743G>A (p.Arg248Gln) | - | - | 2C.1 | 2C.1 |
| 327 | Breast cancer | ΑΚΤ1 c.49G>A (p.Glu17Lys), TP53 c.715A>G (p.Asn239Asp) | - | - | ΑΚΤ1 c.49G>A (p.Glu17Lys), TP53 c.715A>G (p.Asn239Asp) | - | - | 2C.2 | 2C.2 |
| 328 | Ovarian cancer |  | - | - |  | - | - | normal | normal |
| 329 | kidney | TP53 c.747G>T (p.Arg249Ser) | - | - | TP53 c.747G>T (p.Arg249Ser) | - | - | 2C.2 | 2C.2 |
| 330 | Melanoma |  |  | - |  | - | - | normal | normal |
| 331 | Pancreatic cancer | KRAS c.35G>T (p.Gly12Val), PIK3CA c.3139C>T (p.His1047Tyr) | - | - | KRAS c.35G>T (p.Gly12Val), PIK3CA c.3139C>T (p.His1047Tyr) | - | - | 2C.1 | 2C.1 |
| 332 | Colon | KRAS c.35G>C (p.Gly12Ala), TP53 c.844C>T (p.Arg282Trp) | - | - | KRAS c.35G>C (p.Gly12Ala), TP53 c.844C>T (p.Arg282Trp) | - | - | 1A.1R | 1A.1R |
| 333 | Urothelial |  | - | - | PIK3CA c.1030G>A (p.Val344Met) | - | - | normal | 2C.1 |
| 334 | Breast |  | - | - |  | - | - | normal | normal |
| 335 | Prostate |  | - |  |  | - |  | normal | normal |
| 336 | Endometrial | TP53 c.843C>G (p.Asp281Glu) | - | - | TP53 c.843C>G (p.Asp281Glu) | - | - | 2C.2 | 2C.2 |
| 337 | Breast | TP53 c.586C>T (p.Arg196Ter) | - | - | TP53 c.586C>T (p.Arg196Ter) | - | - | 2C.2 | 2C.2 |
| 338 | Pancreatic cancer |  | - | - |  | - | - | normal | normal |
| 339 | Colon | TP53 c.743G>A (p.Arg248Gln) | - | - | TP53 c.743G>A (p.Arg248Gln) | - | - | 1A.1 | 1A.1 |
| 340 | Prostate |  | - | - |  | - | - | normal | normal |
| 341 | Adenoid |  | - | - |  | - | - | normal | normal |
| 342 | Lung | TP53 c.713G>A (p.Cys238Tyr) | - | - | TP53 c.713G>A (p.Cys238Tyr) | - | - | 2C.2 | 2C.2 |
| 343 | Breast |  | - | - |  | - | - | normal | normal |
| 344 | Unknown | TP53 c.592G>T (p.Glu198Ter) | - | - | TP53 c.592G>T (p.Glu198Ter) | - | - | 2C.2 | 2C.2 |
| 345 | Breast |  | - | - |  | - | - | normal | normal |
| 346 | Prostate | PIK3CA c.1633G>A (p.Glu545Lys) | - | - | PIK3CA c.1633G>A (p.Glu545Lys) | - | - | 2C.1 | 2C.1 |
| 347 | Prostate |  | - | - |  | - | - | normal | normal |
| 348 | Colon | BRAF: c.1799T>A p.V600E) | - | - | BRAF: c.1799T>A p.V600E) | - | - | 1A.2 | 1A.2 |
| 349 | Prostate | - | - | - | - | - | - | normal | normal |
| 350 | Pancreatic cancer | TP53 c.916C>T (p.Arg306Ter) | - | - | TP53 c.916C>T (p.Arg306Ter) | - | - | 2C.2 | 2C.2 |
| 351 | Pancreatic cancer | KRAS c.35G>T (p.Gly12Val) | - | - | KRAS c.35G>T (p.Gly12Val) | - | - | 2C.1 | 2C.1 |
| 352 | Ovarian |  | - | - |  | - | - | normal | normal |
| 353 | Liver | KRAS c.35G>A (p.Gly12Asp) | - | - | KRAS c.35G>A (p.Gly12Asp), CDKN2A c.130_131insC (p.Tyr44fs) | - | - | 2C.1 | 2C.1 |
| 354 | Gastric |  | - | - |  | - | - | normal | normal |
| 355 | Malignant Mesenchymal |  | - | - |  | - | - | normal | normal |
| 356 | Oesophagus |  | - | - |  | - | - | normal | normal |
| 357 | Osteosarcoma |  | - | - |  | - | - | normal | normal |
| 358 | Pancreatic cancer | KRAS c.35G>A (p.Gly12Asp), TP53 c.731G>T (p.Gly244Val) | - | - | KRAS c.35G>A (p.Gly12Asp), TP53 c.731G>T (p.Gly244Val) | - | - | 2C.1 | 2C.1 |
| 359 | kidney cancer |  |  | - |  | - | - | normal | normal |
| 360 | Astrocytoma | TP53 c.817C>T (p.Arg273Cys) | - | - | IDH1 c.394C>G (p.Arg132Gly), TP53 c.817C>T (p.Arg273Cys) | - | - | 2C.2 | 2C.1 |
| 361 | Lung |  | - | - |  | - | - | normal | normal |
| 362 | Breast | AKT1 c.49G>A (p.Glu17Lys) | - | - | AKT1 c.49G>A (p.Glu17Lys) | - | - | 2C.2 | 2C.2 |
| 363 | Head and Neck | TP53 c.743G>A (p.Arg248Gln) | - | - | TP53 c.743G>A (p.Arg248Gln) | - | - | 2C.2 | 2C.2 |
| 364 | Pancreatic cancer | TP53 c.646G (p.Val216Leu) | - | - | TP53 c.646G (p.Val216Leu) | - | - | 2C.2 | 2C.2 |
| 365 | Colon | KRAS c.35G>A (p.Gly12Asp), PIK3CA c.3140A>G (p.His1047Arg) | - |  | KRAS c.35G>A (p.Gly12Asp), PIK3CA c.3140A>G (p.His1047Arg) | - |  | 1A.1R | 1A.1R |
| 366 | Osteosarcoma |  | - | - |  | - | - | normal | normal |
| 367 | Lymph Nodes | TP53 c.706T>C (p.Tyr236His) | - | - | TP53 c.706T>C (p.Tyr236His) | - | - | 2C.2 | 2C.2 |
| 368 | Pancreatic cancer |  | - | - |  | - | - | normal | normal |
| 369 | Possible relapse of Ewing sarcoma |  | - | - |  | - | - | normal | normal |
| 370 | Pancreatic cancer | KRAS c.35G>A (p.Gly12Asp), SMAD4 c.1576G>T (p.Glu526Ter), TP53 c.733G>A (p.Gly245Ser) | - | - | KRAS c.35G>A (p.Gly12Asp), SMAD4 c.1576G>T (p.Glu526Ter), TP53 c.733G>A (p.Gly245Ser) | - | - | 2C.1 | 2C.1 |
| 371 | Cholangiocarcinoma | KRAS c.35G>A (p.Gly12Asp) | - | - | KRAS c.35G>A (p.Gly12Asp) | - | - | 2C.1 | 2C.1 |
| 372 | Lung | BRAF c.1799T>A (p.Val600Glu), STK11 c.109C>T (p.Gln37Ter) | - | - | BRAF c.1799T>A (p.Val600Glu), STK11 c.109C>T (p.Gln37Ter) | - | - | 1A.1 | 1A.1 |
| 373 | Brain | - | FGFR1 Amplification | - | - | FGFR1 Amplification | - | 2C.1 | 2C.1 |
| 374 | Pancreatic cancer | KRAS c.35G>A (p.Gly12Asp), TP53 c.524G>A (p.Arg175His) | - | - | KRAS c.35G>A (p.Gly12Asp), TP53 c.524G>A (p.Arg175His) | - | - | 2C.1 | 2C.1 |
| 375 | Endometrium | PIK3CA c.3129G<T (p.Met1043Ile), TP53 c.818G>A (p.Arg273His) | - | - | PIK3CA c.3129G<T (p.Met1043Ile), TP53 c.818G>A (p.Arg273His) | - | - | 2C.1 | 2C.1 |
| 376 | Stomach | TP53 c.524G>A (p.Arg175His) | - | - | TP53 c.524G>A (p.Arg175His) | - | - | 2C.2 | 2C.2 |
| 377 | Pancreatic cancer |  | - | - | CDKN2B c.244C>T (p.Arg82Ter) | - | - | normal | 2C.2 |
| 378 | Lung | KRAS c.35G>C (p.Gly12Ala) | - | - | KRAS c.35G>C (p.Gly12Ala) | - | - | 1A.2R | 1A.2R |
| 379 | Lung |  | - | - |  | - | - | normal | normal |
| 380 | Head and Neck |  | - | - |  | - | - | normal | normal |
| 381 | Prostate |  | - | - |  | - | - | normal | normal |
| 382 | Breast | PIK3CA c.3140A>G (p.His1047Arg), PTEN c.367C>T (p.His123Tyr), PTEN c.511C>T (p.Gln171Ter) | - | - | PIK3CA c.3140A>G (p.His1047Arg), PTEN c.367C>T (p.His123Tyr), PTEN c.511C>T (p.Gln171Ter) | - | - | 1A.1 | 1A.1 |
| 383 | Lung | KRAS c.35G>T (p.Gly12Val), TP53 c.613T>G (p.Tyr205Asp) | - | - | KRAS c.35G>T (p.Gly12Val), TP53 c.613T>G (p.Tyr205Asp) | - | - | 1A.2R | 1A.2R |
| 384 | Pancreatic cancer | KRAS c.34G>C (p.Gly12Arg) | - | - | KRAS c.34G>C (p.Gly12Arg) | - | - | 2C.1 | 2C.1 |
| 385 | Pancreatic cancer | KRAS c.38G>A (p.Gly13Asp), TP53 c.742C>T (p.Arg248Trp) | - | - | KRAS c.38G>A (p.Gly13Asp), TP53 c.742C>T (p.Arg248Trp) | - | - | 2C.1 | 2C.1 |
| 386 | Metastatic right thigh liposarcoma | PIK3CA c.3140A>G (p.His1047Arg) | - | - | PIK3CA c.3140A>G (p.His1047Arg) | - | - | 2C.1 | 2C.1 |
| 387 | Lung | KRAS c.34G>A (p.Gly12Ser) | KRAS Amplification | - | KRAS c.34G>A (p.Gly12Ser) | KRAS Amplification | - | 1A.2R | 1A.2R |
| 388 | Hepatocellular carcinoma |  | - | - |  | - | - | normal | normal |
| 389 | Gastric |  | - | - |  | - | - | normal | normal |
| 390 | Gastric |  | - | - |  | - | - | normal | normal |
| 391 | Lung Metastatic adenorcinoma | TP53 c.614_621delATTTGGAT (p.Tyr205Ter) | - | - | TP53 c.614_621delATTTGGAT (p.Tyr205Ter) | - | - | 2C.2 | 2C.2 |
| 392 | Breast cancer | PIK3CA c.1633G>A (p.Glu545Lys) | - | - | PIK3CA c.1633G>A (p.Glu545Lys) | - | - | 1A.1 | 1A.1 |
| 393 | Liver ncer Hepatocellular rcinoma | - | - | - | - | - | - | normal | normal |
| 394 | Lung cancer | ERBB2 c.2326_2327insTGT (p.Gly776delinsValCys) | - | - | ERBB2 c.2326_2327insTGT (p.Gly776delinsValCys) | - | - | 1B | 1B |
| 395 | kidney kidney | - | - | - | - | - | - | normal | normal |
| 396 | Gastric Gastric | TP53 c.743G>A (p.Arg248Gln) | - | - | TP53 c.743G>A (p.Arg248Gln) | - | - | 2C.2 | 2C.2 |
| 397 | brain tumor |  | - | - |  | - | - | normal | normal |
| 398 | Ovarian | TP53 c.617delT (p.Leu206fs) | - | - |  | - | - | normal | normal |
| 399 | Lung Metastatic Lung |  | - | - | TP53 c.724T>C (p.Cys242Arg) | - | - | 2C.2 | 2C.2 |
| 400 | Colon cancer | BRAF c.1799T>A (p.Val600Glu) | - | - | BRAF c.1799T>A (p.Val600Glu) | - | - | 1A.2 | 1A.2 |
| 401 | Colorectal cancer | TP53 c.844C>T (p.Arg282Trp | - | - | TP53 c.844C>T (p.Arg282Trp | - | - | 2C.2 | 2C.2 |
| 402 | Prostate cancer | FGFR3 c.742C>T (p.Arg248Cys) | - | - | FGFR3 c.742C>T (p.Arg248Cys) | - | - | 2C.1 | 2C.1 |
| 403 | Pancreatic cancer | KRAS c.35G>A (p.Gly12Asp) | - | - | KRAS c.35G>A (p.Gly12Asp) | - | - | 2C.1 | 2C.1 |
| 404 | Lymphoma Lymphoma |  | - | - |  | - | - | normal | normal |
| 405 | Pancreatic cancer | KRAS c.34G>C (p.Gly12Arg) | - | - | KRAS c.34G>C (p.Gly12Arg) | - | - | 2C.1 | 2C.1 |
| 406 | Colon cancer | KRAS c.38G>A (p.Gly13Asp), AKT1 c.155T>G (p.Leu52Arg) | - | - | KRAS c.38G>A (p.Gly13Asp), AKT1 c.155T>G (p.Leu52Arg) | - | - | 1A.1R | 1A.1R |
| 407 | Pancreatic cancer | KRAS c.35G>A (p.Gly12Asp) | - | - | KRAS c.35G>A (p.Gly12Asp) | - | - | 2C.1 | 2C.1 |
| 408 | Esophageal cancer | TP53 c.818G>T (p.Arg273Leu) | - | - | TP53 c.818G>T (p.Arg273Leu) | - | - | 2C.2 | 2C.2 |
| 409 | Prostate cancer | TP53 c.662_672delAGCCGCCTGAG (p.Glu221fs), c.215_216insG (p.Val73fs) | - | - | TP53 c.662_672delAGCCGCCTGAG (p.Glu221fs), c.215_216insG (p.Val73fs) | - | - | 2C.2 | 2C.2 |
| 410 | Pancreatic cancer | KRAS c.183A>C (p.Gln61His) | - | - | KRAS c.183A>C (p.Gln61His) | - | - | 2C.1 | 2C.1 |
| 411 | Colon cancer | KRAS c.180_181delTCinsAA (p.Gln61Lys) | - | - | KRAS c.180_181delTCinsAA (p.Gln61Lys) | - | - | 1A.1R | 1A.1R |
| 412 | Bladder cancer | - | - | - | - | - | - | normal | normal |
| 413 | Pancreatic cancer | KRAS c.35G>T (p.Gly12Val), TP53 c.577C>T (p.His193Tyr) | - | - | KRAS c.35G>T (p.Gly12Val), TP53 c.577C>T (p.His193Tyr) | - | - | 2C.1 | 2C.1 |
| 414 | Prostate cancer |  | - | - |  | - | - | normal | normal |
| 415 | Unknown |  | - | - |  | - | - | normal | normal |
| 416 | Colon cancer | NRAS c.182A>G (p.Gln61Arg), PIK3CA c.1634A>C (p.Glu545Ala) | - | - | NRAS c.182A>G (p.Gln61Arg), PIK3CA c.1634A>C (p.Glu545Ala) | - | - | 1A.1R | 1A.1R |
| 417 | Prostate cancer |  | - | - |  | - | - | normal | normal |
| 418 | Lung cancer |  | - | - |  | - | - | normal | normal |
| 419 | Pancreatic cancer | KRAS c.35G>T (p.Gly12Val) | - | - |  | - | - | 2C.1 | 2C.1 |
| 420 | Prostate cancer |  | - | - |  | - | - | normal | normal |
| 421 | Pancreatic cancer | TP53 c.817C>T (p.Arg273Cys) |  | - | TP53 c.817C>T (p.Arg273Cys) | - | - | 2C.2 | 2C.2 |
| 422 | Ovarian cancer | TP53 c.711G>A (p.Met237Ile) | - | - |  | - | - | 2C.2 | 2C.2 |
| 423 | Liver cancer |  | - | - |  | - | - | normal | normal |
| 424 | Pancreatic cancer | KRAS c.35G>A (p.Gly12Asp), TP53 c.480_496delGGCTCTAAGGT (p.Met160fs) | - | - | KRAS c.35G>A (p.Gly12Asp), TP53 c.480_496delGGCTCTAAGGT (p.Met160fs) | - | - | 2C.1 | 2C.1 |
| 425 | brain tumor Glioblastoma |  | - | - |  | - | - | normal | normal |
| 426 | Pancreatic cancer | KRAS c.35G>A (p.Gly12Asp), TP53 c.839G>C (p.Arg280Thr) | - | - | KRAS c.35G>A (p.Gly12Asp), TP53 c.839G>C (p.Arg280Thr) | - | - | 2C.1 | 2C.1 |
| 427 | Metastatic neuroendocrine cainoma |  | - | - |  | - | - | normal | normal |
| 428 | Lung cancer | EGFR exon 19 c.2235_2249delGGAATTAAGAGAAGC (p.Glu746_Ala750del) | - | - | EGFR exon 19 c.2235_2249delGGAATTAAGAGAAGC (p.Glu746_Ala750del) | - | - | 1A.1 | 1A.1 |
| 429 | Lung cancer | KRAS c.35G>C (p.Gly12Ala), TP53 c.469G>T (p.Val157Phe) | - | - | KRAS c.35G>C (p.Gly12Ala), TP53 c.469G>T (p.Val157Phe) | - | - | 1A.2R | 1A.2R |
| 430 | Sarcoma Angiosarcoma |  | - | - | IDH1 c.394C>T (p.Arg132Cys) | - | - | normal | 2C.1 |
| 431 | Cholangiocarcinoma |  | - | - |  | - | - | normal | normal |
| 432 | Lung cancer | PIK3CA c.1633G>A (p.Glu545Lys),TP53 c.949C>T (p.Gln317Ter) | - | - | PIK3CA c.1633G>A (p.Glu545Lys),TP53 c.949C>T (p.Gln317Ter) | - | - | 2C.1 | 2C.1 |
| 433 | Breast cancer | TP53c.743G>A (p.Arg248Gln) | - | - | TP53c.743G>A (p.Arg248Gln) | - | - | 2C.2 | 2C.2 |
| 434 | Pancreatic cancer |  | - | - |  | - | - | normal | normal |
| 435 | Cervical cancer |  | AKT1 Amplification | - |  | AKT1 Amplification | - | 2C.2 | 2C.2 |
| 436 | Colon cancer |  | - | - |  | - | - | 1A.1 | 1A.1 |
| 437 | Thymic carcinoma |  | - | - |  | - | - | normal | normal |
| 438 | Small intestine | KRAS c.182_183delAAinsTT (p.Gln61Leu), SMAD4 c.1081C>T (p.Arg361Cys), TP53 c.733G>A (p.Gly245Ser) | - | - | KRAS c.182_183delAAinsTT (p.Gln61Leu), SMAD4 c.1081C>T (p.Arg361Cys), TP53 c.733G>A (p.Gly245Ser) | - | - | 2C.1 | 2C.1 |
| 439 | Breast cancer | TP53 c.742C>T (p.Arg248Trp) | - | - | TP53 c.742C>T (p.Arg248Trp) | - | - | 2C.2 | 2C.2 |
| 440 | brain tumor |  | - | - |  | - | - | normal | normal |
| 441 | Ovarian cancer | TP53 c.659A>G (p.Tyr220Cys) | - | - | TP53 c.659A>G (p.Tyr220Cys) | - | - | 2C.2 | 2C.2 |
| 442 | Gastric cancer |  | - | - |  | - | - | normal | normal |
| 443 | Melanoma | NRAS c.182A>G (p.Gln61Arg), FGFR3 c.1921G>A (p.Asp641Asn) | - | - | NRAS c.182A>G (p.Gln61Arg), FGFR3 c.1921G>A (p.Asp641Asn) | - | - | normal | normal |
| 444 | Pancreatic cancer |  | - | - |  | - | - | normal | normal |
| 445 | Unknown | PIK3CA c.1633G>A (p.Glu545Lys) | - | - | PIK3CA c.1633G>A (p.Glu545Lys) | - | - | 2C.1 | 2C.1 |
| 446 | Breast cancer | PIK3CA c.3127A>G (p.Met1043Val) | KIT Amplification, PDGFRA Amplification, FGFR1 Amplification |  | PIK3CA c.3127A>G (p.Met1043Val) | - |  | 1A.1 | 1A.1 |
| 447 | Ovarian cancer | TP53 c.379T>A (p.Ser127Thr) | - | - | TP53 c.379T>A (p.Ser127Thr) | - |  | 2C.2 | 2C.2 |
| 448 | Pancreatic cancer | KRAS c.34G>T (p.Gly12Cys), TP53 c.524G>A (p.Arg175His) | - | - | KRAS c.34G>T (p.Gly12Cys), TP53 c.524G>A (p.Arg175His) | - |  | 2C.1 | 2C.1 |
| 449 | colon Rectum | NRAS c.34G>A (p.Gly12Ser) | - | - | NRAS c.34G>A (p.Gly12Ser) | - |  | 1A.1R | 1A.1R |
| 450 | Pancreatic cancer | KRAS c.35G>A (p.Gly12Asp), PIK3CA c.1633G>A (p.Glu545Lys), TP53 c.524G>A (p.Arg175His) | - | - | KRAS c.35G>A (p.Gly12Asp), PIK3CA c.1633G>A (p.Glu545Lys), TP53 c.524G>A (p.Arg175His) | - |  | 2C.1 | 2C.1 |
| 451 | Sarcoma Sarcoma |  | - | - |  | - |  | normal | normal |
| 452 | Prostate cancer |  | ALK Amplification, PDGFRA Amplification, KIT Amplification |  |  | - |  | 2C.2 | 2C.2 |
| 453 | Pancreatic cancer | KRAS c.35G>A (p.Gly12Asp) | - |  | KRAS c.35G>A (p.Gly12Asp) | - |  | 2C.1 | 2C.1 |
| 454 | Lung cancer |  | - | RET, KIF5B(15)-RET(11) |  | - | RET, KIF5B(15)-RET(11) | 1A.1 | 1A.1 |
| 455 | Lung cancer | EGFR c.2235_2249delGGAATTAAGAGAAGC (p.Glu746_Ala750del), TP53 c.730G>A (p.Gly244Ser) | - | - | EGFR c.2235_2249delGGAATTAAGAGAAGC (p.Glu746_Ala750del), TP53 c.730G>A (p.Gly244Ser) | - | - | 1A.1 | 1A.1 |
| 456 | Pancreatic cancer | KRAS c.35G>A (p.Gly12Asp) | - | - | KRAS c.35G>A (p.Gly12Asp) | - | - | 2C.1 | 2C.1 |
| 457 | Unknown | TP53 c.535C>T (p.His179Tyr) | - | - | TP53 c.535C>T (p.His179Tyr) | - | - | 2C.2 | 2C.2 |
| 458 | Ovarian cancer |  | ERBB2 AMPLIFICATION | - |  | - | - | 2C.1 | 2C.1 |
| 459 | Pancreatic cancer | KRAS c.35G>A (p.Gly12Asp) | - | - | KRAS c.35G>A (p.Gly12Asp) | - | - | 2C.1 | 2C.1 |
| 460 | Head and Neck Head and Neck | TP53 c.1027G>T (p.Glu343Ter) | - | - | TP53 c.1027G>T (p.Glu343Ter) | - | - | 2C.2 | 2C.2 |
| 461 | Colon cancer | FBXW7 c.1513C>T (p.Arg505Cys), TP53 c.844C>T (p.Arg282Trp) | - | - | FBXW7 c.1513C>T (p.Arg505Cys), TP53 c.844C>T (p.Arg282Trp) | - | - | 1A.1 | 1A.1 |
| 462 | Pancreatic Possible Pancreatic tic ncer or Billiary duct | KRAS c.35G>A (p.Gly12Asp) | - | - | KRAS c.35G>A (p.Gly12Asp) | - | - | 2C.1 | 2C.1 |
| 463 | Tongue Tongue |  | - | - |  | - | - | normal | normal |
| 464 | Pancreatic cancer | KRAS c.35G>T (p.Gly12Val), TP53 c.566_567insCCCT (p.Gln192fs) | - | - | KRAS c.35G>T (p.Gly12Val), TP53 c.566_567insCCCT (p.Gln192fs) | - | - | 2C.1 | 2C.1 |
| 465 | Lung cancer | BRAF c.1397G>T (p.Gly466Val), KRAS c.436G>C (p.Ala146Pro), STK11 c.182delG (p.Gly61fs) |  | - | BRAF c.1397G>T (p.Gly466Val), KRAS c.436G>C (p.Ala146Pro), STK11 c.182delG (p.Gly61fs) | - | - | 1A.2R | 1A.2R |
| 466 | Metastatic clear cell carcinoma |  | - | - |  | - | - | normal | normal |
| 467 | Pancreatic cancer | KRAS c.35G>A (p.Gly12Asp), PIK3CA c.331A>G (p.Lys111Glu), TP53 c.472C>G (p.Arg158Gly) | - | - | KRAS c.35G>A (p.Gly12Asp), PIK3CA c.331A>G (p.Lys111Glu), CDKN2A c.172C>T (p.Arg58Ter), TP53 c.472C>G (p.Arg158Gly) | - | - | 2C.1 | 2C.1 |
| 468 | Breast cancer | TP53 c.742C>T (p.Arg248Trp) | - | - | TP53 c.742C>T (p.Arg248Trp) | - | - | 2C.2 | 2C.2 |
| 469 | Vaginal cancer |  | - | - |  | - | - | normal | normal |
| 470 | Colon cancer | KRAS c.35G>T (p.Gly12Val) | - | - | KRAS c.35G>T (p.Gly12Val) | - | - | 1A.1R | 1A.1R |
| 471 | Pancreatic cancer | KRAS c.34G>C (p.Gly12Arg), TP53 c.743G>A (p.Arg248Gln) | - | - | KRAS c.34G>C (p.Gly12Arg), TP53 c.743G>A (p.Arg248Gln) | - | - | 2C.1 | 2C.1 |
| 472 | Lung cancer |  | - | - |  | - | - | normal | normal |
| 473 | Melanoma |  | - | - |  | - | - | normal | normal |
| 474 | Pancreatic cancer | KRAS c.35G>T (p.Gly12Val) | - | - | KRAS c.35G>T (p.Gly12Val) | - | - | 2C.1 | 2C.1 |
| 475 | Breast cancer |  |  | - |  | - | - | normal | normal |
| 476 | Gastric cancer |  | - | - |  | - | - | normal | normal |
| 477 | Colon cancer | KRAS c.35G>C (p.Gly12Ala), TP53 c.836_837delGG (p.Gly279fs) | - | - | KRAS c.35G>C (p.Gly12Ala), TP53 c.836_837delGG (p.Gly279fs) | - | - | 1A.1R | 1A.1R |
| 478 | Lung cancer |  | - | - |  | - | - | normal | normal |
| 479 | Liver cancer | TP53 c.452C>G (p.Pro151Arg) | - | - | TP53 c.452C>G (p.Pro151Arg) | - | - | 2C.2 | 2C.2 |
| 480 | Esophageal cancer |  | - | - |  | - | - | normal | normal |
| 481 | Endometrial cancer | FGFR2 c.755C>G (p.Ser252Trp) | - | - | FGFR2 c.755C>G (p.Ser252Trp) | - | - | 2C.1 | 2C.1 |
| 482 | Ovarian cancer | TP53 c.452C>G (p.Pro151Arg) | - | - | TP53 c.452C>G (p.Pro151Arg) | - | - | 2C.2 | 2C.2 |
| 483 | Breast cancer |  | ERBB2 Amplification | - |  | - | - | 1A.1 | 1A.1 |
| 484 | Teratoma |  | - | - |  | - | - | normal | normal |
| 485 | Lung cancer | TP53 c.801delG (p.Asn268fs) | - | - | TP53 c.801delG (p.Asn268fs) | - | - | 2C.2 | 2C.2 |
| 486 | Breast cancer | TP53 c.818G>A (p.Arg273His) | - | - | TP53 c.818G>A (p.Arg273His) | - | - | 2C.2 | 2C.2 |
| 487 | Pancreatic cancer | KRAS c.35G>A (p.Gly12Asp), TP53 c.578A>G (p.His193Arg) | - | - | KRAS c.35G>A (p.Gly12Asp), TP53 c.578A>G (p.His193Arg) | - | - | 2C.1 | 2C.1 |
| 488 | Endometrial cancer | PIK3CA c.1624G>A (p.Glu542Lys), AKT1 c.49G>A (p.Glu17Lys), CTNNB1 c.98C>G (p.Ser33Cys) | - | - | PIK3CA c.1624G>A (p.Glu542Lys), AKT1 c.49G>A (p.Glu17Lys), CTNNB1 c.98C>G (p.Ser33Cys) | - | - | 2C.1 | 2C.1 |
| 489 | Liver cancer |  | - | - |  | - | - | normal | normal |
| 490 | Gasrtric cancer | TP53 c.734G>A (p.Gly245Asp) | - | - | TP53 c.734G>A (p.Gly245Asp) | - | - | 2C.2 | 2C.2 |
| 491 | Pancreatic cancer | KRAS c.35G>A (p.Gly12Asp) | - | - | KRAS c.35G>A (p.Gly12Asp) | - | - | 2C.1 | 2C.1 |
| 492 | Colon cancer | KRAS c.34G>T (p.Gly12Cys), TP53 c.281C>A (p.Ser94Ter) | - | - | KRAS c.34G>T (p.Gly12Cys), TP53 c.281C>A (p.Ser94Ter) | - | - | 1A.1R | 1A.1R |
| 493 | Ovarian cancer | PIK3CA c.3140A>G (p.His1047Arg), TP53 c.1014_1018delCGAGA (p.Glu339fs) | - | - | PIK3CA c.3140A>G (p.His1047Arg), TP53 c.1014_1018delCGAGA (p.Glu339fs) | - | - | 2C.1 | 2C.1 |
| 494 | Colon cancer | TP53 c.487_488insCT (p.Tyr163fs) | KRAS Amplification, FGFR2 Amplification |  | TP53 c.487_488insCT (p.Tyr163fs) | KRAS Amplification, FGFR2 Amplification | - | 1A.1 | 1A.1 |
| 495 | Sarcoma | - | - | - | - | - | - | normal | normal |
| 496 | Prostate cancer | - | - | - | - | - | - | normal | normal |
| 497 | Endometrial cancer | - | - | - | - | - | - | normal | normal |
| 498 | Brain tumor | TP53 c.731G>A (p.Gly244Asp) | MET Amplification, PDGFRA AMPLIFICATION |  | TP53 c.731G>A (p.Gly244Asp) | MET Amplification, PDGFRA AMPLIFICATION | - | 2C.1 | 2C.1 |
| 499 | Pancreatic cancer | - | - | - |  | - | - | normal | normal |
| 500 | Pancreatic cancer | KRAS c.35G>T (p.Gly12Val) | - | - | KRAS c.35G>T (p.Gly12Val) | - | - | 2C.1 | 2C.1 |
| 501 | Pancreatic cancer | KRAS c.183A>C (p.Gln61His), TP53 c.524G>A (p.Arg175His) | ERBB2 Amplification | - | KRAS c.183A>C (p.Gln61His), TP53 c.524G>A (p.Arg175His) | ERBB2 Amplification | - | 2C.1 | 2C.1 |
| 502 | Angioblastoma | - | - | - | - | - | - | normal | normal |
| 503 | Gasrtric cancer | - | - | - | - | - | - | normal | normal |
| 504 | Lung cancer | - | - | - | - | - | - | normal | normal |
| 505 | Pancreatic cancer | - | - | - | - | - | - | normal | normal |
| 506 | Pancreatic cancer | KRAS c.35G>T (p.Gly12Val) | - | - | KRAS c.35G>T (p.Gly12Val) | - | - | 2C.1 | 2C.1 |
| 507 | Breast cancer | PIK3CA c.1633G>A (p.Glu545Lys) | - | - | PIK3CA c.1633G>A (p.Glu545Lys) | - | - | 1A.1 | 1A.1 |
| 508 | Prostate cancer | - | - | - | - | - | - | normal | normal |
| 509 | Gasrtric cancer | - | - | - | - | - | - | normal | normal |
| 510 | Colon cancer | KRAS c.35G>A (p.Gly12Asp) | - | - | KRAS c.35G>A (p.Gly12Asp) | - | - | 1A.1R | 1A.1R |
| 511 | Colon cancer | BRAF c.1799T>A (p.Val600Glu), TP53 c.394A>C (p.Lys132Gln) | - | - | BRAF c.1799T>A (p.Val600Glu), TP53 c.394A>C (p.Lys132Gln) | - | - | 1A.2 | 1A.2 |
| 512 | Colon cancer | KRAS c.35G>A (p.Gly12Asp), PIK3CA c.3140A>G (p.His1047Arg), TP53 c.586C>T (p.Arg196Ter) | - | - | KRAS c.35G>A (p.Gly12Asp), PIK3CA c.3140A>G (p.His1047Arg), TP53 c.586C>T (p.Arg196Ter) | - | - | 1A.1R | 1A.1R |
| 513 | Pancreatic cancer | KRAS c.34G>C (p.Gly12Arg) | - | - | KRAS c.34G>C (p.Gly12Arg) | - | - | 2C.1 | 2C.1 |
| 514 | Prostate cancer |  | - | - |  | - | - | normal | normal |
| 515 | Gastric cancer |  | - | - |  | - | - | normal | normal |
| 516 | Head and Neck cancer | HRAS c.182A>G (p.Gln61Arg), PIK3CA c.3140A>G (p.His1047Arg) | - | - | HRAS c.182A>G (p.Gln61Arg), PIK3CA c.3140A>G (p.His1047Arg) | - | - | 2C.1 | 2C.1 |
| 517 | Ovarian cancer | - | FGFR2 Amplification | - | - | FGFR2 Amplification | - | 2C.1 | 2C.1 |
| 518 | Prostate cancer | TP53 c.403T>C (p.Cys135Arg) | FGFR1 Amplification | - | - | FGFR1 Amplification | - | 2C.1 | 2C.1 |
| 519 | Colon cancer | - | - | - | - | - | - | 1A.1 | 1A.1 |
| 520 | Cholangiocarcinoma | - | - | - | IDH1 c.394C>T(p.Arg132Cys) | - | - | normal | 2C.1 |
| 521 | Pancreatic cancer | KRAS c.35G>A(p.Gly12Asp), TP53 c.742C>T(p.Arg248Trp) | - | - | KRAS c.35G>A(p.Gly12Asp), TP53 c.742C>T(p.Arg248Trp) | - | - | 2C.1 | 2C.1 |
| 522 | Breast cancer | PIK3CA c.1624G>A (p.Glu542Lys | - | - | PIK3CA c.1624G>A (p.Glu542Lys | - | - | 1A.1 | 1A.1 |
| 523 | Head and Neck cancer | PIK3CA c.1357G>A(p.Glu453Lys) | - | - | PIK3CA c.1357G>A(p.Glu453Lys) | - | - | 2C.1 | 2C.1 |
| 524 | Pancreatic cancer | KRAS c.35G>T(p.Gly12Val), TP53 c.526T>A( p.Cys176Ser) | - | - | KRAS c.35G>T(p.Gly12Val), TP53 c.526T>A( p.Cys176Ser) | - | - | 2C.1 | 2C.1 |
| 525 | Colon cancer | TP53 c.993G>T(p.Gln331His) | ERBB2 Amplification | - | TP53 c.993G>T(p.Gln331His) | ERBB2 Amplification | - | 1A.2 | 1A.2 |
| 526 | Pancreatic cancer | KRAS c.35G>A(p.Gly12Asp), TP53 c.226_227insG(p.Ala76fs) | - | - | KRAS c.35G>A(p.Gly12Asp), CDKN2A c.247C>T(p.His83Tyr), TP53 c.226_227insG(p.Ala76fs) | - | - | 2C.1 | 2C.1 |
| 527 | Breast cancer |  | - | - | PIK3CA c.1357G>A(p.Glu453Lys) | - | - | normal | 1A.1 |
| 528 | Lung cancer | SMAD4 c.1051G>C(p.Asp351His) | - | - | SMAD4 c.1051G>C(p.Asp351His) | - | - | 2C.2 | 2C.2 |
| 529 | Brain tumor |  | - | - |  | - | - | normal | normal |
| 530 | Colon cancer | KRAS c.38G>A (p.Gly13Asp), TP53 c.844C>T (p.Arg282Trp) | - | - | KRAS c.38G>A (p.Gly13Asp), TP53 c.844C>T (p.Arg282Trp) | - | - | 1A.1R | 1A.1R |
| 531 | Unknown | KRAS c.35G>A(p.Gly12Asp) | - | - | KRAS c.35G>A(p.Gly12Asp) | - | - | 2C.1 | 2C.1 |
| 532 | Gastric cancer | FBXW7 c.1393C>T(p.Arg465Cys | - | - | FBXW7 c.1393C>T(p.Arg465Cys | - | - | 2C.2 | 2C.2 |
| 533 | Cholangiocarcinoma | KRAS c.35G>T(p.Gly12Val), TP53 c.742C>T(p.Arg248Trp) | - | - | KRAS c.35G>T(p.Gly12Val), TP53 c.742C>T(p.Arg248Trp) | - | - | 2C.1 | 2C.1 |
| 534 | Sarcoma | - | - | - | - | - | - | normal | normal |
| 535 | Cholangiocarcinoma | - | - | - | - | - | - | normal | normal |
| 536 | Unknown Primary | - | - | - | - | - | - | normal | normal |
| 537 | Pancreatic cancer | - | - | - | - | - | - | normal | normal |
| 538 | Unknown Primary | - | - | - | - | - | - | normal | normal |
| 539 | Parotide gland cancer | - | - | - | - | - | - | normal | normal |
| 540 | Cholangiocarcinoma | - | - | - | - | - | - | normal | normal |
| 541 | Prostate cancer | TP53 c.524G>T (p.Arg175Leu) |  |  | TP53 c.524G>T (p.Arg175Leu) |  | - | normal | normal |
| 542 | Cholangiocarcinoma | - | - | - | - | - | - | normal | normal |
| 543 | Renal cancer | - | - | - | - | - | - | normal | normal |
| 544 | Pancreatic cancer | - | - | - | - | - | - | normal | normal |
| 545 | Breast cancer | PIK3CA c.3140A>G (p.His1047Arg) | - | - | PIK3CA c.3140A>G (p.His1047Arg) | - | - | 1A.1 | 1A.1 |
| 546 | kidney cancer | - | - | - | - | - | - | normal | normal |
| 547 | Gastric cancer | KRAS c.35G>A (p.Gly12Asp), PIK3CA c.3140A>G (p.His1047Arg) | - | - | KRAS c.35G>A (p.Gly12Asp), PIK3CA c.3140A>G (p.His1047Arg) |  | - | 2C.1 | 2C.1 |
| 548 | ADENOCARCINOMA OF THE CARDIOESOPHAGEAL JUNCTION |  | ERBB2 AMPLIFICATION |  |  | ERBB2 Amplification | - | 1A.1 | 1A.1 |
| 549 | Endometrial cancer | PIK3CA c.3140A>G (p.His1047Arg), CTNNB1 c.98C>T (p.Ser33Phe) | - | - | PIK3CA c.3140A>G (p.His1047Arg), CTNNB1 c.98C>T (p.Ser33Phe) | - | - | 2C.1 | 2C.1 |
| 550 | Breast cancer | PIK3CA c.3140A>G (p.His1047Arg) | - | - | PIK3CA c.3140A>G (p.His1047Arg) | - | - | 1A.1 | 1A.1 |
| 551 | ADRENAL CANCER | - | - | - |  | - | - | normal | normal |
| 552 | Ovarian cancer | - | - | - |  | - | - | normal | normal |
| 553 | Brain tumor | TP53 c.642_643delTA (p.His214fs) | - | - | TP53 c.642_643delTA (p.His214fs) | - | - | 2C.2 | 2C.2 |
| 554 | Breast cancer |  | - | - |  | - | - | normal | normal |
| 555 | Prostate cancer | PTEN c.716_719delTGTA (p.Met239fs), TP53 c.1031T>C (p.Leu344Pro | - | - | PTEN c.716_719delTGTA (p.Met239fs), TP53 c.1031T>C (p.Leu344Pro | - | - | 2C.1 | 2C.1 |
| 556 | Brain tumor | PTEN c.203A>G (p.Tyr68Cys) | - | - | PTEN c.203A>G (p.Tyr68Cys), EGFR c.323G>A (p.Arg108Lys) | - | - | 2C.1 | 2C.1 |
| 557 | Breast cancer | - | - | - |  | - | - | normal | normal |
| 558 | kidney cancer | TP53 c.642_643delTA (p.His214fs) | - | - | TP53 c.642_643delTA (p.His214fs) | - | - | 2C.2 | 2C.2 |
| 559 | Cholangiocarcinoma |  | - | - | IDH1 c.394C>T (p.Arg132Cys) | - | - | normal | 2C.1 |
| 560 | ╬ΧWING SARCOMA |  | - | - |  | - | - | normal | normal |
| 561 | Pancreatic cancer | KRAS c.35G>T (p.Gly12Val) | - | - | KRAS c.35G>T (p.Gly12Val) | - | - | 2C.1 | 2C.1 |
| 562 | Pancreatic cancer | KRAS c.35G>A (p.Gly12Asp | - | - | KRAS c.35G>A (p.Gly12Asp | - | - | 2C.1 | 2C.1 |
| 563 | NSCLC |  | - | - |  | - | - | normal | normal |
| 564 | Pancreatic cancer | KRAS c.35G>A (p.Gly12Asp, TP53 c.586C>T (p.Arg196Ter) | - | - | KRAS c.35G>A (p.Gly12Asp, TP53 c.586C>T (p.Arg196Ter) | - | - | 2C.1 | 2C.1 |
| 565 | Breast cancer |  | - | - |  | - | - | normal | normal |
| 566 | VULVAR CANCER | TP53 c.949C>T (p.Gln317Ter) | - | - | TP53 c.949C>T (p.Gln317Ter) | - | - | 2C.2 | 2C.2 |
| 567 | Pancreatic cancer | KRAS c.35G>T (p.Gly12Val), TP53 c.577C>T (p.His193Tyr) | - | - | KRAS c.35G>T (p.Gly12Val), TP53 c.577C>T (p.His193Tyr) | - | - | 2C.1 | 2C.1 |
| 568 | Gastric cancer | TP53 c.745A>T (p.Arg249Trp) | - | - | TP53 c.745A>T (p.Arg249Trp) | - | - | 2C.2 | 2C.2 |
| 569 | Pancreatic cancer |  | - | - |  | - | - | normal | normal |
| 570 | Sarcoma |  | - | - |  | - | - | normal | normal |
| 571 | Pancreatic cancer | KRASExon 2 c.34G>C (p.Gly12Arg) | - | - | KRASExon 2 c.34G>C (p.Gly12Arg) | - | - | 2C.1 | 2C.1 |
| 572 | Cholangiocarcinoma |  | - | - |  | - | - | normal | normal |
| 573 | Ampullary cancer | KRAS Exon 3 c.183A>T (p.Gln61His) | - | - | KRAS Exon 3 c.183A>T (p.Gln61His) | - | - | 2C.1 | 2C.1 |
| 574 | Breast cancer |  | - | - |  | - | - | normal | normal |
| 575 | Unknown Primary | NRAS Exon 3 c.181C>A (p.Gln61Lys) | - | - | NRAS Exon 3 c.181C>A (p.Gln61Lys) | - | - | 2C.1 | 2C.1 |
| 576 | SALIVARY GLAND CARCINOMA |  | - | - |  | - | - | normal | normal |
| 577 | Endometrial cancer | TP53 | - | - | TP53 | - | - | 2C.2 | 2C.2 |
| 578 | Gastric cancer | TP53 | - | - | TP53 | - | - | 2C.2 | 2C.2 |
| 579 | Unknown primary |  | - | - |  | - | - | normal | normal |
| 580 | LEIOMYOSARCOMA |  | - | - |  | - | - | 2C.1 | 2C.1 |
| 581 | Breast cancer |  | - | - |  | - | - | normal | normal |
| 582 | Pancreatic cancer |  | - | - |  | - | - | normal | normal |
| 583 | Desmoplastic small round cell tumor |  | - | - |  | - | - | normal | normal |
| 584 | Sarcoma | TP53 | - | - | TP53 | - | - | 2C.2 | 2C.2 |
| 585 | Breast cancer |  | - | - | PIK3CA c.1035T>A (p.Asn345Lys) | - | - | normal | 1A.1 |
| 586 | Pancreatic cancer | KRAS c.35G>T (p.Gly12Val) | - | - | KRAS c.35G>T (p.Gly12Val) | - | - | 2C.1 | 2C.1 |
| 587 | Penile cancer |  | - | - |  | - | - | normal | normal |
| 588 | Prostate cancer | KRAS c.35G>A, (p.Gly12Asp), TP53 c.799C>T (p.Arg267Trp) | - | - | KRAS c.35G>A, (p.Gly12Asp), PIK3CA c.263G>A (p.Arg88Gln), TP53 c.799C>T (p.Arg267Trp) | - | - | 2C.1 | 2C.1 |
| 589 | Signet ring cell carcinoma | BRAF c.1397G>A (p.Gly466Glu), | - | - | BRAF c.1397G>A (p.Gly466Glu), | - | - | 2C.2 | 2C.2 |
| 590 | Cholangiocarcinoma |  | - | - | CDKN2A c.341C>A (p.Pro114His) | - | - | normal | 2D |
| 591 | Pancreatic cancer |  | - | - |  | - | - | normal | normal |
| 592 | Parotid gland CANCER | TP53 c.537T>A (p.His179Gln) | - | - | TP53 c.537T>A (p.His179Gln) | - | - | 2C.2 | 2C.2 |
| 593 | Prostate cancer |  | - | - |  | - | - | normal | normal |
| 594 | Sarcoma |  | - | - |  | - | - | normal | normal |
| 595 | Ovarian cancer | PIK3CA c.1635G>T (p.Glu545Asp) | - | - | PIK3CA c.1635G>T (p.Glu545Asp) | - | - | 2C.1 | 2C.1 |
| 596 | Breast cancer |  | - | - |  | - | - | normal | normal |
| 597 | Prostate cancer |  | - | - |  | - | - | normal | normal |
| 598 | Endometrial cancer | PTEN c.389G>A (p.Arg130Gln, TP53 c.702C>A (p.Tyr234Ter | - | - | PTEN c.389G>A (p.Arg130Gln, TP53 c.702C>A (p.Tyr234Ter | - | - | 2C.2 | 2C.2 |
| 599 | Pancreatic cancer | KRAS c.35G>T(p.Gly12Val) | - | - | KRAS c.35G>T(p.Gly12Val) | - | - | 2C.1 | 2C.1 |
| 600 | Pancreatic cancer | KRAS c.35G>T(p.Gly12Val), TP53 c.524G>A (p.Arg175His) | - | - | KRAS c.35G>T(p.Gly12Val), TP53 c.524G>A (p.Arg175His) | - | - | 2C.1 | 2C.1 |
| 601 | Esophageal Cancer |  | - | - |  | - | - | normal | normal |
| 602 | GASTRIC CANCER | KRAS c.35G>A (p.Gly12Asp) | - | - | KRAS c.35G>A (p.Gly12Asp) | - | - | 2C.1 | 2C.1 |
| 603 | SOFT TISSUE SARCOMA |  | - | - |  | - | - | normal | normal |
| 604 | LEIOMYOSARCOMA |  | - | - |  | - | - | normal | normal |
| 605 | THYMUS CELL CARCINOMA |  | - | - |  | - | - | normal | normal |
| 606 | HEAD AND NECK CANCER |  | - | - |  | - | - | normal | normal |
| 607 | SARCOMA |  | - | - |  | - | - | normal | normal |
| 608 | PAROTID GLAND CANCER | TP53 C. .537T>A (p.His179Gln) | - | - | TP53 C. .537T>A (p.His179Gln) | - | - | 2C.2 | 2C.2 |
| 609 | ANGIOSARCOMA | PIK3CA c.1624G>A p.Glu542Lys, TP53 c.1024C>T p.Arg342Ter | - | - | PIK3CA c.1624G>A p.Glu542Lys, TP53 c.1024C>T p.Arg342Ter | - | - | 2C.1 | 2C.1 |
| 610 | PROSTATE CANCER |  | - | - |  |  | - | normal | normal |

Additional file 10: Table S6 Simulation results of the alterations that would have been identified if the gene set of the 161 gene NGS panel was used in the PCAWG samples.

| **sampleID** | **tumor_type1** | **TUMOR_TYPE2** | **mut loe** | **max MUT loe** | **GENE CNV** | **MAX LoE CNV** | **fusion LoE** | **MAX loe FUS** | **MAX LoE** |
| --- | --- | --- | --- | --- | --- | --- | --- | --- | --- |
| SP1003 | Bladder | OTHER | ARID1A p.Q1095* 2C.2 , MLH1 p.E754Q 1B , TP53 p.R248W 2C.2 | 1B |  | NL |  | NL | 1B |
| SP10084 | Breast | BREAST | TP53 p.R248Q 2C.2 | 2C.2 | CCND1 amp 2C.2 , FGF19 amp 2D , FGF3 amp 2D , FGFR1 amp 2C.1 , MYC amp 2C.2 | 2C.1 |  | NL | 2C.1 |
| SP1009 | Bladder | OTHER | ARID1A p.D1850Gfs*4 2C.2 , NFE2L2 p.G31A 3 , SF3B1 p.R625C 3 | 2C.2 |  | NL |  | NL | 2C.2 |
| SP10150 | Breast | BREAST | CDKN1B p.T135Lfs*10 2D , PIK3CA p.H1047R 1A.1 , TP53 p.C238Y 2C.2 | 1A.1 | MYC amp 2C.2 | 2C.2 |  | NL | 1A.1 |
| SP101515 | Ovary | OVARY | TP53 p.H168R 2C.2 | 2C.2 |  | NL |  | NL | 2C.2 |
| SP101519 | Ovary | OVARY | TP53 p.V272M 2C.2 | 2C.2 | CCNE1 amp 2C.2 , CCNE1 amp 2C.2 | 2C.2 |  | NL | 2C.2 |
| SP101521 | Ovary | OVARY | TP53 p.H179R 2C.2 | 2C.2 | CCNE1 amp 2C.2 | 2C.2 |  | NL | 2C.2 |
| SP101523 | Ovary | OVARY | TP53 p.A138P 2C.2 | 2C.2 |  | NL |  | NL | 2C.2 |
| SP101526 | Ovary | OVARY | TP53 p.R273H 2C.2 | 2C.2 | AKT2 amp 2C.2 , AKT3 loss 2C.2 , AXL amp 2C.2 , CCNE1 amp 2C.2 | 2C.2 |  | NL | 2C.2 |
| SP101528 | Ovary | OVARY | TP53 p.R282W 2C.2 | 2C.2 | CCND2 amp 2C.2 | 2C.2 |  | NL | 2C.2 |
| SP101532 | Ovary | OVARY | TP53 p.A161T 2C.2 | 2C.2 |  | NL |  | NL | 2C.2 |
| SP101536 | Ovary | OVARY | TP53 p.R273H 2C.2 | 2C.2 |  | NL |  | NL | 2C.2 |
| SP101540 | Ovary | OVARY | TP53 p.A138V 2C.2 | 2C.2 | AKT2 amp 2C.2 , CCNE1 amp 2C.2 , MYC amp 2C.2 | 2C.2 |  | NL | 2C.2 |
| SP101544 | Ovary | OVARY | TP53 p.R273H 2C.2 | 2C.2 | CCNE1 amp 2C.2 , KRAS amp 2C.1 | 2C.1 | ESR1 fusion 2C.1 | 2C.1 | 2C.1 |
| SP101548 | Ovary | OVARY | BRCA2 p.K1453* 1A.1 , RNF43 p.L311Sfs*108 2C.2 | 1A.1 |  | NL |  | NL | 1A.1 |
| SP101552 | Ovary | OVARY | TP53 p.T304Efs*39 2C.2 | 2C.2 |  | NL |  | NL | 2C.2 |
| SP101558 | Ovary | OVARY | TP53 p.W91* 2C.2 | 2C.2 |  | NL |  | NL | 2C.2 |
| SP101564 | Ovary | OVARY | RB1 p.S397* 2C.2 , TP53 p.R249G 2C.2 | 2C.2 |  | NL |  | NL | 2C.2 |
| SP101572 | Ovary | OVARY | TP53 p.C124Wfs*25 2C.2 | 2C.2 | AKT2 amp 2C.2 , CCNE1 amp 2C.2 , ERBB2 amp 2C.1 , MYC amp 2C.2 , PIK3CA loss 2C.1 , PIK3CB loss 2C.1 | 2C.1 |  | NL | 2C.1 |
| SP101576 | Ovary | OVARY | FGFR4 p.R248Q 2C.1 , TP53 p.V172F 2C.2 | 2C.1 | CCNE1 amp 2C.2 , PIK3CB loss 2C.1 | 2C.1 |  | NL | 2C.1 |
| SP101580 | Ovary | OVARY | BRCA1 p.N1259Efs*2 1A.1 , NF1 p.Q1174* 2C.1 , TP53 p.R248Q 2C.2 | 1A.1 | AKT3 loss 2C.2 , MYC amp 2C.2 | 2C.2 |  | NL | 1A.1 |
| SP101584 | Ovary | OVARY | TP53 p.I195T 2C.2 | 2C.2 | CCND2 amp 2C.2 | 2C.2 |  | NL | 2C.2 |
| SP101588 | Ovary | OVARY | TP53 p.R273L 2C.2 | 2C.2 | AKT2 amp 2C.2 | 2C.2 |  | NL | 2C.2 |
| SP101592 | Ovary | OVARY | TP53 . 2C.2 | 2C.2 | AKT2 amp 2C.2 , CCND2 amp 2C.2 , KRAS amp 2C.1 , MYC amp 2C.2 | 2C.1 |  | NL | 2C.1 |
| SP101596 | Ovary | OVARY | TP53 p.Y163C 2C.2 | 2C.2 | AKT2 amp 2C.2 , RICTOR loss 2C.2 , TERT loss 3 | 2C.2 |  | NL | 2C.2 |
| SP101600 | Ovary | OVARY |  | NL |  | NL |  | NL | NL |
| SP101604 | Ovary | OVARY | BRCA1 p.V1176Ffs*34 1A.1 , TP53 p.E286K 2C.2 | 1A.1 |  | NL |  | NL | 1A.1 |
| SP101610 | Ovary | OVARY | TP53 p.A86Lfs*38 2C.2 | 2C.2 |  | NL |  | NL | 2C.2 |
| SP101616 | Ovary | OVARY | SMARCB1 p.R201Q 2D , TP53 p.L130H 2C.2 | 2C.2 |  | NL |  | NL | 2C.2 |
| SP101622 | Ovary | OVARY | TP53 p.W146* 2C.2 | 2C.2 | PIK3CA loss 2C.1 | 2C.1 |  | NL | 2C.1 |
| SP101628 | Ovary | OVARY | TP53 p.V172F 2C.2 | 2C.2 | PIK3CB loss 2C.1 | 2C.1 |  | NL | 2C.1 |
| SP101634 | Ovary | OVARY | TP53 p.Y220C 2C.2 | 2C.2 |  | NL |  | NL | 2C.2 |
| SP101642 | Ovary | OVARY | TP53 p.R342* 2C.2 | 2C.2 |  | NL |  | NL | 2C.2 |
| SP101648 | Ovary | OVARY | NF1 p.L2279* 2C.1 , TP53 p.S241F 2C.2 | 2C.1 | MYC amp 2C.2 | 2C.2 |  | NL | 2C.1 |
| SP101654 | Ovary | OVARY | TP53 p.C124* 2C.2 | 2C.2 | RICTOR loss 2C.2 , TERT loss 3 | 2C.2 | ESR1 fusion 2C.1 | 2C.1 | 2C.1 |
| SP101658 | Ovary | OVARY | TP53 p.V218delV 2C.2 | 2C.2 |  | NL |  | NL | 2C.2 |
| SP101662 | Ovary | OVARY | TP53 p.Y205Wfs*41 2C.2 | 2C.2 |  | NL |  | NL | 2C.2 |
| SP101666 | Ovary | OVARY | TP53 p.E343Gfs*2 2C.2 | 2C.2 |  | NL |  | NL | 2C.2 |
| SP101670 | Ovary | OVARY | TP53 p.R273H 2C.2 | 2C.2 |  | NL |  | NL | 2C.2 |
| SP101674 | Ovary | OVARY | TP53 p.K291* 2C.2 | 2C.2 | MYC amp 2C.2 | 2C.2 |  | NL | 2C.2 |
| SP101678 | Ovary | OVARY | TP53 p.D148_S149insYTP 2C.2 | 2C.2 | MYC amp 2C.2 | 2C.2 |  | NL | 2C.2 |
| SP101686 | Ovary | OVARY | TP53 p.M237I 2C.2 | 2C.2 | AKT2 amp 2C.2 , CCNE1 amp 2C.2 | 2C.2 | AKT2 fusion 3 | 3 | 2C.2 |
| SP101690 | Ovary | OVARY | TP53 p.S96Lfs*27 2C.2 | 2C.2 | CCNE1 amp 2C.2 , EGFR loss 2C.1 , PIK3CA loss 2C.1 | 2C.1 |  | NL | 2C.1 |
| SP101694 | Ovary | OVARY | TP53 p.K132R 2C.2 | 2C.2 | MDM4 loss 2D , NTRK1 loss 2C.1 | 2C.1 |  | NL | 2C.1 |
| SP101700 | Ovary | OVARY | TP53 p.P278R 2C.2 | 2C.2 |  | NL |  | NL | 2C.2 |
| SP101708 | Ovary | OVARY | PALB2 p.R34H 2C.1 , TP53 p.R175H 2C.2 | 2C.1 | CCNE1 amp 2C.2 | 2C.2 | NF1 fusion 2C.1 | 2C.1 | 2C.1 |
| SP101716 | Ovary | OVARY | FBXW7 p.L290V 2C.2 , NOTCH1 p.D545V 2C.2 , TP53 p.Y220C 2C.2 | 2C.2 | CCNE1 amp 2C.2 | 2C.2 |  | NL | 2C.2 |
| SP101724 | Ovary | OVARY | CDK12 p.R1048* 2C.1 , TP53 p.E294* 2C.2 | 2C.1 | CCNE1 amp 2C.2 , ESR1 loss 2C.2 , TERT loss 3 | 2C.2 |  | NL | 2C.1 |
| SP101732 | Ovary | OVARY | RB1 . 2C.2 , TP53 p.R175H 2C.2 | 2C.2 | AKT1 amp 2C.2 | 2C.2 |  | NL | 2C.2 |
| SP101740 | Ovary | OVARY | TP53 p.R158P 2C.2 | 2C.2 |  | NL |  | NL | 2C.2 |
| SP101795 | Ovary | OVARY | TP53 p.Q192* 2C.2 | 2C.2 |  | NL |  | NL | 2C.2 |
| SP101881 | Ovary | OVARY | TP53 p.S240Mfs*25 2C.2 | 2C.2 |  | NL |  | NL | 2C.2 |
| SP101891 | Ovary | OVARY | ATRX p.K869Ifs*2 2C.2 , TP53 p.R248Q 2C.2 | 2C.2 | MYC amp 2C.2 | 2C.2 |  | NL | 2C.2 |
| SP101921 | Ovary | OVARY | NF2 p.R262* 2C.1 | 2C.1 |  | NL |  | NL | 2C.1 |
| SP101931 | Ovary | OVARY | TP53 p.C176G 2C.2 | 2C.2 | ESR1 loss 2C.2 , ESR1 loss 2C.2 , KRAS amp 2C.1 | 2C.1 |  | NL | 2C.1 |
| SP102015 | Ovary | OVARY | FGFR4 p.G176C 2C.1 , TP53 p.I195T 2C.2 | 2C.1 | FGFR3 loss 2C.1 , MYC amp 2C.2 | 2C.1 |  | NL | 2C.1 |
| SP102035 | Ovary | OVARY | TP53 p.H179Q 2C.2 | 2C.2 |  | NL |  | NL | 2C.2 |
| SP102045 | Ovary | OVARY | BRCA2 p.M1I 1A.1 | 1A.1 | NTRK3 amp 2C.1 , PIK3CA loss 2C.1 | 2C.1 |  | NL | 1A.1 |
| SP102055 | Ovary | OVARY | TP53 p.R249Sfs*96 2C.2 | 2C.2 |  | NL |  | NL | 2C.2 |
| SP102064 | Ovary | OVARY | TP53 p.Y220C 2C.2 | 2C.2 |  | NL |  | NL | 2C.2 |
| SP102074 | Ovary | OVARY | TP53 p.F113V 2C.2 , TP53 p.F113C 2C.2 | 2C.2 |  | NL |  | NL | 2C.2 |
| SP102084 | Ovary | OVARY | TP53 p.C135F 2C.2 | 2C.2 |  | NL |  | NL | 2C.2 |
| SP102090 | Ovary | OVARY | CDK12 p.R112S 2C.1 , TP53 p.R273H 2C.2 | 2C.1 |  | NL |  | NL | 2C.1 |
| SP102096 | Ovary | OVARY | TP53 p.P85Lfs*38 2C.2 | 2C.2 | CCNE1 amp 2C.2 , MYC amp 2C.2 | 2C.2 |  | NL | 2C.2 |
| SP102103 | Ovary | OVARY | TP53 p.G245D 2C.2 | 2C.2 | CCNE1 amp 2C.2 | 2C.2 |  | NL | 2C.2 |
| SP102113 | Ovary | OVARY | KRAS p.G12V 2C.1 , TP53 p.Q192* 2C.2 | 2C.1 |  | NL |  | NL | 2C.1 |
| SP102123 | Ovary | OVARY | AKT1 p.R249W 2C.2 , TP53 p.E339Rfs*6 2C.2 | 2C.2 | PIK3CA loss 2C.1 | 2C.1 |  | NL | 2C.1 |
| SP102133 | Ovary | OVARY | ARID1A p.E38Rfs*73 2C.2 , CDK12 p.D494N 2C.1 , TP53 p.H168P 2C.2 | 2C.1 | CCNE1 amp 2C.2 | 2C.2 |  | NL | 2C.1 |
| SP102143 | Ovary | OVARY | TP53 p.R280Kfs*26 2C.2 | 2C.2 |  | NL |  | NL | 2C.2 |
| SP102161 | Ovary | OVARY | RB1 p.E204Kfs*10 2C.2 , TP53 p.I195T 2C.2 | 2C.2 |  | NL |  | NL | 2C.2 |
| SP102168 | Ovary | OVARY | CDK12 p.G239* 2C.1 , TP53 p.I255F 2C.2 | 2C.1 |  | NL |  | NL | 2C.1 |
| SP102174 | Ovary | OVARY | TP53 p.H193R 2C.2 | 2C.2 | AR amp 2C.2 , CCND3 loss 2C.2 , ERBB2 amp 2C.1 , ESR1 loss 2C.2 , ESR1 loss 2C.2 , FGFR3 loss 2C.1 , MYC amp 2C.2 , PIK3CA loss 2C.1 , PIK3CB loss 2C.1 | 2C.1 | ESR1 fusion 2C.1 | 2C.1 | 2C.1 |
| SP102187 | Ovary | OVARY | BRCA1 p.Q1467* 1A.1 , PALB2 p.S518* 2C.1 , TP53 p.M237I 2C.2 | 1A.1 |  | NL |  | NL | 1A.1 |
| SP102690 | Prostate | PROSTATE |  | NL |  | NL |  | NL | NL |
| SP103866 | Skin | SKIN | IDH1 p.R132C 2C.1 , KDR p.G1063E 2D , KRAS p.Q61R 2C.1 , KRAS p.E62K 2C.1 , NOTCH2 p.P2219S 2C.2 , RB1 p.Q257* 2C.2 | 2C.1 |  | NL |  | NL | 2C.1 |
| SP103894 | Skin | SKIN | NRAS p.Q61K 2C.1 , NTRK2 p.P295S 2C.1 | 2C.1 | MDM2 amp 2C.2 | 2C.2 |  | NL | 2C.1 |
| SP104056 | Skin | SKIN | ERBB4 p.P800L 2C.2 , NF1 p.R1870Q 2C.1 , SETD2 p.Q757* 2D , TP53 p.R213Q 2C.2 | 2C.1 |  | NL |  | NL | 2C.1 |
| SP104530 | Skin | SKIN | BRAF p.V600E 1A.1 , CDK4 p.R24S 2C.1 , NTRK1 p.Q660L 2C.1 , PIK3CA p.E545K 2C.1 | 1A.1 | BRAF loss 2C.1 | 2C.1 |  | NL | 1A.1 |
| SP10470 | Breast | BREAST | PIK3CA p.R88Q 1A.1 , TP53 p.G108Vfs*15 2C.2 | 1A.1 | ESR1 loss 2C.2 , PIK3CA loss 2C.1 | 2C.1 |  | NL | 1A.1 |
| SP104984 | Stomach | OTHER | TP53 p.R306* 2C.2 | 2C.2 |  | NL |  | NL | 2C.2 |
| SP105006 | Stomach | OTHER | ARID1A p.P1379Lfs*102 2C.2 , CTNNB1 p.G34R 2C.2 | 2C.2 | ERBB2 amp 1A.1 , ERBB2 amp 1A.1 | 1A.1 |  | NL | 1A.1 |
| SP105018 | Stomach | OTHER | BRCA2 p.P1088Lfs*16 2C.1 , NOTCH2 p.D1733H 2C.2 , SMAD4 p.G352A 2C.2 | 2C.1 |  | NL |  | NL | 2C.1 |
| SP105086 | Stomach | OTHER | CREBBP p.G1542S 2C.2 , CTNNB1 p.T41I 2C.2 , ERBB2 p.D769Y 2C.1 , RNF43 p.R371* 2C.2 | 2C.1 |  | NL |  | NL | 2C.1 |
| SP105159 | Stomach | OTHER | ERBB2 p.D769Y 2C.1 , ERBB3 p.K314T 2C.2 , PIK3CB p.E1047K 2C.1 , PIK3CB p.A1048V 2C.1 , PTEN p.D24E 2C.1 | 2C.1 |  | NL |  | NL | 2C.1 |
| SP105213 | Stomach | OTHER | ARID1A p.Q176* 2C.2 , PIK3CA p.N345K 2C.1 | 2C.1 |  | NL |  | NL | 2C.1 |
| SP105253 | Stomach | OTHER | TP53 p.F134C 2C.2 | 2C.2 |  | NL |  | NL | 2C.2 |
| SP105261 | Stomach | OTHER | TP53 p.P98Afs*51 2C.2 | 2C.2 |  | NL |  | NL | 2C.2 |
| SP105577 | Stomach | OTHER | ARID1A p.V1491Afs*9 2C.2 , PIK3CA p.G118D 2C.1 | 2C.1 |  | NL |  | NL | 2C.1 |
| SP10563 | Breast | BREAST | PIK3CA p.E545K 1A.1 | 1A.1 | ERBB2 amp 1A.1 , MDM4 loss 2D , MYC amp 2C.2 | 1A.1 |  | NL | 1A.1 |
| SP105673 | Stomach | OTHER | PTEN p.Y346*fs*1 2C.1 | 2C.1 |  | NL |  | NL | 2C.1 |
| SP105708 | Thyroid | OTHER |  | NL |  | NL |  | NL | NL |
| SP105759 | Thyroid | OTHER |  | NL |  | NL |  | NL | NL |
| SP105807 | Thyroid | OTHER |  | NL |  | NL |  | NL | NL |
| SP1059 | Bladder | OTHER | ATR p.A1291V 2C.2 , FGFR3 p.S249C 1A.1 , SRC p.R391C 2C.2 | 1A.1 | CCND1 amp 2C.2 , FGF19 amp 2D , FGF3 amp 2D , MDM2 amp 2C.2 , MYC amp 2C.2 , RICTOR loss 2C.2 | 2C.2 |  | NL | 1A.1 |
| SP105922 | Thyroid | OTHER |  | NL |  | NL |  | NL | NL |
| SP10635 | Breast | BREAST |  | NL |  | NL |  | NL | NL |
| SP106560 | Kidney | OTHER | BAP1 p.P324Hfs*11 2C.1 | 2C.1 |  | NL |  | NL | 2C.1 |
| SP106577 | Kidney | OTHER | SMARCA4 p.K991E 2C.2 | 2C.2 |  | NL |  | NL | 2C.2 |
| SP106602 | Kidney | OTHER |  | NL |  | NL |  | NL | NL |
| SP106631 | Liver | OTHER | BAP1 p.F170V 2C.1 | 2C.1 |  | NL |  | NL | 2C.1 |
| SP106638 | Kidney | OTHER | FBXW7 p.R393Efs*2 2C.2 , MET p.M1268T 2C.1 | 2C.1 |  | NL |  | NL | 2C.1 |
| SP106656 | Kidney | OTHER |  | NL |  | NL |  | NL | NL |
| SP106677 | Liver | OTHER | TP53 p.V157G 2C.2 | 2C.2 | AKT3 loss 2C.2 , NTRK1 loss 2C.1 | 2C.1 |  | NL | 2C.1 |
| SP106743 | Liver | OTHER | KRAS p.R68M 2C.1 | 2C.1 | AR amp 2C.2 | 2C.2 |  | NL | 2C.1 |
| SP107575 | Cervix | OTHER | STK11 p.T185Sfs*97 2C.1 | 2C.1 |  | NL |  | NL | 2C.1 |
| SP107595 | Cervix | OTHER |  | NL |  | NL |  | NL | NL |
| SP107603 | Cervix | OTHER | NOTCH1 p.H2018Lfs*9 2C.2 , STK11 p.D53*fs*1 2C.1 | 2C.1 |  | NL |  | NL | 2C.1 |
| SP107607 | Cervix | OTHER |  | NL |  | NL |  | NL | NL |
| SP107624 | Cervix | OTHER |  | NL |  | NL |  | NL | NL |
| SP107640 | Cervix | OTHER | FBXW7 p.R479P 2C.2 , PIK3CA p.E545Q 2C.1 | 2C.1 |  | NL |  | NL | 2C.1 |
| SP107650 | Cervix | OTHER | FBXW7 p.D600N 2C.2 | 2C.2 |  | NL |  | NL | 2C.2 |
| SP1086 | Bladder | OTHER | TP53 p.Y220C 2C.2 | 2C.2 | KRAS amp 2C.1 | 2C.1 |  | NL | 2C.1 |
| SP109301 | Liver | OTHER | ARID1A p.Q1974Tfs*43 2C.2 , CTNNB1 p.S37F 2C.2 , TP53 p.Q192* 2C.2 | 2C.2 |  | NL |  | NL | 2C.2 |
| SP109384 | Liver | OTHER |  | NL |  | NL |  | NL | NL |
| SP10944 | Breast | BREAST | MRE11A p.E451Q 2C.1 , TP53 p.E285Rfs*54 2C.2 | 2C.1 | CCND1 amp 2C.2 , EGFR loss 2C.1 , ERBB2 amp 1A.1 , FGF19 amp 2D , FGF3 amp 2D , FGFR1 amp 2C.1 , MDM4 loss 2D , MYC amp 2C.2 , NTRK1 loss 2C.1 , PDGFRB loss 2C.1 , PIK3CA loss 2C.1 , PPARG loss 3 | 1A.1 | FGFR1 fusion 2C.1 | 2C.1 | 1A.1 |
| SP109457 | Kidney | OTHER | SMARCA4 p.E821K 2C.2 | 2C.2 |  | NL |  | NL | 2C.2 |
| SP109470 | Kidney | OTHER | NF2 p.P134H 2C.1 , NFE2L2 p.T80K 3 , SMARCB1 p.T357*fs*1 2D | 2C.1 |  | NL |  | NL | 2C.1 |
| SP109478 | Kidney | OTHER |  | NL |  | NL |  | NL | NL |
| SP109544 | Kidney | OTHER | ARID1A p.P1876Qfs*7 2C.2 , CREBBP p.S32L 2C.2 | 2C.2 |  | NL |  | NL | 2C.2 |
| SP109649 | Prostate | PROSTATE |  | NL |  | NL |  | NL | NL |
| SP109801 | Cervix | OTHER | CDKN2A p.D146G 2C.1 | 2C.1 | PIK3CA loss 2C.1 , PIK3CB loss 2C.1 , RICTOR loss 2C.2 , TERT loss 3 | 2C.1 |  | NL | 2C.1 |
| SP109941 | Cervix | OTHER |  | NL |  | NL |  | NL | NL |
| SP109953 | Cervix | OTHER | FBXW7 p.R465L 2C.2 , KRAS p.G12D 2C.1 | 2C.1 |  | NL |  | NL | 2C.1 |
| SP109957 | Cervix | OTHER | ERBB2 p.S974F 2C.1 | 2C.1 | PIK3CA loss 2C.1 | 2C.1 |  | NL | 2C.1 |
| SP11045 | Breast | BREAST |  | NL | ERBB2 amp 1A.1 | 1A.1 |  | NL | 1A.1 |
| SP110847 | Esophagus | OTHER | TP53 p.V173M 2C.2 | 2C.2 |  | NL |  | NL | 2C.2 |
| SP110849 | Esophagus | OTHER | KRAS p.G13D 2C.1 , PIK3CA p.E542K 2C.1 , SMAD4 p.P356S 2C.2 , TP53 p.C176F 2C.2 | 2C.1 |  | NL |  | NL | 2C.1 |
| SP111024 | Esophagus | OTHER | TP53 p.R306* 2C.2 | 2C.2 | ERBB2 amp 2C.1 | 2C.1 |  | NL | 2C.1 |
| SP111070 | Esophagus | OTHER | ARID1A p.S90Pfs*11 2C.2 , CTNNB1 p.S37F 2C.2 , SMARCA4 p.E920K 2C.2 | 2C.2 |  | NL |  | NL | 2C.2 |
| SP111095 | Esophagus | OTHER | ERBB3 p.E928G 2C.2 , TP53 p.K132N 2C.2 | 2C.2 | BRAF loss 2C.1 , KRAS amp 2C.1 | 2C.1 |  | NL | 2C.1 |
| SP111099 | Esophagus | OTHER | TP53 p.V173M 2C.2 | 2C.2 |  | NL |  | NL | 2C.2 |
| SP111175 | Esophagus | OTHER | ATM p.S1455Vfs*3 2C.1 , NOTCH2 p.R2453W 2C.2 , TP53 p.R273H 2C.2 | 2C.1 |  | NL |  | NL | 2C.1 |
| SP1114 | Bladder | OTHER | ERBB3 p.V104L 2C.2 , FGFR3 p.S249C 1A.1 , TSC2 p.E656* 2C.1 | 1A.1 |  | NL |  | NL | 1A.1 |
| SP11171 | Breast | BREAST | TP53 p.L111P 2C.2 | 2C.2 |  | NL |  | NL | 2C.2 |
| SP11235 | Breast | BREAST |  | NL |  | NL |  | NL | NL |
| SP11292 | Breast | BREAST | TP53 p.P278T 2C.2 | 2C.2 |  | NL |  | NL | 2C.2 |
| SP113197 | Skin | SKIN |  | NL | CCND1 amp 2C.2 , FGF19 amp 2D , FGF3 amp 2D , FGF3 amp 2D | 2C.2 |  | NL | 2C.2 |
| SP1132 | Bladder | OTHER | ARID1A p.G55Afs*44 2C.2 , ERBB3 p.H228Q 2C.2 , NOTCH1 p.S2467* 2C.2 , RB1 p.E31Vfs*17 2C.2 , TP53 p.R248Q 2C.2 | 2C.2 |  | NL |  | NL | 2C.2 |
| SP113926 | Kidney | OTHER | PTEN . 2C.1 | 2C.1 |  | NL |  | NL | 2C.1 |
| SP114016 | Cervix | OTHER | PIK3CA p.E545K 2C.1 | 2C.1 | PIK3CA loss 2C.1 | 2C.1 |  | NL | 2C.1 |
| SP114020 | Cervix | OTHER | RAD50 p.H158Y 2C.1 | 2C.1 |  | NL |  | NL | 2C.1 |
| SP114032 | Cervix | OTHER | FGFR2 p.V680I 2C.1 , KRAS p.G12V 2C.1 | 2C.1 |  | NL |  | NL | 2C.1 |
| SP1144 | Bladder | OTHER | RB1 p.Q850* 2C.2 , TP53 p.R213Q 2C.2 | 2C.2 | AKT2 amp 2C.2 , CCNE1 amp 2C.2 | 2C.2 |  | NL | 2C.2 |
| SP115162 | Head/Neck | OTHER | FGFR3 p.S249C 2C.1 | 2C.1 |  | NL |  | NL | 2C.1 |
| SP115498 | Liver | OTHER | TP53 p.G266R 2C.2 | 2C.2 |  | NL |  | NL | 2C.2 |
| SP115501 | Liver | OTHER | NRAS p.Q61K 2C.1 | 2C.1 |  | NL |  | NL | 2C.1 |
| SP115830 | Liver | OTHER |  | NL |  | NL |  | NL | NL |
| SP116604 | Lymphoid | OTHER | ARID1A p.Y215* 2C.2 | 2C.2 |  | NL |  | NL | 2C.2 |
| SP116606 | Lymphoid | OTHER | SETD2 p.A2339V 2D , SPOP p.M117I 3 | 2D |  | NL |  | NL | 2D |
| SP116608 | Lymphoid | OTHER | MYD88 p.S219C 2C.2 | 2C.2 |  | NL |  | NL | 2C.2 |
| SP116610 | Lymphoid | OTHER | CREBBP p.Y1125* 2C.2 , TP53 p.Y236S 2C.2 | 2C.2 |  | NL |  | NL | 2C.2 |
| SP116612 | Lymphoid | OTHER | ATM p.Y2019F 2C.1 , FGFR2 p.G647E 2C.1 , MYD88 p.L273P 2C.2 | 2C.1 |  | NL |  | NL | 2C.1 |
| SP116614 | Lymphoid | OTHER | CREBBP p.W592* 2C.2 , EZH2 p.Y641N 2C.2 | 2C.2 |  | NL |  | NL | 2C.2 |
| SP116616 | Lymphoid | OTHER | CREBBP p.Q1330* 2C.2 , EZH2 p.Y641F 2C.2 , MYD88 p.S219C 2C.2 | 2C.2 |  | NL |  | NL | 2C.2 |
| SP116618 | Lymphoid | OTHER | CREBBP p.L1464* 2C.2 | 2C.2 |  | NL |  | NL | 2C.2 |
| SP116620 | Lymphoid | OTHER | CREBBP p.N199Rfs*10 2C.2 , EZH2 p.Y641N 2C.2 | 2C.2 |  | NL |  | NL | 2C.2 |
| SP116622 | Lymphoid | OTHER |  | NL |  | NL |  | NL | NL |
| SP116624 | Lymphoid | OTHER | HNF1A p.R229Q 2C.2 | 2C.2 |  | NL |  | NL | 2C.2 |
| SP116627 | Lymphoid | OTHER |  | NL |  | NL |  | NL | NL |
| SP116630 | Lymphoid | OTHER | TP53 p.L257P 2C.2 | 2C.2 |  | NL |  | NL | 2C.2 |
| SP116635 | Lymphoid | OTHER | JAK1 p.K696I 2D | 2D |  | NL |  | NL | 2D |
| SP116638 | Lymphoid | OTHER | CREBBP p.R1446C 2C.2 | 2C.2 |  | NL |  | NL | 2C.2 |
| SP116642 | Lymphoid | OTHER | CREBBP p.Q790* 2C.2 | 2C.2 |  | NL |  | NL | 2C.2 |
| SP116645 | Lymphoid | OTHER | CREBBP p.Y1125Ifs*5 2C.2 , KIT p.P838H 2C.1 , TP53 p.D281N 2C.2 | 2C.1 |  | NL |  | NL | 2C.1 |
| SP116648 | Lymphoid | OTHER | ATRX p.K805E 2C.2 , ATRX p.Y2163H 2C.2 , FANCD2 p.L139F 2C.1 | 2C.1 | AR amp 2C.2 | 2C.2 |  | NL | 2C.1 |
| SP116649 | Lymphoid | OTHER | CREBBP p.Q286* 2C.2 | 2C.2 |  | NL |  | NL | 2C.2 |
| SP116654 | Lymphoid | OTHER | ARID1A p.V63Wfs*38 2C.2 | 2C.2 |  | NL |  | NL | 2C.2 |
| SP116657 | Lymphoid | OTHER | MYD88 p.L273P 2C.2 | 2C.2 |  | NL |  | NL | 2C.2 |
| SP116659 | Lymphoid | OTHER | EZH2 p.Y641N 2C.2 , MYC p.A59T 2C.2 | 2C.2 |  | NL |  | NL | 2C.2 |
| SP116663 | Lymphoid | OTHER |  | NL |  | NL |  | NL | NL |
| SP116665 | Lymphoid | OTHER |  | NL |  | NL |  | NL | NL |
| SP116668 | Lymphoid | OTHER | MAP2K1 p.K57T 2C.1 , NRAS p.Q61H 2C.1 , SMAD4 p.L389Ffs*4 2C.2 | 2C.1 |  | NL |  | NL | 2C.1 |
| SP116670 | Lymphoid | OTHER |  | NL |  | NL |  | NL | NL |
| SP116672 | Lymphoid | OTHER |  | NL |  | NL |  | NL | NL |
| SP116674 | Lymphoid | OTHER | TP53 p.R273C 2C.2 | 2C.2 | CDK6 loss 2C.1 | 2C.1 |  | NL | 2C.1 |
| SP116676 | Lymphoid | OTHER | ARID1A p.Q1364* 2C.2 , XPO1 p.E571K 2C.2 | 2C.2 |  | NL |  | NL | 2C.2 |
| SP116679 | Lymphoid | OTHER | EZH2 p.Y641C 2C.2 | 2C.2 |  | NL |  | NL | 2C.2 |
| SP116683 | Lymphoid | OTHER | CREBBP p.R1446C 2C.2 | 2C.2 |  | NL |  | NL | 2C.2 |
| SP116686 | Lymphoid | OTHER | SETD2 p.K1906T 2D , STAT3 p.E594K 2C.2 | 2C.2 |  | NL |  | NL | 2C.2 |
| SP116688 | Lymphoid | OTHER | ARID1A p.Y1226* 2C.2 , MYC p.T73N 2C.2 | 2C.2 |  | NL |  | NL | 2C.2 |
| SP116690 | Lymphoid | OTHER | PALB2 p.F23L 2C.1 , TP53 p.R249S 2C.2 | 2C.1 |  | NL |  | NL | 2C.1 |
| SP116694 | Lymphoid | OTHER | CREBBP p.I1649Hfs*11 2C.2 , EZH2 p.Y641N 2C.2 , RB1 p.F721Vfs*30 2C.2 | 2C.2 |  | NL |  | NL | 2C.2 |
| SP116697 | Lymphoid | OTHER | SMARCA4 p.R704W 2C.2 | 2C.2 |  | NL | NOTCH1 fusion 3 | 3 | 2C.2 |
| SP116701 | Lymphoid | OTHER |  | NL |  | NL |  | NL | NL |
| SP116703 | Lymphoid | OTHER | EZH2 p.Y641H 2C.2 | 2C.2 |  | NL |  | NL | 2C.2 |
| SP116706 | Lymphoid | OTHER | STAT3 p.S614R 2C.2 | 2C.2 |  | NL |  | NL | 2C.2 |
| SP116709 | Lymphoid | OTHER | CREBBP p.Q598* 2C.2 , STAT3 p.G618R 2C.2 | 2C.2 |  | NL |  | NL | 2C.2 |
| SP116712 | Lymphoid | OTHER | MSH6 p.A1055V 1B , MYC p.A59V 2C.2 , NRAS p.G12D 2C.1 | 1B |  | NL |  | NL | 1B |
| SP116715 | Lymphoid | OTHER | PTCH1 p.A392Gfs*47 2C.1 | 2C.1 |  | NL |  | NL | 2C.1 |
| SP116718 | Lymphoid | OTHER |  | NL |  | NL |  | NL | NL |
| SP116720 | Lymphoid | OTHER | CREBBP p.P1488L 2C.2 | 2C.2 |  | NL |  | NL | 2C.2 |
| SP116723 | Lymphoid | OTHER |  | NL |  | NL |  | NL | NL |
| SP116725 | Lymphoid | OTHER | ARID1A p.Q335* 2C.2 , PTEN p.I101T 2C.1 , TP53 p.S241P 2C.2 | 2C.1 |  | NL |  | NL | 2C.1 |
| SP116726 | Lymphoid | OTHER | MYD88 p.L273P 2C.2 | 2C.2 |  | NL |  | NL | 2C.2 |
| SP1174 | Bladder | OTHER | NFE2L2 p.D29Y 3 | 3 |  | NL |  | NL | 3 |
| SP11808 | Breast | BREAST | ATM p.R2993* 2C.1 | 2C.1 | ERBB2 amp 1A.1 , ERBB2 amp 1A.1 | 1A.1 | ERBB2 fusion 2C.1 | 2C.1 | 1A.1 |
| SP11878 | Breast | BREAST |  | NL | MYC amp 2C.2 | 2C.2 |  | NL | 2C.2 |
| SP11948 | Breast | BREAST | NBN p.S213L 2C.1 , PIK3CA p.H1047Y 1A.1 , TP53 p.R273H 2C.2 , TP53 p.D208G 2C.2 | 1A.1 | AKT3 loss 2C.2 , CCND2 amp 2C.2 | 2C.2 |  | NL | 1A.1 |
| SP119755 | Colon/Rectum | COLON/RECTUM | FBXW7 p.R224* 2C.2 , FBXW7 p.R465C 2C.2 | 2C.2 |  | NL |  | NL | 2C.2 |
| SP12049 | Breast | BREAST | ALK p.E1340V 2C.1 , TP53 p.C176Y 2C.2 | 2C.1 | CCND2 amp 2C.2 | 2C.2 |  | NL | 2C.1 |
| SP120767 | Thyroid | OTHER | BRAF p.V600E 1A.1 | 1A.1 |  | NL |  | NL | 1A.1 |
| SP121761 | Bone/SoftTissue | OTHER |  | NL | CDK4 amp 2C.1 , MDM2 amp 2C.2 , MDM2 amp 2C.2 , MDM2 amp 2C.2 , MDM2 amp 2C.2 , NTRK1 loss 2C.1 , NTRK1 loss 2C.1 | 2C.1 |  | NL | 2C.1 |
| SP121763 | Bone/SoftTissue | OTHER |  | NL | MDM2 amp 2C.2 , TERT loss 3 | 2C.2 |  | NL | 2C.2 |
| SP121774 | Bone/SoftTissue | OTHER |  | NL | CDK4 amp 2C.1 , MDM2 amp 2C.2 | 2C.1 |  | NL | 2C.1 |
| SP121781 | Bone/SoftTissue | OTHER | ATRX p.V277Gfs*5 2C.2 | 2C.2 | NTRK1 loss 2C.1 , NTRK1 loss 2C.1 , NTRK1 loss 2C.1 , PPARG loss 3 , TERT loss 3 | 2C.1 |  | NL | 2C.1 |
| SP121783 | Bone/SoftTissue | OTHER |  | NL | CDK4 amp 2C.1 , MDM2 amp 2C.2 , MDM2 amp 2C.2 , MDM2 amp 2C.2 , MDM2 amp 2C.2 , TERT loss 3 | 2C.1 | TERT fusion 2C.2 | 2C.2 | 2C.1 |
| SP121790 | Bone/SoftTissue | OTHER |  | NL | CCNE1 amp 2C.2 , CDK2 amp 2D , CDK4 amp 2C.1 , FGFR4 loss 2C.1 , MDM2 amp 2C.2 , MDM2 amp 2C.2 , MDM2 amp 2C.2 , PDGFRB loss 2C.1 | 2C.1 |  | NL | 2C.1 |
| SP121808 | Bone/SoftTissue | OTHER |  | NL | CCND2 amp 2C.2 , CDK2 amp 2D , CDK2 amp 2D , CDK2 amp 2D , CDK4 amp 2C.1 , MDM2 amp 2C.2 , MDM2 amp 2C.2 , MDM2 amp 2C.2 | 2C.1 |  | NL | 2C.1 |
| SP121811 | Bone/SoftTissue | OTHER |  | NL | CDK4 amp 2C.1 , MDM2 amp 2C.2 , MDM2 amp 2C.2 , MDM2 amp 2C.2 | 2C.1 |  | NL | 2C.1 |
| SP121816 | Bone/SoftTissue | OTHER |  | NL | CDK4 amp 2C.1 , MDM2 amp 2C.2 , MDM2 amp 2C.2 , MDM4 loss 2D | 2C.1 | TERT fusion 2C.2 | 2C.2 | 2C.1 |
| SP121824 | Bone/SoftTissue | OTHER |  | NL | AXL amp 2C.2 , CDK4 amp 2C.1 , CDK4 amp 2C.1 , MDM2 amp 2C.2 , MDM2 amp 2C.2 , MDM2 amp 2C.2 , MDM2 amp 2C.2 | 2C.1 | AXL fusion 2C.2 | 2C.2 | 2C.1 |
| SP121828 | Bone/SoftTissue | OTHER |  | NL | CDK4 amp 2C.1 , FGFR1 amp 2C.1 , FGFR1 amp 2C.1 , MDM2 amp 2C.2 , MDM2 amp 2C.2 , MDM2 amp 2C.2 , MDM2 amp 2C.2 , MDM2 amp 2C.2 , MDM2 amp 2C.2 , MDM2 amp 2C.2 , TERT loss 3 , TERT loss 3 | 2C.1 | JAK2 fusion 2C.1 | 2C.1 | 2C.1 |
| SP121831 | Bone/SoftTissue | OTHER |  | NL | CCNE1 amp 2C.2 , CDK4 amp 2C.1 , MDM2 amp 2C.2 | 2C.1 |  | NL | 2C.1 |
| SP121837 | Bone/SoftTissue | OTHER | SF3B1 p.F746V 3 | 3 | CCND3 loss 2C.2 , CCND3 loss 2C.2 , CCND3 loss 2C.2 , CDK4 amp 2C.1 , MDM2 amp 2C.2 , MDM2 amp 2C.2 | 2C.1 |  | NL | 2C.1 |
| SP121841 | Bone/SoftTissue | OTHER |  | NL | CDK4 amp 2C.1 , MDM2 amp 2C.2 | 2C.1 |  | NL | 2C.1 |
| SP121847 | Bone/SoftTissue | OTHER |  | NL | CDK2 amp 2D , CDK2 amp 2D , CDK4 amp 2C.1 , FGFR4 loss 2C.1 , MDM2 amp 2C.2 , MDM2 amp 2C.2 , PIK3CB loss 2C.1 | 2C.1 |  | NL | 2C.1 |
| SP121852 | Bone/SoftTissue | OTHER |  | NL | AR amp 2C.2 , CDK4 amp 2C.1 , MDM2 amp 2C.2 , MDM2 amp 2C.2 , MDM2 amp 2C.2 , MDM2 amp 2C.2 , MDM2 amp 2C.2 | 2C.1 |  | NL | 2C.1 |
| SP121859 | Bone/SoftTissue | OTHER |  | NL | CDK4 amp 2C.1 , MDM2 amp 2C.2 , MDM2 amp 2C.2 , MDM2 amp 2C.2 | 2C.1 |  | NL | 2C.1 |
| SP12186 | Breast | BREAST | NOTCH2 p.S965C 2C.2 , PIK3CA p.E542K 1A.1 , TP53 p.A161T 2C.2 | 1A.1 |  | NL |  | NL | 1A.1 |
| SP121861 | Bone/SoftTissue | OTHER |  | NL | CDK4 amp 2C.1 , MDM2 amp 2C.2 , MDM2 amp 2C.2 | 2C.1 |  | NL | 2C.1 |
| SP121865 | Bone/SoftTissue | OTHER | RB1 p.R46Kfs*3 2C.2 , RET p.R886Q 2C.1 | 2C.1 | IGF1R amp 2D | 2D |  | NL | 2C.1 |
| SP121870 | Bone/SoftTissue | OTHER | PIK3CA p.C420R 2C.1 | 2C.1 |  | NL |  | NL | 2C.1 |
| SP122361 | Bone/SoftTissue | OTHER |  | NL | CCND3 loss 2C.2 , MDM2 amp 2C.2 , MDM2 amp 2C.2 , MDM2 amp 2C.2 , NTRK2 amp 2C.1 | 2C.1 | ETS1 fusion 2D , PIK3CA fusion 2C.1 | 2C.1 | 2C.1 |
| SP122372 | Bone/SoftTissue | OTHER |  | NL |  | NL |  | NL | NL |
| SP122392 | Bone/SoftTissue | OTHER | TSC1 p.R892Kfs*11 2C.1 | 2C.1 |  | NL |  | NL | 2C.1 |
| SP122412 | Bone/SoftTissue | OTHER |  | NL |  | NL |  | NL | NL |
| SP122476 | Bone/SoftTissue | OTHER | TP53 p.G262Vfs*83 2C.2 | 2C.2 |  | NL |  | NL | 2C.2 |
| SP122489 | Bone/SoftTissue | OTHER | TP53 p.W91* 2C.2 | 2C.2 |  | NL |  | NL | 2C.2 |
| SP122560 | Bone/SoftTissue | OTHER | GATA2 p.Q328K 2C.2 , RB1 . 2C.2 , TP53 p.E336Afs*10 2C.2 | 2C.2 |  | NL |  | NL | 2C.2 |
| SP122590 | Bone/SoftTissue | OTHER | PTEN p.G127R 2C.1 | 2C.1 |  | NL |  | NL | 2C.1 |
| SP122634 | Bone/SoftTissue | OTHER | RB1 p.R445* 2C.2 , TP53 p.K132R 2C.2 | 2C.2 |  | NL |  | NL | 2C.2 |
| SP122676 | Bone/SoftTissue | OTHER | TP53 p.Q317* 2C.2 | 2C.2 |  | NL |  | NL | 2C.2 |
| SP122702 | Bone/SoftTissue | OTHER | RB1 p.I441Lfs*16 2C.2 , TP53 p.R337L 2C.2 | 2C.2 | CCND3 loss 2C.2 | 2C.2 |  | NL | 2C.2 |
| SP122714 | Bone/SoftTissue | OTHER |  | NL |  | NL |  | NL | NL |
| SP122725 | Bone/SoftTissue | OTHER | KRAS p.Q61H 2C.1 , PIK3CA p.H1047R 2C.1 , PPP2R1A p.P179R 2C.1 , TP53 p.Y205C 2C.2 | 2C.1 | KRAS amp 2C.1 , PIK3CA loss 2C.1 | 2C.1 |  | NL | 2C.1 |
| SP123010 | Bone/SoftTissue | OTHER | BRCA2 p.T219Lfs*11 2C.1 , CCND1 p.V290M 2C.2 , KIT p.W557_E561delWKVVE 1A.1 | 1A.1 |  | NL |  | NL | 1A.1 |
| SP123836 | Kidney | OTHER |  | NL |  | NL |  | NL | NL |
| SP123840 | Kidney | OTHER |  | NL |  | NL |  | NL | NL |
| SP123842 | Kidney | OTHER | TSC1 p.E174Sfs*32 2C.1 | 2C.1 |  | NL |  | NL | 2C.1 |
| SP123844 | Kidney | OTHER |  | NL |  | NL |  | NL | NL |
| SP123846 | Kidney | OTHER |  | NL |  | NL |  | NL | NL |
| SP123850 | Kidney | OTHER |  | NL |  | NL |  | NL | NL |
| SP123852 | Kidney | OTHER |  | NL |  | NL |  | NL | NL |
| SP123854 | Kidney | OTHER |  | NL |  | NL |  | NL | NL |
| SP123856 | Kidney | OTHER |  | NL |  | NL |  | NL | NL |
| SP123858 | Kidney | OTHER |  | NL |  | NL |  | NL | NL |
| SP123870 | Kidney | OTHER |  | NL |  | NL |  | NL | NL |
| SP123872 | Kidney | OTHER | TP53 p.K292* 2C.2 | 2C.2 |  | NL |  | NL | 2C.2 |
| SP123874 | Kidney | OTHER | TSC2 p.E1049K 2C.1 | 2C.1 |  | NL |  | NL | 2C.1 |
| SP123876 | Kidney | OTHER |  | NL |  | NL |  | NL | NL |
| SP123878 | Kidney | OTHER |  | NL |  | NL |  | NL | NL |
| SP123882 | Kidney | OTHER |  | NL |  | NL |  | NL | NL |
| SP123884 | Kidney | OTHER | SETD2 p.R2399* 2D | 2D |  | NL |  | NL | 2D |
| SP123886 | Kidney | OTHER |  | NL |  | NL |  | NL | NL |
| SP123888 | Kidney | OTHER |  | NL |  | NL |  | NL | NL |
| SP123890 | Kidney | OTHER | MTOR p.I2017T 2C.1 | 2C.1 |  | NL |  | NL | 2C.1 |
| SP123892 | Kidney | OTHER |  | NL |  | NL |  | NL | NL |
| SP123894 | Kidney | OTHER |  | NL |  | NL |  | NL | NL |
| SP123897 | Kidney | OTHER |  | NL |  | NL |  | NL | NL |
| SP123900 | Kidney | OTHER |  | NL |  | NL |  | NL | NL |
| SP123902 | Kidney | OTHER | TSC1 p.R718Pfs*3 2C.1 | 2C.1 |  | NL |  | NL | 2C.1 |
| SP123950 | Kidney | OTHER | TP53 p.H178Q 2C.2 | 2C.2 |  | NL |  | NL | 2C.2 |
| SP123953 | Kidney | OTHER |  | NL |  | NL |  | NL | NL |
| SP123955 | Kidney | OTHER |  | NL |  | NL |  | NL | NL |
| SP123958 | Kidney | OTHER |  | NL |  | NL |  | NL | NL |
| SP123964 | Kidney | OTHER | TP53 p.R337H 2C.2 , TP53 p.C176F 2C.2 | 2C.2 |  | NL |  | NL | 2C.2 |
| SP123967 | Kidney | OTHER |  | NL |  | NL |  | NL | NL |
| SP123969 | Kidney | OTHER |  | NL | MYC amp 2C.2 | 2C.2 |  | NL | 2C.2 |
| SP123972 | Kidney | OTHER | NRAS p.Q61K 2C.1 | 2C.1 |  | NL |  | NL | 2C.1 |
| SP123978 | Kidney | OTHER |  | NL |  | NL |  | NL | NL |
| SP123984 | Kidney | OTHER |  | NL |  | NL |  | NL | NL |
| SP123988 | Kidney | OTHER |  | NL |  | NL |  | NL | NL |
| SP123995 | Kidney | OTHER | TP53 p.C141W 2C.2 | 2C.2 |  | NL |  | NL | 2C.2 |
| SP123998 | Kidney | OTHER | ATM p.G724V 2C.1 | 2C.1 |  | NL |  | NL | 2C.1 |
| SP124003 | Kidney | OTHER | MTOR p.L2427R 2C.1 | 2C.1 |  | NL |  | NL | 2C.1 |
| SP124013 | Kidney | OTHER |  | NL |  | NL |  | NL | NL |
| SP124017 | Kidney | OTHER |  | NL |  | NL |  | NL | NL |
| SP124021 | Kidney | OTHER |  | NL |  | NL |  | NL | NL |
| SP124033 | Kidney | OTHER |  | NL |  | NL |  | NL | NL |
| SP124969 | Lymphoid | OTHER | BRAF p.V600E 2C.1 , MTOR p.V2006F 2C.1 | 2C.1 |  | NL |  | NL | 2C.1 |
| SP124971 | Lymphoid | OTHER | TP53 p.Y236N 2C.2 | 2C.2 |  | NL |  | NL | 2C.2 |
| SP124977 | Lymphoid | OTHER | SPOP p.M117R 3 | 3 |  | NL |  | NL | 3 |
| SP124981 | Lymphoid | OTHER | ATM p.Y1470C 2C.1 , MYD88 p.L273P 2C.2 | 2C.1 |  | NL |  | NL | 2C.1 |
| SP127628 | Lymphoid | OTHER | MYC p.Q321H 2C.2 | 2C.2 |  | NL |  | NL | 2C.2 |
| SP127630 | Lymphoid | OTHER |  | NL |  | NL |  | NL | NL |
| SP127632 | Lymphoid | OTHER |  | NL |  | NL |  | NL | NL |
| SP127634 | Lymphoid | OTHER |  | NL |  | NL |  | NL | NL |
| SP127636 | Lymphoid | OTHER |  | NL |  | NL |  | NL | NL |
| SP127638 | Lymphoid | OTHER | TP53 p.E171* 2C.2 | 2C.2 |  | NL |  | NL | 2C.2 |
| SP127640 | Lymphoid | OTHER | MAP2K1 p.F53L 2C.1 | 2C.1 |  | NL |  | NL | 2C.1 |
| SP12856 | Breast | BREAST | PIK3CA p.E545K 1A.1 , PIK3R1 p.E515K 2C.2 , SETD2 p.R1509T 2D , STK11 p.E33K 2C.1 | 1A.1 |  | NL |  | NL | 1A.1 |
| SP13036 | Breast | BREAST | PIK3CA p.H1047R 1A.1 , TP53 p.R273C 2C.2 | 1A.1 | CCNE1 amp 2C.2 , CCNE1 amp 2C.2 , KRAS amp 2C.1 | 2C.1 |  | NL | 1A.1 |
| SP1305 | Bladder | OTHER | ERCC2 p.N238S 2C.2 , NOTCH2 p.G292R 2C.2 , TP53 p.H214R 2C.2 | 2C.2 | EGFR loss 2C.1 | 2C.1 |  | NL | 2C.1 |
| SP13072 | Cervix | OTHER | ARID1A p.Q1363* 2C.2 , ERBB3 p.V104M 2C.2 , SMARCA4 p.R973W 2C.2 | 2C.2 |  | NL |  | NL | 2C.2 |
| SP13078 | Cervix | OTHER |  | NL |  | NL |  | NL | NL |
| SP13084 | Cervix | OTHER |  | NL |  | NL |  | NL | NL |
| SP13206 | Cervix | OTHER |  | NL |  | NL |  | NL | NL |
| SP13242 | Cervix | OTHER |  | NL |  | NL |  | NL | NL |
| SP1365 | Bladder | OTHER | TP53 p.C238F 2C.2 | 2C.2 | FGFR3 loss 2C.1 , MDM4 loss 2D , TERT loss 3 | 2C.1 |  | NL | 2C.1 |
| SP1377 | Bladder | OTHER | NF1 p.E1889K 2C.1 , TSC1 p.L536* 2C.1 | 2C.1 | FGFR3 loss 2C.1 | 2C.1 |  | NL | 2C.1 |
| SP1419 | Bladder | OTHER | NOTCH1 p.D260Y 2C.2 | 2C.2 | CCND2 amp 2C.2 , FGFR1 amp 2C.1 , NTRK3 amp 2C.1 , RICTOR loss 2C.2 , TERT loss 3 | 2C.1 |  | NL | 2C.1 |
| SP1431 | Bladder | OTHER | ARID1A p.S1171Rfs*8 2C.2 | 2C.2 |  | NL |  | NL | 2C.2 |
| SP1491 | Bladder | OTHER | BRCA2 p.M192T 2C.1 , ERCC2 p.S44L 2C.2 , HRAS p.Q61K 2C.1 , MED12 p.G1218R 3 | 2C.1 |  | NL |  | NL | 2C.1 |
| SP1677 | Bladder | OTHER | CREBBP p.Q887* 2C.2 , FGFR3 p.G380R 1A.1 , SMARCA4 p.T786N 2C.2 | 1A.1 |  | NL |  | NL | 1A.1 |
| SP16886 | Colon/Rectum | COLON/RECTUM | ATM p.R337C 2C.1 , ATR p.E706* 2C.2 , ATRX p.A2137T 2C.2 , BRCA2 p.E1493* 2C.1 , CREBBP p.A1093T 2D , EZH2 p.R679H 2C.2 , FBXW7 p.D279Y 2C.2 , KRAS p.Q22K 1A.1 , KRAS p.K117N 1A.1 , MAP2K4 p.E203* 2C.1 , MAP2K4 p.E141* 2C.1 , MET p.R1166Q 2C.1 , MET p.R1188* 2C.1 , NF1 p.E1074G 2C.1 , PIK3CA p.R88Q 2C.1 , PIK3CA p.H1047Q 2C.1 , POLE p.P286R 2C.1 , PTEN p.E114* 2C.1 , PTEN p.W111* 2C.1 , RAD50 p.E1033* 2C.1 , RAD51C p.V156A 2C.1 , RET p.R959W 2C.1 , RNF43 p.D140N 2C.2 , SMAD4 p.Q534P 2C.2 , TP53 p.R213* 2C.2 , TP53 p.S94* 2C.2 | 1A.1 |  | NL |  | NL | 1A.1 |
| SP16934 | Colon/Rectum | COLON/RECTUM | NRAS p.G12C 1.A.1 , PIK3CA p.T1052K 2C.1 | 1.A.1 |  | NL |  | NL | 1.A.1 |
| SP16958 | Colon/Rectum | COLON/RECTUM | ATM p.W2769* 2C.1 , BRAF p.V600E 1B , PIK3R1 p.P92Hfs*23 2C.2 , RAD51C p.R312W 2C.1 | 1B |  | NL | RSPO3 fusion 3 | 3 | 1B |
| SP17016 | Colon/Rectum | COLON/RECTUM | ATM p.D2721N 2C.1 , KRAS p.G12V 1A.1 , PIK3CA p.E545K 2C.1 | 1A.1 |  | NL |  | NL | 1A.1 |
| SP1712 | Bladder | OTHER | ATM p.R337H 2C.1 , FGFR3 p.S249C 1A.1 , PIK3CA p.E545K 2C.1 | 1A.1 |  | NL |  | NL | 1A.1 |
| SP1724 | Bladder | OTHER | FGFR3 p.S249C 1A.1 | 1A.1 |  | NL |  | NL | 1A.1 |
| SP17294 | Colon/Rectum | COLON/RECTUM | KRAS p.G12V 1A.1 , PIK3CA p.D350N 2C.1 , TP53 p.R175H 2C.2 | 1A.1 |  | NL |  | NL | 1A.1 |
| SP17329 | Colon/Rectum | COLON/RECTUM | KRAS p.G12V 1A.1 | 1A.1 |  | NL |  | NL | 1A.1 |
| SP17430 | Colon/Rectum | COLON/RECTUM | TP53 p.Y205C 2C.2 | 2C.2 |  | NL |  | NL | 2C.2 |
| SP1781 | Bladder | OTHER | ARID1A p.E2035* 2C.2 , ATM p.E158Q 2C.1 , CDKN1B p.S7C 2D , NF1 p.Q756* 2C.1 , TP53 p.E271K 2C.2 , TSC2 p.R367W 2C.1 | 2C.1 | CCND1 amp 2C.2 , FGF19 amp 2D , FGF3 amp 2D | 2C.2 |  | NL | 2C.1 |
| SP17905 | Colon/Rectum | COLON/RECTUM | AR p.E32K 2C.2 , ATM p.R1618* 2C.1 , ATM p.W1058R 2C.1 , ATM p.L612I 2C.1 , ATM p.R250* 2C.1 , ATR p.E306* 2C.2 , ATR p.R1015Q 2C.2 , ATR p.E1078* 2C.2 , ATR p.L1268F 2C.2 , ATRX p.D898Y 2C.2 , ATRX p.K2072T 2C.2 , BAP1 p.D74Y 2C.1 , BRAF p.S637P 2C.1 , BRCA1 p.E203* 2C.1 , BRCA2 p.E97* 2C.1 , BRCA2 p.E2635G 2C.1 , BRCA2 p.S445Y 2C.1 , CDK6 p.S204I 2C.1 , CREBBP p.Y1457* 2D , CTNNB1 p.E334A 2C.2 , ERCC2 p.R88Q 2C.2 , FBXW7 p.R393Q 2C.2 , FBXW7 p.R658* 2C.2 , KIT p.A784T 1A.1 , KIT p.Y568S 1A.1 , MAX p.L46S 3 , MRE11A p.D394Y 2C.1 , MRE11A p.E460* 2C.1 , MSH6 p.R732Q 1B , NBN p.R43Q 2C.1 , NBN p.Y322C 2C.1 , NF1 p.I2684L 2C.1 , NF1 p.T1853A 2C.1 , NF1 p.R1434I 2C.1 , NF2 p.S506Y 2C.1 , NF2 p.K123N 2C.1 , NOTCH2 p.R1931C 2C.2 , PDGFRA p.K939N 2C.1 , PIK3CA p.R88Q 2C.1 , PIK3CB p.R149Q 2C.1 , PIK3CB p.S1070Y 2C.1 , PIK3R1 p.E683K 2C.2 , PIK3R1 p.E52* 2C.2 , POLE p.P286S 2C.1 , POLE p.P286R 2C.1 , RAD50 p.K6N 2C.1 , SMAD4 p.R361H 2C.2 , SMARCA4 p.L1383P 2C.2 , TSC1 p.E981* 2C.1 | 1A.1 |  | NL |  | NL | 1A.1 |
| SP18121 | Colon/Rectum | COLON/RECTUM | BRCA2 p.T3033Lfs*29 2C.1 , FANCD2 p.K195E 2C.1 , KRAS p.G13D 1A.1 , KRAS p.A146T 1A.1 , NF2 p.R262* 2C.1 , NOTCH1 p.R1854H 2C.2 , PIK3CA p.E726K 2C.1 , POLE p.Y473C 2C.1 , PTCH1 p.T807A 2C.1 , RNF43 p.G659Vfs*41 2C.2 , SMARCA4 p.R1369S 2C.2 , SMARCA4 p.A1218V 2C.2 , SMARCA4 p.G1136Afs*4 2C.2 | 1A.1 |  | NL |  | NL | 1A.1 |
| SP18787 | Colon/Rectum | COLON/RECTUM | ERBB2 p.V842I 2C.1 , KRAS p.G12D 1A.1 | 1A.1 |  | NL |  | NL | 1A.1 |
| SP18946 | Colon/Rectum | COLON/RECTUM | ATM p.E2676* 2C.1 , ATR p.R1015Q 2C.2 , ATR p.R1647H 2C.2 , ATRX p.D514Y 2C.2 , ATRX p.K1583N 2C.2 , CDK4 p.R139Q 2C.1 , DDR2 p.P541S 2C.2 , FANCI p.K869N 2C.1 , KRAS p.K117N 1A.1 , MED12 p.G44C 3 , MSH6 p.R922Q 1B , MSH6 p.A175T 1B , NBN p.E62* 2C.1 , NOTCH2 p.L1740I 2C.2 , NOTCH2 p.D1975G 2C.2 , PIK3CA p.M1043I 2C.1 , PIK3CA p.R88Q 2C.1 , POLE p.F367S 2C.1 , PTEN p.E299* 2C.1 , PTPN11 p.E69D 2C.2 , RAD50 p.A182T 2C.1 , SMARCA4 p.W875R 2C.2 , SMARCA4 p.R381Q 2C.2 , TP53 p.R213* 2C.2 , TP53 p.K132T 2C.2 | 1A.1 |  | NL |  | NL | 1A.1 |
| SP19215 | Colon/Rectum | COLON/RECTUM | CDK12 p.F802V 2C.1 , ERBB3 p.V104M 2C.2 , RNF43 p.R529Q 2C.2 | 2C.1 |  | NL |  | NL | 2C.1 |
| SP19295 | Colon/Rectum | COLON/RECTUM | ARID1A p.R1989* 2C.2 , ATM p.F2839L 2C.1 , ATRX p.L1612I 2C.2 , BRCA2 p.E2129* 2C.1 , IDH1 p.R132C 2C.1 , KDR p.R1032Q 2D , KRAS p.A146T 1A.1 , MSH6 p.R1242H 1B , MTOR p.E1799K 2C.1 , NF1 p.R2450* 2C.1 , NF1 p.E524* 2C.1 , PIK3CA p.T1025A 2C.1 , PIK3CA p.E542A 2C.1 , POLE p.P286H 2C.1 , SMAD4 p.E520* 2C.2 | 1A.1 |  | NL |  | NL | 1A.1 |
| SP19582 | Colon/Rectum | COLON/RECTUM | FBXW7 p.R505C 2C.2 , RAD50 p.L1211F 2C.1 , TP53 p.Y236* 2C.2 | 2C.1 | CCND2 amp 2C.2 | 2C.2 |  | NL | 2C.1 |
| SP19606 | Colon/Rectum | COLON/RECTUM | GNAQ p.R181T 2C.2 , NRAS p.Q61K 1.A.1 , TP53 p.F109C 2C.2 | 1.A.1 |  | NL |  | NL | 1.A.1 |
| SP19670 | Colon/Rectum | COLON/RECTUM | ARID1A p.Q944* 2C.2 , KRAS p.K117N 1A.1 , TP53 p.R196* 2C.2 | 1A.1 |  | NL |  | NL | 1A.1 |
| SP19750 | Colon/Rectum | COLON/RECTUM | TP53 p.P278R 2C.2 | 2C.2 |  | NL |  | NL | 2C.2 |
| SP19983 | Colon/Rectum | COLON/RECTUM | KRAS p.G12D 1A.1 , PIK3CA p.E545K 2C.1 | 1A.1 | ERBB2 amp 2C.1 | 2C.1 |  | NL | 1A.1 |
| SP20993 | Colon/Rectum | COLON/RECTUM | NF1 p.R1276Q 2C.1 , TP53 p.V173M 2C.2 | 2C.1 |  | NL |  | NL | 2C.1 |
| SP21057 | Colon/Rectum | COLON/RECTUM | TP53 p.P301Qfs*44 2C.2 | 2C.2 |  | NL |  | NL | 2C.2 |
| SP21193 | Colon/Rectum | COLON/RECTUM | KRAS p.G12V 1A.1 , TP53 p.L145R 2C.2 | 1A.1 |  | NL |  | NL | 1A.1 |
| SP21400 | Colon/Rectum | COLON/RECTUM | ATM p.L2077I 2C.1 , ATM p.R1730* 2C.1 , CREBBP p.R1446H 2D , FANCD2 p.R1273* 2C.1 , FGFR1 p.S549L 2C.1 , KRAS p.G13D 1A.1 , MET p.F228C 2C.1 , MRE11A p.G180D 2C.1 , MRE11A p.N617H 2C.1 , MSH2 p.E580* 1B , MSH6 p.E946* 1B , PIK3CA p.Y1021C 2C.1 , PIK3CA p.V344A 2C.1 , POLE p.V411L 2C.1 , RET p.P914S 2C.1 , SETD2 p.E138* 2D , SETD2 p.R1523C 2D , SETD2 p.R2122Q 2D , SMARCA4 p.R1623W 2C.2 , TSC1 p.L72R 2C.1 , TSC1 p.R228Q 2C.1 | 1A.1 |  | NL |  | NL | 1A.1 |
| SP21528 | Colon/Rectum | COLON/RECTUM |  | NL |  | NL |  | NL | NL |
| SP22031 | Colon/Rectum | COLON/RECTUM | ATM p.R250* 2C.1 , BRCA2 p.R2494Q 2C.1 , GNAS p.R201H 2C.2 , KRAS p.A146T 1A.1 , MAP2K4 p.D197Y 2C.1 , MRE11A p.K298N 2C.1 , MTOR p.S2215Y 2C.1 , PIK3CA p.M1043I 2C.1 , PIK3CA p.R88Q 2C.1 , PIK3R1 p.R348* 2C.2 , POLE p.S297F 2C.1 , RAD51C p.D254Y 2C.1 , SETD2 p.R529I 2D , TP53 p.P152L 2C.2 | 1A.1 |  | NL |  | NL | 1A.1 |
| SP22750 | Colon/Rectum | COLON/RECTUM | ARID1A p.P1898Hfs*25 2C.2 , ARID1A p.R1722* 2C.2 , PPP2R1A p.R183W 2C.1 | 2C.1 |  | NL |  | NL | 2C.1 |
| SP23078 | Colon/Rectum | COLON/RECTUM | TP53 p.R175H 2C.2 | 2C.2 |  | NL |  | NL | 2C.2 |
| SP23639 | CNS | CNS |  | NL | EGFR loss 2C.1 , EGFR loss 2C.1 | 2C.1 | FGFR3 fusion 2C.1 | 2C.1 | 2C.1 |
| SP23925 | CNS | CNS | ATRX p.K358Tfs*3 2C.2 , IDH1 p.R132H 2C.1 | 2C.1 |  | NL |  | NL | 2C.1 |
| SP24236 | CNS | CNS | EGFR p.G598V 2C.1 , RAD50 p.K545E 2C.1 | 2C.1 | EGFR loss 2C.1 | 2C.1 | EGFR fusion 2C.1 | 2C.1 | 2C.1 |
| SP24565 | CNS | CNS | PIK3CA p.M1043V 2C.1 , PTPN11 p.D61Y 2C.2 | 2C.1 |  | NL |  | NL | 2C.1 |
| SP24815 | CNS | CNS | PIK3CA p.C90G 2C.1 | 2C.1 | CDK4 amp 2C.1 | 2C.1 |  | NL | 2C.1 |
| SP25350 | CNS | CNS | ATM p.N1094Dfs*14 2C.1 | 2C.1 | EGFR loss 2C.1 | 2C.1 |  | NL | 2C.1 |
| SP25494 | CNS | CNS | CREBBP p.D1481N 2D , EGFR p.A289V 2C.1 , FGFR1 p.D585N 2C.1 , FLT3 p.T526M 2C.1 , NOTCH1 p.D1958N 2C.2 , TP53 p.G266E 2C.2 , TSC2 p.D1631N 1A.1 | 1A.1 | EGFR loss 2C.1 , EGFR loss 2C.1 , EGFR loss 2C.1 | 2C.1 |  | NL | 1A.1 |
| SP25518 | CNS | CNS | TP53 p.H179R 2C.2 | 2C.2 | EGFR loss 2C.1 | 2C.1 | EGFR fusion 2C.1 | 2C.1 | 2C.1 |
| SP25833 | CNS | CNS |  | NL | EGFR loss 2C.1 , EGFR loss 2C.1 | 2C.1 |  | NL | 2C.1 |
| SP25905 | CNS | CNS | EGFR p.A289T 2C.1 | 2C.1 | CDK6 loss 2C.1 , EGFR loss 2C.1 | 2C.1 | FGFR3 fusion 2C.1 | 2C.1 | 2C.1 |
| SP26439 | CNS | CNS | ATRX p.W2001Cfs*14 2C.2 , IDH1 p.R132H 2C.1 , TP53 p.R273H 2C.2 | 2C.1 | FGFR2 amp 2C.1 | 2C.1 | FGFR3 fusion 2C.1 | 2C.1 | 2C.1 |
| SP26475 | CNS | CNS |  | NL |  | NL |  | NL | NL |
| SP26499 | CNS | CNS | NF1 p.G1082C 2C.1 , PIK3CB p.E1051K 2C.1 | 2C.1 |  | NL | FGFR3 fusion 2C.1 | 2C.1 | 2C.1 |
| SP26649 | CNS | CNS |  | NL | AKT1 amp 2C.2 , EGFR loss 2C.1 , EGFR loss 2C.1 | 2C.1 |  | NL | 2C.1 |
| SP26709 | CNS | CNS | PTEN p.R130* 2C.1 | 2C.1 | MDM4 loss 2D | 2D | NTRK1 fusion 1A.1 | 1A.1 | 1A.1 |
| SP2714 | Breast | BREAST | CDKN2A p.H83Y 2C.1 , TP53 p.G108Tfs*39 2C.2 , TP53 p.G108Afs*39 2C.2 | 2C.1 |  | NL |  | NL | 2C.1 |
| SP27201 | CNS | CNS | EGFR p.A289D 2C.1 , PIK3CA p.E103G 2C.1 , TP53 p.R248Q 2C.2 , TP53 p.S127Y 2C.2 | 2C.1 | AKT3 loss 2C.2 , CDK4 amp 2C.1 , EGFR loss 2C.1 , KIT loss 2C.1 , KIT loss 2C.1 , PDGFRA loss 2C.1 | 2C.1 |  | NL | 2C.1 |
| SP2731 | Breast | BREAST |  | NL |  | NL |  | NL | NL |
| SP27339 | CNS | CNS | EGFR p.A289V 2C.1 | 2C.1 | CCND2 amp 2C.2 , CDK4 amp 2C.1 , EGFR loss 2C.1 , MDM4 loss 2D | 2C.1 |  | NL | 2C.1 |
| SP27603 | CNS | CNS | EGFR p.G598V 2C.1 , PTPN11 p.Q510H 2C.2 | 2C.1 | EGFR loss 2C.1 , EGFR loss 2C.1 | 2C.1 |  | NL | 2C.1 |
| SP2766 | Breast | BREAST | BAP1 p.Y671Lfs*37 2C.1 , TP53 p.C242Afs*5 2C.2 | 2C.1 |  | NL |  | NL | 2C.1 |
| SP2781 | Breast | BREAST | PIK3CA p.H1047R 1A.1 | 1A.1 | CCND1 amp 2C.2 , FGF19 amp 2D , FGF3 amp 2D | 2C.2 |  | NL | 1A.1 |
| SP2793 | Breast | BREAST | BRCA2 p.C3304S 1A.1 , CDKN2A p.R80Pfs*71 2C.1 | 1A.1 |  | NL |  | NL | 1A.1 |
| SP27957 | CNS | CNS | EGFR p.A289V 2C.1 , RB1 p.L779* 2C.2 , TP53 p.R267P 2C.2 | 2C.1 | EGFR loss 2C.1 , EGFR loss 2C.1 , EGFR loss 2C.1 , EGFR loss 2C.1 | 2C.1 |  | NL | 2C.1 |
| SP2799 | Breast | BREAST | CDK12 p.N939S 2C.1 , PIK3CA p.H1047R 1A.1 | 1A.1 |  | NL |  | NL | 1A.1 |
| SP2801 | Breast | BREAST |  | NL | CCND1 amp 2C.2 , FGF19 amp 2D , FGF3 amp 2D , MDM4 loss 2D | 2C.2 |  | NL | 2C.2 |
| SP2826 | Breast | BREAST | TP53 p.V216M 2C.2 | 2C.2 |  | NL |  | NL | 2C.2 |
| SP28275 | CNS | CNS | CDKN2A p.L78Hfs*41 2C.1 | 2C.1 | CDK6 loss 2C.1 , CDK6 loss 2C.1 , EGFR loss 2C.1 | 2C.1 |  | NL | 2C.1 |
| SP28581 | CNS | CNS | RB1 p.R445* 2C.2 | 2C.2 | CCND2 amp 2C.2 , EGFR loss 2C.1 , EGFR loss 2C.1 , MDM2 amp 2C.2 | 2C.1 |  | NL | 2C.1 |
| SP28791 | CNS | CNS | ATRX p.K1424Gfs*65 2C.2 , IDH1 p.R132H 2C.1 , PIK3CA p.E542V 2C.1 , TP53 p.Q136E 2C.2 | 2C.1 | CDK4 amp 2C.1 | 2C.1 |  | NL | 2C.1 |
| SP2881 | Breast | BREAST | TP53 p.S215I 2C.2 | 2C.2 | MDM4 loss 2D , NTRK1 loss 2C.1 | 2C.1 |  | NL | 2C.1 |
| SP29331 | CNS | CNS | PTEN p.R130G 2C.1 , TP53 p.R248Q 2C.2 | 2C.1 | MDM4 loss 2D , PDGFRA loss 2C.1 | 2C.1 |  | NL | 2C.1 |
| SP29697 | CNS | CNS |  | NL | CDK4 amp 2C.1 , CDK4 amp 2C.1 , EGFR loss 2C.1 , EGFR loss 2C.1 , EGFR loss 2C.1 , EGFR loss 2C.1 , MDM2 amp 2C.2 , MDM2 amp 2C.2 , MDM2 amp 2C.2 , MDM2 amp 2C.2 | 2C.1 | EGFR fusion 2C.1 | 2C.1 | 2C.1 |
| SP29940 | Head/Neck | OTHER | HRAS p.G12S 2C.1 , PPP2R1A p.R183Q 2C.1 | 2C.1 |  | NL |  | NL | 2C.1 |
| SP2997 | Breast | BREAST | NOTCH1 p.E1901K 2C.2 , TP53 p.R175H 2C.2 | 2C.2 | NTRK1 loss 2C.1 | 2C.1 |  | NL | 2C.1 |
| SP29987 | Head/Neck | OTHER | TP53 p.M237V 2C.2 , TP53 p.H179R 2C.2 | 2C.2 | CCND1 amp 2C.2 , FGF19 amp 2D , FGF3 amp 2D | 2C.2 |  | NL | 2C.2 |
| SP30011 | Head/Neck | OTHER |  | NL |  | NL |  | NL | NL |
| SP30071 | Head/Neck | OTHER | ATRX p.A2137T 2C.2 , HRAS p.G13R 2C.1 | 2C.1 |  | NL |  | NL | 2C.1 |
| SP30077 | Head/Neck | OTHER | BRCA1 p.D853N 2C.1 , CDKN2A p.Y129* 2C.1 , TP53 p.R175H 2C.2 | 2C.1 | CCND1 amp 2C.2 , EGFR loss 2C.1 , EGFR loss 2C.1 , FGF19 amp 2D , FGF3 amp 2D | 2C.1 |  | NL | 2C.1 |
| SP30083 | Head/Neck | OTHER | CDKN2A p.R80* 2C.1 , TP53 p.V272L 2C.2 , TP53 p.R273H 2C.2 | 2C.1 |  | NL |  | NL | 2C.1 |
| SP30093 | Head/Neck | OTHER | CDKN2A p.R80* 2C.1 , NOTCH1 p.C381Vfs*250 2C.2 , TP53 p.P177_C182delPHHERC 2C.2 , TP53 p.H193L 2C.2 | 2C.1 | CCND2 amp 2C.2 , IGF1R amp 2D | 2C.2 |  | NL | 2C.1 |
| SP30113 | Head/Neck | OTHER | CDKN2A p.E61* 2C.1 , CREBBP p.V1634L 2C.2 , TP53 p.R156P 2C.2 , TP53 p.C238F 2C.2 | 2C.1 |  | NL |  | NL | 2C.1 |
| SP30143 | Head/Neck | OTHER | NOTCH1 p.C461Y 2C.2 | 2C.2 |  | NL |  | NL | 2C.2 |
| SP3016 | Breast | BREAST | TP53 p.E221* 2C.2 | 2C.2 | MYC amp 2C.2 | 2C.2 |  | NL | 2C.2 |
| SP30185 | Head/Neck | OTHER | PIK3CA p.E545K 2C.1 | 2C.1 |  | NL |  | NL | 2C.1 |
| SP30213 | Head/Neck | OTHER |  | NL |  | NL |  | NL | NL |
| SP30332 | Head/Neck | OTHER | CDKN2A p.R80* 2C.1 , MLH1 p.P138R 1B | 1B | AKT1 amp 2C.2 , BRAF loss 2C.1 , CCND2 amp 2C.2 , EGFR loss 2C.1 , KRAS amp 2C.1 , PIK3CA loss 2C.1 , PIK3CB loss 2C.1 , RICTOR loss 2C.2 | 2C.1 | ERBB2 fusion 2C.1 | 2C.1 | 1B |
| SP30493 | Head/Neck | OTHER | PIK3CA p.E545K 2C.1 | 2C.1 |  | NL |  | NL | 2C.1 |
| SP30675 | Head/Neck | OTHER | NOTCH1 p.G481C 2C.2 , TP53 p.V73Rfs*76 2C.2 , TP53 p.Q136P 2C.2 | 2C.2 | CCND1 amp 2C.2 , EGFR loss 2C.1 , FGF19 amp 2D , FGF3 amp 2D | 2C.1 |  | NL | 2C.1 |
| SP30803 | Head/Neck | OTHER | STK11 p.D194N 2C.1 , STK11 p.G268R 2C.1 , STK11 p.E265Rfs*22 2C.1 | 2C.1 |  | NL |  | NL | 2C.1 |
| SP30843 | Head/Neck | OTHER |  | NL |  | NL |  | NL | NL |
| SP30907 | Head/Neck | OTHER | TP53 p.V274dupV 2C.2 | 2C.2 | CCND1 amp 2C.2 , FGF19 amp 2D , FGF3 amp 2D | 2C.2 |  | NL | 2C.2 |
| SP31030 | Head/Neck | OTHER | PIK3CA p.E545K 2C.1 | 2C.1 |  | NL |  | NL | 2C.1 |
| SP31046 | Head/Neck | OTHER |  | NL |  | NL |  | NL | NL |
| SP31126 | Head/Neck | OTHER | TP53 p.R306* 2C.2 | 2C.2 | FLT3 amp 2C.1 | 2C.1 |  | NL | 2C.1 |
| SP31174 | Head/Neck | OTHER | CDKN2A p.P48L 2C.1 , TP53 p.R248W 2C.2 | 2C.1 | EGFR loss 2C.1 | 2C.1 |  | NL | 2C.1 |
| SP31190 | Head/Neck | OTHER | CDKN2A p.R58* 2C.1 , TP53 p.K101* 2C.2 | 2C.1 | IGF1R amp 2D | 2D |  | NL | 2C.1 |
| SP31334 | Head/Neck | OTHER | ATR p.M2526Rfs*15 2C.2 , NF1 p.S1448R 2C.1 , NOTCH1 p.C739* 2C.2 , NOTCH2 p.D341Y 2C.2 , TP53 p.T256Hfs*8 2C.2 | 2C.1 | CCND1 amp 2C.2 , FGF19 amp 2D , FGF3 amp 2D , PIK3CA loss 2C.1 | 2C.1 |  | NL | 2C.1 |
| SP31606 | Head/Neck | OTHER |  | NL |  | NL |  | NL | NL |
| SP31790 | Head/Neck | OTHER | CDKN2A p.R24*fs*1 2C.1 , NOTCH1 p.R1984* 2C.2 , TP53 p.R282W 2C.2 | 2C.1 |  | NL |  | NL | 2C.1 |
| SP31814 | Head/Neck | OTHER | NF2 p.D268Vfs*29 2C.1 , PIK3CA p.E545K 2C.1 , TP53 p.H193L 2C.2 | 2C.1 |  | NL |  | NL | 2C.1 |
| SP31854 | Head/Neck | OTHER | TP53 p.E294Sfs*51 2C.2 | 2C.2 | CCND1 amp 2C.2 , FGF19 amp 2D , FGF3 amp 2D | 2C.2 |  | NL | 2C.2 |
| SP32014 | Head/Neck | OTHER | TP53 p.H168L 2C.2 | 2C.2 |  | NL |  | NL | 2C.2 |
| SP32222 | Head/Neck | OTHER |  | NL |  | NL |  | NL | NL |
| SP32662 | Head/Neck | OTHER | PIK3CA p.E545K 2C.1 , TP53 p.R175H 2C.2 , TP53 p.R283P 2C.2 | 2C.1 | CCND1 amp 2C.2 , FGF19 amp 2D , FGF3 amp 2D | 2C.2 |  | NL | 2C.1 |
| SP32694 | Head/Neck | OTHER | NOTCH1 p.D352V 2C.2 , RHOA p.E40Q 3 | 2C.2 | CCND1 amp 2C.2 , FGF19 amp 2D , FGF3 amp 2D | 2C.2 |  | NL | 2C.2 |
| SP32742 | Head/Neck | OTHER |  | NL |  | NL |  | NL | NL |
| SP32894 | Head/Neck | OTHER |  | NL |  | NL | FGFR3 fusion 2C.1 | 2C.1 | 2C.1 |
| SP32958 | Head/Neck | OTHER | PIK3CA p.E545K 2C.1 | 2C.1 | PIK3CA loss 2C.1 | 2C.1 |  | NL | 2C.1 |
| SP33094 | Head/Neck | OTHER | TP53 p.I195T 2C.2 | 2C.2 | CCND1 amp 2C.2 , CDK6 loss 2C.1 , EGFR loss 2C.1 , ERBB2 amp 2C.1 , FGF19 amp 2D , FGF3 amp 2D , MET loss 2C.1 | 2C.1 |  | NL | 2C.1 |
| SP33496 | Head/Neck | OTHER | ATRX p.L2364F 2C.2 , NOTCH1 p.E1305K 2C.2 , TP53 p.P278S 2C.2 , TP53 p.Y205*fs*1 2C.2 | 2C.2 | CCND1 amp 2C.2 , CCND1 amp 2C.2 , FGF19 amp 2D , FGF3 amp 2D | 2C.2 |  | NL | 2C.2 |
| SP33544 | Head/Neck | OTHER | PIK3CA p.K111N 2C.1 , POLE p.E537G 2C.1 , TP53 p.R110Wfs*12 2C.2 | 2C.1 |  | NL |  | NL | 2C.1 |
| SP3368 | Breast | BREAST | TP53 p.R248W 2C.2 , TP53 p.L114* 2C.2 | 2C.2 |  | NL |  | NL | 2C.2 |
| SP33688 | Head/Neck | OTHER | DDR2 p.M629V 2C.2 , PIK3CA p.E545K 2C.1 , RET p.T946A 2C.1 | 2C.1 |  | NL |  | NL | 2C.1 |
| SP33774 | Head/Neck | OTHER | AKT1 p.E17K 2C.2 | 2C.2 |  | NL |  | NL | 2C.2 |
| SP33837 | Head/Neck | OTHER |  | NL | MYC amp 2C.2 | 2C.2 |  | NL | 2C.2 |
| SP34005 | Head/Neck | OTHER |  | NL |  | NL |  | NL | NL |
| SP3415 | Breast | BREAST | BRCA1 p.V83F 1A.1 , TP53 p.R282W 2C.2 | 1A.1 | MYC amp 2C.2 , NTRK1 loss 2C.1 | 2C.1 |  | NL | 1A.1 |
| SP34186 | Kidney | OTHER | PTEN p.L146*fs*1 2C.1 , SETD2 p.P1822Qfs*16 2D | 2C.1 |  | NL |  | NL | 2C.1 |
| SP34191 | Kidney | OTHER | AKT2 p.D324H 2C.2 , SETD2 p.E1720* 2D | 2C.2 |  | NL |  | NL | 2C.2 |
| SP34246 | Kidney | OTHER | ATM p.H1083Rfs*7 2C.1 | 2C.1 |  | NL |  | NL | 2C.1 |
| SP34431 | Kidney | OTHER |  | NL |  | NL |  | NL | NL |
| SP34452 | Kidney | OTHER |  | NL |  | NL |  | NL | NL |
| SP34493 | Kidney | OTHER |  | NL |  | NL |  | NL | NL |
| SP35412 | Kidney | OTHER |  | NL |  | NL |  | NL | NL |
| SP35617 | Kidney | OTHER | PIK3CA p.E545K 2C.1 | 2C.1 |  | NL |  | NL | 2C.1 |
| SP35849 | Kidney | OTHER |  | NL |  | NL |  | NL | NL |
| SP35951 | Kidney | OTHER |  | NL |  | NL |  | NL | NL |
| SP35989 | Kidney | OTHER |  | NL |  | NL |  | NL | NL |
| SP36036 | Kidney | OTHER | CDK12 p.I1188Tfs*27 2C.1 , RNF43 p.M173T 2C.2 | 2C.1 |  | NL |  | NL | 2C.1 |
| SP36218 | Kidney | OTHER |  | NL |  | NL |  | NL | NL |
| SP3631 | Breast | BREAST | SETD2 p.S180* 2D , TP53 p.Y205C 2C.2 | 2C.2 | CDK4 amp 2C.1 , ERBB2 amp 1A.1 | 1A.1 |  | NL | 1A.1 |
| SP36498 | Kidney | OTHER |  | NL |  | NL |  | NL | NL |
| SP36586 | Kidney | OTHER |  | NL |  | NL |  | NL | NL |
| SP37369 | Kidney | OTHER |  | NL |  | NL |  | NL | NL |
| SP37432 | Kidney | OTHER | PTEN p.K197Qfs*5 2C.1 | 2C.1 |  | NL |  | NL | 2C.1 |
| SP37516 | Kidney | OTHER | ATM p.T2853R 2C.1 | 2C.1 |  | NL |  | NL | 2C.1 |
| SP37636 | Kidney | OTHER | SETD2 p.L271Mfs*29 2D | 2D |  | NL |  | NL | 2D |
| SP37903 | Kidney | OTHER | POLE p.L1137M 2C.1 | 2C.1 |  | NL |  | NL | 2C.1 |
| SP37970 | Kidney | OTHER | NBN p.P357Qfs*2 2C.1 | 2C.1 |  | NL |  | NL | 2C.1 |
| SP38044 | Kidney | OTHER | SETD2 p.L2124* 2D | 2D |  | NL |  | NL | 2D |
| SP38271 | Kidney | OTHER |  | NL |  | NL |  | NL | NL |
| SP38759 | Kidney | OTHER |  | NL |  | NL |  | NL | NL |
| SP39102 | Kidney | OTHER | SETD2 p.F1650S 2D , SMARCB1 p.Q368* 2D | 2D |  | NL |  | NL | 2D |
| SP39248 | Kidney | OTHER |  | NL |  | NL |  | NL | NL |
| SP39298 | Kidney | OTHER |  | NL |  | NL |  | NL | NL |
| SP39349 | Kidney | OTHER | MTOR p.K1452N 2C.1 | 2C.1 |  | NL |  | NL | 2C.1 |
| SP39594 | Kidney | OTHER |  | NL |  | NL |  | NL | NL |
| SP39907 | Kidney | OTHER |  | NL |  | NL |  | NL | NL |
| SP39997 | Kidney | OTHER |  | NL |  | NL |  | NL | NL |
| SP40047 | Kidney | OTHER |  | NL |  | NL |  | NL | NL |
| SP40736 | Kidney | OTHER |  | NL |  | NL |  | NL | NL |
| SP41453 | Kidney | OTHER |  | NL |  | NL |  | NL | NL |
| SP42154 | Kidney | OTHER | MET p.V1088E 2C.1 | 2C.1 |  | NL |  | NL | 2C.1 |
| SP4265 | Breast | BREAST | TP53 p.R196* 2C.2 | 2C.2 |  | NL | RAD51B fusion 2C.1 | 2C.1 | 2C.1 |
| SP42829 | Kidney | OTHER | NF1 p.L2380R 2C.1 , SETD2 p.P230Tfs*7 2D | 2C.1 |  | NL |  | NL | 2C.1 |
| SP43201 | Kidney | OTHER |  | NL |  | NL |  | NL | NL |
| SP43510 | Kidney | OTHER |  | NL |  | NL |  | NL | NL |
| SP43514 | Kidney | OTHER |  | NL |  | NL |  | NL | NL |
| SP43532 | Kidney | OTHER |  | NL |  | NL |  | NL | NL |
| SP43664 | Kidney | OTHER |  | NL | CDK6 loss 2C.1 , MET loss 2C.1 | 2C.1 |  | NL | 2C.1 |
| SP43688 | Kidney | OTHER |  | NL |  | NL |  | NL | NL |
| SP43696 | Kidney | OTHER |  | NL |  | NL |  | NL | NL |
| SP43770 | Kidney | OTHER | BAP1 p.K425Sfs*5 2C.1 | 2C.1 |  | NL |  | NL | 2C.1 |
| SP43792 | Kidney | OTHER |  | NL |  | NL |  | NL | NL |
| SP43808 | Kidney | OTHER | MET p.V1088E 2C.1 | 2C.1 |  | NL |  | NL | 2C.1 |
| SP43822 | Kidney | OTHER |  | NL | ERBB2 amp 2C.1 , FGFR4 loss 2C.1 | 2C.1 |  | NL | 2C.1 |
| SP4472 | Breast | BREAST | PIK3CA p.H1047R 1A.1 , TP53 p.C238F 2C.2 | 1A.1 | CCND1 amp 2C.2 , FGF19 amp 2D , FGF3 amp 2D | 2C.2 |  | NL | 1A.1 |
| SP4523 | Breast | BREAST |  | NL | CCND1 amp 2C.2 , CCND1 amp 2C.2 , ERBB2 amp 1A.1 , FGF19 amp 2D , FGF3 amp 2D | 1A.1 | ESR1 fusion 2C.1 , NF1 fusion 2C.1 | 2C.1 | 1A.1 |
| SP4535 | Breast | BREAST |  | NL |  | NL |  | NL | NL |
| SP4557 | Breast | BREAST | PIK3CA p.H1047R 1A.1 | 1A.1 |  | NL |  | NL | 1A.1 |
| SP4593 | Breast | BREAST | ARID1A p.D1850Gfs*4 2C.2 | 2C.2 |  | NL |  | NL | 2C.2 |
| SP47628 | CNS | CNS | IDH1 p.R132H 2C.1 , TP53 p.R213* 2C.2 , TP53 p.R282W 2C.2 | 2C.1 |  | NL |  | NL | 2C.1 |
| SP47652 | CNS | CNS | ARID1A p.L1977Rfs*21 2C.2 , IDH1 p.R132H 2C.1 | 2C.1 |  | NL |  | NL | 2C.1 |
| SP47708 | CNS | CNS | IDH1 p.R132H 2C.1 | 2C.1 |  | NL |  | NL | 2C.1 |
| SP47808 | CNS | CNS | IDH2 p.R172M 2C.1 , NOTCH1 p.C129Y 2C.2 | 2C.1 |  | NL |  | NL | 2C.1 |
| SP47990 | CNS | CNS | ATRX p.D699Gfs*2 2C.2 , IDH1 p.R132H 2C.1 , TP53 p.E339* 2C.2 | 2C.1 |  | NL |  | NL | 2C.1 |
| SP48008 | CNS | CNS | IDH1 p.R132H 2C.1 | 2C.1 |  | NL |  | NL | 2C.1 |
| SP48010 | CNS | CNS | ATRX p.V469Ifs*44 2C.2 , IDH1 p.R132H 2C.1 , TP53 p.F109V 2C.2 | 2C.1 |  | NL |  | NL | 2C.1 |
| SP48073 | CNS | CNS | IDH1 p.R132H 2C.1 | 2C.1 |  | NL |  | NL | 2C.1 |
| SP48135 | CNS | CNS | PTEN p.R173C 2C.1 , PTPN11 p.A72D 2C.2 | 2C.1 | MDM4 loss 2D | 2D |  | NL | 2C.1 |
| SP48189 | CNS | CNS | ATRX p.K826Efs*4 2C.2 , IDH1 p.R132H 2C.1 , TP53 p.Q136E 2C.2 | 2C.1 |  | NL |  | NL | 2C.1 |
| SP4820 | Breast | BREAST | RNF43 p.K514Sfs*9 2C.2 | 2C.2 |  | NL |  | NL | 2C.2 |
| SP48263 | CNS | CNS | IDH1 p.R132H 2C.1 | 2C.1 |  | NL |  | NL | 2C.1 |
| SP48414 | CNS | CNS | ATRX p.L359Tfs*3 2C.2 , IDH1 p.R132S 2C.1 , TP53 p.R337C 2C.2 | 2C.1 |  | NL |  | NL | 2C.1 |
| SP48426 | CNS | CNS | IDH1 p.R132H 2C.1 | 2C.1 |  | NL |  | NL | 2C.1 |
| SP48480 | CNS | CNS | IDH1 p.R132H 2C.1 | 2C.1 |  | NL |  | NL | 2C.1 |
| SP48504 | CNS | CNS | IDH1 p.R132H 2C.1 | 2C.1 |  | NL |  | NL | 2C.1 |
| SP48534 | CNS | CNS | IDH1 p.R132H 2C.1 | 2C.1 |  | NL |  | NL | 2C.1 |
| SP4875 | Breast | BREAST | TP53 p.R175H 2C.2 | 2C.2 |  | NL |  | NL | 2C.2 |
| SP48850 | CNS | CNS | ATRX p.K1045*fs*1 2C.2 , IDH1 p.R132H 2C.1 , TP53 p.R273C 2C.2 | 2C.1 | CDK4 amp 2C.1 | 2C.1 |  | NL | 2C.1 |
| SP48888 | CNS | CNS | IDH1 p.R132H 2C.1 | 2C.1 |  | NL |  | NL | 2C.1 |
| SP49114 | Liver | OTHER | TP53 p.Y126D 2C.2 | 2C.2 |  | NL |  | NL | 2C.2 |
| SP49119 | Liver | OTHER |  | NL | MYC amp 2C.2 | 2C.2 |  | NL | 2C.2 |
| SP49124 | Liver | OTHER | ERCC2 p.T484A 2C.2 | 2C.2 |  | NL |  | NL | 2C.2 |
| SP49157 | Liver | OTHER | CTNNB1 p.T41I 2C.2 , SF3B1 p.K666T 3 | 2C.2 |  | NL |  | NL | 2C.2 |
| SP49175 | Liver | OTHER | BAP1 p.V569Cfs*2 2C.1 , IDH1 p.R132C 2C.1 | 2C.1 |  | NL |  | NL | 2C.1 |
| SP49187 | Liver | OTHER | MAP2K4 p.K143Nfs*9 2C.1 | 2C.1 |  | NL |  | NL | 2C.1 |
| SP49205 | Liver | OTHER |  | NL |  | NL |  | NL | NL |
| SP49223 | Liver | OTHER | BAP1 p.Q280* 2C.1 , IDH2 p.R172S 2C.1 , NOTCH2 p.G254R 2C.2 | 2C.1 |  | NL |  | NL | 2C.1 |
| SP49229 | Liver | OTHER | IDH1 p.R132C 2C.1 , RAF1 p.P261R 2C.2 | 2C.1 |  | NL |  | NL | 2C.1 |
| SP49247 | Liver | OTHER | TP53 p.R273H 2C.2 | 2C.2 |  | NL |  | NL | 2C.2 |
| SP49286 | Liver | OTHER | NFE2L2 p.W24_D29delinsY 3 , SETD2 p.T2118I 2D , TP53 p.H193R 2C.2 | 2C.2 |  | NL |  | NL | 2C.2 |
| SP49322 | Liver | OTHER | TP53 p.R248W 2C.2 | 2C.2 |  | NL |  | NL | 2C.2 |
| SP49328 | Liver | OTHER | ARID1A p.Q2039Hfs*60 2C.2 | 2C.2 |  | NL |  | NL | 2C.2 |
| SP49334 | Liver | OTHER | CTNNB1 p.G34E 2C.2 , FANCI p.P998L 2C.1 | 2C.1 |  | NL |  | NL | 2C.1 |
| SP49379 | Liver | OTHER |  | NL |  | NL |  | NL | NL |
| SP49385 | Liver | OTHER |  | NL |  | NL |  | NL | NL |
| SP49391 | Liver | OTHER | TP53 p.E171* 2C.2 | 2C.2 | MYC amp 2C.2 , RICTOR loss 2C.2 , TERT loss 3 | 2C.2 |  | NL | 2C.2 |
| SP49433 | Liver | OTHER | GNAS p.R201C 2C.2 | 2C.2 |  | NL |  | NL | 2C.2 |
| SP49449 | Liver | OTHER | ARID1A p.R1721* 2C.2 | 2C.2 |  | NL |  | NL | 2C.2 |
| SP49469 | Liver | OTHER | CTNNB1 p.K335I 2C.2 | 2C.2 |  | NL |  | NL | 2C.2 |
| SP49481 | Liver | OTHER |  | NL |  | NL |  | NL | NL |
| SP49531 | Liver | OTHER |  | NL | CCND3 loss 2C.2 | 2C.2 |  | NL | 2C.2 |
| SP49541 | Liver | OTHER | TSC1 p.K722N 2C.1 | 2C.1 | MET loss 2C.1 , MET loss 2C.1 | 2C.1 |  | NL | 2C.1 |
| SP49551 | Liver | OTHER | NF2 p.E427* 2C.1 | 2C.1 | MYC amp 2C.2 | 2C.2 |  | NL | 2C.1 |
| SP49591 | Liver | OTHER | TP53 p.A161S 2C.2 | 2C.2 |  | NL |  | NL | 2C.2 |
| SP49651 | Liver | OTHER |  | NL |  | NL |  | NL | NL |
| SP50115 | Liver | OTHER |  | NL |  | NL |  | NL | NL |
| SP5017 | Breast | BREAST | TP53 p.R342* 2C.2 | 2C.2 |  | NL |  | NL | 2C.2 |
| SP50263 | Lung | LUNG | EGFR p.L861Q 1A.1 , RB1 p.E97Nfs*14 2C.2 , TP53 p.R110L 2C.2 | 1A.1 | EGFR loss 2C.1 , EGFR loss 2C.1 , MYC amp 2C.2 | 2C.1 |  | NL | 1A.1 |
| SP50317 | Lung | LUNG | TP53 p.Y220C 2C.2 | 2C.2 | CCND3 loss 2C.2 | 2C.2 |  | NL | 2C.2 |
| SP50321 | Lung | LUNG | KRAS p.G12C 1A.2 | 1A.2 |  | NL |  | NL | 1A.2 |
| SP50406 | Lung | LUNG | KRAS p.G12D 1A.2 | 1A.2 |  | NL |  | NL | 1A.2 |
| SP50412 | Lung | LUNG | SETD2 p.N1535Wfs*7 2D | 2D |  | NL | ROS1 fusion 1A.1 | 1A.1 | 1A.1 |
| SP50485 | Lung | LUNG | CDKN2A p.M54Dfs*66 2C.1 , KRAS p.G12A 1A.2 | 1A.2 | MDM2 amp 2C.2 | 2C.2 |  | NL | 1A.2 |
| SP50518 | Lung | LUNG | EGFR p.L833V 2C.1 , EGFR p.K754_I759delKANKEI 1A.1 , TP53 p.F54Sfs*69 2C.2 | 1A.1 | AKT2 amp 2C.2 , EGFR loss 2C.1 , EGFR loss 2C.1 | 2C.1 |  | NL | 1A.1 |
| SP5052 | Breast | BREAST | FANCA p.R487W 2C.1 , TP53 p.E204* 2C.2 | 2C.1 |  | NL |  | NL | 2C.1 |
| SP50592 | Lung | LUNG |  | NL | AR amp 2C.2 , EGFR loss 2C.1 , MYC amp 2C.2 , NTRK1 loss 2C.1 | 2C.1 |  | NL | 2C.1 |
| SP50611 | Lung | LUNG | SF3B1 p.K741N 3 | 3 | EGFR loss 2C.1 | 2C.1 |  | NL | 2C.1 |
| SP50713 | Lung | LUNG | NOTCH1 p.C1425F 2C.2 , STK11 p.N181I 2C.1 | 2C.1 |  | NL |  | NL | 2C.1 |
| SP50827 | Lung | LUNG | TP53 p.K139Rfs*31 2C.2 | 2C.2 |  | NL |  | NL | 2C.2 |
| SP51037 | Lung | LUNG |  | NL |  | NL |  | NL | NL |
| SP51446 | Lung | LUNG | CTNNB1 p.G34V 2C.2 , EGFR p.E746_A750delELREA 1A.1 | 1A.1 |  | NL |  | NL | 1A.1 |
| SP51824 | Lung | LUNG | BRAF p.G469V 2C.1 , CREBBP p.P10S 2D , CTNNB1 p.T41A 2C.2 | 2C.1 |  | NL |  | NL | 2C.1 |
| SP52232 | Lung | LUNG | ATM p.R1437* 2C.1 , KRAS p.G12V 1A.2 , STK11 p.D53Gfs*110 2C.1 | 1A.2 |  | NL |  | NL | 1A.2 |
| SP52284 | Lung | LUNG | RB1 p.N290Kfs*20 2C.2 , TP53 p.Y163C 2C.2 | 2C.2 | TERT loss 3 | 3 |  | NL | 2C.2 |
| SP52607 | Lung | LUNG | U2AF1 p.S34F 3 | 3 |  | NL | ROS1 fusion 1A.1 | 1A.1 | 1A.1 |
| SP52667 | Lung | LUNG | HRAS p.Q61L 2C.1 , TP53 p.R280I 2C.2 | 2C.1 | CCND3 loss 2C.2 | 2C.2 |  | NL | 2C.1 |
| SP52779 | Lung | LUNG | CREBBP p.Q301H 2D , CTNNB1 p.S37F 2C.2 , KRAS p.G12V 1A.2 | 1A.2 |  | NL |  | NL | 1A.2 |
| SP5279 | Breast | BREAST | TP53 p.V157F 2C.2 | 2C.2 | CCND1 amp 2C.2 , ERBB2 amp 1A.1 , PIK3CA loss 2C.1 , PIK3CA loss 2C.1 | 1A.1 | PIK3CA fusion 2C.1 | 2C.1 | 1A.1 |
| SP53073 | Lung | LUNG | NF1 p.R135Ifs*29 2C.1 , TP53 p.Q192* 2C.2 | 2C.1 |  | NL |  | NL | 2C.1 |
| SP53387 | Lung | LUNG | STK11 p.G288V 2C.1 | 2C.1 | TERT loss 3 | 3 |  | NL | 2C.1 |
| SP53548 | Lung | LUNG | ATM p.R3008H 2C.1 | 2C.1 |  | NL | ALK fusion 1A.1 | 1A.1 | 1A.1 |
| SP53618 | Lung | LUNG | ARID1A p.K1928* 2C.2 , MAP2K1 p.K57N 2C.1 , TP53 p.E224D 2C.2 | 2C.1 | CCND1 amp 2C.2 , FGF19 amp 2D , FLT3 amp 2C.1 | 2C.1 |  | NL | 2C.1 |
| SP5381 | Breast | BREAST | PIK3CA p.E545K 1A.1 | 1A.1 |  | NL |  | NL | 1A.1 |
| SP53810 | Lung | LUNG | BRAF p.G596R 2C.1 , NOTCH1 p.G863V 2C.2 , TP53 p.G262V 2C.2 , TSC2 p.D396Y 2C.1 | 2C.1 |  | NL |  | NL | 2C.1 |
| SP5393 | Breast | BREAST | TP53 p.V122Dfs*26 2C.2 | 2C.2 |  | NL |  | NL | 2C.2 |
| SP54113 | Lung | LUNG | ARID1A p.A160Rfs*67 2C.2 , BRCA2 p.G2063R 2C.1 , PIK3CA p.E545K 2C.1 , RB1 p.D697E 2C.2 , TP53 p.R249Gfs*96 2C.2 | 2C.1 |  | NL |  | NL | 2C.1 |
| SP54363 | Lung | LUNG | KRAS p.G12V 1A.2 , POLE p.K983N 2C.1 , TP53 p.C176Y 2C.2 | 1A.2 |  | NL |  | NL | 1A.2 |
| SP5448 | Breast | BREAST | PIK3CA p.H1047R 1A.1 , TP53 p.Q165Afs*6 2C.2 | 1A.1 | CCND1 amp 2C.2 , ERBB2 amp 1A.1 , FGF19 amp 2D , FGF3 amp 2D , MYC amp 2C.2 | 1A.1 |  | NL | 1A.1 |
| SP5473 | Breast | BREAST | NOTCH1 p.A2441Gfs*39 2C.2 , TP53 p.K292Gfs*52 2C.2 | 2C.2 | AKT2 amp 2C.2 , CCNE1 amp 2C.2 , MYC amp 2C.2 | 2C.2 |  | NL | 2C.2 |
| SP54745 | Lung | LUNG | SMARCA4 p.E1525* 2C.2 | 2C.2 |  | NL |  | NL | 2C.2 |
| SP55004 | Lung | LUNG |  | NL |  | NL |  | NL | NL |
| SP55142 | Lung | LUNG | ARID1A p.W1073Cfs*20 2C.2 , CCND1 p.D25A 2C.2 , CTNNB1 p.S37C 2C.2 , PTCH1 p.W256C 2C.1 , SMARCA4 p.Y1115*fs*1 2C.2 | 2C.1 |  | NL |  | NL | 2C.1 |
| SP55235 | Lung | LUNG | FGFR1 p.D678N 2C.1 , SETD2 p.T305Qfs*35 2D , TP53 p.G105D 2C.2 | 2C.1 | BRAF loss 2C.1 , CCND2 amp 2C.2 , CDK6 loss 2C.1 , EGFR loss 2C.1 , NTRK1 loss 2C.1 | 2C.1 | ERBB4 fusion 2C.1 | 2C.1 | 2C.1 |
| SP55309 | Lung | LUNG |  | NL |  | NL | RET fusion 1A.1 | 1A.1 | 1A.1 |
| SP55387 | Lung | LUNG | TP53 p.Y205F 2C.2 | 2C.2 |  | NL |  | NL | 2C.2 |
| SP55509 | Lung | LUNG | KRAS p.G12C 1A.2 | 1A.2 |  | NL |  | NL | 1A.2 |
| SP5559 | Breast | BREAST | AKT1 p.E17K 2C.2 , ATRX p.R840Nfs*8 2C.2 , TP53 p.R209Kfs*6 2C.2 | 2C.2 |  | NL |  | NL | 2C.2 |
| SP55711 | Lung | LUNG |  | NL | CCNE1 amp 2C.2 , CDK2 amp 2D , CDK4 amp 2C.1 , MDM2 amp 2C.2 , TERT loss 3 , TERT loss 3 | 2C.1 |  | NL | 2C.1 |
| SP56079 | Lung | LUNG | CDK6 p.P113T 2C.1 , CDKN1B p.L13Gfs*111 2D , TP53 p.R248P 2C.2 | 2C.1 | BRAF loss 2C.1 , CDK6 loss 2C.1 , MET loss 2C.1 | 2C.1 |  | NL | 2C.1 |
| SP56168 | Lung | LUNG |  | NL | CDK4 amp 2C.1 , MDM2 amp 2C.2 , TERT loss 3 | 2C.1 |  | NL | 2C.1 |
| SP56303 | Lung | LUNG | EGFR p.E746_A750delELREA 1A.1 , SMARCB1 p.R377H 2D | 1A.1 | CCND1 amp 2C.2 , CDK4 amp 2C.1 , EGFR loss 2C.1 , FGF19 amp 2D , FGFR1 amp 2C.1 , FGFR3 loss 2C.1 , MDM2 amp 2C.2 , NTRK1 loss 2C.1 , RICTOR loss 2C.2 | 2C.1 |  | NL | 1A.1 |
| SP56460 | Lung | LUNG | HIST1H3B p.E74K 3 , NFE2L2 p.G31A 2C.2 , TP53 p.R282Q 2C.2 | 2C.2 |  | NL |  | NL | 2C.2 |
| SP56474 | Lung | LUNG | SMARCA4 p.E1242K 2C.2 , TP53 p.P190L 2C.2 | 2C.2 | CCNE1 amp 2C.2 , TERT loss 3 | 2C.2 |  | NL | 2C.2 |
| SP56502 | Lung | LUNG | PTEN p.Q245* 2C.1 , TP53 p.H193L 2C.2 | 2C.1 |  | NL |  | NL | 2C.1 |
| SP56533 | Lung | LUNG | NFE2L2 p.E79Q 2C.2 , TP53 p.G244S 2C.2 | 2C.2 |  | NL |  | NL | 2C.2 |
| SP56537 | Lung | LUNG | TP53 p.R337L 2C.2 | 2C.2 | PIK3CA loss 2C.1 | 2C.1 |  | NL | 2C.1 |
| SP56541 | Lung | LUNG | RET p.W917R 2C.1 , TP53 p.Q192_H193delQH 2C.2 | 2C.1 |  | NL |  | NL | 2C.1 |
| SP56553 | Lung | LUNG | CDKN2A p.G150S 2C.1 , NF1 p.D1481H 2C.1 , TP53 p.R213* 2C.2 | 2C.1 | CCND1 amp 2C.2 , EGFR loss 2C.1 , FGF19 amp 2D , FGF3 amp 2D , PIK3CA loss 2C.1 | 2C.1 |  | NL | 2C.1 |
| SP56566 | Lung | LUNG | CCND1 p.H181Q 2C.2 , MRE11A p.N354I 2C.1 , NFE2L2 p.R34G 2C.2 , TP53 p.G245C 2C.2 | 2C.1 | CCNE1 amp 2C.2 , ERBB2 amp 2C.1 , FGFR1 amp 2C.1 , MYC amp 2C.2 , PIK3CA loss 2C.1 , PIK3CB loss 2C.1 | 2C.1 |  | NL | 2C.1 |
| SP56569 | Lung | LUNG |  | NL |  | NL |  | NL | NL |
| SP56607 | Lung | LUNG | CDKN2A p.D84N 2C.1 , CREBBP p.K1327* 2D , NFE2L2 p.R34Q 2C.2 , TP53 p.E286K 2C.2 | 2C.1 |  | NL |  | NL | 2C.1 |
| SP56644 | Lung | LUNG | FBXW7 p.S398Y 2C.2 , MAP2K4 p.S184L 2C.1 , NFE2L2 p.R34P 2C.2 | 2C.1 | EGFR loss 2C.1 | 2C.1 |  | NL | 2C.1 |
| SP5666 | Breast | BREAST | AKT1 p.E17K 2C.2 , TP53 p.M246I 2C.2 | 2C.2 |  | NL |  | NL | 2C.2 |
| SP56704 | Lung | LUNG | PIK3CA p.D1045V 2C.1 , TP53 p.G244C 2C.2 | 2C.1 |  | NL |  | NL | 2C.1 |
| SP56730 | Lung | LUNG | NOTCH1 p.Y550N 2C.2 , PTEN p.V166Sfs*14 2C.1 , TP53 p.I162dupI 2C.2 | 2C.1 |  | NL |  | NL | 2C.1 |
| SP56754 | Lung | LUNG | HRAS p.G13R 2C.1 , NFE2L2 p.D29H 2C.2 , TP53 p.G245S 2C.2 | 2C.1 | PIK3CA loss 2C.1 , PIK3CB loss 2C.1 | 2C.1 |  | NL | 2C.1 |
| SP56771 | Lung | LUNG | CDKN2A p.L94Rfs*42 2C.1 , CREBBP p.R1140Q 2D , NFE2L2 p.R34P 2C.2 , TP53 p.C242F 2C.2 | 2C.1 |  | NL |  | NL | 2C.1 |
| SP56821 | Lung | LUNG | ARID1A p.W1844* 2C.2 , TP53 p.S315Lfs*30 2C.2 | 2C.2 | CCND2 amp 2C.2 , KRAS amp 2C.1 , PIK3CA loss 2C.1 | 2C.1 |  | NL | 2C.1 |
| SP56827 | Lung | LUNG | NF1 p.H1943Rfs*17 2C.1 , PTCH1 p.R783L 2C.1 , SMARCA4 p.D694Y 2C.2 , TP53 p.Y163C 2C.2 | 2C.1 | AKT2 amp 2C.2 , AXL amp 2C.2 , CCNE1 amp 2C.2 , PIK3CA loss 2C.1 | 2C.1 |  | NL | 2C.1 |
| SP56941 | Lung | LUNG | ARID1A p.Q449Hfs*171 2C.2 , BAP1 p.R227H 2C.1 , CREBBP p.R1446C 2D , CREBBP p.V1449Ffs*10 2D , FBXW7 p.R465H 2C.2 , FGFR3 p.R248C 2C.1 , PIK3CA p.E542K 2C.1 , PIK3CA p.D538N 2C.1 , RAD51D p.Q130E 2C.1 | 2C.1 |  | NL |  | NL | 2C.1 |
| SP57024 | Lung | LUNG | TP53 p.R248Q 2C.2 | 2C.2 |  | NL |  | NL | 2C.2 |
| SP57066 | Lung | LUNG | CREBBP p.P616R 2D , NFE2L2 p.R34Q 2C.2 , NOTCH1 p.W1075* 2C.2 , TP53 p.R158L 2C.2 | 2C.2 |  | NL |  | NL | 2C.2 |
| SP57084 | Lung | LUNG | CDKN2A p.V59Afs*58 2C.1 , CREBBP p.S646I 2D , TP53 p.R175H 2C.2 | 2C.1 | PIK3CA loss 2C.1 | 2C.1 |  | NL | 2C.1 |
| SP57189 | Lung | LUNG | BRCA1 p.S988* 2C.1 , ERBB4 p.L713F 2C.2 , FGFR2 p.K660E 2C.1 , MYCN p.R398L 3 , PTEN p.G165E 2C.1 , TP53 p.G245V 2C.2 | 2C.1 |  | NL |  | NL | 2C.1 |
| SP57251 | Lung | LUNG | FBXW7 p.R505G 2C.2 , TP53 p.H179R 2C.2 | 2C.2 | CCND1 amp 2C.2 , FGF19 amp 2D , FGF3 amp 2D , FGFR4 loss 2C.1 , NTRK1 loss 2C.1 , PIK3CA loss 2C.1 | 2C.1 |  | NL | 2C.1 |
| SP57267 | Lung | LUNG | CDKN2A p.M53I 2C.1 , CREBBP p.W1472C 2D , NFE2L2 p.Q26L 2C.2 , TP53 p.E68* 2C.2 | 2C.1 | CCND1 amp 2C.2 , FGF19 amp 2D , FGF3 amp 2D | 2C.2 |  | NL | 2C.1 |
| SP57450 | Lung | LUNG | CDKN2A p.D108Y 2C.1 , NFE2L2 p.R34Q 2C.2 , TP53 p.V157F 2C.2 | 2C.1 | CCND2 amp 2C.2 , KRAS amp 2C.1 , MDM2 amp 2C.2 , PIK3CA loss 2C.1 , RICTOR loss 2C.2 | 2C.1 |  | NL | 2C.1 |
| SP57538 | Lung | LUNG | NOTCH1 p.E2075* 2C.2 | 2C.2 | CCND1 amp 2C.2 , FGF19 amp 2D | 2C.2 |  | NL | 2C.2 |
| SP57586 | Lung | LUNG | NFE2L2 p.E79G 2C.2 , NFE2L2 p.E79D 2C.2 , TP53 p.C135F 2C.2 | 2C.2 | CCND1 amp 2C.2 , FGF19 amp 2D , FGF3 amp 2D | 2C.2 |  | NL | 2C.2 |
| SP57619 | Lung | LUNG | NOTCH1 p.K428* 2C.2 , TP53 p.Y220C 2C.2 | 2C.2 |  | NL |  | NL | 2C.2 |
| SP57629 | Lung | LUNG | CDKN2A p.S56Afs*90 2C.1 , NFE2L2 p.W24C 2C.2 | 2C.1 | PIK3CA loss 2C.1 | 2C.1 |  | NL | 2C.1 |
| SP57651 | Lung | LUNG | TP53 p.L265Tfs*7 2C.2 | 2C.2 | PIK3CA loss 2C.1 | 2C.1 |  | NL | 2C.1 |
| SP57669 | Lung | LUNG | FGFR4 p.V262G 2C.1 , NOTCH1 p.A973Qfs*206 2C.2 , TP53 p.Q104* 2C.2 | 2C.1 |  | NL |  | NL | 2C.1 |
| SP57735 | Lung | LUNG | ATM p.G2897S 2C.1 , NOTCH1 p.S1152Afs*27 2C.2 , TP53 p.R175G 2C.2 | 2C.1 | CCND1 amp 2C.2 , FGF19 amp 2D , FGF3 amp 2D , MYC amp 2C.2 | 2C.2 |  | NL | 2C.1 |
| SP57818 | Lung | LUNG | KRAS p.G12A 1A.2 , NFE2L2 p.D29G 2C.2 , NFE2L2 p.R34L 2C.2 , PIK3CA p.E545K 2C.1 | 1A.2 |  | NL |  | NL | 1A.2 |
| SP5784 | Breast | BREAST | PTEN p.D326Efs*4 2C.1 , PTEN p.A126G 2C.1 , TP53 p.W91* 2C.2 | 2C.1 |  | NL |  | NL | 2C.1 |
| SP57846 | Lung | LUNG | CREBBP p.Q503* 2D , HRAS p.Q61K 2C.1 , HRAS p.V14L 2C.1 , SETD2 p.V2370F 2D , TP53 p.G266V 2C.2 | 2C.1 | PIK3CA loss 2C.1 , PIK3CB loss 2C.1 | 2C.1 |  | NL | 2C.1 |
| SP57901 | Lung | LUNG | NFE2L2 p.R34Q 2C.2 , TP53 p.L252_T253delinsP 2C.2 | 2C.2 | PIK3CA loss 2C.1 | 2C.1 |  | NL | 2C.1 |
| SP57933 | Lung | LUNG | BRCA1 p.R320T 2C.1 , BRCA2 p.R2500Sfs*24 2C.1 , NOTCH1 p.V2038L 2C.2 , PIK3CA p.H1047R 2C.1 , TP53 p.I195S 2C.2 | 2C.1 | CCND1 amp 2C.2 , FGF19 amp 2D , FGF3 amp 2D | 2C.2 |  | NL | 2C.1 |
| SP57941 | Lung | LUNG | BRCA1 p.E515V 2C.1 , TP53 p.R175G 2C.2 | 2C.1 | BRAF loss 2C.1 , FGFR1 amp 2C.1 , MYC amp 2C.2 , PIK3CB loss 2C.1 , RICTOR loss 2C.2 , TERT loss 3 | 2C.1 |  | NL | 2C.1 |
| SP5808 | Breast | BREAST | BRCA1 p.E720* 1A.1 | 1A.1 | AKT3 loss 2C.2 , IGF1R amp 2D , IGF1R amp 2D , IGF1R amp 2D , IGF1R amp 2D , MDM4 loss 2D , RICTOR loss 2C.2 | 2C.2 |  | NL | 1A.1 |
| SP58101 | Lung | LUNG | CDKN2A p.Y44*fs*1 2C.1 , NOTCH1 p.R1784L 2C.2 , SMAD4 p.D537E 2C.2 , TP53 p.E221* 2C.2 | 2C.1 |  | NL |  | NL | 2C.1 |
| SP5820 | Breast | BREAST |  | NL | ERBB2 amp 1A.1 , MDM4 loss 2D | 1A.1 |  | NL | 1A.1 |
| SP58245 | Lung | LUNG | NOTCH1 p.R353C 2C.2 , NOTCH1 p.C429S 2C.2 | 2C.2 | FGFR1 amp 2C.1 | 2C.1 |  | NL | 2C.1 |
| SP58326 | Lung | LUNG | NFE2L2 p.D29Y 2C.2 , PDGFRA p.L593I 2C.1 , TP53 p.R248Q 2C.2 | 2C.1 | PIK3CA loss 2C.1 | 2C.1 |  | NL | 2C.1 |
| SP58342 | Lung | LUNG | NOTCH1 p.S2211* 2C.2 , NTRK1 p.G368C 2C.1 , PIK3CA p.E545K 2C.1 , TP53 p.R249G 2C.2 | 2C.1 | AKT2 amp 2C.2 , FGFR1 amp 2C.1 , PIK3CA loss 2C.1 , PIK3CB loss 2C.1 | 2C.1 | FGR fusion 2C.2 | 2C.2 | 2C.1 |
| SP58349 | Lung | LUNG | ARID1A p.A1029Pfs*10 2C.2 , CREBBP p.D1435Y 2D , NOTCH1 p.G1165Qfs*13 2C.2 , TP53 p.H179L 2C.2 | 2C.2 | FGFR3 loss 2C.1 | 2C.1 |  | NL | 2C.1 |
| SP5844 | Breast | BREAST | TP53 p.A86Cfs*63 2C.2 | 2C.2 | FGFR1 amp 2C.1 , FGFR2 amp 2C.1 , MYC amp 2C.2 | 2C.1 |  | NL | 2C.1 |
| SP58612 | Lung | LUNG | NOTCH2 p.G1707Vfs*6 2C.2 , TP53 p.H193D 2C.2 | 2C.2 | PIK3CA loss 2C.1 , PIK3CB loss 2C.1 , RICTOR loss 2C.2 | 2C.1 |  | NL | 2C.1 |
| SP58668 | Lung | LUNG | CDKN2A p.R58* 2C.1 , PIK3CA p.E542K 2C.1 , RET p.L940V 2C.1 | 2C.1 | CCND1 amp 2C.2 , FGF19 amp 2D , FGF3 amp 2D , MET loss 2C.1 | 2C.1 |  | NL | 2C.1 |
| SP58882 | Lung | LUNG | CDKN2A p.A109_P114delAWGRLP 2C.1 , NFE2L2 p.R34G 2C.2 , TP53 p.G105C 2C.2 | 2C.1 | PIK3CA loss 2C.1 | 2C.1 |  | NL | 2C.1 |
| SP58991 | Lung | LUNG | NOTCH1 p.E1719* 2C.2 , SMARCA4 p.A1168Qfs*4 2C.2 , TP53 p.Q38* 2C.2 | 2C.2 | FGFR1 amp 2C.1 | 2C.1 |  | NL | 2C.1 |
| SP59245 | Lung | LUNG | NFE2L2 p.G31A 2C.2 , PIK3CA p.E545K 2C.1 , SMARCB1 p.T149K 2D , TP53 p.H193L 2C.2 | 2C.1 | AKT2 amp 2C.2 , PIK3CA loss 2C.1 | 2C.1 |  | NL | 2C.1 |
| SP59270 | Lymphoid | OTHER | ARID1A p.R1528* 2C.2 , SMARCA4 p.R973Q 2C.2 | 2C.2 |  | NL |  | NL | 2C.2 |
| SP59272 | Lymphoid | OTHER | RHOA p.R5Q 3 , TP53 p.V172D 2C.2 | 2C.2 |  | NL |  | NL | 2C.2 |
| SP59276 | Lymphoid | OTHER | CREBBP p.Q1415* 2C.2 , MYC p.F153C 2C.2 , TP53 p.S215R 2C.2 | 2C.2 |  | NL |  | NL | 2C.2 |
| SP59280 | Lymphoid | OTHER | MYC p.P74S 2C.2 , RHOA p.R5Q 3 , RHOA p.I23R 3 | 2C.2 |  | NL |  | NL | 2C.2 |
| SP59284 | Lymphoid | OTHER | MYC p.Y31H 2C.2 , PIK3CA p.V344G 2C.1 , RHOA p.R5Q 3 , SMARCB1 p.R376G 2D , TP53 p.R273H 2C.2 | 2C.1 |  | NL |  | NL | 2C.1 |
| SP59288 | Lymphoid | OTHER | PTEN . 2C.1 | 2C.1 |  | NL |  | NL | 2C.1 |
| SP59292 | Lymphoid | OTHER |  | NL |  | NL |  | NL | NL |
| SP59296 | Lymphoid | OTHER | PTEN p.R233* 2C.1 , SMARCA4 p.R973W 2C.2 , TP53 p.R273H 2C.2 | 2C.1 |  | NL |  | NL | 2C.1 |
| SP59300 | Lymphoid | OTHER |  | NL |  | NL |  | NL | NL |
| SP59304 | Lymphoid | OTHER | ATR p.S1095* 2C.2 | 2C.2 |  | NL |  | NL | 2C.2 |
| SP59308 | Lymphoid | OTHER |  | NL |  | NL |  | NL | NL |
| SP59312 | Lymphoid | OTHER | NOTCH2 p.R2400* 2C.2 | 2C.2 |  | NL |  | NL | 2C.2 |
| SP59316 | Lymphoid | OTHER |  | NL |  | NL |  | NL | NL |
| SP59320 | Lymphoid | OTHER | CREBBP p.A684Yfs*10 2C.2 | 2C.2 |  | NL |  | NL | 2C.2 |
| SP59324 | Lymphoid | OTHER |  | NL |  | NL |  | NL | NL |
| SP59328 | Lymphoid | OTHER | EZH2 p.Y641H 2C.2 | 2C.2 |  | NL |  | NL | 2C.2 |
| SP59332 | Lymphoid | OTHER | SMARCA4 p.K981E 2C.2 | 2C.2 |  | NL |  | NL | 2C.2 |
| SP59336 | Lymphoid | OTHER | RHOA p.Y42F 3 , SMARCA4 p.R1243W 2C.2 , TP53 p.G245S 2C.2 | 2C.2 |  | NL |  | NL | 2C.2 |
| SP59340 | Lymphoid | OTHER | SMARCA4 p.R1157W 2C.2 | 2C.2 |  | NL |  | NL | 2C.2 |
| SP59344 | Lymphoid | OTHER | ARID1A p.Q1358* 2C.2 | 2C.2 |  | NL |  | NL | 2C.2 |
| SP59348 | Lymphoid | OTHER |  | NL |  | NL |  | NL | NL |
| SP59352 | Lymphoid | OTHER | CREBBP p.R1360* 2C.2 , CREBBP p.I1649Sfs*95 2C.2 , EZH2 p.Y641F 2C.2 | 2C.2 |  | NL |  | NL | 2C.2 |
| SP59356 | Lymphoid | OTHER |  | NL |  | NL |  | NL | NL |
| SP59360 | Lymphoid | OTHER |  | NL |  | NL |  | NL | NL |
| SP59364 | Lymphoid | OTHER |  | NL |  | NL |  | NL | NL |
| SP59368 | Lymphoid | OTHER |  | NL |  | NL |  | NL | NL |
| SP59372 | Lymphoid | OTHER |  | NL |  | NL |  | NL | NL |
| SP59376 | Lymphoid | OTHER |  | NL | CDK6 loss 2C.1 | 2C.1 |  | NL | 2C.1 |
| SP59380 | Lymphoid | OTHER | CREBBP p.P1488L 2C.2 , PIK3R1 p.D708V 2C.2 | 2C.2 |  | NL |  | NL | 2C.2 |
| SP59384 | Lymphoid | OTHER | PIK3R1 p.K567E 2C.2 , TP53 p.P190L 2C.2 | 2C.2 |  | NL |  | NL | 2C.2 |
| SP59388 | Lymphoid | OTHER | MYC p.P72S 2C.2 , SMARCA4 p.G1232S 2C.2 , TP53 p.S241P 2C.2 | 2C.2 |  | NL |  | NL | 2C.2 |
| SP59392 | Lymphoid | OTHER | MYC p.P75A 2C.2 , TP53 p.K139N 2C.2 | 2C.2 |  | NL |  | NL | 2C.2 |
| SP59396 | Lymphoid | OTHER | MTOR p.C1483Y 2C.1 , SMARCA4 p.R973Q 2C.2 , TP53 p.R248Q 2C.2 | 2C.1 |  | NL |  | NL | 2C.1 |
| SP59400 | Lymphoid | OTHER | ATRX p.E1904V 2C.2 , PIK3R1 p.R562C 2C.2 | 2C.2 |  | NL |  | NL | 2C.2 |
| SP59404 | Lymphoid | OTHER | SMARCA4 p.P974S 2C.2 , TP53 p.V218dupV 2C.2 , TP53 p.T284Rfs*21 2C.2 | 2C.2 |  | NL |  | NL | 2C.2 |
| SP59412 | Lymphoid | OTHER | CREBBP p.I340Cfs*9 2C.2 , TP53 p.R273H 2C.2 , XPO1 p.E571K 2C.2 | 2C.2 |  | NL |  | NL | 2C.2 |
| SP59416 | Lymphoid | OTHER | ARID1A p.Q74* 2C.2 , CREBBP p.P704Qfs*9 2C.2 , EZH2 p.Y641S 2C.2 | 2C.2 |  | NL |  | NL | 2C.2 |
| SP59420 | Lymphoid | OTHER | CREBBP p.S802Ffs*30 2C.2 , EZH2 p.Y641F 2C.2 | 2C.2 |  | NL |  | NL | 2C.2 |
| SP59428 | Lymphoid | OTHER |  | NL |  | NL |  | NL | NL |
| SP59432 | Lymphoid | OTHER |  | NL |  | NL |  | NL | NL |
| SP59436 | Lymphoid | OTHER |  | NL |  | NL |  | NL | NL |
| SP59440 | Lymphoid | OTHER |  | NL |  | NL |  | NL | NL |
| SP59444 | Lymphoid | OTHER | EZH2 p.Y641N 2C.2 , SMARCA4 p.G782A 2C.2 | 2C.2 |  | NL |  | NL | 2C.2 |
| SP59448 | Lymphoid | OTHER | IDH1 p.R132H 2C.1 , PTEN p.F21Wfs*24 2C.1 , SMARCA4 p.R973Q 2C.2 , TP53 p.C141Y 2C.2 , XPO1 p.E571G 2C.2 | 2C.1 |  | NL |  | NL | 2C.1 |
| SP59456 | Lymphoid | OTHER |  | NL |  | NL |  | NL | NL |
| SP59460 | Lymphoid | OTHER | ARID1A p.Q1947* 2C.2 , EZH2 p.Y641F 2C.2 , MYD88 p.S219C 2C.2 | 2C.2 |  | NL |  | NL | 2C.2 |
| SP59464 | Lymphoid | OTHER |  | NL |  | NL |  | NL | NL |
| SP5980 | Breast | BREAST | BRCA2 p.E2650Q 1A.1 , CREBBP p.N797Kfs*35 2D | 1A.1 |  | NL |  | NL | 1A.1 |
| SP59803 | Ovary | OVARY | TP53 p.P72Rfs*76 2C.2 | 2C.2 | CCND2 amp 2C.2 | 2C.2 |  | NL | 2C.2 |
| SP59860 | Ovary | OVARY |  | NL | ALK loss 2C.1 , CDK4 amp 2C.1 , KRAS amp 2C.1 , RICTOR loss 2C.2 | 2C.1 |  | NL | 2C.1 |
| SP59938 | Ovary | OVARY |  | NL |  | NL | PTEN fusion 2C.1 | 2C.1 | 2C.1 |
| SP60322 | Ovary | OVARY | BRCA2 p.S1230Lfs*9 1A.1 | 1A.1 |  | NL |  | NL | 1A.1 |
| SP60610 | Ovary | OVARY | NF1 p.V2096Cfs*24 2C.1 | 2C.1 |  | NL |  | NL | 2C.1 |
| SP60842 | Ovary | OVARY | BRCA1 p.S1217Rfs*21 1A.1 , TP53 p.R248G 2C.2 | 1A.1 | MYC amp 2C.2 | 2C.2 | RB1 fusion 2C.2 | 2C.2 | 1A.1 |
| SP6115 | Breast | BREAST | NOTCH2 p.R2400* 2C.2 , TP53 p.R282W 2C.2 | 2C.2 | KIT loss 2C.1 | 2C.1 |  | NL | 2C.1 |
| SP61343 | Ovary | OVARY |  | NL |  | NL |  | NL | NL |
| SP61703 | Ovary | OVARY | TP53 p.C176F 2C.2 | 2C.2 | AKT2 amp 2C.2 , CCNE1 amp 2C.2 , RICTOR loss 2C.2 | 2C.2 |  | NL | 2C.2 |
| SP6223 | Breast | BREAST | TP53 p.S241C 2C.2 | 2C.2 | KRAS amp 2C.1 , MYC amp 2C.2 | 2C.1 |  | NL | 2C.1 |
| SP63716 | Ovary | OVARY | CDK12 p.A88Gfs*30 2C.1 , TP53 p.K132E 2C.2 | 2C.1 | MYC amp 2C.2 | 2C.2 |  | NL | 2C.1 |
| SP63966 | Ovary | OVARY | NOTCH2 p.R1931H 2C.2 , TP53 p.R273H 2C.2 | 2C.2 | AKT2 amp 2C.2 | 2C.2 |  | NL | 2C.2 |
| SP64036 | Ovary | OVARY | SETD2 p.Y2176* 2C.2 , TP53 p.V274G 2C.2 | 2C.2 | IGF1R amp 2D | 2D |  | NL | 2C.2 |
| SP6429 | Breast | BREAST | FBXW7 p.R479Q 2C.2 , TP53 p.R196* 2C.2 | 2C.2 | MYC amp 2C.2 , TERT loss 3 | 2C.2 |  | NL | 2C.2 |
| SP64546 | Ovary | OVARY | TP53 p.R273H 2C.2 | 2C.2 |  | NL |  | NL | 2C.2 |
| SP64976 | Ovary | OVARY | TP53 p.R248Q 2C.2 | 2C.2 | AKT1 amp 2C.2 | 2C.2 |  | NL | 2C.2 |
| SP6519 | Breast | BREAST |  | NL | ERBB2 amp 1A.1 | 1A.1 |  | NL | 1A.1 |
| SP65376 | Ovary | OVARY | PIK3CA p.Q546K 2C.1 | 2C.1 | MYC amp 2C.2 | 2C.2 |  | NL | 2C.1 |
| SP66687 | Ovary | OVARY | PPP2R1A p.R260C 2C.1 , TP53 p.Y220C 2C.2 | 2C.1 |  | NL |  | NL | 2C.1 |
| SP6673 | Breast | BREAST | PIK3CA p.H1047R 1A.1 | 1A.1 |  | NL |  | NL | 1A.1 |
| SP66960 | Ovary | OVARY | TP53 p.P278H 2C.2 | 2C.2 | CCNE1 amp 2C.2 | 2C.2 |  | NL | 2C.2 |
| SP6730 | Breast | BREAST | CDK4 p.K155Q 2C.1 | 2C.1 | ERBB2 amp 1A.1 | 1A.1 |  | NL | 1A.1 |
| SP67428 | Ovary | OVARY | FBXW7 p.E693K 2C.2 , NOTCH1 p.S2516F 2C.2 , TP53 p.E51Gfs*6 2C.2 | 2C.2 |  | NL |  | NL | 2C.2 |
| SP6766 | Breast | BREAST | AKT1 p.E17K 2C.2 , NF1 p.R1250W 2C.1 | 2C.1 |  | NL |  | NL | 2C.1 |
| SP6813 | Breast | BREAST | TP53 p.Y205N 2C.2 | 2C.2 | ERBB2 amp 1A.1 | 1A.1 | ERBB2 fusion 2C.1 | 2C.1 | 1A.1 |
| SP6825 | Breast | BREAST | HIST1H3B p.E74K 3 | 3 | ERBB2 amp 1A.1 , ERBB2 amp 1A.1 | 1A.1 | ERBB2 fusion 2C.1 , NF1 fusion 2C.1 | 2C.1 | 1A.1 |
| SP68348 | Ovary | OVARY | ATR p.H942Y 2C.2 , TP53 p.V272M 2C.2 | 2C.2 |  | NL |  | NL | 2C.2 |
| SP68659 | Ovary | OVARY |  | NL |  | NL |  | NL | NL |
| SP68725 | Ovary | OVARY | TP53 p.H179R 2C.2 | 2C.2 |  | NL |  | NL | 2C.2 |
| SP7291 | Breast | BREAST | AKT1 p.L52R 2C.2 | 2C.2 |  | NL |  | NL | 2C.2 |
| SP7378 | Breast | BREAST | PIK3CA p.N345K 1A.1 | 1A.1 |  | NL |  | NL | 1A.1 |
| SP7421 | Breast | BREAST | FANCD2 p.A731V 2C.1 , NOTCH2 p.P2297Lfs*9 2C.2 , PIK3CA p.E542K 1A.1 , TP53 p.S303Afs*42 2C.2 | 1A.1 |  | NL |  | NL | 1A.1 |
| SP7456 | Breast | BREAST | PIK3CA p.H1047R 1A.1 | 1A.1 | CCND1 amp 2C.2 , CCND1 amp 2C.2 , FGF19 amp 2D , FGF3 amp 2D , MDM4 loss 2D | 2C.2 |  | NL | 1A.1 |
| SP7692 | Breast | BREAST | PIK3CA p.G118D 1A.1 , TP53 p.Y234C 2C.2 | 1A.1 | AR amp 2C.2 , ERBB2 amp 1A.1 | 1A.1 |  | NL | 1A.1 |
| SP7785 | Breast | BREAST | RB1 p.A74Efs*4 2C.2 , TP53 p.T125T 2C.2 | 2C.2 |  | NL |  | NL | 2C.2 |
| SP79907 | Prostate | PROSTATE |  | NL |  | NL |  | NL | NL |
| SP79939 | Prostate | PROSTATE |  | NL |  | NL | ETV4 fusion 2C.2 | 2C.2 | 2C.2 |
| SP79958 | Prostate | PROSTATE |  | NL |  | NL |  | NL | NL |
| SP79968 | Prostate | PROSTATE | SPOP p.W131G 3 | 3 |  | NL |  | NL | 3 |
| SP79971 | Prostate | PROSTATE | KRAS p.G12A 2C.1 | 2C.1 |  | NL | ETV4 fusion 2C.2 | 2C.2 | 2C.1 |
| SP79988 | Prostate | PROSTATE |  | NL |  | NL |  | NL | NL |
| SP79998 | Prostate | PROSTATE |  | NL |  | NL | ERG fusion 2D | 2D | 2D |
| SP80014 | Prostate | PROSTATE |  | NL |  | NL | ERG fusion 2D | 2D | 2D |
| SP80037 | Prostate | PROSTATE |  | NL |  | NL | ERG fusion 2D | 2D | 2D |
| SP80042 | Prostate | PROSTATE |  | NL |  | NL |  | NL | NL |
| SP80160 | Prostate | PROSTATE |  | NL |  | NL | ERG fusion 2D | 2D | 2D |
| SP80183 | Prostate | PROSTATE |  | NL |  | NL | ERG fusion 2D | 2D | 2D |
| SP80205 | Prostate | PROSTATE |  | NL |  | NL |  | NL | NL |
| SP80213 | Prostate | PROSTATE |  | NL |  | NL | ERG fusion 2D | 2D | 2D |
| SP80216 | Prostate | PROSTATE |  | NL |  | NL |  | NL | NL |
| SP80217 | Prostate | PROSTATE |  | NL |  | NL |  | NL | NL |
| SP80244 | Prostate | PROSTATE |  | NL |  | NL | ERG fusion 2D | 2D | 2D |
| SP80271 | Colon/Rectum | COLON/RECTUM | KRAS p.G12C 1A.1 , KRAS p.G12V 1A.1 , PMS2 p.M362I 1B , TP53 p.P151H 2C.2 | 1A.1 |  | NL |  | NL | 1A.1 |
| SP80367 | Colon/Rectum | COLON/RECTUM | TP53 p.R335Qfs*2 2C.2 | 2C.2 |  | NL |  | NL | 2C.2 |
| SP80423 | Colon/Rectum | COLON/RECTUM | PIK3CA p.E545K 2C.1 , TP53 p.Q331* 2C.2 | 2C.1 |  | NL |  | NL | 2C.1 |
| SP80615 | Colon/Rectum | COLON/RECTUM | AKT2 p.D153N 2C.2 , ARID1A p.R1989* 2C.2 , ATM p.L1408I 2C.1 , ATM p.R250* 2C.1 , ATR p.E969* 2C.2 , ATR p.L2393R 2C.2 , ATRX p.R1665I 2C.2 , BRCA1 p.E572* 2C.1 , BRCA2 p.L3180R 2C.1 , BRCA2 p.K2868N 2C.1 , BRCA2 p.S2670L 2C.1 , BRCA2 p.R2494Q 2C.1 , CDK12 p.R890H 2C.1 , CDK12 p.K837N 2C.1 , CDK4 p.R101M 2C.1 , DDR2 p.K616N 2C.2 , ESR1 p.E247K 2C.2 , FANCI p.R533Q 2C.1 , FANCI p.R321I 2C.1 , FBXW7 p.R465H 2C.2 , KDR p.D1064N 2D , KRAS p.A146T 1A.1 , MET p.R1188* 2C.1 , NBN p.F316L 2C.1 , NBN p.E383* 2C.1 , NF1 p.L2367Q 2C.1 , NF1 p.E1266* 2C.1 , NOTCH1 p.A2101T 2C.2 , NOTCH2 p.A1654T 2C.2 , PDGFRA p.D681Y 1A.1 , PIK3R1 p.R348* 2C.2 , PMS2 p.S274L 1B , POLE p.P286R 2C.1 , PTEN p.E299* 2C.1 , PTEN p.E7* 2C.1 , RB1 p.R876C 2C.2 , RB1 p.E322K 2C.2 , RB1 p.E54* 2C.2 , RNF43 p.F279L 2C.2 , SETD2 p.R53Q 2D , SETD2 p.D350Y 2D , SMARCA4 p.R1336H 2C.2 , SMARCA4 p.D1169Y 2C.2 , TP53 p.R213* 2C.2 , TSC1 p.K167N 2C.1 , TSC1 p.M223I 2C.1 | 1A.1 |  | NL |  | NL | 1A.1 |
| SP80657 | Colon/Rectum | COLON/RECTUM | KRAS p.A146T 1A.1 , PIK3CA p.V344G 2C.1 , PTEN p.Y68N 2C.1 , SMAD4 p.P356L 2C.2 | 1A.1 |  | NL |  | NL | 1A.1 |
| SP80754 | Colon/Rectum | COLON/RECTUM | ATM p.E2039K 2C.1 , ATM p.D1682Y 2C.1 , FBXW7 p.R505C 2C.2 , KRAS p.K117N 1A.1 , TP53 p.G262V 2C.2 | 1A.1 | FLT3 amp 2C.1 | 2C.1 |  | NL | 1A.1 |
| SP8085 | Breast | BREAST | PIK3CA p.H1047R 1A.1 , TP53 p.N131Tfs*39 2C.2 | 1A.1 | ESR1 loss 2C.2 , MYC amp 2C.2 , PPARG loss 3 | 2C.2 | ESR1 fusion 2C.1 | 2C.1 | 1A.1 |
| SP80950 | Colon/Rectum | COLON/RECTUM |  | NL |  | NL |  | NL | NL |
| SP81137 | Colon/Rectum | COLON/RECTUM | TP53 p.G226Rfs*23 2C.2 | 2C.2 |  | NL |  | NL | 2C.2 |
| SP81440 | Colon/Rectum | COLON/RECTUM | KRAS p.G12C 1A.1 , TP53 p.R248W 2C.2 | 1A.1 | FLT3 amp 2C.1 | 2C.1 |  | NL | 1A.1 |
| SP81494 | Colon/Rectum | COLON/RECTUM | ATRX p.D2213Y 2C.2 , KRAS p.G12D 1A.1 , NF2 p.R516L 2C.1 , NF2 p.R516W 2C.1 , NRAS p.G12C 1.A.1 , SETD2 p.R2399* 2D , TP53 p.I251F 2C.2 | 1A.1 |  | NL |  | NL | 1A.1 |
| SP8157 | Breast | BREAST |  | NL | CCNE1 amp 2C.2 , FGFR1 amp 2C.1 | 2C.1 |  | NL | 2C.1 |
| SP81711 | Colon/Rectum | COLON/RECTUM | SMARCA4 p.I996F 2C.2 , TP53 p.S127P 2C.2 | 2C.2 |  | NL |  | NL | 2C.2 |
| SP81840 | Colon/Rectum | COLON/RECTUM | ATM p.R250* 2C.1 , KRAS p.G13D 1A.1 , MAP2K4 p.R134W 2C.1 | 1A.1 |  | NL |  | NL | 1A.1 |
| SP82087 | Colon/Rectum | COLON/RECTUM | AR p.F828L 2C.2 , KRAS p.G12C 1A.1 , SETD2 p.R404I 2D , TP53 p.A159V 2C.2 | 1A.1 |  | NL |  | NL | 1A.1 |
| SP82103 | Colon/Rectum | COLON/RECTUM | ARID1A p.G1729Wfs*7 2C.2 | 2C.2 |  | NL |  | NL | 2C.2 |
| SP8229 | Breast | BREAST | TP53 p.R213* 2C.2 | 2C.2 | FGFR1 amp 2C.1 | 2C.1 |  | NL | 2C.1 |
| SP82399 | Skin | SKIN | BRAF p.V600E 1A.1 , CDKN2A p.P81L 2C.1 | 1A.1 |  | NL |  | NL | 1A.1 |
| SP82417 | Skin | SKIN | BRAF p.V600M 1A.1 , BRAF p.V600E 1A.1 , BRCA1 p.E272K 2C.1 , SF3B1 p.R625C 3 , SMARCA4 p.P1536L 2C.2 | 1A.1 |  | NL |  | NL | 1A.1 |
| SP82429 | Skin | SKIN | BRAF p.V600E 1A.1 | 1A.1 | EGFR loss 2C.1 | 2C.1 |  | NL | 1A.1 |
| SP82431 | Skin | SKIN | ATR p.D1298N 2C.2 , BRAF p.V600E 1A.1 , CDKN2A p.R58Afs*57 2C.1 , FGFR1 p.K450E 2C.1 , MAP2K1 p.P124S 2C.1 , PIK3R1 p.P89L 2C.2 , POLE p.R2145* 2C.1 , RAC1 p.P29S 3 , SMARCA4 p.E1435G 2C.2 | 1A.1 |  | NL |  | NL | 1A.1 |
| SP82433 | Skin | SKIN | BRAF p.V600M 1A.1 , BRAF p.V600E 1A.1 , PTEN p.V166Sfs*14 2C.1 | 1A.1 |  | NL |  | NL | 1A.1 |
| SP82435 | Skin | SKIN | BRAF p.V600E 1A.1 , CDK6 p.P199L 2C.1 | 1A.1 |  | NL |  | NL | 1A.1 |
| SP82445 | Skin | SKIN | FBXW7 p.S601F 2C.2 , MET p.L1158F 2C.1 , NF1 p.R2517* 2C.1 , RAC1 p.P34S 3 , TP53 p.A159V 2C.2 , TP53 p.R290C 2C.2 | 2C.1 |  | NL |  | NL | 2C.1 |
| SP82451 | Skin | SKIN | BRAF p.V600E 1A.1 | 1A.1 | BRAF loss 2C.1 , CDK6 loss 2C.1 , MET loss 2C.1 | 2C.1 |  | NL | 1A.1 |
| SP82459 | Skin | SKIN | BRAF p.L597Q 2C.1 , ERBB4 p.G456E 2C.2 , MAP2K4 p.L146F 2C.1 , POLE p.L607F 2C.1 | 2C.1 |  | NL |  | NL | 2C.1 |
| SP82461 | Skin | SKIN | BRAF p.K601E 1A.1 , KDR p.G493R 2D , NOTCH2 p.K2127* 2C.2 , SMARCA4 p.H884Y 2C.2 | 1A.1 |  | NL |  | NL | 1A.1 |
| SP82471 | Skin | SKIN | FANCD2 p.M197I 2C.1 , NRAS p.G12R 2C.1 | 2C.1 | CDK4 amp 2C.1 , KRAS amp 2C.1 | 2C.1 |  | NL | 2C.1 |
| SP82532 | Skin | SKIN | KRAS p.G12D 2C.1 | 2C.1 | CCND1 amp 2C.2 , FGF19 amp 2D | 2C.2 |  | NL | 2C.1 |
| SP82614 | Skin | SKIN | BRAF p.V600E 1A.1 , FBXW7 p.H500Y 2C.2 | 1A.1 |  | NL |  | NL | 1A.1 |
| SP82636 | Skin | SKIN | KIT p.N822K 1B | 1B | CDK4 amp 2C.1 | 2C.1 |  | NL | 1B |
| SP82644 | Skin | SKIN | EGFR p.P622S 2C.1 | 2C.1 |  | NL | RAF1 fusion 2C.1 | 2C.1 | 2C.1 |
| SP82756 | Skin | SKIN |  | NL |  | NL | RAF1 fusion 2C.1 | 2C.1 | 2C.1 |
| SP82780 | Skin | SKIN | CDKN2A p.R80* 2C.1 , NRAS p.Q61K 2C.1 | 2C.1 |  | NL |  | NL | 2C.1 |
| SP82796 | Skin | SKIN | NRAS p.Q61K 2C.1 , NRAS p.Q61R 2C.1 | 2C.1 |  | NL |  | NL | 2C.1 |
| SP82836 | Skin | SKIN | AKT1 p.H194Y 2C.2 , BRAF p.V600E 1A.1 , NOTCH2 p.P690S 2C.2 , PTEN p.L108P 2C.1 | 1A.1 |  | NL |  | NL | 1A.1 |
| SP82900 | Skin | SKIN | CDKN2A p.P48L 2C.1 , FANCI p.S708F 2C.1 , NRAS p.Q61K 2C.1 , TP53 p.R280K 2C.2 | 2C.1 |  | NL |  | NL | 2C.1 |
| SP82988 | Skin | SKIN | ATRX p.R2153C 2C.2 , IDH1 p.R132C 2C.1 , NF1 p.R1362* 2C.1 , NRAS p.Q61L 2C.1 , RAC1 p.P29S 3 | 2C.1 |  | NL |  | NL | 2C.1 |
| SP83019 | Skin | SKIN | BRAF p.V600M 1A.1 , BRAF p.V600E 1A.1 , NF1 p.S2496F 2C.1 , STK11 p.K296Rfs*40 2C.1 | 1A.1 |  | NL |  | NL | 1A.1 |
| SP83027 | Skin | SKIN |  | NL | MDM4 loss 2D , MYC amp 2C.2 | 2C.2 | BRAF fusion 2C.1 | 2C.1 | 2C.1 |
| SP83083 | Skin | SKIN | NRAS p.Q61K 2C.1 | 2C.1 |  | NL |  | NL | 2C.1 |
| SP83099 | Skin | SKIN | BRAF p.V600E 1A.1 | 1A.1 |  | NL |  | NL | 1A.1 |
| SP83146 | Skin | SKIN | SMO p.R199W 2C.1 , TP53 p.Q317* 2C.2 | 2C.1 |  | NL |  | NL | 2C.1 |
| SP83242 | Skin | SKIN | BRAF p.V600E 1A.1 , NOTCH1 p.P362L 2C.2 | 1A.1 |  | NL |  | NL | 1A.1 |
| SP83312 | Skin | SKIN | BRAF p.V600E 1A.1 , CDKN2A p.R80* 2C.1 | 1A.1 |  | NL |  | NL | 1A.1 |
| SP83382 | Skin | SKIN | ATM p.E166Gfs*2 2C.1 , NRAS p.Q61K 2C.1 , PTCH1 p.P681L 2C.1 , PTEN p.E256* 2C.1 | 2C.1 |  | NL |  | NL | 2C.1 |
| SP83482 | Skin | SKIN | BRAF p.V600M 1A.1 , BRAF p.V600E 1A.1 , TP53 p.R342* 2C.2 | 1A.1 |  | NL |  | NL | 1A.1 |
| SP83844 | Skin | SKIN | BRAF p.V600E 1A.1 , KDR p.D1052N 2D , TP53 p.K120E 2C.2 | 1A.1 |  | NL |  | NL | 1A.1 |
| SP8394 | Breast | BREAST | PIK3CA p.E542K 1A.1 , TP53 p.Q331* 2C.2 | 1A.1 | EGFR loss 2C.1 , ERBB2 amp 1A.1 | 1A.1 |  | NL | 1A.1 |
| SP83967 | Stomach | OTHER | ARID1A p.L2016Qfs*8 2C.2 , ARID1A p.Q1512* 2C.2 , PIK3CA p.E542K 2C.1 | 2C.1 | MDM2 amp 2C.2 | 2C.2 |  | NL | 2C.1 |
| SP84056 | Stomach | OTHER | CDKN2A p.R112Vfs*34 2C.1 | 2C.1 |  | NL |  | NL | 2C.1 |
| SP84062 | Stomach | OTHER | SMAD4 p.R361_L364delRFCL 2C.2 | 2C.2 | CDK6 loss 2C.1 | 2C.1 |  | NL | 2C.1 |
| SP84392 | Stomach | OTHER | ARID1A p.T294Pfs*69 2C.2 , ATR p.R1814Efs*10 2C.2 , BRCA1 p.R1397M 2C.1 , CDK12 p.G923V 2C.1 , FANCA p.K453N 2C.1 , KRAS p.G13D 2C.1 , MRE11A p.E10G 2C.1 , NF1 p.I679Dfs*21 2C.1 , NOTCH1 p.R1937H 2C.2 , PIK3CA p.H1047R 2C.1 , RNF43 p.G659Vfs*41 2C.2 | 2C.1 |  | NL |  | NL | 2C.1 |
| SP84408 | Stomach | OTHER | ARID1A p.Q1333Afs*5 2C.2 , ARID1A p.E33Gfs*62 2C.2 , RHOA p.F39C 3 , SMAD4 p.C363Y 2C.2 | 2C.2 |  | NL |  | NL | 2C.2 |
| SP84439 | Stomach | OTHER | ARID1A p.D1850Tfs*33 2C.2 , ATM p.R270M 2C.1 , BRCA2 p.T3033Lfs*29 2C.1 , FANCA p.A786V 2C.1 , FGFR2 p.D656G 2C.1 , MRE11A p.R364* 2C.1 , NF2 p.C133Y 2C.1 , NOTCH1 p.G317C 2C.2 , PIK3CA p.H1047R 2C.1 , POLE p.V474I 2C.1 , POLE p.R1111Q 2C.1 , POLE p.Y1889C 2C.1 , PTCH1 p.L590F 2C.1 , PTCH1 p.A918T 2C.1 , PTEN p.I33delI 2C.1 , RNF43 p.G659Vfs*41 2C.2 , SMARCA4 p.A1002V 2C.2 , SMARCA4 p.G630D 2C.2 | 2C.1 |  | NL |  | NL | 2C.1 |
| SP84491 | Stomach | OTHER | CTNNB1 p.S37F 2C.2 , CTNNB1 p.T339N 2C.2 , TP53 p.T284Kfs*61 2C.2 | 2C.2 |  | NL |  | NL | 2C.2 |
| SP84719 | Stomach | OTHER | PIK3CA p.E542K 2C.1 , SMARCA4 p.G782S 2C.2 | 2C.1 |  | NL |  | NL | 2C.1 |
| SP84743 | Stomach | OTHER | PIK3R1 p.M206Ifs*4 2C.2 , PTEN p.L42P 2C.1 , ROS1 p.C2067G 2C.1 , TP53 p.R273H 2C.2 | 2C.1 | CCND3 loss 2C.2 , CCNE1 amp 2C.2 | 2C.2 |  | NL | 2C.1 |
| SP84858 | Stomach | OTHER | KRAS p.G12C 2C.1 , PIK3CA p.E545K 2C.1 , TP53 p.R175H 2C.2 | 2C.1 | ERBB2 amp 1A.1 | 1A.1 | ERBB2 fusion 2C.1 | 2C.1 | 1A.1 |
| SP84962 | Stomach | OTHER | ARID1A p.A1517Pfs*10 2C.2 , TP53 p.H193R 2C.2 | 2C.2 |  | NL |  | NL | 2C.2 |
| SP84982 | Stomach | OTHER | BAP1 p.E198Rfs*33 2C.1 , KRAS p.G13D 2C.1 , SETD2 p.T1652Yfs*14 2D | 2C.1 |  | NL |  | NL | 2C.1 |
| SP84998 | Stomach | OTHER |  | NL |  | NL |  | NL | NL |
| SP85130 | Stomach | OTHER |  | NL |  | NL |  | NL | NL |
| SP85222 | Stomach | OTHER | CDKN2A p.H83Y 2C.1 , SMAD4 p.R361_G365delRFCLG 2C.2 , TP53 p.R248Q 2C.2 | 2C.1 | CCND1 amp 2C.2 , CDK6 loss 2C.1 , ERBB2 amp 1A.1 , ERBB2 amp 1A.1 , FGF19 amp 2D , FGF3 amp 2D | 1A.1 | ERBB2 fusion 2C.1 | 2C.1 | 1A.1 |
| SP85230 | Stomach | OTHER | BRCA1 p.D1692N 2C.1 | 2C.1 |  | NL |  | NL | 2C.1 |
| SP85251 | Stomach | OTHER | TP53 p.H214R 2C.2 | 2C.2 | KRAS amp 2C.1 | 2C.1 |  | NL | 2C.1 |
| SP8532 | Breast | BREAST | CDK12 p.R902P 2C.1 , TP53 p.I255delI 2C.2 | 2C.1 | FGFR1 amp 2C.1 | 2C.1 |  | NL | 2C.1 |
| SP85339 | Stomach | OTHER | TP53 p.R282W 2C.2 | 2C.2 |  | NL |  | NL | 2C.2 |
| SP85379 | Stomach | OTHER | CDK12 p.R779H 2C.1 , SMARCA4 p.R468H 2C.2 | 2C.1 | MYC amp 2C.2 | 2C.2 |  | NL | 2C.1 |
| SP85487 | Thyroid | OTHER | NRAS p.Q61K 2C.1 | 2C.1 |  | NL |  | NL | 2C.1 |
| SP85491 | Thyroid | OTHER |  | NL |  | NL | RET fusion 2C.1 | 2C.1 | 2C.1 |
| SP85495 | Thyroid | OTHER | BRAF p.V600E 1A.1 | 1A.1 |  | NL |  | NL | 1A.1 |
| SP85511 | Thyroid | OTHER | BRAF p.V600E 1A.1 | 1A.1 |  | NL |  | NL | 1A.1 |
| SP85582 | Thyroid | OTHER | BRAF p.V600E 1A.1 | 1A.1 |  | NL |  | NL | 1A.1 |
| SP85623 | Thyroid | OTHER |  | NL |  | NL | RET fusion 2C.1 | 2C.1 | 2C.1 |
| SP8564 | Breast | BREAST |  | NL | ERBB2 amp 1A.1 | 1A.1 | ESR1 fusion 2C.1 | 2C.1 | 1A.1 |
| SP85647 | Thyroid | OTHER |  | NL |  | NL |  | NL | NL |
| SP85725 | Thyroid | OTHER |  | NL |  | NL |  | NL | NL |
| SP85733 | Thyroid | OTHER |  | NL |  | NL |  | NL | NL |
| SP85787 | Thyroid | OTHER |  | NL |  | NL |  | NL | NL |
| SP85818 | Thyroid | OTHER |  | NL |  | NL |  | NL | NL |
| SP85836 | Thyroid | OTHER |  | NL |  | NL |  | NL | NL |
| SP85840 | Thyroid | OTHER |  | NL |  | NL |  | NL | NL |
| SP85864 | Thyroid | OTHER | BRAF p.V600E 1A.1 , NOTCH2 p.C877Y 2C.2 | 1A.1 |  | NL |  | NL | 1A.1 |
| SP85952 | Thyroid | OTHER |  | NL |  | NL | MET fusion 2C.1 | 2C.1 | 2C.1 |
| SP86118 | Thyroid | OTHER |  | NL |  | NL |  | NL | NL |
| SP86130 | Thyroid | OTHER |  | NL |  | NL | ALK fusion 2C.1 | 2C.1 | 2C.1 |
| SP86306 | Thyroid | OTHER | HRAS p.Q61R 2C.1 | 2C.1 |  | NL |  | NL | 2C.1 |
| SP86425 | Thyroid | OTHER | BRAF p.V600E 1A.1 | 2C.1 |  | NL |  | NL | 2C.1 |
| SP8660 | Breast | BREAST | TP53 p.T155P 2C.2 | 2C.2 |  | NL |  | NL | 2C.2 |
| SP86660 | Thyroid | OTHER | AKT1 p.E17K 2C.2 , BRAF p.V600E 1A.1 | 1A.1 |  | NL |  | NL | 1A.1 |
| SP86775 | Thyroid | OTHER |  | NL |  | NL | RET fusion 2C.1 | 2C.1 | 2C.1 |
| SP86836 | Thyroid | OTHER |  | NL |  | NL |  | NL | NL |
| SP86929 | Thyroid | OTHER | BRAF p.V600E 1A.1 | 1A.1 |  | NL |  | NL | 1A.1 |
| SP86989 | Thyroid | OTHER |  | NL |  | NL | RET fusion 1A.1 | 1A.1 | 1A.1 |
| SP87099 | Thyroid | OTHER |  | NL |  | NL |  | NL | NL |
| SP87337 | Thyroid | OTHER |  | NL |  | NL | RET fusion 1A.1 | 1A.1 | 1A.1 |
| SP87434 | Thyroid | OTHER |  | NL |  | NL |  | NL | NL |
| SP87446 | Thyroid | OTHER | BRAF p.V600E 1A.1 | 1A.1 |  | NL |  | NL | 1A.1 |
| SP87534 | Thyroid | OTHER | NRAS p.Q61R 2C.1 , TP53 p.T125T 2C.2 | 2C.1 |  | NL |  | NL | 2C.1 |
| SP87582 | Thyroid | OTHER |  | NL |  | NL | RET fusion 1A.1 | 1A.1 | 1A.1 |
| SP87675 | Thyroid | OTHER |  | NL |  | NL |  | NL | NL |
| SP87903 | Thyroid | OTHER |  | NL |  | NL | ALK fusion 2C.1 | 2C.1 | 2C.1 |
| SP8795 | Breast | BREAST | FANCD2 p.F843I 2C.1 , TP53 p.R248Q 2C.2 | 2C.1 | MYC amp 2C.2 | 2C.2 |  | NL | 2C.1 |
| SP88050 | Thyroid | OTHER | BRAF p.V600E 1A.1 | 1A.1 |  | NL |  | NL | 1A.1 |
| SP88098 | Thyroid | OTHER |  | NL |  | NL |  | NL | NL |
| SP88158 | Thyroid | OTHER |  | NL |  | NL | NTRK3 fusion 1A.1 | 1A.1 | 1A.1 |
| SP8831 | Breast | BREAST | FBXW7 p.Y545C 2C.2 , TP53 p.R110Sfs*14 2C.2 | 2C.2 | FGFR1 amp 2C.1 , MYC amp 2C.2 | 2C.1 |  | NL | 2C.1 |
| SP88322 | Thyroid | OTHER |  | NL |  | NL |  | NL | NL |
| SP88593 | Thyroid | OTHER |  | NL |  | NL |  | NL | NL |
| SP88757 | Thyroid | OTHER |  | NL |  | NL |  | NL | NL |
| SP88776 | Thyroid | OTHER |  | NL |  | NL |  | NL | NL |
| SP8891 | Breast | BREAST | ATRX p.D139Mfs*14 2C.2 , PIK3CA p.E453K 1A.1 | 1A.1 | MYC amp 2C.2 | 2C.2 |  | NL | 1A.1 |
| SP89090 | Thyroid | OTHER |  | NL |  | NL |  | NL | NL |
| SP89245 | Thyroid | OTHER |  | NL |  | NL |  | NL | NL |
| SP89291 | Thyroid | OTHER |  | NL |  | NL | BRAF fusion 2C.1 | 2C.1 | 2C.1 |
| SP89389 | Uterus | OTHER | PPP2R1A p.S256F 2C.1 , TP53 p.Q331* 2C.2 | 2C.1 | CCNE1 amp 2C.2 , EGFR loss 2C.1 , EGFR loss 2C.1 | 2C.1 |  | NL | 2C.1 |
| SP89443 | Uterus | OTHER | PPP2R1A p.P179R 2C.1 , TP53 p.P151S 2C.2 | 2C.1 | PIK3CA loss 2C.1 | 2C.1 |  | NL | 2C.1 |
| SP89519 | Uterus | OTHER | PTEN p.S170N 2C.1 , PTEN p.R130P 2C.1 | 2C.1 |  | NL |  | NL | 2C.1 |
| SP89651 | Uterus | OTHER | PIK3CA p.R88Q 2C.1 , TP53 p.G262V 2C.2 | 2C.1 |  | NL |  | NL | 2C.1 |
| SP89687 | Uterus | OTHER | PIK3CA p.H1047L 2C.1 , TP53 p.R248W 2C.2 | 2C.1 | PPARG loss 3 | 3 |  | NL | 2C.1 |
| SP8987 | Breast | BREAST | NOTCH2 p.P2189Ffs*34 2C.2 , TP53 p.T211Lfs*36 2C.2 | 2C.2 |  | NL |  | NL | 2C.2 |
| SP89909 | Uterus | OTHER | ARID1A p.K1094Sfs*67 2C.2 , ATM p.V2830D 2C.1 , DDR2 p.W756C 2C.2 , PIK3CA p.E542K 2C.1 , PTEN p.C136Mfs*8 2C.1 | 2C.1 |  | NL |  | NL | 2C.1 |
| SP89957 | Uterus | OTHER | PIK3R1 p.R348* 2C.2 | 2C.2 | CCNE1 amp 2C.2 , MYC amp 2C.2 | 2C.2 |  | NL | 2C.2 |
| SP90125 | Uterus | OTHER | TP53 p.G266V 2C.2 | 2C.2 | CCNE1 amp 2C.2 , MYC amp 2C.2 | 2C.2 |  | NL | 2C.2 |
| SP90245 | Uterus | OTHER | ATM p.R1466P 2C.1 , PIK3R1 p.R386* 2C.2 , TP53 p.C277dupC 2C.2 | 2C.1 | AKT2 amp 2C.2 , CCNE1 amp 2C.2 , ERBB2 amp 2C.1 , FGFR3 loss 2C.1 | 2C.1 |  | NL | 2C.1 |
| SP90269 | Uterus | OTHER | PIK3CA p.R93Q 2C.1 , TP53 p.R306* 2C.2 | 2C.1 |  | NL |  | NL | 2C.1 |
| SP90503 | Uterus | OTHER | FGFR2 p.N550K 2C.1 , PPP2R1A p.P179R 2C.1 , TP53 p.S241C 2C.2 | 2C.1 | MYC amp 2C.2 , RICTOR loss 2C.2 , TERT loss 3 | 2C.2 |  | NL | 2C.1 |
| SP90629 | Uterus | OTHER | FBXW7 p.R505G 2C.2 , PIK3CA p.E545K 2C.1 , PIK3CA p.E453Q 2C.1 , SETD2 p.R1496Q 2D , TP53 p.R249S 2C.2 | 2C.1 | CDK2 amp 2D , ERBB2 amp 2C.1 , ERBB2 amp 2C.1 , PIK3CA loss 2C.1 | 2C.1 |  | NL | 2C.1 |
| SP90725 | Uterus | OTHER | AKT1 p.E17K 2C.2 , ARID1A p.E1733* 2C.2 , BRCA2 p.S1882* 2C.1 , CTNNB1 p.G34R 2C.2 , MTOR p.D2512Y 2C.1 | 2C.1 |  | NL |  | NL | 2C.1 |
| SP90893 | Uterus | OTHER | FBXW7 p.R465H 2C.2 , MSH2 . 1B , MTOR p.D2424H 2C.1 , TP53 p.Y205C 2C.2 | 2C.1 | ERBB2 amp 2C.1 | 2C.1 |  | NL | 2C.1 |
| SP91265 | Uterus | OTHER | PTCH1 p.L1024I 2C.1 , PTEN p.R130L 2C.1 , TP53 p.R282W 2C.2 | 2C.1 | FGFR1 amp 2C.1 , FGFR1 amp 2C.1 , KIT loss 2C.1 , PDGFRA loss 2C.1 , TERT loss 3 | 2C.1 | ESR1 fusion 2C.1 | 2C.1 | 2C.1 |
| SP91666 | Uterus | OTHER | FBXW7 p.R224* 2C.2 , SPOP p.M117V 3 , TP53 p.Q144* 2C.2 | 2C.2 | MYC amp 2C.2 | 2C.2 |  | NL | 2C.2 |
| SP91730 | Uterus | OTHER | TP53 p.R249W 2C.2 | 2C.2 |  | NL | MET fusion 2C.1 | 2C.1 | 2C.1 |
| SP91746 | Uterus | OTHER | PIK3CA p.N345I 2C.1 , PPP2R1A p.P179R 2C.1 , TP53 p.R248W 2C.2 | 2C.1 | CCNE1 amp 2C.2 | 2C.2 |  | NL | 2C.1 |
| SP92195 | Uterus | OTHER | SPOP p.D140G 3 , TP53 p.R273H 2C.2 | 2C.2 | MYC amp 2C.2 | 2C.2 |  | NL | 2C.2 |
| SP92268 | Uterus | OTHER | ARID1A p.H415Pfs*205 2C.2 , CCND1 p.P287A 2C.2 , CTNNB1 p.S45F 2C.2 , KRAS p.G13C 2C.1 , PIK3CA p.E545K 2C.1 , PTEN p.L152P 2C.1 | 2C.1 |  | NL |  | NL | 2C.1 |
| SP92332 | Uterus | OTHER | ARID1A p.N917Ifs*2 2C.2 , PTEN p.G132D 2C.1 , PTEN p.G127R 2C.1 , SMARCA4 p.R370C 2C.2 , TP53 p.Y220C 2C.2 | 2C.1 | FGFR1 amp 2C.1 , MYCN loss 2C.2 , PIK3CB loss 2C.1 | 2C.1 |  | NL | 2C.1 |
| SP92364 | Uterus | OTHER | ARID1A p.Q766Sfs*67 2C.2 , ARID1A p.Y551Tfs*68 2C.2 , ATR p.R2431K 2C.2 , CCND1 p.C243R 2C.2 , CREBBP p.C1474Vfs*76 2C.2 , CREBBP p.K1495* 2.C2 , FANCI p.L985R 2C.1 , MSH6 p.F1088Lfs*5 1B , NBN p.R466Gfs*18 2C.1 , NOTCH1 p.V1576A 2C.2 , PIK3CA p.M1043I 2C.1 , POLE p.Y1889C 2C.1 , PTEN p.R233* 2C.1 , PTEN p.C136* 2C.1 , RAD50 p.K722Rfs*14 2C.1 , RB1 p.F755I 2C.2 , RB1 p.I124Rfs*6 2C.2 , SMARCA4 p.L1161Sfs*3 2C.2 , TP53 p.R273C 2C.2 , TP53 p.S90Pfs*33 2C.2 , TP53 p.R158C 2C.2 | 1B |  | NL |  | NL | 1B |
| SP92460 | Uterus | OTHER | ATR p.K2587N 2C.2 , MRE11A p.N511Ifs*13 2C.1 , PIK3R1 p.R642* 2C.2 , PTEN p.R130L 2C.1 , RNF43 p.G659Vfs*41 2C.2 | 2C.1 |  | NL |  | NL | 2C.1 |
| SP9251 | Breast | BREAST | PTEN p.I135K 2C.1 , TP53 p.Y220H 2C.2 | 2C.1 |  | NL |  | NL | 2C.1 |
| SP92659 | Uterus | OTHER | AR p.A253V 2C.2 , ARID1A p.R1989* 2C.2 , ATM p.L2866I 2C.1 , ATRX p.R781Q 2C.2 , CREBBP p.F1439L 2C.2 , ERBB4 p.R711C 2C.2 , KRAS p.G12V 2C.1 , MTOR p.F1888V 2C.1 , PIK3CA p.R108H 2C.1 , PIK3R1 p.R348* 2C.2 , PTEN p.L146* 2C.1 , PTEN p.R130G 2C.1 | 2C.1 |  | NL |  | NL | 2C.1 |
| SP92707 | Uterus | OTHER | PTEN p.T319Nfs*6 2C.1 , PTEN p.R130Q 2C.1 | 2C.1 |  | NL |  | NL | 2C.1 |
| SP92723 | Uterus | OTHER | ARID1A p.R1276* 2C.2 , NRAS p.Q61R 2C.1 , PIK3R1 p.T576delT 2C.2 , PIK3R1 p.S460Vfs*20 2C.2 , PTEN p.N329Kfs*14 2C.1 , PTEN p.T319*fs*1 2C.1 | 2C.1 |  | NL |  | NL | 2C.1 |
| SP92787 | Uterus | OTHER | PIK3CA p.G106_N107delGN 2C.1 , RNF43 p.P441Lfs*61 2C.2 , TP53 p.Y220C 2C.2 | 2C.1 |  | NL |  | NL | 2C.1 |
| SP92931 | Uterus | OTHER | CDKN2A p.Y44Lfs*76 2C.1 , FGFR2 p.S252W 2C.1 , PIK3R1 p.L581Ffs*21 2C.2 , PTEN p.C124W 2C.1 , PTEN p.C124F 2C.1 , PTEN . 2C.1 , TP53 p.C141Y 2C.2 , TP53 p.C135R 2C.2 | 2C.1 |  | NL |  | NL | 2C.1 |
| SP92947 | Uterus | OTHER | AKT1 p.E17K 2C.2 , CTNNB1 p.S33F 2C.2 | 2C.2 |  | NL |  | NL | 2C.2 |
| SP93652 | Uterus | OTHER | PIK3CA p.E81K 2C.1 , TP53 p.R273H 2C.2 | 2C.1 |  | NL |  | NL | 2C.1 |
| SP93772 | Uterus | OTHER | PIK3CA p.E542K 2C.1 , TP53 p.Q167Hfs*3 2C.2 | 2C.1 | MYCN loss 2C.2 | 2C.2 |  | NL | 2C.1 |
| SP94060 | Uterus | OTHER | FBXW7 p.R393* 2C.2 , PIK3CA p.Q546K 2C.1 , TP53 p.R248Q 2C.2 | 2C.1 |  | NL | FGR fusion 2C.2 | 2C.2 | 2C.1 |
| SP94236 | Uterus | OTHER | NOTCH2 p.P1157H 2C.2 , RAC1 p.P29S 3 , SETD2 p.E346* 2D , TP53 p.P278T 2C.2 , U2AF1 p.S34F 2D | 2C.2 | TERT loss 3 | 3 |  | NL | 2C.2 |
| SP9433 | Breast | BREAST | TP53 p.K132E 2C.2 | 2C.2 | ERBB2 amp 1A.1 | 1A.1 |  | NL | 1A.1 |
| SP94332 | Uterus | OTHER | TP53 p.H193R 2C.2 | 2C.2 |  | NL |  | NL | 2C.2 |
| SP94540 | Uterus | OTHER | KRAS p.G12A 2C.1 , PIK3CA p.R108H 2C.1 , PIK3R1 p.Y408* 2C.2 , TP53 p.H178D 2C.2 | 2C.1 |  | NL |  | NL | 2C.1 |
| SP94588 | Uterus | OTHER | FBXW7 p.G423V 2C.2 , PPP2R1A p.P179R 2C.1 , TP53 p.P128_L130delPAL 2C.2 | 2C.1 |  | NL |  | NL | 2C.1 |
| SP94661 | Uterus | OTHER | PIK3CA p.K111E 2C.1 , TP53 p.R248Q 2C.2 | 2C.1 | ERBB2 amp 2C.1 , ERBB2 amp 2C.1 | 2C.1 |  | NL | 2C.1 |
| SP9481 | Breast | BREAST |  | NL | ERBB2 amp 1A.1 | 1A.1 |  | NL | 1A.1 |
| SP94917 | Uterus | OTHER | ARID1A p.D1850Tfs*33 2C.2 , NF1 p.N78Ifs*7 2C.1 , SMARCA4 p.Q201* 2C.2 , TP53 p.V157_P177delVRAMAIYKQSQHMTEVVRRCP 2C.2 , TSC1 p.N891Tfs*40 2C.1 | 2C.1 |  | NL |  | NL | 2C.1 |
| SP94933 | Uterus | OTHER | ATR p.L1576R 2C.2 , ATRX p.R2271G 2C.2 , ERBB2 p.R678Q 2C.1 , NF1 p.N839D 2C.1 , PIK3CA p.E110delE 2C.1 , PTEN p.N323Kfs*2 2C.1 , PTEN p.D268Gfs*30 2C.1 , RAD50 p.N934Ifs*6 2C.1 | 2C.1 |  | NL |  | NL | 2C.1 |
| SP95126 | Uterus | OTHER | DDR2 p.T836M 2C.2 , GNAS p.G206A 2C.2 , PPP2R1A p.P179R 2C.1 , TP53 p.R273S 2C.2 | 2C.1 | BRAF loss 2C.1 , NTRK1 loss 2C.1 | 2C.1 |  | NL | 2C.1 |
| SP95222 | Uterus | OTHER | BRAF p.R462K 2C.1 , TP53 p.R273H 2C.2 | 2C.1 | CCNE1 amp 2C.2 , MYC amp 2C.2 | 2C.2 |  | NL | 2C.1 |
| SP953 | Bladder | OTHER | ERBB2 p.D769N 2C.1 , PIK3CA p.E453Q 2C.1 , POLE p.E1715K 2C.1 , TP53 p.C124Wfs*25 2C.2 | 2C.1 | CCND1 amp 2C.2 , ERBB2 amp 2C.1 , FGF19 amp 2D , FGF3 amp 2D , FGFR1 amp 2C.1 | 2C.1 |  | NL | 2C.1 |
| SP95406 | Uterus | OTHER | PIK3R1 . 2C.2 , PIK3R1 . 2C.2 , RB1 p.Q762* 2C.2 , TP53 p.N239D 2C.2 | 2C.2 | FGFR1 amp 2C.1 | 2C.1 |  | NL | 2C.1 |
| SP95550 | Uterus | OTHER | ERBB3 p.G284R 2C.2 , MTOR p.Y1974C 2C.1 , PIK3CA p.N345K 2C.1 , TP53 p.R175H 2C.2 | 2C.1 |  | NL |  | NL | 2C.1 |
| SP95646 | Uterus | OTHER |  | NL | AKT2 amp 2C.2 , CCNE1 amp 2C.2 , ERBB2 amp 2C.1 | 2C.1 |  | NL | 2C.1 |
| SP96110 | Colon/Rectum | COLON/RECTUM | FBXW7 p.R465H 2C.2 , NOTCH2 p.R2453Q 2C.2 , PIK3CA p.P104R 2C.1 | 2C.1 |  | NL |  | NL | 2C.1 |
| SP96112 | Colon/Rectum | COLON/RECTUM | KRAS p.G12C 1A.1 , TP53 p.R273H 2C.2 | 1A.1 |  | NL |  | NL | 1A.1 |
| SP96114 | Colon/Rectum | COLON/RECTUM | BRCA2 p.R2842C 2C.1 , KRAS p.G12V 1A.1 , PIK3CA p.M1043I 2C.1 , TP53 p.R175H 2C.2 | 1A.1 |  | NL |  | NL | 1A.1 |
| SP96116 | Colon/Rectum | COLON/RECTUM | KRAS p.G12D 1A.1 , TP53 p.V173L 2C.2 | 1A.1 |  | NL |  | NL | 1A.1 |
| SP96118 | Colon/Rectum | COLON/RECTUM | CTNNB1 p.S45P 2C.2 , FBXW7 p.R465C 2C.2 , PTPN11 p.G503V 2C.2 , SMAD4 p.R361H 2C.2 | 2C.2 |  | NL |  | NL | 2C.2 |
| SP96120 | Colon/Rectum | COLON/RECTUM | CREBBP p.R1103* 2D , FBXW7 p.T653M 2C.2 , KRAS p.G12C 1A.1 , TP53 p.C242Afs*5 2C.2 | 1A.1 | CDK6 loss 2C.1 , FLT3 amp 2C.1 , FLT3 amp 2C.1 | 2C.1 |  | NL | 1A.1 |
| SP96122 | Colon/Rectum | COLON/RECTUM | KRAS p.K117N 1A.1 , PTCH1 p.D860Y 2C.1 | 1A.1 |  | NL |  | NL | 1A.1 |
| SP96124 | Colon/Rectum | COLON/RECTUM | FBXW7 p.L660Qfs*47 2C.2 , PIK3CA p.H1047R 2C.1 , TP53 p.Y236H 2C.2 | 2C.1 |  | NL | RSPO3 fusion 3 | 3 | 2C.1 |
| SP96126 | Colon/Rectum | COLON/RECTUM | ERBB3 p.V104M 2C.2 , KRAS p.G12V 1A.1 , TP53 p.R273H 2C.2 | 1A.1 |  | NL |  | NL | 1A.1 |
| SP96129 | Colon/Rectum | COLON/RECTUM | ALK p.R1209Q 2C.1 , ATM p.D814N 2C.1 , TP53 p.Q38Kfs*6 2C.2 | 2C.1 |  | NL |  | NL | 2C.1 |
| SP96136 | Bladder | OTHER | ARID1A p.Q515* 2C.2 , CREBBP p.F1484S 2C.2 , ERCC2 p.T484M 2C.2 , RB1 p.T271Nfs*5 2C.2 , TP53 p.C277F 2C.2 | 2C.2 | EGFR loss 2C.1 , RICTOR loss 2C.2 , TERT loss 3 | 2C.1 |  | NL | 2C.1 |
| SP96147 | Breast | BREAST | TP53 p.L111P 2C.2 | 2C.2 | AKT2 amp 2C.2 , CCND3 loss 2C.2 , CCND3 loss 2C.2 , MYC amp 2C.2 | 2C.2 |  | NL | 2C.2 |
| SP96163 | Breast | BREAST | FBXW7 p.R465C 2C.2 , TP53 p.R156delR 2C.2 | 2C.2 |  | NL |  | NL | 2C.2 |
| SP963 | Bladder | OTHER | BAP1 p.I47M 2C.1 , ERBB2 p.L313V 2C.1 , SETD2 p.H1629Y 2D , TP53 p.A161T 2C.2 | 2C.1 | CCND1 amp 2C.2 , EGFR loss 2C.1 , ERBB2 amp 2C.1 , FGF19 amp 2D , FGF3 amp 2D | 2C.1 | ERBB2 fusion 2C.1 | 2C.1 | 2C.1 |
| SP9648 | Breast | BREAST | ATR p.K93Nfs*2 2C.2 , TP53 p.H178Pfs*2 2C.2 | 2C.2 |  | NL |  | NL | 2C.2 |
| SP96511 | Breast | BREAST | TP53 p.R342* 2C.2 | 2C.2 | MYC amp 2C.2 | 2C.2 |  | NL | 2C.2 |
| SP96540 | Cervix | OTHER |  | NL |  | NL |  | NL | NL |
| SP967 | Bladder | OTHER |  | NL |  | NL | FGFR3 fusion 2C.1 | 2C.1 | 2C.1 |
| SP97104 | Kidney | OTHER |  | NL |  | NL |  | NL | NL |
| SP97113 | Kidney | OTHER |  | NL |  | NL |  | NL | NL |
| SP97124 | Kidney | OTHER |  | NL |  | NL |  | NL | NL |
| SP97145 | Kidney | OTHER |  | NL |  | NL |  | NL | NL |
| SP97161 | Kidney | OTHER |  | NL |  | NL |  | NL | NL |
| SP97194 | Kidney | OTHER | ALK p.Q1336H 2C.1 , FANCI p.V42Lfs*6 2C.1 , SETD2 p.F721Ifs*5 2D | 2C.1 |  | NL |  | NL | 2C.1 |
| SP97243 | Kidney | OTHER |  | NL |  | NL |  | NL | NL |
| SP97249 | Kidney | OTHER | TP53 p.S241C 2C.2 | 2C.2 | PIK3CB loss 2C.1 | 2C.1 |  | NL | 2C.1 |
| SP97258 | Kidney | OTHER | ATR p.L502M 2C.2 , MSH6 p.E255Gfs*3 1B | 1B |  | NL |  | NL | 1B |
| SP97269 | Kidney | OTHER | MET p.V1088G 2C.1 , MET p.V1088M 2C.1 | 2C.1 |  | NL |  | NL | 2C.1 |
| SP97278 | Kidney | OTHER |  | NL |  | NL |  | NL | NL |
| SP975 | Bladder | OTHER | ERCC2 p.N238S 2C.2 , NBN . 2C.1 , SETD2 p.E1991* 2D | 2C.1 | CCNE1 amp 2C.2 , MDM2 amp 2C.2 , MDM2 amp 2C.2 | 2C.2 |  | NL | 2C.1 |
| SP98053 | Liver | OTHER | ARID1A p.E1542* 2C.2 | 2C.2 |  | NL |  | NL | 2C.2 |
| SP98060 | Liver | OTHER | NF2 p.Y144*fs*1 2C.1 , TSC2 p.C256* 2C.1 | 2C.1 |  | NL |  | NL | 2C.1 |
| SP98065 | Liver | OTHER | CTNNB1 p.S33C 2C.2 , TP53 p.G266V 2C.2 | 2C.2 |  | NL |  | NL | 2C.2 |
| SP98078 | Liver | OTHER |  | NL |  | NL |  | NL | NL |
| SP98082 | Liver | OTHER | AKT2 p.D324G 2C.2 | 2C.2 |  | NL |  | NL | 2C.2 |
| SP98090 | Liver | OTHER |  | NL | MDM4 loss 2D , NTRK1 loss 2C.1 | 2C.1 |  | NL | 2C.1 |
| SP98092 | Liver | OTHER | PIK3CA p.T1052K 2C.1 , PTEN p.P246R 2C.1 | 2C.1 |  | NL |  | NL | 2C.1 |
| SP98096 | Liver | OTHER |  | NL |  | NL |  | NL | NL |
| SP9816 | Breast | BREAST | TP53 p.R248Q 2C.2 | 2C.2 |  | NL |  | NL | 2C.2 |
| SP98164 | Liver | OTHER |  | NL |  | NL |  | NL | NL |
| SP98192 | Liver | OTHER | BAP1 p.W196G 2C.1 , IDH1 p.R132G 2C.1 | 2C.1 |  | NL |  | NL | 2C.1 |
| SP98265 | Liver | OTHER | TP53 p.Y103Tfs*20 2C.2 | 2C.2 | AKT1 amp 2C.2 | 2C.2 |  | NL | 2C.2 |
| SP98289 | Liver | OTHER | TP53 p.Y220C 2C.2 | 2C.2 |  | NL |  | NL | 2C.2 |
| SP98297 | Liver | OTHER | CTNNB1 p.D32G 2C.2 | 2C.2 |  | NL |  | NL | 2C.2 |
| SP98305 | Liver | OTHER | TP53 p.C135* 2C.2 | 2C.2 |  | NL |  | NL | 2C.2 |
| SP98313 | Liver | OTHER |  | NL | CCND1 amp 2C.2 , FGF19 amp 2D , FGF3 amp 2D | 2C.2 |  | NL | 2C.2 |
| SP98327 | Liver | OTHER | MAP2K1 p.Y130C 2C.1 , TP53 p.R306* 2C.2 , TSC1 p.T356Pfs*84 2C.1 | 2C.1 |  | NL |  | NL | 2C.1 |
| SP98359 | Liver | OTHER |  | NL | CCND3 loss 2C.2 | 2C.2 |  | NL | 2C.2 |
| SP98896 | Liver | OTHER | ARID1A p.S634Dfs*12 2C.2 , TP53 p.H214R 2C.2 | 2C.2 |  | NL |  | NL | 2C.2 |
| SP98898 | Liver | OTHER | TSC1 p.R420Kfs*22 2C.1 | 2C.1 |  | NL |  | NL | 2C.1 |
| SP98900 | Liver | OTHER | TP53 p.V274A 2C.2 | 2C.2 | MYC amp 2C.2 | 2C.2 |  | NL | 2C.2 |
| SP98902 | Liver | OTHER | CTNNB1 p.S45P 2C.2 , NFE2L2 p.E82A 3 | 2C.2 |  | NL |  | NL | 2C.2 |
| SP98913 | Liver | OTHER | CTNNB1 p.G34R 2C.2 | 2C.2 |  | NL |  | NL | 2C.2 |
| SP98915 | Liver | OTHER |  | NL |  | NL |  | NL | NL |
| SP98921 | Liver | OTHER |  | NL |  | NL |  | NL | NL |
| SP98925 | Liver | OTHER |  | NL | MYC amp 2C.2 | 2C.2 |  | NL | 2C.2 |
| SP98941 | Liver | OTHER |  | NL |  | NL |  | NL | NL |
| SP98945 | Liver | OTHER |  | NL |  | NL |  | NL | NL |
| SP98955 | Liver | OTHER | NF1 p.Y1292C 2C.1 | 2C.1 |  | NL |  | NL | 2C.1 |
| SP98965 | Liver | OTHER | NFE2L2 p.G81C 3 , NOTCH2 p.D1004A 2C.2 , TP53 p.S215R 2C.2 | 2C.2 | AKT3 loss 2C.2 , MDM4 loss 2D , NTRK1 loss 2C.1 | 2C.1 |  | NL | 2C.1 |
| SP98967 | Liver | OTHER | ATM p.Q2442P 2C.1 | 2C.1 |  | NL |  | NL | 2C.1 |
| SP98981 | Liver | OTHER | TP53 p.Q317Sfs*28 2C.2 | 2C.2 |  | NL |  | NL | 2C.2 |
| SP98985 | Liver | OTHER | CTNNB1 p.S33C 2C.2 | 2C.2 |  | NL |  | NL | 2C.2 |
| SP98991 | Liver | OTHER |  | NL |  | NL |  | NL | NL |
| SP99001 | Liver | OTHER |  | NL |  | NL |  | NL | NL |
| SP99007 | Liver | OTHER | TP53 p.Y234*fs*1 2C.2 | 2C.2 |  | NL |  | NL | 2C.2 |
| SP99011 | Liver | OTHER | TP53 p.R280G 2C.2 | 2C.2 |  | NL |  | NL | 2C.2 |
| SP99041 | Liver | OTHER |  | NL |  | NL |  | NL | NL |
| SP99045 | Liver | OTHER | CTNNB1 p.S45P 2C.2 , TP53 p.H178Pfs*3 2C.2 | 2C.2 | MYC amp 2C.2 | 2C.2 |  | NL | 2C.2 |
| SP99049 | Liver | OTHER | SETD2 p.R1312* 2D | 2D |  | NL |  | NL | 2D |
| SP99053 | Liver | OTHER |  | NL | MYC amp 2C.2 | 2C.2 |  | NL | 2C.2 |
| SP99057 | Liver | OTHER |  | NL |  | NL |  | NL | NL |
| SP99061 | Liver | OTHER | FLT3 p.R815S 2C.1 , SETD2 p.E2120* 2D | 2C.1 | MYC amp 2C.2 | 2C.2 |  | NL | 2C.1 |
| SP99065 | Liver | OTHER | CTNNB1 p.S45F 2C.2 | 2C.2 |  | NL |  | NL | 2C.2 |
| SP99069 | Liver | OTHER | TP53 p.L130F 2C.2 | 2C.2 |  | NL |  | NL | 2C.2 |
| SP99077 | Liver | OTHER | CTNNB1 p.T41A 2C.2 , PIK3CA p.H1047R 2C.1 | 2C.1 |  | NL | TERT fusion 2C.2 | 2C.2 | 2C.1 |
| SP99093 | Liver | OTHER | TP53 p.Y163C 2C.2 | 2C.2 |  | NL |  | NL | 2C.2 |
| SP99101 | Liver | OTHER |  | NL |  | NL |  | NL | NL |
| SP99113 | Biliary | OTHER | NOTCH1 p.L1853P 2C.2 | 2C.2 |  | NL |  | NL | 2C.2 |
| SP99117 | Liver | OTHER | ATRX p.S783Ffs*8 2C.2 , TP53 p.R174K 2C.2 , TP53 p.R174S 2C.2 | 2C.2 |  | NL |  | NL | 2C.2 |
| SP99129 | Liver | OTHER | BAP1 p.W196G 2C.1 , BAP1 p.R264Kfs*20 2C.1 | 2C.1 | AKT3 loss 2C.2 | 2C.2 |  | NL | 2C.1 |
| SP99133 | Liver | OTHER |  | NL | CCND1 amp 2C.2 , FGF19 amp 2D | 2C.2 |  | NL | 2C.2 |
| SP99149 | Liver | OTHER |  | NL |  | NL |  | NL | NL |
| SP99157 | Liver | OTHER |  | NL |  | NL |  | NL | NL |
| SP99161 | Liver | OTHER | TP53 p.D259Y 2C.2 | 2C.2 |  | NL |  | NL | 2C.2 |
| SP99165 | Liver | OTHER | ALK p.S1308P 2C.1 , TP53 p.I254T 2C.2 | 2C.1 |  | NL |  | NL | 2C.1 |
| SP99169 | Liver | OTHER |  | NL |  | NL |  | NL | NL |
| SP99173 | Liver | OTHER | RB1 p.A488E 2C.2 | 2C.2 |  | NL |  | NL | 2C.2 |
| SP99177 | Liver | OTHER | NOTCH2 p.C403Y 2C.2 | 2C.2 |  | NL |  | NL | 2C.2 |
| SP99181 | Liver | OTHER |  | NL |  | NL |  | NL | NL |
| SP99185 | Biliary | OTHER |  | NL |  | NL |  | NL | NL |
| SP99189 | Liver | OTHER |  | NL |  | NL |  | NL | NL |
| SP99197 | Liver | OTHER |  | NL | MYCN loss 2C.2 | 2C.2 |  | NL | 2C.2 |
| SP99201 | Liver | OTHER |  | NL |  | NL |  | NL | NL |
| SP99209 | Biliary | OTHER |  | NL |  | NL |  | NL | NL |
| SP99213 | Biliary | OTHER | PIK3CA p.E453K 2C.1 | 2C.1 |  | NL |  | NL | 2C.1 |
| SP99217 | Liver | OTHER | NTRK2 p.T748M 2C.1 | 2C.1 |  | NL |  | NL | 2C.1 |
| SP99221 | Biliary | OTHER |  | NL |  | NL | RAD51B fusion 2C.1 | 2C.1 | 2C.1 |
| SP99225 | Biliary | OTHER | KRAS p.Q61H 2C.1 | 2C.1 |  | NL |  | NL | 2C.1 |
| SP99229 | Liver | OTHER | RB1 p.I679Tfs*17 2C.2 | 2C.2 |  | NL |  | NL | 2C.2 |
| SP99241 | Biliary | OTHER | CDKN1B p.W76* 2D , FGFR2 p.Y376C 2C.1 | 2C.1 |  | NL | FGFR2 fusion 1A.1 | 1A.1 | 1A.1 |
| SP99249 | Liver | OTHER |  | NL |  | NL |  | NL | NL |
| SP99279 | Liver | OTHER | TP53 p.A63Cfs*57 2C.2 | 2C.2 |  | NL |  | NL | 2C.2 |
| SP99287 | Biliary | OTHER |  | NL |  | NL |  | NL | NL |
| SP99293 | Biliary | OTHER |  | NL | CDK2 amp 2D , MYC amp 2C.2 , PIK3CA loss 2C.1 | 2C.1 |  | NL | 2C.1 |
| SP99297 | Biliary | OTHER |  | NL |  | NL |  | NL | NL |
| SP9930 | Breast | BREAST | TP53 p.Y163C 2C.2 | 2C.2 |  | NL |  | NL | 2C.2 |
| SP99301 | Biliary | OTHER |  | NL |  | NL |  | NL | NL |
| SP99305 | Biliary | OTHER | ARID1A p.A1155Qfs*6 2C.2 | 2C.2 |  | NL |  | NL | 2C.2 |
| SP99321 | Biliary | OTHER | KRAS p.G12V 2C.1 , SF3B1 p.K700E 3 | 2C.1 |  | NL |  | NL | 2C.1 |
| SP99325 | Biliary | OTHER | ARID1A p.Q449Afs*173 2C.2 , ATRX p.R1302Efs*44 2C.2 , BRAF p.L537M 2C.1 , EGFR p.S720F 2C.1 , FBXW7 p.T610Qfs*18 2C.2 , MSH2 p.C199R 1B , MSH6 p.F1088Sfs*2 1B , NF1 p.S2687Cfs*5 2C.1 , NF1 p.R1769Q 2C.1 , NF1 p.Y628Tfs*3 2C.1 , NF2 p.A4Pfs*6 2C.1 , RNF43 p.C119Afs*39 2C.2 , TP53 p.R273C 2C.2 , TSC1 p.P603Rfs*26 2C.1 | 1B |  | NL |  | NL | 1B |
| SP99329 | Biliary | OTHER | BRAF p.D594N 2C.1 , SMAD4 p.Q366* 2C.2 | 2C.1 |  | NL |  | NL | 2C.1 |
| SP99333 | Biliary | OTHER | BAP1 p.A206Vfs*18 2C.1 , PIK3CA p.E542K 2C.1 | 2C.1 |  | NL |  | NL | 2C.1 |
| SP99337 | Biliary | OTHER | IDH1 p.R132C 2C.1 , NF2 p.L542H 2C.1 | 2C.1 |  | NL |  | NL | 2C.1 |
| SP99341 | Biliary | OTHER |  | NL | AR amp 2C.2 , FGFR1 amp 2C.1 , MYC amp 2C.2 | 2C.1 | MET fusion 2C.1 | 2C.1 | 2C.1 |
| SP9979 | Breast | BREAST |  | NL |  | NL |  | NL | NL |

Additional file 11: Table S7 Simulation results of the alterations that would have been identified if the gene set of the 514 gene NGS panel was used in the PCAWG samples.

| **sample** | **Tumor_type1** | **Tumor_type2** | **Mutation, LoE** | **LoE MAX MUT** | **GENE CNV LoE** | **LoE MAX CNV** | **fusion LoE** | **LoE max FUS** | **MAX LoE** |
| --- | --- | --- | --- | --- | --- | --- | --- | --- | --- |
| SP1003 | Bladder | OTHER | ARID1A p.Q1095* 2C.2 , ELF3 p.S133*fs*1 3 , MLH1 p.E754Q 1B , POT1 p.S119L 3 , TP53 p.R248W 2C.2 | 1B |  | NL |  | NL | 1B |
| SP10084 | Breast | BREAST | TP53 2C.2 | 2C.2 | CCND1 amp 2C.2 , FGF19 amp 2D , FGF3 amp 2D , FGF4 amp 2D , FGFR1 amp 2C.1 , GNAS amp 2C.2 , MCL1 amp 2D , MYC amp 2C.2 , RIT1 amp 3 , RPTOR amp 3 , SPOP amp 3 | 2C.1 |  | NL | 2C.1 |
| SP1009 | Bladder | OTHER | ARID1A 2C.2 , MLL2 p.S1632* 3 , MLL2 p.E3562* 3 , MLL3 p.A2858Lfs*6 3 , NFE2L2 p.G31A 3 , SF3B1 p.R625C 3 | 2C.2 | CDKN2A loss 2C.1 , CDKN2B loss 2C.2 , MTAP loss 3 | 2C.1 |  | NL | 2C.1 |
| SP10150 | Breast | BREAST | CDKN1B 2D , DICER1 p.S1747L 3 , PIK3CA p.H1047R 1A.1 , TP53 p.C238Y 2C.2 | 1A.1 | FAM135B amp 3 , MYC amp 2C.2 , PXDNL amp 3 | 2C.2 |  | NL | 1A.1 |
| SP101515 | Ovary | OVARY | TP53 2C.2 | 2C.2 |  | NL |  | NL | 2C.2 |
| SP101519 | Ovary | OVARY | TP53 2C.2 | 2C.2 | CCNE1 amp 2C.2 , CCNE1 amp 2C.2 | 2C.2 |  | NL | 2C.2 |
| SP101521 | Ovary | OVARY | TP53 2C.2 | 2C.2 | ARAF amp 2C.1 , CCNE1 amp 2C.2 , CTNND2 amp 3 , CTNND2 amp 3 , MECOM amp 3 , MPL amp 3 , PRKACA amp 3 | 2C.1 |  | NL | 2C.1 |
| SP101523 | Ovary | OVARY | EPHA2 2C.2 , MLL3 p.S2751L 3 , TP53 p.A138P 2C.2 | 2C.2 |  | NL |  | NL | 2C.2 |
| SP101526 | Ovary | OVARY | TP53 2C.2 | 2C.2 | AKT2 amp 2C.2 , AKT3 amp 2C.2 , AXL amp 2C.2 , CCNE1 amp 2C.2 , MAPK1 amp 2C.2 , MPL amp 3 , PIM1 amp 3 | 2C.2 |  | NL | 2C.2 |
| SP101528 | Ovary | OVARY | RBM10 3 , TP53 p.R282W 2C.2 | 2C.2 | CCND2 amp 2C.2 , FGF23 amp 3 | 2C.2 |  | NL | 2C.2 |
| SP101532 | Ovary | OVARY | TP53 2C.2 | 2C.2 |  | NL |  | NL | 2C.2 |
| SP101536 | Ovary | OVARY | TP53 2C.2 | 2C.2 |  | NL |  | NL | 2C.2 |
| SP101540 | Ovary | OVARY | TP53 2C.2 | 2C.2 | AKT2 amp 2C.2 , CCNE1 amp 2C.2 , FAM135B amp 3 , IKBKB amp 3 , MYC amp 2C.2 | 2C.2 |  | NL | 2C.2 |
| SP101544 | Ovary | OVARY | TP53 2C.2 | 2C.2 | CCNE1 amp 2C.2 , KRAS amp 2C.1 , PRKACA amp 3 | 2C.1 | ESR1 fusion 2C.1 | 2C.1 | 2C.1 |
| SP101548 | Ovary | OVARY | BRCA2 1A.1 , RNF43 p.L311Sfs*108 2C.2 | 1A.1 | NF1 loss 2C.1 | 2C.1 |  | NL | 1A.1 |
| SP101552 | Ovary | OVARY | TP53 2C.2 | 2C.2 |  | NL |  | NL | 2C.2 |
| SP101558 | Ovary | OVARY | TP53 2C.2 | 2C.2 |  | NL |  | NL | 2C.2 |
| SP101564 | Ovary | OVARY | RB1 2C.2 , TP53 p.R249G 2C.2 | 2C.2 | MECOM amp 3 | 3 |  | NL | 2C.2 |
| SP101572 | Ovary | OVARY | TP53 2C.2 | 2C.2 | AKT2 amp 2C.2 , BCL6 amp 3 , CCNE1 amp 2C.2 , ERBB2 amp 2C.1 , GATA2 amp 2C.2 , MECOM amp 3 , MEF2B amp 3 , MYC amp 2C.2 , PIK3CA amp 2C.1 , PIK3CB amp 2C.1 , PIK3R2 amp 2C.2 , RHEB amp 3 , ZNF429 amp 3 | 2C.1 |  | NL | 2C.1 |
| SP101576 | Ovary | OVARY | TP53 2C.2 | 2C.2 | BCL2L12 amp 3 , CCNE1 amp 2C.2 , GATA2 amp 2C.2 , MECOM amp 3 , MPL amp 3 , PIK3CB amp 2C.1 , RIT1 amp 3 , TPMT amp 3 | 2C.1 |  | NL | 2C.1 |
| SP101580 | Ovary | OVARY | BRCA1 1A.1 , NF1 p.Q1174* 2C.1 , TP53 p.R248Q 2C.2 | 1A.1 | AKT3 amp 2C.2 , FAM135B amp 3 , H3F3A amp 2C.2 , MYC amp 2C.2 | 2C.2 |  | NL | 1A.1 |
| SP101584 | Ovary | OVARY | TP53 2C.2 | 2C.2 | CCND2 amp 2C.2 , FGF23 amp 3 , MEF2B amp 3 , PIK3R2 amp 2C.2 | 2C.2 |  | NL | 2C.2 |
| SP101588 | Ovary | OVARY | NCOR1 3 , TP53 p.R273L 2C.2 | 2C.2 | AKT2 amp 2C.2 | 2C.2 |  | NL | 2C.2 |
| SP101592 | Ovary | OVARY | TP53 2C.2 | 2C.2 | AKT2 amp 2C.2 , CCND2 amp 2C.2 , CHD4 amp 3 , FAM135B amp 3 , FGF23 amp 3 , KRAS amp 2C.1 , MYC amp 2C.2 , PXDNL amp 3 , SLCO1B3 amp 3 | 2C.1 |  | NL | 2C.1 |
| SP101596 | Ovary | OVARY | TP53 2C.2 | 2C.2 | AKT2 amp 2C.2 , CTNND2 amp 3 , IL7R amp 3 , RICTOR amp 2C.2 , TERT amp 3 | 2C.2 |  | NL | 2C.2 |
| SP101600 | Ovary | OVARY |  | NL |  | NL |  | NL | NL |
| SP101604 | Ovary | OVARY | ARID1B 2D , ASXL1 p.D1004E 2C.2 , BRCA1 p.V1176Ffs*34 1A.1 , TP53 p.E286K 2C.2 | 1A.1 |  | NL |  | NL | 1A.1 |
| SP101610 | Ovary | OVARY | TP53 2C.2 | 2C.2 |  | NL |  | NL | 2C.2 |
| SP101616 | Ovary | OVARY | ACVR1 3 , SMARCB1 p.R201Q 2D , TP53 p.L130H 2C.2 | 2C.2 |  | NL |  | NL | 2C.2 |
| SP101622 | Ovary | OVARY | ASXL1 2C.2 , TP53 p.W146* 2C.2 | 2C.2 | FAM135B amp 3 , PIK3CA amp 2C.1 | 2C.1 |  | NL | 2C.1 |
| SP101628 | Ovary | OVARY | TP53 2C.2 | 2C.2 | GATA2 amp 2C.2 , MTOR amp 2C.1 , PIK3CB amp 2C.1 , PIK3R2 amp 2C.2 , SETBP1 amp 3 | 2C.1 |  | NL | 2C.1 |
| SP101634 | Ovary | OVARY | TP53 2C.2 | 2C.2 |  | NL |  | NL | 2C.2 |
| SP101642 | Ovary | OVARY | AXIN2 2D , TP53 p.R342* 2C.2 | 2C.2 |  | NL |  | NL | 2C.2 |
| SP101648 | Ovary | OVARY | CASP8 2D , FAT1 p.V1117L 3 , NF1 p.L2279* 2C.1 , TP53 p.S241F 2C.2 , ZFHX3 p.R2227Qfs*10 3 | 2C.1 | MYC amp 2C.2 | 2C.2 |  | NL | 2C.1 |
| SP101654 | Ovary | OVARY | RASA1 3 , TP53 p.C124* 2C.2 | 2C.2 | CTNND2 amp 3 , IL7R amp 3 , RICTOR amp 2C.2 , TERT amp 3 | 2C.2 | ESR1 fusion 2C.1 | 2C.1 | 2C.1 |
| SP101658 | Ovary | OVARY | TP53 2C.2 | 2C.2 |  | NL |  | NL | 2C.2 |
| SP101662 | Ovary | OVARY | ARHGAP35 3 , TP53 p.Y205Wfs*41 2C.2 | 2C.2 |  | NL |  | NL | 2C.2 |
| SP101666 | Ovary | OVARY | TP53 2C.2 | 2C.2 |  | NL |  | NL | 2C.2 |
| SP101670 | Ovary | OVARY | TP53 2C.2 | 2C.2 |  | NL |  | NL | 2C.2 |
| SP101674 | Ovary | OVARY | TP53 2C.2 | 2C.2 | MYC amp 2C.2 | 2C.2 |  | NL | 2C.2 |
| SP101678 | Ovary | OVARY | TP53 2C.2 | 2C.2 | MYC amp 2C.2 , TPMT amp 3 | 2C.2 |  | NL | 2C.2 |
| SP101686 | Ovary | OVARY | TP53 2C.2 | 2C.2 | AKT2 amp 2C.2 , CCNE1 amp 2C.2 | 2C.2 | AKT2 fusion 3 | 3 | 2C.2 |
| SP101690 | Ovary | OVARY | TP53 2C.2 | 2C.2 | CCNE1 amp 2C.2 , EGFR amp 2C.1 , GLI3 amp 3 , MECOM amp 3 , PIK3CA amp 2C.1 | 2C.1 |  | NL | 2C.1 |
| SP101694 | Ovary | OVARY | EPHA2 2C.2 , TP53 p.K132R 2C.2 | 2C.2 | ABL2 amp 3 , DDR2 amp 2C.2 , H3F3A amp 2C.2 , MCL1 amp 2D , MDM4 amp 2D , NTRK1 amp 2C.1 , PIK3C2B amp 3 , RIT1 amp 3 | 2C.1 |  | NL | 2C.1 |
| SP101700 | Ovary | OVARY | TP53 2C.2 | 2C.2 |  | NL |  | NL | 2C.2 |
| SP101708 | Ovary | OVARY | PALB2 2C.1 , TP53 p.R175H 2C.2 | 2C.1 | CCNE1 amp 2C.2 , PTEN loss 2C.1 | 2C.1 | NF1 fusion 2C.1 | 2C.1 | 2C.1 |
| SP101716 | Ovary | OVARY | FBXW7 2C.2 , NOTCH1 p.D545V 2C.2 , TP53 p.Y220C 2C.2 | 2C.2 | B2M loss 2C.2 , CCNE1 amp 2C.2 , NF1 loss 2C.1 , NF1 loss 2C.1 | 2C.1 |  | NL | 2C.1 |
| SP101724 | Ovary | OVARY | CDK12 2C.1 , RBM10 p.R793* 3 , TP53 p.E294* 2C.2 | 2C.1 | CCNE1 amp 2C.2 , CTNND2 amp 3 , CTNND2 amp 3 , CTNND2 amp 3 , ESR1 amp 2C.2 , IL7R amp 3 , TERT amp 3 | 2C.2 |  | NL | 2C.1 |
| SP101732 | Ovary | OVARY | RB1 2C.2 , TP53 p.R175H 2C.2 | 2C.2 | AKT1 amp 2C.2 | 2C.2 |  | NL | 2C.2 |
| SP101740 | Ovary | OVARY | TP53 2C.2 | 2C.2 |  | NL |  | NL | 2C.2 |
| SP101795 | Ovary | OVARY | TP53 2C.2 | 2C.2 | PTEN loss 2C.1 , RIT1 amp 3 , RIT1 amp 3 | 2C.1 |  | NL | 2C.1 |
| SP101881 | Ovary | OVARY | AR 2C.2 , TP53 p.S240Mfs*25 2C.2 | 2C.2 |  | NL |  | NL | 2C.2 |
| SP101891 | Ovary | OVARY | ATRX 2C.2 , DDX3X p.R475C 3 , TP53 p.R248Q 2C.2 | 2C.2 | FAM135B amp 3 , MYC amp 2C.2 | 2C.2 |  | NL | 2C.2 |
| SP101921 | Ovary | OVARY | NF2 2C.1 | 2C.1 | RB1 loss 2C.2 | 2C.2 |  | NL | 2C.1 |
| SP101931 | Ovary | OVARY | DPYD 3 , TP53 p.C176G 2C.2 | 2C.2 | ESR1 amp 2C.2 , ESR1 amp 2C.2 , KRAS amp 2C.1 , MPL amp 3 , PXDNL amp 3 | 2C.1 |  | NL | 2C.1 |
| SP102015 | Ovary | OVARY | TP53 2C.2 | 2C.2 | FAM135B amp 3 , FGFR3 amp 2C.1 , MYC amp 2C.2 | 2C.1 |  | NL | 2C.1 |
| SP102035 | Ovary | OVARY | TP53 2C.2 | 2C.2 |  | NL |  | NL | 2C.2 |
| SP102045 | Ovary | OVARY | ALK 2C.1 , BRCA2 p.M1I 1A.1 , FLT3 p.D698Y 2C.1 , FOXA1 p.S242C 3 | 1A.1 | BCL6 amp 3 , IDH2 amp 2C.1 , MECOM amp 3 , NTRK3 amp 2C.1 , PIK3CA amp 2C.1 | 2C.1 |  | NL | 1A.1 |
| SP102055 | Ovary | OVARY | TP53 2C.2 | 2C.2 |  | NL |  | NL | 2C.2 |
| SP102064 | Ovary | OVARY | IDH2 2C.1 , TP53 p.Y220C 2C.2 | 2C.1 | CDKN2A loss 2C.1 , CDKN2B loss 2D , MTAP loss 3 | 2C.1 |  | NL | 2C.1 |
| SP102074 | Ovary | OVARY | PDGFRA 2C.1 , TP53 p.F113V 2C.2 , TP53 p.F113C 2C.2 | 2C.1 | MAP2K4 loss 2C.1 | 2C.1 |  | NL | 2C.1 |
| SP102084 | Ovary | OVARY | MUTYH 2C.2 , TP53 p.C135F 2C.2 | 2C.2 |  | NL |  | NL | 2C.2 |
| SP102090 | Ovary | OVARY | CDK12 2C.1 , TP53 p.R273H 2C.2 | 2C.1 |  | NL |  | NL | 2C.1 |
| SP102096 | Ovary | OVARY | JAK3 2D , TP53 p.P85Lfs*38 2C.2 | 2C.2 | CCNE1 amp 2C.2 , MYC amp 2C.2 | 2C.2 |  | NL | 2C.2 |
| SP102103 | Ovary | OVARY | TP53 2C.2 | 2C.2 | CCNE1 amp 2C.2 | 2C.2 |  | NL | 2C.2 |
| SP102113 | Ovary | OVARY | KRAS 2C.1 , MLL2 p.F1367L 3 , TP53 p.Q192* 2C.2 | 2C.1 |  | NL |  | NL | 2C.1 |
| SP102123 | Ovary | OVARY | AKT1 2C.2 , TP53 p.E339Rfs*6 2C.2 | 2C.2 | MECOM amp 3 , PIK3CA amp 2C.1 | 2C.1 |  | NL | 2C.1 |
| SP102133 | Ovary | OVARY | ARID1A 2C.2 , ASXL1 p.G646Wfs*12 2C.2 , CDK12 p.D494N 2C.1 , CHD4 p.R1105Q 3 , FAM175A p.N303Kfs*2 3 , STAG2 p.L360H 3 , SUFU p.A25Gfs*23 2D , TP53 p.H168P 2C.2 | 2C.1 | CCNE1 amp 2C.2 | 2C.2 |  | NL | 2C.1 |
| SP102143 | Ovary | OVARY | FANCE 2C.1 , TP53 p.R280Kfs*26 2C.2 | 2C.1 |  | NL |  | NL | 2C.1 |
| SP102161 | Ovary | OVARY | RB1 2C.2 , TP53 p.I195T 2C.2 | 2C.2 | SPOP amp 3 | 3 |  | NL | 2C.2 |
| SP102168 | Ovary | OVARY | CDK12 2C.1 , TP53 p.I255F 2C.2 | 2C.1 | MECOM amp 3 | 3 |  | NL | 2C.1 |
| SP102174 | Ovary | OVARY | INPP4B 3 , TP53 p.H193R 2C.2 | 2C.2 | AR amp 2C.2 , CCND3 amp 2C.2 , DDR1 amp 3 , ERBB2 amp 2C.1 , ESR1 amp 2C.2 , ESR1 amp 2C.2 , FAM135B amp 3 , FGFR3 amp 2C.1 , MAPK1 amp 2C.2 , MYC amp 2C.2 , PIK3CA amp 2C.1 , PIK3CB amp 2C.1 , RIT1 amp 3 , RPTOR amp 3 | 2C.1 | ESR1 fusion 2C.1 | 2C.1 | 2C.1 |
| SP102187 | Ovary | OVARY | BRCA1 1A.1 , PALB2 p.S518* 2C.1 , TP53 p.M237I 2C.2 | 1A.1 |  | NL |  | NL | 1A.1 |
| SP102690 | Prostate | PROSTATE | MLL3 3 | 3 | CBFB loss 3 | 3 |  | NL | 3 |
| SP103866 | Skin | SKIN | IDH1 2C.1 , KDR p.G1063E 2D , KRAS p.Q61R 2C.1 , KRAS p.E62K 2C.1 , MSH3 p.S782F 2C.2 , NOTCH2 p.P2219S 2C.2 , PMS1 p.P241S 2C.2 , RB1 p.Q257* 2C.2 | 2C.1 |  | NL |  | NL | 2C.1 |
| SP103894 | Skin | SKIN | ARHGAP35 3 , BRIP1 p.K1040Dfs*4 2C.1 , NRAS p.Q61K 2C.1 | 2C.1 | MDM2 amp 2C.2 | 2C.2 |  | NL | 2C.1 |
| SP104056 | Skin | SKIN | APC 2C.2 , ERBB4 p.P800L 2C.2 , MLL p.S2228F 3 , NF1 p.R1870Q 2C.1 , RASA2 p.G780* 3 , RB1 p.W195* 2C.2 , SETBP1 p.L1197F 3 , SETD2 p.Q757* 2D , SMAD2 p.S397F 3 , TP53 p.R213Q 2C.2 | 2C.1 |  | NL |  | NL | 2C.1 |
| SP104530 | Skin | SKIN | BRAF 1A.1 , CDK4 p.R24S 2C.1 , DPYD p.P789S 3 , DPYD p.D965N 3 , JAK2 p.E577Sfs*8 2D , MGA p.R2396* 3 , NTRK1 p.Q660L 2C.1 , PIK3CA p.E545K 2C.1 , TAF1 p.P856S 3 | 1A.1 | BRAF amp 2C.1 , RHEB amp 3 , SMO amp 2C.1 | 2C.1 |  | NL | 1A.1 |
| SP10470 | Breast | BREAST | ACVR2A 3 , PIK3CA p.R88Q 1A.1 , TP53 p.G108Vfs*15 2C.2 | 1A.1 | ESR1 amp 2C.2 , PIK3CA amp 2C.1 , RPTOR amp 3 | 2C.1 |  | NL | 1A.1 |
| SP104984 | Stomach | OTHER | TP53 2C.2 | 2C.2 |  | NL |  | NL | 2C.2 |
| SP105006 | Stomach | OTHER | ARID1A 2C.2 , CTNNB1 p.G34R 2C.2 | 2C.2 | ERBB2 amp 1A.1 , ERBB2 amp 1A.1 , RARA amp 3 | 1A.1 |  | NL | 1A.1 |
| SP105018 | Stomach | OTHER | BRCA2 2C.1 , GNA13 p.V240A 3 , NOTCH2 p.D1733H 2C.2 , PMS1 p.R883H 2C.2 , SMAD4 p.G352A 2C.2 , TCF7L2 p.S177Y 3 | 2C.1 | AURKA amp 3 , PIK3R1 loss 2C.2 , ZNF217 amp 3 | 2C.2 |  | NL | 2C.1 |
| SP105086 | Stomach | OTHER | ABCB1 3 , BCL6 p.S506F 2C.2 , CREBBP p.G1542S 2C.2 , CTNNB1 p.T41I 2C.2 , ERBB2 p.D769Y 2C.1 , RNF43 p.R371* 2C.2 | 2C.1 | FAT1 loss 3 , GPS2 loss 3 , INPP4B loss 3 , MAP2K4 loss 2C.1 , NCOR1 loss 3 , RPA1 loss 2C.2 , TP53 loss 2C.2 | 2C.1 |  | NL | 2C.1 |
| SP105159 | Stomach | OTHER | ERBB2 2C.1 , ERBB3 p.K314T 2C.2 , FGFR4 p.K645E 2C.1 , JAK3 p.R403H 2C.2 , MLL2 p.R4484* 3 , PIK3CB p.E1047K 2C.1 , PIK3CB p.A1048V 2C.1 , PTEN p.D24E 2C.1 | 2C.1 |  | NL |  | NL | 2C.1 |
| SP105213 | Stomach | OTHER | ARID1A 2C.2 , JAK2 p.R683T 2D , PIK3CA p.N345K 2C.1 , PIK3CA p.E365K 2C.1 | 2C.1 |  | NL |  | NL | 2C.1 |
| SP105253 | Stomach | OTHER | TP53 2C.2 | 2C.2 |  | NL |  | NL | 2C.2 |
| SP105261 | Stomach | OTHER | TP53 2C.2 | 2C.2 | GNAS amp 2C.2 | 2C.2 |  | NL | 2C.2 |
| SP105577 | Stomach | OTHER | ARID1A 2C.2 , PIK3CA p.G118D 2C.1 | 2C.1 |  | NL |  | NL | 2C.1 |
| SP10563 | Breast | BREAST | PIK3CA 1A.1 | 1A.1 | ERBB2 amp 1A.1 , FAM135B amp 3 , FAM135B amp 3 , FAM135B amp 3 , FAM135B amp 3 , H3F3A amp 2C.2 , MDM4 amp 2D , MYC amp 2C.2 , PIK3C2B amp 3 , RARA amp 3 , RARA amp 3 | 1A.1 |  | NL | 1A.1 |
| SP105673 | Stomach | OTHER | B2M 2C.2 , INPP4B p.K819M 3 , PTEN p.Y346*fs*1 2C.1 | 2C.1 |  | NL |  | NL | 2C.1 |
| SP105708 | Thyroid | OTHER |  | NL |  | NL |  | NL | NL |
| SP105759 | Thyroid | OTHER |  | NL |  | NL |  | NL | NL |
| SP105807 | Thyroid | OTHER |  | NL |  | NL |  | NL | NL |
| SP1059 | Bladder | OTHER | ARHGAP35 3 , ATR p.A1291V 2C.2 , EPHA2 p.E640* 2C.2 , FGFR3 p.S249C 1A.1 , MLL2 p.E415* 3 , MLL2 p.G3819Pfs*189 3 , MLL3 p.Q2503* 3 , SRC p.R391C 2C.2 , STAG2 p.L405Sfs*20 3 | 1A.1 | CCND1 amp 2C.2 , CTNND2 amp 3 , FAM135B amp 3 , FGF19 amp 2D , FGF3 amp 2D , FGF4 amp 2D , IL7R amp 3 , MDM2 amp 2C.2 , MYC amp 2C.2 , PLCG1 amp 3 , RICTOR amp 2C.2 , SRC amp 2C.2 , TOP1 amp 2D | 2C.2 |  | NL | 1A.1 |
| SP105922 | Thyroid | OTHER |  | NL |  | NL |  | NL | NL |
| SP10635 | Breast | BREAST | CTCF 3 , MAP2K4 . 2C.1 , NCOR1 p.V188Nfs*10 3 | 2C.1 |  | NL |  | NL | 2C.1 |
| SP106560 | Kidney | OTHER | BAP1 2C.1 | 2C.1 |  | NL |  | NL | 2C.1 |
| SP106577 | Kidney | OTHER | DNMT3A 3 , SMARCA4 p.K991E 2C.2 | 2C.2 |  | NL |  | NL | 2C.2 |
| SP106602 | Kidney | OTHER | FAT1 3 | 3 |  | NL |  | NL | 3 |
| SP106631 | Liver | OTHER | BAP1 2C.1 , KEAP1 p.Y525C 2C.2 | 2C.1 |  | NL |  | NL | 2C.1 |
| SP106638 | Kidney | OTHER | FBXW7 2C.2 , MET p.M1268T 2C.1 | 2C.1 |  | NL |  | NL | 2C.1 |
| SP106656 | Kidney | OTHER |  | NL |  | NL |  | NL | NL |
| SP106677 | Liver | OTHER | TP53 2C.2 | 2C.2 | AKT3 amp 2C.2 , FAM135B amp 3 , MCL1 amp 2D , NTRK1 amp 2C.1 , RIT1 amp 3 | 2C.1 |  | NL | 2C.1 |
| SP106743 | Liver | OTHER | CASP8 2D , KRAS p.R68M 2C.1 | 2C.1 | AR amp 2C.2 , ARAF amp 2C.1 , EIF1AX amp 3 , SMC1A amp 3 | 2C.1 |  | NL | 2C.1 |
| SP107575 | Cervix | OTHER | STK11 2C.1 | 2C.1 |  | NL |  | NL | 2C.1 |
| SP107595 | Cervix | OTHER |  | NL | IKBKB amp 3 , IKBKB amp 3 , YAP1 amp 3 , YAP1 amp 3 | 3 |  | NL | 3 |
| SP107603 | Cervix | OTHER | FAT1 3 , FAT1 p.H2508Qfs*27 3 , NOTCH1 p.H2018Lfs*9 2C.2 , STK11 p.D53*fs*1 2C.1 | 2C.1 |  | NL |  | NL | 2C.1 |
| SP107607 | Cervix | OTHER | AXIN2 2D | 2D |  | NL |  | NL | 2D |
| SP107624 | Cervix | OTHER |  | NL |  | NL |  | NL | NL |
| SP107640 | Cervix | OTHER | FBXW7 2C.2 , MLL3 p.E3845K 3 , PIK3CA p.E545Q 2C.1 | 2C.1 |  | NL |  | NL | 2C.1 |
| SP107650 | Cervix | OTHER | FBXW7 2C.2 , MLL3 p.L2862Ffs*6 3 , SMAD4 p.H92Y 2C.2 | 2C.2 |  | NL |  | NL | 2C.2 |
| SP1086 | Bladder | OTHER | RBM10 3 , TP53 p.Y220C 2C.2 | 2C.2 | ABL2 amp 3 , CDKN2A loss 2C.1 , CDKN2B loss 2C.2 , DDR2 amp 2C.2 , KRAS amp 2C.1 | 2C.1 |  | NL | 2C.1 |
| SP109301 | Liver | OTHER | ARID1A 2C.2 , CTNNB1 p.S37F 2C.2 , DPYD p.G567V 3 , TP53 p.Q192* 2C.2 | 2C.2 |  | NL |  | NL | 2C.2 |
| SP109384 | Liver | OTHER |  | NL |  | NL |  | NL | NL |
| SP10944 | Breast | BREAST | MRE11A 2C.1 , TP53 p.E285Rfs*54 2C.2 | 2C.1 | ABL2 amp 3 , AURKA amp 3 , BCL6 amp 3 , CBL amp 3 , CCND1 amp 2C.2 , DDR2 amp 2C.2 , EGFR amp 2C.1 , ERBB2 amp 1A.1 , FAM135B amp 3 , FGF19 amp 2D , FGF3 amp 2D , FGF4 amp 2D , FGFR1 amp 2C.1 , GNAS amp 2C.2 , H3F3A amp 2C.2 , MAP2K4 loss 2C.1 , MCL1 amp 2D , MDM4 amp 2D , MITF amp 3 , MYC amp 2C.2 , MYD88 amp 2C.2 , NTRK1 amp 2C.1 , PDGFRB amp 2C.1 , PIK3C2B amp 3 , PIK3CA amp 2C.1 , PLCG1 amp 3 , RAF1 amp 2C.2 , RIT1 amp 3 , SPOP amp 3 , SRC amp 2C.2 , TOP1 amp 2D , TPMT amp 3 , YAP1 amp 3 , ZNF217 amp 3 | 1A.1 | FGFR1 fusion 2C.1 | 2C.1 | 1A.1 |
| SP109457 | Kidney | OTHER | SMARCA4 2C.2 | 2C.2 | CDKN2A loss 2C.1 , CDKN2B loss 2C.2 , MTAP loss 3 | 2C.1 |  | NL | 2C.1 |
| SP109470 | Kidney | OTHER | NF2 2C.1 , NFE2L2 p.T80K 3 , SMARCB1 p.T357*fs*1 2D | 2C.1 |  | NL |  | NL | 2C.1 |
| SP109478 | Kidney | OTHER | SRSF2 3 | 3 |  | NL |  | NL | 3 |
| SP109544 | Kidney | OTHER | ARID1A 2C.2 , CREBBP p.S32L 2C.2 | 2C.2 |  | NL |  | NL | 2C.2 |
| SP109649 | Prostate | PROSTATE |  | NL |  | NL |  | NL | NL |
| SP109801 | Cervix | OTHER | ARID1B 2D , CDKN2A p.D146G 2C.1 | 2C.1 | BCL6 amp 2C.2 , CTNND2 amp 3 , FAT1 loss 3 , GATA2 amp 2C.2 , IL7R amp 3 , MECOM amp 3 , PIK3CA amp 2C.1 , PIK3CB amp 2C.1 , RICTOR amp 2C.2 , TERT amp 3 | 2C.1 |  | NL | 2C.1 |
| SP109941 | Cervix | OTHER | TGFBR2 2D | 2D |  | NL |  | NL | 2D |
| SP109953 | Cervix | OTHER | ACVR2A 3 , FBXW7 p.R465L 2C.2 , KRAS p.G12D 2C.1 | 2C.1 | PTEN loss 2C.1 | 2C.1 |  | NL | 2C.1 |
| SP109957 | Cervix | OTHER | ERBB2 2C.1 | 2C.1 | BCL6 amp 2C.2 , MECOM amp 3 , MECOM amp 3 , PIK3CA amp 2C.1 | 2C.1 |  | NL | 2C.1 |
| SP11045 | Breast | BREAST |  | NL | ERBB2 amp 1A.1 , MCL1 amp 2D , RPS6KB1 amp 3 , SPOP amp 3 | 1A.1 |  | NL | 1A.1 |
| SP110847 | Esophagus | OTHER | APC 2C.2 , MGA p.V1369A 3 , SMAD4 p.A118V 2C.2 , TP53 p.V173M 2C.2 | 2C.2 |  | NL |  | NL | 2C.2 |
| SP110849 | Esophagus | OTHER | ASXL1 2C.2 , KRAS p.G13D 2C.1 , MAP3K1 p.R1355H 2C.2 , PIK3CA p.E542K 2C.1 , PPM1D p.T483I 3 , SMAD4 p.P356S 2C.2 , TP53 p.C176F 2C.2 | 2C.1 | PTEN loss 2C.1 , SDHA loss 2C.2 | 2C.1 |  | NL | 2C.1 |
| SP111024 | Esophagus | OTHER | ARID2 3 , TP53 p.R306* 2C.2 | 2C.2 | ERBB2 amp 2C.1 | 2C.1 |  | NL | 2C.1 |
| SP111070 | Esophagus | OTHER | ARID1A 2C.2 , CTNNB1 p.S37F 2C.2 , SMARCA4 p.E920K 2C.2 | 2C.2 | CDKN2A loss 2C.1 , CDKN2B loss 2C.2 , MTAP loss 3 | 2C.1 |  | NL | 2C.1 |
| SP111095 | Esophagus | OTHER | ERBB3 2C.2 , TP53 p.K132N 2C.2 | 2C.2 | ADAMTS12 loss 3 , BRAF amp 2C.1 , KRAS amp 2C.1 | 2C.1 |  | NL | 2C.1 |
| SP111099 | Esophagus | OTHER | APC 2C.2 , TP53 p.V173M 2C.2 | 2C.2 | SMAD4 loss 2C.2 | 2C.2 |  | NL | 2C.2 |
| SP111175 | Esophagus | OTHER | ATM 2C.1 , EPHA2 p.E825K 2C.2 , NOTCH2 p.R2453W 2C.2 , TP53 p.R273H 2C.2 | 2C.1 |  | NL |  | NL | 2C.1 |
| SP1114 | Bladder | OTHER | ERBB3 2C.2 , FGFR3 p.S249C 1A.1 , KDM6A p.E278* 2C.2 , STAG2 p.K917*fs*1 3 , TSC2 p.E656* 2C.1 | 1A.1 | CDKN2A loss 2C.1 , CDKN2B loss 2C.2 , MTAP loss 3 | 2C.1 |  | NL | 1A.1 |
| SP11171 | Breast | BREAST | TP53 2C.2 | 2C.2 | ACVR1B loss 3 , PTEN loss 2C.1 , RB1 loss 2C.2 | 2C.1 |  | NL | 2C.1 |
| SP11235 | Breast | BREAST |  | NL | RB1 loss 2C.2 | 2C.2 |  | NL | 2C.2 |
| SP11292 | Breast | BREAST | TP53 2C.2 | 2C.2 | DDR2 amp 2C.2 | 2C.2 |  | NL | 2C.2 |
| SP113197 | Skin | SKIN |  | NL | CCND1 amp 2C.2 , FGF19 amp 2D , FGF3 amp 2D , FGF3 amp 2D , FGF4 amp 2D , H3F3B amp 3 , SETBP1 amp 3 , USP8 amp 3 , USP8 amp 3 , USP8 amp 3 | 2C.2 |  | NL | 2C.2 |
| SP1132 | Bladder | OTHER | ARID1A 2C.2 , ERBB3 p.H228Q 2C.2 , NOTCH1 p.S2467* 2C.2 , RB1 p.E31Vfs*17 2C.2 , TP53 p.R248Q 2C.2 | 2C.2 |  | NL |  | NL | 2C.2 |
| SP113926 | Kidney | OTHER | PTEN 2C.1 | 2C.1 |  | NL |  | NL | 2C.1 |
| SP114016 | Cervix | OTHER | FLT4 2C.2 , MLL2 p.Q2380* 3 , MLL2 p.R4282* 3 , PIK3CA p.E545K 2C.1 | 2C.1 | BCL6 amp 2C.2 , MECOM amp 3 , PIK3CA amp 2C.1 | 2C.1 |  | NL | 2C.1 |
| SP114020 | Cervix | OTHER | ARID2 3 , RAD50 p.H158Y 2C.1 | 2C.1 | FAT1 loss 3 , PTEN loss 2C.1 | 2C.1 |  | NL | 2C.1 |
| SP114032 | Cervix | OTHER | FGFR2 2C.1 , KRAS p.G12V 2C.1 | 2C.1 | MECOM amp 3 | 3 |  | NL | 2C.1 |
| SP1144 | Bladder | OTHER | ABL1 2C.1 , MLL2 p.E730* 3 , RB1 p.Q850* 2C.2 , TP53 p.R213Q 2C.2 | 2C.1 | AKT2 amp 2C.2 , CCNE1 amp 2C.2 | 2C.2 |  | NL | 2C.1 |
| SP115162 | Head/Neck | OTHER | FGFR3 2C.1 | 2C.1 |  | NL |  | NL | 2C.1 |
| SP115498 | Liver | OTHER | TBX3 2D , TP53 p.G266R 2C.2 | 2C.2 | RPTOR amp 3 , TET2 loss 2C.2 | 2C.2 |  | NL | 2C.2 |
| SP115501 | Liver | OTHER | NRAS 2C.1 | 2C.1 | AURKA amp 3 , GNAS amp 2C.2 , ZNF217 amp 3 | 2C.2 |  | NL | 2C.1 |
| SP115830 | Liver | OTHER | BARD1 2C.1 | 2C.1 |  | NL |  | NL | 2C.1 |
| SP116604 | Lymphoid | OTHER | ARID1A 2C.2 , MLL2 p.D4855Ifs*3 3 | 2C.2 |  | NL |  | NL | 2C.2 |
| SP116606 | Lymphoid | OTHER | ARID5B 3 , ARID5B p.I35F 3 , SETD2 p.A2339V 2D , SOCS1 p.I67F 3 , SOCS1 p.E176D 3 , SPOP p.M117I 3 | 2D |  | NL |  | NL | 2D |
| SP116608 | Lymphoid | OTHER | MYD88 2C.2 | 2C.2 | CDKN2A loss 2C.1 | 2C.1 |  | NL | 2C.1 |
| SP116610 | Lymphoid | OTHER | CREBBP 2C.2 , TP53 p.Y236S 2C.2 | 2C.2 | XPO1 amp 2C.2 | 2C.2 |  | NL | 2C.2 |
| SP116612 | Lymphoid | OTHER | ATM 2C.1 , CD79B p.Y197C 3 , FGFR2 p.G647E 2C.1 , IRF4 p.L70V 3 , MYD88 p.L273P 2C.2 | 2C.1 | BCL2L12 amp 3 , CDKN2A loss 2C.1 | 2C.1 |  | NL | 2C.1 |
| SP116614 | Lymphoid | OTHER | B2M 2C.2 , CARD11 p.D230N 2C.2 , CREBBP p.W592* 2C.2 , EZH2 p.Y641N 2C.2 , MLL2 p.W2818Gfs*33 3 , MLL2 p.F3185Lfs*12 3 | 2C.2 |  | NL |  | NL | 2C.2 |
| SP116616 | Lymphoid | OTHER | CREBBP 2C.2 , EZH2 p.Y641F 2C.2 , GNA13 p.S90Tfs*4 3 , MYD88 p.S219C 2C.2 | 2C.2 |  | NL |  | NL | 2C.2 |
| SP116618 | Lymphoid | OTHER | CREBBP 2C.2 | 2C.2 |  | NL |  | NL | 2C.2 |
| SP116620 | Lymphoid | OTHER | B2M 2C.2 , B2M p.I27N 2C.2 , B2M p.L7* 2C.2 , CREBBP p.N199Rfs*10 2C.2 , EZH2 p.Y641N 2C.2 , FAS p.C143R 3 , PHF6 p.V250A 3 | 2C.2 |  | NL |  | NL | 2C.2 |
| SP116622 | Lymphoid | OTHER | FAT1 3 , STAT6 p.E372K 3 , TNFRSF14 p.W12* 3 | 3 |  | NL |  | NL | 3 |
| SP116624 | Lymphoid | OTHER | HNF1A 2C.2 | 2C.2 | BCL2 amp 2C.2 , CDKN2A loss 2C.1 , CDKN2B loss 2C.2 , MTAP loss 3 , PRDM1 loss 3 | 2C.1 |  | NL | 2C.1 |
| SP116627 | Lymphoid | OTHER | BCOR 3 , FAT1 p.D2349N 3 , MLL2 p.Q3681* 3 | 3 | CDKN2A loss 2C.1 , CDKN2B loss 2C.2 , MTAP loss 3 | 2C.1 |  | NL | 2C.1 |
| SP116630 | Lymphoid | OTHER | GPS2 3 , TP53 p.L257P 2C.2 | 2C.2 |  | NL |  | NL | 2C.2 |
| SP116635 | Lymphoid | OTHER | DDX3X 3 , JAK1 p.K696I 2D | 2D |  | NL |  | NL | 2D |
| SP116638 | Lymphoid | OTHER | CREBBP 2C.2 , MLL2 p.R1903* 3 | 2C.2 |  | NL |  | NL | 2C.2 |
| SP116642 | Lymphoid | OTHER | B2M 2C.2 , CREBBP p.Q790* 2C.2 , CYLD p.C802S 3 , SOCS1 p.T100I 3 | 2C.2 |  | NL |  | NL | 2C.2 |
| SP116645 | Lymphoid | OTHER | CREBBP 2C.2 , KIT p.P838H 2C.1 , SRSF2 p.P95L 3 , STAT6 p.G416R 3 , TP53 p.D281N 2C.2 | 2C.1 |  | NL |  | NL | 2C.1 |
| SP116648 | Lymphoid | OTHER | ATRX 2C.2 , ATRX p.Y2163H 2C.2 , B2M p.L74* 2C.2 , B2M p.G63* 2C.2 , FANCD2 p.L139F 2C.1 , IRF4 p.K59R 3 , IRF4 p.K55R 3 | 2C.1 | AR amp 2C.2 , ARAF amp 2C.1 , EIF1AX amp 3 , SMC1A amp 3 | 2C.1 |  | NL | 2C.1 |
| SP116649 | Lymphoid | OTHER | CREBBP 2C.2 , STAT6 p.G416R 3 | 2C.2 |  | NL |  | NL | 2C.2 |
| SP116654 | Lymphoid | OTHER | ARID1A 2C.2 , ETV6 p.R418S 2C.2 , MLL2 p.L1155Rfs*9 3 , PHF6 p.N316K 3 , TNFRSF14 p.P10Gfs*10 3 , TNFRSF14 p.W7Cfs*13 3 | 2C.2 |  | NL |  | NL | 2C.2 |
| SP116657 | Lymphoid | OTHER | CD79B 3 , KDM6A p.R1351* 2C.2 , MYD88 p.L273P 2C.2 , PRDM1 p.R137G 3 | 2C.2 | BCL2 amp 2C.2 | 2C.2 |  | NL | 2C.2 |
| SP116659 | Lymphoid | OTHER | CYLD 3 , EZH2 p.Y641N 2C.2 , FOXO1 p.R21H 3 , MLL2 p.K4756Nfs*41 3 , MYC p.A59T 2C.2 , TNFRSF14 p.P167Rfs*23 3 | 2C.2 |  | NL |  | NL | 2C.2 |
| SP116663 | Lymphoid | OTHER | B2M 2C.2 , B2M p.L7* 2C.2 | 2C.2 |  | NL |  | NL | 2C.2 |
| SP116665 | Lymphoid | OTHER |  | NL |  | NL |  | NL | NL |
| SP116668 | Lymphoid | OTHER | EP300 2C.2 , FOXO1 p.R21H 3 , HLA-A p.R205C 2C.2 , HLA-A p.Q204* 2C.2 , MAP2K1 p.K57T 2C.1 , MLL p.L2930I 3 , NRAS p.Q61H 2C.1 , SMAD4 p.L389Ffs*4 2C.2 , SOCS1 p.S116N 3 , TET2 p.P1194L 2C.2 , TET2 p.T848Kfs*15 2C.2 | 2C.1 |  | NL |  | NL | 2C.1 |
| SP116670 | Lymphoid | OTHER | HLA-A 2C.2 , HLA-A p.Q179* 2C.2 , HLA-B p.Q111* 3 , HLA-B p.Q139* 3 , HLA-B p.P274S 3 , MLL2 p.Q1754* 3 , SOCS1 p.Q175H 3 , SOCS1 p.E176K 3 , SOCS1 p.E176D 3 | 2C.2 | TP63 loss 3 | 3 |  | NL | 2C.2 |
| SP116672 | Lymphoid | OTHER | EP300 2C.2 , TNFRSF14 p.C42Sfs*22 3 | 2C.2 |  | NL |  | NL | 2C.2 |
| SP116674 | Lymphoid | OTHER | MLL2 3 , TP53 p.R273C 2C.2 | 2C.2 | CDK6 amp 2C.1 , TNFAIP3 loss 3 | 2C.1 |  | NL | 2C.1 |
| SP116676 | Lymphoid | OTHER | ARID1A 2C.2 , MEN1 p.L269P 2C.2 , SOCS1 p.E91Afs*24 3 , SOCS1 p.V103M 3 , STAT6 p.N417Y 3 , STAT6 p.D419A 3 , XPO1 p.E571K 2C.2 | 2C.2 |  | NL |  | NL | 2C.2 |
| SP116679 | Lymphoid | OTHER | EZH2 2C.2 , MLL2 p.Y2199Ifs*65 3 | 2C.2 |  | NL |  | NL | 2C.2 |
| SP116683 | Lymphoid | OTHER | CREBBP 2C.2 , TET2 p.Q1034* 2C.2 | 2C.2 |  | NL |  | NL | 2C.2 |
| SP116686 | Lymphoid | OTHER | SETD2 2D , STAG2 p.R370W 3 , STAT3 p.E594K 2C.2 , TNFAIP3 p.Y306S 3 , TNFRSF14 p.Q151* 3 | 2C.2 |  | NL |  | NL | 2C.2 |
| SP116688 | Lymphoid | OTHER | ARID1A 2C.2 , GNA13 p.Y343H 3 , MYC p.T73N 2C.2 | 2C.2 |  | NL |  | NL | 2C.2 |
| SP116690 | Lymphoid | OTHER | ARID5B 3 , BCL6 p.C576G 2C.2 , PALB2 p.F23L 2C.1 , SOCS1 p.L162R 3 , TP53 p.R249S 2C.2 | 2C.1 |  | NL |  | NL | 2C.1 |
| SP116694 | Lymphoid | OTHER | B2M 2C.2 , CREBBP p.I1649Hfs*11 2C.2 , EZH2 p.Y641N 2C.2 , GNA13 p.R264H 3 , MLL2 p.A2983Lfs*21 3 , RB1 p.F721Vfs*30 2C.2 | 2C.2 |  | NL |  | NL | 2C.2 |
| SP116697 | Lymphoid | OTHER | FUBP1 3 , HLA-A p.G144D 2C.2 , HNF1A p.N266K 2C.2 , IRF4 . 3 , KDM5C p.F383L 3 , KDM6A p.E991K 2C.2 , POT1 p.E390K 3 , SMARCA4 p.R704W 2C.2 | 2C.2 |  | NL | NOTCH1 fusion 3 | 3 | 2C.2 |
| SP116701 | Lymphoid | OTHER | MLL2 3 , MLL2 p.Q3341* 3 , TNFAIP3 p.A125Gfs*15 3 | 3 |  | NL |  | NL | 3 |
| SP116703 | Lymphoid | OTHER | EZH2 2C.2 | 2C.2 |  | NL |  | NL | 2C.2 |
| SP116706 | Lymphoid | OTHER | CARD11 2C.2 , MLL2 p.E2290Sfs*31 3 , MLL2 p.L3897* 3 , STAT3 p.S614R 2C.2 , TNFAIP3 p.R45Tfs*50 3 | 2C.2 |  | NL |  | NL | 2C.2 |
| SP116709 | Lymphoid | OTHER | CREBBP 2C.2 , CYLD p.K767* 3 , MLL2 p.I1058*fs*1 3 , SRSF2 p.G93R 3 , STAT3 p.G618R 2C.2 , STAT6 p.D419G 3 | 2C.2 |  | NL |  | NL | 2C.2 |
| SP116712 | Lymphoid | OTHER | MSH6 1B , MYC p.A59V 2C.2 , NRAS p.G12D 2C.1 | 1B |  | NL |  | NL | 1B |
| SP116715 | Lymphoid | OTHER | HLA-B 3 , MLL2 p.R5086* 3 , PTCH1 p.A392Gfs*47 2C.1 , SOCS1 p.L177M 3 | 2C.1 | CBL amp 3 , MCL1 amp 2D | 2D |  | NL | 2C.1 |
| SP116718 | Lymphoid | OTHER | CARD11 2C.2 , FOXO1 p.T24A 3 | 2C.2 |  | NL |  | NL | 2C.2 |
| SP116720 | Lymphoid | OTHER | CREBBP 2C.2 , MLL2 p.Q3371* 3 | 2C.2 | ARID1B loss 2D | 2D |  | NL | 2C.2 |
| SP116723 | Lymphoid | OTHER | MLL2 3 , TNFRSF14 p.P55L 3 | 3 |  | NL |  | NL | 3 |
| SP116725 | Lymphoid | OTHER | ARID1A 2C.2 , PTEN p.I101T 2C.1 , TP53 p.S241P 2C.2 | 2C.1 |  | NL |  | NL | 2C.1 |
| SP116726 | Lymphoid | OTHER | CARD11 2C.2 , FAT1 p.C3892Y 3 , MLL2 p.Q3679* 3 , MYD88 p.L273P 2C.2 | 2C.2 | CDKN2A loss 2C.1 , CDKN2B loss 2C.2 | 2C.1 |  | NL | 2C.1 |
| SP1174 | Bladder | OTHER | ATM 2C.1 , BLM p.K653M 2C.1 , CASP8 p.R491* 2D , MLL2 p.Y1514* 3 , MLL2 p.D2325Qfs*49 3 , MLL3 p.R1349* 3 , NFE2L2 p.D29Y 3 | 2C.1 | CDKN2A loss 2C.1 , CDKN2B loss 2C.2 , RAC1 amp 3 | 2C.1 |  | NL | 2C.1 |
| SP11808 | Breast | BREAST | ATM 2C.1 | 2C.1 | BCL6 amp 3 , ERBB2 amp 1A.1 , ERBB2 amp 1A.1 , H3F3B amp 3 , RARA amp 3 , RPS6KB1 amp 3 , SPOP amp 3 | 1A.1 | ERBB2 fusion 2C.1 | 2C.1 | 1A.1 |
| SP11878 | Breast | BREAST | DDX5 3 , TCF7L2 p.A419T 3 | 3 | DDR2 amp 2C.2 , MYC amp 2C.2 , PXDNL amp 3 , YES1 amp 3 | 2C.2 |  | NL | 2C.2 |
| SP11948 | Breast | BREAST | NBN 2C.1 , PIK3CA p.H1047Y 1A.1 , TP53 p.R273H 2C.2 , TP53 p.D208G 2C.2 | 1A.1 | AKT3 amp 2C.2 , CCND2 amp 2C.2 , CHD4 amp 3 , FAM135B amp 3 , FGF23 amp 3 , SETBP1 amp 3 | 2C.2 |  | NL | 1A.1 |
| SP119755 | Colon/Rectum | COLON/RECTUM | B2M 2C.2 , CHD4 p.R975H 3 , FBXW7 p.R224* 2C.2 , FBXW7 p.R465C 2C.2 , SMAD2 p.P305A 3 | 2C.2 |  | NL |  | NL | 2C.2 |
| SP12049 | Breast | BREAST | ALK 2C.1 , MLL3 p.P3278Lfs*3 3 , TP53 p.C176Y 2C.2 | 2C.1 | CCND2 amp 2C.2 , FAM135B amp 3 , FAM135B amp 3 , FGF23 amp 3 | 2C.2 |  | NL | 2C.1 |
| SP120767 | Thyroid | OTHER | BRAF 1A.1 , TBX3 p.Q115* 2D | 1A.1 |  | NL |  | NL | 1A.1 |
| SP121761 | Bone/SoftTissue | OTHER |  | NL | ABL2 amp 3 , CDK4 amp 2C.1 , DDR2 amp 2C.2 , MDM2 amp 2C.2 , MDM2 amp 2C.2 , MDM2 amp 2C.2 , MDM2 amp 2C.2 , NTRK1 amp 2C.1 , NTRK1 amp 2C.1 , STAT6 amp 3 | 2C.1 |  | NL | 2C.1 |
| SP121763 | Bone/SoftTissue | OTHER |  | NL | MDM2 amp 2C.2 , PTPN11 amp 2C.2 , PTPN11 amp 2C.2 , TERT amp 3 | 2C.2 |  | NL | 2C.2 |
| SP121774 | Bone/SoftTissue | OTHER | FLT4 2C.2 | 2C.2 | CDK4 amp 2C.1 , MDM2 amp 2C.2 | 2C.1 |  | NL | 2C.1 |
| SP121781 | Bone/SoftTissue | OTHER | ATRX 2C.2 | 2C.2 | CDKN2A loss 2C.1 , CDKN2B loss 2C.2 , MTAP loss 3 , NTRK1 amp 2C.1 , RAF1 amp 2C.2 , SMO amp 2C.1 , TERT amp 3 | 2C.1 |  | NL | 2C.1 |
| SP121783 | Bone/SoftTissue | OTHER |  | NL | CDK4 amp 2C.1 , MAP2K1 amp 2C.1 , MAP2K1 amp 2C.1 , MDM2 amp 2C.2 , MDM2 amp 2C.2 , MDM2 amp 2C.2 , TERT amp 3 | 2C.1 | TERT fusion 2C.2 | 2C.2 | 2C.1 |
| SP121790 | Bone/SoftTissue | OTHER |  | NL | CCNE1 amp 2C.2 , CDK4 amp 2C.1 , CHD4 amp 3 , ERBB3 amp 2C.2 , FGFR4 amp 2C.1 , FLT4 amp 2C.2 , MDM2 amp 2C.2 , MDM2 amp 2C.2 , MDM2 amp 2C.2 , PCBP1 amp 3 , PDGFRB amp 2C.1 , PRKACA amp 3 , SLCO1B3 amp 3 , STAT6 amp 3 , XPO1 amp 2C.2 | 2C.1 |  | NL | 2C.1 |
| SP121808 | Bone/SoftTissue | OTHER |  | NL | CCND2 amp 2C.2 , CDK4 amp 2C.1 , ERBB3 amp 2C.2 , FGF23 amp 3 , MDM2 amp 2C.2 , MDM2 amp 2C.2 , MDM2 amp 2C.2 | 2C.1 |  | NL | 2C.1 |
| SP121811 | Bone/SoftTissue | OTHER |  | NL | CDK4 amp 2C.1 , MDM2 amp 2C.2 , MDM2 amp 2C.2 , MDM2 amp 2C.2 , STAT6 amp 3 | 2C.1 |  | NL | 2C.1 |
| SP121816 | Bone/SoftTissue | OTHER |  | NL | CDK4 amp 2C.1 , MDM2 amp 2C.2 , MDM2 amp 2C.2 , MDM4 amp 2D , PIK3C2B amp 3 , PIK3C2B amp 3 | 2C.1 | TERT fusion 2C.2 | 2C.2 | 2C.1 |
| SP121824 | Bone/SoftTissue | OTHER |  | NL | AXL amp 2C.2 , CDK4 amp 2C.1 , CDK4 amp 2C.1 , MDM2 amp 2C.2 , MDM2 amp 2C.2 , MDM2 amp 2C.2 , MDM2 amp 2C.2 , MDM2 amp 2C.2 | 2C.1 | AXL fusion 2C.2 | 2C.2 | 2C.1 |
| SP121828 | Bone/SoftTissue | OTHER |  | NL | CDK4 amp 2C.1 , FGFR1 amp 2C.1 , MDM2 amp 2C.2 , MDM2 amp 2C.2 , MDM2 amp 2C.2 , MDM2 amp 2C.2 , MDM2 amp 2C.2 , MDM2 amp 2C.2 , PLCG1 amp 3 , TERT amp 3 , TERT amp 3 , TERT amp 3 , TOP1 amp 2D | 2C.1 | JAK2 fusion 2C.1 | 2C.1 | 2C.1 |
| SP121831 | Bone/SoftTissue | OTHER |  | NL | CCNE1 amp 2C.2 , CDK4 amp 2C.1 , MDM2 amp 2C.2 | 2C.1 |  | NL | 2C.1 |
| SP121837 | Bone/SoftTissue | OTHER |  | NL | ABL1 amp 2C.1 , CCND3 amp 2C.2 , CCND3 amp 2C.2 , CCND3 amp 2C.2 , CDK4 amp 2C.1 , MDM2 amp 2C.2 , MDM2 amp 2C.2 , PPP6C amp 3 | 2C.1 |  | NL | 2C.1 |
| SP121841 | Bone/SoftTissue | OTHER |  | NL | CDK4 amp 2C.1 , MDM2 amp 2C.2 | 2C.1 |  | NL | 2C.1 |
| SP121847 | Bone/SoftTissue | OTHER |  | NL | ATRX loss 2C.2 , ATRX loss 2C.2 , CDK4 amp 2C.1 , ERBB3 amp 2C.2 , ERBB3 amp 2C.2 , FGFR4 amp 2C.1 , FLT4 amp 2C.2 , MDM2 amp 2C.2 , MDM2 amp 2C.2 , PIK3CB amp 2C.1 | 2C.1 |  | NL | 2C.1 |
| SP121852 | Bone/SoftTissue | OTHER |  | NL | AR amp 2C.2 , ARAF amp 2C.1 , CDK4 amp 2C.1 , EIF1AX amp 3 , MDM2 amp 2C.2 , MDM2 amp 2C.2 , MDM2 amp 2C.2 , MDM2 amp 2C.2 , MDM2 amp 2C.2 , NF1 loss 2C.1 , NF1 loss 2C.1 , SMC1A amp 3 | 2C.1 |  | NL | 2C.1 |
| SP121859 | Bone/SoftTissue | OTHER |  | NL | CDK4 amp 2C.1 , CDKN2A loss 2C.1 , CDKN2B loss 2C.2 , MDM2 amp 2C.2 , MDM2 amp 2C.2 , MDM2 amp 2C.2 , PDCD1 loss 2C.2 | 2C.1 |  | NL | 2C.1 |
| SP12186 | Breast | BREAST | NOTCH2 2C.2 , PIK3CA p.E542K 1A.1 , TP53 p.A161T 2C.2 | 1A.1 |  | NL |  | NL | 1A.1 |
| SP121861 | Bone/SoftTissue | OTHER |  | NL | CDK4 amp 2C.1 , MDM2 amp 2C.2 , MDM2 amp 2C.2 | 2C.1 |  | NL | 2C.1 |
| SP121865 | Bone/SoftTissue | OTHER | RB1 2C.2 , RET p.R886Q 2C.1 | 2C.1 | IDH2 amp 2C.1 , IGF1R amp 2D | 2C.1 |  | NL | 2C.1 |
| SP121870 | Bone/SoftTissue | OTHER | PIK3CA 2C.1 | 2C.1 | CDKN2A loss 2C.1 , CDKN2B loss 2C.2 | 2C.1 |  | NL | 2C.1 |
| SP122361 | Bone/SoftTissue | OTHER |  | NL | CCND3 amp 2C.2 , CTNND2 amp 3 , CTNND2 amp 3 , CTNND2 amp 3 , FLT4 amp 2C.2 , MDM2 amp 2C.2 , MDM2 amp 2C.2 , MDM2 amp 2C.2 , RAC1 amp 3 , ZNF429 amp 3 , ZNF429 amp 3 | 2C.2 | ETS1 fusion 2D , PIK3CA fusion 2C.1 | 2C.1 | 2C.1 |
| SP122372 | Bone/SoftTissue | OTHER |  | NL |  | NL |  | NL | NL |
| SP122392 | Bone/SoftTissue | OTHER | CSF1R 2C.2 , TSC1 p.R892Kfs*11 2C.1 | 2C.1 | CDKN2A loss 2C.1 , CDKN2B loss 2C.2 , CDKN2C loss 2D , CIC loss 2D , MTAP loss 3 , RAD51B loss 2C.1 | 2C.1 |  | NL | 2C.1 |
| SP122412 | Bone/SoftTissue | OTHER |  | NL | CDKN1A loss 2C.2 , CDKN2A loss 2C.1 , CDKN2B loss 2C.2 , PTEN loss 2C.1 | 2C.1 |  | NL | 2C.1 |
| SP122476 | Bone/SoftTissue | OTHER | DPYD 3 , TP53 p.G262Vfs*83 2C.2 | 2C.2 | PTEN loss 2C.1 | 2C.1 |  | NL | 2C.1 |
| SP122489 | Bone/SoftTissue | OTHER | TP53 2C.2 | 2C.2 | RB1 loss 2C.2 | 2C.2 |  | NL | 2C.2 |
| SP122560 | Bone/SoftTissue | OTHER | GATA2 2C.2 , RB1 . 2C.2 , TP53 p.E336Afs*10 2C.2 | 2C.2 |  | NL |  | NL | 2C.2 |
| SP122590 | Bone/SoftTissue | OTHER | PTEN 2C.1 | 2C.1 |  | NL |  | NL | 2C.1 |
| SP122634 | Bone/SoftTissue | OTHER | RB1 2C.2 , TP53 p.K132R 2C.2 | 2C.2 |  | NL |  | NL | 2C.2 |
| SP122676 | Bone/SoftTissue | OTHER | TP53 2C.2 | 2C.2 | LARP4B loss 3 , TSC2 loss 2C.1 | 2C.1 |  | NL | 2C.1 |
| SP122702 | Bone/SoftTissue | OTHER | RB1 2C.2 , TP53 p.R337L 2C.2 | 2C.2 | CCND3 amp 2C.2 , PIM1 amp 3 | 2C.2 |  | NL | 2C.2 |
| SP122714 | Bone/SoftTissue | OTHER |  | NL |  | NL |  | NL | NL |
| SP122725 | Bone/SoftTissue | OTHER | KRAS 2C.1 , PIK3CA p.H1047R 2C.1 , PPP2R1A p.P179R 2C.1 , TP53 p.Y205C 2C.2 | 2C.1 | CDKN2A loss 2C.1 , CDKN2B loss 2C.2 , KRAS amp 2C.1 , MTAP loss 3 , PIK3CA amp 2C.1 | 2C.1 |  | NL | 2C.1 |
| SP123010 | Bone/SoftTissue | OTHER | BRCA2 2C.1 , CCND1 p.V290M 2C.2 , KIT p.W557_E561delWKVVE 2C.1 | 2C.1 |  | NL |  | NL | 2C.1 |
| SP123836 | Kidney | OTHER |  | NL |  | NL |  | NL | NL |
| SP123840 | Kidney | OTHER |  | NL |  | NL |  | NL | NL |
| SP123842 | Kidney | OTHER | TSC1 2C.1 | 2C.1 |  | NL |  | NL | 2C.1 |
| SP123844 | Kidney | OTHER |  | NL |  | NL |  | NL | NL |
| SP123846 | Kidney | OTHER |  | NL |  | NL |  | NL | NL |
| SP123850 | Kidney | OTHER |  | NL |  | NL |  | NL | NL |
| SP123852 | Kidney | OTHER | CDKN1A 2C.2 | 2C.2 |  | NL |  | NL | 2C.2 |
| SP123854 | Kidney | OTHER |  | NL |  | NL |  | NL | NL |
| SP123856 | Kidney | OTHER |  | NL |  | NL |  | NL | NL |
| SP123858 | Kidney | OTHER |  | NL |  | NL |  | NL | NL |
| SP123870 | Kidney | OTHER |  | NL |  | NL |  | NL | NL |
| SP123872 | Kidney | OTHER | TP53 2C.2 | 2C.2 | PXDNL amp 3 , PXDNL amp 3 , PXDNL amp 3 | 3 |  | NL | 2C.2 |
| SP123874 | Kidney | OTHER | TSC2 2C.1 | 2C.1 |  | NL |  | NL | 2C.1 |
| SP123876 | Kidney | OTHER |  | NL |  | NL |  | NL | NL |
| SP123878 | Kidney | OTHER |  | NL |  | NL |  | NL | NL |
| SP123882 | Kidney | OTHER |  | NL |  | NL |  | NL | NL |
| SP123884 | Kidney | OTHER | HNF1A 2C.2 , SETD2 p.R2399* 2D | 2C.2 |  | NL |  | NL | 2C.2 |
| SP123886 | Kidney | OTHER |  | NL |  | NL |  | NL | NL |
| SP123888 | Kidney | OTHER |  | NL |  | NL |  | NL | NL |
| SP123890 | Kidney | OTHER |  | NL |  | NL |  | NL | NL |
| SP123892 | Kidney | OTHER |  | NL |  | NL |  | NL | NL |
| SP123894 | Kidney | OTHER |  | NL |  | NL |  | NL | NL |
| SP123897 | Kidney | OTHER | KDM6A 2C.2 | 2C.2 |  | NL |  | NL | 2C.2 |
| SP123900 | Kidney | OTHER |  | NL |  | NL |  | NL | NL |
| SP123902 | Kidney | OTHER | TSC1 2C.1 | 2C.1 |  | NL |  | NL | 2C.1 |
| SP123950 | Kidney | OTHER | TP53 2C.2 | 2C.2 |  | NL |  | NL | 2C.2 |
| SP123953 | Kidney | OTHER |  | NL |  | NL |  | NL | NL |
| SP123955 | Kidney | OTHER |  | NL |  | NL |  | NL | NL |
| SP123958 | Kidney | OTHER |  | NL |  | NL |  | NL | NL |
| SP123964 | Kidney | OTHER | TP53 2C.2 , TP53 p.C176F 2C.2 | 2C.2 |  | NL |  | NL | 2C.2 |
| SP123967 | Kidney | OTHER |  | NL | PTEN loss 2C.1 | 2C.1 |  | NL | 2C.1 |
| SP123969 | Kidney | OTHER |  | NL | FAM135B amp 3 , MYC amp 2C.2 , PXDNL amp 3 | 2C.2 |  | NL | 2C.2 |
| SP123972 | Kidney | OTHER | NRAS 2C.1 | 2C.1 |  | NL |  | NL | 2C.1 |
| SP123978 | Kidney | OTHER |  | NL |  | NL |  | NL | NL |
| SP123984 | Kidney | OTHER |  | NL |  | NL |  | NL | NL |
| SP123988 | Kidney | OTHER |  | NL |  | NL |  | NL | NL |
| SP123995 | Kidney | OTHER | TP53 2C.2 | 2C.2 |  | NL |  | NL | 2C.2 |
| SP123998 | Kidney | OTHER | ATM 2C.1 | 2C.1 | CDKN2C loss 2D | 2D |  | NL | 2C.1 |
| SP124003 | Kidney | OTHER | MTOR 2C.1 | 2C.1 |  | NL |  | NL | 2C.1 |
| SP124013 | Kidney | OTHER |  | NL |  | NL |  | NL | NL |
| SP124017 | Kidney | OTHER |  | NL |  | NL |  | NL | NL |
| SP124021 | Kidney | OTHER |  | NL |  | NL |  | NL | NL |
| SP124033 | Kidney | OTHER |  | NL |  | NL |  | NL | NL |
| SP124969 | Lymphoid | OTHER | BRAF 2C.1 , MLL p.F3949L 3 , MTOR p.V2006F 2C.1 , SOCS1 p.V2E 3 | 2C.1 |  | NL |  | NL | 2C.1 |
| SP124971 | Lymphoid | OTHER | TP53 2C.2 , WT1 p.A199T 2C.2 | 2C.2 | AXIN2 loss 2D , CYLD loss 3 , GNA13 loss 3 | 2D |  | NL | 2C.2 |
| SP124977 | Lymphoid | OTHER | CARD11 2C.2 , PRDM1 p.E576G 3 , SPOP p.M117R 3 | 2C.2 |  | NL |  | NL | 2C.2 |
| SP124981 | Lymphoid | OTHER | ATM 2C.1 , CD79B p.Y197C 3 , KDM6A p.R78Kfs*2 2C.2 , KDM6A p.R78Lfs*2 2C.2 , MYD88 p.L273P 2C.2 , STAG2 p.R667W 3 | 2C.1 | CDKN2A loss 2C.1 , CDKN2B loss 2C.2 , MTAP loss 3 , MTAP loss 3 | 2C.1 |  | NL | 2C.1 |
| SP127628 | Lymphoid | OTHER | CASP8 2D , CASP8 p.R292W 2D , FOXO1 p.S22T 3 , FOXO1 p.T24A 3 , MYC p.P260A 2C.2 , MYC p.Q321H 2C.2 | 2C.2 |  | NL |  | NL | 2C.2 |
| SP127630 | Lymphoid | OTHER | FOXO1 3 | 3 | CDKN2A loss 2C.1 , CDKN2B loss 2C.2 , MTAP loss 3 | 2C.1 |  | NL | 2C.1 |
| SP127632 | Lymphoid | OTHER | B2M 2C.2 , CUL4A p.T617M 3 , JAK3 p.A573V 2C.2 , TNFAIP3 p.L324Qfs*7 3 | 2C.2 |  | NL |  | NL | 2C.2 |
| SP127634 | Lymphoid | OTHER | ASXL1 2C.2 , INPP4B p.K833T 3 , MLL2 p.A1390Gfs*42 3 , RAF1 p.E478K 2C.2 | 2C.2 | CDKN2A loss 2C.1 , CDKN2B loss 2C.2 , MTAP loss 3 | 2C.1 |  | NL | 2C.1 |
| SP127636 | Lymphoid | OTHER | TET2 2C.2 | 2C.2 |  | NL |  | NL | 2C.2 |
| SP127638 | Lymphoid | OTHER | B2M 2C.2 , MLL2 p.S807Ffs*5 3 , MLL2 p.Q809Pfs*3 3 , TP53 p.E171* 2C.2 | 2C.2 | XPO1 amp 2C.2 | 2C.2 |  | NL | 2C.2 |
| SP127640 | Lymphoid | OTHER | DDX3X 3 , DDX3X p.V526G 3 , MAP2K1 p.F53L 2C.1 , PIK3CD p.E1021K 3 , RAF1 p.L476F 2C.2 , TET2 p.L1322Wfs*41 2C.2 , TET2 p.Q1051*fs*1 2C.2 | 2C.1 |  | NL |  | NL | 2C.1 |
| SP12856 | Breast | BREAST | CDH1 2D , FOXA1 p.D226G 2C.2 , PIK3CA p.E545K 1A.1 , PIK3R1 p.E515K 2C.2 , SETD2 p.R1509T 2D , STK11 p.E33K 2C.1 | 1A.1 |  | NL |  | NL | 1A.1 |
| SP13036 | Breast | BREAST | PIK3CA 1A.1 , TP53 p.R273C 2C.2 | 1A.1 | CCNE1 amp 2C.2 , CCNE1 amp 2C.2 , KRAS amp 2C.1 , SLCO1B3 amp 3 , SRC amp 2C.2 | 2C.1 |  | NL | 1A.1 |
| SP1305 | Bladder | OTHER | ERCC2 2C.2 , KDM6A p.R511* 2C.2 , NOTCH2 p.G292R 2C.2 , TP53 p.H214R 2C.2 | 2C.2 | CARD11 amp 2C.2 , EGFR amp 2C.1 , RAC1 amp 3 | 2C.1 |  | NL | 2C.1 |
| SP13072 | Cervix | OTHER | ARID1A 2C.2 , ERBB3 p.V104M 2C.2 , SMARCA4 p.R973W 2C.2 | 2C.2 |  | NL |  | NL | 2C.2 |
| SP13078 | Cervix | OTHER |  | NL |  | NL |  | NL | NL |
| SP13084 | Cervix | OTHER | EP300 2C.2 , PHF6 p.R274* 3 | 2C.2 | PTEN loss 2C.1 | 2C.1 |  | NL | 2C.1 |
| SP13206 | Cervix | OTHER | EP300 2C.2 | 2C.2 | PRKACA amp 3 , YAP1 amp 3 | 3 |  | NL | 2C.2 |
| SP13242 | Cervix | OTHER | CASP8 2D | 2D | YAP1 amp 3 | 3 |  | NL | 2D |
| SP1365 | Bladder | OTHER | MLL2 3 , MLL2 p.P4423Hfs*9 3 , TP53 p.C238F 2C.2 | 2C.2 | CDKN2A loss 2C.1 , CDKN2B loss 2C.2 , FGFR3 amp 2C.1 , FOXA1 amp 3 , H3F3B amp 3 , MDM4 amp 2D , MTAP loss 3 , TERT amp 3 | 2C.1 |  | NL | 2C.1 |
| SP1377 | Bladder | OTHER | ELF3 3 , KDM6A p.P1107Lfs*13 2C.2 , NF1 p.E1889K 2C.1 , TSC1 p.L536* 2C.1 | 2C.1 | CDKN2A loss 2C.1 , CDKN2B loss 2C.2 , FGFR3 amp 2C.1 , MTAP loss 3 | 2C.1 |  | NL | 2C.1 |
| SP1419 | Bladder | OTHER | MECOM 3 , NOTCH1 p.D260Y 2C.2 | 2C.2 | CCND2 amp 2C.2 , CHD4 amp 3 , CTNND2 amp 3 , FGF23 amp 3 , FGFR1 amp 2C.1 , IL7R amp 3 , MECOM amp 3 , NTRK3 amp 2C.1 , RICTOR amp 2C.2 , TERT amp 3 | 2C.1 |  | NL | 2C.1 |
| SP1431 | Bladder | OTHER | ARID1A 2C.2 , ELF3 p.D195*fs*1 3 , MLL p.D2842N 3 , MLL p.E2509K 3 , NCOR1 p.R627* 3 , TET2 p.S509* 2C.2 | 2C.2 | CDKN2A loss 2C.1 , CDKN2B loss 2C.2 , KDM6A loss 2C.2 , MAP2K4 loss 2C.1 , MTAP loss 3 | 2C.1 |  | NL | 2C.1 |
| SP1491 | Bladder | OTHER | BRCA2 2C.1 , ERCC2 p.S44L 2C.2 , HRAS p.Q61K 2C.1 | 2C.1 |  | NL |  | NL | 2C.1 |
| SP1677 | Bladder | OTHER | CDKN1A 2C.2 , CREBBP p.Q887* 2C.2 , FGFR3 p.G380R 1A.1 , GPS2 p.S113* 3 , JAK3 p.L575F 2C.2 , KDM6A p.V91Gfs*9 2C.2 , SMARCA4 p.T786N 2C.2 | 1A.1 | CDKN2A loss 2C.1 , CDKN2B loss 2C.2 , MTAP loss 3 | 2C.1 |  | NL | 1A.1 |
| SP16886 | Colon/Rectum | COLON/RECTUM | ACVR2A 3 , APC p.R1114* 2C.2 , APC p.T1556Nfs*3 2C.2 , ARID2 p.S1783* 3 , ARID2 p.I403S 3 , ASXL1 p.E537K 2C.2 , ATM p.R337C 2C.1 , ATR p.E706* 2C.2 , ATRX p.A2137T 2C.2 , B2M p.L43R 2C.2 , BARD1 p.R520I 2C.1 , BRCA2 p.E1493* 2C.1 , CASP8 p.C404F 2D , CHEK2 p.N290Y 2C.1 , CREBBP p.A1093T 2D , DICER1 p.E707K 3 , EP300 p.R397* 2D , ERBB4 p.R938C 2C.2 , ERCC4 p.I717T 2C.2 , EZH2 p.R679H 2C.2 , FAM123B p.E933K 3 , FBXW7 p.D279Y 2C.2 , GNAS p.A109T 2C.2 , JAK2 p.M964V 2D , KRAS p.Q22K 1A.1 , KRAS p.K117N 1A.1 , MAP2K4 p.E203* 2C.1 , MAP2K4 p.E141* 2C.1 , MET p.R1166Q 2C.1 , MET p.R1188* 2C.1 , MGA p.R1242* 3 , MLL p.R3291I 3 , NF1 p.E1074G 2C.1 , PIK3CA p.R88Q 2C.1 , PIK3CA p.H1047Q 2C.1 , POLE p.P286R 2C.1 , PTEN p.E114* 2C.1 , PTEN p.W111* 2C.1 , RAD50 p.E1033* 2C.1 , RAD51C p.V156A 2C.1 , RASA1 p.R281I 3 , RET p.R959W 2C.1 , RNF43 p.D140N 2C.2 , SMAD4 p.Q534P 2C.2 , SMAD4 p.G89* 2C.2 , TBX3 p.E111* 2D , TNFAIP3 p.F678L 3 , TP53 p.R213* 2C.2 , TP53 p.S94* 2C.2 , TPMT p.S124Y 3 | 1A.1 |  | NL |  | NL | 1A.1 |
| SP16934 | Colon/Rectum | COLON/RECTUM | ACVR2A 3 , APC p.R1450* 2C.2 , APC p.S1419N 2C.2 , APC p.S940* 2C.2 , NRAS p.G12C 1.A.1 , PIK3CA p.T1052K 2C.1 , SMAD2 p.R321Q 3 | 1.A.1 |  | NL |  | NL | 1.A.1 |
| SP16958 | Colon/Rectum | COLON/RECTUM | ATM 2C.1 , BRAF p.V600E 1B , PIK3R1 p.P92Hfs*23 2C.2 , RAD51C p.R312W 2C.1 | 1B | SMAD4 loss 2C.2 | 2C.2 | RSPO3 fusion 3 | 3 | 1B |
| SP17016 | Colon/Rectum | COLON/RECTUM | ATM 2C.1 , KRAS p.G12V 1A.1 , PIK3CA p.E545K 2C.1 | 1A.1 |  | NL |  | NL | 1A.1 |
| SP1712 | Bladder | OTHER | ATM 2C.1 , CDH1 p.E386K 2D , FGFR3 p.S249C 1A.1 , PIK3CA p.E545K 2C.1 | 1A.1 | CDKN2A loss 2C.1 , MTAP loss 3 | 2C.1 |  | NL | 1A.1 |
| SP1724 | Bladder | OTHER | ARHGAP35 3 , ASXL1 p.Q225* 2C.2 , CDKN1A p.S15Rfs*18 2C.2 , FAT1 p.Q138* 3 , FAT1 p.Q4114* 3 , FGFR3 p.S249C 1A.1 , GPS2 p.Q192* 3 , STAG2 p.Q593* 3 | 1A.1 | CDKN2A loss 2C.1 , CDKN2B loss 2C.2 , MTAP loss 3 | 2C.1 |  | NL | 1A.1 |
| SP17294 | Colon/Rectum | COLON/RECTUM | APC 2C.2 , KRAS p.G12V 1A.1 , PIK3CA p.D350N 2C.1 , TP53 p.R175H 2C.2 | 1A.1 |  | NL |  | NL | 1A.1 |
| SP17329 | Colon/Rectum | COLON/RECTUM | APC 2C.2 , KRAS p.G12V 1A.1 | 1A.1 |  | NL |  | NL | 1A.1 |
| SP17430 | Colon/Rectum | COLON/RECTUM | APC 2C.2 , APC p.Q978* 2C.2 , TP53 p.Y205C 2C.2 | 2C.2 |  | NL |  | NL | 2C.2 |
| SP1781 | Bladder | OTHER | ARID1A 2C.2 , ARID1B p.S676* 2D , ATM p.E158Q 2C.1 , BLM p.D1079N 2C.1 , CDKN1B p.S7C 2D , KDM6A p.S1061* 2C.2 , KDM6A p.E315Q 2C.2 , MLL3 p.R2454T 3 , MSH3 p.E995Q 2C.2 , NF1 p.Q756* 2C.1 , PBRM1 p.E767Q 2D , PBRM1 p.E1107K 2D , PDGFRA p.S1081L 2C.1 , TP53 p.E271K 2C.2 , TSC2 p.R367W 2C.1 | 2C.1 | CCND1 amp 2C.2 , FGF19 amp 2D , FGF3 amp 2D , FGF4 amp 2D | 2C.2 |  | NL | 2C.1 |
| SP17905 | Colon/Rectum | COLON/RECTUM | ACVR1B 3 , ACVR1B p.Y451* 3 , APC p.R1450* 2C.2 , APC p.S1392* 2C.2 , APC p.R348* 2C.2 , ARID1B p.E673* 2D , ARID2 p.R314C 3 , ATM p.R1618* 2C.1 , ATM p.W1058R 2C.1 , ATM p.L612I 2C.1 , ATM p.R250* 2C.1 , ATR p.E306* 2C.2 , ATR p.R1015Q 2C.2 , ATR p.E1078* 2C.2 , ATR p.L1268F 2C.2 , ATRX p.D898Y 2C.2 , ATRX p.K2072T 2C.2 , AXIN2 p.K748I 2D , BAP1 p.D74Y 2C.1 , BRAF p.S637P 1B , BRCA1 p.E203* 2C.1 , BRCA2 p.E97* 2C.1 , BRCA2 p.E2635G 2C.1 , BRCA2 p.S445Y 2C.1 , BRIP1 p.A496V 2C.1 , CARD11 p.P100S 3 , CASP8 p.E441* 2D , CBFB p.R187C 3 , CDC73 p.R91Q 3 , CDK6 p.S204I 2C.1 , CHD4 p.R1338I 3 , CREBBP p.Y1457* 2D , CTNNB1 p.E334A 2C.2 , CUL4A p.Y558C 3 , CYLD p.E27* 3 , DICER1 p.R45K 3 , DICER1 p.R944Q 3 , DPYD p.E378* 3 , ERBB4 p.E928* 2C.2 , ERCC2 p.R88Q 2C.2 , ERCC4 p.S253F 2C.2 , FAM123B p.E383* 3 , FAS p.E114K 3 , FBXW7 p.R393Q 2C.2 , FBXW7 p.R658* 2C.2 , FGFR3 p.D722N 2C.1 , HDAC2 p.F292C 3 , HDAC9 p.R432I 3 , JAK1 p.R174Q 2D , JAK1 p.F366V 2D , JAK1 p.R755Q 2D , JAK2 p.E274* 2D , KIT p.A784T 1A.1 , KIT p.Y568S 1A.1 , MAX p.L46S 3 , MGA p.D738N 3 , MLL3 p.E198* 3 , MLL3 p.D1738Y 3 , MPL p.D47A 3 , MRE11A p.D394Y 2C.1 , MRE11A p.E460* 2C.1 , MSH3 p.E637D 2C.2 , MSH6 p.R732Q 1B , NBN p.R43Q 2C.1 , NBN p.Y322C 2C.1 , NCOR1 p.K482N 3 , NCOR1 p.D725N 3 , NCOR1 p.R933* 3 , NCOR1 p.S2337F 3 , NF1 p.I2684L 2C.1 , NF1 p.T1853A 2C.1 , NF1 p.R1434I 2C.1 , NF2 p.S506Y 2C.1 , NF2 p.K123N 2C.1 , NOTCH2 p.R1931C 2C.2 , NT5C2 p.E390K 3 , PBRM1 p.R876C 2D , PDGFRA p.K939N 2C.1 , PIK3CA p.R88Q 2C.1 , PIK3CB p.S1070Y 2C.1 , PIK3R1 p.E683K 2C.2 , PIK3R1 p.E52* 2C.2 , PIK3R2 p.Q445P 2C.2 , PMS1 p.S507* 2C.2 , POLE p.P286S 2C.1 , POLE p.P286R 2C.1 , PPP6C p.D235A 3 , RAD50 p.K6N 2C.1 , RASA1 p.K935T 3 , RASA1 p.R427Q 3 , RPL22 p.Y90H 3 , RPL5 p.E82K 3 , SMAD4 p.R361H 2C.2 , SMARCA4 p.L1383P 2C.2 , SPEN p.E2314* 3 , TET2 p.S1611Y 2D , TET2 p.S1190Y 2D , THRAP3 p.S211Y 3 , TSC1 p.E981* 2C.1 | 1A.1 |  | NL |  | NL | 1A.1 |
| SP18121 | Colon/Rectum | COLON/RECTUM | ACVR2A 3 , APC p.S1465Wfs*3 2C.2 , ARID1B p.R1843C 2D , ARID2 p.S218Pfs*74 3 , ASXL1 p.S846Qfs*5 2C.2 , AXIN2 p.E405Gfs*56 2D , BRCA2 p.T3033Lfs*29 2C.1 , CTCF p.T204Nfs*26 3 , CUL3 p.A635T 3 , DICER1 p.K1486Nfs*4 3 , FANCD2 p.K195E 2C.1 , FAT1 p.L1107I 3 , FLT4 p.A806T 2C.2 , JAK1 p.G1093C 2D , KRAS p.G13D 1A.1 , KRAS p.A146T 1A.1 , MAP3K4 p.D693V 3 , MLL2 p.R1687H 3 , MLL2 p.R5351L 3 , NF2 p.R262* 2C.1 , NOTCH1 p.R1854H 2C.2 , PIK3CA p.E726K 2C.1 , POLE p.Y473C 2C.1 , PTCH1 p.T807A 2C.1 , RNF43 p.G659Vfs*41 2C.2 , SMARCA4 p.R1369S 2C.2 , SMARCA4 p.A1218V 2C.2 , SMARCA4 p.G1136Afs*4 2C.2 , SPEN p.R2339H 3 , TBX3 p.E195K 2D , TGFBR2 p.L348P 2D | 1A.1 |  | NL |  | NL | 1A.1 |
| SP18787 | Colon/Rectum | COLON/RECTUM | ACVR1B 3 , APC p.R805* 2C.2 , APC p.L1488Yfs*19 2C.2 , ERBB2 p.V842I 2C.1 , KRAS p.G12D 1A.1 , RBM10 p.R230* 3 | 1A.1 | RARA amp 3 | 3 |  | NL | 1A.1 |
| SP18946 | Colon/Rectum | COLON/RECTUM | APC 2C.2 , APC p.S2029Y 2C.2 , APC p.F1840V 2C.2 , APC p.E1309* 2C.2 , APC p.D149G 2C.2 , ARID1B p.P1097S 2D , ARID2 p.F567C 3 , ARID5B p.S834P 3 , ARID5B p.R766I 3 , ARID5B p.S189L 3 , ATM p.E2676* 2C.1 , ATR p.R1015Q 2C.2 , ATR p.R1647H 2C.2 , ATRX p.D514Y 2C.2 , ATRX p.K1583N 2C.2 , BLM p.S1252F 2C.1 , BRIP1 p.R798Q 2C.1 , BRIP1 p.S1129Y 2C.1 , CDK4 p.R139Q 2C.1 , CHD4 p.E1094K 3 , DICER1 p.E524* 3 , DICER1 p.R944Q 3 , EP300 p.F1205L 2D , ERCC4 p.A428V 2C.2 , ESR1 p.N519S 2C.2 , FANCI p.K869N 2C.1 , FAT1 p.E3136* 3 , HDAC9 p.F853V 3 , HLA-A p.A160Hfs*15 2C.2 , HLA-A p.A160Hfs*15 2C.2 , INPP4B p.E82K 3 , INPP4B p.S850L 3 , KRAS p.K117N 1A.1 , MAP3K4 p.R275Q 3 , MECOM p.R846C 3 , MED12 p.G44C 3 , MLH3 p.E586* 2C.2 , MSH6 p.R922Q 1B , MSH6 p.A175T 1B , NBN p.E62* 2C.1 , NCOR1 p.R1794Q 3 , NOTCH2 p.L1740I 2C.2 , NOTCH2 p.D1975G 2C.2 , PIK3CA p.M1043I 2C.1 , PIK3CA p.R88Q 2C.1 , POLE p.F367S 2C.1 , PTEN p.E299* 2C.1 , PTPN11 p.E69D 2C.2 , RAD50 p.A182T 2C.1 , RASA2 p.R760I 3 , SMAD2 p.R415I 3 , SMAD2 p.S467P 3 , SMARCA4 p.W875R 2C.2 , SMARCA4 p.R381Q 2C.2 , SPEN p.R637Q 3 , TCF7L2 p.R471C 3 , TCF7L2 p.A397V 3 , TCF7L2 p.F357L 3 , TET2 p.L956I 2D , TP53 p.R213* 2C.2 , TP53 p.K132T 2C.2 | 1A.1 |  | NL |  | NL | 1A.1 |
| SP19215 | Colon/Rectum | COLON/RECTUM | ARID5B 3 , BARD1 p.K217Nfs*4 2C.1 , CASP8 p.A100D 2D , CDK12 p.F802V 2C.1 , CTCF p.T317Rfs*91 3 , DAXX p.R256C 2C.2 , EGFR p.T354M 2C.1 , EP300 p.H1377R 2D , ERBB3 p.V104M 2C.2 , FAT1 p.F434Lfs*5 3 , FLT4 p.R957H 2C.2 , HDAC2 p.G220Afs*10 3 , KDM5C p.R332Q 3 , KEAP1 p.A356V 2C.2 , MLL2 p.P2354Lfs*30 3 , MSH3 p.K383Rfs*32 2C.2 , RNF43 p.R529Q 2C.2 , SPEN p.A2105Lfs*33 3 , TGFBR1 p.R487W 3 | 2C.1 |  | NL |  | NL | 2C.1 |
| SP19295 | Colon/Rectum | COLON/RECTUM | APC 2C.2 , APC p.S2464Y 2C.2 , APC p.P2338H 2C.2 , APC p.R2311I 2C.2 , APC p.R640W 2C.2 , APC p.R499* 2C.2 , ARID1A p.R1989* 2C.2 , ATM p.F2839L 2C.1 , ATRX p.L1612I 2C.2 , BCOR p.L1009* 3 , BRCA2 p.E2129* 2C.1 , EP300 p.R568W 2D , HLA-A p.E82* 2C.2 , IDH1 p.R132C 2C.1 , KDR p.R1032Q 2D , KRAS p.A146T 1A.1 , MGA p.R1242* 3 , MGA p.R1155* 3 , MSH6 p.R1242H 1B , MTOR p.E1799K 2C.1 , NCOR1 p.R688Q 3 , NF1 p.R2450* 2C.1 , NF1 p.E524* 2C.1 , PIK3CA p.T1025A 2C.1 , PIK3CA p.E542A 2C.1 , POLE p.P286H 2C.1 , SMAD4 p.E520* 2C.2 , SMAD4 p.E33* 2C.2 , SPEN p.N2002Kfs*2 3 , STAG2 p.R654I 3 | 1A.1 |  | NL |  | NL | 1A.1 |
| SP19582 | Colon/Rectum | COLON/RECTUM | ACVR1B 3 , APC p.R213* 2C.2 , APC p.S1403Mfs*13 2C.2 , FBXW7 p.R505C 2C.2 , RAD50 p.L1211F 2C.1 , TP53 p.Y236* 2C.2 | 2C.1 | CCND2 amp 2C.2 , FGF23 amp 3 | 2C.2 |  | NL | 2C.1 |
| SP19606 | Colon/Rectum | COLON/RECTUM | APC 2C.2 , APC p.S1355Lfs*60 2C.2 , FAM123B p.R497* 3 , GNAQ p.R181T 2C.2 , MTOR p.I2333M 2C.1 , NRAS p.Q61K 1.A.1 , SMAD4 p.V437D 2C.2 , TP53 p.F109C 2C.2 | 1.A.1 |  | NL |  | NL | 1.A.1 |
| SP19670 | Colon/Rectum | COLON/RECTUM | APC 2C.2 , APC p.H1490Ifs*17 2C.2 , ARID1A p.Q944* 2C.2 , KRAS p.K117N 1A.1 , SOX9 p.T478Hfs*100 3 , SOX9 p.F270L 3 , TP53 p.R196* 2C.2 | 1A.1 |  | NL |  | NL | 1A.1 |
| SP19750 | Colon/Rectum | COLON/RECTUM | APC 2C.2 , APC p.Q412* 2C.2 , TP53 p.P278R 2C.2 | 2C.2 | KLF5 amp 3 | 3 |  | NL | 2C.2 |
| SP19983 | Colon/Rectum | COLON/RECTUM | APC 2C.2 , KRAS p.G12D 1A.1 , PIK3CA p.E545K 2C.1 , SMAD4 p.C324R 2C.2 | 1A.1 | ERBB2 amp 2C.1 | 2C.1 |  | NL | 1A.1 |
| SP20993 | Colon/Rectum | COLON/RECTUM | APC 2C.2 , APC p.P1442Lfs*31 2C.2 , NF1 p.R1276Q 2C.1 , SPEN p.D485N 3 , TP53 p.V173M 2C.2 | 2C.1 |  | NL |  | NL | 2C.1 |
| SP21057 | Colon/Rectum | COLON/RECTUM | APC 2C.2 , APC p.L292Ffs*4 2C.2 , TP53 p.P301Qfs*44 2C.2 | 2C.2 |  | NL |  | NL | 2C.2 |
| SP21193 | Colon/Rectum | COLON/RECTUM | APC 2C.2 , FAM123B p.E480* 3 , KRAS p.G12V 1A.1 , TCF7L2 p.R471C 3 , TP53 p.L145R 2C.2 | 1A.1 | PTEN loss 2C.1 | 2C.1 |  | NL | 1A.1 |
| SP21400 | Colon/Rectum | COLON/RECTUM | ABCB1 3 , APC p.E1408* 2C.2 , APC p.S1281* 2C.2 , ARHGAP35 p.R744C 3 , ARID5B p.Y1099N 3 , ATM p.L2077I 2C.1 , ATM p.R1730* 2C.1 , AXIN2 p.F190V 2D , BLM p.R1144I 2C.1 , BRIP1 p.S624L 2C.1 , BTK p.Y334C 2C.2 , CBFB p.D171Y 3 , CDC73 p.R91Q 3 , CDH1 p.D291N 2D , CREBBP p.R1446H 2D , DPYD p.R561* 3 , EGFR p.R309Q 2C.1 , EP300 p.R1076C 2D , EP300 p.R580Q 2D , FANCD2 p.R1273* 2C.1 , FGFR1 p.S549L 2C.1 , HDAC9 p.R663Q 3 , KDM5C p.R679C 3 , KRAS p.G13D 1A.1 , MET p.F228C 2C.1 , MGA p.R1818* 3 , MLH3 p.E225* 2C.2 , MLL p.A1795T 3 , MLL2 p.R5448* 3 , MLL3 p.E4808K 3 , MRE11A p.G180D 2C.1 , MRE11A p.N617H 2C.1 , MSH2 p.E580* 1B , MSH6 p.E946* 1B , NFE2L2 p.K538T 3 , NT5C2 p.K361N 3 , PIK3CA p.Y1021C 2C.1 , PIK3CA p.R357Q 2C.1 , PIK3CA p.V344A 2C.1 , POLE p.V411L 2C.1 , RASA1 p.R709* 3 , RET p.P914S 2C.1 , SDHA p.F326L 2D , SDHA p.A236T 2D , SETD2 p.E138* 2D , SETD2 p.R1523C 2D , SETD2 p.R2122Q 2D , SMAD2 p.N307H 3 , SMARCA4 p.R1623W 2C.2 , SOS1 p.R248C 3 , SPEN p.K790N 3 , SPEN p.R438I 3 , SUFU p.R224W 2D , SUFU p.Y74H 2D , TCF7L2 p.K351N 3 , TSC1 p.L72R 2C.1 , TSC1 p.R228Q 2C.1 | 1A.1 | RAC1 loss 3 | 3 |  | NL | 1A.1 |
| SP21528 | Colon/Rectum | COLON/RECTUM | CTNNB1 2C.2 | 2C.2 |  | NL |  | NL | 2C.2 |
| SP22031 | Colon/Rectum | COLON/RECTUM | APC 2C.2 , APC p.L1522* 2C.2 , ATM p.R250* 2C.1 , BRCA2 p.R2494Q 2C.1 , CARD11 p.R1027L 3 , CDKN2C p.A110T 2D , DICER1 p.D1709N 3 , DICER1 p.A555T 3 , EP300 p.K1277N 2D , FAT1 p.R1627* 3 , GNAS p.R201H 2C.2 , KRAS p.A146T 1A.1 , MAP2K4 p.D197Y 2C.1 , MAP3K1 p.S1330L 2C.2 , MGA p.R2396* 3 , MRE11A p.K298N 2C.1 , MTOR p.S2215Y 2C.1 , PIK3CA p.M1043I 2C.1 , PIK3CA p.R88Q 2C.1 , PIK3R1 p.R348* 2C.2 , POLE p.S297F 2C.1 , RAD51C p.D254Y 2C.1 , SETD2 p.R529I 2D , SOX9 p.W115* 3 , TCF7L2 p.K351N 3 , TGFBR2 p.D471N 2D , TGFBR2 p.M148I 2D , TP53 p.P152L 2C.2 | 1A.1 |  | NL |  | NL | 1A.1 |
| SP22750 | Colon/Rectum | COLON/RECTUM | ARID1A 2C.2 , ARID1A p.R1722* 2C.2 , PPP2R1A p.R183W 2C.1 , SOX9 p.E190* 3 , TGFBR2 p.E453K 2D | 2C.1 |  | NL |  | NL | 2C.1 |
| SP23078 | Colon/Rectum | COLON/RECTUM | APC 2C.2 , APC p.A239Qfs*54 2C.2 , RUNX1 p.R201G 3 , TP53 p.R175H 2C.2 | 2C.2 | AURKA amp 3 , GNAS amp 2C.2 , PLCG1 amp 3 , SRC amp 2C.2 , TOP1 amp 2C.2 , ZNF217 amp 3 | 2C.2 |  | NL | 2C.2 |
| SP23639 | CNS | CNS |  | NL | CDKN2A loss 2C.1 , CDKN2B loss 2C.2 , EGFR amp 2C.1 , EGFR amp 2C.1 , MTAP loss 3 | 2C.1 | FGFR3 fusion 2C.1 | 2C.1 | 2C.1 |
| SP23925 | CNS | CNS | ARID2 3 , ATRX p.K358Tfs*3 2C.2 , IDH1 p.R132H 2C.1 | 2C.1 |  | NL |  | NL | 2C.1 |
| SP24236 | CNS | CNS | EGFR 2C.1 , RAD50 p.K545E 2C.1 , STAG2 p.T966K 3 | 2C.1 | CDKN2A loss 2C.1 , CDKN2B loss 2C.2 , EGFR amp 2C.1 | 2C.1 | EGFR fusion 2C.1 | 2C.1 | 2C.1 |
| SP24565 | CNS | CNS | PIK3CA 2C.1 , PTPN11 p.D61Y 2C.2 | 2C.1 | CDKN2A loss 2C.1 , CDKN2B loss 2C.2 , MTAP loss 3 | 2C.1 |  | NL | 2C.1 |
| SP24815 | CNS | CNS | PIK3CA 2C.1 | 2C.1 | CDK4 amp 2C.1 | 2C.1 |  | NL | 2C.1 |
| SP25350 | CNS | CNS | ATM 2C.1 , WT1 p.R458* 2C.2 | 2C.1 | CDKN2A loss 2C.1 , CDKN2B loss 2C.2 , EGFR amp 2C.1 , MTAP loss 3 , SMO amp 2C.1 | 2C.1 |  | NL | 2C.1 |
| SP25494 | CNS | CNS | ARID1B 2D , BCL6 p.A372T 3 , BLM p.G1132E 2C.1 , CDC73 p.G306S 3 , CREBBP p.D1481N 2D , EGFR p.A289V 2C.1 , ERBB4 p.P739S 2C.2 , ERCC4 p.G703D 2C.2 , ERCC4 p.A672S 2C.2 , ETV6 p.E13K 2C.2 , FANCC p.W403* 2C.1 , FAT1 p.R1340K 3 , FGFR1 p.D585N 2C.1 , HDAC9 p.V466I 3 , MLL3 p.G4207E 3 , MLL3 p.M4765I 3 , NOTCH1 p.D1958N 2C.2 , RPL5 p.P84L 3 , TP53 p.G266E 2C.2 , TSC2 p.D1631N 1A.1 | 1A.1 | CDKN2A loss 2C.1 , CDKN2B loss 2C.2 , EGFR amp 2C.1 , EGFR amp 2C.1 , EGFR amp 2C.1 | 2C.1 |  | NL | 1A.1 |
| SP25518 | CNS | CNS | TP53 2C.2 | 2C.2 | CDKN2A loss 2C.1 , CDKN2B loss 2C.2 , EGFR amp 2C.1 , MTAP loss 3 | 2C.1 | EGFR fusion 2C.1 | 2C.1 | 2C.1 |
| SP25833 | CNS | CNS |  | NL | CDKN2A loss 2C.1 , CDKN2B loss 2C.2 , EGFR amp 2C.1 , EGFR amp 2C.1 , MTAP loss 3 | 2C.1 |  | NL | 2C.1 |
| SP25905 | CNS | CNS | EGFR 2C.1 | 2C.1 | CDK6 amp 2C.1 , CDKN2A loss 2C.1 , CDKN2B loss 2C.2 , EGFR amp 2C.1 , MTAP loss 3 | 2C.1 | FGFR3 fusion 2C.1 | 2C.1 | 2C.1 |
| SP26439 | CNS | CNS | ATRX 2C.2 , IDH1 p.R132H 2C.1 , TP53 p.R273H 2C.2 | 2C.1 | FGFR2 amp 2C.1 | 2C.1 | FGFR3 fusion 2C.1 | 2C.1 | 2C.1 |
| SP26475 | CNS | CNS |  | NL |  | NL |  | NL | NL |
| SP26499 | CNS | CNS | NF1 2C.1 , PIK3CB p.E1051K 2C.1 | 2C.1 |  | NL | FGFR3 fusion 2C.1 | 2C.1 | 2C.1 |
| SP26649 | CNS | CNS |  | NL | AKT1 amp 2C.2 , CDKN2A loss 2C.1 , CDKN2B loss 2C.2 , EGFR amp 2C.1 , EGFR amp 2C.1 , MTAP loss 3 | 2C.1 |  | NL | 2C.1 |
| SP26709 | CNS | CNS | PTEN 2C.1 | 2C.1 | CDKN2A loss 2C.1 , CDKN2C loss 2D , MDM4 amp 2D , PIK3C2B amp 3 | 2C.1 | NTRK1 fusion 1A.1 | 1A.1 | 1A.1 |
| SP2714 | Breast | BREAST | CDKN2A 2C.1 , TP53 p.G108Tfs*39 2C.2 , TP53 p.G108Afs*39 2C.2 | 2C.1 |  | NL |  | NL | 2C.1 |
| SP27201 | CNS | CNS | EGFR 2C.1 , PIK3CA p.E103G 2C.1 , TP53 p.R248Q 2C.2 , TP53 p.S127Y 2C.2 | 2C.1 | AKT3 amp 2C.2 , CDK4 amp 2C.1 , EGFR amp 2C.1 , KIT amp 2C.1 , KIT amp 2C.1 , PDGFRA amp 2C.1 | 2C.1 |  | NL | 2C.1 |
| SP2731 | Breast | BREAST | GATA3 2D | 2D | H3F3B amp 3 , RPS6KB1 amp 3 , RPS6KB1 amp 3 | 3 |  | NL | 2D |
| SP27339 | CNS | CNS | EGFR 2C.1 | 2C.1 | CCND2 amp 2C.2 , CDK4 amp 2C.1 , EGFR amp 2C.1 , MDM4 amp 2D , PIK3C2B amp 3 , PIK3C2B amp 3 | 2C.1 |  | NL | 2C.1 |
| SP27603 | CNS | CNS | EGFR 2C.1 , PTPN11 p.Q510H 2C.2 | 2C.1 | CDKN2A loss 2C.1 , CDKN2B loss 2C.2 , EGFR amp 2C.1 , EGFR amp 2C.1 , MTAP loss 3 | 2C.1 |  | NL | 2C.1 |
| SP2766 | Breast | BREAST | BAP1 2C.1 , TP53 p.C242Afs*5 2C.2 | 2C.1 |  | NL |  | NL | 2C.1 |
| SP2781 | Breast | BREAST | CDH1 2D , PIK3CA p.H1047R 1A.1 | 1A.1 | CCND1 amp 2C.2 , FGF19 amp 2D , FGF3 amp 2D , FGF4 amp 2D , IKBKB amp 3 | 2C.2 |  | NL | 1A.1 |
| SP2793 | Breast | BREAST | BRCA2 1A.1 , CDKN2A p.R80Pfs*71 2C.1 | 1A.1 | FYN amp 3 | 3 |  | NL | 1A.1 |
| SP27957 | CNS | CNS | EGFR 2C.1 , RB1 p.L779* 2C.2 , TP53 p.R267P 2C.2 | 2C.1 | EGFR amp 2C.1 , EGFR amp 2C.1 , EGFR amp 2C.1 , EGFR amp 2C.1 , GLI3 amp 3 , GLI3 amp 3 | 2C.1 |  | NL | 2C.1 |
| SP2799 | Breast | BREAST | ARID1B 2D , CDK12 p.N939S 2C.1 , PIK3CA p.H1047R 1A.1 | 1A.1 |  | NL |  | NL | 1A.1 |
| SP2801 | Breast | BREAST |  | NL | CCND1 amp 2C.2 , CDKN2A loss 2C.1 , CDKN2B loss 2D , FGF19 amp 2D , FGF3 amp 2D , FGF4 amp 2D , MAGOH amp 3 , MDM4 amp 2D , MTAP loss 3 , PIK3C2B amp 3 | 2C.1 |  | NL | 2C.1 |
| SP2826 | Breast | BREAST | TP53 2C.2 | 2C.2 | MCL1 amp 2D , RIT1 amp 3 | 2D |  | NL | 2C.2 |
| SP28275 | CNS | CNS | CDKN2A 2C.1 , DICER1 p.E1861K 3 | 2C.1 | CDK6 amp 2C.1 , CDK6 amp 2C.1 , EGFR amp 2C.1 | 2C.1 |  | NL | 2C.1 |
| SP28581 | CNS | CNS | RB1 2C.2 | 2C.2 | CCND2 amp 2C.2 , EGFR amp 2C.1 , EGFR amp 2C.1 , MDM2 amp 2C.2 | 2C.1 |  | NL | 2C.1 |
| SP28791 | CNS | CNS | ATRX 2C.2 , IDH1 p.R132H 2C.1 , PIK3CA p.E542V 2C.1 , TP53 p.Q136E 2C.2 | 2C.1 | CDK4 amp 2C.1 | 2C.1 |  | NL | 2C.1 |
| SP2881 | Breast | BREAST | MLL2 3 , TP53 p.S215I 2C.2 | 2C.2 | ABL2 amp 3 , DDR2 amp 2C.2 , H3F3A amp 2C.2 , MCL1 amp 2D , MDM4 amp 2D , NRAS amp 2C.1 , NTRK1 amp 2C.1 , PIK3C2B amp 3 , RIT1 amp 3 | 2C.1 |  | NL | 2C.1 |
| SP29331 | CNS | CNS | PTEN 2C.1 , TP53 p.R248Q 2C.2 | 2C.1 | CDKN2A loss 2C.1 , CDKN2B loss 2C.2 , MDM4 amp 2D , MTAP loss 3 , PDGFRA amp 2C.1 , PIK3C2B amp 3 | 2C.1 |  | NL | 2C.1 |
| SP29697 | CNS | CNS |  | NL | CDK4 amp 2C.1 , CDK4 amp 2C.1 , EGFR amp 2C.1 , EGFR amp 2C.1 , EGFR amp 2C.1 , EGFR amp 2C.1 , EGFR amp 2C.1 , EGFR amp 2C.1 , EGFR amp 2C.1 , MDM2 amp 2C.2 , MDM2 amp 2C.2 , MDM2 amp 2C.2 , MDM2 amp 2C.2 | 2C.1 | EGFR fusion 2C.1 | 2C.1 | 2C.1 |
| SP29940 | Head/Neck | OTHER | CTCF 3 , HRAS p.G12S 2C.1 , PPP2R1A p.R183Q 2C.1 | 2C.1 | CDKN2A loss 2C.1 , CDKN2B loss 2C.2 , MTAP loss 3 | 2C.1 |  | NL | 2C.1 |
| SP2997 | Breast | BREAST | NOTCH1 2C.2 , TP53 p.R175H 2C.2 | 2C.2 | ABL2 amp 3 , DDR2 amp 2C.2 , MCL1 amp 2D , NTRK1 amp 2C.1 , RIT1 amp 3 | 2C.1 |  | NL | 2C.1 |
| SP29987 | Head/Neck | OTHER | HDAC9 3 , MLL3 p.D2755H 3 , TP53 p.M237V 2C.2 , TP53 p.H179R 2C.2 | 2C.2 | CCND1 amp 2C.2 , FGF19 amp 2D , FGF3 amp 2D , FGF4 amp 2D | 2C.2 |  | NL | 2C.2 |
| SP30011 | Head/Neck | OTHER |  | NL |  | NL |  | NL | NL |
| SP30071 | Head/Neck | OTHER | ATRX 2C.2 , CASP8 p.S434* 2D , HRAS p.G13R 2C.1 | 2C.1 |  | NL |  | NL | 2C.1 |
| SP30077 | Head/Neck | OTHER | BRCA1 2C.1 , CDKN2A p.Y129* 2C.1 , TP53 p.R175H 2C.2 | 2C.1 | CCND1 amp 2C.2 , EGFR amp 2C.1 , EGFR amp 2C.1 , FGF19 amp 2D , FGF3 amp 2D , FGF4 amp 2D , NFE2L2 amp 3 | 2C.1 |  | NL | 2C.1 |
| SP30083 | Head/Neck | OTHER | CDKN2A 2C.1 , RUNX1 p.S322Ffs*232 2C.2 , TP53 p.V272L 2C.2 , TP53 p.R273H 2C.2 | 2C.1 |  | NL |  | NL | 2C.1 |
| SP30093 | Head/Neck | OTHER | CASP8 2D , CDKN2A p.R80* 2C.1 , EPHA2 p.R465* 2C.2 , NOTCH1 p.C381Vfs*250 2C.2 , TP53 p.P177_C182delPHHERC 2C.2 , TP53 p.H193L 2C.2 | 2C.1 | CCND2 amp 2C.2 , FGF23 amp 3 , IGF1R amp 2D , MAPK1 amp 2C.2 , MAPK1 amp 2C.2 | 2C.2 |  | NL | 2C.1 |
| SP30113 | Head/Neck | OTHER | ARHGAP35 3 , CDKN2A p.E61* 2C.1 , CREBBP p.V1634L 2C.2 , CUL3 p.L321F 3 , EPHA2 p.E572* 2C.2 , KIT p.N486I 2C.1 , PRDM1 p.K188M 3 , TP53 p.R156P 2C.2 , TP53 p.C238F 2C.2 | 2C.1 | YES1 amp 3 | 3 |  | NL | 2C.1 |
| SP30143 | Head/Neck | OTHER | NOTCH1 2C.2 | 2C.2 |  | NL |  | NL | 2C.2 |
| SP3016 | Breast | BREAST | TP53 2C.2 | 2C.2 | BCL2L12 amp 3 , MYC amp 2C.2 , RIT1 amp 3 , STAT3 amp 2C.2 | 2C.2 |  | NL | 2C.2 |
| SP30185 | Head/Neck | OTHER | PIK3CA 2C.1 | 2C.1 |  | NL |  | NL | 2C.1 |
| SP30213 | Head/Neck | OTHER | B2M 2C.2 | 2C.2 |  | NL |  | NL | 2C.2 |
| SP30332 | Head/Neck | OTHER | CDKN2A 2C.1 , MLH1 p.P138R 1B | 1B | ADAMTS2 loss 3 , AKT1 amp 2C.2 , BCL6 amp 2C.2 , BRAF amp 2C.1 , CCND2 amp 2C.2 , CHD4 amp 3 , EGFR amp 2C.1 , ERBB2 amp 2C.1 , EZH2 amp 2C.2 , FGF23 amp 3 , FOXA1 amp 3 , GATA2 amp 2C.2 , GLI3 amp 3 , IL7R amp 3 , KRAS amp 2C.1 , MAPK1 amp 2C.2 , MAX amp 3 , MECOM amp 3 , MITF amp 3 , MITF amp 3 , PIK3CA amp 2C.1 , PIK3CB amp 2C.1 , PXDNL amp 3 , RHEB amp 3 , RICTOR amp 2C.2 , SLCO1B3 amp 3 , SMO amp 2C.1 , YES1 amp 3 | 2C.1 | ERBB2 fusion 2C.1 | 2C.1 | 1B |
| SP30493 | Head/Neck | OTHER | PIK3CA 2C.1 | 2C.1 |  | NL |  | NL | 2C.1 |
| SP30675 | Head/Neck | OTHER | MLH3 2C.2 , MLL2 p.S654Pfs*276 3 , NOTCH1 p.G481C 2C.2 , TP53 p.V73Rfs*76 2C.2 , TP53 p.Q136P 2C.2 | 2C.2 | CCND1 amp 2C.2 , CDKN2A loss 2C.1 , CDKN2B loss 2C.2 , EGFR amp 2C.1 , FGF19 amp 2D , FGF3 amp 2D , FGF4 amp 2D , MAX amp 3 | 2C.1 |  | NL | 2C.1 |
| SP30803 | Head/Neck | OTHER | RUNX1 2C.2 , STK11 p.D194N 2C.1 , STK11 p.G268R 2C.1 , STK11 p.E265Rfs*22 2C.1 , TGFBR2 p.S175* 2D | 2C.1 |  | NL |  | NL | 2C.1 |
| SP30843 | Head/Neck | OTHER |  | NL |  | NL |  | NL | NL |
| SP30907 | Head/Neck | OTHER | ARAF 2C.1 , KDM6A p.R519* 2C.2 , TP53 p.V274dupV 2C.2 | 2C.1 | CCND1 amp 2C.2 , CDKN2A loss 2C.1 , CDKN2B loss 2C.2 , FGF19 amp 2D , FGF3 amp 2D , FGF4 amp 2D , YAP1 amp 3 | 2C.1 |  | NL | 2C.1 |
| SP31030 | Head/Neck | OTHER | ARID1B 2D , JAK3 p.W709C 2C.2 , KDM5C p.D337N 3 , PAX5 p.A100T 3 , PIK3CA p.E545K 2C.1 | 2C.1 |  | NL |  | NL | 2C.1 |
| SP31046 | Head/Neck | OTHER | CYLD 3 , EP300 p.D1399N 2C.2 | 2C.2 | PTEN loss 2C.1 | 2C.1 |  | NL | 2C.1 |
| SP31126 | Head/Neck | OTHER | TP53 2C.2 | 2C.2 | FLT3 amp 2C.1 , KLF5 amp 3 | 2C.1 |  | NL | 2C.1 |
| SP31174 | Head/Neck | OTHER | CDKN2A 2C.1 , TP53 p.R248W 2C.2 | 2C.1 | EGFR amp 2C.1 , KLF5 amp 3 | 2C.1 |  | NL | 2C.1 |
| SP31190 | Head/Neck | OTHER | CDKN2A 2C.1 , CTCF p.K649* 3 , TP53 p.K101* 2C.2 | 2C.1 | IGF1R amp 2D , YAP1 amp 3 , YAP1 amp 3 , YAP1 amp 3 , YAP1 amp 3 | 2D |  | NL | 2C.1 |
| SP31334 | Head/Neck | OTHER | ATR 2C.2 , CUL3 p.E206Q 3 , FAT1 p.R1262* 3 , NF1 p.S1448R 2C.1 , NOTCH1 p.C739* 2C.2 , NOTCH2 p.D341Y 2C.2 , TP53 p.T256Hfs*8 2C.2 | 2C.1 | CCND1 amp 2C.2 , CDKN2A loss 2C.1 , CDKN2B loss 2C.2 , EIF1AX amp 3 , FGF19 amp 2D , FGF3 amp 2D , FGF4 amp 2D , MECOM amp 3 , PIK3CA amp 2C.1 , TPMT amp 3 | 2C.1 |  | NL | 2C.1 |
| SP31606 | Head/Neck | OTHER | HLA-A 2C.2 | 2C.2 |  | NL |  | NL | 2C.2 |
| SP31790 | Head/Neck | OTHER | CDKN2A 2C.1 , HLA-B p.V309Gfs*24 3 , NOTCH1 p.R1984* 2C.2 , TP53 p.R282W 2C.2 | 2C.1 | YAP1 amp 3 , YAP1 amp 3 , YAP1 amp 3 | 3 |  | NL | 2C.1 |
| SP31814 | Head/Neck | OTHER | NF2 2C.1 , PIK3CA p.E545K 2C.1 , TP53 p.H193L 2C.2 | 2C.1 | CDKN2A loss 2C.1 , CDKN2B loss 2C.2 | 2C.1 |  | NL | 2C.1 |
| SP31854 | Head/Neck | OTHER | MLL2 3 , TP53 p.E294Sfs*51 2C.2 | 2C.2 | CCND1 amp 2C.2 , FGF19 amp 2D , FGF3 amp 2D , FGF4 amp 2D , YAP1 amp 3 | 2C.2 |  | NL | 2C.2 |
| SP32014 | Head/Neck | OTHER | TP53 2C.2 | 2C.2 | PRKACA amp 3 | 3 |  | NL | 2C.2 |
| SP32222 | Head/Neck | OTHER |  | NL |  | NL |  | NL | NL |
| SP32662 | Head/Neck | OTHER | PIK3CA 2C.1 , TP53 p.R175H 2C.2 , TP53 p.R283P 2C.2 | 2C.1 | CCND1 amp 2C.2 , FGF19 amp 2D , FGF19 amp 2D , FGF3 amp 2D , FGF4 amp 2D | 2C.2 |  | NL | 2C.1 |
| SP32694 | Head/Neck | OTHER | NOTCH1 2C.2 , RHOA p.E40Q 3 | 2C.2 | CCND1 amp 2C.2 , FGF19 amp 2D , FGF3 amp 2D , FGF4 amp 2D | 2C.2 |  | NL | 2C.2 |
| SP32742 | Head/Neck | OTHER | FUBP1 3 , GATA3 p.S408W 2D , MGA p.L674V 3 | 2D |  | NL |  | NL | 2D |
| SP32894 | Head/Neck | OTHER | EP300 2C.2 | 2C.2 |  | NL | FGFR3 fusion 2C.1 | 2C.1 | 2C.1 |
| SP32958 | Head/Neck | OTHER | EP300 2C.2 , MLL2 p.Q56* 3 , MLL2 p.S1632* 3 , PIK3CA p.E545K 2C.1 | 2C.1 | BCL6 amp 2C.2 , MECOM amp 3 , PIK3CA amp 2C.1 | 2C.1 |  | NL | 2C.1 |
| SP33094 | Head/Neck | OTHER | TP53 2C.2 | 2C.2 | ABCB1 amp 3 , CCND1 amp 2C.2 , CDK6 amp 2C.1 , CDKN2A loss 2C.1 , CDKN2B loss 2C.2 , EGFR amp 2C.1 , ERBB2 amp 2C.1 , FGF19 amp 2D , FGF3 amp 2D , FGF4 amp 2D , MET amp 2C.1 , MTAP loss 3 | 2C.1 |  | NL | 2C.1 |
| SP33496 | Head/Neck | OTHER | ATRX 2C.2 , MLL2 p.G1916Vfs*129 3 , NOTCH1 p.E1305K 2C.2 , TP53 p.P278S 2C.2 , TP53 p.Y205*fs*1 2C.2 | 2C.2 | CCND1 amp 2C.2 , CCND1 amp 2C.2 , CDKN2A loss 2C.1 , CDKN2B loss 2C.2 , FGF19 amp 2D , FGF3 amp 2D , FGF4 amp 2D | 2C.1 |  | NL | 2C.1 |
| SP33544 | Head/Neck | OTHER | FAT1 3 , PIK3CA p.K111N 2C.1 , POLE p.E537G 2C.1 , SPEN p.Y626C 3 , TP53 p.R110Wfs*12 2C.2 | 2C.1 | TGFBR2 loss 2D | 2D |  | NL | 2C.1 |
| SP3368 | Breast | BREAST | TP53 2C.2 , TP53 p.L114* 2C.2 | 2C.2 |  | NL |  | NL | 2C.2 |
| SP33688 | Head/Neck | OTHER | DDR2 2C.2 , PIK3CA p.E545K 2C.1 , RET p.T946A 2C.1 | 2C.1 |  | NL |  | NL | 2C.1 |
| SP33774 | Head/Neck | OTHER | AKT1 2C.2 | 2C.2 |  | NL |  | NL | 2C.2 |
| SP33837 | Head/Neck | OTHER |  | NL | FAM135B amp 3 , MYC amp 2C.2 | 2C.2 |  | NL | 2C.2 |
| SP34005 | Head/Neck | OTHER | RAC1 3 | 3 |  | NL |  | NL | 3 |
| SP3415 | Breast | BREAST | BRCA1 1A.1 , TP53 p.R282W 2C.2 | 1A.1 | MCL1 amp 2D , MYC amp 2C.2 , NTRK1 amp 2C.1 , RIT1 amp 3 | 2C.1 |  | NL | 1A.1 |
| SP34186 | Kidney | OTHER | PTEN 2C.1 , SETD2 p.P1822Qfs*16 2D , VHL p.P172Rfs*29 2C.2 | 2C.1 |  | NL |  | NL | 2C.1 |
| SP34191 | Kidney | OTHER | AKT2 2C.2 , PBRM1 p.V194Cfs*11 2D , SETD2 p.E1720* 2D | 2C.2 |  | NL |  | NL | 2C.2 |
| SP34246 | Kidney | OTHER | ATM 2C.1 , VHL p.L158R 2C.2 | 2C.1 |  | NL |  | NL | 2C.1 |
| SP34431 | Kidney | OTHER | VHL 2C.2 | 2C.2 |  | NL |  | NL | 2C.2 |
| SP34452 | Kidney | OTHER | MAP3K4 3 , VHL p.T124Hfs*35 2C.2 | 2C.2 |  | NL |  | NL | 2C.2 |
| SP34493 | Kidney | OTHER |  | NL |  | NL |  | NL | NL |
| SP35412 | Kidney | OTHER | KDM5C 3 , PBRM1 p.P1384Rfs*48 2D , VHL p.L118P 2C.2 | 2C.2 |  | NL |  | NL | 2C.2 |
| SP35617 | Kidney | OTHER | PIK3CA 2C.1 , VHL p.Q145* 2C.2 | 2C.1 |  | NL |  | NL | 2C.1 |
| SP35849 | Kidney | OTHER | AXIN2 2D , VHL p.L158R 2C.2 | 2C.2 |  | NL |  | NL | 2C.2 |
| SP35951 | Kidney | OTHER | MGA 3 , PBRM1 . 2D , PBRM1 p.V1478Afs*11 2D , VHL p.K159* 2C.2 | 2C.2 |  | NL |  | NL | 2C.2 |
| SP35989 | Kidney | OTHER | VHL 2C.2 | 2C.2 |  | NL |  | NL | 2C.2 |
| SP36036 | Kidney | OTHER | CDK12 2C.1 , PBRM1 p.E160* 2D , RNF43 p.M173T 2C.2 , VHL p.V74G 2C.2 | 2C.1 |  | NL |  | NL | 2C.1 |
| SP36218 | Kidney | OTHER | VHL 2C.2 | 2C.2 |  | NL |  | NL | 2C.2 |
| SP3631 | Breast | BREAST | ARID2 3 , B2M p.R3C 2C.2 , PBRM1 p.R394S 2D , SETD2 p.S180* 2D , SPEN p.E2583* 3 , TET2 p.Q1603* 2D , TP53 p.Y205C 2C.2 | 2C.2 | CDK4 amp 2C.1 , ERBB2 amp 1A.1 , GNAS amp 2C.2 , ZNF217 amp 3 | 1A.1 |  | NL | 1A.1 |
| SP36498 | Kidney | OTHER | PBRM1 2D , VHL p.E94_P97delinsD 2C.2 | 2C.2 |  | NL |  | NL | 2C.2 |
| SP36586 | Kidney | OTHER |  | NL |  | NL |  | NL | NL |
| SP37369 | Kidney | OTHER | MEN1 2C.2 , PBRM1 p.L617Ffs*25 2D , VHL p.I147Yfs*27 2C.2 | 2C.2 |  | NL |  | NL | 2C.2 |
| SP37432 | Kidney | OTHER | KDR 2D , PTEN p.K197Qfs*5 2C.1 , STAG2 p.R216Q 3 , VHL p.T202Dfs*54 2C.2 | 2C.1 |  | NL |  | NL | 2C.1 |
| SP37516 | Kidney | OTHER | ATM 2C.1 | 2C.1 |  | NL |  | NL | 2C.1 |
| SP37636 | Kidney | OTHER | SETD2 2D , VHL p.Q203Gfs*52 2C.2 | 2C.2 | KDM5C loss 3 | 3 |  | NL | 2C.2 |
| SP37903 | Kidney | OTHER | POLE 2C.1 | 2C.1 |  | NL |  | NL | 2C.1 |
| SP37970 | Kidney | OTHER | NBN 2C.1 , PBRM1 p.R332Vfs*30 2D , VHL p.E173* 2C.2 | 2C.1 |  | NL |  | NL | 2C.1 |
| SP38044 | Kidney | OTHER | MAP3K1 2C.2 , SETD2 p.L2124* 2D , VHL p.N78S 2C.2 | 2C.2 |  | NL |  | NL | 2C.2 |
| SP38271 | Kidney | OTHER | MLL3 3 | 3 |  | NL |  | NL | 3 |
| SP38759 | Kidney | OTHER | ERCC4 2C.2 | 2C.2 |  | NL |  | NL | 2C.2 |
| SP39102 | Kidney | OTHER | SETD2 2D , SMARCB1 p.Q368* 2D , VHL p.W117Gfs*42 2C.2 | 2C.2 |  | NL |  | NL | 2C.2 |
| SP39248 | Kidney | OTHER | PBRM1 2D | 2D |  | NL |  | NL | 2D |
| SP39298 | Kidney | OTHER | ACO1 3 | 3 |  | NL |  | NL | 3 |
| SP39349 | Kidney | OTHER | MTOR 2C.1 , PBRM1 p.V44Cfs*9 2D , VHL p.L188P 2C.2 | 2C.1 |  | NL |  | NL | 2C.1 |
| SP39594 | Kidney | OTHER | PTPRB 3 , VHL p.N78S 2C.2 | 2C.2 |  | NL |  | NL | 2C.2 |
| SP39907 | Kidney | OTHER |  | NL |  | NL |  | NL | NL |
| SP39997 | Kidney | OTHER |  | NL |  | NL |  | NL | NL |
| SP40047 | Kidney | OTHER | PBRM1 2D , VHL p.P192Qfs*10 2C.2 | 2C.2 |  | NL |  | NL | 2C.2 |
| SP40736 | Kidney | OTHER | PBRM1 2D , VHL p.N131Tfs*28 2C.2 | 2C.2 |  | NL |  | NL | 2C.2 |
| SP41453 | Kidney | OTHER |  | NL |  | NL |  | NL | NL |
| SP42154 | Kidney | OTHER | ELF3 3 , MET p.V1088E 2C.1 | 2C.1 | CDKN2A loss 2C.1 , MTAP loss 3 | 2C.1 |  | NL | 2C.1 |
| SP4265 | Breast | BREAST | HLA-A 2C.2 , TP53 p.R196* 2C.2 | 2C.2 | PTEN loss 2C.1 | 2C.1 | RAD51B fusion 2C.1 | 2C.1 | 2C.1 |
| SP42829 | Kidney | OTHER | NF1 2C.1 , SETD2 p.P230Tfs*7 2D , VHL p.Q73_F76delinsL 2C.2 | 2C.1 |  | NL |  | NL | 2C.1 |
| SP43201 | Kidney | OTHER |  | NL |  | NL |  | NL | NL |
| SP43510 | Kidney | OTHER |  | NL |  | NL |  | NL | NL |
| SP43514 | Kidney | OTHER |  | NL |  | NL |  | NL | NL |
| SP43532 | Kidney | OTHER |  | NL |  | NL |  | NL | NL |
| SP43664 | Kidney | OTHER | FAT1 3 | 3 | CDK6 amp 2C.1 , MET amp 2C.1 , SMO amp 2C.1 | 2C.1 |  | NL | 2C.1 |
| SP43688 | Kidney | OTHER | GNA13 3 | 3 |  | NL |  | NL | 3 |
| SP43696 | Kidney | OTHER | RAF1 2C.2 | 2C.2 |  | NL |  | NL | 2C.2 |
| SP43770 | Kidney | OTHER | BAP1 2C.1 , VHL p.W117Lfs*15 2C.2 | 2C.1 |  | NL |  | NL | 2C.1 |
| SP43792 | Kidney | OTHER | MLL3 3 | 3 |  | NL |  | NL | 3 |
| SP43808 | Kidney | OTHER | MET 2C.1 | 2C.1 |  | NL |  | NL | 2C.1 |
| SP43822 | Kidney | OTHER |  | NL | CDKN2A loss 2C.1 , CDKN2B loss 2C.2 , ERBB2 amp 2C.1 , FGFR4 amp 2C.1 , FLT4 amp 2C.2 , H3F3B amp 3 , MTAP loss 3 , RARA amp 3 , RPTOR amp 3 , RPTOR amp 3 , STAT3 amp 2C.2 | 2C.1 |  | NL | 2C.1 |
| SP4472 | Breast | BREAST | PIK3CA 1A.1 , TP53 p.C238F 2C.2 | 1A.1 | CCND1 amp 2C.2 , FGF19 amp 2D , FGF3 amp 2D , FGF4 amp 2D , H3F3A amp 2C.2 | 2C.2 |  | NL | 1A.1 |
| SP4523 | Breast | BREAST | MEN1 2C.2 | 2C.2 | CCND1 amp 2C.2 , CCND1 amp 2C.2 , ERBB2 amp 1A.1 , FGF19 amp 2D , FGF3 amp 2D , FGF4 amp 2D , RPS6KB1 amp 3 , RPS6KB1 amp 3 , RPS6KB1 amp 3 | 1A.1 | ESR1 fusion 2C.1 , NF1 fusion 2C.1 | 2C.1 | 1A.1 |
| SP4535 | Breast | BREAST | MAP3K1 2C.2 | 2C.2 |  | NL |  | NL | 2C.2 |
| SP4557 | Breast | BREAST | FAT1 3 , FOXA1 p.F266S 2C.2 , PIK3CA p.H1047R 1A.1 | 1A.1 |  | NL |  | NL | 1A.1 |
| SP4593 | Breast | BREAST | ARID1A 2C.2 | 2C.2 |  | NL |  | NL | 2C.2 |
| SP47628 | CNS | CNS | IDH1 2C.1 , TP53 p.R213* 2C.2 , TP53 p.R282W 2C.2 | 2C.1 |  | NL |  | NL | 2C.1 |
| SP47652 | CNS | CNS | ARID1A 2C.2 , IDH1 p.R132H 2C.1 | 2C.1 |  | NL |  | NL | 2C.1 |
| SP47708 | CNS | CNS | CIC 3 , CIC p.W238R 3 , IDH1 p.R132H 2C.1 | 2C.1 |  | NL |  | NL | 2C.1 |
| SP47808 | CNS | CNS | BCOR 3 , CIC p.V778Pfs*155 3 , IDH2 p.R172M 2C.1 , NOTCH1 p.C129Y 2C.2 | 2C.1 |  | NL |  | NL | 2C.1 |
| SP47990 | CNS | CNS | ATRX 2C.2 , IDH1 p.R132H 2C.1 , TP53 p.E339* 2C.2 | 2C.1 |  | NL |  | NL | 2C.1 |
| SP48008 | CNS | CNS | CIC 3 , IDH1 p.R132H 2C.1 | 2C.1 | CDKN2A loss 2C.1 | 2C.1 |  | NL | 2C.1 |
| SP48010 | CNS | CNS | ATRX 2C.2 , IDH1 p.R132H 2C.1 , TP53 p.F109V 2C.2 | 2C.1 |  | NL |  | NL | 2C.1 |
| SP48073 | CNS | CNS | CIC 3 , FUBP1 p.Y489*fs*1 3 , IDH1 p.R132H 2C.1 | 2C.1 |  | NL |  | NL | 2C.1 |
| SP48135 | CNS | CNS | PTEN 2C.1 , PTPN11 p.A72D 2C.2 | 2C.1 | CDKN2A loss 2C.1 , CDKN2B loss 2C.2 , MDM4 amp 2D , MTAP loss 3 , PIK3C2B amp 3 | 2C.1 |  | NL | 2C.1 |
| SP48189 | CNS | CNS | ATRX 2C.2 , CHEK2 p.A480T 2C.1 , IDH1 p.R132H 2C.1 , TP53 p.Q136E 2C.2 | 2C.1 |  | NL |  | NL | 2C.1 |
| SP4820 | Breast | BREAST | RNF43 2C.2 | 2C.2 | BRCA2 loss 2C.1 , RB1 loss 2C.2 | 2C.1 |  | NL | 2C.1 |
| SP48263 | CNS | CNS | CIC 3 , IDH1 p.R132H 2C.1 | 2C.1 | FUBP1 loss 3 | 3 |  | NL | 2C.1 |
| SP48414 | CNS | CNS | ATRX 2C.2 , IDH1 p.R132S 2C.1 , TP53 p.R337C 2C.2 | 2C.1 | TP53 loss 2C.2 | 2C.2 |  | NL | 2C.1 |
| SP48426 | CNS | CNS | CIC 3 , IDH1 p.R132H 2C.1 | 2C.1 |  | NL |  | NL | 2C.1 |
| SP48480 | CNS | CNS | FUBP1 3 , IDH1 p.R132H 2C.1 | 2C.1 |  | NL |  | NL | 2C.1 |
| SP48504 | CNS | CNS | CIC 3 , CIC p.S146*fs*1 3 , FUBP1 p.I136V 3 , IDH1 p.R132H 2C.1 | 2C.1 |  | NL |  | NL | 2C.1 |
| SP48534 | CNS | CNS | CIC 3 , IDH1 p.R132H 2C.1 | 2C.1 |  | NL |  | NL | 2C.1 |
| SP4875 | Breast | BREAST | TP53 2C.2 | 2C.2 | AURKA amp 3 , GNAS amp 2C.2 , PTEN loss 2C.1 , ZNF217 amp 3 | 2C.1 |  | NL | 2C.1 |
| SP48850 | CNS | CNS | ATRX 2C.2 , IDH1 p.R132H 2C.1 , TP53 p.R273C 2C.2 | 2C.1 | CDK4 amp 2C.1 | 2C.1 |  | NL | 2C.1 |
| SP48888 | CNS | CNS | IDH1 2C.1 | 2C.1 |  | NL |  | NL | 2C.1 |
| SP49114 | Liver | OTHER | HNF1A 2C.2 , HNF1A p.E48Pfs*106 2C.2 , TP53 p.Y126D 2C.2 | 2C.2 |  | NL |  | NL | 2C.2 |
| SP49119 | Liver | OTHER | AXIN1 2C.2 , AXIN1 p.S334Pfs*16 2C.2 | 2C.2 | FAM135B amp 3 , MYC amp 2C.2 , PXDNL amp 3 | 2C.2 |  | NL | 2C.2 |
| SP49124 | Liver | OTHER | AXIN2 2D , ERCC2 p.T484A 2C.2 , IL6ST p.P216H 3 | 2C.2 |  | NL |  | NL | 2C.2 |
| SP49157 | Liver | OTHER | CTNNB1 2C.2 , EP300 p.H1255L 2C.2 , SF3B1 p.K666T 3 | 2C.2 |  | NL |  | NL | 2C.2 |
| SP49175 | Liver | OTHER | ASXL1 2C.2 , BAP1 p.V569Cfs*2 2C.1 , IDH1 p.R132C 2C.1 | 2C.1 |  | NL |  | NL | 2C.1 |
| SP49187 | Liver | OTHER | MAP2K4 2C.1 , PMS1 p.E480Kfs*36 2C.2 | 2C.1 |  | NL |  | NL | 2C.1 |
| SP49205 | Liver | OTHER |  | NL | EPHA2 loss 2C.2 | 2C.2 |  | NL | 2C.2 |
| SP49223 | Liver | OTHER | BAP1 2C.1 , IDH2 p.R172S 2C.1 , NOTCH2 p.G254R 2C.2 | 2C.1 |  | NL |  | NL | 2C.1 |
| SP49229 | Liver | OTHER | IDH1 2C.1 , PBRM1 p.M1184Nfs*11 2D , RAF1 p.P261R 2C.2 | 2C.1 |  | NL |  | NL | 2C.1 |
| SP49247 | Liver | OTHER | TP53 2C.2 | 2C.2 | MCL1 amp 2D | 2D |  | NL | 2C.2 |
| SP49286 | Liver | OTHER | DPYD 3 , NFE2L2 p.W24_D29delinsY 3 , SETD2 p.T2118I 2D , TP53 p.H193R 2C.2 | 2C.2 | CDKN2A loss 2C.1 , CDKN2B loss 2C.2 , MTAP loss 3 | 2C.1 |  | NL | 2C.1 |
| SP49322 | Liver | OTHER | TP53 2C.2 | 2C.2 | PTEN loss 2C.1 , SMAD4 loss 2C.2 | 2C.1 |  | NL | 2C.1 |
| SP49328 | Liver | OTHER | ARID1A 2C.2 | 2C.2 | CDKN2A loss 2C.1 , CDKN2B loss 2C.2 | 2C.1 |  | NL | 2C.1 |
| SP49334 | Liver | OTHER | CTNNB1 2C.2 , FANCI p.P998L 2C.1 | 2C.1 |  | NL |  | NL | 2C.1 |
| SP49379 | Liver | OTHER | CTNNB1 2C.2 | 2C.2 |  | NL |  | NL | 2C.2 |
| SP49385 | Liver | OTHER |  | NL |  | NL |  | NL | NL |
| SP49391 | Liver | OTHER | TP53 2C.2 | 2C.2 | FAM135B amp 3 , IL7R amp 3 , MEF2B amp 3 , MYC amp 2C.2 , PIK3R2 amp 2C.2 , RICTOR amp 2C.2 , TERT amp 3 , ZNF429 amp 3 | 2C.2 |  | NL | 2C.2 |
| SP49433 | Liver | OTHER | GNAS 2C.2 | 2C.2 |  | NL |  | NL | 2C.2 |
| SP49449 | Liver | OTHER | ARID1A 2C.2 , AXIN1 p.R647Pfs*17 2C.2 | 2C.2 |  | NL |  | NL | 2C.2 |
| SP49469 | Liver | OTHER | CTNNB1 2C.2 | 2C.2 |  | NL |  | NL | 2C.2 |
| SP49481 | Liver | OTHER |  | NL |  | NL |  | NL | NL |
| SP49531 | Liver | OTHER | CDKN1A 2C.2 , CDKN1A p.M1R 2C.2 | 2C.2 | CCND3 amp 2C.2 , DDR1 amp 3 , PIM1 amp 3 , RPS6KB1 amp 3 , RPTOR amp 3 , SPOP amp 3 , TPMT amp 3 | 2C.2 |  | NL | 2C.2 |
| SP49541 | Liver | OTHER | TSC1 2C.1 | 2C.1 | MET amp 2C.1 , MET amp 2C.1 | 2C.1 |  | NL | 2C.1 |
| SP49551 | Liver | OTHER | AXIN2 2D , NF2 p.E427* 2C.1 | 2C.1 | MCL1 amp 2D , MYC amp 2C.2 , RIT1 amp 3 | 2C.2 |  | NL | 2C.1 |
| SP49591 | Liver | OTHER | FAM123B 3 , KEAP1 p.V271L 2C.2 , TP53 p.A161S 2C.2 | 2C.2 | SF3B1 amp 3 | 3 |  | NL | 2C.2 |
| SP49651 | Liver | OTHER | ASXL1 2C.2 , AXIN2 p.G833C 2D | 2C.2 |  | NL |  | NL | 2C.2 |
| SP50115 | Liver | OTHER | DICER1 3 , MAP3K1 p.E747Dfs*15 2C.2 | 2C.2 | B2M loss 2C.2 | 2C.2 |  | NL | 2C.2 |
| SP5017 | Breast | BREAST | ACVR1B 3 , TGFBR2 p.W546* 2D , TP53 p.R342* 2C.2 | 2C.2 | CDKN2A loss 2C.1 , CDKN2B loss 2D , MTAP loss 3 | 2C.1 |  | NL | 2C.1 |
| SP50263 | Lung | LUNG | EGFR 1A.1 , RB1 p.E97Nfs*14 2C.2 , TP53 p.R110L 2C.2 | 1A.1 | EGFR amp 2C.1 , EGFR amp 2C.1 , MYC amp 2C.2 , PXDNL amp 3 | 2C.1 |  | NL | 1A.1 |
| SP50317 | Lung | LUNG | APC 2C.2 , TP53 p.Y220C 2C.2 | 2C.2 | CCND3 amp 2C.2 | 2C.2 |  | NL | 2C.2 |
| SP50321 | Lung | LUNG | KRAS 1a.2 | 1a.2 | CDKN2A loss 2C.1 , CDKN2B loss 2D | 2C.1 |  | NL | 1a.2 |
| SP50406 | Lung | LUNG | KRAS 1a.2 | 1a.2 |  | NL |  | NL | 1a.2 |
| SP50412 | Lung | LUNG | SETD2 2D | 2D |  | NL | ROS1 fusion 1A.1 | 1A.1 | 1A.1 |
| SP50485 | Lung | LUNG | CDKN2A 2C.1 , KRAS p.G12A 1A.2 | 1A.2 | MDM2 amp 2C.2 | 2C.2 |  | NL | 1A.2 |
| SP50518 | Lung | LUNG | EGFR 2C.1 , EGFR p.K754_I759delKANKEI 1A.1 , TP53 p.F54Sfs*69 2C.2 | 1A.1 | AKT2 amp 2C.2 , AURKA amp 3 , CARD11 amp 3 , EGFR amp 2C.1 , EGFR amp 2C.1 , GLI3 amp 3 , GNAS amp 2C.2 , RAC1 amp 3 , ZNF217 amp 3 | 2C.1 |  | NL | 1A.1 |
| SP5052 | Breast | BREAST | FANCA 2C.1 , FAT1 p.Q2852* 3 , INPP4B p.K819N 3 , TP53 p.E204* 2C.2 , ZRSR2 p.P303S 3 | 2C.1 | JAK1 loss 2D , ROS1 amp 2C.1 | 2C.1 |  | NL | 2C.1 |
| SP50592 | Lung | LUNG |  | NL | AR amp 2C.2 , ARAF amp 2C.1 , AURKA amp 3 , DDR2 amp 2C.2 , EGFR amp 2C.1 , EIF1AX amp 3 , FAM135B amp 3 , GNAS amp 2C.2 , MCL1 amp 2D , MYC amp 2C.2 , NTRK1 amp 2C.1 , PLCG1 amp 3 , RIT1 amp 3 , SMC1A amp 3 , SRC amp 2C.2 , TOP1 amp 2D , ZNF217 amp 3 | 2C.1 |  | NL | 2C.1 |
| SP50611 | Lung | LUNG | ARHGAP35 3 , SF3B1 p.K741N 3 , TBX3 p.R637L 2D | 2D | CARD11 amp 3 , EGFR amp 2C.1 , GLI3 amp 3 , RAC1 amp 3 , RHEB amp 3 , RHEB amp 3 , YAP1 amp 3 | 2C.1 |  | NL | 2C.1 |
| SP50713 | Lung | LUNG | ACVR1 3 , KDM5C p.E23K 3 , KEAP1 p.G333S 2C.2 , NOTCH1 p.C1425F 2C.2 , STK11 p.N181I 2C.1 | 2C.1 |  | NL |  | NL | 2C.1 |
| SP50827 | Lung | LUNG | ARID2 3 , ARID2 p.E108Rfs*107 3 , ASXL1 p.R545P 2C.2 , ELF3 p.D74V 3 , KDM5C p.E784* 3 , RBM10 p.W871* 3 , STAG2 p.L997F 3 , TP53 p.K139Rfs*31 2C.2 | 2C.2 |  | NL |  | NL | 2C.2 |
| SP51037 | Lung | LUNG | SMAD4 2C.2 | 2C.2 | CDKN2A loss 2C.1 , CDKN2B loss 2D | 2C.1 |  | NL | 2C.1 |
| SP51446 | Lung | LUNG | CTNNB1 2C.2 , EGFR p.E746_A750delELREA 1A.1 | 1A.1 |  | NL |  | NL | 1A.1 |
| SP51824 | Lung | LUNG | BRAF 2C.1 , CREBBP p.P10S 2D , CTNNB1 p.T41A 2C.2 , KEAP1 p.F246L 2C.2 | 2C.1 | IKBKB amp 3 | 3 |  | NL | 2C.1 |
| SP52232 | Lung | LUNG | ATM 2C.1 , KRAS p.G12V 1a.2 , NTRK1 p.G625W 2C.1 , STK11 p.D53Gfs*110 2C.1 | 1a.2 | IKBKB amp 3 , PXDNL amp 3 | 3 |  | NL | 1a.2 |
| SP52284 | Lung | LUNG | RASA1 3 , RB1 p.N290Kfs*20 2C.2 , RBM10 . 3 , TP53 p.Y163C 2C.2 | 2C.2 | TERT amp 3 | 3 |  | NL | 2C.2 |
| SP52607 | Lung | LUNG | U2AF1 3 | 3 |  | NL | ROS1 fusion 1A.1 | 1A.1 | 1A.1 |
| SP52667 | Lung | LUNG | HRAS 2C.1 , KDM5C p.M506I 3 , TP53 p.R280I 2C.2 | 2C.1 | CCND3 amp 2C.2 , IKBKB amp 3 | 2C.2 |  | NL | 2C.1 |
| SP52779 | Lung | LUNG | CREBBP 2D , CTNNB1 p.S37F 2C.2 , KRAS p.G12V 1a.2 , MLL p.R2521H 3 , RBM10 p.E800* 3 | 1a.2 |  | NL |  | NL | 1a.2 |
| SP5279 | Breast | BREAST | BRIP1 2C.1 , TP53 p.V157F 2C.2 | 2C.1 | AURKA amp 3 , CCND1 amp 2C.2 , CDKN2A loss 2C.1 , CDKN2B loss 2D , ERBB2 amp 1A.1 , GNAS amp 2C.2 , MTAP loss 3 , PIK3CA amp 2C.1 , PIK3CA amp 2C.1 , RPS6KB1 amp 3 , ZNF217 amp 3 | 1A.1 | PIK3CA fusion 2C.1 | 2C.1 | 1A.1 |
| SP53073 | Lung | LUNG | NF1 2C.1 , TP53 p.Q192* 2C.2 | 2C.1 | MAP2K4 loss 2C.1 | 2C.1 |  | NL | 2C.1 |
| SP53387 | Lung | LUNG | FLT3 2C.1 , STK11 p.G288V 2C.1 | 2C.1 | TERT amp 3 | 3 |  | NL | 2C.1 |
| SP53548 | Lung | LUNG | ATM 2C.1 , BCOR p.N1459S 3 | 2C.1 |  | NL | ALK fusion 1A.1 | 1A.1 | 1A.1 |
| SP53618 | Lung | LUNG | ARID1A 2C.2 , MAP2K1 p.K57N 2C.1 , TP53 p.E224D 2C.2 | 2C.1 | CCND1 amp 2C.2 , FGF19 amp 2D , FLT3 amp 2C.1 , FOXA1 amp 3 | 2C.1 |  | NL | 2C.1 |
| SP5381 | Breast | BREAST | MAP3K1 2C.2 , MAP3K1 p.N325Lfs*3 2C.2 , PIK3CA p.E545K 1A.1 | 1A.1 |  | NL |  | NL | 1A.1 |
| SP53810 | Lung | LUNG | AXIN1 2C.2 , BRAF p.G596R 2C.1 , DICER1 p.K1557E 3 , DPYD p.G341E 3 , FAS p.C143F 3 , GATA2 p.G143V 2C.2 , MLL3 p.E66V 3 , MLL3 p.P3459Tfs*3 3 , NOTCH1 p.G863V 2C.2 , TET2 p.A1158S 2D , TP53 p.G262V 2C.2 , TSC2 p.D396Y 2C.1 | 2C.1 | CDKN2A loss 2C.1 , CDKN2B loss 2D , JAK2 loss 2D | 2C.1 |  | NL | 2C.1 |
| SP5393 | Breast | BREAST | CYLD 3 , TP53 p.V122Dfs*26 2C.2 | 2C.2 | PMS1 loss 2C.2 , PTEN loss 2C.1 | 2C.1 |  | NL | 2C.1 |
| SP54113 | Lung | LUNG | ARID1A 2C.2 , BRCA2 p.G2063R 2C.1 , FLT4 p.M697I 2C.2 , PIK3CA p.E545K 2C.1 , RB1 p.D697E 2C.2 , TP53 p.R249Gfs*96 2C.2 | 2C.1 |  | NL |  | NL | 2C.1 |
| SP54363 | Lung | LUNG | KEAP1 2C.2 , KRAS p.G12V 1a.2 , POLE p.K983N 2C.1 , RBM10 p.W658C 3 , RBM10 p.W658* 3 , TP53 p.C176Y 2C.2 | 1a.2 |  | NL |  | NL | 1a.2 |
| SP5448 | Breast | BREAST | CDH1 2D , CDH1 p.P201Lfs*14 2D , PIK3CA p.H1047R 1A.1 , TP53 p.Q165Afs*6 2C.2 | 1A.1 | AURKC amp 3 , BCL2L12 amp 3 , CCND1 amp 2C.2 , ERBB2 amp 1A.1 , FAM135B amp 3 , FGF19 amp 2D , FGF3 amp 2D , FGF4 amp 2D , GLI3 amp 3 , GLI3 amp 3 , IKBKB amp 3 , MYC amp 2C.2 , PPP2R1A amp 2C.1 , SPOP amp 3 | 1A.1 |  | NL | 1A.1 |
| SP5473 | Breast | BREAST | NOTCH1 2C.2 , TP53 p.K292Gfs*52 2C.2 | 2C.2 | AKT2 amp 2C.2 , CCNE1 amp 2C.2 , FAM135B amp 3 , MYC amp 2C.2 , PXDNL amp 3 | 2C.2 |  | NL | 2C.2 |
| SP54745 | Lung | LUNG | KDM5C 3 , SMARCA4 p.E1525* 2C.2 | 2C.2 | CDKN2A loss 2C.1 , CDKN2B loss 2D | 2C.1 |  | NL | 2C.1 |
| SP55004 | Lung | LUNG |  | NL |  | NL |  | NL | NL |
| SP55142 | Lung | LUNG | ARID1A 2C.2 , ASXL1 p.S135Y 2C.2 , BCL6 p.R214L 3 , CCND1 p.D25A 2C.2 , CTNNB1 p.S37C 2C.2 , IKBKB p.E495* 3 , JAK1 p.K496N 2D , MLL p.L1602I 3 , MLL2 p.G2141W 3 , POT1 p.D224H 3 , PTCH1 p.W256C 2C.1 , RUNX1 p.D198N 3 , SETBP1 p.E255Q 3 , SMARCA4 p.Y1115*fs*1 2C.2 , TRRAP p.E729K 3 | 2C.1 |  | NL |  | NL | 2C.1 |
| SP55235 | Lung | LUNG | FGFR1 2C.1 , MUTYH p.A287D 2C.2 , SETD2 p.T305Qfs*35 2D , TP53 p.G105D 2C.2 | 2C.1 | BRAF amp 2C.1 , CARD11 amp 3 , CCND2 amp 2C.2 , CDK6 amp 2C.1 , CHD4 amp 3 , DDR2 amp 2C.2 , EGFR amp 2C.1 , FGF23 amp 3 , GLI3 amp 3 , IL7R amp 3 , MCL1 amp 2D , NTRK1 amp 2C.1 , RAC1 amp 3 , RIT1 amp 3 , SMO amp 2C.1 , SRC amp 2C.2 | 2C.1 | ERBB4 fusion 2C.1 | 2C.1 | 2C.1 |
| SP55309 | Lung | LUNG |  | NL |  | NL | RET fusion 1A.1 | 1A.1 | 1A.1 |
| SP55387 | Lung | LUNG | TP53 2C.2 | 2C.2 |  | NL |  | NL | 2C.2 |
| SP55509 | Lung | LUNG | FAM123B 3 , KRAS p.G12C 1a.2 , RBM10 p.G870C 3 , SRGAP1 p.F764C 3 | 1a.2 | CDKN2A loss 2C.1 | 2C.1 |  | NL | 1a.2 |
| SP5559 | Breast | BREAST | ABL1 2C.1 , AKT1 p.E17K 2C.2 , ATRX p.R840Nfs*8 2C.2 , MLL3 p.K3784Ifs*8 3 , SMAD4 p.N316Ifs*20 2C.2 , TP53 p.R209Kfs*6 2C.2 | 2C.1 | FAT1 loss 3 , JAK1 loss 2D , PLCG1 amp 3 , TOP1 amp 2D | 2D |  | NL | 2C.1 |
| SP55711 | Lung | LUNG | HLA-A 2C.2 | 2C.2 | CCNE1 amp 2C.2 , CDK4 amp 2C.1 , ERBB3 amp 2C.2 , MDM2 amp 2C.2 , PPP6C amp 3 , STAT6 amp 3 , TERT amp 3 , TERT amp 3 , ZNF429 amp 3 , ZNF429 amp 3 | 2C.1 |  | NL | 2C.1 |
| SP56079 | Lung | LUNG | CDK6 2C.1 , CDKN1B p.L13Gfs*111 2D , FANCG p.K504* 2C.1 , HDAC9 p.V801F 3 , KEAP1 p.G603W 2C.2 , TP53 p.R248P 2C.2 | 2C.1 | ABCB1 amp 3 , BRAF amp 2C.1 , CDK6 amp 2C.1 , MET amp 2C.1 , SMO amp 2C.1 | 2C.1 |  | NL | 2C.1 |
| SP56168 | Lung | LUNG | ARID1B 2D , ARID1B p.Q1938H 2D | 2D | CDK4 amp 2C.1 , CTNND2 amp 3 , CTNND2 amp 3 , CTNND2 amp 3 , CTNND2 amp 3 , CTNND2 amp 3 , CTNND2 amp 3 , MDM2 amp 2C.2 , SMAD4 loss 2C.2 , STAT6 amp 3 , TERT amp 3 | 2C.1 |  | NL | 2C.1 |
| SP56303 | Lung | LUNG | EGFR 1A.1 , MGA p.E437* 3 , SMARCB1 p.R377H 2D | 1A.1 | ABL2 amp 3 , CARD11 amp 3 , CCND1 amp 2C.2 , CDK4 amp 2C.1 , DDR2 amp 2C.2 , EGFR amp 2C.1 , FGF19 amp 2D , FGF4 amp 2D , FGFR1 amp 2C.1 , FGFR3 amp 2C.1 , FOXA1 amp 3 , GLI3 amp 3 , IKBKB amp 3 , MCL1 amp 2D , MDM2 amp 2C.2 , NTRK1 amp 2C.1 , RAC1 amp 3 , RICTOR amp 2C.2 , RIT1 amp 3 , SLCO1B3 amp 3 , STAT6 amp 3 | 2C.1 |  | NL | 1A.1 |
| SP56460 | Lung | LUNG | HIST1H3B 3 , NFE2L2 p.G31A 2C.2 , TP53 p.R282Q 2C.2 | 2C.2 | CDKN2A loss 2C.1 , CDKN2B loss 2D , MTAP loss 3 , PTEN loss 2C.1 | 2C.1 |  | NL | 2C.1 |
| SP56474 | Lung | LUNG | FLT4 2C.2 , SMARCA4 p.E1242K 2C.2 , TP53 p.P190L 2C.2 | 2C.2 | CCNE1 amp 2C.2 , TERT amp 3 | 2C.2 |  | NL | 2C.2 |
| SP56502 | Lung | LUNG | ARID1B 2D , MLL2 p.Q4364Sfs*20 3 , PTEN p.Q245* 2C.1 , TP53 p.H193L 2C.2 | 2C.1 | CDKN2A loss 2C.1 , CDKN2B loss 2D , FAT1 loss 3 , H3F3B amp 3 | 2C.1 |  | NL | 2C.1 |
| SP56533 | Lung | LUNG | APC 2C.2 , MLL2 p.R2687Qfs*4 3 , NFE2L2 p.E79Q 2C.2 , TP53 p.G244S 2C.2 | 2C.2 |  | NL |  | NL | 2C.2 |
| SP56537 | Lung | LUNG | CD79B 3 , DPYD p.G68C 3 , EP300 p.L1398P 2D , TP53 p.R337L 2C.2 | 2C.2 | MECOM amp 3 , PIK3CA amp 2C.1 , PTEN loss 2C.1 | 2C.1 |  | NL | 2C.1 |
| SP56541 | Lung | LUNG | ARID1B 2D , RET p.W917R 2C.1 , TP53 p.Q192_H193delQH 2C.2 | 2C.1 | RB1 loss 2C.2 | 2C.2 |  | NL | 2C.1 |
| SP56553 | Lung | LUNG | BARD1 2C.1 , BMPR2 p.S428F 2C.1 , CDKN2A p.G150S 2C.1 , CUL3 p.L321F 3 , DICER1 p.M684I 3 , HLA-B p.H117D 3 , INPP4B p.M879I 3 , MGA p.L714F 3 , NF1 p.D1481H 2C.1 , PBRM1 p.D823H 2D , RASA2 p.R366L 3 , TET2 p.P1367S 2D , TET2 p.G1313R 2D , TP53 p.R213* 2C.2 | 2C.1 | CCND1 amp 2C.2 , EGFR amp 2C.1 , FGF19 amp 2D , FGF3 amp 2D , FGF4 amp 2D , PIK3CA amp 2C.1 , YES1 amp 3 | 2C.1 |  | NL | 2C.1 |
| SP56566 | Lung | LUNG | CCND1 2C.2 , CDH1 p.A873T 2D , CYLD p.C817Y 3 , ELF3 p.R251L 3 , MRE11A p.N354I 2C.1 , NCOR1 p.P1033L 3 , NFE2L2 p.R34G 2C.2 , TP53 p.G245C 2C.2 | 2C.1 | BCL6 amp 3 , CCNE1 amp 2C.2 , CDKN2A loss 2C.1 , CDKN2B loss 2D , ERBB2 amp 2C.1 , FGFR1 amp 2C.1 , MTAP loss 3 , MYC amp 2C.2 , PIK3CA amp 2C.1 , PIK3CB amp 2C.1 , RARA amp 3 | 2C.1 |  | NL | 2C.1 |
| SP56569 | Lung | LUNG |  | NL | PTEN loss 2C.1 | 2C.1 |  | NL | 2C.1 |
| SP56607 | Lung | LUNG | CDKN2A 2C.1 , CREBBP p.K1327* 2D , NFE2L2 p.R34Q 2C.2 , TP53 p.E286K 2C.2 | 2C.1 |  | NL |  | NL | 2C.1 |
| SP56644 | Lung | LUNG | BRAF 2C.1 , DNMT3A p.R209P 3 , ERBB4 p.Y921N 2C.2 , FANCF p.W26* 2C.1 , FAT1 p.Y3981Ffs*2 3 , FBXW7 p.S398Y 2C.2 , MAP2K4 p.S184L 2C.1 , MLH3 p.Q1271R 2C.2 , MLL2 p.T4938Pfs*57 3 , NFE2L2 p.R34P 2C.2 , RHOA p.E47K 3 | 2C.1 | BCL6 amp 3 , EGFR amp 2C.1 , MECOM amp 3 | 2C.1 |  | NL | 2C.1 |
| SP5666 | Breast | BREAST | AKT1 2C.2 , CDH1 p.D291Mfs*3 2D , TP53 p.M246I 2C.2 | 2C.2 |  | NL |  | NL | 2C.2 |
| SP56704 | Lung | LUNG | FAT1 3 , FLT4 p.T552N 2C.2 , KDM5C p.E646V 3 , PIK3CA p.D1045V 2C.1 , TNFAIP3 p.G622C 3 , TP53 p.G244C 2C.2 | 2C.1 |  | NL |  | NL | 2C.1 |
| SP56730 | Lung | LUNG | NOTCH1 2C.2 , PTEN p.V166Sfs*14 2C.1 , TP53 p.I162dupI 2C.2 | 2C.1 |  | NL |  | NL | 2C.1 |
| SP56754 | Lung | LUNG | HDAC9 3 , HRAS p.G13R 2C.1 , NFE2L2 p.D29H 2C.2 , TP53 p.G245S 2C.2 , ZFHX3 p.W804Vfs*5 3 | 2C.1 | BCL6 amp 3 , GATA2 amp 2C.2 , MECOM amp 3 , PIK3CA amp 2C.1 , PIK3CB amp 2C.1 , RIT1 amp 3 , YES1 amp 3 | 2C.1 |  | NL | 2C.1 |
| SP56771 | Lung | LUNG | CDKN2A 2C.1 , CREBBP p.R1140Q 2D , HNF1A p.P94H 2C.2 , NFE2L2 p.R34P 2C.2 , TP53 p.C242F 2C.2 | 2C.1 |  | NL |  | NL | 2C.1 |
| SP56821 | Lung | LUNG | ARID1A 2C.2 , ETV6 p.R181L 2C.2 , FAT1 p.Q1981* 3 , TP53 p.S315Lfs*30 2C.2 | 2C.2 | BCL6 amp 3 , CCND2 amp 2C.2 , CHD4 amp 3 , FGF23 amp 3 , KRAS amp 2C.1 , MECOM amp 3 , PIK3CA amp 2C.1 , SLCO1B3 amp 3 | 2C.1 |  | NL | 2C.1 |
| SP56827 | Lung | LUNG | NF1 2C.1 , PTCH1 p.R783L 2C.1 , RASA1 p.M802I 3 , SMARCA4 p.D694Y 2C.2 , TP53 p.Y163C 2C.2 | 2C.1 | AKT2 amp 2C.2 , AXL amp 2C.2 , BAP1 loss 2C.1 , BCL6 amp 3 , CCNE1 amp 2C.2 , PIK3CA amp 2C.1 | 2C.1 |  | NL | 2C.1 |
| SP56941 | Lung | LUNG | ARID1A 2C.2 , BAP1 p.R227H 2C.1 , CREBBP p.R1446C 2D , CREBBP p.V1449Ffs*10 2D , FBXW7 p.R465H 2C.2 , FGFR3 p.R248C 2C.1 , MLL2 p.Q3745* 3 , MLL3 p.S2494Efs*31 3 , PIK3CA p.E542K 2C.1 , PIK3CA p.D538N 2C.1 , RAD51D p.Q130E 2C.1 | 2C.1 | CDKN2A loss 2C.1 , MTAP loss 3 | 2C.1 |  | NL | 2C.1 |
| SP57024 | Lung | LUNG | TP53 2C.2 | 2C.2 | CYLD loss 3 | 3 |  | NL | 2C.2 |
| SP57066 | Lung | LUNG | ARID1B 2D , CREBBP p.P616R 2D , NFE2L2 p.R34Q 2C.2 , NOTCH1 p.W1075* 2C.2 , TP53 p.R158L 2C.2 | 2C.2 | XPO1 amp 2C.2 | 2C.2 |  | NL | 2C.2 |
| SP57084 | Lung | LUNG | CDKN2A 2C.1 , CREBBP p.S646I 2D , TP53 p.R175H 2C.2 | 2C.1 | MECOM amp 3 , PIK3CA amp 2C.1 , PIM1 amp 3 | 2C.1 |  | NL | 2C.1 |
| SP57189 | Lung | LUNG | BRCA1 2C.1 , CUL3 p.S199* 3 , ERBB4 p.L713F 2C.2 , FGFR2 p.K660E 2C.1 , KDM6A p.G693W 2D , KEAP1 p.G186R 2C.2 , MLL2 p.E1742* 3 , MYCN p.R398L 3 , PTEN p.G165E 2C.1 , TP53 p.G245V 2C.2 | 2C.1 | CDKN2A loss 2C.1 , CDKN2B loss 2D , MTAP loss 3 , YES1 amp 3 | 2C.1 |  | NL | 2C.1 |
| SP57251 | Lung | LUNG | DPYD 3 , FBXW7 p.R505G 2C.2 , TP53 p.H179R 2C.2 | 2C.2 | BCL6 amp 3 , CCND1 amp 2C.2 , CDKN2A loss 2C.1 , CDKN2B loss 2D , DDR2 amp 2C.2 , FGF19 amp 2D , FGF3 amp 2D , FGF4 amp 2D , FGFR4 amp 2C.1 , MCL1 amp 2D , MECOM amp 3 , MTAP loss 3 , NTRK1 amp 2C.1 , PIK3CA amp 2C.1 , RIT1 amp 3 | 2C.1 |  | NL | 2C.1 |
| SP57267 | Lung | LUNG | CDKN2A 2C.1 , CREBBP p.W1472C 2D , NFE2L2 p.Q26L 2C.2 , PIK3C2B p.G1109W 3 , TP53 p.E68* 2C.2 | 2C.1 | CCND1 amp 2C.2 , FGF19 amp 2D , FGF3 amp 2D , FGF4 amp 2D , MECOM amp 3 , YAP1 amp 3 , YAP1 amp 3 , YAP1 amp 3 | 2C.2 |  | NL | 2C.1 |
| SP57450 | Lung | LUNG | CDKN2A 2C.1 , MLL2 p.E2393* 3 , NFE2L2 p.R34Q 2C.2 , TP53 p.V157F 2C.2 | 2C.1 | CCND2 amp 2C.2 , CHD4 amp 3 , FGF23 amp 3 , IL7R amp 3 , KRAS amp 2C.1 , MDM2 amp 2C.2 , PIK3CA amp 2C.1 , RICTOR amp 2C.2 , SLCO1B3 amp 3 | 2C.1 |  | NL | 2C.1 |
| SP57538 | Lung | LUNG | FAT1 3 , HDAC9 p.D162H 3 , NOTCH1 p.E2075* 2C.2 , RASA1 p.R537Sfs*7 3 | 2C.2 | CCND1 amp 2C.2 , CDKN2A loss 2C.1 , CDKN2B loss 2D , FGF19 amp 2D , SLCO1B3 amp 3 | 2C.1 |  | NL | 2C.1 |
| SP57586 | Lung | LUNG | NFE2L2 2C.2 , NFE2L2 p.E79D 2C.2 , TP53 p.C135F 2C.2 | 2C.2 | CCND1 amp 2C.2 , FGF19 amp 2D , FGF3 amp 2D , FGF4 amp 2D , FOXA1 amp 3 , XPO1 amp 2C.2 , YAP1 amp 3 | 2C.2 |  | NL | 2C.2 |
| SP57619 | Lung | LUNG | BLM 2C.1 , MLL2 p.Q3333* 3 , NOTCH1 p.K428* 2C.2 , PPP2R1A p.R144H 2C.1 , TP53 p.Y220C 2C.2 | 2C.1 | ERAP2 loss 3 , YAP1 amp 3 , YAP1 amp 3 , YAP1 amp 3 , YAP1 amp 3 | 3 |  | NL | 2C.1 |
| SP57629 | Lung | LUNG | CDKN2A 2C.1 , NFE2L2 p.W24C 2C.2 | 2C.1 | BCL6 amp 3 , KDM6A loss 2D , MECOM amp 3 , PIK3CA amp 2C.1 | 2C.1 |  | NL | 2C.1 |
| SP57651 | Lung | LUNG | BLM 2C.1 , TP53 p.L265Tfs*7 2C.2 | 2C.1 | MECOM amp 3 , PIK3CA amp 2C.1 | 2C.1 |  | NL | 2C.1 |
| SP57669 | Lung | LUNG | MLL2 3 , NOTCH1 p.A973Qfs*206 2C.2 , TP53 p.Q104* 2C.2 | 2C.2 | PTEN loss 2C.1 , XPO1 amp 2C.2 | 2C.1 |  | NL | 2C.1 |
| SP57735 | Lung | LUNG | ARID1B 2D , ATM p.G2897S 2C.1 , CDC73 p.T304K 3 , MLL2 p.Q3907* 3 , NOTCH1 p.S1152Afs*27 2C.2 , TP53 p.R175G 2C.2 | 2C.1 | CCND1 amp 2C.2 , FGF19 amp 2D , FGF3 amp 2D , FGF4 amp 2D , MYC amp 2C.2 | 2C.2 |  | NL | 2C.1 |
| SP57818 | Lung | LUNG | HDAC9 3 , KRAS p.G12A 1a.2 , NFE2L2 p.D29G 2C.2 , NFE2L2 p.R34L 2C.2 , PIK3CA p.E545K 2C.1 , RBM10 p.E393* 3 | 1a.2 | CDKN2A loss 2C.1 , CDKN2B loss 2D , MTAP loss 3 | 2C.1 |  | NL | 1a.2 |
| SP5784 | Breast | BREAST | HDAC9 3 , PTEN p.D326Efs*4 2C.1 , PTEN p.A126G 2C.1 , TP53 p.W91* 2C.2 | 2C.1 | BCL6 amp 3 , CDKN2A loss 2C.1 , CDKN2B loss 2D , JAK2 loss 2D , NF1 loss 2C.1 | 2C.1 |  | NL | 2C.1 |
| SP57846 | Lung | LUNG | ARHGAP35 3 , CBFB p.R156T 3 , CREBBP p.Q503* 2D , ERBB2 p.E1021Q 2C.1 , HRAS p.Q61K 2C.1 , HRAS p.V14L 2C.1 , MLL2 p.Q3370Pfs*53 3 , MLL3 p.Q1791* 3 , SETD2 p.V2370F 2D , TP53 p.G266V 2C.2 | 2C.1 | BCL6 amp 3 , GATA2 amp 2C.2 , MECOM amp 3 , PIK3CA amp 2C.1 , PIK3CB amp 2C.1 | 2C.1 |  | NL | 2C.1 |
| SP57901 | Lung | LUNG | CUL3 3 , NFE2L2 p.R34Q 2C.2 , TP53 p.L252_T253delinsP 2C.2 | 2C.2 | DPYD loss 3 , PIK3CA amp 2C.1 | 2C.1 |  | NL | 2C.1 |
| SP57933 | Lung | LUNG | BRCA1 2C.1 , BRCA2 p.R2500Sfs*24 2C.1 , JAK2 p.P58Hfs*30 2D , NOTCH1 p.V2038L 2C.2 , PIK3CA p.H1047R 2C.1 , TET2 p.G92Efs*19 2D , TET2 p.G92Afs*19 2D , TP53 p.I195S 2C.2 | 2C.1 | CCND1 amp 2C.2 , FGF19 amp 2D , FGF3 amp 2D , FGF4 amp 2D , FOXA1 amp 3 , RB1 loss 2C.2 | 2C.2 |  | NL | 2C.1 |
| SP57941 | Lung | LUNG | ARID1B 2D , BRCA1 p.E515V 2C.1 , TP53 p.R175G 2C.2 | 2C.1 | BRAF amp 2C.1 , CTNND2 amp 3 , FAM135B amp 3 , FGFR1 amp 2C.1 , IL7R amp 3 , MGA loss 3 , MYC amp 2C.2 , PIK3CB amp 2C.1 , PXDNL amp 3 , RHEB amp 3 , RICTOR amp 2C.2 , TERT amp 3 | 2C.1 |  | NL | 2C.1 |
| SP5808 | Breast | BREAST | BRCA1 1A.1 , GATA3 p.M401Vfs*106 2D , SMAD4 p.L109R 2C.2 , TNFAIP3 . 3 | 1A.1 | AKT3 amp 2C.2 , BCL6 amp 3 , CTNND2 amp 3 , FAM135B amp 3 , FOXA1 amp 2C.2 , H3F3A amp 2C.2 , IDH2 amp 2C.1 , IGF1R amp 2D , IGF1R amp 2D , IGF1R amp 2D , IGF1R amp 2D , IL7R amp 3 , MDM4 amp 2D , PIK3C2B amp 3 , RICTOR amp 2C.2 , RPS6KB1 amp 3 , SPOP amp 3 , SPOP amp 3 | 2C.1 |  | NL | 1A.1 |
| SP58101 | Lung | LUNG | APC 2C.2 , CDKN2A p.Y44*fs*1 2C.1 , NOTCH1 p.R1784L 2C.2 , SMAD4 p.D537E 2C.2 , TP53 p.E221* 2C.2 | 2C.1 | XPO1 amp 2C.2 | 2C.2 |  | NL | 2C.1 |
| SP5820 | Breast | BREAST |  | NL | ERBB2 amp 1A.1 , H3F3B amp 3 , MDM4 amp 2D , PIK3C2B amp 3 , PIK3C2B amp 3 , PIK3C2B amp 3 , SPOP amp 3 | 1A.1 |  | NL | 1A.1 |
| SP58245 | Lung | LUNG | NOTCH1 2C.2 , NOTCH1 p.C429S 2C.2 | 2C.2 | FGFR1 amp 2C.1 | 2C.1 |  | NL | 2C.1 |
| SP58326 | Lung | LUNG | BCOR 3 , CUL3 p.V643Ffs*11 3 , NFE2L2 p.D29Y 2C.2 , TP53 p.R248Q 2C.2 | 2C.2 | B2M loss 2C.2 , BCL6 amp 3 , CDKN2A loss 2C.1 , CDKN2B loss 2D , MTAP loss 3 , PIK3CA amp 2C.1 | 2C.1 |  | NL | 2C.1 |
| SP58342 | Lung | LUNG | CDH1 2D , FLT4 p.S260* 2C.2 , FLT4 p.G1072S 2C.2 , NOTCH1 p.S2211* 2C.2 , NTRK1 p.G368C 2C.1 , PIK3CA p.E545K 2C.1 , TP53 p.R249G 2C.2 | 2C.1 | AKT2 amp 2C.2 , BCL6 amp 3 , CDKN2A loss 2C.1 , CDKN2B loss 2D , FGFR1 amp 2C.1 , GATA2 amp 2C.2 , MECOM amp 3 , MTAP loss 3 , NFE2L2 amp 2C.2 , PIK3CA amp 2C.1 , PIK3CB amp 2C.1 | 2C.1 | FGR fusion 2C.2 | 2C.2 | 2C.1 |
| SP58349 | Lung | LUNG | ARID1A 2C.2 , CREBBP p.D1435Y 2D , NOTCH1 p.G1165Qfs*13 2C.2 , TP53 p.H179L 2C.2 | 2C.2 | CDKN2A loss 2C.1 , CDKN2B loss 2D , FGFR3 amp 2C.1 , MTAP loss 3 | 2C.1 |  | NL | 2C.1 |
| SP5844 | Breast | BREAST | APC 2C.2 , KDM6A . 2D , TP53 p.A86Cfs*63 2C.2 | 2C.2 | FAM135B amp 3 , FGFR1 amp 2C.1 , FGFR2 amp 2C.1 , IKBKB amp 3 , MYC amp 2C.2 , PTEN loss 2C.1 , PXDNL amp 3 | 2C.1 |  | NL | 2C.1 |
| SP58612 | Lung | LUNG | NOTCH2 2C.2 , TP53 p.H193D 2C.2 , WT1 p.Y354D 2C.2 | 2C.2 | BCL6 amp 3 , CTNND2 amp 3 , IL7R amp 3 , MECOM amp 3 , PIK3CA amp 2C.1 , PIK3CB amp 2C.1 , RICTOR amp 2C.2 , ZNF429 amp 3 | 2C.1 |  | NL | 2C.1 |
| SP58668 | Lung | LUNG | B2M 2C.2 , CDKN2A p.R58* 2C.1 , PIK3CA p.E542K 2C.1 , RET p.L940V 2C.1 | 2C.1 | CCND1 amp 2C.2 , FGF19 amp 2D , FGF3 amp 2D , FGF4 amp 2D , FOXA1 amp 3 , MET amp 2C.1 | 2C.1 |  | NL | 2C.1 |
| SP58882 | Lung | LUNG | CDKN2A 2C.1 , NFE2L2 p.R34G 2C.2 , TP53 p.G105C 2C.2 | 2C.1 | BCL6 amp 3 , MECOM amp 3 , PIK3CA amp 2C.1 | 2C.1 |  | NL | 2C.1 |
| SP58991 | Lung | LUNG | KDM6A 2D , NOTCH1 p.E1719* 2C.2 , SMARCA4 p.A1168Qfs*4 2C.2 , TP53 p.Q38* 2C.2 | 2C.2 | FGFR1 amp 2C.1 | 2C.1 |  | NL | 2C.1 |
| SP59245 | Lung | LUNG | FLT3 2C.1 , NFE2L2 p.G31A 2C.2 , PIK3CA p.E545K 2C.1 , SMARCB1 p.T149K 2D , SPEN p.R3536G 3 , TP53 p.H193L 2C.2 | 2C.1 | AKT2 amp 2C.2 , PIK3CA amp 2C.1 | 2C.1 |  | NL | 2C.1 |
| SP59270 | Lymphoid | OTHER | ARID1A 2C.2 , DDX3X p.R475H 3 , SMARCA4 p.R973Q 2C.2 | 2C.2 |  | NL |  | NL | 2C.2 |
| SP59272 | Lymphoid | OTHER | FOXO1 3 , RAF1 p.E478K 2C.2 , RHOA p.R5Q 3 , TP53 p.V172D 2C.2 | 2C.2 |  | NL |  | NL | 2C.2 |
| SP59276 | Lymphoid | OTHER | CREBBP 2C.2 , MYC p.F153C 2C.2 , TP53 p.S215R 2C.2 | 2C.2 |  | NL |  | NL | 2C.2 |
| SP59280 | Lymphoid | OTHER | MYC 2C.2 , RHOA p.R5Q 3 , RHOA p.I23R 3 , SPEN p.L440P 3 | 2C.2 |  | NL |  | NL | 2C.2 |
| SP59284 | Lymphoid | OTHER | APC 2C.2 , PIK3CA p.V344G 2C.1 , RHOA p.R5Q 3 , SMARCB1 p.R376G 2D , TP53 p.R273H 2C.2 | 2C.1 |  | NL |  | NL | 2C.1 |
| SP59288 | Lymphoid | OTHER | ERBB4 2C.2 , PTEN . 2C.1 | 2C.1 |  | NL |  | NL | 2C.1 |
| SP59292 | Lymphoid | OTHER | MLL2 3 | 3 |  | NL |  | NL | 3 |
| SP59296 | Lymphoid | OTHER | PTEN 2C.1 , SMARCA4 p.R973W 2C.2 , TP53 p.R273H 2C.2 | 2C.1 |  | NL |  | NL | 2C.1 |
| SP59300 | Lymphoid | OTHER | POT1 3 | 3 | DDR2 amp 2C.2 , FAM135B amp 3 | 2C.2 |  | NL | 2C.2 |
| SP59304 | Lymphoid | OTHER | ATR 2C.2 , BCL6 p.H669Y 2C.2 , PTPN11 p.L475P 2C.2 , SOCS1 p.V2I 3 , SOCS1 p.R160H 3 | 2C.2 |  | NL |  | NL | 2C.2 |
| SP59308 | Lymphoid | OTHER | MLL2 3 , MLL2 p.Q2820* 3 | 3 |  | NL |  | NL | 3 |
| SP59312 | Lymphoid | OTHER | MLL2 3 , NOTCH2 p.R2400* 2C.2 , PIK3CD p.E1021K 3 , TBX3 p.R310* 2D | 2C.2 |  | NL |  | NL | 2C.2 |
| SP59316 | Lymphoid | OTHER | MLL2 3 , MLL2 p.V2551Cfs*104 3 | 3 |  | NL |  | NL | 3 |
| SP59320 | Lymphoid | OTHER | CREBBP 2C.2 , MLL2 p.Q3732Rfs*17 3 , PBRM1 p.N122Y 2D , STAT6 p.D419H 3 | 2C.2 |  | NL |  | NL | 2C.2 |
| SP59324 | Lymphoid | OTHER |  | NL | CDKN2A loss 2C.1 , CDKN2B loss 2C.2 | 2C.1 |  | NL | 2C.1 |
| SP59328 | Lymphoid | OTHER | CARD11 2C.2 , CTCF p.D451V 3 , EZH2 p.Y641H 2C.2 | 2C.2 |  | NL |  | NL | 2C.2 |
| SP59332 | Lymphoid | OTHER | DDX3X 3 , SMARCA4 p.K981E 2C.2 | 2C.2 |  | NL |  | NL | 2C.2 |
| SP59336 | Lymphoid | OTHER | RHOA 3 , SMARCA4 p.R1243W 2C.2 , TP53 p.G245S 2C.2 | 2C.2 |  | NL |  | NL | 2C.2 |
| SP59340 | Lymphoid | OTHER | EP300 2C.2 , MLL2 p.R2915* 3 , MLL2 p.H3000Pfs*2 3 , SMARCA4 p.R1157W 2C.2 | 2C.2 | CDKN2A loss 2C.1 , CDKN2B loss 2C.2 | 2C.1 |  | NL | 2C.1 |
| SP59344 | Lymphoid | OTHER | ARID1A 2C.2 | 2C.2 |  | NL |  | NL | 2C.2 |
| SP59348 | Lymphoid | OTHER | ATM 2C.1 , ELF3 p.R349Q 3 , STAT6 p.G416R 3 | 2C.1 |  | NL |  | NL | 2C.1 |
| SP59352 | Lymphoid | OTHER | CREBBP 2C.2 , CREBBP p.I1649Sfs*95 2C.2 , DPYD p.P232L 3 , EZH2 p.Y641F 2C.2 | 2C.2 |  | NL |  | NL | 2C.2 |
| SP59356 | Lymphoid | OTHER | SPEN 3 | 3 | TNFRSF14 loss 3 | 3 |  | NL | 3 |
| SP59360 | Lymphoid | OTHER | DDX3X 3 | 3 |  | NL |  | NL | 3 |
| SP59364 | Lymphoid | OTHER |  | NL | B2M loss 2C.2 | 2C.2 |  | NL | 2C.2 |
| SP59368 | Lymphoid | OTHER |  | NL | PXDNL loss 3 | 3 |  | NL | 3 |
| SP59372 | Lymphoid | OTHER | HLA-B 3 | 3 |  | NL |  | NL | 3 |
| SP59376 | Lymphoid | OTHER |  | NL | CDK6 amp 2C.1 | 2C.1 |  | NL | 2C.1 |
| SP59380 | Lymphoid | OTHER | CREBBP 2C.2 , PIK3R1 p.D708V 2C.2 | 2C.2 |  | NL |  | NL | 2C.2 |
| SP59384 | Lymphoid | OTHER | PIK3R1 2C.2 , TP53 p.P190L 2C.2 | 2C.2 |  | NL |  | NL | 2C.2 |
| SP59388 | Lymphoid | OTHER | DDX3X 3 , MYC p.P72S 2C.2 , SMARCA4 p.G1232S 2C.2 , TP53 p.S241P 2C.2 | 2C.2 |  | NL |  | NL | 2C.2 |
| SP59392 | Lymphoid | OTHER | GNA13 3 , MYC p.P75A 2C.2 , TP53 p.K139N 2C.2 | 2C.2 |  | NL |  | NL | 2C.2 |
| SP59396 | Lymphoid | OTHER | CARD11 2C.2 , CDC73 p.K188N 3 , FOXO1 p.T24I 3 , GNA13 p.R356Hfs*7 3 , MTOR p.C1483Y 2C.1 , SMARCA4 p.R973Q 2C.2 , TP53 p.R248Q 2C.2 | 2C.1 |  | NL |  | NL | 2C.1 |
| SP59400 | Lymphoid | OTHER | ATRX 2C.2 , MLL2 p.G4182Cfs*24 3 , PIK3R1 p.R562C 2C.2 | 2C.2 | CDKN2A loss 2C.1 | 2C.1 |  | NL | 2C.1 |
| SP59404 | Lymphoid | OTHER | SMARCA4 2C.2 , TP53 p.V218dupV 2C.2 , TP53 p.T284Rfs*21 2C.2 | 2C.2 |  | NL |  | NL | 2C.2 |
| SP59412 | Lymphoid | OTHER | B2M 2C.2 , CREBBP p.I340Cfs*9 2C.2 , IRF4 p.H56Y 3 , MLL2 p.V4740Sfs*57 3 , TP53 p.R273H 2C.2 , XPO1 p.E571K 2C.2 | 2C.2 |  | NL |  | NL | 2C.2 |
| SP59416 | Lymphoid | OTHER | ARID1A 2C.2 , CREBBP p.P704Qfs*9 2C.2 , EP300 p.Y1467D 2C.2 , EZH2 p.Y641S 2C.2 , MLL2 p.E4510Rfs*9 3 , STAT6 p.E372K 3 , TNFRSF14 p.K17* 3 | 2C.2 |  | NL |  | NL | 2C.2 |
| SP59420 | Lymphoid | OTHER | CREBBP 2C.2 , EZH2 p.Y641F 2C.2 | 2C.2 |  | NL |  | NL | 2C.2 |
| SP59428 | Lymphoid | OTHER |  | NL |  | NL |  | NL | NL |
| SP59432 | Lymphoid | OTHER | MLL2 3 , MLL2 p.Q3796* 3 | 3 |  | NL |  | NL | 3 |
| SP59436 | Lymphoid | OTHER | MLL3 3 | 3 |  | NL |  | NL | 3 |
| SP59440 | Lymphoid | OTHER |  | NL | B2M loss 2C.2 | 2C.2 |  | NL | 2C.2 |
| SP59444 | Lymphoid | OTHER | EZH2 2C.2 , FAT1 p.E183K 3 , MLL2 p.Q3959* 3 , MLL2 p.Q4636* 3 , SMARCA4 p.G782A 2C.2 | 2C.2 |  | NL |  | NL | 2C.2 |
| SP59448 | Lymphoid | OTHER | B2M 2C.2 , FAT1 p.G3495E 3 , GNA13 p.F272S 3 , IDH1 p.R132H 2C.1 , PTEN p.F21Wfs*24 2C.1 , SMARCA4 p.R973Q 2C.2 , SOCS1 p.Q180Hfs*425 3 , TNFAIP3 p.C779R 3 , TP53 p.C141Y 2C.2 , XPO1 p.E571G 2C.2 | 2C.1 |  | NL |  | NL | 2C.1 |
| SP59456 | Lymphoid | OTHER |  | NL |  | NL |  | NL | NL |
| SP59460 | Lymphoid | OTHER | ARID1A 2C.2 , EZH2 p.Y641F 2C.2 , MLL2 p.Q3585* 3 , MYD88 p.S219C 2C.2 | 2C.2 |  | NL |  | NL | 2C.2 |
| SP59464 | Lymphoid | OTHER | MLL2 3 | 3 |  | NL |  | NL | 3 |
| SP5980 | Breast | BREAST | BRCA2 1A.1 , CREBBP p.N797Kfs*35 2D , CTCF p.W191* 3 | 1A.1 | CDKN2A loss 2C.1 , CDKN2B loss 2D , MTAP loss 3 , NF1 loss 2C.1 , NOTCH3 loss 2C.2 , PTEN loss 2C.1 , RASA2 loss 3 | 2C.1 |  | NL | 1A.1 |
| SP59803 | Ovary | OVARY | TP53 2C.2 | 2C.2 | CCND2 amp 2C.2 , CHD4 amp 3 , FGF23 amp 3 | 2C.2 |  | NL | 2C.2 |
| SP59860 | Ovary | OVARY | MGA 3 | 3 | ALK amp 2C.1 , ARAF amp 2C.1 , CDK4 amp 2C.1 , KRAS amp 2C.1 , PIK3R2 amp 2C.2 , PRKACA amp 3 , RICTOR amp 2C.2 , XPO1 amp 2C.2 | 2C.1 |  | NL | 2C.1 |
| SP59938 | Ovary | OVARY | EPHA2 2C.2 , RASA2 p.R75H 3 | 2C.2 |  | NL | PTEN fusion 2C.1 | 2C.1 | 2C.1 |
| SP60322 | Ovary | OVARY | BRCA2 1A.1 | 1A.1 |  | NL |  | NL | 1A.1 |
| SP60610 | Ovary | OVARY | CHEK2 2C.1 , NF1 p.V2096Cfs*24 2C.1 | 2C.1 |  | NL |  | NL | 2C.1 |
| SP60842 | Ovary | OVARY | BRCA1 1A.1 , SUFU p.D69Y 2D , SUFU p.L68F 2D , TP53 p.R248G 2C.2 | 1A.1 | MYC amp 2C.2 | 2C.2 | RB1 fusion 2C.2 | 2C.2 | 1A.1 |
| SP6115 | Breast | BREAST | APC 2C.2 , BCL6 p.A562T 3 , NOTCH2 p.R2400* 2C.2 , TP53 p.R282W 2C.2 | 2C.2 | KDR amp 2D , KIT amp 2C.1 , PTEN loss 2C.1 | 2C.1 |  | NL | 2C.1 |
| SP61343 | Ovary | OVARY |  | NL |  | NL |  | NL | NL |
| SP61703 | Ovary | OVARY | TP53 2C.2 | 2C.2 | AKT2 amp 2C.2 , BCL2L12 amp 3 , CCNE1 amp 2C.2 , IL7R amp 3 , RICTOR amp 2C.2 | 2C.2 |  | NL | 2C.2 |
| SP6223 | Breast | BREAST | TP53 2C.2 | 2C.2 | FAM135B amp 3 , GATA2 amp 2C.2 , KRAS amp 2C.1 , MYC amp 2C.2 , SLCO1B3 amp 3 | 2C.1 |  | NL | 2C.1 |
| SP63716 | Ovary | OVARY | CDK12 2C.1 , TP53 p.K132E 2C.2 | 2C.1 | MECOM amp 3 , MYC amp 2C.2 | 2C.2 |  | NL | 2C.1 |
| SP63966 | Ovary | OVARY | NOTCH2 2C.2 , TP53 p.R273H 2C.2 | 2C.2 | AKT2 amp 2C.2 | 2C.2 |  | NL | 2C.2 |
| SP64036 | Ovary | OVARY | SETD2 2C.2 , TP53 p.V274G 2C.2 | 2C.2 | IGF1R amp 2D , SMC1A amp 3 | 2D |  | NL | 2C.2 |
| SP6429 | Breast | BREAST | FBXW7 2C.2 , TP53 p.R196* 2C.2 | 2C.2 | MYC amp 2C.2 , RAD51B loss 2C.1 , TERT amp 3 , ZNF217 amp 3 | 2C.1 |  | NL | 2C.1 |
| SP64546 | Ovary | OVARY | TP53 2C.2 | 2C.2 |  | NL |  | NL | 2C.2 |
| SP64976 | Ovary | OVARY | EPHA2 2C.2 , TP53 p.R248Q 2C.2 | 2C.2 | AKT1 amp 2C.2 | 2C.2 |  | NL | 2C.2 |
| SP6519 | Breast | BREAST |  | NL | ERBB2 amp 1A.1 , RARA amp 3 , RARA amp 3 | 1A.1 |  | NL | 1A.1 |
| SP65376 | Ovary | OVARY | CYLD 3 , PIK3CA p.Q546K 2C.1 | 2C.1 | MYC amp 2C.2 | 2C.2 |  | NL | 2C.1 |
| SP66687 | Ovary | OVARY | MAP2K4 2C.1 , MGA p.R2434W 3 , PPP2R1A p.R260C 2C.1 , TP53 p.Y220C 2C.2 | 2C.1 | MAP3K1 loss 2C.2 | 2C.2 |  | NL | 2C.1 |
| SP6673 | Breast | BREAST | EPHA2 2C.2 , JAK2 p.Y132* 2D , PIK3CA p.H1047R 1A.1 | 1A.1 |  | NL |  | NL | 1A.1 |
| SP66960 | Ovary | OVARY | TP53 2C.2 | 2C.2 | CCNE1 amp 2C.2 , IKBKB amp 3 | 2C.2 |  | NL | 2C.2 |
| SP6730 | Breast | BREAST | CDK4 2C.1 | 2C.1 | ERBB2 amp 1A.1 , MAP2K4 loss 2C.1 , RARA amp 3 | 1A.1 |  | NL | 1A.1 |
| SP67428 | Ovary | OVARY | FBXW7 2C.2 , NOTCH1 p.S2516F 2C.2 , RASA1 p.G700C 3 , TP53 p.E51Gfs*6 2C.2 | 2C.2 | PTEN loss 2C.1 , RB1 loss 2C.2 | 2C.1 |  | NL | 2C.1 |
| SP6766 | Breast | BREAST | AKT1 2C.2 , MLL3 p.L804Sfs*2 3 , NF1 p.R1250W 2C.1 | 2C.1 |  | NL |  | NL | 2C.1 |
| SP6813 | Breast | BREAST | TP53 2C.2 | 2C.2 | ERBB2 amp 1A.1 , ERBB2 amp 1A.1 , MCL1 amp 2D | 1A.1 | ERBB2 fusion 2C.1 | 2C.1 | 1A.1 |
| SP6825 | Breast | BREAST | HIST1H3B 3 , NCOR1 p.R1275* 3 , SPEN p.E800Gfs*21 3 | 3 | ERBB2 amp 1A.1 , ERBB2 amp 1A.1 , RARA amp 3 , RARA amp 3 , RPS6KB1 amp 3 , RPS6KB1 amp 3 | 1A.1 | ERBB2 fusion 2C.1 , NF1 fusion 2C.1 | 2C.1 | 1A.1 |
| SP68348 | Ovary | OVARY | APC 2C.2 , ARHGAP35 p.S985* 3 , ATR p.H942Y 2C.2 , CUL3 p.D92H 3 , TP53 p.V272M 2C.2 | 2C.2 |  | NL |  | NL | 2C.2 |
| SP68659 | Ovary | OVARY |  | NL |  | NL |  | NL | NL |
| SP68725 | Ovary | OVARY | TP53 2C.2 | 2C.2 |  | NL |  | NL | 2C.2 |
| SP7291 | Breast | BREAST | AKT1 2C.2 , CTCF . 3 , GATA3 p.P409Afs*99 2D , MAP2K4 p.Y160Ifs*14 2C.1 , TBX3 p.F219Ifs*8 2D | 2C.1 |  | NL |  | NL | 2C.1 |
| SP7378 | Breast | BREAST | PIK3CA 1A.1 | 1A.1 | AURKC amp 3 , H3F3B amp 3 , RPTOR amp 3 , RPTOR amp 3 , SRC amp 2C.2 | 2C.2 |  | NL | 1A.1 |
| SP7421 | Breast | BREAST | FANCD2 2C.1 , NOTCH2 p.P2297Lfs*9 2C.2 , PIK3CA p.E542K 1A.1 , TP53 p.S303Afs*42 2C.2 | 1A.1 |  | NL |  | NL | 1A.1 |
| SP7456 | Breast | BREAST | CDH1 2D , MLL3 p.S4247*fs*1 3 , PIK3CA p.H1047R 1A.1 | 1A.1 | CCND1 amp 2C.2 , CCND1 amp 2C.2 , FGF19 amp 2D , FGF3 amp 2D , FGF4 amp 2D , MDM4 amp 2D , PIK3C2B amp 3 | 2C.2 |  | NL | 1A.1 |
| SP7692 | Breast | BREAST | MLL 3 , PIK3CA p.G118D 1A.1 , SPEN p.E2846K 3 , SPEN p.K2833N 3 , SPEN p.Q2822H 3 , SPEN p.E2212K 3 , TP53 p.Y234C 2C.2 | 1A.1 | AR amp 2C.2 , ERBB2 amp 1A.1 | 1A.1 |  | NL | 1A.1 |
| SP7785 | Breast | BREAST | MLL3 3 , MSH3 p.H729Q 2C.2 , RB1 p.A74Efs*4 2C.2 , SDHA p.R451H 2D , TP53 p.T125T 2C.2 | 2C.2 | ARID2 loss 3 , CDKN2A loss 2C.1 , CDKN2B loss 2D | 2C.1 |  | NL | 2C.1 |
| SP79907 | Prostate | PROSTATE |  | NL |  | NL |  | NL | NL |
| SP79939 | Prostate | PROSTATE |  | NL |  | NL | ETV4 fusion 2C.2 | 2C.2 | 2C.2 |
| SP79958 | Prostate | PROSTATE |  | NL |  | NL |  | NL | NL |
| SP79968 | Prostate | PROSTATE | ERCC2 2C.2 , SPOP p.W131G 3 | 2C.2 | APC loss 2C.2 | 2C.2 |  | NL | 2C.2 |
| SP79971 | Prostate | PROSTATE | KRAS 2C.1 | 2C.1 | MAP3K1 loss 2C.2 | 2C.2 | ETV4 fusion 2C.2 | 2C.2 | 2C.1 |
| SP79988 | Prostate | PROSTATE |  | NL |  | NL |  | NL | NL |
| SP79998 | Prostate | PROSTATE |  | NL |  | NL | ERG fusion 2D | 2D | 2D |
| SP80014 | Prostate | PROSTATE |  | NL | PTEN loss 2C.1 | 2C.1 | ERG fusion 2D | 2D | 2C.1 |
| SP80037 | Prostate | PROSTATE | ACVR1B 3 , NCOR1 p.N1657Qfs*57 3 | 3 | ASXL2 loss 3 , RNASEH2B loss 3 | 3 | ERG fusion 2D | 2D | 2D |
| SP80042 | Prostate | PROSTATE | AXIN1 2C.2 | 2C.2 |  | NL |  | NL | 2C.2 |
| SP80160 | Prostate | PROSTATE |  | NL |  | NL | ERG fusion 2D | 2D | 2D |
| SP80183 | Prostate | PROSTATE |  | NL |  | NL | ERG fusion 2D | 2D | 2D |
| SP80205 | Prostate | PROSTATE |  | NL |  | NL |  | NL | NL |
| SP80213 | Prostate | PROSTATE |  | NL |  | NL | ERG fusion 2D | 2D | 2D |
| SP80216 | Prostate | PROSTATE |  | NL |  | NL |  | NL | NL |
| SP80217 | Prostate | PROSTATE |  | NL |  | NL |  | NL | NL |
| SP80244 | Prostate | PROSTATE |  | NL |  | NL | ERG fusion 2D | 2D | 2D |
| SP80271 | Colon/Rectum | COLON/RECTUM | KRAS 1A.1 , KRAS p.G12V 1A.1 , PMS2 p.M362I 1B , SOX9 p.I480Pfs*44 3 , TP53 p.P151H 2C.2 | 1A.1 | AURKA amp 3 , GNAS amp 2C.2 , PLCG1 amp 3 , SRC amp 2C.2 , TOP1 amp 2C.2 , ZNF217 amp 3 | 2C.2 |  | NL | 1A.1 |
| SP80367 | Colon/Rectum | COLON/RECTUM | APC 2C.2 , SMAD2 p.P305L 3 , TP53 p.R335Qfs*2 2C.2 | 2C.2 |  | NL |  | NL | 2C.2 |
| SP80423 | Colon/Rectum | COLON/RECTUM | APC 2C.2 , PIK3CA p.E545K 2C.1 , TP53 p.Q331* 2C.2 | 2C.1 | AURKA amp 3 , GNAS amp 2C.2 , SRC amp 2C.2 , ZNF217 amp 3 | 2C.2 |  | NL | 2C.1 |
| SP80615 | Colon/Rectum | COLON/RECTUM | ABL1 2C.1 , ACVR2A p.R478I 3 , ACVR2A p.E232* 3 , ACVR2A p.E214K 3 , ACVR2A p.G77D 3 , APC p.R2237* 2C.2 , APC p.R1114* 2C.2 , APC p.R2204* 2C.2 , APC p.S2026Y 2C.2 , APC p.S1400* 2C.2 , ARHGAP35 p.R997* 3 , ARID1A p.R1989* 2C.2 , ARID1B p.G1611A 2D , ARID2 p.R1414I 3 , ARID5B p.R785H 3 , ARID5B p.T354I 3 , ATM p.L1408I 2C.1 , ATM p.R250* 2C.1 , ATR p.E969* 2C.2 , ATR p.L2393R 2C.2 , ATRX p.R1665I 2C.2 , BLM p.S1252Y 2C.1 , BRCA1 p.E572* 2C.1 , BRCA2 p.L3180R 2C.1 , BRCA2 p.K2868N 2C.1 , BRCA2 p.S2670L 2C.1 , BRCA2 p.R2494Q 2C.1 , CARD11 p.F91L 3 , CDC73 p.E302* 3 , CDH1 p.D221A 2D , CDH1 p.E210* 2D , CDK12 p.R890H 2C.1 , CDK12 p.K837N 2C.1 , CDK4 p.R101M 2C.1 , CDKN1A p.E56K 2D , CTCF p.R603C 3 , CTCF p.E7K 3 , CYLD p.T778K 3 , DICER1 p.E382K 3 , DICER1 p.R676Q 3 , DICER1 p.R944Q 3 , DNMT3A p.G822D 3 , EP300 p.R568W 2D , ERBB4 p.E937* 2C.2 , ERCC4 p.R267C 2C.2 , ESR1 p.E353G 2C.2 , ESR1 p.E247K 2C.2 , FANCI p.R533Q 2C.1 , FANCI p.R321I 2C.1 , FAT1 p.E1671* 3 , FAT1 p.W2868C 3 , FAT1 p.F3590L 3 , FAT1 p.K4335R 3 , FBXW7 p.R465H 2C.2 , FGFR2 p.E732K 2C.1 , FUBP1 p.E155* 3 , GATA3 p.R306Q 2D , HDAC2 p.S198* 3 , IDH2 p.G421S 2C.1 , IKBKB p.I164S 3 , JAK1 p.G948R 2D , JAK2 p.K414N 2D , JAK3 p.I872S 2D , KDM5C p.D87N 3 , KDM5C p.R332* 3 , KDR p.D1064N 2D , KRAS p.A146T 1A.1 , MAP2K4 p.E352* 2C.1 , MET p.R1188* 2C.1 , MGA p.R2650Q 3 , MGA p.R1016* 3 , MLL p.R2163Q 3 , MLL2 p.A3498V 3 , MLL2 p.R5282* 3 , MLL3 p.R56Q 3 , MLL3 p.R3995* 3 , MSH3 p.I1082M 2C.2 , MSH3 p.K122N 2C.2 , NBN p.F316L 2C.1 , NBN p.E383* 2C.1 , NCOR1 p.F254C 3 , NCOR1 p.A703T 3 , NF1 p.L2367Q 2C.1 , NF1 p.E1266* 2C.1 , NOTCH1 p.A2101T 2C.2 , NOTCH2 p.A1654T 2C.2 , PBRM1 p.K414N 2D , PBRM1 p.R461C 2D , PBRM1 p.R876C 2D , PBRM1 p.R1160* 2D , PDGFRA p.D681Y 1A.1 , PDGFRA p.L542R 1A.1 , PIK3R1 p.R348* 2C.2 , PMS2 p.S274L 1B , POLE p.P286R 2C.1 , PPM1D p.R528I 3 , PPP6C p.K282T 3 , PTEN p.E299* 2C.1 , PTEN p.E7* 2C.1 , RAD54L p.K302N 2C.1 , RAF1 p.L476I 2C.2 , RASA1 p.E429* 3 , RASA2 p.R536S 3 , RB1 p.R876C 2C.2 , RB1 p.E322K 2C.2 , RB1 p.E54* 2C.2 , RNF43 p.F279L 2C.2 , RUNX1 p.R320* 3 , SETD2 p.R53Q 2D , SETD2 p.D350Y 2D , SMARCA4 p.R1336H 2C.2 , SMARCA4 p.D1169Y 2C.2 , SPEN p.E2584* 3 , SPEN p.E1043* 3 , SPEN p.R637Q 3 , TET2 p.E783* 2D , TP53 p.R213* 2C.2 , TPMT p.I204T 3 , TSC1 p.K167N 2C.1 , TSC1 p.M223I 2C.1 , VHL p.D197Y 2C.2 , ZFHX3 p.R2246I 3 | 1A.1 |  | NL |  | NL | 1A.1 |
| SP80657 | Colon/Rectum | COLON/RECTUM | APC 2C.2 , APC p.S1042Mfs*6 2C.2 , KRAS p.A146T 1A.1 , PCBP1 p.L100Q 3 , PIK3CA p.V344G 2C.1 , PTEN p.Y68N 2C.1 , SMAD4 p.P356L 2C.2 | 1A.1 |  | NL |  | NL | 1A.1 |
| SP80754 | Colon/Rectum | COLON/RECTUM | APC 2C.2 , APC p.N1026Ifs*11 2C.2 , ATM p.E2039K 2C.1 , ATM p.D1682Y 2C.1 , FBXW7 p.R505C 2C.2 , KRAS p.K117N 1A.1 , SMAD4 p.S325Yfs*5 2C.2 , TP53 p.G262V 2C.2 | 1A.1 | FGF9 amp 2D , FLT3 amp 2C.1 , KLF5 amp 3 , MAP2K4 loss 2C.1 | 2C.1 |  | NL | 1A.1 |
| SP8085 | Breast | BREAST | PIK3CA 1A.1 , TP53 p.N131Tfs*39 2C.2 | 1A.1 | ESR1 amp 2C.2 , MYC amp 2C.2 , NFE2L2 amp 2C.2 , PPP6C amp 3 , RAF1 amp 2C.2 | 2C.2 | ESR1 fusion 2C.1 | 2C.1 | 1A.1 |
| SP80950 | Colon/Rectum | COLON/RECTUM | APC 2C.2 | 2C.2 | PLCG1 amp 3 , SRC amp 2C.2 , TOP1 amp 2C.2 | 2C.2 |  | NL | 2C.2 |
| SP81137 | Colon/Rectum | COLON/RECTUM | APC 2C.2 , TP53 p.G226Rfs*23 2C.2 | 2C.2 | KLF5 amp 3 , SRC amp 2C.2 , ZNF217 amp 3 | 2C.2 |  | NL | 2C.2 |
| SP81440 | Colon/Rectum | COLON/RECTUM | APC 2C.2 , APC p.E1322* 2C.2 , ARID2 p.D174Y 3 , KRAS p.G12C 1A.1 , TP53 p.R248W 2C.2 | 1A.1 | AURKA amp 3 , FLT3 amp 2C.1 , GNAS amp 2C.2 , PLCG1 amp 3 , SRC amp 2C.2 , TOP1 amp 2C.2 , ZNF217 amp 3 | 2C.1 |  | NL | 1A.1 |
| SP81494 | Colon/Rectum | COLON/RECTUM | APC 2C.2 , ATRX p.D2213Y 2C.2 , EPHA2 p.E765G 2C.2 , KRAS p.G12D 1A.1 , NF2 p.R516L 2C.1 , NF2 p.R516W 2C.1 , NRAS p.G12C 1.A.1 , PCBP1 p.L100Q 3 , SETD2 p.R2399* 2D , TP53 p.I251F 2C.2 , TSHR p.P639Q 3 | 1.A.1 | AURKA amp 3 , GNAS amp 2C.2 , PLCG1 amp 3 , SRC amp 2C.2 , TOP1 amp 2C.2 , ZNF217 amp 3 | 2C.2 |  | NL | 1.A.1 |
| SP8157 | Breast | BREAST |  | NL | CCNE1 amp 2C.2 , ESR1 loss 2C.2 , FGFR1 amp 2C.1 , IKBKB amp 3 , IKBKB amp 3 , ZNF429 loss 3 | 2C.1 |  | NL | 2C.1 |
| SP81711 | Colon/Rectum | COLON/RECTUM | APC 2C.2 , APC p.Q1303* 2C.2 , CHD4 p.S1158I 3 , SMARCA4 p.I996F 2C.2 , TP53 p.S127P 2C.2 | 2C.2 | AURKA amp 3 , GNAS amp 2C.2 , PLCG1 amp 3 , SRC amp 2C.2 , TOP1 amp 2C.2 , ZNF217 amp 3 | 2C.2 |  | NL | 2C.2 |
| SP81840 | Colon/Rectum | COLON/RECTUM | APC 2C.2 , ATM p.R250* 2C.1 , KRAS p.G13D 1A.1 , MAP2K4 p.R134W 2C.1 | 1A.1 |  | NL |  | NL | 1A.1 |
| SP82087 | Colon/Rectum | COLON/RECTUM | APC 2C.2 , AR p.F828L 2C.2 , FAM123B p.R626* 3 , KRAS p.G12C 1A.1 , SETD2 p.R404I 2D , TP53 p.A159V 2C.2 | 1A.1 |  | NL |  | NL | 1A.1 |
| SP82103 | Colon/Rectum | COLON/RECTUM | APC 2C.2 , ARID1A p.G1729Wfs*7 2C.2 , RASA1 p.S962Tfs*6 3 , RASA1 p.R285* 3 | 2C.2 |  | NL |  | NL | 2C.2 |
| SP8229 | Breast | BREAST | TP53 2C.2 | 2C.2 | FAM135B amp 3 , FAM135B amp 3 , FGFR1 amp 2C.1 , PXDNL amp 3 , PXDNL amp 3 | 2C.1 |  | NL | 2C.1 |
| SP82399 | Skin | SKIN | BRAF 1A.1 , CDKN2A p.P81L 2C.1 | 1A.1 | CDKN2A loss 2C.1 , CDKN2B loss 2D | 2C.1 |  | NL | 1A.1 |
| SP82417 | Skin | SKIN | BRAF 1A.1 , BRAF p.V600E 1A.1 , BRCA1 p.E272K 2C.1 , DDX3X p.Y200S 3 , DNMT3A p.G685E 3 , EPHA2 p.D942N 2C.2 , HDAC9 p.G167R 3 , PPP6C p.R301C 3 , SF3B1 p.R625C 3 , SMARCA4 p.P1536L 2C.2 | 1A.1 |  | NL |  | NL | 1A.1 |
| SP82429 | Skin | SKIN | BRAF 1A.1 , MGA p.P1451L 3 | 1A.1 | CARD11 amp 3 , EGFR amp 2C.1 , GLI3 amp 3 , RAC1 amp 3 | 2C.1 |  | NL | 1A.1 |
| SP82431 | Skin | SKIN | APC 2C.2 , ATR p.D1298N 2C.2 , BRAF p.V600E 1A.1 , CBFB p.R147W 3 , CDKN2A p.R58Afs*57 2C.1 , CIC p.P1177L 3 , FAT1 p.S4291F 3 , MAP2K1 p.P124S 2C.1 , MEN1 p.W188* 2C.2 , PIK3R1 p.P89L 2C.2 , POLE p.R2145* 2C.1 , RAC1 p.P29S 3 , SMARCA4 p.E1435G 2C.2 , TCF7L2 p.P218L 3 , TRRAP p.S722F 3 | 1A.1 |  | NL |  | NL | 1A.1 |
| SP82433 | Skin | SKIN | BRAF 1A.1 , BRAF p.V600E 1A.1 , MECOM p.G794E 3 , PTEN p.V166Sfs*14 2C.1 | 1A.1 |  | NL |  | NL | 1A.1 |
| SP82435 | Skin | SKIN | BRAF 1A.1 , CDK6 p.P199L 2C.1 , DPYD p.N120S 3 | 1A.1 | CDKN2A loss 2C.1 , CDKN2B loss 2D , MTAP loss 3 , PTEN loss 2C.1 | 2C.1 |  | NL | 1A.1 |
| SP82445 | Skin | SKIN | ABCB1 2D , ABCB1 p.G390E 2D , ABCB1 p.S943F 2D , ARID2 p.K1366* 3 , DPYD p.R43K 3 , EPHA2 p.E607K 2C.2 , FBXW7 p.S601F 2C.2 , HDAC9 p.P15S 3 , MET p.L1158F 2C.1 , MLL3 p.R4145C 3 , MSH3 p.P740S 2C.2 , NF1 p.R2517* 2C.1 , RAC1 p.P34S 3 , SUFU p.R123C 2D , TP53 p.A159V 2C.2 , TP53 p.R290C 2C.2 , WT1 p.T314I 2C.2 | 2C.1 |  | NL |  | NL | 2C.1 |
| SP82451 | Skin | SKIN | BRAF 1A.1 , PPP6C p.G149E 3 , TBX3 p.P42H 2D | 1A.1 | ABCB1 amp 3 , BRAF amp 2C.1 , CDK6 amp 2C.1 , EZH2 amp 2C.2 , MET amp 2C.1 , MITF amp 2c,2 , RHEB amp 3 , SMO amp 2C.1 | 2c,2 |  | NL | 1A.1 |
| SP82459 | Skin | SKIN | ABL2 3 , BRAF p.L597Q 2C.1 , MAP2K4 p.L146F 2C.1 , POLE p.L607F 2C.1 | 2C.1 |  | NL |  | NL | 2C.1 |
| SP82461 | Skin | SKIN | ARID2 3 , BRAF p.K601E 1A.1 , KDR p.G493R 2D , MLL2 p.M1360Sfs*16 3 , MLL2 p.M1360Sfs*16 3 , MLL2 p.P3423L 3 , NOTCH2 p.K2127* 2C.2 , PBRM1 p.Y85* 2D , SMARCA4 p.H884Y 2C.2 | 1A.1 | FAM135B amp 3 | 3 |  | NL | 1A.1 |
| SP82471 | Skin | SKIN | FANCD2 2C.1 , NRAS p.G12R 2C.1 | 2C.1 | CDK4 amp 2C.1 , KRAS amp 2C.1 , PXDNL amp 3 , STAT6 amp 3 | 2C.1 |  | NL | 2C.1 |
| SP82532 | Skin | SKIN | AXIN1 2C.2 , CDH1 p.P429L 2D , DDX3X p.S412F 3 , DPYD p.P545L 3 , ERBB4 p.P759L 2C.2 , GATA2 p.G272R 2C.2 , KDR p.G345R 2D , KDR p.G345E 2D , KRAS p.G12D 2C.1 , PRDM1 p.P300L 3 , RAC1 p.P106L 3 | 2C.1 | CCND1 amp 2C.2 , FGF19 amp 2D , FGF4 amp 2D , RAC1 amp 3 | 2C.2 |  | NL | 2C.1 |
| SP82614 | Skin | SKIN | BRAF 1A.1 , FBXW7 p.H500Y 2C.2 , SPOP p.F199Y 3 , STAT6 p.P617L 3 | 1A.1 |  | NL |  | NL | 1A.1 |
| SP82636 | Skin | SKIN | KIT 1B | 1B | CDK4 amp 2C.1 | 2C.1 |  | NL | 1B |
| SP82644 | Skin | SKIN | CYLD 3 , DPYD p.S204F 3 , EGFR p.P622S 2C.1 , FAM123B p.Q979* 3 , PBRM1 p.K717* 2D | 2C.1 | CDKN2A loss 2C.1 , CDKN2B loss 2D | 2C.1 | RAF1 fusion 2C.1 | 2C.1 | 2C.1 |
| SP82756 | Skin | SKIN |  | NL |  | NL | RAF1 fusion 2C.1 | 2C.1 | 2C.1 |
| SP82780 | Skin | SKIN | CDKN2A 2C.1 , HDAC9 p.G928E 3 , NRAS p.Q61K 2C.1 | 2C.1 |  | NL |  | NL | 2C.1 |
| SP82796 | Skin | SKIN | NRAS 2C.1 , NRAS p.Q61R 2C.1 | 2C.1 | CDKN2A loss 2C.1 , CDKN2B loss 2D , DDR1 amp 3 , MTAP loss 3 , TPMT amp 3 | 2C.1 |  | NL | 2C.1 |
| SP82836 | Skin | SKIN | AKT1 2C.2 , ARID2 . 3 , BRAF p.V600E 1A.1 , NOTCH2 p.P690S 2C.2 , PTEN p.L108P 2C.1 | 1A.1 |  | NL |  | NL | 1A.1 |
| SP82900 | Skin | SKIN | CARD11 3 , CARD11 p.K356N 3 , CDKN2A p.P48L 2C.1 , FANCI p.S708F 2C.1 , FGFR2 p.E696K 2C.1 , JAK3 p.D912N 2D , NRAS p.Q61K 2C.1 , SETBP1 p.E599K 3 , TET2 p.E1490K 2D , TP53 p.R280K 2C.2 | 2C.1 |  | NL |  | NL | 2C.1 |
| SP82988 | Skin | SKIN | ARID2 3 , ATRX p.R2153C 2C.2 , IDH1 p.R132C 2C.1 , NF1 p.R1362* 2C.1 , NRAS p.Q61L 2C.1 , POT1 p.H245Y 3 , RAC1 p.P29S 3 | 2C.1 |  | NL |  | NL | 2C.1 |
| SP83019 | Skin | SKIN | BRAF 1A.1 , BRAF p.V600E 1A.1 , MLL p.P280L 3 , NF1 p.S2496F 2C.1 , SPEN p.P2541L 3 , STK11 p.K296Rfs*40 2C.1 , TRRAP p.S722F 3 | 1A.1 |  | NL |  | NL | 1A.1 |
| SP83027 | Skin | SKIN | RAC1 3 | 3 | EZH2 amp 2C.2 , FAM135B amp 3 , MDM4 amp 2D , MYC amp 2C.2 , PIK3C2B amp 3 , PXDNL amp 3 | 2C.2 | BRAF fusion 2C.1 | 2C.1 | 2C.1 |
| SP83083 | Skin | SKIN | KDM5C 3 , NRAS p.Q61K 2C.1 | 2C.1 |  | NL |  | NL | 2C.1 |
| SP83099 | Skin | SKIN | BRAF 1A.1 | 1A.1 | TSC1 loss 2C.1 | 2C.1 |  | NL | 1A.1 |
| SP83146 | Skin | SKIN | ARID2 3 , EP300 p.Q852* 2D , KDM6A p.P1107S 2D , MEN1 p.M239I 2C.2 , MGA p.T2606I 3 , NF1 p.L604* 2C.1 , RHEB p.L137F 3 , SDHA p.T405I 2C.2 , TBX3 p.A562T 2D , TP53 p.Q317* 2C.2 , TSHR p.F286Y 3 | 2C.1 |  | NL |  | NL | 2C.1 |
| SP83242 | Skin | SKIN | BRAF 1A.1 , CTNNB1 p.C429G 2C.2 , NOTCH1 p.P362L 2C.2 , POLR2B p.D617N 3 | 1A.1 | CDKN2A loss 2C.1 , CDKN2B loss 2D , MTAP loss 3 | 2C.1 |  | NL | 1A.1 |
| SP83312 | Skin | SKIN | BRAF 1A.1 , CDKN2A p.R80* 2C.1 , TCF7L2 p.P254L 3 | 1A.1 | MCL1 amp 2D | 2D |  | NL | 1A.1 |
| SP83382 | Skin | SKIN | ATM 2C.1 , DDX3X p.Q225* 3 , FUBP1 p.S270L 3 , NRAS p.Q61K 2C.1 , PTCH1 p.P681L 2C.1 , PTEN p.E256* 2C.1 , SPEN p.E845K 3 | 2C.1 |  | NL |  | NL | 2C.1 |
| SP83482 | Skin | SKIN | APC 2C.2 , BRAF p.V600M 1A.1 , BRAF p.V600E 1A.1 , MAP3K1 p.R45W 2C.2 , MLH3 p.L1316F 2C.2 , TP53 p.R342* 2C.2 | 1A.1 |  | NL |  | NL | 1A.1 |
| SP83844 | Skin | SKIN | BRAF 1A.1 , FLT3 p.S941L 2C.1 , HDAC9 p.G957R 3 , KDR p.D1052N 2D , TP53 p.K120E 2C.2 | 1A.1 | IDH2 amp 2C.1 , MAP2K1 amp 2C.1 , MITF amp 2c,2 , RAC1 amp 3 | 2c,2 |  | NL | 1A.1 |
| SP8394 | Breast | BREAST | PIK3CA 1A.1 , TP53 p.Q331* 2C.2 | 1A.1 | EGFR amp 2C.1 , ERBB2 amp 1A.1 | 1A.1 |  | NL | 1A.1 |
| SP83967 | Stomach | OTHER | ARID1A 2C.2 , ARID1A p.Q1512* 2C.2 , HDAC2 p.H282Y 3 , KDM6A p.S1028F 2C.2 , PIK3CA p.E542K 2C.1 | 2C.1 | MDM2 amp 2C.2 | 2C.2 |  | NL | 2C.1 |
| SP84056 | Stomach | OTHER | CDKN2A 2C.1 | 2C.1 | SMAD4 loss 2C.2 | 2C.2 |  | NL | 2C.1 |
| SP84062 | Stomach | OTHER | CDH1 2D , SMAD4 p.R361_L364delRFCL 2C.2 | 2C.2 | CDK6 amp 2C.1 | 2C.1 |  | NL | 2C.1 |
| SP84392 | Stomach | OTHER | ACVR2A 3 , ACVR2A p.K437Rfs*5 3 , ARHGAP35 p.R44H 3 , ARID1A p.T294Pfs*69 2C.2 , ASXL1 p.G645Vfs*58 2C.2 , ASXL1 p.V172Cfs*81 2C.2 , ATR p.R1814Efs*10 2C.2 , BRCA1 p.R1397M 2C.1 , CDK12 p.G923V 2C.1 , CIC p.R353Pfs*54 2D , DPYD p.F707Lfs*4 3 , EP300 p.S800Ffs*38 2C.2 , FANCA p.K453N 2C.1 , FLT3 p.F691Lfs*13 2C.1 , FLT4 p.T168M 2C.2 , HLA-B p.P209Qfs*5 3 , JAK1 p.K142Rfs*26 2D , JAK1 p.K860Nfs*16 2D , KDM5C p.R634H 3 , KRAS p.G13D 2C.1 , MLL p.R1686C 3 , MLL2 p.S102Afs*28 3 , MLL2 p.G1235Vfs*95 3 , MRE11A p.E10G 2C.1 , NF1 p.I679Dfs*21 2C.1 , NOTCH1 p.R1937H 2C.2 , PIK3CA p.H1047R 2C.1 , RNF43 p.G659Vfs*41 2C.2 , STAG2 p.C176Vfs*7 3 | 2C.1 |  | NL |  | NL | 2C.1 |
| SP84408 | Stomach | OTHER | ARID1A 2C.2 , ARID1A p.E33Gfs*62 2C.2 , RHOA p.F39C 3 , SMAD4 p.C363Y 2C.2 | 2C.2 | CDKN2A loss 2C.1 , CDKN2B loss 2C.2 , MTAP loss 3 | 2C.1 |  | NL | 2C.1 |
| SP84439 | Stomach | OTHER | ACVR2A 3 , APC p.E2550V 2C.2 , ARID1A p.D1850Tfs*33 2C.2 , ARID2 p.D367V 3 , ATM p.R270M 2C.1 , BRCA2 p.T3033Lfs*29 2C.1 , CIC p.P111Hfs*94 2D , DNMT3A p.G646W 3 , DNMT3A p.F751I 3 , EPHA2 p.G240Vfs*153 2C.2 , EPHA2 p.P460Rfs*33 2C.2 , ERCC2 p.R293Q 2C.2 , ERCC2 p.A440T 2C.2 , FANCA p.A786V 2C.1 , FAT1 p.K3889T 3 , FGFR2 p.D656G 2C.1 , FLT4 p.L546F 2C.2 , FUBP1 p.Y514* 3 , INPP4B p.V524M 3 , JAK1 p.P430Rfs*2 2D , JAK1 p.K860Nfs*16 2D , JAK3 p.D813N 2C.2 , KDM5C p.H632Y 3 , KEAP1 p.S45F 2C.2 , MGA p.L1606*fs*1 3 , MGA p.Q1433* 3 , MLL p.F1458S 3 , MLL2 p.R2683H 3 , MLL3 p.D3174V 3 , MLL3 p.P3277Hfs*4 3 , MLL3 p.S3618T 3 , MRE11A p.R364* 2C.1 , NF2 p.C133Y 2C.1 , NOTCH1 p.G317C 2C.2 , NUP93 p.L26P 3 , PBRM1 p.P1411Lfs*21 2D , PIK3CA p.H1047R 2C.1 , PIK3CA p.R412Q 2C.1 , POLE p.V474I 2C.1 , POLE p.R1111Q 2C.1 , POLE p.Y1889C 2C.1 , PRDM1 p.E247Vfs*23 3 , PTCH1 p.L590F 2C.1 , PTCH1 p.A918T 2C.1 , PTEN p.I33delI 2C.1 , RNF43 p.G659Vfs*41 2C.2 , SDHA p.V406M 2C.2 , SMARCA4 p.A1002V 2C.2 , SMARCA4 p.G630D 2C.2 , STAG2 p.R110* 3 , TGFBR2 p.R279H 2D , TGFBR2 p.D128G 2D , ZFHX3 p.Y293* 3 | 2C.1 |  | NL |  | NL | 2C.1 |
| SP84491 | Stomach | OTHER | CTNNB1 2C.2 , CTNNB1 p.T339N 2C.2 , TP53 p.T284Kfs*61 2C.2 | 2C.2 | FAT1 loss 3 , MAP2K4 loss 2C.1 | 2C.1 |  | NL | 2C.1 |
| SP84719 | Stomach | OTHER | ARID2 3 , ARID2 p.Q1096* 3 , CDH1 p.V188D 2D , PIK3CA p.E542K 2C.1 , SMARCA4 p.G782S 2C.2 , SOX9 p.R160Pfs*92 3 | 2C.1 |  | NL |  | NL | 2C.1 |
| SP84743 | Stomach | OTHER | APC 2C.2 , ATP6AP2 p.R275S 3 , PIK3R1 p.M206Ifs*4 2C.2 , PTEN p.L42P 2C.1 , ROS1 p.C2067G 2C.1 , TP53 p.R273H 2C.2 | 2C.1 | CCND3 amp 2C.2 , CCNE1 amp 2C.2 | 2C.2 |  | NL | 2C.1 |
| SP84858 | Stomach | OTHER | JAK2 2D , KRAS p.G12C 2C.1 , PIK3CA p.E545K 2C.1 , RASA1 p.R711* 3 , SMAD4 p.C499Y 2C.2 , SMAD4 p.Y412Lfs*17 2C.2 , TP53 p.R175H 2C.2 | 2C.1 | CDKN2A loss 2C.1 , CDKN2B loss 2C.2 , ERBB2 amp 1A.1 , MTAP loss 3 , RARA amp 3 , RARA amp 3 | 1A.1 | ERBB2 fusion 2C.1 | 2C.1 | 1A.1 |
| SP84962 | Stomach | OTHER | ARID1A 2C.2 , TP53 p.H193R 2C.2 | 2C.2 | MCL1 amp 2D , TGFBR2 loss 2D | 2D |  | NL | 2C.2 |
| SP84982 | Stomach | OTHER | ACVR2A 3 , ARID1B p.S736Ifs*27 2D , BAP1 p.E198Rfs*33 2C.1 , BLM p.N515Mfs*16 2C.1 , CASP8 p.G477* 2D , FLT4 p.A862T 2C.2 , INPP4B p.R874L 3 , KRAS p.G13D 2C.1 , MAP3K1 p.S1330L 2C.2 , SETD2 p.T1652Yfs*14 2D | 2C.1 |  | NL |  | NL | 2C.1 |
| SP84998 | Stomach | OTHER | ARID1B 2D , CHD4 p.R1338I 3 , MGA p.R627* 3 | 2D |  | NL |  | NL | 2D |
| SP85130 | Stomach | OTHER | APC 2C.2 | 2C.2 | CDKN2A loss 2C.1 , CDKN2B loss 2C.2 , MTAP loss 3 | 2C.1 |  | NL | 2C.1 |
| SP85222 | Stomach | OTHER | CDKN2A 2C.1 , SMAD4 p.R361_G365delRFCLG 2C.2 , TP53 p.R248Q 2C.2 | 2C.1 | CCND1 amp 2C.2 , CDK6 amp 2C.1 , ERBB2 amp 1A.1 , ERBB2 amp 1A.1 , FGF19 amp 2D , FGF3 amp 2D , FGF4 amp 2D , IKBKB amp 3 | 1A.1 | ERBB2 fusion 2C.1 | 2C.1 | 1A.1 |
| SP85230 | Stomach | OTHER | BRCA1 2C.1 , MLL2 p.E1682* 3 , PIK3C2B p.W645S 3 | 2C.1 | PTEN loss 2C.1 | 2C.1 |  | NL | 2C.1 |
| SP85251 | Stomach | OTHER | PMS1 2C.2 , TP53 p.H214R 2C.2 | 2C.2 | KRAS amp 2C.1 | 2C.1 |  | NL | 2C.1 |
| SP8532 | Breast | BREAST | ABL2 3 , CDK12 p.R902P 2C.1 , MLL3 p.L1217Rfs*7 3 , TP53 p.I255delI 2C.2 | 2C.1 | CDH1 loss 2D , FGFR1 amp 2C.1 , IKBKB amp 3 , MAPK1 amp 2C.2 | 2C.1 |  | NL | 2C.1 |
| SP85339 | Stomach | OTHER | ACVR1B 3 , CYLD p.E259K 3 , MEN1 p.Q266* 2C.2 , MEN1 p.D423Y 2C.2 , TP53 p.R282W 2C.2 | 2C.2 | ARID2 loss 3 | 3 |  | NL | 2C.2 |
| SP85379 | Stomach | OTHER | BCOR 3 , CDK12 p.R779H 2C.1 , SMARCA4 p.R468H 2C.2 | 2C.1 | CDKN2A loss 2C.1 , IKBKB amp 3 , MYC amp 2C.2 | 2C.1 |  | NL | 2C.1 |
| SP85487 | Thyroid | OTHER | NRAS 2C.1 | 2C.1 |  | NL |  | NL | 2C.1 |
| SP85491 | Thyroid | OTHER |  | NL |  | NL | RET fusion 2C.1 | 2C.1 | 2C.1 |
| SP85495 | Thyroid | OTHER | BRAF 1A.1 | 1A.1 |  | NL |  | NL | 1A.1 |
| SP85511 | Thyroid | OTHER | BRAF 1A.1 | 1A.1 |  | NL |  | NL | 1A.1 |
| SP85582 | Thyroid | OTHER | BRAF 1A.1 | 1A.1 |  | NL |  | NL | 1A.1 |
| SP85623 | Thyroid | OTHER |  | NL |  | NL | RET fusion 2C.1 | 2C.1 | 2C.1 |
| SP8564 | Breast | BREAST | TNFAIP3 3 | 3 | ERBB2 amp 1A.1 , MCL1 amp 2D , RIT1 amp 3 , RPS6KB1 amp 3 | 1A.1 | ESR1 fusion 2C.1 | 2C.1 | 1A.1 |
| SP85647 | Thyroid | OTHER |  | NL |  | NL |  | NL | NL |
| SP85725 | Thyroid | OTHER |  | NL |  | NL |  | NL | NL |
| SP85733 | Thyroid | OTHER |  | NL |  | NL |  | NL | NL |
| SP85787 | Thyroid | OTHER |  | NL |  | NL |  | NL | NL |
| SP85818 | Thyroid | OTHER |  | NL |  | NL |  | NL | NL |
| SP85836 | Thyroid | OTHER |  | NL |  | NL |  | NL | NL |
| SP85840 | Thyroid | OTHER |  | NL |  | NL |  | NL | NL |
| SP85864 | Thyroid | OTHER | BRAF 1A.1 , NOTCH2 p.C877Y 2C.2 | 1A.1 |  | NL |  | NL | 1A.1 |
| SP85952 | Thyroid | OTHER |  | NL |  | NL | MET fusion 2C.1 | 2C.1 | 2C.1 |
| SP86118 | Thyroid | OTHER |  | NL |  | NL |  | NL | NL |
| SP86130 | Thyroid | OTHER |  | NL |  | NL | ALK fusion 2C.1 | 2C.1 | 2C.1 |
| SP86306 | Thyroid | OTHER | HRAS 2C.1 | 2C.1 |  | NL |  | NL | 2C.1 |
| SP86425 | Thyroid | OTHER | BRAF 1A.1 | 1A.1 |  | NL |  | NL | 1A.1 |
| SP8660 | Breast | BREAST | TP53 2C.2 | 2C.2 | BRCA2 loss 2C.1 | 2C.1 |  | NL | 2C.1 |
| SP86660 | Thyroid | OTHER | AKT1 2C.2 , BRAF p.V600E 1A.1 , PPM1D p.T483Kfs*4 3 | 1A.1 |  | NL |  | NL | 1A.1 |
| SP86775 | Thyroid | OTHER |  | NL |  | NL | RET fusion 2C.1 | 2C.1 | 2C.1 |
| SP86836 | Thyroid | OTHER |  | NL |  | NL |  | NL | NL |
| SP86929 | Thyroid | OTHER | BRAF 1A.1 | 1A.1 |  | NL |  | NL | 1A.1 |
| SP86989 | Thyroid | OTHER |  | NL |  | NL | RET fusion 1A.1 | 1A.1 | 1A.1 |
| SP87099 | Thyroid | OTHER | ARID2 3 | 3 |  | NL |  | NL | 3 |
| SP87337 | Thyroid | OTHER |  | NL |  | NL | RET fusion 1A.1 | 1A.1 | 1A.1 |
| SP87434 | Thyroid | OTHER |  | NL |  | NL |  | NL | NL |
| SP87446 | Thyroid | OTHER | BRAF 1A.1 | 1A.1 |  | NL |  | NL | 1A.1 |
| SP87534 | Thyroid | OTHER | NRAS 2C.1 , TP53 p.T125T 2C.2 | 2C.1 |  | NL |  | NL | 2C.1 |
| SP87582 | Thyroid | OTHER |  | NL |  | NL | RET fusion 1A.1 | 1A.1 | 1A.1 |
| SP87675 | Thyroid | OTHER | TSHR 3 | 3 |  | NL |  | NL | 3 |
| SP87903 | Thyroid | OTHER |  | NL |  | NL | ALK fusion 2C.1 | 2C.1 | 2C.1 |
| SP8795 | Breast | BREAST | FANCD2 2C.1 , TP53 p.R248Q 2C.2 | 2C.1 | DDR2 amp 2C.2 , MCL1 amp 2D , MYC amp 2C.2 | 2C.2 |  | NL | 2C.1 |
| SP88050 | Thyroid | OTHER | BRAF 1A.1 | 1A.1 |  | NL |  | NL | 1A.1 |
| SP88098 | Thyroid | OTHER | NUP93 3 | 3 |  | NL |  | NL | 3 |
| SP88158 | Thyroid | OTHER |  | NL |  | NL | NTRK3 fusion 1A.1 | 1A.1 | 1A.1 |
| SP8831 | Breast | BREAST | FBXW7 2C.2 , TP53 p.R110Sfs*14 2C.2 | 2C.2 | FGFR1 amp 2C.1 , FLT4 amp 2C.2 , H3F3B amp 3 , MCL1 amp 2D , MYC amp 2C.2 , SMO amp 2C.1 | 2C.1 |  | NL | 2C.1 |
| SP88322 | Thyroid | OTHER | PPM1D 3 | 3 |  | NL |  | NL | 3 |
| SP88593 | Thyroid | OTHER | DNMT3A 3 | 3 |  | NL |  | NL | 3 |
| SP88757 | Thyroid | OTHER |  | NL |  | NL |  | NL | NL |
| SP88776 | Thyroid | OTHER |  | NL |  | NL |  | NL | NL |
| SP8891 | Breast | BREAST | ATRX 2C.2 , PIK3CA p.E453K 1A.1 | 1A.1 | FAM135B amp 3 , MYC amp 2C.2 | 2C.2 |  | NL | 1A.1 |
| SP89090 | Thyroid | OTHER |  | NL |  | NL |  | NL | NL |
| SP89245 | Thyroid | OTHER | APC 2C.2 | 2C.2 |  | NL |  | NL | 2C.2 |
| SP89291 | Thyroid | OTHER |  | NL |  | NL | BRAF fusion 2C.1 | 2C.1 | 2C.1 |
| SP89389 | Uterus | OTHER | FAT1 3 , PPP2R1A p.S256F 2C.1 , TP53 p.Q331* 2C.2 | 2C.1 | CCNE1 amp 2C.2 , EGFR amp 2C.1 , EGFR amp 2C.1 , MCL1 amp 2D | 2C.1 |  | NL | 2C.1 |
| SP89443 | Uterus | OTHER | PPP2R1A 2C.1 , TP53 p.P151S 2C.2 | 2C.1 | PIK3CA amp 2C.1 | 2C.1 |  | NL | 2C.1 |
| SP89519 | Uterus | OTHER | PTEN 2C.1 , PTEN p.R130P 2C.1 | 2C.1 |  | NL |  | NL | 2C.1 |
| SP89651 | Uterus | OTHER | PIK3CA 2C.1 , SMC1A p.A178V 3 , TP53 p.G262V 2C.2 | 2C.1 |  | NL |  | NL | 2C.1 |
| SP89687 | Uterus | OTHER | NRAS 2C.1 , PIK3CA p.H1047L 2C.1 , TP53 p.R248W 2C.2 | 2C.1 | RAF1 amp 2C.2 | 2C.2 |  | NL | 2C.1 |
| SP8987 | Breast | BREAST | MLL2 3 , NOTCH2 p.P2189Ffs*34 2C.2 , TP53 p.T211Lfs*36 2C.2 | 2C.2 | CDKN2A loss 2C.1 , MTAP loss 3 , PBRM1 loss 2D , PTEN loss 2C.1 | 2C.1 |  | NL | 2C.1 |
| SP89909 | Uterus | OTHER | ARID1A 2C.2 , ATM p.V2830D 2C.1 , DDR2 p.W756C 2C.2 , HDAC2 p.V12Sfs*20 3 , MAP3K1 p.T947Nfs*56 2C.2 , MSH3 p.K383Rfs*32 2C.2 , PIK3CA p.E542K 2C.1 , PTEN p.C136Mfs*8 2C.1 | 2C.1 |  | NL |  | NL | 2C.1 |
| SP89957 | Uterus | OTHER | PIK3CA 2C.1 , PIK3R1 p.R348* 2C.2 | 2C.1 | CCNE1 amp 2C.2 , MYC amp 2C.2 | 2C.2 |  | NL | 2C.1 |
| SP90125 | Uterus | OTHER | SMC1A 3 , TP53 p.G266V 2C.2 | 2C.2 | CCNE1 amp 2C.2 , MYC amp 2C.2 | 2C.2 |  | NL | 2C.2 |
| SP90245 | Uterus | OTHER | ARHGAP35 3 , ATM p.R1466P 2C.1 , GATA3 p.P163L 2D , MGA p.E2438* 3 , PIK3R1 p.R386* 2C.2 , TP53 p.C277dupC 2C.2 | 2C.1 | AKT2 amp 2C.2 , CCNE1 amp 2C.2 , ERBB2 amp 2C.1 , FGFR3 amp 2C.1 , MPL amp 2C.2 | 2C.1 |  | NL | 2C.1 |
| SP90269 | Uterus | OTHER | PIK3CA 2C.1 , TP53 p.R306* 2C.2 | 2C.1 | MCL1 amp 2D | 2D |  | NL | 2C.1 |
| SP90503 | Uterus | OTHER | FGFR2 2C.1 , PPP2R1A p.P179R 2C.1 , TP53 p.S241C 2C.2 | 2C.1 | CTNND2 amp 3 , FAM135B amp 3 , IKBKB amp 3 , IL7R amp 3 , MEF2B amp 3 , MYC amp 2C.2 , PIK3R2 amp 2C.2 , PRKACA amp 3 , RICTOR amp 2C.2 , TERT amp 3 | 2C.2 |  | NL | 2C.1 |
| SP90629 | Uterus | OTHER | ARHGAP35 3 , FBXW7 p.R505G 2C.2 , MLL3 p.R199Yfs*15 3 , MLL3 p.R199Hfs*15 3 , PIK3CA p.E545K 2C.1 , PIK3CA p.E453Q 2C.1 , SETD2 p.R1496Q 2D , TP53 p.R249S 2C.2 , WWOX p.V190L 3 | 2C.1 | BCL6 amp 2C.2 , ERBB2 amp 2C.1 , ERBB2 amp 2C.1 , ERBB3 amp 2C.2 , PIK3CA amp 2C.1 , STAT3 amp 2C.2 | 2C.1 |  | NL | 2C.1 |
| SP90725 | Uterus | OTHER | AKT1 2C.2 , ARID1A p.E1733* 2C.2 , BCOR p.N1459S 3 , BRCA2 p.S1882* 2C.1 , CTCF p.E616*fs*1 3 , CTNNB1 p.G34R 2C.2 , MTOR p.D2512Y 2C.1 | 2C.1 |  | NL |  | NL | 2C.1 |
[truncated: 66,561 more chars]
